# Improving readability of layperson abstracts and summaries in oncology using task-specific large language model powered tool: results from the BRIDGE-AI 7 study

**DOI:** 10.1093/jamiaopen/ooag082

**Published:** 2026-06-25

**Authors:** Aalamnoor S Pannu, Ilicia Cano, Ethan Layne, Gus Miranda, Jie Cai, Conner Ganjavi, Vasileios Magoulianitis, Karanvir Gill, Gerhard Fuchs, Mihir Desai, Inderbir Gill, Giovanni E Cacciamani

**Affiliations:** USC Institute of Urology and Catherine and Joseph Aresty Department of Urology, Keck School of Medicine, University of Southern California, Los Angeles, CA 90033, United States; Artificial Intelligence Center at USC Urology, Los Angeles, CA 90033, United States; USC Institute of Urology and Catherine and Joseph Aresty Department of Urology, Keck School of Medicine, University of Southern California, Los Angeles, CA 90033, United States; Artificial Intelligence Center at USC Urology, Los Angeles, CA 90033, United States; USC Institute of Urology and Catherine and Joseph Aresty Department of Urology, Keck School of Medicine, University of Southern California, Los Angeles, CA 90033, United States; Artificial Intelligence Center at USC Urology, Los Angeles, CA 90033, United States; USC Institute of Urology and Catherine and Joseph Aresty Department of Urology, Keck School of Medicine, University of Southern California, Los Angeles, CA 90033, United States; Artificial Intelligence Center at USC Urology, Los Angeles, CA 90033, United States; USC Institute of Urology and Catherine and Joseph Aresty Department of Urology, Keck School of Medicine, University of Southern California, Los Angeles, CA 90033, United States; Artificial Intelligence Center at USC Urology, Los Angeles, CA 90033, United States; USC Institute of Urology and Catherine and Joseph Aresty Department of Urology, Keck School of Medicine, University of Southern California, Los Angeles, CA 90033, United States; Artificial Intelligence Center at USC Urology, Los Angeles, CA 90033, United States; USC Institute of Urology and Catherine and Joseph Aresty Department of Urology, Keck School of Medicine, University of Southern California, Los Angeles, CA 90033, United States; Artificial Intelligence Center at USC Urology, Los Angeles, CA 90033, United States; USC Institute of Urology and Catherine and Joseph Aresty Department of Urology, Keck School of Medicine, University of Southern California, Los Angeles, CA 90033, United States; Artificial Intelligence Center at USC Urology, Los Angeles, CA 90033, United States; USC Institute of Urology and Catherine and Joseph Aresty Department of Urology, Keck School of Medicine, University of Southern California, Los Angeles, CA 90033, United States; Artificial Intelligence Center at USC Urology, Los Angeles, CA 90033, United States; USC Institute of Urology and Catherine and Joseph Aresty Department of Urology, Keck School of Medicine, University of Southern California, Los Angeles, CA 90033, United States; Artificial Intelligence Center at USC Urology, Los Angeles, CA 90033, United States; USC Institute of Urology and Catherine and Joseph Aresty Department of Urology, Keck School of Medicine, University of Southern California, Los Angeles, CA 90033, United States; Artificial Intelligence Center at USC Urology, Los Angeles, CA 90033, United States; USC Institute of Urology and Catherine and Joseph Aresty Department of Urology, Keck School of Medicine, University of Southern California, Los Angeles, CA 90033, United States; Artificial Intelligence Center at USC Urology, Los Angeles, CA 90033, United States

**Keywords:** GPT, generative AI, large language models, artificial intelligence, GPT-5, lay summary, Pub2Post, readability

## Abstract

**Objectives:**

To compare the performance of task-specific generative AI, with general-purpose large language models (LLMs) in generating more readable lay abstracts and summaries (LASs) of Oncology research.

**Materials and Methods:**

Twenty-five randomly selected abstracts from the top 5 journals in Oncology were processed into LASs using a task specific LLM-powered tool (Pub2Post) and 5 general-purpose LLMs (ChatGPT-5, Claude, Gemini, DeepSeek and Grok). Two prompting strategies (Specific and Generic) were applied. Consistency was tested across 3 outputs, producing a total of 825 LASs. Readability-scores and text-metrics were calculated. The “best test” per model was selected based on lowest SMOG Index, which was subsequently used to compare the 6 GAI platforms. Comparisons were performed using Kruskal-Wallis tests, with significance set at *P* < .05.

**Results:**

All the platforms demonstrated consistent intra-model outputs across triplicate generations (all *P* > .05). However, inter-model comparisons revealed significant differences (all *P* < .001) with Pub2Post outperforming the LLMs, across the 2 prompting styles, demonstrating superior readability-scores (FRES 82.3; FKGL 5.2; GFS 6.6; SI 4.4; CLI 10.4; ARI 6.2) with longer outputs (27 sentences; 388 words) and fewer complex-words (3.7%). The general-purpose LLMs generated shorter, denser outputs (4-9 sentences; 81-156 words) with higher grade-levels (FRES 38.0-61.6; FKGL 9.6-13.4; GFS 10.6-15.9; SI 7.6-11.2; CLI 13.4-17.0; ARI 11.0-15.2).

**Discussion and Conclusion:**

Task-specific GAI powered tools (Pub2Post) generated consistently more readable LASs compared to 5 commercially available LLMs, regardless of prompting strategy. These findings highlight the value of purpose-built GAI tools for enhancing public understanding and accessibility of oncology research, with implications for improving patient-education.

## Background

Access to reliable and understandable health information remains a major public concern, with online searches for medical content consistently ranking among the most frequent internet queries.^1–3^ In recent years, the deployment of Generative Artificial Intelligence (GAI) with Large Language Models (LLMs), has shown the potential application in healthcare,^4–7^ specifically in transforming the way health information is delivered to the public.^8–12^ These models produce near-human like contents and give the appearance of easily readable and readily understandable information.^13^

Despite their widespread use, questions remain whether the information generated by these GAI platforms are readable and understandable by the public while maintaining accuracy. Readability is a paramount metric when it comes to the dissemination of medical information, for which a level of Grade 6-8 is considered suitable.^14–16^ However, studies revealed that medical information whether in print or online often exceeds these levels.^17–20^ Addressing this issue is especially important for oncological literature, as patients with oncological conditions and their family members often rely on online searches to find out more information about their condition, treatments, and prognosis.^21^ The potential of GAI output variability based on prompting dramatically changes the way information can be generated, therefore making it difficult to rely on these AI systems in generating lay person abstracts and summaries.^9^ On the other hand, task-specific GAI powered tools like Pub2Post—a GAI-powered tool crafted specifically to create readable contents tailored for lay people from scholar articles—have shown higher consistency of responses in providing readable, accurate and complete lay people tailored medical contents.^9–11^ However, the readability scores of AI generated lay person summaries from scientific abstracts of domain-specific workflows have not been compared across different general GAI platforms.

## Objectives

The aim of this study is to compare task-specific GAI powered tool and general LLMs in generating patient-tailored abstracts and summaries in oncology. In particular, we seek to evaluate whether the task-specific generative AI tool consistently produces more readable lay summaries of oncology research compared to commercially available large language models. A central objective is to assess both the readability and the consistency of performance across different outputs.

## Materials and methods

### Selection of the source and generation of LASs

Original articles and review papers were retrieved from Web of Science (https://www.webofscience.com/) restricted to publications between July 1^st^, 2024 and July 1^st^, 2025 in the top 5 oncology journals: *Journal of Clinical Oncology*, *Nature Reviews Cancer*, *Cancer Research*, *The Lancet Oncology*, and *Cancer Cell*. These journals were chosen based on their H-index ranking according to SCImago Journal Rank (https://www.scimagojr.com/). A random number generator was then applied to select 5 abstracts from each journal, yielding a total of 25 abstracts for analysis. This study is part of the Bridging Readable and Informative Dissemination with GenerativE Artificial Intelligence (BRIDGE-AI) initiative.^22^

A task-specific GAI-powered tool (www.pub2post.com), already validated to create lay abstract and summaries (LASs) from medical literature, was used to generate lay abstracts and summaries (LASs) that are accurate and suitable for public readability, targeting a reading level of grate 6-8 are required by the current recommendations.^14^ This tool has been shown to reliably produce layperson summaries from scholarly literature while ensuring compliance with established guidelines for good lay summary practices, literacy recommendations, and clinical trial lay summary standards.^12^^,^^14^^,^^18^^,^^23^ The outputs generated by Pub2Post were then compared with those produced by general large language models, including ChatGPT-5 (OpenAI, San Francisco, CA, USA), Google Gemini (Alphabet Inc., Mountain View, CA, USA), Meta AI (Meta, Menlo Park, CA, USA), xAI/Grok (X Corp, Bastrop, TX, USA), and DeepSeek (DeepSeek, Hangzhou, China). All the generations were made between 07-31-24 and 08-14-25 To evaluate performance, 2 prompts were used to generate layperson summaries from the general LLMs: “Specific Prompt” developed for summarizing medical contents from scientific abstracts^13^ and a “Generic Prompt,” which was developed through an internal audit conducted by the authors, designed to simulate typical user interactions with general LLMs when attempting to simplify and digest complex medical content. Details of both prompts are provided in the supplementary materials (Table S1) and each of the analysis reported below performed for each of the 2 prompts tested, separately.

### Readability metric assessed

Readability scores (RSs), grade-level indicators (GLIs), and text metrics (TMs) were calculated for LASs using a previously validated web-based tool (www.WebFX.com) as previously reported.^9^^,^^10^^,^^12^^,^^17^^,^^18^ Standardized readability measures (RSs and GLIs) included the Flesch–Kincaid Reading Ease Score (FRES), Gunning Fog Score (GFS), SMOG Index (SI), Coleman–Liau Index (CLI), and Automated Readability Index (ARI). Text metrics (TMs) were used to characterize structural features of the text and included total word count, sentence count, mean words per sentence, mean syllables per word, number of complex words, and percentage of complex words. The FRES and FKGL analyze sentence length and syllables per word, whereas the GFS measures sentence length alongside complex words (typically those with 3 or more syllables). The CLI and ARI base their scores on letters per word and sentence length.^24^ The SMOG formula, specifically designed for healthcare materials, assesses complex words across a 30-sentence sample from the text, using a correction factor for shorter documents.^25^^,^^26^

The recommended reading age for healthcare information is 11 to 12 years old.^27^ To achieve this, texts should target a score of 6.9 on the FKGL, GF, CLI, and SMOG tests, or a score of 80 on the 100-point FRES scale (where higher scores indicate easier reading).

### Consistency of the output and best test selection

To assess output consistency, each LASs was generated in triplicate, yielding 3 versions (T1, T2, and T3) per prompt. In total, 825 LASs were produced, comprising 25 outputs per generation (T1-T3) for each of the 6 generative AI (GAI) platforms across 2 prompts. RSs, GLIs and TMs were calculated. Additional details on the GAI-generated LASs are available in the supplementary materials (Table S2). After generating T1-T3, the LAS with the lowest SI for each abstract was selected for further analysis (the “best test”). SI was prioritized to identify the most readable LAS across GAI platforms, as it combines sentence length and word complexity into a single readability score and has been widely applied in medical literature.^25^ In cases where 2 LASs yielded identical SI values, the FRES was used as a tie-breaker. The best LASs, defined by the lowest SI (or by FRES in the event of a tie), were subsequently used for all comparative analyses.

#### Statistical analysis

Differences in RSs and TMs among LASs generated across the 6 GAI platforms were assessed using the Kruskal-Wallis test. Consistency across T1-T3 was evaluated with paired statistical tests, including repeated-measures analysis for readability metrics. Comparisons among GAI platforms were performed using the “best” LAS from each, with the top-performing platform identified based on superior readability metrics. Mean differences (MD), 95% CIs, and percentage changes (%Δ) for RSs, GLIs, and TMs were calculated between the best-performing platform (“winner”) against all the all others. Statistical analyses were conducted in SAS. with significance set at *P* value <.05.

## Results

### Readability and text metrics consistency of GAI-generated LASs

The consistency analysis of GAI-generated LASs showed consistency in median (Lower and Upper Quartiles) RSs, GLIs, and TMs across Tests 1, 2, and 3, with no statistically significant differences observed (Tables S5 and S6; all *P*-values > .05) between the 3 tests for any of the 6 GAI platforms compared. This held true for Specific Prompt^13^ as well as Generic Prompt. The comparative analysis of the median, showed that across all readability indices, results from the 3 repeated trials (Tests 1, 2, and 3) were consistent within each system. For Pub2Post, FRES remained between 78.8 and 80.4 across both prompting strategies, with corresponding FKGL of 5.7-6.0, GFS of 7.2-7.5, SI of 4.7-5.0, and ARI scores of 7.0-7.2. This stability was observed across all 3 repetitions, with no statistically significant intra-model differences detected (Tables S5 and S6; all *P* > .05). Comparable consistency was also observed for the other LLMs. ChatGPT-5, Grok, Gemini, DeepSeek, and Claude each produced outputs with FKGL in the 9.9-15.1 range, GFS and SI above 11 and 8 respectively, and ARI values between 12.1 and 16.9. Scores for these systems showed minimal variation between Test 1, Test 2, and Test 3, and no significant intra-model differences were identified across repetitions (all *P* > .05). Structural characteristics of outputs evaluated across the task specific and the general LLMs under 2 prompting strategies, with each output tested in triplicate. Across both prompting conditions, intra-model consistency was high, with no statistically significant variation between Test 1, 2, and 3 for any metric (Tables S5 and S6; all *P* > .05).

Pub2Post consistently generated the longest outputs, averaging 26-27 sentences and 365-394 words, compared to ChatGPT-5, Grok, Gemini, DeepSeek, and Claude, which produced shorter texts. Under publication-based prompting, the latter systems averaged 6-9 sentences and 118-161 words, while with generic prompting their outputs decreased further to 4-5 sentences and 80-110 words. Lexical complexity measures also remained stable across the 3 tests. Pub2Post outputs contained 15-18 complex words (4-5% of total words), while the other systems produced higher proportions of complex words, ranging from 13 to 20 and accounting for 11-20% of their text. Sentence-level metrics showed similar patterns: Pub2Post maintained an average of 14-15 words per sentence and 1.32-1.34 syllables per word, whereas the other systems produced longer and denser sentences (18-27 words per sentence, 1.67-1.78 syllables per word). Details are reported in supplementary materials (Table S7; all *P* > .05).

### Readability and text metrics comparison of GAI-generated LASs

**Figure 1.**
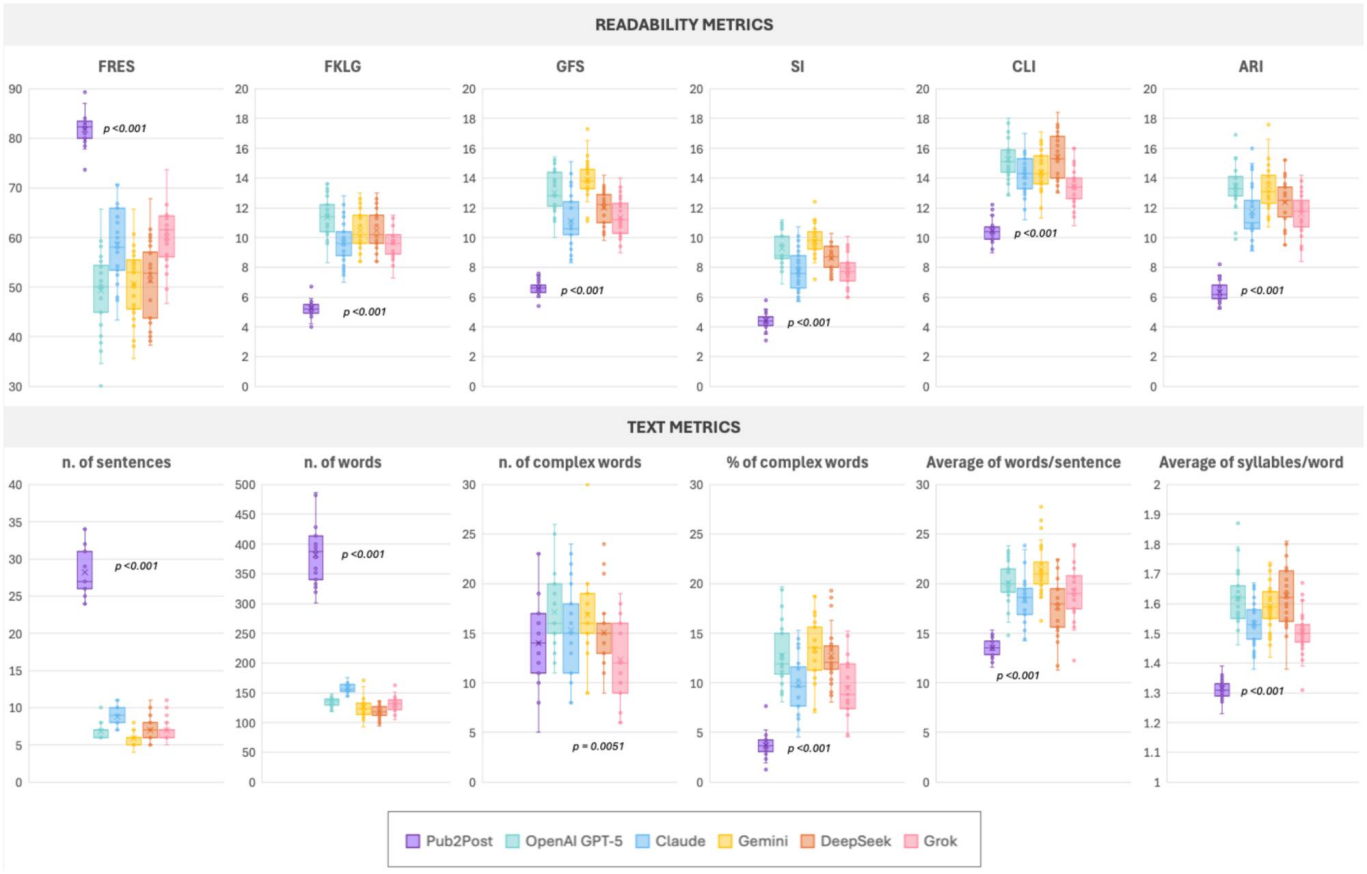
Comparative analysis of readability and text complexity metrics across Task Specific GAI powered tool (Pub2Post) and 5 General Purpose LLM (OpenAI GPT-5, Claude, Gemini, DeepSeek, and Grok) prompted with specific prompt. The upper panel reports 6 readability indices: Flesch Reading Ease Score (FRES), Flesch–Kincaid Grade Level (FKLG), Gunning Fog Score (GFS), Smog Index (SI), Coleman–Liau Index (CLI), and Automated Readability Index (ARI). The lower panel presents text-level measures, including the number of sentences, number of words, number and percentage of complex words, mean words per sentence, and mean syllables per word. Pub2Post exhibits markedly higher readability scores and longer outputs, with significant differences indicated (*P* < .001 unless otherwise specified).

**Figure 2.**
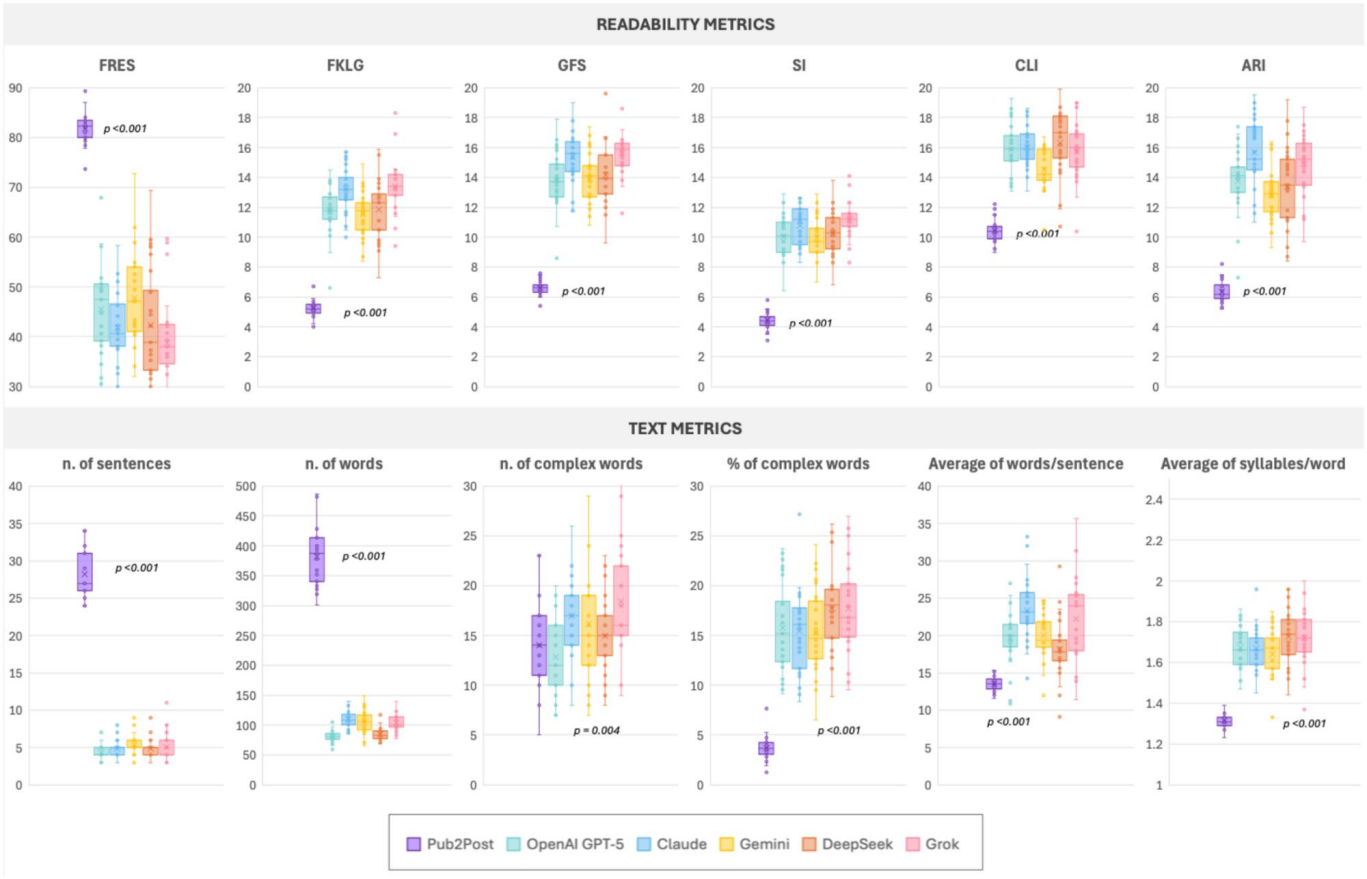
Comparative analysis of readability and text complexity metrics across Task Specific GAI powered tool (Pub2Post) and 5 General Purpose LLM (OpenAI GPT-5, Claude, Gemini, DeepSeek, and Grok) prompted with generic prompt. The upper panel reports 6 readability indices: Flesch Reading Ease Score (FRES), Flesch–Kincaid Grade Level (FKLG), Gunning Fog Score (GFS), Smog Index (SI), Coleman–Liau Index (CLI), and Automated Readability Index (ARI). The lower panel presents text-level measures, including the number of sentences, number of words, number and percentage of complex words, mean words per sentence, and mean syllables per word. Pub2Post exhibits markedly higher readability scores and longer outputs, with significant differences indicated (*P* < .001 unless otherwise specified).

The best test for each GAI model-defined as the output with the lowest SI among the 3 tests was then used for the comparisons among the 6 different GAI software’s. When the best tests were compared across systems, statistically significant differences were observed between systems for all readability and text indices under both prompting strategies (Figures 1 and 2, Tables S8 and S9; *P* < .0001, except complex words: *P* = .0051 for Specific Prompt; *P* = .004 for Generic Prompt). Compared across both prompting approaches, Pub2Post consistently produced the most readable text. FRES remained >82, with corresponding FKGL of 5.2 (4.9-5.5). GFS (6.6), SI (4.4), CLI (10.4), and ARI (6.2) values also reflected readability at a middle school level. By contrast, ChatGPT-5, Grok, Gemini, DeepSeek, and Claude generated significantly more complex outputs. For Specific Prompt, these systems produced FKGL between 9.6 and 11.4, GFS of 10.6-13.8, SI of 7.6-9.8, and ARI of 11.0-13.3. Under Generic Prompt, complexity increased further, with FKGL ranging from 11.8 to 13.4, GFS 13.7-15.9, SI 9.7-11.2, and ARI values 12.9-15.2. Text indices analysis revealed a consistent pattern. Pub2Post outputs were longer, with 27 sentences and ∼388 words, containing fewer complex words (14.0; 3.7% of total), shorter sentences (13.5 words), and lower syllabic density (1.31 syllables per word). In contrast, the other systems generated markedly shorter outputs (4-9 sentences, 81-156 words) that were more lexically and syntactically dense, with higher proportions of complex words (12-17.0; 8-18%), longer sentences (18-24 words), and higher syllable counts per word (1.50-1.74).

### Comparison of task specific vs general-purpose LLM best tests

**Figure 3.**
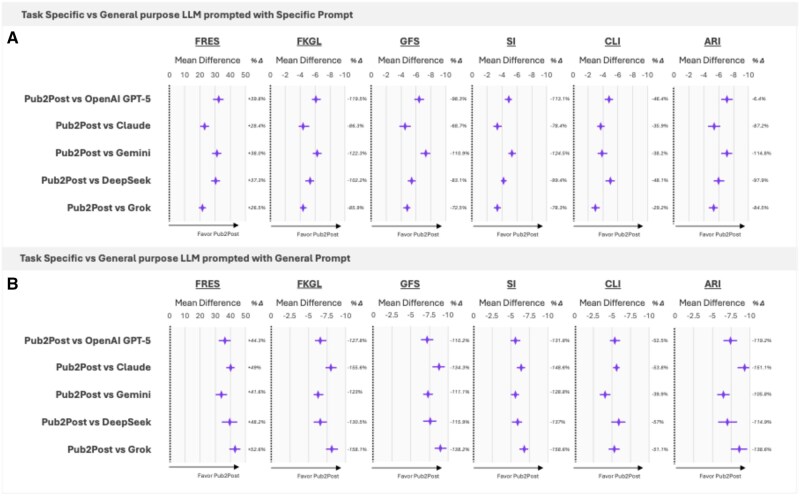
Pairwise comparisons of readability metrics between Task Specific GAI powered tool (Pub2Post) and 5 General Purpose LLM (OpenAI GPT-5, Claude, Gemini, DeepSeek, and Grok). Panels display mean differences and percentage changes (Δ%) in Flesch Reading Ease Score (FRES), Flesch–Kincaid Grade Level (FKGL), Gunning Fog Score (GFS), Smog Index (SI), Coleman–Liau Index (CLI), and Automated Readability Index (ARI). (A) Comparisons with models prompted using task-specific instructions. (B) Comparisons with models prompted using a general instruction. The direction of the effect indicates the system favored, with rightward shifts denoting advantages for Pub2Post. Diamonds represent mean differences, while horizontal lines denote 95% confidence intervals.

Across both the specific prompt (Figure 3a, Table S10) and generic prompt (Figure 3b, Table S11) conditions, Pub2Post outputs showed statistically significant differences compared with all comparator systems (all *P* < .05). Pub2Post consistently produced more readable text, with substantially higher FRES scores than GPT-5, Claude, Gemini, DeepSeek, and Grok. In the specific prompt condition (Figure 3a, Table S10), gains ranged from +26.5% to +39.8%; under the generic prompt condition (Figure 3b, Table S11), they were even larger, from +41.6% to +52.6%. Correspondingly, grade-level indices (FKGL, GFS, SI, CLI, ARI) were consistently and significantly lower, with mean differences ranging from -3.4 to -9.3 (-78% to −159%), indicating simpler sentence structures and reduced reading difficulty. Regarding text metrics, Pub2Post produced substantially longer outputs, with 224.0-300.4 more words (+58-79%) and 19-24 additional sentences (+69-85%) relative to comparators. Despite the increased length, sentences were shorter (reductions of 4-10 words, -31-73%), and words were phonetically simpler, with average syllables per word reduced by 14-31%. In terms of readability metrics, the absolute counts of complex words showed reductions versus some models but not all, with several nonsignificant differences (eg, vs Claude or DeepSeek under the specific prompt, or vs GPT-5 and Gemini under the generic prompt). However, the *proportion* of complex words was consistently and dramatically lower across all conditions and comparators, with reductions ranging from -178% to -433%.

## Discussion

The present study assessed the differences between a task-specific GAI software and 5 commercially available LLMs in producing LASs for oncology research. Pub2Post, the task-specific GAI powered tool consistently produced more readable LASs, as measured by objective metrics, compared to 5 commercially available LLMs. These findings highlight the potential of a GAI system specifically designed for generating more accessible layperson summaries of oncology contents which has the potential to improve public understanding and trust in medical information. As we showed in the present study task-specific GAI is preferable to general-purpose models for medical communication because it minimizes output variability and delivers more consistent, reliable results. General GAI systems are inherently stochastic and highly sensitive to prompting, leading to fluctuations in content that can compromise accuracy and reproducibility. In contrast, task-specific tools such as Pub2Post are designed with defined objectives, standardized prompts, and structured workflows tailored to tasks like generating lay summaries. By constraining how outputs are produced, thanks to well validated and tailored prompting in the back-end, these systems reduce randomness and consistently generate clearer, more accurate, and complete patient-facing content, making them a more dependable choice than general-purpose AI.

This study is part of the BRIDGE-AI studies initiative, which have shown that GAI-generated LASs scientific content, especially medical topics, are often more readable than original versions while maintaining high-quality information.^9^^,^^28^

The analysis of RSs, GLIs, and TMs revealed strong consistency in outputs across 3 tests for all 6 LLMs, with no statistically significant intra-test differences observed (all *P *> .05). This reproducibility held true under both prompting strategies, aligning with previous studies that demonstrated reliability and stability across multiple generations of GAI software.

Despite this consistency, clear differences emerged between the task-specific model and the general-purpose LLMs. Pub2Post consistently produced longer outputs (26-27 sentences; 365-394 words) with a lower proportion of complex words (4-5%) and higher readability scores (FRES 78.8-80.4; FKGL 5.7-6.0; SI 4.7-5.0) across all tests. In contrast, ChatGPT-5, Grok, Gemini, DeepSeek, and Claude generated shorter summaries (4-9 sentences; 80-161 words) with denser sentence structures (18-27 words per sentence), a higher proportion of complex words (11-20%), and grade levels in the 9.9-15.1 range. These patterns remained stable across all 3 repetitions, underscoring intra-test consistency while highlighting systematic differences between platforms.^29^

Together, these findings confirm that LLM outputs are reproducible across repeated trials and prompting strategies, but readability and structural characteristics remain GAI model-and prompt dependent. The stability observed strengthens confidence in comparative evaluations of GAI platforms, showing that differences between systems reflect systematic behaviors rather than random variability.

Between the 2 prompts used for the 5 general LLMs that were tested, the Specific Prompt yielded a consistently higher word count and sentence average compared to the Generic Prompt across multiple tests, suggesting more detailed outputs were generated under the Specific Prompt. This is potentially related to the specific crafting of the Specific Prompt for medical content, although still resulting in a readability performance below the threshold required. In fact, in the publication the extended prompt showed median FRES of 40.9 yet still failed to reach the minimum readability threshold recommended for plain-language medical communication which is reported to be above 70 to ensure readability of laypeople.^25^^,^^30^ The findings, of the present study, even doing 3 different tests using showed consistency of the specific prompting even when used in different LLMs with low readability compared to the minimum threshold required, confirming the previous findings^13^ This coincides with the idea that commercially available LLMs can produce more desirable outputs when specific prompt engineering is utilized.^31^

The results of the best comparison demonstrate the potential of task-specific GAI powered tool to consistently produce more readable lay abstracts/summaries. Across both prompting styles the task-specific LLM, which does not require user prompting, consistently achieved superior readability metrics. This was affirmed by a median SI, specifically tailored for measuring the readability of the medical contents of 4.4 and compared to commercially available large language models which fall in the range of 7.6-9.8 for Specific Prompt and 9.7-11.2 for Generic Prompt (*P* < .001). The established guidelines for effective lay summaries, literacy standards, and clinical trial summary requirements for the public, where a SI of 6-8 is considered favorable,^25^^,^^30^ which aligns with the results of the task-specific GAI tool. The higher readability of the outputs from Pub2Post likely stems from its task-specific design, which optimizes content for lay audiences by reducing the percentage of complex words, and producing longer outputs. In contrast, LLMs like ChatGPT 5, Claude, Gemini, DeepSeek and Grok exhibited higher percentages of complex words and shorter sentences, implying that even though the summaries were shorter they were not necessarily easier to read.

The practical implications of these results can be significant, particularly for patient education in oncology, as more accessible summaries can enhance understanding and trust in research findings.^32^ Task-specific GAI powered tools-like Pub2Post’s, could serve as a valuable tool for translating complex medical information into formats suitable for a wide range of audiences while the accuracy, completeness and clarity of the information’s is ensured^9^^,^^12^^,^^17^

Providing patients with information that is readable, understandable, and accurate can be difficult when health information is always being updated.^33^ For medical content specifically, there is a persistent lack of readability for the public.^17^ As previously described, health information on the internet can generally be classified as readable but not certified or difficult to read but certified.^34^^,^^35^ The dilemma faced by many is how to find health or medical information that is both readable and certified, as integrating the 2 is often difficult. Professionals in the health information sphere, when assisted by a tool that gives real-time feedback on readability, still require a large amount of time to write a more readable version of health information.^36^ GAI can help alleviate the time burden of writing readable medical information when careful closed prompting strategies are used to ensure consistency and the output is verified by assessing objective test metrics like SI.

The study is limited by the small sample size of articles and the focus on oncology medical abstracts that were used as the inputs to both a task-specific GAI tool and multi-purpose GAI models. To enhance the generalizability of our results more studies are needed to compare across a larger set of medical abstracts that cover all medical disciplines. Our study did not involve humans to assess the understandability or perception of the information generated by task specific tools as compared to general-purpose GAI models. This assessment was already performed showing consistent accuracy of the information provided, while improving the readability and understandability.^12^ Additionally, only 2 prompting strategies were tested for the general-purpose LLMs. While the Specific Prompt and Generic Prompt were designed to reflect structured medical prompting and typical user behavior, they cannot represent the full range of prompt variability seen in real-world use. More extensive prompt testing, including iterative prompting, patient-generated prompts, and expert-engineered prompts, would better characterize how prompt design influences readability and consistency. In our study, outputs were generated during a defined time period, and commercially available LLMs are frequently updated; therefore, results may change as model versions, safety layers, and response behaviors evolve. Finally, the study was limited to English-language oncology abstracts, and the findings may not apply to other languages, cultural contexts, or populations with different literacy needs. Future research should evaluate multilingual outputs, culturally adapted summaries, and the performance of task-specific GAI tools in diverse patient populations.

## Conclusion

Task-specific GAI-powered model produced consistently more readable LASs, based on objective metrics, compared to 5 commercially available LLMs. These results highlight the prospects of using a GAI tool specifically designed for the task of producing more readable LASs of oncology abstracts, with the potential to enhance public understanding and trust in medical information.

## Supplementary Material

ooag082_Supplementary_Data

## Data Availability

The data underlying this study are available within the article and its supplementary materials. The readability score dataset generated and analyzed during the current study has been deposited in the Dryad repository and is available at: 10.5061/dryad.2z34tmq1z.
