# Supplementary material for: Improving readability of layperson abstracts and summaries in oncology using task-specific large language model powered tool: results from the BRIDGE-AI 7 study
Source: JAMIA Open. 2026 Jun 25;9(3):ooag082. doi: 10.1093/jamiaopen/ooag082 (PMC13296995; doi:10.1093/jamiaopen/ooag082)
Supplement: ooag082_Supplementary_Data [file ooag082_supplementary_data.docx]

**Supplementary Materials**

**Improving Readability of Layperson Abstracts and Summaries in Oncology Using Task-Specific Large Language Model Powered Tool: Results from the BRIDGE-AI 7 Study**

Aalamnoor Pannu, MD^1,2^, Ilicia Cano, BS^1,2^,, Ethan Layne, BS^1,2^,, Gus Miranda, BS^1,2^,, Jie Cai, MS ^1,2^,,

Conner Ganjavi, BS^1,2^,, Vasileios Magoulianitis, PhD^1,2^, Karanvir Gill, MS^1,2^,, Gerhard Fuchs, MD^1,2^,, Mihir Desai, MD^1,2^,, Inderbir Gill, MD^1,2^,, Giovanni E. Cacciamani, MD^1,2^,

1. *USC Institute of Urology and Catherine and Joseph Aresty Department of Urology, Keck School of Medicine, University of Southern California, Los Angeles, CA, USA;*
2. *Artificial Intelligence Center at USC Urology*

**Table 1s**: Prompts employed to generate layperson-friendly abstracts and summaries using generic LLMs.

**Table 2s:** GAI generated Lay abstracts and summaries (Test 1-3), using a “Specific Prompt” in the General purpose LLMs.

**Table 3s:** GAI generated Lay abstracts and summaries (Test 1-3), using a “Generic Prompt” in the General purpose LLMs.

**Table 4s:** GAI generated Lay abstracts and summaries (Test 1-3), using Task specific LLM-powered tool (Pub2Post).

**Tabel 5s:** Comparison of Readability Scores, Indexes and Text Statistics between GAI-Generated Lay Abstracts and Summaries across Three Consecutive Tests, using a “Specific Prompt” in the Generic LLMs.

**Tabel 6s:** Comparison of Readability Scores, Indexes and Text Statistics between GAI-Generated Lay Abstracts and Summaries across Three Consecutive Tests, using a “Generic Prompt” in the Generic LLMs.

**Table 7s:** Comparison of Readability Scores, Indexes and Text Statistics between GAI-Generated Lay Abstracts and Summaries across Three Consecutive Tests, using a task-specific LLM powered tool (Pub2post).

**Table 8s.** Comparison of Readability Scores, Indexes and Text Statistics between GAI-Generated Lay Abstracts and Summaries Generated by Task Specific vs Generic LLMs (using a “Specific Prompt” in the Generic LLMs).

**Table 9s.** Comparison of Readability Scores, Indexes and Text Statistics between GAI-Generated Lay Abstracts and Summaries Generated by Task Specific vs Generic LLMs (using a “Generic Prompt” in the Generic LLMs).

**Table 10s.** Comparison of Task specific vs General-purpose LLM Best Tests using the specific prompt.

**Table 11s.** Comparison of Task specific vs General-purpose LLM Best Tests using the generic prompt.

***Table 1s:*** *Prompts employed to generate layperson-friendly abstracts and summaries using generic LLMs.*

| **Specific Prompt (1)** |
| --- |
| I kindly request your assistance in crafting a Simple Summary as part of a scientific study. The Simple Summary must adhere to the following guidelines:  It should be written in one paragraph, in layman’s terms, to explain why the research is being suggested, what the authors aim to achieve, and how the findings from this research may impact the research community. Please use as few abbreviations as possible, and do not cite references in the Simple Summary. The Simple Summary should not exceed 150 words.  The Simple Summary should be crafted with a focus on maximizing readability, aiming for the highest possible Flesch Kincaid Reading Ease score.  To provide you with the necessary context for creating this Simple Summary, I will supply you with the study title, a scientifically accurate abstract (not in Layman’s terms), and the relevant keywords. Study title: “…” Scientifically accurate abstract: “…” Keywords: “…”  Please note: Summarize this unstructured abstract (simple summary) with at most 150 words in lay language at a 6th grade reading level, highlighting the study purpose, methods, key findings, and practical importance of these findings for the general public. Additionally, be aware that the Simple Summary should not exceed 150 words, but it should make the most of this limit. |
| **Generic Prompt** |
| Summarize this text in one short paragraph using simple, easy-to-understand language |

1. Rinderknecht EA-O, Schmelzer A, Kravchuk AA-O, Goßler CA-O, Breyer J, Gilfrich CA-O, et al. Leveraging Large Language Models for High-Quality Lay Summaries: Efficacy of ChatGPT-4 with Custom Prompts in a Consecutive Series of Prostate Cancer Manuscripts. LID - 10.3390/curroncol32020102 [doi] LID - 102. (1718-7729 (Electronic)).

**Table 2s: GAI generated Lay abstracts and summaries (Test 1-3), using a “Specific Prompt” in the General purpose LLMs.**

| **Journal** | **Abstract** | **DOI** | **Arm** | **Original Abstract** | **Test 1** | **Test 2** | **Test 3** |
| --- | --- | --- | --- | --- | --- | --- | --- |
| JCO | 1 | 10.1200/JCO-24-02008 | Open AI | PURPOSETo our knowledge, the ACHILLES/TORG1834 trial is the first randomized study comparing afatinib and chemotherapy in patients with non-small cell lung cancer (NSCLC) harboring sensitizing uncommon epidermal growth factor receptor (EGFR) mutations.METHODSThis randomized, open-label study was performed at 51 Japanese institutions and recruited treatment-na & iuml;ve patients with nonsquamous NSCLC with uncommon EGFR mutations, excluding exon 20 insertions and T790M mutations. Patients were randomly assigned 2:1 to receive afatinib (30 or 40 mg orally, at the treating physician's discretion) or a combination of platinum (cisplatin or carboplatin) and pemetrexed, followed by pemetrexed maintenance. The primary end point was progression-free survival (PFS). Secondary end points included objective response rate (ORR), overall survival, and safety. A prespecified interim analysis was planned to provide clinically meaningful information promptly, along with a crossover recommendation if necessary.RESULTSA total of 109 patients were enrolled between March 2019 and February 2023. In the interim analysis, the Data and Safety Monitoring Committee recommended early study termination. The median PFS was significantly longer in patients receiving afatinib than in those undergoing chemotherapy (10.6 v 5.7 months; hazard ratio, 0.421 [95% CI, 0.251 to 0.706]; P = .0010). ORRs to afatinib were similar across the overall population and among participants with major uncommon (G719X, L861Q, and S768I), compound, and other mutations (61.7%, 55.8%, 72.7%, and 60.0%, respectively). The most common grade 3 or higher adverse events were diarrhea, paronychia, and rash for afatinib, and appetite loss and nausea for chemotherapy.CONCLUSIONAfatinib should be considered the standard initial therapy for patients with NSCLC with sensitizing uncommon EGFR mutations. | This study looked at the best first treatment for people with a type of lung cancer called non-small cell lung cancer that has certain rare changes in the EGFR gene. These changes can affect how cancer grows and how it responds to medicine. Researchers compared two treatments: afatinib, a daily pill that targets EGFR, and standard chemotherapy. Over 100 patients from Japan took part in the trial. Results showed that people who took afatinib lived longer without their cancer getting worse (about 11 months) compared to those who had chemotherapy (about 6 months). Afatinib worked well across different types of these rare gene changes, with common side effects including diarrhea, skin changes, and nail problems. These findings suggest that afatinib should be the preferred first treatment for this group of patients, which may help doctors provide more effective and personalized care. | This study looked at the best first treatment for people with a certain type of lung cancer called non-small cell lung cancer, who have rare changes in a gene called EGFR. Researchers compared a targeted pill medicine called afatinib with standard chemotherapy. Over 100 patients in Japan joined the study. People who took afatinib lived longer before their cancer grew again compared to those on chemotherapy. The chances of the cancer shrinking were also high with afatinib for all types of rare EGFR changes studied. Side effects were different for each treatment: afatinib most often caused diarrhea, skin changes, and nail problems, while chemotherapy caused appetite loss and nausea. These results show that afatinib should be the first treatment choice for people with this kind of lung cancer, giving doctors and patients a clearer path forward. | This study tested two treatments for people with a type of lung cancer called non-small cell lung cancer that has rare changes in the EGFR gene. One treatment was afatinib, a pill that targets these changes, and the other was standard chemotherapy. Over 100 patients in Japan who had not received prior treatment were randomly assigned to one of the two options. The results showed that people taking afatinib lived longer before their cancer got worse compared to those receiving chemotherapy. Afatinib also worked well across different types of uncommon EGFR changes. Side effects were different for each treatment but generally matched what is already known. These results suggest that afatinib should be the first choice for treating this group of patients, which could lead to better outcomes and more personalized care for people with these rare forms of lung cancer. |
| JCO | 1 | 10.1200/JCO-24-02009 | Claude | PURPOSETo our knowledge, the ACHILLES/TORG1834 trial is the first randomized study comparing afatinib and chemotherapy in patients with non-small cell lung cancer (NSCLC) harboring sensitizing uncommon epidermal growth factor receptor (EGFR) mutations.METHODSThis randomized, open-label study was performed at 51 Japanese institutions and recruited treatment-na & iuml;ve patients with nonsquamous NSCLC with uncommon EGFR mutations, excluding exon 20 insertions and T790M mutations. Patients were randomly assigned 2:1 to receive afatinib (30 or 40 mg orally, at the treating physician's discretion) or a combination of platinum (cisplatin or carboplatin) and pemetrexed, followed by pemetrexed maintenance. The primary end point was progression-free survival (PFS). Secondary end points included objective response rate (ORR), overall survival, and safety. A prespecified interim analysis was planned to provide clinically meaningful information promptly, along with a crossover recommendation if necessary.RESULTSA total of 109 patients were enrolled between March 2019 and February 2023. In the interim analysis, the Data and Safety Monitoring Committee recommended early study termination. The median PFS was significantly longer in patients receiving afatinib than in those undergoing chemotherapy (10.6 v 5.7 months; hazard ratio, 0.421 [95% CI, 0.251 to 0.706]; P = .0010). ORRs to afatinib were similar across the overall population and among participants with major uncommon (G719X, L861Q, and S768I), compound, and other mutations (61.7%, 55.8%, 72.7%, and 60.0%, respectively). The most common grade 3 or higher adverse events were diarrhea, paronychia, and rash for afatinib, and appetite loss and nausea for chemotherapy.CONCLUSIONAfatinib should be considered the standard initial therapy for patients with NSCLC with sensitizing uncommon EGFR mutations. | Lung cancer is one of the most serious types of cancer, and some patients have specific changes in their genes that affect how well treatments work. This study looked at patients with a type of lung cancer called non-small cell lung cancer who had rare gene changes in something called the growth factor receptor. Doctors wanted to find out which treatment works better: a pill called afatinib or the usual chemotherapy given through a vein. They studied 109 patients in Japan and split them into two groups to compare the treatments. The results showed that patients who took the afatinib pill lived much longer without their cancer getting worse compared to those who got chemotherapy - about 10.6 months versus 5.7 months. Both treatments had side effects, but they were manageable. This research is important because it shows doctors that the afatinib pill should be the first choice of treatment for patients with this specific type of lung cancer, giving them better outcomes and potentially improving their quality of life. | Lung cancer is one of the most common cancers worldwide. Some lung cancers have special changes in a gene called EGFR that help doctors choose the best treatment. Most research has focused on the two most common EGFR changes, but there are other rare EGFR changes that affect about 10-15% of patients. Doctors were not sure which treatment worked best for these patients with rare EGFR changes. This study compared two treatments: a targeted pill called afatinib versus standard chemotherapy drugs given through an IV. The researchers studied 109 patients from 51 hospitals in Japan. They found that patients taking afatinib lived longer without their cancer getting worse compared to those getting chemotherapy (10.6 months versus 5.7 months). About 62% of patients responded well to afatinib. These results show that afatinib should be the first treatment choice for lung cancer patients with these rare EGFR gene changes, giving them a better option than chemotherapy. | Lung cancer is one of the most common and deadly cancers worldwide. Some lung cancer patients have changes in a gene called EGFR that can help doctors choose the best treatment. Most research has focused on common EGFR changes, but some patients have rare EGFR changes that are less understood. This study compared two treatments for patients with these rare EGFR changes: a targeted drug called afatinib versus standard chemotherapy. Researchers followed 109 patients in Japan and found that afatinib worked much better than chemotherapy. Patients taking afatinib lived almost twice as long without their cancer getting worse (10.6 months versus 5.7 months). About 62% of patients responded well to afatinib treatment. The most common side effects from afatinib were diarrhea, nail problems, and skin rash, while chemotherapy caused more appetite loss and nausea. This research shows that afatinib should be the first treatment choice for lung cancer patients with these rare EGFR changes, giving them a better chance of controlling their disease. |
| JCO | 1 | 10.1200/JCO-24-02010 | Gemini | PURPOSETo our knowledge, the ACHILLES/TORG1834 trial is the first randomized study comparing afatinib and chemotherapy in patients with non-small cell lung cancer (NSCLC) harboring sensitizing uncommon epidermal growth factor receptor (EGFR) mutations.METHODSThis randomized, open-label study was performed at 51 Japanese institutions and recruited treatment-na & iuml;ve patients with nonsquamous NSCLC with uncommon EGFR mutations, excluding exon 20 insertions and T790M mutations. Patients were randomly assigned 2:1 to receive afatinib (30 or 40 mg orally, at the treating physician's discretion) or a combination of platinum (cisplatin or carboplatin) and pemetrexed, followed by pemetrexed maintenance. The primary end point was progression-free survival (PFS). Secondary end points included objective response rate (ORR), overall survival, and safety. A prespecified interim analysis was planned to provide clinically meaningful information promptly, along with a crossover recommendation if necessary.RESULTSA total of 109 patients were enrolled between March 2019 and February 2023. In the interim analysis, the Data and Safety Monitoring Committee recommended early study termination. The median PFS was significantly longer in patients receiving afatinib than in those undergoing chemotherapy (10.6 v 5.7 months; hazard ratio, 0.421 [95% CI, 0.251 to 0.706]; P = .0010). ORRs to afatinib were similar across the overall population and among participants with major uncommon (G719X, L861Q, and S768I), compound, and other mutations (61.7%, 55.8%, 72.7%, and 60.0%, respectively). The most common grade 3 or higher adverse events were diarrhea, paronychia, and rash for afatinib, and appetite loss and nausea for chemotherapy.CONCLUSIONAfatinib should be considered the standard initial therapy for patients with NSCLC with sensitizing uncommon EGFR mutations. | This study was conducted to find the best initial treatment for a specific type of lung cancer caused by uncommon genetic changes in cancer cells. Researchers compared a targeted medicine, afatinib, to traditional chemotherapy. The results clearly showed that patients who received afatinib lived significantly longer without their cancer getting worse. Because the benefits of afatinib were so clear, the study was stopped early to offer this treatment to all participants. This important finding establishes afatinib as the new standard first treatment for these patients, giving them a much better chance for a positive outcome. | This study compared two treatments, afatinib and chemotherapy, for patients with a specific type of lung cancer. These patients have a unique genetic change in their cancer cells, known as uncommon epidermal growth factor receptor mutations. The research aimed to determine which treatment was more effective at stopping the cancer from growing. The study found that patients who received afatinib lived longer without their cancer getting worse compared to those who received chemotherapy. This suggests that afatinib is a better initial treatment choice for these patients. These findings are important for doctors and researchers because they establish afatinib as the new standard first-line treatment for this group of lung cancer patients. | This study addresses a gap in lung cancer treatment by exploring the effectiveness of afatinib, a targeted therapy, against standard chemotherapy for patients with a specific type of non-small cell lung cancer. These patients have rare genetic changes, or mutations, in a gene called the epidermal growth factor receptor (EGFR). The research aimed to determine if afatinib could delay cancer progression more effectively than chemotherapy. The findings revealed that patients receiving afatinib had significantly longer periods before their cancer worsened, proving it to be a more effective initial treatment. This discovery is important because it establishes a new standard of care, offering a better first-line treatment option for patients with these uncommon EGFR mutations and improving their outcomes. |
| JCO | 1 | 10.1200/JCO-24-02011 | DeepSeek | PURPOSETo our knowledge, the ACHILLES/TORG1834 trial is the first randomized study comparing afatinib and chemotherapy in patients with non-small cell lung cancer (NSCLC) harboring sensitizing uncommon epidermal growth factor receptor (EGFR) mutations.METHODSThis randomized, open-label study was performed at 51 Japanese institutions and recruited treatment-na & iuml;ve patients with nonsquamous NSCLC with uncommon EGFR mutations, excluding exon 20 insertions and T790M mutations. Patients were randomly assigned 2:1 to receive afatinib (30 or 40 mg orally, at the treating physician's discretion) or a combination of platinum (cisplatin or carboplatin) and pemetrexed, followed by pemetrexed maintenance. The primary end point was progression-free survival (PFS). Secondary end points included objective response rate (ORR), overall survival, and safety. A prespecified interim analysis was planned to provide clinically meaningful information promptly, along with a crossover recommendation if necessary.RESULTSA total of 109 patients were enrolled between March 2019 and February 2023. In the interim analysis, the Data and Safety Monitoring Committee recommended early study termination. The median PFS was significantly longer in patients receiving afatinib than in those undergoing chemotherapy (10.6 v 5.7 months; hazard ratio, 0.421 [95% CI, 0.251 to 0.706]; P = .0010). ORRs to afatinib were similar across the overall population and among participants with major uncommon (G719X, L861Q, and S768I), compound, and other mutations (61.7%, 55.8%, 72.7%, and 60.0%, respectively). The most common grade 3 or higher adverse events were diarrhea, paronychia, and rash for afatinib, and appetite loss and nausea for chemotherapy.CONCLUSIONAfatinib should be considered the standard initial therapy for patients with NSCLC with sensitizing uncommon EGFR mutations. | This study tested whether a drug called afatinib works better than standard chemotherapy for patients with a rare type of lung cancer (non-small cell lung cancer, or NSCLC) caused by unusual EGFR gene mutations. Researchers compared the two treatments in 109 patients who had never been treated before. They found that afatinib slowed cancer growth longer (10.6 months vs. 5.7 months) and had fewer severe side effects than chemotherapy. The drug worked well across different uncommon mutations. Based on these results, afatinib could become the preferred first treatment for these patients, offering them more time without their cancer worsening. This is important because it gives doctors clearer guidance on how to help people with this specific type of lung cancer live better for longer. | This study tested whether the drug afatinib works better than standard chemotherapy for patients with a rare type of lung cancer caused by uncommon EGFR gene changes. Researchers split 109 patients into two groups: two-thirds took afatinib (a daily pill), while the rest received chemotherapy (IV drugs). Early results showed afatinib nearly doubled the time before the cancer worsened (10.6 vs. 5.7 months) and had fewer severe side effects like nausea. The drug worked well across different uncommon EGFR mutations. Because afatinib was clearly more effective, the trial was stopped early to give all patients access to it. This finding is important—it suggests afatinib should be the first-choice treatment for people with these rare lung cancer mutations, offering hope for better outcomes. | This study tested whether the drug afatinib works better than standard chemotherapy for patients with a rare type of lung cancer (non-small cell lung cancer or NSCLC) caused by unusual EGFR gene mutations. The trial included 109 patients in Japan who had not received prior treatment. They were randomly given either afatinib (a daily pill) or chemotherapy (IV drugs). Early results showed that afatinib nearly doubled the time before the cancer worsened (10.6 months vs. 5.7 months) and had fewer severe side effects like nausea. While some patients on afatinib had diarrhea or skin rashes, these were manageable. The findings suggest that afatinib should be the first-choice treatment for this specific group of lung cancer patients, offering them more time without disease progression compared to chemotherapy. This could change how doctors treat these cases worldwide. |
| JCO | 1 | 10.1200/JCO-24-02012 | Grok | PURPOSETo our knowledge, the ACHILLES/TORG1834 trial is the first randomized study comparing afatinib and chemotherapy in patients with non-small cell lung cancer (NSCLC) harboring sensitizing uncommon epidermal growth factor receptor (EGFR) mutations.METHODSThis randomized, open-label study was performed at 51 Japanese institutions and recruited treatment-na & iuml;ve patients with nonsquamous NSCLC with uncommon EGFR mutations, excluding exon 20 insertions and T790M mutations. Patients were randomly assigned 2:1 to receive afatinib (30 or 40 mg orally, at the treating physician's discretion) or a combination of platinum (cisplatin or carboplatin) and pemetrexed, followed by pemetrexed maintenance. The primary end point was progression-free survival (PFS). Secondary end points included objective response rate (ORR), overall survival, and safety. A prespecified interim analysis was planned to provide clinically meaningful information promptly, along with a crossover recommendation if necessary.RESULTSA total of 109 patients were enrolled between March 2019 and February 2023. In the interim analysis, the Data and Safety Monitoring Committee recommended early study termination. The median PFS was significantly longer in patients receiving afatinib than in those undergoing chemotherapy (10.6 v 5.7 months; hazard ratio, 0.421 [95% CI, 0.251 to 0.706]; P = .0010). ORRs to afatinib were similar across the overall population and among participants with major uncommon (G719X, L861Q, and S768I), compound, and other mutations (61.7%, 55.8%, 72.7%, and 60.0%, respectively). The most common grade 3 or higher adverse events were diarrhea, paronychia, and rash for afatinib, and appetite loss and nausea for chemotherapy.CONCLUSIONAfatinib should be considered the standard initial therapy for patients with NSCLC with sensitizing uncommon EGFR mutations. | This study, called ACHILLES, looked at a new way to treat people with a type of lung cancer that has rare changes in a gene called EGFR. The researchers wanted to see if a medicine called afatinib works better than standard chemotherapy to help patients live longer without their cancer growing. They tested 109 patients, giving some afatinib and others chemotherapy. The results showed that afatinib helped patients stay cancer-free for about 10.6 months, compared to 5.7 months for chemotherapy. This means afatinib could be a better first treatment for these patients. The findings are important because they give doctors a clearer choice for treating this kind of lung cancer, which could help more patients live longer and feel better. This research will guide future studies and improve care for people with this rare cancer. | This study, called ACHILLES, explores better treatment options for people with a specific type of lung cancer called non-small cell lung cancer, which has rare changes in a gene called EGFR. The researchers wanted to see if a drug called afatinib works better than standard chemotherapy to stop the cancer from growing. They tested 109 patients, giving some afatinib pills and others chemotherapy drugs. The results showed that afatinib helped patients live longer without their cancer worsening compared to chemotherapy. This means afatinib could be a better first treatment for people with this kind of lung cancer. The findings are important because they help doctors choose the best medicine to improve patients’ lives and guide future research to find even better treatments. | This study, called ACHILLES, looks at better ways to treat a type of lung cancer called non-small cell lung cancer in people with rare changes in a gene called EGFR. Doctors wanted to see if a pill called afatinib works better than standard chemotherapy, which uses strong drugs to kill cancer cells. They tested 109 patients, giving some afatinib and others chemotherapy. The study found that people taking afatinib lived longer without their cancer growing compared to those on chemotherapy (about 10.6 months versus 5.7 months). Afatinib also helped shrink tumors in many patients. This discovery is exciting because it suggests afatinib could be a better first treatment for these patients, helping them live longer with fewer side effects. Researchers now have stronger evidence to guide doctors in choosing the best treatment for this lung cancer. |
| JCO | 2 | 10.1200/JCO-24-01270 | Open AI | PURPOSE Mobocertinib is an oral epidermal growth factor receptor (EGFR) tyrosine kinase inhibitor that targets EGFR exon 20 insertion (ex20ins) mutations in non-small cell lung cancer (NSCLC). This open-label, phase III trial (EXCLAIM-2, ClinicalTrials.gov identifier: NCT04129502) compared mobocertinib versus platinum-based chemotherapy as first-line treatment of EGFR ex20ins+ advanced/metastatic NSCLC. METHODS Patients with treatment-naive EGFR ex20ins+ locally advanced/metastatic NSCLC were randomly assigned 1:1 to mobocertinib 160 mg once daily or pemetrexed plus cisplatin or carboplatin every 3 weeks for four cycles followed by maintenance pemetrexed. The primary end point was progression-free survival (PFS) by blinded independent central review (BICR), with planned interim analysis (IA) after approximately 70% of 227 expected PFS events. RESULTS A total of 354 patients were randomly assigned (mobocertinib: n = 179; chemotherapy: n = 175). Baseline characteristics were balanced between arms. At IA (cutoff: April 4, 2023), the median PFS per BICR was 9.6 months in each treatment arm (hazard ratio [HR], 1.04 [95% CI, 0.77 to 1.39]; P = .803). The primary end point crossed the prespecified futility boundary (HR > 1). The confirmed objective response rate (95% CI) per BICR was 32% (26 to 40) with mobocertinib versus 30% (24 to 38) with chemotherapy; the median duration of response was 12.0 versus 8.4 months. Quality-of-life assessments indicated clinically meaningful delays in time to deterioration of lung cancer symptoms, cognitive function, and constipation with mobocertinib versus chemotherapy. Grade >= 3 adverse events in >5% of patients (mobocertinib, chemotherapy) were diarrhea (20%, 1%), anemia (6%, 10%), increased lipase (6%, 0%), and decreased neutrophil count (1%, 7%). CONCLUSION The EXCLAIM-2 trial did not meet its primary end point. The efficacy of mobocertinib was not superior to platinum-based chemotherapy for first-line treatment of patients with EGFR ex20ins+ advanced/metastatic NSCLC. | This study looked at whether mobocertinib, a daily pill, works better than standard chemotherapy as the first treatment for people with advanced lung cancer caused by a specific change in the EGFR gene called exon 20 insertion. In this phase III trial, 354 patients were randomly given mobocertinib or chemotherapy. Researchers measured how long patients lived without their cancer getting worse and how well their tumors responded to treatment. Both treatments kept the cancer from worsening for about the same time—around 9.6 months. Tumor shrinkage rates were also similar. However, mobocertinib helped delay the worsening of certain symptoms and some side effects were different between the groups. While mobocertinib was not more effective than chemotherapy overall, the results give important information to guide future research and treatment choices for this rare type of lung cancer. | This study compared two treatments for people with a type of advanced lung cancer caused by a rare change in the EGFR gene called exon 20 insertion. One treatment, mobocertinib, is a daily pill designed to block this change. The other is standard chemotherapy with platinum drugs and pemetrexed. In the trial, 354 patients who had not received prior treatment were randomly assigned to one of the two options. Both treatments worked about the same in keeping the cancer from getting worse, with a median time of about 9.6 months. Response rates were also similar, though mobocertinib’s effects lasted longer on average. People taking mobocertinib reported better quality of life in some areas, such as fewer lung cancer symptoms and less constipation. Side effects were different for each treatment. Although mobocertinib did not prove better than chemotherapy, these results give important information to guide future treatment choices. | This study looked at a new pill called mobocertinib compared with standard chemotherapy for people with advanced lung cancer caused by a specific gene change called EGFR exon 20 insertion. The goal was to see if mobocertinib could help patients live longer without the cancer getting worse. In the trial, 354 patients who had not received treatment before were randomly given either mobocertinib or standard chemotherapy. Results showed that both treatments worked similarly, with patients living about the same amount of time before their cancer progressed. Mobocertinib helped some symptoms and side effects less than chemotherapy, but overall it was not better at controlling the cancer. These findings help doctors understand that while mobocertinib is an option, standard chemotherapy remains an effective first treatment for this type of lung cancer. |
| JCO | 2 | 10.1200/JCO-24-01271 | Claude | PURPOSE Mobocertinib is an oral epidermal growth factor receptor (EGFR) tyrosine kinase inhibitor that targets EGFR exon 20 insertion (ex20ins) mutations in non-small cell lung cancer (NSCLC). This open-label, phase III trial (EXCLAIM-2, ClinicalTrials.gov identifier: NCT04129502) compared mobocertinib versus platinum-based chemotherapy as first-line treatment of EGFR ex20ins+ advanced/metastatic NSCLC. METHODS Patients with treatment-naive EGFR ex20ins+ locally advanced/metastatic NSCLC were randomly assigned 1:1 to mobocertinib 160 mg once daily or pemetrexed plus cisplatin or carboplatin every 3 weeks for four cycles followed by maintenance pemetrexed. The primary end point was progression-free survival (PFS) by blinded independent central review (BICR), with planned interim analysis (IA) after approximately 70% of 227 expected PFS events. RESULTS A total of 354 patients were randomly assigned (mobocertinib: n = 179; chemotherapy: n = 175). Baseline characteristics were balanced between arms. At IA (cutoff: April 4, 2023), the median PFS per BICR was 9.6 months in each treatment arm (hazard ratio [HR], 1.04 [95% CI, 0.77 to 1.39]; P = .803). The primary end point crossed the prespecified futility boundary (HR > 1). The confirmed objective response rate (95% CI) per BICR was 32% (26 to 40) with mobocertinib versus 30% (24 to 38) with chemotherapy; the median duration of response was 12.0 versus 8.4 months. Quality-of-life assessments indicated clinically meaningful delays in time to deterioration of lung cancer symptoms, cognitive function, and constipation with mobocertinib versus chemotherapy. Grade >= 3 adverse events in >5% of patients (mobocertinib, chemotherapy) were diarrhea (20%, 1%), anemia (6%, 10%), increased lipase (6%, 0%), and decreased neutrophil count (1%, 7%). CONCLUSION The EXCLAIM-2 trial did not meet its primary end point. The efficacy of mobocertinib was not superior to platinum-based chemotherapy for first-line treatment of patients with EGFR ex20ins+ advanced/metastatic NSCLC. | Lung cancer is one of the most common and deadly cancers worldwide. Some lung cancer patients have a specific genetic change that makes their cancer grow. Scientists wanted to test if a new pill called mobocertinib works better than standard chemotherapy drugs for treating these patients. They studied 354 people with advanced lung cancer who had never received treatment before. Half the patients took the new pill daily, while the other half received chemotherapy through an IV every three weeks. The researchers watched how long it took for each patient's cancer to get worse. Both treatments worked equally well - patients lived about 9.6 months before their cancer grew. The new pill did not work better than chemotherapy. However, patients taking the pill had fewer problems with low blood counts and reported better quality of life in some areas, though they had more diarrhea. This study helps doctors understand that the new pill is not superior to current treatments for this type of lung cancer. | Lung cancer is one of the most common and deadly cancers worldwide. Some lung cancers have changes in a gene called EGFR that make them grow and spread. Scientists created a new pill called mobocertinib to fight these specific lung cancers. This study tested whether this new pill works better than standard chemotherapy drugs given through a vein. Researchers studied 354 people with advanced lung cancer that had spread to other parts of their body. Half the patients took the new pill daily, while the other half received chemotherapy every three weeks. The main goal was to see which treatment kept the cancer from getting worse for a longer time. Results showed both treatments worked equally well, with patients staying cancer-free for about 9.6 months on average. However, people taking the pill had fewer serious side effects and better quality of life. While the new pill did not work better than chemotherapy, it offers patients an easier treatment option with similar results and fewer harsh side effects. | Lung cancer is a serious disease that affects many people worldwide. Some lung cancers have changes in a gene called EGFR that make them harder to treat with regular medicines. Scientists created a new pill called mobocertinib to target these specific gene changes and help patients live longer with better quality of life. In this study, researchers wanted to see if this new pill worked better than the standard cancer drugs that doctors usually give patients. They tested 354 people with this type of lung cancer, giving half the new pill and half the usual chemotherapy treatment. After studying the results, they found that both treatments helped patients for about the same amount of time - around 9.6 months before the cancer got worse. While the new pill did not work better than chemotherapy, it did help patients feel better in some ways, causing less harm to their thinking and fewer bowel problems. This research helps doctors understand treatment options for patients with this specific type of lung cancer. |
| JCO | 2 | 10.1200/JCO-24-01272 | Gemini | PURPOSE Mobocertinib is an oral epidermal growth factor receptor (EGFR) tyrosine kinase inhibitor that targets EGFR exon 20 insertion (ex20ins) mutations in non-small cell lung cancer (NSCLC). This open-label, phase III trial (EXCLAIM-2, ClinicalTrials.gov identifier: NCT04129502) compared mobocertinib versus platinum-based chemotherapy as first-line treatment of EGFR ex20ins+ advanced/metastatic NSCLC. METHODS Patients with treatment-naive EGFR ex20ins+ locally advanced/metastatic NSCLC were randomly assigned 1:1 to mobocertinib 160 mg once daily or pemetrexed plus cisplatin or carboplatin every 3 weeks for four cycles followed by maintenance pemetrexed. The primary end point was progression-free survival (PFS) by blinded independent central review (BICR), with planned interim analysis (IA) after approximately 70% of 227 expected PFS events. RESULTS A total of 354 patients were randomly assigned (mobocertinib: n = 179; chemotherapy: n = 175). Baseline characteristics were balanced between arms. At IA (cutoff: April 4, 2023), the median PFS per BICR was 9.6 months in each treatment arm (hazard ratio [HR], 1.04 [95% CI, 0.77 to 1.39]; P = .803). The primary end point crossed the prespecified futility boundary (HR > 1). The confirmed objective response rate (95% CI) per BICR was 32% (26 to 40) with mobocertinib versus 30% (24 to 38) with chemotherapy; the median duration of response was 12.0 versus 8.4 months. Quality-of-life assessments indicated clinically meaningful delays in time to deterioration of lung cancer symptoms, cognitive function, and constipation with mobocertinib versus chemotherapy. Grade >= 3 adverse events in >5% of patients (mobocertinib, chemotherapy) were diarrhea (20%, 1%), anemia (6%, 10%), increased lipase (6%, 0%), and decreased neutrophil count (1%, 7%). CONCLUSION The EXCLAIM-2 trial did not meet its primary end point. The efficacy of mobocertinib was not superior to platinum-based chemotherapy for first-line treatment of patients with EGFR ex20ins+ advanced/metastatic NSCLC. | This study explores whether a new oral medicine, mobocertinib, is a better first treatment for a specific type of advanced lung cancer compared to standard chemotherapy. Researchers gave either the new pill or traditional chemotherapy to patients with this particular kind of lung cancer. The main goal was to see if the new medicine could slow the cancer's growth for a longer time. The study found that both treatments worked equally well at controlling the cancer's progression. While mobocertinib did not perform better, it did offer some benefits, such as delaying a decline in a patient’s quality of life and causing different side effects. These findings are important for doctors and researchers to help guide future treatment decisions for patients with this form of lung cancer. | This study looked at a new drug called mobocertinib to see if it was a better first treatment for a specific type of lung cancer compared to standard chemotherapy. This cancer has a unique genetic change, which the new drug was designed to target. The researchers hoped to find that mobocertinib could slow down the cancer's growth for a longer time than chemotherapy. While the study found that both treatments worked about the same in terms of stopping the cancer from getting worse, mobocertinib did show some benefits, such as helping patients feel better for longer and delaying some of the cancer's symptoms. This research is important because it adds to our understanding of how to treat this specific type of lung cancer and helps doctors make better decisions for their patients. | This study tested a new pill called mobocertinib to see if it was a better initial treatment for a specific type of lung cancer compared to standard chemotherapy. This particular lung cancer has a unique genetic change that makes it difficult to treat. In this trial, researchers gave either the new pill or traditional chemotherapy to patients with this advanced cancer. The main finding was that the new medicine was not more effective at stopping the cancer from growing than chemotherapy. Both treatments worked for a similar amount of time. However, the study also found that the mobocertinib pill improved patients' quality of life by delaying the worsening of symptoms like breathing problems and cognitive function. This is an important result for doctors and researchers, as it helps them understand the best ways to treat this cancer and informs future studies on finding better treatment options. |
| JCO | 2 | 10.1200/JCO-24-01273 | DeepSeek | PURPOSE Mobocertinib is an oral epidermal growth factor receptor (EGFR) tyrosine kinase inhibitor that targets EGFR exon 20 insertion (ex20ins) mutations in non-small cell lung cancer (NSCLC). This open-label, phase III trial (EXCLAIM-2, ClinicalTrials.gov identifier: NCT04129502) compared mobocertinib versus platinum-based chemotherapy as first-line treatment of EGFR ex20ins+ advanced/metastatic NSCLC. METHODS Patients with treatment-naive EGFR ex20ins+ locally advanced/metastatic NSCLC were randomly assigned 1:1 to mobocertinib 160 mg once daily or pemetrexed plus cisplatin or carboplatin every 3 weeks for four cycles followed by maintenance pemetrexed. The primary end point was progression-free survival (PFS) by blinded independent central review (BICR), with planned interim analysis (IA) after approximately 70% of 227 expected PFS events. RESULTS A total of 354 patients were randomly assigned (mobocertinib: n = 179; chemotherapy: n = 175). Baseline characteristics were balanced between arms. At IA (cutoff: April 4, 2023), the median PFS per BICR was 9.6 months in each treatment arm (hazard ratio [HR], 1.04 [95% CI, 0.77 to 1.39]; P = .803). The primary end point crossed the prespecified futility boundary (HR > 1). The confirmed objective response rate (95% CI) per BICR was 32% (26 to 40) with mobocertinib versus 30% (24 to 38) with chemotherapy; the median duration of response was 12.0 versus 8.4 months. Quality-of-life assessments indicated clinically meaningful delays in time to deterioration of lung cancer symptoms, cognitive function, and constipation with mobocertinib versus chemotherapy. Grade >= 3 adverse events in >5% of patients (mobocertinib, chemotherapy) were diarrhea (20%, 1%), anemia (6%, 10%), increased lipase (6%, 0%), and decreased neutrophil count (1%, 7%). CONCLUSION The EXCLAIM-2 trial did not meet its primary end point. The efficacy of mobocertinib was not superior to platinum-based chemotherapy for first-line treatment of patients with EGFR ex20ins+ advanced/metastatic NSCLC. | This study tested whether a new pill called mobocertinib works better than standard chemotherapy as the first treatment for advanced lung cancer with a specific gene change (EGFR exon 20 insertion). Researchers compared the two treatments in 354 patients. Both treatments kept the cancer from growing for about 9.6 months on average, showing no major difference in effectiveness. However, mobocertinib helped patients maintain their quality of life longer, with fewer worsening symptoms like coughing and thinking problems. Side effects differed—mobocertinib caused more diarrhea, while chemotherapy led to more anemia. Although the new drug wasn’t more effective than chemotherapy, it may still be a helpful option for some patients due to its ease of use (a daily pill) and better symptom control. | This study tested whether a new pill called mobocertinib works better than standard chemotherapy as the first treatment for people with a rare type of lung cancer (EGFR exon 20-positive NSCLC). Researchers split 354 patients into two groups: one took mobocertinib daily, while the other received chemotherapy. Both treatments kept the cancer from growing for about 9.6 months on average. While mobocertinib didn’t outperform chemotherapy overall, it helped patients maintain their quality of life longer, with fewer worsening symptoms like cough or fatigue. Side effects differed—diarrhea was more common with mobocertinib, while chemotherapy caused more anemia. Though the new drug wasn’t more effective, it offers a convenient oral option with different side effects, which may benefit some patients. These findings help doctors and patients weigh treatment choices for this hard-to-treat cancer. | This study tested whether a new pill called mobocertinib works better than standard chemotherapy as the first treatment for advanced lung cancer with a specific gene change (EGFR exon 20). Researchers gave 354 patients either mobocertinib daily or chemotherapy every 3 weeks. Both treatments kept the cancer from growing for about 9.6 months on average. While mobocertinib didn’t work better than chemotherapy overall, it helped some patients live longer without worsening symptoms like cough or memory problems. Side effects differed—mobocertinib caused more diarrhea, while chemotherapy led to more anemia. These results show that chemotherapy remains a standard option, but mobocertinib may still help some patients with fewer side effects on daily life. |
| JCO | 2 | 10.1200/JCO-24-01274 | Grok | PURPOSE Mobocertinib is an oral epidermal growth factor receptor (EGFR) tyrosine kinase inhibitor that targets EGFR exon 20 insertion (ex20ins) mutations in non-small cell lung cancer (NSCLC). This open-label, phase III trial (EXCLAIM-2, ClinicalTrials.gov identifier: NCT04129502) compared mobocertinib versus platinum-based chemotherapy as first-line treatment of EGFR ex20ins+ advanced/metastatic NSCLC. METHODS Patients with treatment-naive EGFR ex20ins+ locally advanced/metastatic NSCLC were randomly assigned 1:1 to mobocertinib 160 mg once daily or pemetrexed plus cisplatin or carboplatin every 3 weeks for four cycles followed by maintenance pemetrexed. The primary end point was progression-free survival (PFS) by blinded independent central review (BICR), with planned interim analysis (IA) after approximately 70% of 227 expected PFS events. RESULTS A total of 354 patients were randomly assigned (mobocertinib: n = 179; chemotherapy: n = 175). Baseline characteristics were balanced between arms. At IA (cutoff: April 4, 2023), the median PFS per BICR was 9.6 months in each treatment arm (hazard ratio [HR], 1.04 [95% CI, 0.77 to 1.39]; P = .803). The primary end point crossed the prespecified futility boundary (HR > 1). The confirmed objective response rate (95% CI) per BICR was 32% (26 to 40) with mobocertinib versus 30% (24 to 38) with chemotherapy; the median duration of response was 12.0 versus 8.4 months. Quality-of-life assessments indicated clinically meaningful delays in time to deterioration of lung cancer symptoms, cognitive function, and constipation with mobocertinib versus chemotherapy. Grade >= 3 adverse events in >5% of patients (mobocertinib, chemotherapy) were diarrhea (20%, 1%), anemia (6%, 10%), increased lipase (6%, 0%), and decreased neutrophil count (1%, 7%). CONCLUSION The EXCLAIM-2 trial did not meet its primary end point. The efficacy of mobocertinib was not superior to platinum-based chemotherapy for first-line treatment of patients with EGFR ex20ins+ advanced/metastatic NSCLC. | This study explored a new medicine called mobocertinib to help people with a specific type of lung cancer caused by a rare change in a gene called EGFR. The researchers wanted to see if mobocertinib works better than standard chemotherapy for patients who haven’t had treatment before. They tested 354 patients, splitting them into two groups: one got mobocertinib, and the other got chemotherapy. The goal was to check how long patients stayed free of cancer growth. The results showed both treatments worked about the same, with cancer controlled for around 9.6 months. However, mobocertinib helped delay worsening of symptoms like lung problems and improved quality of life. This study helps scientists understand that mobocertinib might be a useful option, encouraging more research to improve lung cancer treatments. | Researchers wanted to find a better treatment for people with a specific type of lung cancer caused by a rare genetic change called EGFR exon 20 insertion. They tested a new pill called mobocertinib against standard chemotherapy to see which worked better for patients who hadn’t been treated before. The study aimed to help patients live longer without their cancer getting worse. They found that both treatments kept the cancer under control for about 9.6 months, so mobocertinib wasn’t better than chemotherapy. However, mobocertinib improved quality of life by delaying symptoms like lung problems and constipation. This study helps doctors understand that mobocertinib may not be the best first treatment but could still improve patients’ daily lives, guiding future research for better lung cancer treatments. | This study, called EXCLAIM-2, was done to see if a new drug, mobocertinib, works better than standard chemotherapy for people with a rare type of lung cancer caused by a specific gene change. Researchers wanted to find out if mobocertinib could help patients live longer without their cancer growing. They tested 354 patients, giving half mobocertinib and half chemotherapy. The results showed that both treatments worked about the same, with cancer growth stopped for around 9.6 months. However, mobocertinib helped delay worsening of lung cancer symptoms and improved quality of life compared to chemotherapy. This research is important because it shows mobocertinib is not better than chemotherapy but may offer other benefits, helping scientists find better ways to treat this type of lung cancer in the future. |
| JCO | 3 | 10.1200/JCO-24-01442 | Open AI | PURPOSETo evaluate the cost utility of a 9-month supervised exercise program for patients with metastatic breast cancer (mBC), compared with control (usual care, supplemented with general activity advice and an activity tracker). Evidence on the cost-effectiveness of exercise for patients with mBC is essential for implementation in clinical practice and is currently lacking.METHODSA cost-utility analysis was performed alongside the multinational PREFERABLE-EFFECT randomized controlled trial, conducted in 8 centers across Europe and Australia. Patients with mBC (N = 357) were randomly assigned to either a 9-month, twice-weekly, supervised exercise group (EG) or control group (CG). Costs of the exercise program were calculated through a bottom-up approach. Other health care resource use, productivity losses, and quality of life were collected using country-adapted, self-reported questionnaires. Analyses were conducted from a societal perspective with a time horizon of 9 months. Costs were collected and reported in 2021 Euros (1 = $1.18 US dollars).RESULTSCompared with the CG, EG resulted in a quality-adjusted life-year (QALY) gain of 0.013 (95% CI, -0.02 to 0.05) over a 9-month period. The mean costs of the exercise program were 1,696 per patient with one-on-one supervision (scenario 1) and 609 with one-on-four supervision (scenario 2). These costs were offset by savings in health care and productivity costs, resulting in mean total cost differences of -163 (scenario 1) and -1,249 (scenario 2) in favor of EG. The probability of supervised exercise being cost-effective was 65% in scenario 1 and 91% in scenario 2 at a willingness-to-pay threshold of 20,000 per QALY.CONCLUSIONExercise for patients with mBC increases quality of life, decreases costs, and is likely to be cost-effective. Group-based supervision is expected to have even higher cost-savings. Our positive findings can inform reimbursement of supervised exercise interventions for patients with mBC. | This study looked at whether a supervised exercise program is worth the cost for people with breast cancer that has spread to other parts of the body. Over nine months, patients either joined exercise sessions twice a week with supervision or received usual care with general activity advice. The researchers measured quality of life, health care costs, and work productivity. They found that exercise slightly improved quality of life and reduced overall costs, especially when patients exercised in small groups instead of one-on-one sessions. These results suggest that supervised exercise not only helps patients feel better but can also save money for health systems and society. Encouraging group-based exercise programs for patients with advanced breast cancer could make treatment more effective, improve daily well-being, and guide health care providers in offering cost-friendly support. | This study looked at whether a supervised exercise program is worth the cost for people with advanced breast cancer that has spread to other parts of the body. Over nine months, 357 patients either joined twice-weekly guided exercise sessions or received usual care with general activity advice and an activity tracker. Researchers measured both health benefits and costs, including medical care and work productivity. They found that exercise slightly improved patients’ quality of life while saving money overall, especially when exercises were done in small groups rather than one-on-one. These findings suggest that supervised exercise is not only safe and helpful but also likely to be a smart use of resources. Supporting such programs could improve the well-being of patients with advanced breast cancer and guide health systems on funding effective exercise interventions. | This study looked at whether a supervised exercise program is worth the cost for people with metastatic breast cancer, a type of advanced breast cancer. Over nine months, 357 patients either joined a program with twice-weekly guided exercise sessions or continued their usual care with general activity advice. The researchers measured costs, health care use, work productivity, and quality of life. They found that patients who exercised under supervision slightly improved their quality of life while overall costs were lower than those who did not participate. Exercise sessions with small groups were especially cost-saving. These results suggest that supervised exercise is not only beneficial for patients’ well-being but also a smart use of resources. Offering such programs could improve care for people with advanced breast cancer and guide health care policies on supporting exercise as part of treatment. |
| JCO | 3 | 10.1200/JCO-24-01443 | Claude | PURPOSETo evaluate the cost utility of a 9-month supervised exercise program for patients with metastatic breast cancer (mBC), compared with control (usual care, supplemented with general activity advice and an activity tracker). Evidence on the cost-effectiveness of exercise for patients with mBC is essential for implementation in clinical practice and is currently lacking.METHODSA cost-utility analysis was performed alongside the multinational PREFERABLE-EFFECT randomized controlled trial, conducted in 8 centers across Europe and Australia. Patients with mBC (N = 357) were randomly assigned to either a 9-month, twice-weekly, supervised exercise group (EG) or control group (CG). Costs of the exercise program were calculated through a bottom-up approach. Other health care resource use, productivity losses, and quality of life were collected using country-adapted, self-reported questionnaires. Analyses were conducted from a societal perspective with a time horizon of 9 months. Costs were collected and reported in 2021 Euros (1 = $1.18 US dollars).RESULTSCompared with the CG, EG resulted in a quality-adjusted life-year (QALY) gain of 0.013 (95% CI, -0.02 to 0.05) over a 9-month period. The mean costs of the exercise program were 1,696 per patient with one-on-one supervision (scenario 1) and 609 with one-on-four supervision (scenario 2). These costs were offset by savings in health care and productivity costs, resulting in mean total cost differences of -163 (scenario 1) and -1,249 (scenario 2) in favor of EG. The probability of supervised exercise being cost-effective was 65% in scenario 1 and 91% in scenario 2 at a willingness-to-pay threshold of 20,000 per QALY.CONCLUSIONExercise for patients with mBC increases quality of life, decreases costs, and is likely to be cost-effective. Group-based supervision is expected to have even higher cost-savings. Our positive findings can inform reimbursement of supervised exercise interventions for patients with mBC. | When breast cancer spreads to other parts of the body, it is called metastatic breast cancer. This study looked at whether supervised exercise programs are worth the money for women with this advanced cancer. Researchers compared 357 patients across Europe and Australia. Half did a 9-month exercise program with trainers twice a week, while the other half got basic activity advice and a fitness tracker. The team tracked how much the exercise program cost and measured patients' quality of life and healthcare expenses. Results showed that exercise patients had better quality of life and actually saved money overall. Even though the exercise program cost money upfront, patients needed less medical care and missed fewer work days, which saved more money than the program cost. Group exercise classes with one trainer for four patients saved even more money than one-on-one training. This research proves that supervised exercise programs for women with advanced breast cancer improve lives while reducing healthcare costs, making them a smart investment for hospitals and insurance companies. | When breast cancer spreads to other parts of the body, it is called metastatic breast cancer. This study looked at whether exercise programs help these patients and save money for healthcare systems. Researchers studied 357 patients across Europe and Australia for nine months. Half did supervised exercise twice a week, while the other half got normal care plus basic activity advice. The exercise group had better quality of life and the program actually saved money overall. Even though the exercise sessions cost money to run, patients needed less other medical care and missed less work, which saved more money than the program cost. When one trainer worked with four patients at once instead of one-on-one, the savings were even bigger. The study shows that supervised exercise programs for metastatic breast cancer patients improve their lives while reducing healthcare costs, making them a smart investment for hospitals and insurance companies. | Women with breast cancer that has spread to other parts of the body (called metastatic breast cancer) often struggle with poor quality of life and high medical costs. This study looked at whether a supervised exercise program could help these patients while also saving money. Researchers followed 357 women for nine months, comparing those who did twice-weekly supervised exercise sessions with those who received usual care plus basic activity advice. The exercise program cost between 609 and 1,696 euros per patient, depending on whether patients exercised in groups or one-on-one with a trainer. However, women in the exercise program had better quality of life and used fewer healthcare services, which saved money overall. The savings were 163 euros per patient for one-on-one training and 1,249 euros for group training. These findings suggest that supervised exercise programs for women with metastatic breast cancer improve wellbeing while reducing healthcare costs, making them a smart investment for healthcare systems. |
| JCO | 3 | 10.1200/JCO-24-01444 | Gemini | PURPOSETo evaluate the cost utility of a 9-month supervised exercise program for patients with metastatic breast cancer (mBC), compared with control (usual care, supplemented with general activity advice and an activity tracker). Evidence on the cost-effectiveness of exercise for patients with mBC is essential for implementation in clinical practice and is currently lacking.METHODSA cost-utility analysis was performed alongside the multinational PREFERABLE-EFFECT randomized controlled trial, conducted in 8 centers across Europe and Australia. Patients with mBC (N = 357) were randomly assigned to either a 9-month, twice-weekly, supervised exercise group (EG) or control group (CG). Costs of the exercise program were calculated through a bottom-up approach. Other health care resource use, productivity losses, and quality of life were collected using country-adapted, self-reported questionnaires. Analyses were conducted from a societal perspective with a time horizon of 9 months. Costs were collected and reported in 2021 Euros (1 = $1.18 US dollars).RESULTSCompared with the CG, EG resulted in a quality-adjusted life-year (QALY) gain of 0.013 (95% CI, -0.02 to 0.05) over a 9-month period. The mean costs of the exercise program were 1,696 per patient with one-on-one supervision (scenario 1) and 609 with one-on-four supervision (scenario 2). These costs were offset by savings in health care and productivity costs, resulting in mean total cost differences of -163 (scenario 1) and -1,249 (scenario 2) in favor of EG. The probability of supervised exercise being cost-effective was 65% in scenario 1 and 91% in scenario 2 at a willingness-to-pay threshold of 20,000 per QALY.CONCLUSIONExercise for patients with mBC increases quality of life, decreases costs, and is likely to be cost-effective. Group-based supervision is expected to have even higher cost-savings. Our positive findings can inform reimbursement of supervised exercise interventions for patients with mBC. | In this study, researchers looked at whether a supervised exercise program is a good investment for patients with breast cancer that has spread. The goal was to see if the benefits of exercise are worth the costs. Patients were split into two groups: one that received a nine-month, twice-weekly exercise program and one that received standard care. The findings showed that the exercise program not only improved the patients' quality of life but also lowered overall health care costs. Because the exercise program paid for itself through these savings, it is likely a very cost-effective treatment. These results are important because they can help doctors and insurance companies decide to cover these helpful exercise programs for breast cancer patients. | This study explored the value of a 9-month supervised exercise program for patients with metastatic breast cancer. Researchers compared this program to standard care, finding that regular exercise not only improved the patients' quality of life but also led to lower overall healthcare costs. The exercise program was found to be very cost-effective, especially when patients exercised in a small group. These positive findings are important because they can help medical providers and insurance companies understand the financial and health benefits of exercise, potentially making it easier for more patients to access these helpful programs in the future. | This study explored the financial benefits and overall well-being of a supervised exercise program for patients with metastatic breast cancer. While it is known that exercise can be helpful for these patients, it was not yet clear if such programs were a good use of healthcare money. The research compared a group of patients who exercised regularly under supervision with a group that received standard care. The findings showed that the exercise program not only improved the patients' quality of life but also led to cost savings, mainly by reducing other healthcare expenses. This suggests that supervised exercise is a smart and affordable choice that can improve patient care and should be considered for wider adoption and insurance coverage. |
| JCO | 3 | 10.1200/JCO-24-01445 | DeepSeek | PURPOSETo evaluate the cost utility of a 9-month supervised exercise program for patients with metastatic breast cancer (mBC), compared with control (usual care, supplemented with general activity advice and an activity tracker). Evidence on the cost-effectiveness of exercise for patients with mBC is essential for implementation in clinical practice and is currently lacking.METHODSA cost-utility analysis was performed alongside the multinational PREFERABLE-EFFECT randomized controlled trial, conducted in 8 centers across Europe and Australia. Patients with mBC (N = 357) were randomly assigned to either a 9-month, twice-weekly, supervised exercise group (EG) or control group (CG). Costs of the exercise program were calculated through a bottom-up approach. Other health care resource use, productivity losses, and quality of life were collected using country-adapted, self-reported questionnaires. Analyses were conducted from a societal perspective with a time horizon of 9 months. Costs were collected and reported in 2021 Euros (1 = $1.18 US dollars).RESULTSCompared with the CG, EG resulted in a quality-adjusted life-year (QALY) gain of 0.013 (95% CI, -0.02 to 0.05) over a 9-month period. The mean costs of the exercise program were 1,696 per patient with one-on-one supervision (scenario 1) and 609 with one-on-four supervision (scenario 2). These costs were offset by savings in health care and productivity costs, resulting in mean total cost differences of -163 (scenario 1) and -1,249 (scenario 2) in favor of EG. The probability of supervised exercise being cost-effective was 65% in scenario 1 and 91% in scenario 2 at a willingness-to-pay threshold of 20,000 per QALY.CONCLUSIONExercise for patients with mBC increases quality of life, decreases costs, and is likely to be cost-effective. Group-based supervision is expected to have even higher cost-savings. Our positive findings can inform reimbursement of supervised exercise interventions for patients with mBC. | This study looked at whether a supervised exercise program is worth the cost for people with advanced (metastatic) breast cancer. Researchers compared a 9-month exercise program (two sessions per week) to usual care in 357 patients across Europe and Australia. They found that exercise improved patients’ quality of life while also saving money—especially when done in small groups instead of one-on-one. The savings came from fewer health care visits and less lost work time. At a reasonable cost, there was a 65-91% chance the program was a good investment. These results suggest that supervised exercise should be more widely available for advanced breast cancer patients, as it benefits both their health and finances. | This study looked at whether a supervised exercise program could improve the lives of people with advanced (metastatic) breast cancer while also being cost-effective. Researchers compared a 9-month exercise program (two sessions per week) with usual care in 357 patients across Europe and Australia. The exercise program improved patients' quality of life and, when done in small groups, actually saved money by reducing other healthcare and work-related costs. One-on-one training also had benefits but was slightly less cost-effective. Overall, supervised exercise—especially in groups—appears to be a worthwhile investment for patients and healthcare systems. These findings support making exercise programs more available to people with advanced breast cancer to help them feel better without adding financial burden. | This study looked at whether a supervised exercise program is worth the cost for patients with advanced (metastatic) breast cancer. Researchers compared a 9-month exercise program (two sessions per week) with usual care in 357 patients across Europe and Australia. They found that exercise improved patients’ quality of life and, surprisingly, saved money—especially when done in small groups instead of one-on-one. The savings came from fewer health care visits and less work time lost. At a reasonable cost per health benefit, group exercise had a 91% chance of being cost-effective. These results suggest that insurance and health systems should cover supervised exercise for metastatic breast cancer, as it helps patients feel better while reducing costs. |
| JCO | 3 | 10.1200/JCO-24-01446 | Grok | PURPOSETo evaluate the cost utility of a 9-month supervised exercise program for patients with metastatic breast cancer (mBC), compared with control (usual care, supplemented with general activity advice and an activity tracker). Evidence on the cost-effectiveness of exercise for patients with mBC is essential for implementation in clinical practice and is currently lacking.METHODSA cost-utility analysis was performed alongside the multinational PREFERABLE-EFFECT randomized controlled trial, conducted in 8 centers across Europe and Australia. Patients with mBC (N = 357) were randomly assigned to either a 9-month, twice-weekly, supervised exercise group (EG) or control group (CG). Costs of the exercise program were calculated through a bottom-up approach. Other health care resource use, productivity losses, and quality of life were collected using country-adapted, self-reported questionnaires. Analyses were conducted from a societal perspective with a time horizon of 9 months. Costs were collected and reported in 2021 Euros (1 = $1.18 US dollars).RESULTSCompared with the CG, EG resulted in a quality-adjusted life-year (QALY) gain of 0.013 (95% CI, -0.02 to 0.05) over a 9-month period. The mean costs of the exercise program were 1,696 per patient with one-on-one supervision (scenario 1) and 609 with one-on-four supervision (scenario 2). These costs were offset by savings in health care and productivity costs, resulting in mean total cost differences of -163 (scenario 1) and -1,249 (scenario 2) in favor of EG. The probability of supervised exercise being cost-effective was 65% in scenario 1 and 91% in scenario 2 at a willingness-to-pay threshold of 20,000 per QALY.CONCLUSIONExercise for patients with mBC increases quality of life, decreases costs, and is likely to be cost-effective. Group-based supervision is expected to have even higher cost-savings. Our positive findings can inform reimbursement of supervised exercise interventions for patients with mBC. | This study explores whether a 9-month supervised exercise program helps people with metastatic breast cancer and if it’s worth the cost. Researchers compared two groups: one doing twice-weekly supervised exercise and another getting usual care with basic activity advice and a tracker. The exercise group reported slightly better quality of life and lower overall costs, mainly because they needed less medical care and missed less work. The program cost less when supervised in small groups compared to one-on-one sessions. These findings suggest that exercise programs could improve patients’ lives while saving money, encouraging health systems to support such programs. This research could guide doctors and policymakers to include supervised exercise as a standard option for people with metastatic breast cancer, improving their well-being and reducing health care expenses. | We studied whether a 9-month exercise program, with trained professionals guiding patients with metastatic breast cancer, is worth the cost compared to usual care, where patients got general activity advice and a tracker. This research matters because we need to know if exercise programs are affordable and helpful for these patients. We looked at 357 patients from Europe and Australia, split into an exercise group and a usual care group. We checked their health costs, work productivity, and quality of life using questionnaires. The exercise program slightly improved quality of life and saved money, especially when done in small groups. These savings came from lower health care and work-related costs. Our findings suggest that exercise programs could be a smart choice for hospitals and patients, encouraging more support for such programs in cancer care. | We want to find out if a 9-month exercise program, guided by experts, helps people with advanced breast cancer and saves money compared to usual care. Researchers studied 357 patients in Europe and Australia, splitting them into two groups: one did supervised exercise twice a week, and the other got general activity advice and a tracker. The exercise program slightly improved patients’ quality of life and cost less overall, saving money on health care and work-related losses. Group exercise sessions saved even more. These results show that guided exercise is likely a smart choice for patients with advanced breast cancer, improving lives while cutting costs. This could encourage health systems to support such programs, helping more patients stay active and feel better. |
| JCO | 4 | 10.1200/JCO-24-02087 | Open AI | PURPOSE Cyclin-dependent kinase 4/6 inhibitors (CDK4/6i) combined with endocrine therapy (ET) are the standard first-line treatment for hormone receptor-positive (HR+), human epidermal growth factor receptor 2-negative (HER2-) advanced breast cancer (ABC); however, disease progression occurs in almost all patients and additional treatment options are needed. Herein, we report outcomes of the postMONARCH trial investigating a switch in ET with/without CDK4/6 inhibition with abemaciclib after disease progression on CDK4/6i. METHODS This double-blind, randomized phase III study enrolled patients with disease progression on previous CDK4/6i plus aromatase inhibitor as initial therapy for advanced disease or recurrence on/after adjuvant CDK4/6i + ET. Patients were randomly assigned (1:1) to abemaciclib + fulvestrant or placebo + fulvestrant. The primary end point was investigator-assessed progression-free survival (PFS). Secondary end points included PFS by blinded independent central review, objective response rate (ORR), and safety. RESULTS This study randomly assigned 368 patients (abemaciclib + fulvestrant, n = 182 placebo + fulvestrant, n = 186). At the primary analysis (258 events), the hazard ratio (HR) was 0.73 (95% CI, 0.57 to 0.95; nominal P = .017), with median PFS 6.0 (95% CI, 5.6 to 8.6) versus 5.3 (95% CI, 3.7 to 5.6) months and 6-month PFS rates of 50% and 37% in the abemaciclib + fulvestrant and placebo + fulvestrant arms, respectively. These results were supported by BICR-assessed PFS (HR, 0.55 [95% CI, 0.39 to 0.77]; nominal P < .001). A consistent treatment effect was seen across major clinical and genomic subgroups, including with/without ESR1 or PIK3CA mutations. Among patients with measurable disease, investigator-assessed ORR was improved with abemaciclib + fulvestrant versus placebo + fulvestrant (17% v 7%; nominal P = .015). No new safety signals were observed, with findings consistent with the known safety profile of abemaciclib. CONCLUSION Abemaciclib + fulvestrant significantly improved PFS after disease progression on previous CDK4/6i + ET in patients with HR+, HER2- ABC, offering an additional targeted therapy option for these patients. | This study tested a new treatment approach for people with advanced breast cancer that has grown despite standard therapy. Usually, patients with hormone-sensitive, HER2-negative advanced breast cancer are treated with a combination of hormone therapy and a type of medicine called a CDK4/6 inhibitor. However, the cancer often continues to progress, and new options are needed. In this trial, patients whose cancer had progressed were given either a combination of the drug abemaciclib with fulvestrant or fulvestrant alone. The results showed that adding abemaciclib helped patients live longer without their cancer worsening and increased the chance of shrinking tumors, without new safety concerns. These findings suggest that abemaciclib plus fulvestrant provides an effective next treatment for patients whose cancer progresses after first-line therapy, offering hope for improved disease control and quality of life. | Advanced breast cancer often stops responding to standard hormone treatments combined with certain targeted drugs, leaving patients with few options. This study tested whether switching hormone therapy and adding the drug abemaciclib could help patients whose cancer had progressed despite previous treatment. In a large clinical trial, 368 patients were randomly given either abemaciclib with hormone therapy or hormone therapy alone. Results showed that patients receiving abemaciclib had slower cancer growth, with more patients living without disease progression at six months compared with those on hormone therapy alone. The benefit was seen across different patient groups, including those with specific genetic changes. Side effects were consistent with what is already known about abemaciclib. These findings suggest that combining abemaciclib with hormone therapy offers an effective new treatment option for patients with advanced breast cancer who have exhausted previous therapies. | This study looked at a new treatment option for people with advanced breast cancer that grows despite standard hormone-based therapy. The researchers tested whether combining the drug abemaciclib with the hormone therapy fulvestrant could slow cancer growth after other similar treatments stopped working. In a clinical trial, 368 patients were randomly assigned to receive either the combination treatment or fulvestrant alone. The results showed that the combination treatment helped keep the cancer from growing for a longer time and improved the chances of tumor shrinkage, without adding unexpected side effects. These benefits were seen in many different patient groups, including those with certain genetic changes in their tumors. This research offers doctors and patients an additional therapy option when initial treatments fail, helping guide future care and improving outcomes for people with advanced breast cancer. |
| JCO | 4 | 10.1200/JCO-24-02088 | Claude | PURPOSE Cyclin-dependent kinase 4/6 inhibitors (CDK4/6i) combined with endocrine therapy (ET) are the standard first-line treatment for hormone receptor-positive (HR+), human epidermal growth factor receptor 2-negative (HER2-) advanced breast cancer (ABC); however, disease progression occurs in almost all patients and additional treatment options are needed. Herein, we report outcomes of the postMONARCH trial investigating a switch in ET with/without CDK4/6 inhibition with abemaciclib after disease progression on CDK4/6i. METHODS This double-blind, randomized phase III study enrolled patients with disease progression on previous CDK4/6i plus aromatase inhibitor as initial therapy for advanced disease or recurrence on/after adjuvant CDK4/6i + ET. Patients were randomly assigned (1:1) to abemaciclib + fulvestrant or placebo + fulvestrant. The primary end point was investigator-assessed progression-free survival (PFS). Secondary end points included PFS by blinded independent central review, objective response rate (ORR), and safety. RESULTS This study randomly assigned 368 patients (abemaciclib + fulvestrant, n = 182 placebo + fulvestrant, n = 186). At the primary analysis (258 events), the hazard ratio (HR) was 0.73 (95% CI, 0.57 to 0.95; nominal P = .017), with median PFS 6.0 (95% CI, 5.6 to 8.6) versus 5.3 (95% CI, 3.7 to 5.6) months and 6-month PFS rates of 50% and 37% in the abemaciclib + fulvestrant and placebo + fulvestrant arms, respectively. These results were supported by BICR-assessed PFS (HR, 0.55 [95% CI, 0.39 to 0.77]; nominal P < .001). A consistent treatment effect was seen across major clinical and genomic subgroups, including with/without ESR1 or PIK3CA mutations. Among patients with measurable disease, investigator-assessed ORR was improved with abemaciclib + fulvestrant versus placebo + fulvestrant (17% v 7%; nominal P = .015). No new safety signals were observed, with findings consistent with the known safety profile of abemaciclib. CONCLUSION Abemaciclib + fulvestrant significantly improved PFS after disease progression on previous CDK4/6i + ET in patients with HR+, HER2- ABC, offering an additional targeted therapy option for these patients. | "Breast cancer that spreads to other parts of the body is often treated with hormone-blocking drugs and medicines called CDK4/6 inhibitors. While these treatments work well at first, the cancer usually starts growing again after some time. This study tested whether adding a drug called abemaciclib to a hormone-blocking medicine called fulvestrant could help patients whose cancer had gotten worse despite previous treatment. Researchers studied 368 women with advanced breast cancer whose disease had progressed on earlier treatments. Half the patients got abemaciclib plus fulvestrant, while the other half got a fake pill plus fulvestrant. The results showed that patients who received abemaciclib lived longer without their cancer getting worse - about 6 months compared to 5.3 months for those who got the fake pill. More patients also saw their tumors shrink with the abemaciclib treatment. The side effects were manageable and similar to what doctors already know about this drug. This study gives doctors a new treatment option for patients whose breast cancer stops responding to standard treatments. | "Most women with advanced hormone-positive breast cancer first receive a drug combination that blocks cancer growth by targeting specific proteins. However, the cancer eventually grows despite this treatment, and doctors need new options. This study tested whether adding a drug called abemaciclib to a hormone therapy called fulvestrant could help patients whose cancer had already grown through the first treatment. Researchers studied 368 women whose breast cancer had gotten worse after the initial treatment. Half received abemaciclib plus fulvestrant, while the other half received fulvestrant alone. The women who got both drugs lived longer without their cancer growing compared to those who got fulvestrant alone - about 6 months versus 5.3 months. The combination also helped shrink tumors better than fulvestrant alone. Side effects were manageable and expected. This research shows that using abemaciclib with fulvestrant gives women with advanced breast cancer another effective treatment option when their first treatment stops working. | Breast cancer that spreads to other parts of the body is often treated with hormone-blocking drugs combined with medications called CDK4/6 inhibitors. While this treatment works well at first, the cancer almost always gets worse over time, and doctors need new treatment options. This study tested whether switching to a different combination of drugs could help patients whose cancer had grown despite previous treatment. Researchers gave 368 women either abemaciclib plus fulvestrant or a fake treatment plus fulvestrant to see which worked better. The results showed that women who received abemaciclib plus fulvestrant lived longer without their cancer getting worse compared to those who got the fake treatment. The cancer stayed controlled for an average of 6 months versus 5.3 months, and more tumors shrank with the real treatment. The side effects were manageable and similar to what doctors expected. This study proves that switching to abemaciclib plus fulvestrant gives patients with advanced breast cancer another effective treatment option when their first treatment stops working. |
| JCO | 4 | 10.1200/JCO-24-02089 | Gemini | PURPOSE Cyclin-dependent kinase 4/6 inhibitors (CDK4/6i) combined with endocrine therapy (ET) are the standard first-line treatment for hormone receptor-positive (HR+), human epidermal growth factor receptor 2-negative (HER2-) advanced breast cancer (ABC); however, disease progression occurs in almost all patients and additional treatment options are needed. Herein, we report outcomes of the postMONARCH trial investigating a switch in ET with/without CDK4/6 inhibition with abemaciclib after disease progression on CDK4/6i. METHODS This double-blind, randomized phase III study enrolled patients with disease progression on previous CDK4/6i plus aromatase inhibitor as initial therapy for advanced disease or recurrence on/after adjuvant CDK4/6i + ET. Patients were randomly assigned (1:1) to abemaciclib + fulvestrant or placebo + fulvestrant. The primary end point was investigator-assessed progression-free survival (PFS). Secondary end points included PFS by blinded independent central review, objective response rate (ORR), and safety. RESULTS This study randomly assigned 368 patients (abemaciclib + fulvestrant, n = 182 placebo + fulvestrant, n = 186). At the primary analysis (258 events), the hazard ratio (HR) was 0.73 (95% CI, 0.57 to 0.95; nominal P = .017), with median PFS 6.0 (95% CI, 5.6 to 8.6) versus 5.3 (95% CI, 3.7 to 5.6) months and 6-month PFS rates of 50% and 37% in the abemaciclib + fulvestrant and placebo + fulvestrant arms, respectively. These results were supported by BICR-assessed PFS (HR, 0.55 [95% CI, 0.39 to 0.77]; nominal P < .001). A consistent treatment effect was seen across major clinical and genomic subgroups, including with/without ESR1 or PIK3CA mutations. Among patients with measurable disease, investigator-assessed ORR was improved with abemaciclib + fulvestrant versus placebo + fulvestrant (17% v 7%; nominal P = .015). No new safety signals were observed, with findings consistent with the known safety profile of abemaciclib. CONCLUSION Abemaciclib + fulvestrant significantly improved PFS after disease progression on previous CDK4/6i + ET in patients with HR+, HER2- ABC, offering an additional targeted therapy option for these patients. | This research was conducted because the standard treatment for a specific type of advanced breast cancer eventually stops working, creating a need for new options. The study tested a drug combination of abemaciclib and fulvestrant to see if it could extend the time before the cancer started growing again in patients whose initial therapy had failed. The findings show that patients who received this new treatment lived significantly longer without their disease progressing compared to those who did not. This is a crucial finding for the medical community because it provides a new and effective treatment choice for patients and doctors, offering more hope and time when facing this challenging disease. | In this study, researchers investigated a new treatment option for people with advanced breast cancer that has grown despite earlier treatment with a similar type of medication. The study specifically focused on a combination of two drugs, abemaciclib and fulvestrant, to see if it could help patients whose cancer had become resistant to an initial treatment. The findings showed that this new drug combination significantly slowed the growth of the cancer. This is important because it provides doctors and patients with another targeted therapy to use when the standard first-line treatment is no longer effective, potentially extending the time before the cancer worsens. | Abemaciclib, when combined with fulvestrant, has shown to significantly improve progression-free survival in patients with advanced breast cancer. The researchers aimed to provide an additional targeted therapy option for patients whose cancer progressed after initial treatment with a CDK4/6 inhibitor and endocrine therapy. This study randomly assigned patients to receive either abemaciclib plus fulvestrant or a placebo plus fulvestrant. The findings suggest that the abemaciclib combination is a viable treatment for patients with hormone receptor-positive, human epidermal growth factor receptor 2-negative advanced breast cancer, offering an important new strategy for doctors and patients facing this condition. The study results are a crucial step toward providing more treatment options and better outcomes for those who have exhausted their initial therapy. |
| JCO | 4 | 10.1200/JCO-24-02090 | DeepSeek | PURPOSE Cyclin-dependent kinase 4/6 inhibitors (CDK4/6i) combined with endocrine therapy (ET) are the standard first-line treatment for hormone receptor-positive (HR+), human epidermal growth factor receptor 2-negative (HER2-) advanced breast cancer (ABC); however, disease progression occurs in almost all patients and additional treatment options are needed. Herein, we report outcomes of the postMONARCH trial investigating a switch in ET with/without CDK4/6 inhibition with abemaciclib after disease progression on CDK4/6i. METHODS This double-blind, randomized phase III study enrolled patients with disease progression on previous CDK4/6i plus aromatase inhibitor as initial therapy for advanced disease or recurrence on/after adjuvant CDK4/6i + ET. Patients were randomly assigned (1:1) to abemaciclib + fulvestrant or placebo + fulvestrant. The primary end point was investigator-assessed progression-free survival (PFS). Secondary end points included PFS by blinded independent central review, objective response rate (ORR), and safety. RESULTS This study randomly assigned 368 patients (abemaciclib + fulvestrant, n = 182 placebo + fulvestrant, n = 186). At the primary analysis (258 events), the hazard ratio (HR) was 0.73 (95% CI, 0.57 to 0.95; nominal P = .017), with median PFS 6.0 (95% CI, 5.6 to 8.6) versus 5.3 (95% CI, 3.7 to 5.6) months and 6-month PFS rates of 50% and 37% in the abemaciclib + fulvestrant and placebo + fulvestrant arms, respectively. These results were supported by BICR-assessed PFS (HR, 0.55 [95% CI, 0.39 to 0.77]; nominal P < .001). A consistent treatment effect was seen across major clinical and genomic subgroups, including with/without ESR1 or PIK3CA mutations. Among patients with measurable disease, investigator-assessed ORR was improved with abemaciclib + fulvestrant versus placebo + fulvestrant (17% v 7%; nominal P = .015). No new safety signals were observed, with findings consistent with the known safety profile of abemaciclib. CONCLUSION Abemaciclib + fulvestrant significantly improved PFS after disease progression on previous CDK4/6i + ET in patients with HR+, HER2- ABC, offering an additional targeted therapy option for these patients. | This study tested whether the drug abemaciclib, combined with fulvestrant, could help patients with advanced breast cancer that had worsened after initial treatment with similar drugs (CDK4/6 inhibitors). Researchers compared this combination to fulvestrant alone in 368 patients. Results showed that adding abemaciclib helped slow cancer growth longer—6 months vs. 5.3 months—and more patients saw their tumors shrink (17% vs. 7%). Side effects matched what was already known about abemaciclib. These findings are important because they offer a new option for patients whose cancer progresses after standard treatment, giving them more time before the disease advances. This could improve care for many people with hormone-sensitive, HER2-negative advanced breast cancer. | This study tested whether the drug abemaciclib, combined with fulvestrant, could help patients with advanced breast cancer that had stopped responding to standard treatment (a *CDK4/6 inhibitor* plus hormone therapy). The trial included 368 patients whose cancer had worsened after initial treatment. Half received abemaciclib + fulvestrant, while the other half got fulvestrant alone. Results showed that adding abemaciclib helped slow cancer growth longer (6 months vs. 5.3 months) and improved response rates (17% vs. 7%) without new safety concerns. This means abemaciclib + fulvestrant could be a helpful next option for patients when their first treatment stops working, giving them more time before the cancer progresses. | This study tested whether combining the drugs abemaciclib and fulvestrant could help women with advanced breast cancer that had stopped responding to standard treatment (a CDK4/6 inhibitor plus hormone therapy). In a trial of 368 patients, half received the new drug combo, while the other half got fulvestrant alone. Results showed that the combination slowed cancer growth longer (6 months vs. 5.3 months) and helped more patients see their tumors shrink (17% vs. 7%). Side effects were similar to what was already known about abemaciclib. These findings suggest that this combo could be a helpful next treatment for women whose cancer worsens after initial therapy, giving them more time before the disease progresses. |
| JCO | 4 | 10.1200/JCO-24-02091 | Grok | PURPOSE Cyclin-dependent kinase 4/6 inhibitors (CDK4/6i) combined with endocrine therapy (ET) are the standard first-line treatment for hormone receptor-positive (HR+), human epidermal growth factor receptor 2-negative (HER2-) advanced breast cancer (ABC); however, disease progression occurs in almost all patients and additional treatment options are needed. Herein, we report outcomes of the postMONARCH trial investigating a switch in ET with/without CDK4/6 inhibition with abemaciclib after disease progression on CDK4/6i. METHODS This double-blind, randomized phase III study enrolled patients with disease progression on previous CDK4/6i plus aromatase inhibitor as initial therapy for advanced disease or recurrence on/after adjuvant CDK4/6i + ET. Patients were randomly assigned (1:1) to abemaciclib + fulvestrant or placebo + fulvestrant. The primary end point was investigator-assessed progression-free survival (PFS). Secondary end points included PFS by blinded independent central review, objective response rate (ORR), and safety. RESULTS This study randomly assigned 368 patients (abemaciclib + fulvestrant, n = 182 placebo + fulvestrant, n = 186). At the primary analysis (258 events), the hazard ratio (HR) was 0.73 (95% CI, 0.57 to 0.95; nominal P = .017), with median PFS 6.0 (95% CI, 5.6 to 8.6) versus 5.3 (95% CI, 3.7 to 5.6) months and 6-month PFS rates of 50% and 37% in the abemaciclib + fulvestrant and placebo + fulvestrant arms, respectively. These results were supported by BICR-assessed PFS (HR, 0.55 [95% CI, 0.39 to 0.77]; nominal P < .001). A consistent treatment effect was seen across major clinical and genomic subgroups, including with/without ESR1 or PIK3CA mutations. Among patients with measurable disease, investigator-assessed ORR was improved with abemaciclib + fulvestrant versus placebo + fulvestrant (17% v 7%; nominal P = .015). No new safety signals were observed, with findings consistent with the known safety profile of abemaciclib. CONCLUSION Abemaciclib + fulvestrant significantly improved PFS after disease progression on previous CDK4/6i + ET in patients with HR+, HER2- ABC, offering an additional targeted therapy option for these patients. | This study, called postMONARCH, explores a new treatment option for women with advanced breast cancer that has worsened despite earlier therapy. Researchers want to see if switching to a drug called abemaciclib, combined with fulvestrant, can slow cancer growth better than fulvestrant alone. They tested this in 368 women, finding that those receiving both drugs went about six months without their cancer growing, compared to five months for those on fulvestrant alone. This improvement was consistent across different patient groups. The findings suggest this drug combination could give doctors a better way to help women with this type of breast cancer, potentially delaying the need for other treatments. This could lead to more research on similar therapies, offering hope for improved care. | This study, called postMONARCH, explores a new treatment option for women with advanced breast cancer that is hormone receptor-positive and HER2-negative, when their cancer worsens after initial therapy with a specific type of drug called a CDK4/6 inhibitor combined with hormone therapy. The researchers aim to see if switching to a different hormone therapy, fulvestrant, with or without continuing the CDK4/6 inhibitor abemaciclib, can slow cancer growth. They found that adding abemaciclib to fulvestrant helped patients live longer without their cancer progressing compared to fulvestrant alone. These findings could give doctors a new way to treat this type of breast cancer, potentially improving patients’ lives by delaying cancer growth. This research may guide future studies to improve treatments for breast cancer patients. | This study, called postMONARCH, explores a new treatment option for people with advanced breast cancer that is hormone receptor-positive and HER2-negative, when their cancer worsens after initial therapy with certain drugs. Researchers want to see if switching to a drug called abemaciclib combined with fulvestrant can slow cancer growth better than fulvestrant alone. They found that patients receiving both drugs lived about six months without their cancer growing, compared to about five months for those on fulvestrant alone. This improvement could give doctors a new way to help patients whose cancer progresses after standard treatments. The findings may guide future research to improve care for breast cancer patients, offering hope for better outcomes. |
| JCO | 5 | 10.1200/JCO.24.00343 | Open AI | PURPOSE Guidelines recommend cardiovascular (CV) risk assessment and counseling for cancer survivors. This study evaluated the automated heart-health assessment (AH-HA) clinical decision support tool to promote provider-patient CV health (CVH) discussions in outpatient oncology. METHODS The AH-HA trial (WF-1804CD), coordinated by the Wake Forest National Cancer Institute Community Oncology Research Program Research Base, randomized practices to the AH-HA tool or usual care (UC) and enrolled survivors receiving routine care >= 6 months after curative cancer treatment. The tool displayed American Heart Association Life's Simple 7 CVH factors (BMI, physical activity, diet, smoking status, blood pressure, cholesterol, and glucose), populated from the electronic health record (EHR), alongside cancer treatments received with cardiotoxic potential. The primary end point was survivor-reported discussion of nonideal or missing CVH factors. A mixed-effects logistic regression model assessed the effect of AH-HA on CVH discussions, adjusting for practice. RESULTS Five UC and four AH-HA practices enrolled 645 survivors (82% breast, 8% endometrial, 5% colorectal, and 5% lymphoma, prostate, or multiple types) from October 1, 2020, to February 28, 2023. Most survivors were female (96%; 84% White/non-Hispanic, 8% Black; 3% Hispanic). Nearly all survivors (98%) in AH-HA practices reported a discussion for >= 1 nonideal or missing CVH factor compared with 55% in UC (P < .001). The average number of survivor-reported factors discussed was higher in AH-HA compared with UC (mean, 4.06 v 1.27; P < .001), as were EHR-documented discussions (3.83 v 0.77; P = .03). Survivors in AH-HA practices were also significantly more likely to report a recommendation to see a primary care provider (39%) compared with UC practices (25%, P = .02). Reported recommendations to see a cardiologist were low (approximately 6%) and did not differ between groups. CONCLUSION The AH-HA tool was effective at promoting CVH discussions during routine follow-up care for survivors and recommendations to consult primary care. | Cancer survivors face a higher risk of heart problems, yet discussions about heart health are often overlooked during routine follow-up care. This study tested an electronic tool called AH-HA, which uses patients’ health records to show key heart health factors such as weight, activity, diet, blood pressure, cholesterol, and blood sugar, alongside information about past cancer treatments that could affect the heart. The goal was to see if the tool helped doctors and patients talk more about heart health. In nine outpatient cancer clinics, survivors using AH-HA reported many more conversations about heart health than those receiving usual care. They were also more likely to be advised to see their primary care doctor. These findings suggest that using simple electronic tools can make heart health discussions more common, helping cancer survivors take steps to protect their heart and overall well-being. | Cancer survivors can face higher risks for heart problems, but discussions about heart health do not always happen during follow-up care. This study tested an electronic tool called AH-HA that automatically shows heart health information, such as body weight, exercise, diet, blood pressure, cholesterol, and blood sugar, alongside any cancer treatments that might affect the heart. In nine community oncology practices, survivors who used the tool reported more conversations with their doctors about heart health than those receiving usual care. They also received more advice about seeing a primary care doctor for heart concerns. This research shows that using electronic health records in this way can help doctors and cancer survivors talk more about heart health. By encouraging these discussions, the tool may help survivors prevent heart problems and improve long-term well-being after cancer treatment. | Cancer survivors can face higher risks for heart problems, but heart health is not always discussed during follow-up visits. This study tested a computer tool called the automated heart-health assessment that helps doctors review heart health factors with their patients. The tool shows information like weight, blood pressure, cholesterol, blood sugar, exercise, diet, and smoking, along with past cancer treatments that could affect the heart. The study compared clinics using the tool with clinics providing usual care. Results showed that nearly all patients using the tool talked with their doctor about at least one heart health issue, compared with about half in usual care. Patients also received more advice to see their regular doctor for heart concerns. These findings suggest that using such a tool can make heart health discussions more common and help cancer survivors manage risks earlier. |
| JCO | 5 | 10.1200/JCO.24.00344 | Claude | PURPOSE Guidelines recommend cardiovascular (CV) risk assessment and counseling for cancer survivors. This study evaluated the automated heart-health assessment (AH-HA) clinical decision support tool to promote provider-patient CV health (CVH) discussions in outpatient oncology. METHODS The AH-HA trial (WF-1804CD), coordinated by the Wake Forest National Cancer Institute Community Oncology Research Program Research Base, randomized practices to the AH-HA tool or usual care (UC) and enrolled survivors receiving routine care >= 6 months after curative cancer treatment. The tool displayed American Heart Association Life's Simple 7 CVH factors (BMI, physical activity, diet, smoking status, blood pressure, cholesterol, and glucose), populated from the electronic health record (EHR), alongside cancer treatments received with cardiotoxic potential. The primary end point was survivor-reported discussion of nonideal or missing CVH factors. A mixed-effects logistic regression model assessed the effect of AH-HA on CVH discussions, adjusting for practice. RESULTS Five UC and four AH-HA practices enrolled 645 survivors (82% breast, 8% endometrial, 5% colorectal, and 5% lymphoma, prostate, or multiple types) from October 1, 2020, to February 28, 2023. Most survivors were female (96%; 84% White/non-Hispanic, 8% Black; 3% Hispanic). Nearly all survivors (98%) in AH-HA practices reported a discussion for >= 1 nonideal or missing CVH factor compared with 55% in UC (P < .001). The average number of survivor-reported factors discussed was higher in AH-HA compared with UC (mean, 4.06 v 1.27; P < .001), as were EHR-documented discussions (3.83 v 0.77; P = .03). Survivors in AH-HA practices were also significantly more likely to report a recommendation to see a primary care provider (39%) compared with UC practices (25%, P = .02). Reported recommendations to see a cardiologist were low (approximately 6%) and did not differ between groups. CONCLUSION The AH-HA tool was effective at promoting CVH discussions during routine follow-up care for survivors and recommendations to consult primary care. | Cancer survivors face higher risks of heart problems due to their treatments, but doctors often don't discuss heart health during follow-up visits. Researchers created a computer tool called AH-HA that helps doctors talk about heart health with cancer survivors. The tool shows important heart health factors like weight, exercise, diet, smoking, blood pressure, cholesterol, and blood sugar levels from patient records. It also shows which cancer treatments might have hurt the heart. The study tested this tool with 645 cancer survivors at nine medical practices over two years. Results showed the tool worked very well. Almost all survivors (98%) using the tool discussed at least one heart health topic with their doctor, compared to only 55% without the tool. Survivors using the tool talked about four times more heart health factors on average. They were also more likely to be told to see their family doctor for heart health care. This simple computer tool helps ensure cancer survivors get the heart health discussions they need during routine visits. | Cancer survivors often face heart health risks from their treatments, but doctors don't always talk about these risks during follow-up visits. Researchers created a computer tool called AH-HA that helps cancer doctors discuss heart health with their patients. The tool pulls information from medical records about seven key heart health factors like weight, exercise, diet, smoking, blood pressure, and blood sugar levels. It also shows which cancer treatments might have hurt the heart. The study tested this tool with 645 cancer survivors at nine medical offices over two years. Results showed that nearly all patients (98%) whose doctors used the AH-HA tool talked about heart health problems, compared to only 55% of patients whose doctors didn't use the tool. Patients with the tool also got more advice to see their family doctor for heart health care. This simple computer tool helps cancer doctors remember to discuss important heart health topics, which could prevent future heart problems in cancer survivors. | Cancer survivors often face heart problems after treatment, but doctors don't always talk about heart health during follow-up visits. This study tested a new computer tool called AH-HA that helps doctors discuss heart health with cancer survivors. The tool shows important heart health information like weight, exercise, diet, smoking, blood pressure, cholesterol, and blood sugar levels right in the patient's medical records. Researchers worked with nine cancer clinics and 645 cancer survivors to see if the tool worked better than usual care. They found that almost all survivors (98%) who saw doctors using the AH-HA tool talked about heart health problems, compared to only 55% who got usual care. Doctors using the tool discussed more heart health topics and were more likely to suggest patients see their regular doctor for heart care. This simple computer tool helps make sure cancer survivors get the heart health care they need during routine cancer follow-up visits. |
| JCO | 5 | 10.1200/JCO.24.00345 | Gemini | PURPOSE Guidelines recommend cardiovascular (CV) risk assessment and counseling for cancer survivors. This study evaluated the automated heart-health assessment (AH-HA) clinical decision support tool to promote provider-patient CV health (CVH) discussions in outpatient oncology. METHODS The AH-HA trial (WF-1804CD), coordinated by the Wake Forest National Cancer Institute Community Oncology Research Program Research Base, randomized practices to the AH-HA tool or usual care (UC) and enrolled survivors receiving routine care >= 6 months after curative cancer treatment. The tool displayed American Heart Association Life's Simple 7 CVH factors (BMI, physical activity, diet, smoking status, blood pressure, cholesterol, and glucose), populated from the electronic health record (EHR), alongside cancer treatments received with cardiotoxic potential. The primary end point was survivor-reported discussion of nonideal or missing CVH factors. A mixed-effects logistic regression model assessed the effect of AH-HA on CVH discussions, adjusting for practice. RESULTS Five UC and four AH-HA practices enrolled 645 survivors (82% breast, 8% endometrial, 5% colorectal, and 5% lymphoma, prostate, or multiple types) from October 1, 2020, to February 28, 2023. Most survivors were female (96%; 84% White/non-Hispanic, 8% Black; 3% Hispanic). Nearly all survivors (98%) in AH-HA practices reported a discussion for >= 1 nonideal or missing CVH factor compared with 55% in UC (P < .001). The average number of survivor-reported factors discussed was higher in AH-HA compared with UC (mean, 4.06 v 1.27; P < .001), as were EHR-documented discussions (3.83 v 0.77; P = .03). Survivors in AH-HA practices were also significantly more likely to report a recommendation to see a primary care provider (39%) compared with UC practices (25%, P = .02). Reported recommendations to see a cardiologist were low (approximately 6%) and did not differ between groups. CONCLUSION The AH-HA tool was effective at promoting CVH discussions during routine follow-up care for survivors and recommendations to consult primary care. | Cancer survivors often face a higher risk of heart problems due to their cancer treatments. To address this, a new tool called the automated heart-health assessment (AH-HA) was developed and tested. This tool, which works with a patient's electronic health record, helps doctors and nurses discuss heart health with their cancer survivor patients. We found that patients whose doctors used the AH-HA tool were much more likely to talk about important heart-health factors like diet, exercise, and blood pressure. The tool also encouraged doctors to recommend that their patients follow up with their primary care physician. These findings show that the AH-HA tool is a simple and effective way to improve heart health care for cancer survivors. | Some cancer treatments, such as chemotherapy and radiation, can damage the heart, putting cancer survivors at a higher risk for cardiovascular disease. This study tested a new electronic health record tool designed to help doctors talk with their patients about heart health. The tool, called AH-HA, automatically highlights seven important health factors, like blood pressure, cholesterol, and exercise habits, for each patient. The study found that when doctors used the AH-HA tool, they were much more likely to discuss these heart health factors with their patients. This led to more conversations about maintaining a healthy lifestyle and more recommendations for patients to follow up with their primary care physician. These findings are important for the medical community because they demonstrate that simple digital tools can improve heart health care for cancer survivors, helping them live longer, healthier lives. | Cancer survivors can face a higher risk of heart problems, making it crucial for doctors to discuss heart health during check-ups. This study tested a new computer tool that automatically pulls a patient's health information from their electronic medical records. The goal was to see if this tool could encourage more heart health conversations between doctors and cancer survivors. Our results show that when doctors used the tool, they had important conversations about heart health with nearly all their patients, a much higher rate than doctors without the tool. The tool also led to more recommendations for patients to follow up with their regular doctors. This research highlights how simple technology can improve communication and care for cancer survivors, ultimately helping them stay healthier. |
| JCO | 5 | 10.1200/JCO.24.00346 | DeepSeek | PURPOSE Guidelines recommend cardiovascular (CV) risk assessment and counseling for cancer survivors. This study evaluated the automated heart-health assessment (AH-HA) clinical decision support tool to promote provider-patient CV health (CVH) discussions in outpatient oncology. METHODS The AH-HA trial (WF-1804CD), coordinated by the Wake Forest National Cancer Institute Community Oncology Research Program Research Base, randomized practices to the AH-HA tool or usual care (UC) and enrolled survivors receiving routine care >= 6 months after curative cancer treatment. The tool displayed American Heart Association Life's Simple 7 CVH factors (BMI, physical activity, diet, smoking status, blood pressure, cholesterol, and glucose), populated from the electronic health record (EHR), alongside cancer treatments received with cardiotoxic potential. The primary end point was survivor-reported discussion of nonideal or missing CVH factors. A mixed-effects logistic regression model assessed the effect of AH-HA on CVH discussions, adjusting for practice. RESULTS Five UC and four AH-HA practices enrolled 645 survivors (82% breast, 8% endometrial, 5% colorectal, and 5% lymphoma, prostate, or multiple types) from October 1, 2020, to February 28, 2023. Most survivors were female (96%; 84% White/non-Hispanic, 8% Black; 3% Hispanic). Nearly all survivors (98%) in AH-HA practices reported a discussion for >= 1 nonideal or missing CVH factor compared with 55% in UC (P < .001). The average number of survivor-reported factors discussed was higher in AH-HA compared with UC (mean, 4.06 v 1.27; P < .001), as were EHR-documented discussions (3.83 v 0.77; P = .03). Survivors in AH-HA practices were also significantly more likely to report a recommendation to see a primary care provider (39%) compared with UC practices (25%, P = .02). Reported recommendations to see a cardiologist were low (approximately 6%) and did not differ between groups. CONCLUSION The AH-HA tool was effective at promoting CVH discussions during routine follow-up care for survivors and recommendations to consult primary care. | Cancer survivors often face heart health risks, but doctors don’t always discuss these during check-ups. This study tested an electronic health record tool (AH-HA) that automatically shows a patient’s heart health risks—like blood pressure, diet, or smoking—alongside their cancer treatment history. Researchers compared clinics using AH-HA to those using usual care. They found that 98% of survivors at AH-HA clinics talked with their doctors about heart health, compared to only 55% at regular clinics. Patients at AH-HA clinics also discussed more heart health issues and were more likely to be sent to a primary care doctor for follow-up. The tool helped doctors and survivors focus on heart health, which could prevent future problems. This simple tech fix could improve long-term care for cancer survivors. | Cancer survivors often face heart health risks, but doctors don’t always discuss these during check-ups. This study tested a tool called AH-HA, which automatically flags heart health risks—like blood pressure, diet, or smoking—in a patient’s electronic health record. Researchers compared cancer survivors who got care with AH-HA to those who got usual care. They found that nearly all survivors (98%) using AH-HA talked with their doctors about heart health, compared to only 55% without the tool. AH-HA also led to more advice to see a primary care doctor for heart health. The tool worked well for survivors of breast, endometrial, and other cancers, helping doctors spot and discuss risks more often. This could improve long-term health for cancer survivors by catching heart problems early. | Cancer survivors often face heart health risks, but doctors don’t always discuss these during check-ups. This study tested a simple computer tool (AH-HA) that automatically shows heart health risks—like blood pressure, diet, and smoking—based on patient records. The tool helped doctors and cancer survivors talk more about heart health during visits. In clinics using AH-HA, 98% of survivors discussed at least one heart risk, compared to only 55% in clinics without it. Survivors also got more advice to see their regular doctor for heart care. While few were sent to heart specialists, the tool made a big difference in starting important conversations. This could help cancer survivors stay healthier by catching heart risks early. |
| JCO | 5 | 10.1200/JCO.24.00347 | Grok | PURPOSE Guidelines recommend cardiovascular (CV) risk assessment and counseling for cancer survivors. This study evaluated the automated heart-health assessment (AH-HA) clinical decision support tool to promote provider-patient CV health (CVH) discussions in outpatient oncology. METHODS The AH-HA trial (WF-1804CD), coordinated by the Wake Forest National Cancer Institute Community Oncology Research Program Research Base, randomized practices to the AH-HA tool or usual care (UC) and enrolled survivors receiving routine care >= 6 months after curative cancer treatment. The tool displayed American Heart Association Life's Simple 7 CVH factors (BMI, physical activity, diet, smoking status, blood pressure, cholesterol, and glucose), populated from the electronic health record (EHR), alongside cancer treatments received with cardiotoxic potential. The primary end point was survivor-reported discussion of nonideal or missing CVH factors. A mixed-effects logistic regression model assessed the effect of AH-HA on CVH discussions, adjusting for practice. RESULTS Five UC and four AH-HA practices enrolled 645 survivors (82% breast, 8% endometrial, 5% colorectal, and 5% lymphoma, prostate, or multiple types) from October 1, 2020, to February 28, 2023. Most survivors were female (96%; 84% White/non-Hispanic, 8% Black; 3% Hispanic). Nearly all survivors (98%) in AH-HA practices reported a discussion for >= 1 nonideal or missing CVH factor compared with 55% in UC (P < .001). The average number of survivor-reported factors discussed was higher in AH-HA compared with UC (mean, 4.06 v 1.27; P < .001), as were EHR-documented discussions (3.83 v 0.77; P = .03). Survivors in AH-HA practices were also significantly more likely to report a recommendation to see a primary care provider (39%) compared with UC practices (25%, P = .02). Reported recommendations to see a cardiologist were low (approximately 6%) and did not differ between groups. CONCLUSION The AH-HA tool was effective at promoting CVH discussions during routine follow-up care for survivors and recommendations to consult primary care. | This study tests a new tool called the Automated Heart-Health Assessment to help cancer survivors and their doctors talk about heart health. Many cancer survivors face heart risks after treatment, so researchers want to see if this tool, which uses health records to show factors like weight, exercise, and diet, encourages these discussions. The study compared clinics using the tool with those providing usual care. Findings show that survivors in clinics using the tool were much more likely to talk about heart health with their doctors and get advice to see a primary care doctor. These results could help the research community create better ways to protect cancer survivors’ hearts, ensuring doctors and patients work together to keep hearts healthy after cancer treatment. | This study, called the AH-HA trial, tested a new tool to help cancer survivors and their doctors talk about heart health in community oncology clinics. Many cancer survivors face heart risks after treatment, so the tool pulls information from medical records to show factors like weight, exercise, diet, smoking, blood pressure, cholesterol, and blood sugar. It also highlights cancer treatments that might affect the heart. The study included 645 survivors, mostly women with breast cancer, and compared clinics using the tool to those using standard care. The tool led to more heart health discussions, with 98% of survivors in tool-using clinics talking about at least one heart health issue, compared to 55% in regular clinics. It also increased suggestions to see a primary care doctor. This tool could help doctors better support survivors’ heart health, improving their overall care. | This study, called WF-1804CD, explored a new tool called the Automated Heart-Health Assessment (AH-HA) to help cancer survivors and their doctors talk about heart health. Many cancer survivors face heart problems after treatment, so researchers wanted to see if this tool could make these discussions easier. The tool uses information from medical records to show heart health factors like weight, exercise, and smoking habits. Practices using AH-HA were compared to those using standard care. Results showed that 98% of survivors in AH-HA practices talked about heart health issues with their doctors, compared to only 55% in standard care. They also discussed more heart health topics and were more likely to be advised to see a primary care doctor. This tool could help doctors and survivors work together to protect heart health, improving care for cancer survivors everywhere. |
| NatRevCan | 1 | 10.1038/s41568-025-00810-2 | Open AI | Phosphoinositide kinases, extending beyond the well-known phosphoinositide 3-kinase (PI3K), are key players in the dynamic and site-specific phosphorylation of lipid phosphoinositides. Unlike PI3Ks, phosphatidylinositol 4-kinases (PI4Ks) and phosphatidylinositol phosphate kinases (PIPKs) do not usually exhibit mutational alterations, but mostly show altered expression in tumours, orchestrating a broad spectrum of signalling, metabolic and immune processes, all of which are crucial in the pathogenesis of cancer. Dysregulation of PI4Ks and PIPKs has been associated with various malignancies, which has sparked considerable interest towards their therapeutic targeting. In this Review we summarize the current understanding of the lesser-studied phosphoinositide kinase families, PI4K and PIPK, focusing on their functions and relevance in cancer. In addition, we provide an overview of ongoing efforts driving the preclinical and clinical development of phosphoinositide kinase-targeting molecules. | This study explores a group of enzymes called phosphoinositide kinases, which help control important cell processes by modifying fats inside cells. While one type, PI3K, is well studied, other kinases like PI4Ks and PIPKs have been less understood. These enzymes are not usually changed by mutations in cancer but are often produced in unusual amounts, affecting how cells grow, use energy, and interact with the immune system. Problems with these enzymes have been linked to many types of cancer. By summarizing what is known about PI4Ks and PIPKs, this work highlights their important roles in cancer and explains why scientists are trying to develop new treatments that target them. Understanding these enzymes better could lead to new therapies and improve how we treat cancer in the future. | This study explores a group of enzymes called phosphoinositide kinases, which help control important cell signals and processes. While one type of these enzymes is well-known in cancer research, others, called PI4Ks and PIPKs, have received less attention. These enzymes are not usually mutated in cancer but are often found at unusual levels in tumors, affecting cell growth, metabolism, and the immune system. Understanding how these enzymes work in cancer can reveal new ways to slow or stop tumor growth. Researchers are now studying drugs that target these enzymes, with some already being tested in early studies. By highlighting the roles of these lesser-known enzymes and ongoing drug development, this review may guide future cancer research and treatment strategies, offering hope for more effective therapies. | This study looks at special proteins called phosphoinositide kinases, which help control important cell activities by adding small chemical tags to fats in cell membranes. While one type, PI3K, has been widely studied in cancer, other types called PI4Ks and PIPKs are less understood. These proteins do not usually have genetic mutations but can be present at unusual levels in tumors, affecting cell growth, metabolism, and immune responses. Changes in their activity are linked to many cancers, making them potential targets for new treatments. This review explains what we currently know about these lesser-studied kinases, how they contribute to cancer, and highlights ongoing research to develop drugs that can block their harmful effects. Understanding these proteins better could help scientists design new therapies and improve outcomes for cancer patients. |
| NatRevCan | 1 | 10.1038/s41568-025-00810-3 | Claude | Phosphoinositide kinases, extending beyond the well-known phosphoinositide 3-kinase (PI3K), are key players in the dynamic and site-specific phosphorylation of lipid phosphoinositides. Unlike PI3Ks, phosphatidylinositol 4-kinases (PI4Ks) and phosphatidylinositol phosphate kinases (PIPKs) do not usually exhibit mutational alterations, but mostly show altered expression in tumours, orchestrating a broad spectrum of signalling, metabolic and immune processes, all of which are crucial in the pathogenesis of cancer. Dysregulation of PI4Ks and PIPKs has been associated with various malignancies, which has sparked considerable interest towards their therapeutic targeting. In this Review we summarize the current understanding of the lesser-studied phosphoinositide kinase families, PI4K and PIPK, focusing on their functions and relevance in cancer. In addition, we provide an overview of ongoing efforts driving the preclinical and clinical development of phosphoinositide kinase-targeting molecules. | Cells use special enzymes called phosphoinositide kinases to control many important processes. Scientists have studied one type called PI3K for years, but two other types - PI4K and PIPK - are just as important but less understood. These enzymes help cells talk to each other, use energy, and fight disease. In cancer, these enzymes don't usually have broken genes like PI3K does. Instead, cancer cells make too much or too little of them. When PI4K and PIPK enzymes work wrong, they can help cancer grow and spread. This makes them good targets for new cancer treatments. Scientists are now studying how these enzymes work in cancer and testing new drugs that can block them. Understanding these enzymes better could lead to new ways to treat cancer patients. This research is important because it opens up fresh approaches to fighting cancer beyond the treatments we already have. | Cancer cells use special proteins called enzymes to help them grow and spread. This study looks at two groups of these enzymes that work inside cells to control important processes. These enzymes are called PI4K and PIPK, and they help control how cells talk to each other, use energy, and fight disease. Unlike some other cancer-causing proteins that have broken parts, these enzymes usually work normally but are made in wrong amounts in cancer cells. When cells make too much or too little of these enzymes, it can lead to different types of cancer. Scientists want to create new medicines that can target these enzymes to stop cancer from growing. This research is important because it helps doctors understand how cancer works at the basic level and gives them new ideas for treatments. By learning more about these enzymes, researchers hope to develop better drugs that can help cancer patients by stopping tumor growth and spread. | Cancer cells need special fat molecules called phosphoinositides to grow and spread throughout the body. These molecules are made by special enzymes called phosphoinositide kinases, which work like tiny factories inside cells. While scientists have studied one type of these enzymes for many years, two other important types called PI4K and PIPK have received much less attention. Unlike other cancer-causing enzymes that become damaged through mutations, PI4K and PIPK enzymes usually remain normal but are produced in wrong amounts in cancer cells. When these enzymes make too much or too little of their fat molecule products, they disrupt important cell processes including how cells communicate, use energy, and fight disease. This disruption helps cancer develop and spread. Understanding how these overlooked enzymes contribute to cancer opens new possibilities for treatment. Researchers are now developing new medicines that target these enzymes, offering hope for better cancer therapies. This research could lead to more effective treatments with fewer side effects for cancer patients. |
| NatRevCan | 1 | 10.1038/s41568-025-00810-4 | Gemini | Phosphoinositide kinases, extending beyond the well-known phosphoinositide 3-kinase (PI3K), are key players in the dynamic and site-specific phosphorylation of lipid phosphoinositides. Unlike PI3Ks, phosphatidylinositol 4-kinases (PI4Ks) and phosphatidylinositol phosphate kinases (PIPKs) do not usually exhibit mutational alterations, but mostly show altered expression in tumours, orchestrating a broad spectrum of signalling, metabolic and immune processes, all of which are crucial in the pathogenesis of cancer. Dysregulation of PI4Ks and PIPKs has been associated with various malignancies, which has sparked considerable interest towards their therapeutic targeting. In this Review we summarize the current understanding of the lesser-studied phosphoinositide kinase families, PI4K and PIPK, focusing on their functions and relevance in cancer. In addition, we provide an overview of ongoing efforts driving the preclinical and clinical development of phosphoinositide kinase-targeting molecules. | This research explores a group of lesser-known enzymes, called phosphoinositide kinases, that play a vital role in the development of cancer. While one type, phosphoinositide 3-kinase, is a common cancer target, two other families, phosphoinositide 4-kinases and phosphatidylinositol phosphate kinases, are less understood. This study aims to provide a comprehensive overview of how these specific kinases contribute to cancer growth and survival. By summarizing their functions and relevance in various cancers, this review helps highlight these enzymes as promising new targets for cancer therapies. The findings are important for guiding the development of new drugs and advancing cancer treatment strategies. | This research looks at a group of enzymes called phosphoinositide kinases, which are important for cell function. While one type, phosphoinositide 3-kinase, is well known, this study focuses on two less-understood types, phosphoinositide 4-kinase and phosphatidylinositol phosphate kinase. We know that in many cancers, the levels of these enzymes are not normal, which affects cell signaling and other processes that are crucial for cancer growth. This work explains how these enzymes work and their role in different cancers. By better understanding these enzymes, scientists can develop new medicines that specifically target them to slow or stop cancer, offering new hope for treatments beyond what is currently available. | This study explores a family of enzymes called phosphoinositide kinases, which are important for cell function. While one of these enzymes, PI3K, is a well-known target in cancer, others called PI4Ks and PIPKs are less understood. We know these enzymes are often overactive in tumors, affecting how cancer cells grow, their metabolism, and their ability to hide from the immune system. This research aims to summarize our current knowledge about these under-studied enzymes and their role in different cancers. By bringing this information together, we hope to highlight new possibilities for developing treatments that specifically target these enzymes, ultimately providing new therapeutic strategies for cancer patients. |
| NatRevCan | 1 | 10.1038/s41568-025-00810-5 | DeepSeek | Phosphoinositide kinases, extending beyond the well-known phosphoinositide 3-kinase (PI3K), are key players in the dynamic and site-specific phosphorylation of lipid phosphoinositides. Unlike PI3Ks, phosphatidylinositol 4-kinases (PI4Ks) and phosphatidylinositol phosphate kinases (PIPKs) do not usually exhibit mutational alterations, but mostly show altered expression in tumours, orchestrating a broad spectrum of signalling, metabolic and immune processes, all of which are crucial in the pathogenesis of cancer. Dysregulation of PI4Ks and PIPKs has been associated with various malignancies, which has sparked considerable interest towards their therapeutic targeting. In this Review we summarize the current understanding of the lesser-studied phosphoinositide kinase families, PI4K and PIPK, focusing on their functions and relevance in cancer. In addition, we provide an overview of ongoing efforts driving the preclinical and clinical development of phosphoinositide kinase-targeting molecules. | Cancer is a complex disease driven by many changes in cell behavior, including how cells process signals and energy. Scientists are studying a group of enzymes called phosphoinositide kinases, which help control these processes. While one type, PI3K, is well-known in cancer, others—like PI4K and PIPK—are less understood but also play important roles. These enzymes are often overactive in tumors, affecting cancer growth, metabolism, and even the immune system. Researchers are exploring how targeting these enzymes with new drugs could lead to better cancer treatments. This review explains what we know about PI4K and PIPK in cancer and highlights promising experimental drugs that may one day help patients. | Cancer is a complex disease driven by many factors, including changes in certain enzymes that control cell growth and survival. While one such enzyme, PI3K, is well-studied in cancer, others—like PI4K and PIPK—are less understood but still play important roles in tumors. These enzymes help regulate signals inside cells, affect metabolism, and influence the immune system, all of which can contribute to cancer growth. Although they aren’t often mutated in cancer, their levels are frequently altered in tumors, making them promising targets for new treatments. This review explains how PI4K and PIPK contribute to cancer and highlights recent progress in developing drugs to block them. Understanding these enzymes could lead to better therapies for patients, offering new hope in the fight against cancer. | Cancer cells rely on certain proteins called phosphoinositide kinases to grow and survive. While one type, PI3K, is well-studied, others like PI4K and PIPK are less understood but also play important roles in cancer. These proteins help control signals inside cells, affect metabolism, and even influence the immune system—all of which can help tumors develop. Although they aren’t often mutated in cancer, their levels are often too high or too low in tumors. Researchers are now exploring how to target these proteins with new drugs to stop cancer growth. This review explains what we know about PI4K and PIPK in cancer and highlights promising treatments being tested in labs and clinics. Understanding these proteins could lead to better ways to fight cancer in the future. |
| NatRevCan | 1 | 10.1038/s41568-025-00810-6 | Grok | Phosphoinositide kinases, extending beyond the well-known phosphoinositide 3-kinase (PI3K), are key players in the dynamic and site-specific phosphorylation of lipid phosphoinositides. Unlike PI3Ks, phosphatidylinositol 4-kinases (PI4Ks) and phosphatidylinositol phosphate kinases (PIPKs) do not usually exhibit mutational alterations, but mostly show altered expression in tumours, orchestrating a broad spectrum of signalling, metabolic and immune processes, all of which are crucial in the pathogenesis of cancer. Dysregulation of PI4Ks and PIPKs has been associated with various malignancies, which has sparked considerable interest towards their therapeutic targeting. In this Review we summarize the current understanding of the lesser-studied phosphoinositide kinase families, PI4K and PIPK, focusing on their functions and relevance in cancer. In addition, we provide an overview of ongoing efforts driving the preclinical and clinical development of phosphoinositide kinase-targeting molecules. | This study explores why certain enzymes, called phosphoinositide kinases, are important in cancer. Unlike some well-known enzymes, these lesser-known ones, named PI4Ks and PIPKs, often change how much they are produced in tumors rather than mutating. They help control processes like cell signaling, metabolism, and immune responses, which can drive cancer growth. The researchers want to understand how these enzymes work in cancer and find ways to target them with new treatments. By studying these enzymes, scientists hope to discover new drugs that could stop cancer from growing or spreading. This work could lead to better treatments for many types of cancer, helping doctors and researchers save more lives in the future. | This research explores special enzymes called phosphoinositide kinases, which help control important processes in our cells but can go wrong in cancer. Unlike some well-known enzymes, these kinases, specifically PI4Ks and PIPKs, often change how much they are made in tumors, affecting cancer growth, metabolism, and immune responses. The study aims to understand how these enzymes work in cancer and find ways to target them with new drugs. By learning more about these kinases, scientists hope to develop better treatments for different types of cancer. This could lead to new medicines that help stop cancer from growing, benefiting researchers and doctors working to improve cancer care. | This study explores special proteins called phosphoinositide kinases, which help control how cells work in the body, especially in cancer. Unlike some similar proteins, these kinases, known as PI4Ks and PIPKs, don’t often have genetic changes but show unusual activity in tumors. They affect how cancer grows by influencing cell signals, metabolism, and immune responses. The researchers aim to understand how these proteins work in cancer and find ways to target them with new medicines. Their findings could help scientists develop better treatments for different cancers, making it easier to stop tumors from growing. This work is important because it opens new paths for creating drugs that could improve cancer treatment and help patients live healthier lives. |
| NatRevCan | 2 | 10.1038/s41568-024-00788-3 | Open AI | Tumours of the appendix - a vestigial digestive organ attached to the colon - are rare. Although we estimate that around 3,000 new appendiceal cancer cases are diagnosed annually in the USA, the challenges of accurately diagnosing and identifying this tumour type suggest that this number may underestimate true population incidence. In the current absence of disease-specific screening and diagnostic imaging modalities, or well-established risk factors, the incidental discovery of appendix tumours is often prompted by acute presentations mimicking appendicitis or when the tumour has already spread into the abdominal cavity - wherein the potential misclassification of appendiceal tumours as malignancies of the colon and ovaries also increases. Notwithstanding these diagnostic difficulties, our understanding of appendix carcinogenesis has advanced in recent years. However, there persist considerable challenges to accelerating the pace of research discoveries towards the path to improved treatments and cures for patients with this group of orphan malignancies. The premise of this Expert Recommendation article is to discuss the current state of the field, to delineate unique challenges for the study of appendiceal tumours, and to propose key priority research areas that will deliver a more complete picture of appendix carcinogenesis and metastasis. The Appendix Cancer Pseudomyxoma Peritonei (ACPMP) Research Foundation Scientific Think Tank delivered a consensus of core research priorities for appendiceal tumours that are poised to be ground-breaking and transformative for scientific discovery and innovation. On the basis of these six research areas, here, we define the first 'cells to society' research framework for appendix tumours. | Appendix cancers are rare tumors that develop in the small, finger-like organ attached to the large intestine. Because these tumors often cause symptoms similar to appendicitis or are found only after spreading in the abdomen, they are hard to detect and sometimes confused with other cancers. Right now, there are no clear screening tests, imaging methods, or known risk factors for this disease. This study brings together expert recommendations to outline the most important research areas for understanding appendix cancers. By looking at the disease from the level of individual cells up to society as a whole, the framework aims to improve how these cancers are studied, diagnosed, and treated. The findings from this approach could guide future research, help doctors recognize appendix tumors earlier, and ultimately lead to better treatments and outcomes for patients with this rare disease. | Appendix tumors are rare cancers that are often discovered by accident, usually when someone shows symptoms similar to appendicitis or when the cancer has already spread. Because these tumors are so uncommon, they are hard to diagnose correctly and can be confused with cancers of the colon or ovaries. This makes it difficult for doctors and scientists to study them and find better treatments. Our work brings together expert recommendations to outline the most important areas for research, from the smallest cells to the broader social impacts of the disease. By defining a “cells to society” framework, we aim to guide future studies, improve understanding of how these cancers develop and spread, and help the research community focus on discoveries that could eventually lead to better diagnosis, treatment, and outcomes for patients with appendix tumors. | Appendix tumors are rare cancers that are often discovered by accident, usually when patients show signs similar to appendicitis or when the cancer has already spread. Because these tumors are hard to identify and can be confused with cancers of the colon or ovaries, researchers face many challenges in studying them. This study reviews what is currently known about how appendix tumors develop and spread and highlights the difficulties in diagnosing and treating them. Experts have identified six key research areas that could lead to major breakthroughs in understanding these cancers. Using these priorities, the study proposes the first “cells to society” framework, which links research from basic cell biology to patient care and public health. This approach aims to guide future studies, improve diagnosis, and ultimately help develop better treatments for people affected by appendix tumors. |
| NatRevCan | 2 | 10.1038/s41568-024-00788-4 | Claude | Tumours of the appendix - a vestigial digestive organ attached to the colon - are rare. Although we estimate that around 3,000 new appendiceal cancer cases are diagnosed annually in the USA, the challenges of accurately diagnosing and identifying this tumour type suggest that this number may underestimate true population incidence. In the current absence of disease-specific screening and diagnostic imaging modalities, or well-established risk factors, the incidental discovery of appendix tumours is often prompted by acute presentations mimicking appendicitis or when the tumour has already spread into the abdominal cavity - wherein the potential misclassification of appendiceal tumours as malignancies of the colon and ovaries also increases. Notwithstanding these diagnostic difficulties, our understanding of appendix carcinogenesis has advanced in recent years. However, there persist considerable challenges to accelerating the pace of research discoveries towards the path to improved treatments and cures for patients with this group of orphan malignancies. The premise of this Expert Recommendation article is to discuss the current state of the field, to delineate unique challenges for the study of appendiceal tumours, and to propose key priority research areas that will deliver a more complete picture of appendix carcinogenesis and metastasis. The Appendix Cancer Pseudomyxoma Peritonei (ACPMP) Research Foundation Scientific Think Tank delivered a consensus of core research priorities for appendiceal tumours that are poised to be ground-breaking and transformative for scientific discovery and innovation. On the basis of these six research areas, here, we define the first 'cells to society' research framework for appendix tumours. | Appendix cancer is a rare disease that affects about 3,000 people each year in the United States. The appendix is a small organ connected to the large intestine that doctors once thought was not needed. These cancers are hard to find early because there are no special tests to screen for them and doctors do not know what causes them. Often, patients only learn they have appendix cancer when they feel sick with stomach pain that seems like a regular appendix infection, or when the cancer has already spread to other parts of the body. This makes treatment harder. A group of cancer experts came together to create a plan for better research on appendix tumors. They want to study everything from how cancer cells grow to how this disease affects patients and families. Their goal is to find new ways to detect, treat, and cure appendix cancer sooner, which could save more lives. | Appendix cancer is a rare disease that affects about 3,000 Americans each year, though the actual number may be higher since these tumors are hard to diagnose correctly. The appendix is a small organ connected to the large intestine that doctors once thought was useless. These cancers are often found by accident when patients think they have appendicitis or when the cancer has already spread throughout the belly. Doctors sometimes mistake appendix cancer for colon or ovary cancer, making treatment harder. While scientists have learned more about how appendix cancer develops in recent years, research moves slowly because the disease is so uncommon. A group of appendix cancer experts came together to identify the most important research questions that need answers. They created a new research plan called "cells to society" that will help scientists study everything from how cancer cells behave to how the disease affects patients and families, ultimately leading to better treatments.RetryClaude can make mistakes. Please double-check responses. | Appendix cancer is a rare disease that affects the small organ attached to the large intestine. About 3,000 people get this cancer each year in America, but doctors think more cases exist because it is hard to find and diagnose. Most people only learn they have appendix cancer when they feel sick with belly pain or when the cancer has already spread. This happens because there are no special tests to check for this cancer early, and doctors often mistake it for other diseases. A group of cancer experts worked together to create a plan for studying appendix cancer better. They want to understand how this cancer starts and spreads so they can find better treatments. The experts identified six key areas that need more research. This new plan, called a "cells to society" framework, will help scientists work together to solve the biggest problems in appendix cancer research and help patients get better care. |
| NatRevCan | 2 | 10.1038/s41568-024-00788-5 | Gemini | Tumours of the appendix - a vestigial digestive organ attached to the colon - are rare. Although we estimate that around 3,000 new appendiceal cancer cases are diagnosed annually in the USA, the challenges of accurately diagnosing and identifying this tumour type suggest that this number may underestimate true population incidence. In the current absence of disease-specific screening and diagnostic imaging modalities, or well-established risk factors, the incidental discovery of appendix tumours is often prompted by acute presentations mimicking appendicitis or when the tumour has already spread into the abdominal cavity - wherein the potential misclassification of appendiceal tumours as malignancies of the colon and ovaries also increases. Notwithstanding these diagnostic difficulties, our understanding of appendix carcinogenesis has advanced in recent years. However, there persist considerable challenges to accelerating the pace of research discoveries towards the path to improved treatments and cures for patients with this group of orphan malignancies. The premise of this Expert Recommendation article is to discuss the current state of the field, to delineate unique challenges for the study of appendiceal tumours, and to propose key priority research areas that will deliver a more complete picture of appendix carcinogenesis and metastasis. The Appendix Cancer Pseudomyxoma Peritonei (ACPMP) Research Foundation Scientific Think Tank delivered a consensus of core research priorities for appendiceal tumours that are poised to be ground-breaking and transformative for scientific discovery and innovation. On the basis of these six research areas, here, we define the first 'cells to society' research framework for appendix tumours. | Appendiceal tumours, which are rare cancers of a small digestive organ, are often difficult to diagnose because they are typically discovered by chance or after they have already spread. To help improve patient outcomes, this study brings together experts to create a clear plan for future research. This plan, called a 'cells to society' framework, identifies six key areas of research, from understanding the basic biology of the cancer cells to finding better ways to diagnose and treat patients in the wider community. The aim is to accelerate scientific discovery and move closer to finding new and effective treatments for this challenging disease. | This paper lays out a new plan to study rare tumors that grow in the appendix. Currently, these tumors are hard to find early because there are no special tests for them, and they are often mistaken for other cancers, like those of the colon or ovaries. This can mean they are only discovered after they have already spread. To speed up the development of better treatments, a group of experts has identified six key areas of research. This research framework, called "cells to society," outlines how to study these tumors from their very beginnings, at the cellular level, all the way to how they affect people and society. The goal is to improve our understanding of how these tumors start and spread, ultimately leading to better care and cures for patients. | Tumors of the appendix are rare and challenging to diagnose. Because they are often mistaken for more common cancers, such as those in the colon or ovaries, there is a lack of specific screening and treatment options. This research aims to address these challenges by outlining a new framework for studying appendiceal tumors. This framework, called the 'cells to society' model, identifies six key research areas. By focusing on these priorities, scientists can better understand how these tumors develop and spread. The findings from this research are expected to accelerate the discovery of improved treatments and cures, ultimately benefiting patients with this type of cancer. |
| NatRevCan | 2 | 10.1038/s41568-024-00788-6 | DeepSeek | Tumours of the appendix - a vestigial digestive organ attached to the colon - are rare. Although we estimate that around 3,000 new appendiceal cancer cases are diagnosed annually in the USA, the challenges of accurately diagnosing and identifying this tumour type suggest that this number may underestimate true population incidence. In the current absence of disease-specific screening and diagnostic imaging modalities, or well-established risk factors, the incidental discovery of appendix tumours is often prompted by acute presentations mimicking appendicitis or when the tumour has already spread into the abdominal cavity - wherein the potential misclassification of appendiceal tumours as malignancies of the colon and ovaries also increases. Notwithstanding these diagnostic difficulties, our understanding of appendix carcinogenesis has advanced in recent years. However, there persist considerable challenges to accelerating the pace of research discoveries towards the path to improved treatments and cures for patients with this group of orphan malignancies. The premise of this Expert Recommendation article is to discuss the current state of the field, to delineate unique challenges for the study of appendiceal tumours, and to propose key priority research areas that will deliver a more complete picture of appendix carcinogenesis and metastasis. The Appendix Cancer Pseudomyxoma Peritonei (ACPMP) Research Foundation Scientific Think Tank delivered a consensus of core research priorities for appendiceal tumours that are poised to be ground-breaking and transformative for scientific discovery and innovation. On the basis of these six research areas, here, we define the first 'cells to society' research framework for appendix tumours. | Appendix tumors are rare and hard to diagnose, often found by accident when patients have symptoms like appendicitis or after the cancer has spread. Because these tumors are sometimes mistaken for colon or ovarian cancer, the true number of cases may be higher than reported. There are no special tests to catch them early, and little is known about what causes them. This study brings together experts to outline the biggest challenges in appendix cancer research and suggest key areas for future studies. By focusing on six priority topics, the researchers created a new "cells to society" framework—a roadmap to better understand these tumors, improve diagnosis, and find better treatments. Their work could help speed up discoveries and give hope to patients with this rare disease. | This study focuses on appendix tumors, which are rare and often hard to diagnose. Many cases are found by accident when patients have symptoms like appendicitis or after the cancer has spread. Because these tumors can be mistaken for colon or ovarian cancers, getting the right diagnosis is difficult. Researchers want to improve our understanding of how these tumors form and spread so they can find better treatments. A group of experts identified six key research areas to speed up discoveries. This paper introduces the first “cells to society” research plan, which looks at everything from tiny cell changes to real-world patient care. The goal is to give doctors better tools to diagnose and treat appendix cancer, helping patients live longer, healthier lives. | Appendiceal tumors—rare cancers in the small organ attached to the colon—are often found by accident, usually when patients have symptoms like appendicitis or after the cancer has spread. Because these tumors are hard to diagnose and may be mistaken for colon or ovarian cancer, the true number of cases is likely higher than reported. While research has improved our understanding of these cancers, progress in finding better treatments has been slow. This study brings together experts to identify key research priorities, aiming to speed up discoveries that could help patients. By focusing on six major areas—from studying cancer cells to improving patient care—the team created the first “cells to society” research plan for appendiceal tumors. This roadmap could lead to better diagnosis, treatment, and outcomes for people with this rare disease. |
| NatRevCan | 2 | 10.1038/s41568-024-00788-7 | Grok | Tumours of the appendix - a vestigial digestive organ attached to the colon - are rare. Although we estimate that around 3,000 new appendiceal cancer cases are diagnosed annually in the USA, the challenges of accurately diagnosing and identifying this tumour type suggest that this number may underestimate true population incidence. In the current absence of disease-specific screening and diagnostic imaging modalities, or well-established risk factors, the incidental discovery of appendix tumours is often prompted by acute presentations mimicking appendicitis or when the tumour has already spread into the abdominal cavity - wherein the potential misclassification of appendiceal tumours as malignancies of the colon and ovaries also increases. Notwithstanding these diagnostic difficulties, our understanding of appendix carcinogenesis has advanced in recent years. However, there persist considerable challenges to accelerating the pace of research discoveries towards the path to improved treatments and cures for patients with this group of orphan malignancies. The premise of this Expert Recommendation article is to discuss the current state of the field, to delineate unique challenges for the study of appendiceal tumours, and to propose key priority research areas that will deliver a more complete picture of appendix carcinogenesis and metastasis. The Appendix Cancer Pseudomyxoma Peritonei (ACPMP) Research Foundation Scientific Think Tank delivered a consensus of core research priorities for appendiceal tumours that are poised to be ground-breaking and transformative for scientific discovery and innovation. On the basis of these six research areas, here, we define the first 'cells to society' research framework for appendix tumours. | This research focuses on rare appendix tumors, which are hard to find and often mistaken for other cancers, like those of the colon or ovaries. About 3,000 new cases are found each year in the USA, but the real number might be higher. Doctors usually discover these tumors by chance during surgeries for appendicitis or when the cancer has spread. The study aims to create a new plan called "cells to society" to better understand how these tumors start and spread. By setting six key research goals, the authors hope to improve diagnosis and find better treatments. This work could help scientists learn more about these rare cancers and lead to new ways to help patients, making a big difference in the research community. | Appendiceal tumors, found in the appendix, a small organ near the colon, are rare and hard to spot early. About 3,000 new cases happen yearly in the USA, but the real number might be higher because these tumors are often mistaken for other cancers or found by chance during appendicitis-like emergencies. This research aims to better understand these tumors by creating a new plan called "cells to society." It focuses on improving how we find, study, and treat these cancers. By identifying six key research areas, scientists hope to make discoveries that lead to better treatments and cures. This work could help doctors diagnose these tumors sooner and improve lives by advancing science and care for people with this rare disease. | Appendiceal tumors, which grow in a small part of the digestive system called the appendix, are rare and hard to find because there are no specific tests to detect them early. Doctors often discover these tumors by chance during emergencies like appendicitis or when the cancer has already spread. This research aims to better understand these tumors and find new ways to diagnose and treat them. By creating a plan that looks at everything from the tiny cells in the tumors to how they affect people and society, the study hopes to speed up discoveries. These findings could lead to better treatments and possibly cures, helping doctors and scientists learn more about this rare cancer and improve care for patients. |
| NatRevCan | 3 | 10.1038/s41568-025-00841-9 | Open AI | In the ongoing battle against cancer, the natural world provides promising inspiration for designing novel therapeutic strategies. The field of comparative oncology offers a valuable source of such inspiration. By combining evolutionary biology, ecology, veterinary medicine and clinical oncology, comparative oncology aims to better understand cancer, especially by highlighting taxa that are strongly resistant or susceptible to cancer and to identify the molecular and cellular mechanisms underlying the remarkable cancer resistance of some taxa. Such studies hold profound implications for human cancer research and treatment, and increase the probability of detecting therapeutic avenues that are non-toxic to healthy cells and tissues. This Perspective underscores the importance of comparative oncology, emphasizes its relevance, and showcases recent breakthroughs in identifying natural cancer resistance mechanisms and opportunities for clinical translation. We advocate for a better integration of cancer research on non-conventional model species into oncology and we urge enhanced cooperation between clinicians and comparative oncologists to advance cancer prevention or treatment strategies. | This study explores how learning from animals can help fight cancer in humans. Some animals are naturally very good at resisting cancer, while others are more prone to it. By studying these differences, scientists can uncover how cells and molecules work to prevent or allow cancer to grow. The research combines knowledge from evolution, ecology, animal health, and human medicine to understand cancer better and find new treatments. Discovering natural ways to stop cancer could lead to safer therapies that target cancer cells without harming healthy ones. This approach encourages collaboration between doctors and scientists studying animals and humans, aiming to speed up the development of better cancer prevention and treatment strategies for people. | This study explores how learning from animals and nature can help fight cancer in humans. Some species naturally resist cancer, while others are more vulnerable. By studying these differences, scientists can uncover how cancer develops and how it can be stopped without harming healthy cells. This approach combines knowledge from animal biology, ecology, and medical research to find safer, more effective ways to prevent and treat cancer. The research highlights recent discoveries about natural cancer resistance and shows how these insights could be used in human medicine. By working closely with doctors and studying a wider variety of species, researchers hope to open new paths for cancer prevention and therapy. This work could lead to treatments that are more precise, less toxic, and better tailored to human health. | Cancer is a complex disease that affects many animals, including humans. Studying how different species naturally resist or develop cancer can teach us new ways to prevent and treat the disease. This research approach, called comparative oncology, looks at animals that are unusually resistant to cancer and explores the biological mechanisms that protect them. By combining knowledge from evolution, ecology, veterinary medicine, and human cancer studies, scientists hope to uncover safer, more effective therapies that target cancer without harming healthy cells. Recent discoveries in this field have identified natural strategies that some animals use to fight cancer, offering promising ideas for human medicine. Encouraging collaboration between doctors and researchers who study animals can help turn these insights into real treatments, ultimately improving cancer prevention and care for people. |
| NatRevCan | 3 | 10.1038/s41568-025-00841-10 | Claude | In the ongoing battle against cancer, the natural world provides promising inspiration for designing novel therapeutic strategies. The field of comparative oncology offers a valuable source of such inspiration. By combining evolutionary biology, ecology, veterinary medicine and clinical oncology, comparative oncology aims to better understand cancer, especially by highlighting taxa that are strongly resistant or susceptible to cancer and to identify the molecular and cellular mechanisms underlying the remarkable cancer resistance of some taxa. Such studies hold profound implications for human cancer research and treatment, and increase the probability of detecting therapeutic avenues that are non-toxic to healthy cells and tissues. This Perspective underscores the importance of comparative oncology, emphasizes its relevance, and showcases recent breakthroughs in identifying natural cancer resistance mechanisms and opportunities for clinical translation. We advocate for a better integration of cancer research on non-conventional model species into oncology and we urge enhanced cooperation between clinicians and comparative oncologists to advance cancer prevention or treatment strategies. | Scientists are studying animals in nature to find new ways to fight cancer in humans. Some animals almost never get cancer, while others get it often. By looking at different animals and how their bodies work, researchers want to learn what makes some animals so good at fighting off cancer. This research brings together experts who study evolution, animal health, and human cancer treatment. The goal is to find natural ways that animals protect themselves from cancer and use these discoveries to create better, safer treatments for people. These animal-based treatments could work well without harming healthy parts of the human body. This research is important because it opens up new paths for cancer treatment that we might not find by only studying humans. The authors believe doctors and animal researchers should work together more closely to develop better ways to prevent and treat cancer using nature's own solutions. | Cancer kills millions of people each year, but some animals almost never get cancer. Scientists are now studying these cancer-resistant animals to find new ways to fight human cancer. This research field is called comparative oncology. It looks at different animals to understand why some get cancer easily while others stay healthy. By studying elephants, sharks, and other animals that rarely develop tumors, researchers hope to find natural ways to prevent or treat cancer in humans. These animal studies could lead to safer cancer treatments that do not harm healthy body parts like current treatments often do. The authors believe doctors and animal researchers should work together more closely. This teamwork could speed up the discovery of better cancer treatments. Learning from nature's own cancer-fighting methods may give us powerful new tools to save human lives and reduce cancer suffering worldwide. | Cancer kills millions of people each year, making it one of our biggest health challenges. Scientists are now looking to nature for new ways to fight this disease. Some animals, like elephants and naked mole rats, almost never get cancer, while others get it often. By studying these animals, researchers can learn what makes some creatures so good at fighting off cancer. This approach, called comparative oncology, brings together scientists who study evolution, animal doctors, and cancer doctors to work as a team. They want to find the special tricks that cancer-resistant animals use to stay healthy. These discoveries could lead to new treatments for humans that kill cancer cells but leave healthy cells alone. This research shows why we need animal experts and human cancer doctors to work together more closely. By learning from nature's best cancer fighters, we might find better ways to prevent and treat cancer in people. |
| NatRevCan | 3 | 10.1038/s41568-025-00841-11 | Gemini | In the ongoing battle against cancer, the natural world provides promising inspiration for designing novel therapeutic strategies. The field of comparative oncology offers a valuable source of such inspiration. By combining evolutionary biology, ecology, veterinary medicine and clinical oncology, comparative oncology aims to better understand cancer, especially by highlighting taxa that are strongly resistant or susceptible to cancer and to identify the molecular and cellular mechanisms underlying the remarkable cancer resistance of some taxa. Such studies hold profound implications for human cancer research and treatment, and increase the probability of detecting therapeutic avenues that are non-toxic to healthy cells and tissues. This Perspective underscores the importance of comparative oncology, emphasizes its relevance, and showcases recent breakthroughs in identifying natural cancer resistance mechanisms and opportunities for clinical translation. We advocate for a better integration of cancer research on non-conventional model species into oncology and we urge enhanced cooperation between clinicians and comparative oncologists to advance cancer prevention or treatment strategies. | This research explores how studying cancer in animals can help us fight cancer in people. Some animals are very good at resisting cancer, and by understanding why, we can find new ways to prevent and treat the disease. Our goal is to identify the unique ways these animals stay healthy and use that knowledge to create new cancer medicines that are less harmful to a person’s body. This approach, called comparative oncology, brings together scientists who study animals and doctors who treat people, opening up promising new directions for cancer research and potentially leading to more effective and safer treatments for everyone. | To advance cancer research, scientists are studying animals that are naturally resistant to cancer. This field, called comparative oncology, looks at why some species rarely get cancer, even when they live long lives. By combining knowledge from different fields, researchers hope to uncover the special ways these animals fight off the disease. The goal is to find new ideas for cancer treatments in humans that are less harmful to healthy cells. This approach could lead to new ways to prevent or treat cancer by understanding the natural defenses found in the animal kingdom. | By studying animals that rarely get cancer, researchers can gain new insights into the disease. This field, called comparative oncology, looks at a variety of species to understand why some are so resistant to cancer. The goal of this research is to discover the natural defenses these animals have against cancer and to find new ways to treat cancer in humans. For example, understanding how a specific animal's cells prevent tumors from forming could lead to developing new, more effective drugs that don't harm healthy cells. These findings may also help us develop better ways to prevent and treat cancer, ultimately improving patient outcomes. |
| NatRevCan | 3 | 10.1038/s41568-025-00841-12 | DeepSeek | In the ongoing battle against cancer, the natural world provides promising inspiration for designing novel therapeutic strategies. The field of comparative oncology offers a valuable source of such inspiration. By combining evolutionary biology, ecology, veterinary medicine and clinical oncology, comparative oncology aims to better understand cancer, especially by highlighting taxa that are strongly resistant or susceptible to cancer and to identify the molecular and cellular mechanisms underlying the remarkable cancer resistance of some taxa. Such studies hold profound implications for human cancer research and treatment, and increase the probability of detecting therapeutic avenues that are non-toxic to healthy cells and tissues. This Perspective underscores the importance of comparative oncology, emphasizes its relevance, and showcases recent breakthroughs in identifying natural cancer resistance mechanisms and opportunities for clinical translation. We advocate for a better integration of cancer research on non-conventional model species into oncology and we urge enhanced cooperation between clinicians and comparative oncologists to advance cancer prevention or treatment strategies. | Cancer affects both humans and animals, but some species rarely get cancer or fight it very well. Scientists study these animals—like elephants, naked mole rats, and certain whales—to learn how their bodies naturally resist cancer. By comparing different species, researchers hope to find new, safer ways to treat cancer in humans without harming healthy cells. This field, called comparative oncology, brings together wildlife experts, veterinarians, and doctors to share discoveries. Recent findings could lead to better treatments or even prevention strategies. Working together, scientists and doctors might unlock nature’s secrets to beating cancer, helping patients in the future. | Cancer affects both humans and animals, but some species have surprising natural resistance to the disease. By studying these animals—along with those highly prone to cancer—scientists hope to uncover new ways to fight tumors without harming healthy cells. This research combines wildlife biology, veterinary medicine, and human cancer studies to find safer, more effective treatments. Recent discoveries in species like elephants (which rarely get cancer) have already inspired potential therapies. The goal is to bridge the gap between animal research and human medicine, encouraging doctors and scientists to work together. If successful, these findings could lead to breakthrough treatments that are gentler and more powerful than current options. | Cancer affects both humans and animals, but some species—like elephants and naked mole-rats—rarely get it. Studying these animals helps scientists uncover natural ways to fight cancer. By comparing how different creatures resist or develop cancer, researchers can discover new, safer treatments that don’t harm healthy cells. This field, called comparative oncology, combines wildlife biology, veterinary medicine, and human cancer research to find better therapies. Recent breakthroughs show promise for turning these natural defenses into real treatments. The study calls for stronger teamwork between doctors and animal researchers to speed up progress in preventing and treating cancer. Learning from nature could be the key to beating this disease. |
| NatRevCan | 3 | 10.1038/s41568-025-00841-13 | Grok | In the ongoing battle against cancer, the natural world provides promising inspiration for designing novel therapeutic strategies. The field of comparative oncology offers a valuable source of such inspiration. By combining evolutionary biology, ecology, veterinary medicine and clinical oncology, comparative oncology aims to better understand cancer, especially by highlighting taxa that are strongly resistant or susceptible to cancer and to identify the molecular and cellular mechanisms underlying the remarkable cancer resistance of some taxa. Such studies hold profound implications for human cancer research and treatment, and increase the probability of detecting therapeutic avenues that are non-toxic to healthy cells and tissues. This Perspective underscores the importance of comparative oncology, emphasizes its relevance, and showcases recent breakthroughs in identifying natural cancer resistance mechanisms and opportunities for clinical translation. We advocate for a better integration of cancer research on non-conventional model species into oncology and we urge enhanced cooperation between clinicians and comparative oncologists to advance cancer prevention or treatment strategies. | Scientists are studying cancer in animals to help find new ways to fight cancer in people. By looking at animals that rarely get cancer or get it often, researchers hope to learn what makes some animals resistant to cancer. They want to use these discoveries to create treatments that attack cancer without harming healthy parts of the body. This research, called comparative oncology, combines knowledge from animal doctors, cancer experts, and biologists to improve cancer prevention and treatment. The findings could lead to safer, more effective therapies for humans and spark new ideas for cancer research worldwide. | This study explores how learning from animals can help fight cancer in people. Researchers are studying why some animals rarely get cancer while others get it easily, looking at their biology and environments. By comparing these animals, they hope to find new ways to prevent or treat cancer that are safe for healthy cells. This work is exciting because it could lead to better, less harmful cancer treatments for humans. It also encourages scientists and doctors to work together, using discoveries from animals to improve cancer care. This research could change how we understand and battle cancer, making a big difference in the medical world. | This study suggests using comparative oncology to improve cancer research by learning from animals that rarely get cancer or are more likely to develop it. Researchers want to understand why some animals resist cancer and use those discoveries to create new, safer treatments for humans. By studying different species, they aim to find unique ways to fight cancer that don’t harm healthy cells. This work could lead to better cancer prevention and treatments, helping both people and animals. The findings may inspire scientists to explore new ideas and encourage doctors and animal experts to work together, speeding up progress in cancer care. |
| NatRevCan | 4 | 10.1038/s41568-025-00831-w | Open AI | The nervous and immune systems have co-evolved to detect and respond to internal and external threats, working together to restore homeostasis after tissue injury or infection. Sharing several receptors and ligands, they engage in direct cross-talk that substantially influences disease development. The emerging field of cancer neuro-immunity focuses on the intricate interactions between the nervous system, immune responses and tumour growth. Additional findings have revealed that nerve fibres infiltrating peripheral tumours can release neuromodulatory factors that shape both immune cell behaviour and tumour progression. Conversely, tumour-infiltrating immune cells can modify the activity of local neurons, including pain-transmitting nociceptive sensory neurons. Beyond sensory fibres, sympathetic signalling can foster immunosuppression by recruiting myeloid-derived suppressor cells and promoting T cell exhaustion. This Review summarizes current evidence on how neuronal signalling regulates peripheral antitumour immune responses within the tumour microenvironment. We describe the complex, reciprocal interactions among neurons, immune cells and malignant cells, highlighting the key parts played by the peripheral nervous system in modulating immunity against cancer. By understanding this neuro-immune axis, novel therapeutic approaches may be uncovered to strengthen antitumour immunity and enhance responses to existing cancer treatments. | Our bodies’ nervous and immune systems work closely together to detect threats and keep us healthy. Recent research shows that this partnership also affects how cancer grows. Nerves can enter tumors and release signals that change how immune cells behave, sometimes helping the tumor survive. At the same time, immune cells inside tumors can influence nerve activity, including the nerves that sense pain. Certain nerve signals can weaken the immune system, making it harder for the body to fight cancer. This study reviews how nerves and immune cells communicate within tumors and how this communication affects cancer progression. By understanding these interactions, scientists hope to develop new treatments that boost the immune system’s ability to fight cancer and improve responses to current therapies. | Our bodies’ nervous and immune systems work closely together to detect threats and heal injuries. In cancer, this teamwork becomes more complicated. Nerves that grow into tumors can release signals that change how immune cells act, sometimes helping the tumor grow. At the same time, immune cells can affect nearby nerves, including those that sense pain. Certain nerve signals may even weaken the immune system’s ability to fight cancer. This study reviews how these interactions happen and how the nervous system can influence immune responses against tumors. By understanding this “neuro-immune conversation,” researchers hope to find new ways to boost the immune system and improve cancer treatments. These insights could lead to therapies that make the body’s natural defenses stronger, offering better outcomes for people with cancer. | Our bodies have two important defense systems: the nervous system, which senses changes and sends signals, and the immune system, which fights infections and abnormal cells. These systems work closely together, and their communication can affect how cancer grows. Recent research shows that nerve fibers can enter tumors and release signals that change immune cell behavior, while immune cells in tumors can influence nerve activity, including signals related to pain. Some nerve signals can weaken the immune response, making it harder for the body to fight cancer. By studying how nerves and immune cells interact in tumors, scientists hope to uncover new ways to boost the immune system and improve cancer treatments. Understanding this communication could lead to therapies that make existing treatments more effective and offer new strategies to slow or stop cancer growth. |
| NatRevCan | 4 | 10.1038/s41568-025-00831-w | Claude | The nervous and immune systems have co-evolved to detect and respond to internal and external threats, working together to restore homeostasis after tissue injury or infection. Sharing several receptors and ligands, they engage in direct cross-talk that substantially influences disease development. The emerging field of cancer neuro-immunity focuses on the intricate interactions between the nervous system, immune responses and tumour growth. Additional findings have revealed that nerve fibres infiltrating peripheral tumours can release neuromodulatory factors that shape both immune cell behaviour and tumour progression. Conversely, tumour-infiltrating immune cells can modify the activity of local neurons, including pain-transmitting nociceptive sensory neurons. Beyond sensory fibres, sympathetic signalling can foster immunosuppression by recruiting myeloid-derived suppressor cells and promoting T cell exhaustion. This Review summarizes current evidence on how neuronal signalling regulates peripheral antitumour immune responses within the tumour microenvironment. We describe the complex, reciprocal interactions among neurons, immune cells and malignant cells, highlighting the key parts played by the peripheral nervous system in modulating immunity against cancer. By understanding this neuro-immune axis, novel therapeutic approaches may be uncovered to strengthen antitumour immunity and enhance responses to existing cancer treatments. | Cancer cells don't grow alone in the body. They exist in a complex neighborhood that includes nerve cells and immune cells, all talking to each other through chemical signals. Scientists are studying how these three types of cells work together because this affects how cancer grows and spreads. Nerve fibers that grow into tumors can release chemicals that change how immune cells fight cancer. At the same time, immune cells near tumors can affect nearby nerves, including those that sense pain. Some nerve signals can actually help cancer by weakening the immune system's ability to fight. This research is important because understanding how nerves and immune cells communicate could lead to new cancer treatments. By targeting these communication pathways, doctors might find better ways to boost the body's natural cancer-fighting abilities and make current treatments work better. This could help millions of cancer patients worldwide get more effective care. | Cancer research has discovered that our nervous system and immune system work together in ways that affect how tumors grow and spread. Scientists are studying how nerve fibers that grow into tumors can release chemicals that change how immune cells behave and how cancer develops. At the same time, immune cells fighting the cancer can also affect nearby nerves, including those that send pain signals. Some nerve signals can actually help cancer by weakening the immune response and making immune cells less effective at fighting tumors. This research examines how nerve signals control immune responses against cancer within the tumor environment. The study explores the complex relationships between nerves, immune cells, and cancer cells, focusing on how the peripheral nervous system influences the body's ability to fight cancer. Understanding these connections between the nervous and immune systems could lead to new cancer treatments that boost the immune response and make current therapies work better against tumors. | Our body's nervous system and immune system work as a team to fight diseases and heal injuries. Scientists are now studying how these two systems talk to each other when fighting cancer. This research shows that nerves growing into tumors can release chemicals that change how immune cells work and how tumors grow. At the same time, immune cells in tumors can affect nearby nerves, including those that send pain signals. Some nerve signals can actually help tumors by weakening the immune response and making immune cells tired. This study reviews how nerve signals control the body's immune fight against cancer in the area around tumors. The authors explain the complex ways that nerves, immune cells, and cancer cells interact with each other. Understanding how nerves and immune cells work together could help doctors create new treatments that make the immune system stronger at fighting cancer and improve current cancer treatments. |
| NatRevCan | 4 | 10.1038/s41568-025-00831-w | Gemini | The nervous and immune systems have co-evolved to detect and respond to internal and external threats, working together to restore homeostasis after tissue injury or infection. Sharing several receptors and ligands, they engage in direct cross-talk that substantially influences disease development. The emerging field of cancer neuro-immunity focuses on the intricate interactions between the nervous system, immune responses and tumour growth. Additional findings have revealed that nerve fibres infiltrating peripheral tumours can release neuromodulatory factors that shape both immune cell behaviour and tumour progression. Conversely, tumour-infiltrating immune cells can modify the activity of local neurons, including pain-transmitting nociceptive sensory neurons. Beyond sensory fibres, sympathetic signalling can foster immunosuppression by recruiting myeloid-derived suppressor cells and promoting T cell exhaustion. This Review summarizes current evidence on how neuronal signalling regulates peripheral antitumour immune responses within the tumour microenvironment. We describe the complex, reciprocal interactions among neurons, immune cells and malignant cells, highlighting the key parts played by the peripheral nervous system in modulating immunity against cancer. By understanding this neuro-immune axis, novel therapeutic approaches may be uncovered to strengthen antitumour immunity and enhance responses to existing cancer treatments. | Our bodies have a constant conversation between our nervous and immune systems, which work together to keep us healthy. This research looks at how this communication, called neuro-immune cross-talk, plays a major role in cancer. Scientists have found that nerves in tumors can release signals that affect how immune cells behave and how the tumor grows. At the same time, immune cells can also change the activity of nearby nerves, including those that sense pain. The goal of this review is to explain these complex interactions between nerves, immune cells, and cancer cells. By better understanding how our nervous system influences the body's fight against cancer, this research could lead to new ways to boost our immune response and improve the effectiveness of current cancer treatments. | Our bodies have a complex network that includes the nervous and immune systems, which work together to keep us healthy. In cancer, these systems communicate in ways that can actually help tumors grow. This study explores the fascinating connection between nerves, immune cells, and cancer. We looked at how nerves that grow into tumors release signals that can weaken the body’s immune attack. At the same time, immune cells within the tumor can influence how these nerves behave, including those that sense pain. We also found that specific nerve signals can promote an environment that suppresses the immune system, making it easier for the cancer to thrive. By understanding this communication between nerves and the immune system in cancer, we hope to discover new ways to improve existing cancer treatments and develop more effective therapies. | In the past, we thought of the nervous and immune systems as separate. However, they actually work together to protect our bodies from harm and keep us healthy. A new field of study is now looking at how these two systems interact specifically within cancer. This research aims to understand how nerves and immune cells communicate in and around a tumor. Scientists have found that nerves can release signals that affect how immune cells behave and how a tumor grows. In turn, immune cells can also influence nearby nerves. This study summarizes these complex two-way interactions, showing the important role that nerves play in controlling the body's immune response to cancer. By understanding this relationship, we hope to find new ways to boost the immune system and create better cancer treatments. |
| NatRevCan | 4 | 10.1038/s41568-025-00831-w | DeepSeek | The nervous and immune systems have co-evolved to detect and respond to internal and external threats, working together to restore homeostasis after tissue injury or infection. Sharing several receptors and ligands, they engage in direct cross-talk that substantially influences disease development. The emerging field of cancer neuro-immunity focuses on the intricate interactions between the nervous system, immune responses and tumour growth. Additional findings have revealed that nerve fibres infiltrating peripheral tumours can release neuromodulatory factors that shape both immune cell behaviour and tumour progression. Conversely, tumour-infiltrating immune cells can modify the activity of local neurons, including pain-transmitting nociceptive sensory neurons. Beyond sensory fibres, sympathetic signalling can foster immunosuppression by recruiting myeloid-derived suppressor cells and promoting T cell exhaustion. This Review summarizes current evidence on how neuronal signalling regulates peripheral antitumour immune responses within the tumour microenvironment. We describe the complex, reciprocal interactions among neurons, immune cells and malignant cells, highlighting the key parts played by the peripheral nervous system in modulating immunity against cancer. By understanding this neuro-immune axis, novel therapeutic approaches may be uncovered to strengthen antitumour immunity and enhance responses to existing cancer treatments. | Cancer doesn’t just affect the body’s cells—it also involves communication between nerves and the immune system. Nerves inside tumors can release signals that change how immune cells work, sometimes helping the cancer grow. At the same time, immune cells can influence nerve activity, even affecting pain signals. This two-way talk between nerves, immune cells, and cancer cells plays a big role in tumor growth and how the body fights the disease. By studying these interactions, scientists hope to find new ways to boost the immune system’s ability to attack cancer and improve existing treatments. This research could lead to better therapies that target both the nervous and immune systems to stop cancer more effectively. | Cancer doesn’t just grow on its own—it interacts with nerves and immune cells in surprising ways. This research explores how the nervous system and immune system "talk" to each other inside tumors, affecting cancer’s growth. Nerves release signals that can weaken the immune system’s ability to fight cancer, while immune cells can also change nerve activity, even influencing pain. For example, stress-related nerves might help tumors hide from immune attacks. By understanding these connections, scientists hope to develop new treatments that block harmful nerve signals or boost the immune system’s power against cancer. This could make existing therapies work better and improve outcomes for patients. | Cancer doesn’t just affect the body’s cells—it also involves nerves and the immune system working together in surprising ways. Nerves inside tumors can release signals that change how immune cells behave, sometimes helping cancer grow. At the same time, immune cells can influence nerve activity, even affecting pain signals. For example, stress-related nerves can weaken the immune system’s fight against cancer. This research explores how nerves and immune cells communicate in tumors, revealing new ways they might be targeted to improve cancer treatments. By understanding these connections, scientists hope to develop therapies that boost the immune system’s ability to attack cancer while making existing treatments more effective. |
| NatRevCan | 4 | 10.1038/s41568-025-00831-w | Grok | The nervous and immune systems have co-evolved to detect and respond to internal and external threats, working together to restore homeostasis after tissue injury or infection. Sharing several receptors and ligands, they engage in direct cross-talk that substantially influences disease development. The emerging field of cancer neuro-immunity focuses on the intricate interactions between the nervous system, immune responses and tumour growth. Additional findings have revealed that nerve fibres infiltrating peripheral tumours can release neuromodulatory factors that shape both immune cell behaviour and tumour progression. Conversely, tumour-infiltrating immune cells can modify the activity of local neurons, including pain-transmitting nociceptive sensory neurons. Beyond sensory fibres, sympathetic signalling can foster immunosuppression by recruiting myeloid-derived suppressor cells and promoting T cell exhaustion. This Review summarizes current evidence on how neuronal signalling regulates peripheral antitumour immune responses within the tumour microenvironment. We describe the complex, reciprocal interactions among neurons, immune cells and malignant cells, highlighting the key parts played by the peripheral nervous system in modulating immunity against cancer. By understanding this neuro-immune axis, novel therapeutic approaches may be uncovered to strengthen antitumour immunity and enhance responses to existing cancer treatments. | This study explores how the nervous system and immune system work together in cancer. Researchers want to understand how nerves and immune cells in tumors talk to each other and affect cancer growth. They found that nerves release chemicals that can change how immune cells act, sometimes helping tumors grow. At the same time, immune cells can influence nerve activity, including nerves that cause pain. The study also shows that stress-related nerve signals can weaken the immune system’s fight against cancer. By learning more about these connections, scientists hope to find new ways to boost the body’s ability to fight cancer and improve cancer treatments. These findings could lead to better therapies that help the immune system attack tumors more effectively, benefiting cancer research and patient care. | This research explores how the nervous system and immune system work together in the body to fight cancer. Scientists want to understand how nerves and immune cells talk to each other in areas where cancer grows, as this can affect how the body battles tumors. The study looks at how nerve signals can change the way immune cells act and how tumors grow, while immune cells can also influence nerves, including those that carry pain signals. By learning more about these connections, researchers hope to find new ways to boost the body’s ability to fight cancer and make cancer treatments work better. This could lead to exciting discoveries that help doctors develop stronger treatments for cancer patients in the future. | This research explores how the nervous system and immune system work together in fighting cancer. Scientists want to understand how nerves and immune cells in tumors talk to each other, affecting tumor growth and the body’s defense against cancer. They found that nerves can release chemicals that change how immune cells act, while immune cells can influence nerve activity, including those that cause pain. The study also shows that stress-related nerve signals can weaken the immune system, making it harder to fight cancer. By learning more about these interactions, researchers hope to find new ways to boost the immune system’s ability to attack cancer, improving treatments. This could lead to better cancer therapies, helping doctors and scientists save more lives. |
| NatRevCan | 5 | 10.1038/s41568-025-00829-5 | Open AI | Sex is a significant determinant of cancer incidence and outcome. The effects of sexual differentiation on normal and cancer biology underly this epidemiology. The resultant sex differences in therapeutic target pathways and processes provide a foundation for developing more personalized cancer treatments. However, our efforts at personalization cannot stop there. Humans also have gender, and sex and gender are highly interactive in individuation. Thus, we will also need to consider how gender-sex interactions (GSI) affect cancer biology and clinical parameters such as the timing of diagnoses, clinical trial enrolment, and the completeness of efficacy and toxicity data. Ignoring the effects of GSI can compromise the quality of basic biological and clinical data and the conclusions drawn from them. This is not to say that GSI will always have a significant effect or any effect at all in every cancer study. Rather, it is to say that we know enough about GSI and human cancer to anticipate measurable differences when GSI are considered in research, enabling us to experimentally determine whether their effects are significant. Here, I delve deeply into GSI and cancer, as this approach to treatment personalization holds great promise to benefit all patients with cancer. | This study explores how both biological sex and social aspects of gender can influence cancer. We know that men and women can develop cancer differently and respond to treatments in unique ways. However, personal identity and gender roles also interact with biology, shaping when cancers are detected, who participates in clinical trials, and how treatments work. By studying these interactions, called gender-sex interactions, researchers can better understand differences in cancer biology and treatment outcomes. Recognizing these effects can improve the design of experiments and the accuracy of research findings. Ultimately, considering both sex and gender in cancer research could help create more personalized and effective treatments, making care safer and more successful for everyone affected by cancer. | Cancer can affect men and women differently, and these differences go beyond biology. This study looks at how both biological sex and social aspects of gender interact to influence cancer development, treatment responses, and outcomes. By understanding these interactions, researchers hope to improve how cancer is studied and treated. For example, gender and sex may affect when cancer is diagnosed, who joins clinical trials, and how well treatments work or cause side effects. Ignoring these factors can lead to incomplete or misleading results. This research explores how considering both sex and gender together can make cancer studies more accurate and help develop treatments that better fit each patient. By paying attention to these differences, scientists aim to create more personalized and effective cancer care, ultimately benefiting everyone affected by the disease. | Cancer affects people differently depending on both their biological sex and their gender. While sex influences how often certain cancers occur and how they respond to treatment, gender can also shape experiences with healthcare, such as when a diagnosis is made or how treatments are tested. This study explores how the combination of sex and gender—called gender-sex interactions—affects cancer biology and patient care. By understanding these interactions, researchers can design better experiments and collect more accurate clinical data. The findings suggest that considering both sex and gender could improve how treatments are personalized, helping doctors provide care that is more effective and safer for each individual. Ultimately, this approach could lead to better outcomes for all people living with cancer. |
| NatRevCan | 5 | 10.1038/s41568-025-00829-6 | Claude | Sex is a significant determinant of cancer incidence and outcome. The effects of sexual differentiation on normal and cancer biology underly this epidemiology. The resultant sex differences in therapeutic target pathways and processes provide a foundation for developing more personalized cancer treatments. However, our efforts at personalization cannot stop there. Humans also have gender, and sex and gender are highly interactive in individuation. Thus, we will also need to consider how gender-sex interactions (GSI) affect cancer biology and clinical parameters such as the timing of diagnoses, clinical trial enrolment, and the completeness of efficacy and toxicity data. Ignoring the effects of GSI can compromise the quality of basic biological and clinical data and the conclusions drawn from them. This is not to say that GSI will always have a significant effect or any effect at all in every cancer study. Rather, it is to say that we know enough about GSI and human cancer to anticipate measurable differences when GSI are considered in research, enabling us to experimentally determine whether their effects are significant. Here, I delve deeply into GSI and cancer, as this approach to treatment personalization holds great promise to benefit all patients with cancer. | Cancer affects men and women differently, but scientists are learning that both biological sex and social gender play important roles in how cancer develops and responds to treatment. Sex refers to physical differences between males and females, while gender includes social behaviors and roles. These two factors work together in complex ways that can change how cancer grows, when people seek medical care, and how well treatments work. For example, gender roles might affect whether someone goes to the doctor early or joins clinical trials to test new treatments. By studying how sex and gender interact, researchers hope to create better, more personalized cancer treatments for everyone. This research could help doctors understand why some treatments work better for certain patients and lead to more effective care. While these interactions won't matter in every cancer study, scientists now know enough to predict when they might be important and can design studies to test their effects. | Cancer affects men and women differently, but scientists are just beginning to understand why. This research explores how both biological sex (the physical differences between males and females) and gender (social and cultural factors) work together to influence cancer development, treatment, and outcomes. The study examines how these combined factors affect when people get diagnosed, whether they join clinical trials, and how well treatments work for different groups. By understanding these interactions, doctors could create more personalized cancer treatments that work better for each individual patient. Currently, most cancer research doesn't consider how sex and gender work together, which may lead to missed opportunities for better care. This research aims to show that considering both factors together could improve cancer treatment for everyone. The findings could help researchers design better studies and help doctors choose the most effective treatments based on each patient's unique biological and social characteristics, ultimately leading to better cancer outcomes for all patients. | Cancer affects men and women differently, but most research only looks at biological sex differences and ignores how gender also plays a role. Sex refers to biological differences like hormones and chromosomes, while gender includes social and cultural factors like lifestyle choices and healthcare behaviors. These two factors work together in complex ways that can change how cancer develops, spreads, and responds to treatment. For example, gender differences might affect when people seek medical care, whether they join clinical trials, or how they follow treatment plans. By studying both sex and gender together, researchers can better understand why some treatments work better for certain patients. This knowledge could help doctors create more personalized cancer treatments that consider not just biological differences between men and women, but also how social and cultural factors influence cancer outcomes. Understanding these combined effects could lead to better treatments and improved survival rates for all cancer patients, making medicine more effective and fair for everyone. |
| NatRevCan | 5 | 10.1038/s41568-025-00829-7 | Gemini | Sex is a significant determinant of cancer incidence and outcome. The effects of sexual differentiation on normal and cancer biology underly this epidemiology. The resultant sex differences in therapeutic target pathways and processes provide a foundation for developing more personalized cancer treatments. However, our efforts at personalization cannot stop there. Humans also have gender, and sex and gender are highly interactive in individuation. Thus, we will also need to consider how gender-sex interactions (GSI) affect cancer biology and clinical parameters such as the timing of diagnoses, clinical trial enrolment, and the completeness of efficacy and toxicity data. Ignoring the effects of GSI can compromise the quality of basic biological and clinical data and the conclusions drawn from them. This is not to say that GSI will always have a significant effect or any effect at all in every cancer study. Rather, it is to say that we know enough about GSI and human cancer to anticipate measurable differences when GSI are considered in research, enabling us to experimentally determine whether their effects are significant. Here, I delve deeply into GSI and cancer, as this approach to treatment personalization holds great promise to benefit all patients with cancer. | Biological differences between males and females, known as sex, have a significant impact on cancer. These differences influence who gets cancer and how well they respond to treatment. This research suggests that we must also consider the social and personal identity aspects of being male or female, known as gender. We need to explore how the interaction between sex and gender affects cancer, including when a person is diagnosed, their participation in clinical studies, and the effectiveness of their treatment. By taking these interactions into account, we can improve the quality of our research and develop more personalized and effective treatments for all cancer patients. | Research shows that being male or female impacts a person’s risk of developing cancer and their treatment outcomes. This is because biological differences between the sexes affect how cancer grows and how well treatments work. However, this study suggests we must also consider a person’s gender, which is different from their biological sex. Gender and sex interact in ways that can change cancer biology and how a person experiences healthcare. For example, these interactions may affect when a person is diagnosed or whether they join a clinical trial. By studying these gender-sex interactions, researchers can gather more accurate data, improve their understanding of cancer, and ultimately develop more effective and personalized treatments that benefit all patients. | Some cancers affect men and women differently, influencing how often they occur and how well people respond to treatment. This is partly due to biological differences between the sexes. However, it is also important to consider the social and cultural aspects of gender, which can interact with biological sex and influence cancer in many ways, from when a person is diagnosed to their participation in clinical trials. By studying how gender and sex work together, this research aims to provide a more complete picture of cancer. The findings could lead to more personalized treatments that are better tailored to each individual, ultimately improving care for all patients. |
| NatRevCan | 5 | 10.1038/s41568-025-00829-8 | DeepSeek | Sex is a significant determinant of cancer incidence and outcome. The effects of sexual differentiation on normal and cancer biology underly this epidemiology. The resultant sex differences in therapeutic target pathways and processes provide a foundation for developing more personalized cancer treatments. However, our efforts at personalization cannot stop there. Humans also have gender, and sex and gender are highly interactive in individuation. Thus, we will also need to consider how gender-sex interactions (GSI) affect cancer biology and clinical parameters such as the timing of diagnoses, clinical trial enrolment, and the completeness of efficacy and toxicity data. Ignoring the effects of GSI can compromise the quality of basic biological and clinical data and the conclusions drawn from them. This is not to say that GSI will always have a significant effect or any effect at all in every cancer study. Rather, it is to say that we know enough about GSI and human cancer to anticipate measurable differences when GSI are considered in research, enabling us to experimentally determine whether their effects are significant. Here, I delve deeply into GSI and cancer, as this approach to treatment personalization holds great promise to benefit all patients with cancer. | This study explores how sex (biological differences) and gender (social and cultural factors) work together to influence cancer risk, treatment response, and patient outcomes. While sex affects cancer biology—changing how tumors grow and respond to drugs—gender can impact when people seek care, join clinical trials, or report side effects. Ignoring these interactions may lead to incomplete or biased research results. By studying both sex and gender together, scientists hope to create more personalized cancer treatments that work better for everyone. The research doesn’t assume these factors always matter—but tests when they do, helping doctors and researchers improve care for all patients. | Cancer affects men and women differently due to both biological sex (like genes and hormones) and gender (like social and cultural factors). These differences can influence cancer risk, treatment effectiveness, and side effects. However, research often overlooks how sex and gender interact, which may lead to incomplete or biased results. This study explains why scientists should study these interactions more carefully—doing so could help doctors personalize treatments, improve diagnosis timing, and make clinical trials more inclusive. By understanding how sex and gender work together in cancer, researchers can develop better, fairer care for all patients. | Cancer affects men and women differently due to both biological sex (like hormones and genes) and gender (like lifestyle and social factors). These differences can influence cancer risk, treatment effectiveness, and side effects. Researchers want to better understand how sex and gender interact in cancer to improve personalized treatments. By studying these interactions, doctors can make better decisions about diagnosis, clinical trials, and drug safety. Ignoring these differences could lead to incomplete or biased research results. While not every study will find major differences, scientists now know enough to check for them, helping make cancer care fairer and more effective for everyone. This research could lead to better, more tailored treatments for all patients. |
| NatRevCan | 5 | 10.1038/s41568-025-00829-9 | Grok | Sex is a significant determinant of cancer incidence and outcome. The effects of sexual differentiation on normal and cancer biology underly this epidemiology. The resultant sex differences in therapeutic target pathways and processes provide a foundation for developing more personalized cancer treatments. However, our efforts at personalization cannot stop there. Humans also have gender, and sex and gender are highly interactive in individuation. Thus, we will also need to consider how gender-sex interactions (GSI) affect cancer biology and clinical parameters such as the timing of diagnoses, clinical trial enrolment, and the completeness of efficacy and toxicity data. Ignoring the effects of GSI can compromise the quality of basic biological and clinical data and the conclusions drawn from them. This is not to say that GSI will always have a significant effect or any effect at all in every cancer study. Rather, it is to say that we know enough about GSI and human cancer to anticipate measurable differences when GSI are considered in research, enabling us to experimentally determine whether their effects are significant. Here, I delve deeply into GSI and cancer, as this approach to treatment personalization holds great promise to benefit all patients with cancer. | Sex differences play a big role in how cancer starts and progresses, influencing who gets it and how well treatments work. This research suggests we must also look at how gender—the social and cultural side of being male, female, or otherwise—interacts with biological sex to shape cancer. The authors aim to explore these gender-sex interactions deeply to create more tailored cancer care that fits each person's unique life. By doing this, scientists can spot real differences in things like when cancer is found, who joins studies, and how safe and effective drugs are. The findings could help the research community get better, more complete data, leading to fairer treatments that help everyone with cancer live longer and healthier lives. | Men and women often face different risks and results from cancer due to their biological sex. This happens because sex affects how bodies develop and how cancer grows. The writer suggests studying these sex differences to create better, more personal cancer treatments. But people also have gender, which is about social roles and behaviors, and gender mixes with sex to shape each person uniquely. So, researchers must look at how gender and sex work together in cancer, like when people get diagnosed or join studies. This could make science more accurate and fair. By checking these interactions in research, we can find out if they matter a lot or not. In the end, understanding gender-sex links promises to improve care for everyone with cancer. | Cancer affects men and women differently, often due to biological sex differences that influence how the disease starts and progresses. This research suggests we must also consider how gender—our social roles and identities—interacts with sex to shape cancer outcomes, like when people get diagnosed or how treatments work. The authors aim to explore these gender-sex interactions deeply to create more tailored cancer care for everyone. By including these factors in studies, scientists can gather better data, avoid mistakes, and develop treatments that truly fit individual needs. This could greatly improve cancer research and help all patients live longer, healthier lives. |
| CancerRes | 1 | 10.1158/0008-5472.CAN-24-3174 | Open AI | Perineural invasion (PNI) is a pivotal prognostic factor in pancreatic cancer, associated with aggressive tumor behavior and adverse patient outcomes. The recognized clinical impact of PNI highlights the need to better understand the molecular mechanisms underlying PNI-induced phenotypes. In this study, we isolated PNI-associated cancer-associated fibroblasts (pCAF), which demonstrated a markedly enhanced capacity to promote neural invasion in pancreatic cancer compared with non-PNI-associated CAFs. Single-cell, high-throughput sequencing and metabolomics data showed a significant upregulation of glycolysis in pCAFs, fostering a high-lactate tumor microenvironment conducive to cancer progression. pCAF-derived lactate was absorbed by tumor cells, facilitating histone H3K18 lactylation. The lactate-induced epigenetic modification activated the transcription of neural invasion-associated genes, such as L1CAM and SLIT1, thereby driving PNI in pancreatic cancer. Further exploration of metabolic reprogramming in pCAFs revealed enhanced acetylation of the glycolytic enzyme GAPDH, which correlated with increased enzymatic activity and glycolytic flux. Targeting GAPDH and lactylation modifications significantly inhibited neural invasion in a genetically engineered mouse model. Clinical data suggested that high levels of H3K18 lactylation correlate with severe PNI and poorer patient prognosis. Together, these findings provide critical insights into the role of CAFs in promoting PNI of pancreatic cancer, highlighting glycolytic reprogramming and lactate-driven histone modifications as potential therapeutic targets for PDAC.Significance: Targeting cancer-associated fibroblast metabolism or histone lactylation in pancreatic cancer cells to reverse epigenetic remodeling induced by lactate accumulation in the tumor microenvironment are potential therapeutic strategies to inhibit perineural invasion. | Pancreatic cancer is a deadly disease, partly because it can spread along nerves, a process called perineural invasion, which makes the cancer harder to treat. In this study, we found that certain support cells in the tumor, called cancer-associated fibroblasts, help cancer cells invade nerves by creating an environment rich in a substance called lactate. The cancer cells use this lactate to switch on genes that encourage nerve invasion. We discovered that these support cells increase their energy production through glycolysis, which raises lactate levels, and that blocking this process can reduce nerve invasion in experimental models. These findings show that targeting the way support cells produce lactate or how cancer cells respond to it could be a new way to slow the spread of pancreatic cancer. Understanding this process may help scientists develop treatments to improve patient outcomes. | Pancreatic cancer is often deadly, partly because it can grow along nearby nerves, making the disease harder to treat. In this study, we explored how certain support cells in the tumor, called cancer-associated fibroblasts, help cancer spread along nerves. We found that these cells produce high amounts of lactate, a simple chemical, which is taken up by cancer cells. This lactate changes the way cancer cell genes are switched on, especially those that help tumors invade nerves. By studying these processes in cells and mice, we discovered that blocking lactate production or its effects could slow nerve invasion. Our findings suggest that targeting the metabolism of these support cells or the gene changes caused by lactate might offer new ways to treat pancreatic cancer. Understanding this interaction could help researchers develop therapies to reduce aggressive tumor growth and improve patient outcomes. | Pancreatic cancer can spread along nerves, a process linked to worse outcomes for patients. This study looked at special support cells in tumors, called cancer-associated fibroblasts, that help cancer invade nerves. We found that fibroblasts near nerve-invading tumors produce a lot of lactate, a type of sugar byproduct, which is absorbed by cancer cells. This lactate changes how the cancer’s genes are switched on, turning on genes that promote nerve invasion. The fibroblasts’ metabolism was also changed to make more lactate, helping the cancer spread. By blocking the enzymes that make lactate or the gene changes it causes, we were able to reduce nerve invasion in mice. Understanding this process helps explain how pancreatic cancer spreads and suggests new ways to treat it by targeting the tumor environment, potentially improving outcomes for patients. |
| CancerRes | 1 | 10.1158/0008-5472.CAN-24-3175 | Claude | Perineural invasion (PNI) is a pivotal prognostic factor in pancreatic cancer, associated with aggressive tumor behavior and adverse patient outcomes. The recognized clinical impact of PNI highlights the need to better understand the molecular mechanisms underlying PNI-induced phenotypes. In this study, we isolated PNI-associated cancer-associated fibroblasts (pCAF), which demonstrated a markedly enhanced capacity to promote neural invasion in pancreatic cancer compared with non-PNI-associated CAFs. Single-cell, high-throughput sequencing and metabolomics data showed a significant upregulation of glycolysis in pCAFs, fostering a high-lactate tumor microenvironment conducive to cancer progression. pCAF-derived lactate was absorbed by tumor cells, facilitating histone H3K18 lactylation. The lactate-induced epigenetic modification activated the transcription of neural invasion-associated genes, such as L1CAM and SLIT1, thereby driving PNI in pancreatic cancer. Further exploration of metabolic reprogramming in pCAFs revealed enhanced acetylation of the glycolytic enzyme GAPDH, which correlated with increased enzymatic activity and glycolytic flux. Targeting GAPDH and lactylation modifications significantly inhibited neural invasion in a genetically engineered mouse model. Clinical data suggested that high levels of H3K18 lactylation correlate with severe PNI and poorer patient prognosis. Together, these findings provide critical insights into the role of CAFs in promoting PNI of pancreatic cancer, highlighting glycolytic reprogramming and lactate-driven histone modifications as potential therapeutic targets for PDAC.Significance: Targeting cancer-associated fibroblast metabolism or histone lactylation in pancreatic cancer cells to reverse epigenetic remodeling induced by lactate accumulation in the tumor microenvironment are potential therapeutic strategies to inhibit perineural invasion. | Pancreatic cancer often spreads along nerves, making it harder to treat and leading to worse outcomes for patients. Scientists wanted to understand why this happens and how to stop it. They studied special cells called cancer-associated fibroblasts that help tumors grow. These cells make a lot of sugar-based energy and produce high amounts of lactic acid. This acid changes how cancer genes work, turning on genes that help cancer spread along nerves. The researchers found that when these support cells make more lactic acid, cancer cells absorb it and use it to change their DNA packaging. This makes the cancer more likely to invade nerves. In mouse studies, blocking this process stopped nerve invasion. Patients with high lactic acid changes in their tumors had worse outcomes. These findings show new ways to treat pancreatic cancer by targeting how support cells feed the tumor or by blocking the acid-driven gene changes. | Pancreatic cancer often spreads along nerves, making it harder to treat and leading to worse outcomes for patients. Scientists wanted to understand why this happens and how to stop it. They studied special cells called cancer-associated fibroblasts that surround tumors. These cells were found to produce high amounts of a substance called lactate through a process similar to how muscles make energy during exercise. When cancer cells absorb this lactate, it changes how their genes work, turning on signals that help the cancer spread along nerves. The researchers discovered that blocking either the production of lactate or preventing cancer cells from using it could stop nerve invasion in laboratory mice. Patients with high lactate levels in their tumors had worse outcomes. These findings reveal new ways that supporting cells help pancreatic cancer spread and point to promising new treatment approaches that could block this deadly process by targeting cell metabolism. | Pancreatic cancer often spreads along nerves, making it harder to treat and leading to worse outcomes for patients. Scientists wanted to understand why this happens and how to stop it. They studied special cells called cancer-associated fibroblasts that help tumors grow. These cells make lots of lactate, a type of sugar waste product. The researchers found that cancer cells near nerves soak up this lactate, which changes how their genes work. This change makes the cancer cells better at invading nerves. The team tested their findings in mice and confirmed that blocking lactate production or its effects on genes can stop nerve invasion. They also looked at patient samples and found that people with more lactate-related gene changes had worse outcomes. This research is important because it reveals new ways to treat pancreatic cancer by targeting how these helper cells feed the tumor and change gene activity. |
| CancerRes | 1 | 10.1158/0008-5472.CAN-24-3176 | Gemini | Perineural invasion (PNI) is a pivotal prognostic factor in pancreatic cancer, associated with aggressive tumor behavior and adverse patient outcomes. The recognized clinical impact of PNI highlights the need to better understand the molecular mechanisms underlying PNI-induced phenotypes. In this study, we isolated PNI-associated cancer-associated fibroblasts (pCAF), which demonstrated a markedly enhanced capacity to promote neural invasion in pancreatic cancer compared with non-PNI-associated CAFs. Single-cell, high-throughput sequencing and metabolomics data showed a significant upregulation of glycolysis in pCAFs, fostering a high-lactate tumor microenvironment conducive to cancer progression. pCAF-derived lactate was absorbed by tumor cells, facilitating histone H3K18 lactylation. The lactate-induced epigenetic modification activated the transcription of neural invasion-associated genes, such as L1CAM and SLIT1, thereby driving PNI in pancreatic cancer. Further exploration of metabolic reprogramming in pCAFs revealed enhanced acetylation of the glycolytic enzyme GAPDH, which correlated with increased enzymatic activity and glycolytic flux. Targeting GAPDH and lactylation modifications significantly inhibited neural invasion in a genetically engineered mouse model. Clinical data suggested that high levels of H3K18 lactylation correlate with severe PNI and poorer patient prognosis. Together, these findings provide critical insights into the role of CAFs in promoting PNI of pancreatic cancer, highlighting glycolytic reprogramming and lactate-driven histone modifications as potential therapeutic targets for PDAC.Significance: Targeting cancer-associated fibroblast metabolism or histone lactylation in pancreatic cancer cells to reverse epigenetic remodeling induced by lactate accumulation in the tumor microenvironment are potential therapeutic strategies to inhibit perineural invasion. | In this study, researchers investigated how pancreatic cancer spreads along nerves, a process that makes the disease more aggressive. They found that specific support cells, called cancer-associated fibroblasts, help create a high-energy environment around the tumor by producing large amounts of a substance called lactate. This lactate is then absorbed by the cancer cells and changes how their genes are turned on and off. This change, called lactylation, causes the cancer cells to become more invasive and spread into nearby nerves. By understanding this process, scientists can explore new treatment strategies that target either the support cells' energy production or the changes they cause in the cancer cells. Such treatments could potentially stop the cancer from spreading along nerves and lead to better outcomes for patients. | Cancer-associated fibroblasts, which are special cells found around tumors, play a major role in pancreatic cancer's aggressive behavior. This study found that a specific type of these fibroblasts, called pCAFs, create an environment with high levels of lactate, a substance produced during energy use. The high lactate levels are then absorbed by the cancer cells, causing a change called histone lactylation. This change turns on certain genes that make the cancer cells more likely to spread along nerves, a process known as perineural invasion. This invasion is linked to worse patient outcomes. By understanding this process, researchers believe they can develop new treatments that target these fibroblasts or the lactate-driven changes in the cancer cells, potentially slowing or stopping the cancer's spread and improving the lives of patients. | This research explores how specific cells, called cancer-associated fibroblasts, create a special environment that helps pancreatic cancer spread. By studying these cells, we discovered that they produce a lot of lactate, which is a byproduct of sugar metabolism. This high level of lactate causes changes in the cancer cells' DNA, turning on genes that enable the cancer to invade surrounding nerves. This process, known as perineural invasion, makes the cancer more aggressive and difficult to treat. Our findings suggest that blocking this lactate production or the resulting changes in the cancer cells could be a new way to stop the cancer from spreading along nerves. Ultimately, this research provides new targets for developing more effective treatments for pancreatic cancer patients. |
| CancerRes | 1 | 10.1158/0008-5472.CAN-24-3177 | DeepSeek | Perineural invasion (PNI) is a pivotal prognostic factor in pancreatic cancer, associated with aggressive tumor behavior and adverse patient outcomes. The recognized clinical impact of PNI highlights the need to better understand the molecular mechanisms underlying PNI-induced phenotypes. In this study, we isolated PNI-associated cancer-associated fibroblasts (pCAF), which demonstrated a markedly enhanced capacity to promote neural invasion in pancreatic cancer compared with non-PNI-associated CAFs. Single-cell, high-throughput sequencing and metabolomics data showed a significant upregulation of glycolysis in pCAFs, fostering a high-lactate tumor microenvironment conducive to cancer progression. pCAF-derived lactate was absorbed by tumor cells, facilitating histone H3K18 lactylation. The lactate-induced epigenetic modification activated the transcription of neural invasion-associated genes, such as L1CAM and SLIT1, thereby driving PNI in pancreatic cancer. Further exploration of metabolic reprogramming in pCAFs revealed enhanced acetylation of the glycolytic enzyme GAPDH, which correlated with increased enzymatic activity and glycolytic flux. Targeting GAPDH and lactylation modifications significantly inhibited neural invasion in a genetically engineered mouse model. Clinical data suggested that high levels of H3K18 lactylation correlate with severe PNI and poorer patient prognosis. Together, these findings provide critical insights into the role of CAFs in promoting PNI of pancreatic cancer, highlighting glycolytic reprogramming and lactate-driven histone modifications as potential therapeutic targets for PDAC.Significance: Targeting cancer-associated fibroblast metabolism or histone lactylation in pancreatic cancer cells to reverse epigenetic remodeling induced by lactate accumulation in the tumor microenvironment are potential therapeutic strategies to inhibit perineural invasion. | Pancreatic cancer often spreads along nerves, a process called perineural invasion (PNI), which makes the disease harder to treat. In this study, researchers found that certain cells in the tumor, called cancer-associated fibroblasts (CAFs), produce high levels of lactate, a byproduct of sugar breakdown. This lactate creates an environment that helps cancer cells invade nerves. The lactate also changes how cancer cells use their genes, making them more aggressive. By blocking these changes or targeting the enzymes that produce lactate, the researchers slowed nerve invasion in lab tests. Patients with high lactate-related changes in their tumors had worse outcomes, suggesting that stopping this process could be a new way to treat pancreatic cancer. This discovery could lead to better therapies targeting the tumor’s environment. | This study explores why pancreatic cancer often spreads along nerves, a process called perineural invasion (PNI), which makes the disease harder to treat. Researchers found that certain cells in the tumor, called cancer-associated fibroblasts (CAFs), produce large amounts of lactate, a byproduct of sugar breakdown. This lactate feeds cancer cells, causing changes that help them invade nerves. By studying lab models and patient samples, the team discovered that blocking lactate production or its effects reduced nerve invasion. These findings suggest that targeting lactate or related processes could lead to new treatments for pancreatic cancer, potentially improving survival for patients with aggressive tumors. | Pancreatic cancer often spreads along nerves, making it harder to treat and leading to worse outcomes for patients. In this study, researchers found that certain cells in the tumor, called cancer-associated fibroblasts (CAFs), produce a lot of lactate, a byproduct of sugar breakdown. This lactate fuels cancer growth and helps the tumor invade nerves by changing how certain genes work. The study shows that blocking lactate production or its effects could slow nerve invasion and improve survival. These findings suggest new ways to treat pancreatic cancer by targeting the tumor’s energy supply and its ability to hijack nearby cells. This could lead to better therapies for patients with this aggressive disease. |
| CancerRes | 1 | 10.1158/0008-5472.CAN-24-3178 | Grok | Perineural invasion (PNI) is a pivotal prognostic factor in pancreatic cancer, associated with aggressive tumor behavior and adverse patient outcomes. The recognized clinical impact of PNI highlights the need to better understand the molecular mechanisms underlying PNI-induced phenotypes. In this study, we isolated PNI-associated cancer-associated fibroblasts (pCAF), which demonstrated a markedly enhanced capacity to promote neural invasion in pancreatic cancer compared with non-PNI-associated CAFs. Single-cell, high-throughput sequencing and metabolomics data showed a significant upregulation of glycolysis in pCAFs, fostering a high-lactate tumor microenvironment conducive to cancer progression. pCAF-derived lactate was absorbed by tumor cells, facilitating histone H3K18 lactylation. The lactate-induced epigenetic modification activated the transcription of neural invasion-associated genes, such as L1CAM and SLIT1, thereby driving PNI in pancreatic cancer. Further exploration of metabolic reprogramming in pCAFs revealed enhanced acetylation of the glycolytic enzyme GAPDH, which correlated with increased enzymatic activity and glycolytic flux. Targeting GAPDH and lactylation modifications significantly inhibited neural invasion in a genetically engineered mouse model. Clinical data suggested that high levels of H3K18 lactylation correlate with severe PNI and poorer patient prognosis. Together, these findings provide critical insights into the role of CAFs in promoting PNI of pancreatic cancer, highlighting glycolytic reprogramming and lactate-driven histone modifications as potential therapeutic targets for PDAC.Significance: Targeting cancer-associated fibroblast metabolism or histone lactylation in pancreatic cancer cells to reverse epigenetic remodeling induced by lactate accumulation in the tumor microenvironment are potential therapeutic strategies to inhibit perineural invasion. | Pancreatic cancer often spreads along nerves, making it harder to treat and worsening patient chances. This study looks at why that happens and how to stop it. Researchers found special support cells near the cancer, called cancer-associated fibroblasts, that create a lot of lactate, a waste product from breaking down sugar. Cancer cells soak up this lactate, which changes their genes to help them invade nerves. By studying cells, genes, and mouse models, the team discovered blocking these changes or the support cells' energy process can slow the spread. These findings could help scientists develop new treatments to prevent nerve invasion, improving outcomes for people with pancreatic cancer. | Pancreatic cancer often spreads along nerves, making it harder to treat and worsening patient outcomes. This study looks at why that happens and how to stop it. Researchers focused on special support cells called cancer-associated fibroblasts near these nerves, which produce high levels of lactate, a waste product from sugar breakdown. Cancer cells absorb this lactate, changing their genes to help them invade nerves more easily. By testing in lab cells, mice, and patient samples, the team found that blocking this lactate process or the support cells' sugar use reduced nerve invasion. These insights could lead to new treatments that target this environment, improving survival for people with pancreatic cancer by preventing spread. | Pancreatic cancer often spreads along nerves, which makes it harder to treat and worsens patient outcomes. This study looks at why this happens and how to stop it. Researchers studied special cells called cancer-associated fibroblasts near these nerves. They found these cells create a lot of lactate, a substance that helps cancer cells change their genes to invade nerves more easily. By using lab tests, gene studies, and mouse models, the team showed that blocking this lactate or the gene changes reduced nerve invasion. These discoveries could lead to new treatments that target these cells or lactate buildup, helping doctors slow cancer spread and improve survival for people with pancreatic cancer. |
| CancerRes | 2 | 10.1158/0008-5472.CAN-24-1880 | Open AI | Black individuals experience worse survival after a diagnosis of high-grade serous ovarian carcinoma (HGSC) than White individuals and are underrepresented in ovarian cancer research. To date, the understanding of the molecular and genomic heterogeneity of HGSC is based primarily on the evaluation of tumors from White individuals. In the present study, we performed whole-exome sequencing on HGSC samples from 211 Black patients to identify significantly mutated genes and characterize mutational signatures, assessing their distributions by gene expression subtypes. The occurrence and frequency of somatic mutations and signatures by self-reported race were compared with historic data from The Cancer Genome Atlas (TCGA). Despite technical differences (e.g., formalin-fixed vs. fresh-frozen tissue), the distribution of mutations and their variant classifications for major HGSC genes were nearly identical across study populations. However, de novo significantly mutated gene analysis identified genes not previously reported in TCGA analysis, including the oncogene KRAS and the potential tumor suppressor OBSCN. The prevalence of the homologous recombination deficiency signature was higher among Black individuals with the immunoreactive gene expression subtype compared with the mesenchymal and proliferative subtypes. These findings were confirmed by comparing the data from Black patients with those from 123 White patients with identical tissue collection and processing. Overall, this study suggests that, although most features of HGSC tumor phenotypes are similar in Black and White populations, there may be clinically relevant differences. If validated, these phenotypes may be important for clinical decision-making and would have been missed by characterizing tumors from White individuals only.Significance: Elucidation of the somatic mutational landscape of high-grade serous ovarian carcinoma in Black individuals, who experience poor survival and are underrepresented in research, could inform patient prognosis and enable precision medicine opportunities. | High-grade serous ovarian cancer is a deadly form of ovarian cancer that often affects Black individuals more severely, yet most research has focused on White patients. This study analyzed the genetic makeup of tumors from 211 Black patients to find mutations that may drive the disease. The researchers discovered that, while many tumor features are similar across races, some genes, including KRAS and OBSCN, were uniquely altered in Black patients. They also found differences in specific genetic patterns linked to DNA repair in certain tumor types. These findings suggest that Black patients may have distinct tumor characteristics that could influence how they respond to treatment. By highlighting these differences, this research could help doctors better predict outcomes and tailor therapies, moving toward more personalized care for all patients with this aggressive cancer. | High-grade serous ovarian cancer is a serious type of ovarian cancer that affects people of all races, but Black individuals often have worse outcomes than White individuals. Most research so far has focused on tumors from White patients, leaving a gap in understanding how this cancer behaves in Black patients. In this study, we analyzed the genes of tumors from 211 Black patients to identify important mutations and patterns. We found that, while many features of the cancer were similar across races, some genetic changes were unique to Black patients. These differences could affect how the cancer grows and responds to treatment. Understanding these unique features may help doctors predict outcomes more accurately and tailor treatments for Black patients. This work highlights the importance of including diverse populations in cancer research to improve care for everyone. | High-grade serous ovarian cancer is a serious type of ovarian cancer that affects people of all races, but Black individuals often have worse outcomes and have been included less in research. In this study, we analyzed the complete set of genes in tumors from 211 Black patients to see which genes were changed and how these changes might affect cancer behavior. We found that while most tumor features were similar to those seen in White patients, some gene changes, including in KRAS and OBSCN, were unique to Black patients. We also found that certain patterns linked to DNA repair problems were more common in specific tumor types. Understanding these differences can help doctors better predict outcomes and may guide more personalized treatments in the future. This study highlights the importance of including diverse populations in cancer research to improve care for everyone. |
| CancerRes | 2 | 10.1158/0008-5472.CAN-24-1881 | Claude | Black individuals experience worse survival after a diagnosis of high-grade serous ovarian carcinoma (HGSC) than White individuals and are underrepresented in ovarian cancer research. To date, the understanding of the molecular and genomic heterogeneity of HGSC is based primarily on the evaluation of tumors from White individuals. In the present study, we performed whole-exome sequencing on HGSC samples from 211 Black patients to identify significantly mutated genes and characterize mutational signatures, assessing their distributions by gene expression subtypes. The occurrence and frequency of somatic mutations and signatures by self-reported race were compared with historic data from The Cancer Genome Atlas (TCGA). Despite technical differences (e.g., formalin-fixed vs. fresh-frozen tissue), the distribution of mutations and their variant classifications for major HGSC genes were nearly identical across study populations. However, de novo significantly mutated gene analysis identified genes not previously reported in TCGA analysis, including the oncogene KRAS and the potential tumor suppressor OBSCN. The prevalence of the homologous recombination deficiency signature was higher among Black individuals with the immunoreactive gene expression subtype compared with the mesenchymal and proliferative subtypes. These findings were confirmed by comparing the data from Black patients with those from 123 White patients with identical tissue collection and processing. Overall, this study suggests that, although most features of HGSC tumor phenotypes are similar in Black and White populations, there may be clinically relevant differences. If validated, these phenotypes may be important for clinical decision-making and would have been missed by characterizing tumors from White individuals only.Significance: Elucidation of the somatic mutational landscape of high-grade serous ovarian carcinoma in Black individuals, who experience poor survival and are underrepresented in research, could inform patient prognosis and enable precision medicine opportunities. | Black women with a deadly type of ovarian cancer called high-grade serous ovarian carcinoma have worse survival rates than White women, yet they are rarely included in cancer research studies. Most of what doctors know about this cancer comes from studying tumors in White patients only. This research team wanted to see if there were important differences in the cancer's genetic makeup between Black and White women that could explain survival differences. The scientists studied the complete genetic code of tumors from 211 Black women with this cancer and compared them to data from White patients. They found that while most genetic features were the same between groups, there were some key differences. Black women had mutations in certain genes that were not found in previous studies of mostly White patients, including genes that could make the cancer grow or help stop it. These genetic differences might help explain why outcomes differ and could lead to better, more personalized treatments for all women with this cancer. | Black women with a deadly type of ovarian cancer called high-grade serous ovarian carcinoma have worse survival rates than White women, yet most cancer research has only studied tumors from White patients. This study aimed to understand if there are important differences in the genetic makeup of these tumors between Black and White women that could explain survival differences and improve treatment. Researchers analyzed the complete genetic code of tumor samples from 211 Black women and compared them to existing data from White patients. They found that while most genetic features were similar between groups, Black women's tumors had some unique characteristics, including different mutations in genes like KRAS and different patterns of DNA repair problems depending on tumor type. These genetic differences might help explain why Black women have worse outcomes and could lead to better personalized treatments. By including more diverse patients in cancer research, scientists can ensure that new treatments work for everyone, not just the populations that have been studied most often. | Black women with a deadly type of ovarian cancer called high-grade serous ovarian cancer have worse survival rates than White women, yet most cancer research has only studied tumors from White patients. This study aimed to understand if there are genetic differences in tumors between Black and White women that could explain these survival differences. Researchers analyzed the DNA from ovarian cancer tumors in 211 Black women and compared their findings to previous studies of White women. They found that while most genetic features were similar between the two groups, there were some important differences. Black women's tumors had mutations in certain genes that had not been seen before in previous studies of mostly White patients, including a gene called KRAS that helps cancer grow. The researchers also found differences in DNA repair patterns that varied by tumor type. These genetic differences could help doctors better understand why Black women have worse outcomes and potentially lead to more personalized treatments. This research shows why it is important to include diverse populations in cancer studies to ensure all patients receive the best possible care. |
| CancerRes | 2 | 10.1158/0008-5472.CAN-24-1882 | Gemini | Black individuals experience worse survival after a diagnosis of high-grade serous ovarian carcinoma (HGSC) than White individuals and are underrepresented in ovarian cancer research. To date, the understanding of the molecular and genomic heterogeneity of HGSC is based primarily on the evaluation of tumors from White individuals. In the present study, we performed whole-exome sequencing on HGSC samples from 211 Black patients to identify significantly mutated genes and characterize mutational signatures, assessing their distributions by gene expression subtypes. The occurrence and frequency of somatic mutations and signatures by self-reported race were compared with historic data from The Cancer Genome Atlas (TCGA). Despite technical differences (e.g., formalin-fixed vs. fresh-frozen tissue), the distribution of mutations and their variant classifications for major HGSC genes were nearly identical across study populations. However, de novo significantly mutated gene analysis identified genes not previously reported in TCGA analysis, including the oncogene KRAS and the potential tumor suppressor OBSCN. The prevalence of the homologous recombination deficiency signature was higher among Black individuals with the immunoreactive gene expression subtype compared with the mesenchymal and proliferative subtypes. These findings were confirmed by comparing the data from Black patients with those from 123 White patients with identical tissue collection and processing. Overall, this study suggests that, although most features of HGSC tumor phenotypes are similar in Black and White populations, there may be clinically relevant differences. If validated, these phenotypes may be important for clinical decision-making and would have been missed by characterizing tumors from White individuals only.Significance: Elucidation of the somatic mutational landscape of high-grade serous ovarian carcinoma in Black individuals, who experience poor survival and are underrepresented in research, could inform patient prognosis and enable precision medicine opportunities. | This research examines the genetic makeup of high-grade serous ovarian cancer in Black individuals, a group that experiences worse survival rates and is not often included in ovarian cancer studies. By analyzing tumor samples from 211 Black patients, researchers aimed to identify key genetic changes and compare them to data from mostly White patients. The study found that while many cancer features were similar across racial groups, there were some potentially important differences. For example, specific gene mutations, such as those in the KRAS gene, were found in the Black patient group but had not been previously highlighted in studies of White patients. Additionally, the study noted a higher prevalence of a specific genetic signature, called the homologous recombination deficiency signature, within a certain tumor subtype among Black individuals. These findings suggest that there may be clinically important genetic differences in ovarian cancer between Black and White individuals. If confirmed by future studies, these discoveries could lead to more personalized treatment options and improved care for Black patients with this disease. | This study explores the genetic makeup of high-grade serous ovarian cancer in Black individuals, a group that has historically been underrepresented in research and experiences worse health outcomes. The goal of the research was to identify any unique genetic features in these tumors that might help explain the differences in survival and could lead to new treatment options. By analyzing tumor samples from 211 Black patients, the researchers compared their findings to existing data from White patients. While many genetic characteristics were similar across both groups, the study uncovered some notable differences. For example, some genes were found to be mutated in tumors from Black patients that were not previously identified in studies of White patients. The findings suggest that although most features of this cancer are the same in both populations, there may be important differences that could impact how the disease is treated. Identifying these specific features could lead to more personalized and effective treatments, ultimately helping to improve outcomes for Black patients with this type of cancer. | In this study, researchers aimed to understand the genetic makeup of high-grade serous ovarian cancer in Black individuals, who often have worse health outcomes and are not well-represented in research. By analyzing tumor samples from 211 Black patients, the study found that while many features of the cancer are similar to those seen in White patients, there are also some unique differences. For example, the study identified specific gene mutations, such as one in the KRAS gene, which had not been previously reported in similar large-scale studies that primarily included White individuals. The research also found that a particular type of genetic signature linked to the cancer's development was more common in Black patients with a certain tumor type. If these findings are confirmed in larger studies, they could help doctors better predict patient outcomes and lead to new, more personalized treatment options, ultimately helping to close the health disparity gap for Black patients with this type of cancer. |
| CancerRes | 2 | 10.1158/0008-5472.CAN-24-1883 | DeepSeek | Black individuals experience worse survival after a diagnosis of high-grade serous ovarian carcinoma (HGSC) than White individuals and are underrepresented in ovarian cancer research. To date, the understanding of the molecular and genomic heterogeneity of HGSC is based primarily on the evaluation of tumors from White individuals. In the present study, we performed whole-exome sequencing on HGSC samples from 211 Black patients to identify significantly mutated genes and characterize mutational signatures, assessing their distributions by gene expression subtypes. The occurrence and frequency of somatic mutations and signatures by self-reported race were compared with historic data from The Cancer Genome Atlas (TCGA). Despite technical differences (e.g., formalin-fixed vs. fresh-frozen tissue), the distribution of mutations and their variant classifications for major HGSC genes were nearly identical across study populations. However, de novo significantly mutated gene analysis identified genes not previously reported in TCGA analysis, including the oncogene KRAS and the potential tumor suppressor OBSCN. The prevalence of the homologous recombination deficiency signature was higher among Black individuals with the immunoreactive gene expression subtype compared with the mesenchymal and proliferative subtypes. These findings were confirmed by comparing the data from Black patients with those from 123 White patients with identical tissue collection and processing. Overall, this study suggests that, although most features of HGSC tumor phenotypes are similar in Black and White populations, there may be clinically relevant differences. If validated, these phenotypes may be important for clinical decision-making and would have been missed by characterizing tumors from White individuals only.Significance: Elucidation of the somatic mutational landscape of high-grade serous ovarian carcinoma in Black individuals, who experience poor survival and are underrepresented in research, could inform patient prognosis and enable precision medicine opportunities. | Black women with high-grade serous ovarian cancer (HGSC) often have worse outcomes than White women, but most research on this cancer has focused on White patients. To fix this gap, scientists studied tumors from 211 Black patients, comparing their genetic changes to data from mostly White patients. They found that while most mutations were similar between groups, Black patients had unique changes in genes like KRAS (linked to cancer growth) and OBSCN (which may suppress tumors). Black patients also showed more DNA repair problems in certain tumor types. These differences could affect treatment choices. By including Black patients—who are often left out of cancer studies—this research helps ensure all women get personalized care. If confirmed, these findings may lead to better therapies and improved survival for Black women with HGSC. | Black women with high-grade serous ovarian cancer (HGSC) often have worse outcomes than White women, but most research on this cancer has focused on White patients. To fix this gap, scientists studied tumors from 211 Black patients, comparing their genetic changes to past data from mostly White patients. They found that while most mutations were similar between groups, Black patients had unique changes in genes like KRAS (linked to cancer growth) and OBSCN (which may suppress tumors). Black patients also showed more DNA repair problems in certain tumor types, which could affect treatment responses. These differences might help explain survival gaps and guide better care. By including more Black patients in research, doctors can uncover hidden clues to improve precision medicine for everyone. | Black women with high-grade serous ovarian cancer (HGSC) often have worse outcomes than White women, but most research on this cancer has focused on White patients. This study examined the genetic makeup of tumors from 211 Black patients to see if differences might explain survival gaps. The researchers found that while most tumor features were similar between Black and White patients, they discovered new gene mutations (like KRAS and OBSCN) more common in Black individuals. They also found that certain tumor subtypes linked to treatment response were more frequent in Black patients. These differences could help doctors tailor treatments better. By including more Black patients in cancer research, scientists can uncover hidden clues to improve care for everyone. |
| CancerRes | 2 | 10.1158/0008-5472.CAN-24-1884 | Grok | Black individuals experience worse survival after a diagnosis of high-grade serous ovarian carcinoma (HGSC) than White individuals and are underrepresented in ovarian cancer research. To date, the understanding of the molecular and genomic heterogeneity of HGSC is based primarily on the evaluation of tumors from White individuals. In the present study, we performed whole-exome sequencing on HGSC samples from 211 Black patients to identify significantly mutated genes and characterize mutational signatures, assessing their distributions by gene expression subtypes. The occurrence and frequency of somatic mutations and signatures by self-reported race were compared with historic data from The Cancer Genome Atlas (TCGA). Despite technical differences (e.g., formalin-fixed vs. fresh-frozen tissue), the distribution of mutations and their variant classifications for major HGSC genes were nearly identical across study populations. However, de novo significantly mutated gene analysis identified genes not previously reported in TCGA analysis, including the oncogene KRAS and the potential tumor suppressor OBSCN. The prevalence of the homologous recombination deficiency signature was higher among Black individuals with the immunoreactive gene expression subtype compared with the mesenchymal and proliferative subtypes. These findings were confirmed by comparing the data from Black patients with those from 123 White patients with identical tissue collection and processing. Overall, this study suggests that, although most features of HGSC tumor phenotypes are similar in Black and White populations, there may be clinically relevant differences. If validated, these phenotypes may be important for clinical decision-making and would have been missed by characterizing tumors from White individuals only.Significance: Elucidation of the somatic mutational landscape of high-grade serous ovarian carcinoma in Black individuals, who experience poor survival and are underrepresented in research, could inform patient prognosis and enable precision medicine opportunities. | Black people often face worse outcomes from high-grade serous ovarian cancer than White people, but most studies focus on White patients. This research aims to better understand the disease in Black individuals by examining the genes in tumor samples from 211 Black women using advanced DNA sequencing. The team looked for changed genes and patterns, then compared them to data from mostly White patients. They found many similarities, but also new changes in genes like KRAS and OBSCN, plus a higher rate of a specific DNA repair issue in certain tumor types among Black women. These differences, confirmed against samples from 123 White women, could lead to better ways to predict outcomes and tailor treatments. By including more diverse groups, this work helps the science community create fairer medicine that benefits everyone, avoiding missed chances for better care. | Black people often face worse outcomes from high-grade serous ovarian cancer than white people, and they are not well represented in studies. Most knowledge about this cancer comes from research on white patients. This study aims to better understand the genetic changes in tumors from black patients to see if there are differences that could improve care. Researchers looked at DNA from tumors of 211 black women using a method called whole-exome sequencing. They found most genetic features are similar to those in white patients, but spotted new changes in genes like KRAS and OBSCN. Also, a key DNA repair issue was more common in certain tumor types among black women. These differences, if confirmed, could help doctors make better treatment choices and highlight why studying diverse groups is vital for fair medical advances. | Black women often face worse outcomes after being diagnosed with high-grade serous ovarian cancer compared to White women, and they are not well represented in research studies. This study aimed to examine the genetic makeup of tumors from 211 Black patients using DNA sequencing, identify key mutations and patterns, and compare them to data mostly from White patients. Researchers found that while many genetic features are similar between Black and White groups, Black patients showed unique mutations in genes like KRAS and OBSCN, and a higher rate of a specific DNA repair defect in certain tumor types. These differences, confirmed by direct comparisons, could explain varying survival rates and guide better, personalized treatments. By including diverse groups, this work highlights overlooked details that may improve care and outcomes for everyone, especially underserved communities. |
| CancerRes | 3 | 10.1158/0008-5472.CAN-24-1518 | Open AI | Early detection of lung cancer is important for improving patient survival rates. Liquid biopsy using whole-genome sequencing of cell-free DNA (cfDNA) offers a promising avenue for lung cancer screening, providing a potential alternative or complementary approach to current screening modalities. Here, we aimed to develop and validate an approach by integrating fragment and genomic features of cfDNA to enhance lung cancer detection accuracy across diverse populations. Deep learning-based classifiers were trained using comprehensive cfDNA fragmentomic features from participants in multi-institutional studies, including a Korean discovery dataset (218 patients with lung cancer and 2,559 controls), a Korean validation dataset (111 patients with lung cancer and 1,136 controls), and an independent Caucasian validation cohort (50 patients with lung cancer and 50 controls). In the discovery dataset, classifiers using fragment end motif by size, a feature that captures both fragment end motif and size profiles, outperformed standalone fragment end motif and fragment size classifiers, achieving an area under the curve (AUC) of 0.917. The ensemble classifier integrating fragment end motif by size and genomic coverage achieved an improved performance, with an AUC of 0.937. This performance extended to the Korean validation dataset and demonstrated ethnic generalizability in the Caucasian validation cohort. Overall, the development of a deep learning-based classifier integrating cfDNA fragmentomic and genomic features in this study highlights the potential for accurate lung cancer detection across diverse populations.Significance: Evaluating fragment-based features and genomic coverage in cell-free DNA offers an accurate lung cancer screening method, promising improvements in early cancer detection and addressing challenges associated with current screening methods. | Early detection of lung cancer can save lives, but current screening methods have limitations. This study explores a new, non-invasive approach using a blood test to analyze tiny fragments of DNA released by cells into the bloodstream. By examining both the patterns at the ends of these DNA fragments and the way the DNA is distributed across the genome, and by combining this information with advanced computer learning techniques, we were able to detect lung cancer with high accuracy. Our method was tested on large groups of participants from different ethnic backgrounds, showing consistent results. This approach could provide a more accurate and widely applicable tool for lung cancer screening, helping doctors identify the disease earlier and giving patients a better chance of effective treatment. | Early detection of lung cancer can save lives, but current screening methods have limitations. This study tested a new blood test that looks at tiny fragments of DNA released by cells into the bloodstream, combined with patterns in the DNA itself. Using advanced computer learning, researchers analyzed DNA fragments from people with and without lung cancer across different countries and ethnic groups. They found that examining both the shape and location of these DNA fragments helped detect lung cancer more accurately than looking at either feature alone. This method worked well in multiple populations, suggesting it could be widely useful. By improving the accuracy of blood-based lung cancer detection, this approach may help catch cancer earlier, guide treatment decisions, and reduce deaths, offering a safer and less invasive alternative to traditional screening methods. | Lung cancer is one of the deadliest cancers, and finding it early can save lives. This study explored a new blood test that looks for tiny pieces of DNA released by tumors into the bloodstream. By analyzing both the size and patterns at the ends of these DNA fragments, along with other genetic information, the researchers trained a computer program to tell the difference between people with and without lung cancer. The method was tested in large groups of people from different countries and worked accurately across diverse populations. This approach could make lung cancer screening easier, less invasive, and more reliable, helping doctors detect the disease earlier and give patients a better chance of successful treatment. These findings may also guide future research in using blood-based DNA tests for early cancer detection. |
| CancerRes | 3 | 10.1158/0008-5472.CAN-24-1519 | Claude | Early detection of lung cancer is important for improving patient survival rates. Liquid biopsy using whole-genome sequencing of cell-free DNA (cfDNA) offers a promising avenue for lung cancer screening, providing a potential alternative or complementary approach to current screening modalities. Here, we aimed to develop and validate an approach by integrating fragment and genomic features of cfDNA to enhance lung cancer detection accuracy across diverse populations. Deep learning-based classifiers were trained using comprehensive cfDNA fragmentomic features from participants in multi-institutional studies, including a Korean discovery dataset (218 patients with lung cancer and 2,559 controls), a Korean validation dataset (111 patients with lung cancer and 1,136 controls), and an independent Caucasian validation cohort (50 patients with lung cancer and 50 controls). In the discovery dataset, classifiers using fragment end motif by size, a feature that captures both fragment end motif and size profiles, outperformed standalone fragment end motif and fragment size classifiers, achieving an area under the curve (AUC) of 0.917. The ensemble classifier integrating fragment end motif by size and genomic coverage achieved an improved performance, with an AUC of 0.937. This performance extended to the Korean validation dataset and demonstrated ethnic generalizability in the Caucasian validation cohort. Overall, the development of a deep learning-based classifier integrating cfDNA fragmentomic and genomic features in this study highlights the potential for accurate lung cancer detection across diverse populations.Significance: Evaluating fragment-based features and genomic coverage in cell-free DNA offers an accurate lung cancer screening method, promising improvements in early cancer detection and addressing challenges associated with current screening methods. | Scientists have developed a new blood test that can detect lung cancer early by studying tiny pieces of DNA that float freely in blood. Finding lung cancer early is crucial because it greatly improves a patient's chances of survival. Current screening methods have limitations, so researchers wanted to create a better approach using a simple blood draw. The team used advanced computer programs to analyze DNA fragments from over 4,000 people across different ethnic groups, including Korean and Caucasian populations. They looked at specific patterns in how these DNA pieces break apart and their sizes, along with other genetic features. Their new method correctly identified lung cancer 93.7% of the time in testing. The blood test worked well across different ethnic groups, showing it could be used worldwide. This breakthrough could lead to easier, more accurate lung cancer screening that doesn't require uncomfortable procedures like chest scans. The research shows promise for catching lung cancer earlier in more patients, potentially saving many lives through improved early detection methods. | Finding lung cancer early helps save lives, but current tests are not perfect. Scientists wanted to create a better way to screen for lung cancer using a simple blood test. They studied tiny pieces of DNA that float freely in blood, which come from both normal cells and cancer cells. The researchers looked at the shape, size, and patterns of these DNA pieces in blood samples from over 4,000 people in Korea and the United States, including healthy people and those with lung cancer. They used computer programs to learn the differences between DNA from healthy people and cancer patients. Their new method was very good at telling the difference, correctly identifying lung cancer about 94% of the time. The test worked well for both Korean and Caucasian people, showing it could help many different groups. This blood test could make lung cancer screening easier, cheaper, and less scary than current methods, helping doctors find cancer sooner when treatment works best. | Blood tests can now help doctors find lung cancer early by looking at tiny pieces of DNA that float freely in our blood. Finding lung cancer early is very important because it helps more people survive this disease. Current screening methods like chest scans work well but have some problems, so scientists wanted to create a better way to catch lung cancer sooner. The researchers studied blood samples from over 4,000 people in Korea and the United States, including those with and without lung cancer. They used computer programs to look at special patterns in the DNA pieces, focusing on how big they are and what their ends look like. When they combined these DNA patterns with other genetic information, they could correctly identify lung cancer in 94% of cases. The test worked well for both Korean and Caucasian people, showing it could help patients from different backgrounds. This new blood test could make lung cancer screening easier, cheaper, and more accurate than current methods, potentially saving many lives through earlier detection. |
| CancerRes | 3 | 10.1158/0008-5472.CAN-24-1520 | Gemini | Early detection of lung cancer is important for improving patient survival rates. Liquid biopsy using whole-genome sequencing of cell-free DNA (cfDNA) offers a promising avenue for lung cancer screening, providing a potential alternative or complementary approach to current screening modalities. Here, we aimed to develop and validate an approach by integrating fragment and genomic features of cfDNA to enhance lung cancer detection accuracy across diverse populations. Deep learning-based classifiers were trained using comprehensive cfDNA fragmentomic features from participants in multi-institutional studies, including a Korean discovery dataset (218 patients with lung cancer and 2,559 controls), a Korean validation dataset (111 patients with lung cancer and 1,136 controls), and an independent Caucasian validation cohort (50 patients with lung cancer and 50 controls). In the discovery dataset, classifiers using fragment end motif by size, a feature that captures both fragment end motif and size profiles, outperformed standalone fragment end motif and fragment size classifiers, achieving an area under the curve (AUC) of 0.917. The ensemble classifier integrating fragment end motif by size and genomic coverage achieved an improved performance, with an AUC of 0.937. This performance extended to the Korean validation dataset and demonstrated ethnic generalizability in the Caucasian validation cohort. Overall, the development of a deep learning-based classifier integrating cfDNA fragmentomic and genomic features in this study highlights the potential for accurate lung cancer detection across diverse populations.Significance: Evaluating fragment-based features and genomic coverage in cell-free DNA offers an accurate lung cancer screening method, promising improvements in early cancer detection and addressing challenges associated with current screening methods. | In a new study, researchers developed a blood test to help find lung cancer earlier, which is key to better survival rates. The test works by looking at tiny bits of DNA in the blood, called cell-free DNA. These DNA pieces have unique patterns in people with lung cancer. The researchers trained a computer program to find these specific patterns, including the size and end motifs of the DNA fragments. The new test combines these patterns with other genetic information to make a very accurate prediction. The study was tested on a large group of people from Korea and a smaller group of Caucasian individuals, and it worked well in both groups. This suggests the test could be useful for many different people, offering a promising new tool for cancer screening and helping to catch lung cancer at its earliest stages. | This research is focused on finding new ways to detect lung cancer early, which is crucial for helping patients live longer. The study's authors are exploring a new method using a simple blood test called a liquid biopsy. This test looks at small pieces of DNA in the blood, called cell-free DNA, to find signs of cancer. By using a sophisticated computer program, they were able to combine different DNA characteristics, such as the size and pattern of the DNA pieces, to create a highly accurate test. The new test worked well in a large group of people from different backgrounds, showing its potential to be a reliable screening tool. The findings suggest this approach could one day be a valuable addition to current screening methods, making it easier to find lung cancer early and ultimately helping to save lives. | This research explores a new method for detecting lung cancer early on, which is key to improving survival. The study suggests using a simple blood test, called a liquid biopsy, to analyze tiny DNA fragments in the blood. Researchers developed a computer program that looks at the patterns and sizes of these DNA fragments, as well as where they come from in the genome, to identify signs of lung cancer. The program was trained and tested using samples from thousands of people in Korea and a separate group of Caucasian individuals. The results show that this new method is highly accurate and works well across different ethnic groups. This exciting new approach has the potential to make lung cancer screening more effective and accessible than current methods. |
| CancerRes | 3 | 10.1158/0008-5472.CAN-24-1521 | DeepSeek | Early detection of lung cancer is important for improving patient survival rates. Liquid biopsy using whole-genome sequencing of cell-free DNA (cfDNA) offers a promising avenue for lung cancer screening, providing a potential alternative or complementary approach to current screening modalities. Here, we aimed to develop and validate an approach by integrating fragment and genomic features of cfDNA to enhance lung cancer detection accuracy across diverse populations. Deep learning-based classifiers were trained using comprehensive cfDNA fragmentomic features from participants in multi-institutional studies, including a Korean discovery dataset (218 patients with lung cancer and 2,559 controls), a Korean validation dataset (111 patients with lung cancer and 1,136 controls), and an independent Caucasian validation cohort (50 patients with lung cancer and 50 controls). In the discovery dataset, classifiers using fragment end motif by size, a feature that captures both fragment end motif and size profiles, outperformed standalone fragment end motif and fragment size classifiers, achieving an area under the curve (AUC) of 0.917. The ensemble classifier integrating fragment end motif by size and genomic coverage achieved an improved performance, with an AUC of 0.937. This performance extended to the Korean validation dataset and demonstrated ethnic generalizability in the Caucasian validation cohort. Overall, the development of a deep learning-based classifier integrating cfDNA fragmentomic and genomic features in this study highlights the potential for accurate lung cancer detection across diverse populations.Significance: Evaluating fragment-based features and genomic coverage in cell-free DNA offers an accurate lung cancer screening method, promising improvements in early cancer detection and addressing challenges associated with current screening methods. | Detecting lung cancer early can save lives, but current screening methods aren’t perfect. This study tested a new blood test that looks at tiny pieces of DNA released by tumors into the bloodstream. By analyzing the size and patterns of these DNA fragments, along with other genetic clues, researchers trained a computer program to spot lung cancer more accurately. The method was tested in over 3,000 people, including different ethnic groups, and showed strong results—catching cancer with over 90% accuracy. This approach could make early lung cancer detection easier and more reliable, helping doctors diagnose the disease sooner and improve patient outcomes. | This study explores a new blood test that could help detect lung cancer earlier by analyzing tiny pieces of DNA released by tumors into the bloodstream. The researchers combined two key features of these DNA fragments—their size and unique end patterns—along with genetic data to improve detection accuracy. They tested this method in over 3,000 people, including diverse groups from Korea and Europe, and found it worked well across different populations. The results suggest this approach could offer a simpler, more reliable way to screen for lung cancer compared to current methods, potentially saving lives through earlier diagnosis. This breakthrough may lead to better early detection tools, especially for high-risk individuals. | This study aimed to improve early lung cancer detection by analyzing tiny pieces of DNA in the blood, called cell-free DNA. The researchers combined two key features of this DNA—its size and the patterns at its ends—with other genetic data to create a more accurate test. Using a computer program trained on data from over 3,000 people (including both healthy individuals and lung cancer patients), they developed a method that could spot cancer with high accuracy. The test worked well in different groups, including Korean and Caucasian participants, suggesting it could help detect lung cancer earlier in diverse populations. This approach could offer a simpler, less invasive alternative to current screening methods, potentially saving lives by catching cancer sooner. |
| CancerRes | 3 | 10.1158/0008-5472.CAN-24-1522 | Grok | Early detection of lung cancer is important for improving patient survival rates. Liquid biopsy using whole-genome sequencing of cell-free DNA (cfDNA) offers a promising avenue for lung cancer screening, providing a potential alternative or complementary approach to current screening modalities. Here, we aimed to develop and validate an approach by integrating fragment and genomic features of cfDNA to enhance lung cancer detection accuracy across diverse populations. Deep learning-based classifiers were trained using comprehensive cfDNA fragmentomic features from participants in multi-institutional studies, including a Korean discovery dataset (218 patients with lung cancer and 2,559 controls), a Korean validation dataset (111 patients with lung cancer and 1,136 controls), and an independent Caucasian validation cohort (50 patients with lung cancer and 50 controls). In the discovery dataset, classifiers using fragment end motif by size, a feature that captures both fragment end motif and size profiles, outperformed standalone fragment end motif and fragment size classifiers, achieving an area under the curve (AUC) of 0.917. The ensemble classifier integrating fragment end motif by size and genomic coverage achieved an improved performance, with an AUC of 0.937. This performance extended to the Korean validation dataset and demonstrated ethnic generalizability in the Caucasian validation cohort. Overall, the development of a deep learning-based classifier integrating cfDNA fragmentomic and genomic features in this study highlights the potential for accurate lung cancer detection across diverse populations.Significance: Evaluating fragment-based features and genomic coverage in cell-free DNA offers an accurate lung cancer screening method, promising improvements in early cancer detection and addressing challenges associated with current screening methods. | Lung cancer is often deadly because it's found too late, so finding it early can save lives. This study suggests using a simple blood test to spot lung cancer by looking at tiny bits of DNA floating in the blood, which could be easier and better than current scans. The researchers aimed to create a smart computer program that combines patterns in the size and ends of these DNA bits with other genetic info to detect cancer more accurately in people from different backgrounds. They trained the program on data from over 3,000 Koreans and tested it on more groups, including white people. The program worked well, correctly spotting cancer about 94% of the time. This could help doctors catch lung cancer sooner, leading to better treatments and inspiring more research on blood tests for early cancer detection worldwide. | Lung cancer is often deadly if found late, so catching it early can save lives. Current tests have limits, but using blood samples to check tiny bits of DNA floating in the blood might help. Researchers wanted to create a better way to spot lung cancer by combining details about these DNA pieces' ends, sizes, and where they come from in the genome. They trained smart computer programs on data from hundreds of people with and without cancer from Korea and a group from Europe and America. The best program spotted cancer with high accuracy, up to 94% in tests. This could lead to simpler, more reliable blood tests for early lung cancer screening worldwide, helping doctors find and treat it sooner for more people. | Lung cancer is easier to treat when found early, but current tests have limits. Researchers suggest using a simple blood test that looks at tiny pieces of DNA floating in the blood to spot the cancer sooner. They aimed to create a smart computer program that combines details about the ends and sizes of these DNA pieces with other gene information to detect lung cancer more accurately in people from different backgrounds. In tests with Korean and white groups, this method worked well, correctly identifying cancer in most cases without many mistakes. These findings could help scientists build better screening tools, making early detection easier and saving more lives around the world. |
| CancerRes | 4 | 10.1158/0008-5472.CAN-25-0988 | Open AI | Therapy resistance is a significant cause of death in patients treated with targeted cancer therapy in diverse oncogene-driven cancers. A better understanding of resistance mechanisms can lay the foundation for improving existing and developing new therapies. A recent elegant study published in Nature Chemical Biology sheds light on a new resistance mechanism. The authors define a novel role for ARAF, a member of the RAF protein family (ARAF, BRAF, and CRAF), that is distinct from its previously understood role as a RAS effector and MEK protein kinase in the MAPK pathway. They describe how ARAF sequesters active RAS at the plasma membrane in phase-separated condensates to sustain signaling and prevent inactivation by the RAS GTPase-activating protein neurofibromin 1. This study underscores emerging roles for biomolecular condensates in cancer and highlights important implications for disrupting protein condensates to address treatment resistance to RAS (and RAS pathway)-targeted therapies. The study also illuminates evolutionary functional distinction between the RAF proteins and indicates unique biology for ARAF in normal physiology and disease. | Cancer treatments that target specific genes often stop working because cancer cells find ways to resist therapy. This study investigates one of these resistance mechanisms and finds that a protein called ARAF can protect cancer cells from treatment. ARAF does this by gathering active RAS proteins into tiny clusters at the cell surface, keeping RAS active and preventing it from being turned off. This activity allows cancer cells to continue growing even when drugs try to block RAS signals. Understanding this process reveals that these protein clusters, known as condensates, play a key role in therapy resistance. By targeting these condensates, new treatments may be developed to overcome resistance and improve cancer therapy. The findings also show that ARAF works differently from its related proteins, highlighting new insights into how cells normally function and how cancer cells exploit these processes. | Some cancers are driven by a protein called RAS, and treatments targeting this protein often stop working because cancer cells find ways to resist them. This study discovered a new reason for this resistance. It shows that a protein called ARAF can gather active RAS into tiny clusters inside cells, keeping RAS turned on and protecting it from normal “off” signals. This clustering helps cancer cells survive even when treatments try to block RAS activity. Understanding this process gives scientists a new target for therapy: breaking apart these clusters could make existing drugs work better. The findings also reveal that ARAF behaves differently from related proteins, which could help researchers design more precise treatments. Overall, this study points to new ways to overcome treatment resistance and improve outcomes for patients with RAS-driven cancers. | Cancer treatments that target specific genes often stop working because cancer cells find ways to resist them, which can lead to treatment failure. This study explores a new way cancer cells survive targeted therapy by focusing on a protein called ARAF. Unlike other similar proteins, ARAF can gather active RAS proteins into tiny clusters at the cell surface, keeping cancer signals turned on and protecting them from natural “off switches.” By revealing this hidden role, the research shows that these protein clusters help cancer resist treatment. Understanding this process could guide scientists to develop new strategies that disrupt these clusters, making targeted therapies more effective. This discovery also highlights that ARAF behaves differently from related proteins, offering new insights into both normal cell function and cancer development. |
| CancerRes | 4 | 10.1158/0008-5472.CAN-25-0989 | Claude | Therapy resistance is a significant cause of death in patients treated with targeted cancer therapy in diverse oncogene-driven cancers. A better understanding of resistance mechanisms can lay the foundation for improving existing and developing new therapies. A recent elegant study published in Nature Chemical Biology sheds light on a new resistance mechanism. The authors define a novel role for ARAF, a member of the RAF protein family (ARAF, BRAF, and CRAF), that is distinct from its previously understood role as a RAS effector and MEK protein kinase in the MAPK pathway. They describe how ARAF sequesters active RAS at the plasma membrane in phase-separated condensates to sustain signaling and prevent inactivation by the RAS GTPase-activating protein neurofibromin 1. This study underscores emerging roles for biomolecular condensates in cancer and highlights important implications for disrupting protein condensates to address treatment resistance to RAS (and RAS pathway)-targeted therapies. The study also illuminates evolutionary functional distinction between the RAF proteins and indicates unique biology for ARAF in normal physiology and disease. | Cancer drugs that target specific proteins often stop working over time, causing many patients to die from their disease. Scientists need to understand why these treatments fail so they can create better therapies. A new study reveals an unexpected way that cancer cells become resistant to treatment. The researchers found that a protein called ARAF acts differently than scientists previously thought. Instead of just passing along growth signals like other similar proteins, ARAF creates special clusters at the cell's outer edge that trap and protect another important cancer protein called RAS. These clusters keep RAS active and shield it from proteins that would normally turn it off. This discovery shows how tiny protein clusters inside cells can help cancer survive treatment. Understanding this process could help doctors develop new ways to overcome drug resistance in cancer patients. The findings also reveal that ARAF has unique jobs compared to related proteins, which opens new research directions for cancer treatment. | Cancer treatments often stop working over time, causing many patient deaths. Scientists need to understand why cancer drugs fail so they can make better treatments. New research shows how a protein called ARAF helps cancer cells fight back against drugs. ARAF is part of a family of proteins that usually help cells grow. But this study found ARAF does something different - it grabs onto another important protein called RAS and keeps it active by forming tiny clusters on the cell surface. These clusters protect RAS from being turned off by the body's natural controls. This discovery helps explain why some cancer treatments stop working. It also shows that breaking up these protein clusters might be a new way to treat cancer. The findings reveal that ARAF works differently from other similar proteins, giving scientists new targets for developing cancer drugs that could overcome treatment resistance. | Cancer treatments that target specific proteins often stop working over time, causing many patients to die from their disease. Scientists needed to understand why these treatments fail so they could create better medicines. Researchers discovered a new way that cancer cells become resistant to treatment by studying a protein called ARAF. They found that ARAF acts like a protective shield around another important cancer protein called RAS. ARAF creates special clusters at the cell's outer edge that keep RAS active and prevent other proteins from turning it off. This discovery shows how tiny protein clusters inside cells can help cancer survive treatment. The findings could lead to new medicines that break apart these protective clusters, making current cancer treatments work better. This research also reveals that ARAF works differently than other similar proteins, giving scientists new targets for fighting cancer. Understanding these protein clusters opens up fresh approaches to overcome treatment resistance in many types of cancer. |
| CancerRes | 4 | 10.1158/0008-5472.CAN-25-0990 | Gemini | Therapy resistance is a significant cause of death in patients treated with targeted cancer therapy in diverse oncogene-driven cancers. A better understanding of resistance mechanisms can lay the foundation for improving existing and developing new therapies. A recent elegant study published in Nature Chemical Biology sheds light on a new resistance mechanism. The authors define a novel role for ARAF, a member of the RAF protein family (ARAF, BRAF, and CRAF), that is distinct from its previously understood role as a RAS effector and MEK protein kinase in the MAPK pathway. They describe how ARAF sequesters active RAS at the plasma membrane in phase-separated condensates to sustain signaling and prevent inactivation by the RAS GTPase-activating protein neurofibromin 1. This study underscores emerging roles for biomolecular condensates in cancer and highlights important implications for disrupting protein condensates to address treatment resistance to RAS (and RAS pathway)-targeted therapies. The study also illuminates evolutionary functional distinction between the RAF proteins and indicates unique biology for ARAF in normal physiology and disease. | Therapy resistance is a major problem in treating cancer, causing many deaths. To improve existing treatments and develop new ones, scientists need to better understand how cancer cells become resistant. A recent study reveals a new way that cancer cells resist treatment. The researchers found that a protein called ARAF helps keep another protein, called RAS, active by trapping it in small droplets inside the cell. These droplets protect RAS from a protein that would normally turn it off. By keeping RAS active, cancer cells can continue to grow even when treated with drugs designed to stop them. This discovery shows that targeting these droplets could be a new way to overcome treatment resistance. | Cancer drugs that target a protein called RAS sometimes stop working, which is a major problem for patients. This research looks at a new reason for this drug resistance. Scientists discovered that another protein, ARAF, can trap the active RAS protein in small, liquid-like droplets called condensates. These droplets form on the cell's outer layer and keep the RAS protein active, preventing it from being turned off naturally. This sustained activity allows the cancer to continue growing even with treatment. The findings suggest that targeting and breaking apart these specific condensates could be a new way to overcome drug resistance and improve cancer therapies that target the RAS pathway. This discovery also helps us better understand the unique roles of different proteins in this family. | Therapy resistance poses a major challenge in treating cancer, often leading to poor patient outcomes. This research explores a new reason why certain cancer treatments stop working. The study reveals that a specific protein called ARAF helps cancer cells resist treatment by trapping another protein, called RAS, in tiny droplets inside the cell. These droplets, or condensates, keep the RAS protein active for longer, allowing it to continuously send growth signals that fuel the cancer. By understanding how these droplets contribute to drug resistance, scientists can explore new strategies to disrupt them. This could pave the way for developing better therapies for RAS-driven cancers and improve how we treat patients in the future. |
| CancerRes | 4 | 10.1158/0008-5472.CAN-25-0991 | DeepSeek | Therapy resistance is a significant cause of death in patients treated with targeted cancer therapy in diverse oncogene-driven cancers. A better understanding of resistance mechanisms can lay the foundation for improving existing and developing new therapies. A recent elegant study published in Nature Chemical Biology sheds light on a new resistance mechanism. The authors define a novel role for ARAF, a member of the RAF protein family (ARAF, BRAF, and CRAF), that is distinct from its previously understood role as a RAS effector and MEK protein kinase in the MAPK pathway. They describe how ARAF sequesters active RAS at the plasma membrane in phase-separated condensates to sustain signaling and prevent inactivation by the RAS GTPase-activating protein neurofibromin 1. This study underscores emerging roles for biomolecular condensates in cancer and highlights important implications for disrupting protein condensates to address treatment resistance to RAS (and RAS pathway)-targeted therapies. The study also illuminates evolutionary functional distinction between the RAF proteins and indicates unique biology for ARAF in normal physiology and disease. | Cancer treatments often stop working because tumors become resistant to drugs. Researchers have discovered a new reason why this happens: tiny clumps of proteins called "condensates" help keep cancer signals active. A protein named ARAF traps another cancer-causing protein, RAS, inside these clumps, preventing it from being turned off. This keeps cancer cells growing even when treated with targeted drugs. The findings suggest that breaking apart these protein clumps could make treatments work better, especially for cancers driven by RAS. This study also shows that ARAF works differently than other similar proteins, which could lead to new ways to fight cancer. Understanding this process may help scientists develop better drugs to overcome resistance and save lives. | This study explores why some cancers stop responding to targeted treatments. Researchers discovered that a protein called ARAF, which is part of a group linked to cancer growth, plays a surprising new role. Instead of just helping cancer cells grow, ARAF traps an active cancer-driving protein (RAS) in tiny clumps called condensates at the cell’s surface. This keeps RAS turned "on," allowing cancer cells to resist treatment. The findings suggest that breaking up these condensates could help make existing drugs work better or lead to new therapies. The study also reveals that ARAF behaves differently than related proteins, which could help scientists develop more precise treatments in the future. | This study explores why some cancers stop responding to targeted treatments. Researchers discovered that a protein called ARAF, which normally helps control cell growth, can form tiny clumps (called condensates) that trap another cancer-driving protein, RAS, at the cell membrane. This keeps RAS active, allowing cancer cells to resist treatment. The findings reveal a new way cancers evade drugs and suggest that breaking up these clumps could make treatments work better. The study also shows that ARAF behaves differently than similar proteins, which could help scientists design smarter drugs in the future. This discovery opens doors for improving therapies for cancers driven by RAS and related proteins. |
| CancerRes | 4 | 10.1158/0008-5472.CAN-25-0992 | Grok | Therapy resistance is a significant cause of death in patients treated with targeted cancer therapy in diverse oncogene-driven cancers. A better understanding of resistance mechanisms can lay the foundation for improving existing and developing new therapies. A recent elegant study published in Nature Chemical Biology sheds light on a new resistance mechanism. The authors define a novel role for ARAF, a member of the RAF protein family (ARAF, BRAF, and CRAF), that is distinct from its previously understood role as a RAS effector and MEK protein kinase in the MAPK pathway. They describe how ARAF sequesters active RAS at the plasma membrane in phase-separated condensates to sustain signaling and prevent inactivation by the RAS GTPase-activating protein neurofibromin 1. This study underscores emerging roles for biomolecular condensates in cancer and highlights important implications for disrupting protein condensates to address treatment resistance to RAS (and RAS pathway)-targeted therapies. The study also illuminates evolutionary functional distinction between the RAF proteins and indicates unique biology for ARAF in normal physiology and disease. | Cancer treatments that target specific genes often fail because tumors become resistant, leading to patient deaths. This study explores a new reason for this resistance in cancers driven by faulty RAS proteins. Researchers found that another protein, ARAF, traps active RAS in tiny clusters at the cell surface. These clusters help RAS keep sending signals that promote cancer growth and block a natural off-switch protein. By understanding this, scientists hope to create better drugs that break up these clusters. This could improve treatments for RAS-related cancers, reduce resistance, and save more lives by opening doors to new therapies for the public. | Many people die from cancer because treatments that target specific cancer-causing genes stop working over time. This study looks at why that happens and suggests a new way to fight it. Researchers found that a protein called ARAF helps keep another protein, RAS, active by trapping it in tiny droplets at the cell's edge. These droplets protect RAS from being turned off, allowing cancer signals to continue. The goal is to understand this process better to create improved drugs that break up these droplets. This could help scientists develop better ways to treat cancers driven by RAS problems and reveal how similar proteins work differently in health and disease. It opens doors for new ideas in stopping drug resistance. | Cancer treatments that target specific genes often stop working because tumors become resistant, leading to many deaths. This research explores why resistance happens in cancers driven by faulty RAS proteins, which send constant growth signals. Scientists discovered a new job for the ARAF protein: it traps active RAS in small droplets at the cell's edge, keeping signals strong and blocking a natural off-switch called neurofibromin 1. By studying this, the team aims to uncover ways to break these droplets and improve therapies. The findings could help doctors create better drugs to fight resistance, saving lives, and teach us more about how similar proteins work differently in health and disease. |
| CancerRes | 5 | 10.1158/0008-5472.CAN-24-2290 | Open AI | Non-coding RNA transcribed from active enhancers, known as enhancer RNA (eRNA), is a critical element in gene regulation with a highly specific expression pattern in the regulatory networks of tumor-infiltrating cells. Therefore, eRNA signatures could potentially be applied to represent antitumor immune cells and to improve cancer immunotherapy. In this study, we identified thousands of eRNAs that were significantly correlated with infiltrating immune cell abundance in more than 10,000 patient samples across a variety of cancer types. The expression of these eRNAs was mediated by transcription factors with high expression in antitumor immune cells, as identified through single-cell assays. An eRNA immunotherapy signature (eRIS) developed using the antitumor eRNAs was highly associated with the objective response rate of immunotherapy and was elevated in patients who benefited from immune checkpoint blockade treatment. In comparison with a signature based on protein-coding genes, the eRIS was more effective in predicting the response to immunotherapy. Integration of the eRIS with pharmacogenomic data revealed hundreds of anticancer drugs that have the potential to enhance immunotherapy efficacy. Finally, treatment of a mouse model of IDH-mutant glioma with the histone deacetylase inhibitor vorinostat improved the effects of anti-PD-1 immunotherapy through increased abundance of infiltrating immune cells. Taken together, this study developed an eRIS with demonstrated efficacy in predicting immunotherapy response and used the eRIS to identify a series of effective combination drugs, thus highlighting the clinical utility of the eRIS in immunotherapy enhancement.Significance: An eRNA immunotherapy signature developed using antitumor eRNAs in tumor-infiltrating immune cells improves the prediction of response to immunotherapy and identifies a series of effective drug combinations to enhance immunotherapy efficacy. | This study explored a type of genetic material called enhancer RNA, which helps control how genes are turned on in immune cells that fight cancer. The researchers analyzed thousands of patient samples and found specific enhancer RNAs linked to the presence of these immune cells in tumors. Using this information, they created a new “enhancer RNA signature” that can predict which patients are most likely to benefit from cancer immunotherapy. This signature was more accurate than methods based on traditional gene analysis. By combining this signature with drug data, the researchers also identified existing medications that could work alongside immunotherapy to make it more effective. In experiments with mice, one such drug boosted immune cell activity and improved treatment results. Overall, this work provides a new tool to guide cancer treatment and suggests ways to make immunotherapy stronger and more personalized for patients. | This study explored a special type of genetic material called enhancer RNA, which helps control gene activity in immune cells that fight cancer. The researchers analyzed thousands of patient samples and found specific enhancer RNAs linked to the presence of these cancer-fighting cells. Using this information, they created a signature that can predict how well patients respond to immunotherapy, a treatment that helps the immune system attack tumors. This signature was better at predicting treatment success than traditional methods based on regular genes. The researchers also discovered dozens of existing drugs that could work alongside immunotherapy to make it more effective. In experiments with mice, one of these drugs improved the benefits of immunotherapy by boosting immune cells in tumors. Overall, this work provides a new way to guide cancer treatment, helping doctors select therapies that are more likely to work and identifying combinations that could improve patient outcomes. | This study explores small molecules called enhancer RNAs, which help control gene activity in immune cells that enter tumors. By examining samples from over 10,000 cancer patients, the researchers found specific enhancer RNAs that mark active antitumor immune cells. Using these molecules, they developed a tool, called an enhancer RNA immunotherapy signature, that can predict which patients are most likely to respond to immunotherapy. Compared to previous methods, this tool more accurately identifies patients who will benefit from treatment. The researchers also used it to find existing drugs that could boost immunotherapy effects. In experiments with mice, one of these drugs improved the success of immune-based therapy. Overall, this work offers a new way to guide cancer treatment, helping doctors select the best therapies and combinations to improve patient outcomes. |
| CancerRes | 5 | 10.1158/0008-5472.CAN-24-2291 | Claude | Non-coding RNA transcribed from active enhancers, known as enhancer RNA (eRNA), is a critical element in gene regulation with a highly specific expression pattern in the regulatory networks of tumor-infiltrating cells. Therefore, eRNA signatures could potentially be applied to represent antitumor immune cells and to improve cancer immunotherapy. In this study, we identified thousands of eRNAs that were significantly correlated with infiltrating immune cell abundance in more than 10,000 patient samples across a variety of cancer types. The expression of these eRNAs was mediated by transcription factors with high expression in antitumor immune cells, as identified through single-cell assays. An eRNA immunotherapy signature (eRIS) developed using the antitumor eRNAs was highly associated with the objective response rate of immunotherapy and was elevated in patients who benefited from immune checkpoint blockade treatment. In comparison with a signature based on protein-coding genes, the eRIS was more effective in predicting the response to immunotherapy. Integration of the eRIS with pharmacogenomic data revealed hundreds of anticancer drugs that have the potential to enhance immunotherapy efficacy. Finally, treatment of a mouse model of IDH-mutant glioma with the histone deacetylase inhibitor vorinostat improved the effects of anti-PD-1 immunotherapy through increased abundance of infiltrating immune cells. Taken together, this study developed an eRIS with demonstrated efficacy in predicting immunotherapy response and used the eRIS to identify a series of effective combination drugs, thus highlighting the clinical utility of the eRIS in immunotherapy enhancement.Significance: An eRNA immunotherapy signature developed using antitumor eRNAs in tumor-infiltrating immune cells improves the prediction of response to immunotherapy and identifies a series of effective drug combinations to enhance immunotherapy efficacy. | Cancer immunotherapy helps the body's immune system fight cancer, but it doesn't work for everyone. Scientists wanted to find better ways to predict which patients would benefit from this treatment and discover drugs that could make it work better. They studied special molecules called enhancer RNAs that are found in immune cells that fight tumors. By looking at over 10,000 cancer patients, they created a signature pattern of these molecules that could predict treatment success better than existing methods. This signature showed which patients were more likely to respond to immunotherapy. The researchers also used this pattern to identify hundreds of existing cancer drugs that might boost immunotherapy effectiveness. When they tested one of these drugs in mice with brain cancer, it made the immunotherapy work much better by bringing more cancer-fighting immune cells to the tumor. This discovery could help doctors choose the best treatments for cancer patients and improve success rates. | Cancer treatments that boost the immune system to fight tumors, called immunotherapy, work well for some patients but not others. Scientists wanted to find better ways to predict who would benefit from these treatments and discover new drug combinations to make them more effective. They focused on special molecules called enhancer RNAs that help control genes in immune cells that infiltrate tumors. The researchers studied over 10,000 cancer patients and found thousands of these enhancer RNAs that were linked to immune cells fighting cancer. They created a signature using these molecules that could predict which patients would respond well to immunotherapy better than existing methods. Using this signature, they identified hundreds of potential drugs that could be combined with immunotherapy to make it work better. When they tested one of these drugs in mice with brain tumors, it significantly improved the effectiveness of immunotherapy treatment. This research provides doctors with a new tool to predict treatment success and offers promising drug combinations that could help more cancer patients benefit from immunotherapy | Scientists have developed a new way to predict which cancer patients will respond best to immunotherapy treatments. They discovered special RNA molecules called enhancer RNAs that act like switches to control genes in immune cells that fight tumors. By studying over 10,000 cancer patients, researchers found thousands of these RNA switches that relate to how many tumor-fighting immune cells are present in different cancers. They created a scoring system called an enhancer RNA immunotherapy signature that works better than current methods at predicting whether immunotherapy will help a patient. This scoring system also helped identify hundreds of existing drugs that could be combined with immunotherapy to make treatments more effective. When they tested one of these drug combinations in mice with brain cancer, it significantly improved the immunotherapy results by bringing more immune cells into tumors. This research provides doctors with a better tool to identify which patients will benefit from immunotherapy and offers new combination treatments that could help more cancer patients respond to these important immune-based therapies. |
| CancerRes | 5 | 10.1158/0008-5472.CAN-24-2292 | Gemini | Non-coding RNA transcribed from active enhancers, known as enhancer RNA (eRNA), is a critical element in gene regulation with a highly specific expression pattern in the regulatory networks of tumor-infiltrating cells. Therefore, eRNA signatures could potentially be applied to represent antitumor immune cells and to improve cancer immunotherapy. In this study, we identified thousands of eRNAs that were significantly correlated with infiltrating immune cell abundance in more than 10,000 patient samples across a variety of cancer types. The expression of these eRNAs was mediated by transcription factors with high expression in antitumor immune cells, as identified through single-cell assays. An eRNA immunotherapy signature (eRIS) developed using the antitumor eRNAs was highly associated with the objective response rate of immunotherapy and was elevated in patients who benefited from immune checkpoint blockade treatment. In comparison with a signature based on protein-coding genes, the eRIS was more effective in predicting the response to immunotherapy. Integration of the eRIS with pharmacogenomic data revealed hundreds of anticancer drugs that have the potential to enhance immunotherapy efficacy. Finally, treatment of a mouse model of IDH-mutant glioma with the histone deacetylase inhibitor vorinostat improved the effects of anti-PD-1 immunotherapy through increased abundance of infiltrating immune cells. Taken together, this study developed an eRIS with demonstrated efficacy in predicting immunotherapy response and used the eRIS to identify a series of effective combination drugs, thus highlighting the clinical utility of the eRIS in immunotherapy enhancement.Significance: An eRNA immunotherapy signature developed using antitumor eRNAs in tumor-infiltrating immune cells improves the prediction of response to immunotherapy and identifies a series of effective drug combinations to enhance immunotherapy efficacy. | This research explores how specific non-coding RNAs, called enhancer RNAs, can be used to improve cancer treatment. We looked at thousands of patient samples to identify these special RNAs, which are produced by immune cells that have entered tumors. We developed a unique signature based on these RNAs that proved to be better at predicting which patients would respond well to immunotherapy than existing methods. By combining this new signature with information about various drugs, we were able to pinpoint many medications that could potentially make immunotherapy more effective. Our study successfully showed that a drug used in a mouse model improved the effects of immunotherapy, providing a promising new way to enhance cancer treatment for patients. | This research explores how specific small pieces of genetic material, called enhancer RNA, can help us better understand and fight cancer. These enhancer RNAs act like switches, turning on important genes in immune cells that have entered a tumor. By studying these genetic switches in thousands of patient samples, we developed a new tool to predict whether a patient will respond well to a type of cancer treatment called immunotherapy. This new tool was even better at making predictions than existing methods. We also used this tool to discover new drug combinations that could make immunotherapy more effective. In a mouse study, we successfully used one of these drugs to improve the outcome of immunotherapy for brain cancer. Ultimately, this work provides a new way to predict treatment success and to find better ways to combine treatments for cancer patients. | This research explores how specific non-coding RNAs, called enhancer RNAs, can help us understand the immune cells fighting cancer. We studied thousands of patient samples to identify a unique signature, or pattern, of these enhancer RNAs that is linked to a strong immune response against tumors. We developed a new tool, called the eRIS, which proved to be better than existing methods at predicting which patients will benefit from immunotherapy. By combining our findings with information on different drugs, we found promising drug combinations that could make immunotherapy work better. We even tested one of these combinations in a mouse model of brain cancer, showing that adding a specific drug improved the effectiveness of immunotherapy. Ultimately, this work offers a new way to predict immunotherapy success and find new treatments to boost its power for cancer patients. |
| CancerRes | 5 | 10.1158/0008-5472.CAN-24-2293 | DeepSeek | Non-coding RNA transcribed from active enhancers, known as enhancer RNA (eRNA), is a critical element in gene regulation with a highly specific expression pattern in the regulatory networks of tumor-infiltrating cells. Therefore, eRNA signatures could potentially be applied to represent antitumor immune cells and to improve cancer immunotherapy. In this study, we identified thousands of eRNAs that were significantly correlated with infiltrating immune cell abundance in more than 10,000 patient samples across a variety of cancer types. The expression of these eRNAs was mediated by transcription factors with high expression in antitumor immune cells, as identified through single-cell assays. An eRNA immunotherapy signature (eRIS) developed using the antitumor eRNAs was highly associated with the objective response rate of immunotherapy and was elevated in patients who benefited from immune checkpoint blockade treatment. In comparison with a signature based on protein-coding genes, the eRIS was more effective in predicting the response to immunotherapy. Integration of the eRIS with pharmacogenomic data revealed hundreds of anticancer drugs that have the potential to enhance immunotherapy efficacy. Finally, treatment of a mouse model of IDH-mutant glioma with the histone deacetylase inhibitor vorinostat improved the effects of anti-PD-1 immunotherapy through increased abundance of infiltrating immune cells. Taken together, this study developed an eRIS with demonstrated efficacy in predicting immunotherapy response and used the eRIS to identify a series of effective combination drugs, thus highlighting the clinical utility of the eRIS in immunotherapy enhancement.Significance: An eRNA immunotherapy signature developed using antitumor eRNAs in tumor-infiltrating immune cells improves the prediction of response to immunotherapy and identifies a series of effective drug combinations to enhance immunotherapy efficacy. | This study explores how tiny bits of genetic material called enhancer RNAs (eRNAs) can help predict whether cancer immunotherapy will work. The researchers analyzed over 10,000 patient samples and found that certain eRNAs are linked to strong immune responses against tumors. They created a new tool called eRIS (based on these eRNAs) that predicts immunotherapy success better than older methods. The team also discovered hundreds of drugs—like the cancer treatment vorinostat—that could boost immunotherapy’s power, and they proved this works in mice with brain tumors. These findings could help doctors choose the best treatments for cancer patients and develop smarter drug combos to fight tumors more effectively. | This study explores how certain RNA molecules, called enhancer RNAs (eRNAs), can help predict whether cancer immunotherapy will work. These eRNAs are linked to immune cells that fight tumors. By analyzing over 10,000 patient samples, researchers found that an eRNA-based signature (eRIS) could better predict immunotherapy success than traditional methods. They also discovered drugs—like vorinostat—that may boost immunotherapy by increasing immune cell activity in tumors. In mice with brain cancer, combining vorinostat with immunotherapy improved treatment results. These findings could help doctors choose the best treatments for patients and develop new drug combinations to make immunotherapy more effective. This research offers hope for better cancer care by personalizing and improving immune-based therapies. | This study explores how a special type of RNA, called enhancer RNA (eRNA), can help predict whether cancer immunotherapy will work. By analyzing over 10,000 patient samples, researchers found that certain eRNAs are linked to immune cells that fight tumors. They created an eRNA-based signature (eRIS) that better predicts immunotherapy success than traditional methods. The team also discovered hundreds of drugs—like the cancer drug vorinostat—that could boost immunotherapy by attracting more immune cells to tumors. In mice with brain cancer, combining vorinostat with immunotherapy improved treatment results. These findings could help doctors choose the best treatments for cancer patients and develop new drug combinations to make immunotherapy more effective. |
| CancerRes | 5 | 10.1158/0008-5472.CAN-24-2294 | Grok | Non-coding RNA transcribed from active enhancers, known as enhancer RNA (eRNA), is a critical element in gene regulation with a highly specific expression pattern in the regulatory networks of tumor-infiltrating cells. Therefore, eRNA signatures could potentially be applied to represent antitumor immune cells and to improve cancer immunotherapy. In this study, we identified thousands of eRNAs that were significantly correlated with infiltrating immune cell abundance in more than 10,000 patient samples across a variety of cancer types. The expression of these eRNAs was mediated by transcription factors with high expression in antitumor immune cells, as identified through single-cell assays. An eRNA immunotherapy signature (eRIS) developed using the antitumor eRNAs was highly associated with the objective response rate of immunotherapy and was elevated in patients who benefited from immune checkpoint blockade treatment. In comparison with a signature based on protein-coding genes, the eRIS was more effective in predicting the response to immunotherapy. Integration of the eRIS with pharmacogenomic data revealed hundreds of anticancer drugs that have the potential to enhance immunotherapy efficacy. Finally, treatment of a mouse model of IDH-mutant glioma with the histone deacetylase inhibitor vorinostat improved the effects of anti-PD-1 immunotherapy through increased abundance of infiltrating immune cells. Taken together, this study developed an eRIS with demonstrated efficacy in predicting immunotherapy response and used the eRIS to identify a series of effective combination drugs, thus highlighting the clinical utility of the eRIS in immunotherapy enhancement.Significance: An eRNA immunotherapy signature developed using antitumor eRNAs in tumor-infiltrating immune cells improves the prediction of response to immunotherapy and identifies a series of effective drug combinations to enhance immunotherapy efficacy. | Cancer cells can hide from the body's immune system, making treatments like immunotherapy less effective for some patients. Researchers wanted to find a better way to predict who will benefit from these treatments and how to make them work better. They studied special molecules called enhancer RNAs, which are active in immune cells that fight tumors. By looking at samples from over 10,000 patients with different cancers, they created a simple tool called an enhancer RNA immunotherapy signature. This tool predicts treatment success more accurately than older methods and helps identify hundreds of drugs that could boost immunotherapy. In mouse tests, one drug improved immune cell attacks when combined with immunotherapy. This discovery could help doctors choose the right treatments and save more lives by making cancer therapies stronger for everyone. | Cancer is hard to treat, but our body's immune system can help fight it with therapies like immune checkpoint blockers. However, not everyone responds well, so researchers studied special molecules called enhancer RNAs, which come from active gene switches in immune cells that attack tumors. They looked at samples from over 10,000 patients with different cancers to find these enhancer RNAs linked to strong immune responses. By creating a simple tool called an enhancer RNA immunotherapy signature, they could better predict who would benefit from these treatments compared to older methods. They also discovered hundreds of drugs that might work together with immunotherapy to make it more effective, and tests in mice showed one drug boosted immune cell attacks on brain tumors. This work could help doctors choose better treatments and inspire new ways to improve cancer care for everyone. | Cancer cells can hide from the body's immune system, making treatments like immunotherapy less effective for some patients. This study looked at special molecules called enhancer RNAs, which help control genes in immune cells that fight tumors. Researchers analyzed data from over 10,000 cancer patients to find enhancer RNAs linked to strong anti-tumor immune responses. They created a tool called the enhancer RNA immunotherapy signature to predict who will respond well to immune checkpoint blockers. Key findings showed this signature works better than ones based on regular genes and helped identify hundreds of drugs that could boost immunotherapy. In mouse tests, one drug improved treatment outcomes by increasing immune cells in tumors. For the public, this means better ways to personalize cancer care, potentially saving lives by combining therapies more effectively. |
| CancerRes | 1 | 10.1016/S1470-2045(25)00211-7 | Open AI | Background Oestrogen plus progestin hormone therapy is an established risk factor for breast cancer in postmenopausal women. We examined the less well-studied association between exogenous hormones and breast cancer in young women, who might use hormone therapy after gynaecological surgery or to relieve perimenopausal symptoms. Methods In this pooled cohort analysis, we investigated the relationship between exogenous hormones and breast cancer in young women using data from 10-13 prospective cohorts from North America, Europe, Asia, and Australia. The participating cohorts followed up women for incident breast cancer until age 55 years. We used cohort-stratified, multivariable-adjusted Cox proportional hazards regression to estimate hazard ratios (HRs) and 95% CI for associations of hormone therapy with incident young-onset breast cancer. We also estimated risk differences based on cumulative risk until age 55 years. Findings We included 459 476 women aged 16-54 years (mean 42 center dot 0 years [IQR 35 center dot 5-49 center dot 2]), of whom 8455 (2%) developed young-onset breast cancer (diagnosed before age 55 years; median follow-up 78 years [5 center dot 2-11 center dot 2]). Overall, 15% of participants reported using hormone therapy, with oestrogen plus progestin hormone therapy (6%) and unopposed oestrogen (5%) being the most common types. Cumulative risk of young-onset breast cancer was 41% in non-users. Hormone therapy of any type was not associated with incident young-onset breast cancer (HR 096 [95% CI 088 to 104]), but ever oestrogen hormone therapy use was inversely associated (086 [075 to 098]; risk difference-05% [-10 to-00]). The HR for ever oestrogen plus progestin hormone therapy and young-onset breast cancer was 110 (098 to 124), with positive associations observed for long-term use (118 [101 to 138] for >2 years) and use among women without hysterectomy or bilateral oophorectomy (115 [102 to 131]). Oestrogen hormone therapy and young-onset breast cancer association was similar for all breast cancer subtypes, but oestrogen plus progestin hormone therapy was more strongly associated with oestrogen receptor negative (144 [111 to 188]) and triple-negative disease (150 [102 to 220]) than with other subtypes. Interpretation Oestrogen hormone therapy use was inversely associated with young-onset breast cancer, and oestrogen plus progestin hormone therapy was associated with higher young-onset breast cancer incidence among women with intact uterus and ovaries. These findings largely parallel results from studies of hormone use and later-onset breast cancer and provide novel evidence for establishing clinical recommendations among younger women. | This study looked at whether taking hormone therapy affects the risk of developing breast cancer before age 55. Researchers combined data from over 459,000 women from around the world who were followed for several years. They compared women who had taken hormone therapy—either estrogen alone or estrogen plus progestin—with those who had never used it. They found that estrogen alone was linked to a slightly lower risk of breast cancer in younger women. In contrast, estrogen plus progestin was linked to a higher risk, especially when used for more than two years or in women who still had their uterus and ovaries. The increased risk was greater for certain aggressive cancer types. These results help doctors and patients weigh the benefits and risks of hormone therapy for younger women, especially when choosing between different types and durations of treatment. | This study looked at whether taking hormone therapy, which can be used after certain surgeries or to ease symptoms before menopause, affects the risk of breast cancer in younger women. Researchers combined data from over 450,000 women aged 16 to 54 from several countries and followed them until age 55. They found that using oestrogen alone was linked to a slightly lower risk of developing breast cancer at a younger age, while using oestrogen together with progestin for more than two years, especially in women with an intact uterus and ovaries, was linked to a higher risk. The increased risk was greater for more aggressive breast cancer types. These results give important new evidence to help guide doctors and patients when deciding whether to use hormone therapy in younger women. | This study looked at whether taking hormone therapy, often used for symptoms after gynecological surgery or during the years before menopause, is linked to breast cancer in younger women. We combined data from large studies around the world, following almost half a million women aged 16 to 54 for several years. We found that using estrogen alone was linked to a slightly lower chance of developing breast cancer before age 55. In contrast, using estrogen together with progestin for more than two years, especially in women who still had their uterus and ovaries, was linked to a higher risk. This higher risk was strongest for certain aggressive cancer types. These results give new insight into how hormone therapy affects younger women and may help doctors make safer treatment choices. |
| CancerRes | 1 | 10.1016/S1470-2045(25)00211-8 | Claude | Background Oestrogen plus progestin hormone therapy is an established risk factor for breast cancer in postmenopausal women. We examined the less well-studied association between exogenous hormones and breast cancer in young women, who might use hormone therapy after gynaecological surgery or to relieve perimenopausal symptoms. Methods In this pooled cohort analysis, we investigated the relationship between exogenous hormones and breast cancer in young women using data from 10-13 prospective cohorts from North America, Europe, Asia, and Australia. The participating cohorts followed up women for incident breast cancer until age 55 years. We used cohort-stratified, multivariable-adjusted Cox proportional hazards regression to estimate hazard ratios (HRs) and 95% CI for associations of hormone therapy with incident young-onset breast cancer. We also estimated risk differences based on cumulative risk until age 55 years. Findings We included 459 476 women aged 16-54 years (mean 42 center dot 0 years [IQR 35 center dot 5-49 center dot 2]), of whom 8455 (2%) developed young-onset breast cancer (diagnosed before age 55 years; median follow-up 78 years [5 center dot 2-11 center dot 2]). Overall, 15% of participants reported using hormone therapy, with oestrogen plus progestin hormone therapy (6%) and unopposed oestrogen (5%) being the most common types. Cumulative risk of young-onset breast cancer was 41% in non-users. Hormone therapy of any type was not associated with incident young-onset breast cancer (HR 096 [95% CI 088 to 104]), but ever oestrogen hormone therapy use was inversely associated (086 [075 to 098]; risk difference-05% [-10 to-00]). The HR for ever oestrogen plus progestin hormone therapy and young-onset breast cancer was 110 (098 to 124), with positive associations observed for long-term use (118 [101 to 138] for >2 years) and use among women without hysterectomy or bilateral oophorectomy (115 [102 to 131]). Oestrogen hormone therapy and young-onset breast cancer association was similar for all breast cancer subtypes, but oestrogen plus progestin hormone therapy was more strongly associated with oestrogen receptor negative (144 [111 to 188]) and triple-negative disease (150 [102 to 220]) than with other subtypes. Interpretation Oestrogen hormone therapy use was inversely associated with young-onset breast cancer, and oestrogen plus progestin hormone therapy was associated with higher young-onset breast cancer incidence among women with intact uterus and ovaries. These findings largely parallel results from studies of hormone use and later-onset breast cancer and provide novel evidence for establishing clinical recommendations among younger women. | Many women under 55 use hormone pills to treat symptoms after surgery or during menopause. While doctors know these hormones can raise breast cancer risk in older women, they wanted to learn about risks in younger women. Researchers studied nearly 460,000 women from around the world who were followed for about 8 years. They found that 8,455 women got breast cancer before age 55. Women who took estrogen-only pills actually had a slightly lower chance of getting breast cancer. However, women who took combination pills with both estrogen and progestin had a higher risk, especially if they used them for more than 2 years or still had their uterus and ovaries. The combination pills were linked more strongly to aggressive types of breast cancer. These results help doctors better understand hormone risks in younger women and can guide treatment decisions for women who need hormone therapy. | Researchers studied whether hormone pills increase breast cancer risk in younger women, since most studies focus on older women after menopause. They followed nearly half a million women from different countries until age 55 to see who developed breast cancer. The women were tracked for about 8 years on average. About 15% of the women used hormone pills, either estrogen alone or estrogen combined with progestin. The study found that estrogen-only pills actually lowered breast cancer risk slightly. However, pills combining estrogen and progestin increased breast cancer risk, especially when used for more than 2 years and in women who still had their uterus and ovaries. The combination pills were more strongly linked to aggressive types of breast cancer that don't respond to hormone treatments. These findings help doctors make better decisions about prescribing hormone pills to younger women who need them for medical reasons or menopause symptoms.RetryClaude can make mistakes. Please double-check responses. | Scientists wanted to learn if hormone pills affect breast cancer risk in younger women under 55. While we know these hormones can increase cancer risk in older women after menopause, we didn't know much about younger women who might take hormones after surgery or for early menopause symptoms. The researchers studied nearly half a million women from different countries for about 8 years. They found that 15% of women used hormone therapy, and 2% developed breast cancer before age 55. Women who took estrogen-only pills actually had slightly lower cancer risk. However, women who took estrogen plus progestin pills together had higher cancer risk, especially if they used them for more than 2 years or still had their uterus and ovaries. The combination hormones were linked more strongly to certain aggressive types of breast cancer. These results help doctors better advise younger women about hormone therapy risks and benefits for their specific situation. |
| CancerRes | 1 | 10.1016/S1470-2045(25)00211-9 | Gemini | Background Oestrogen plus progestin hormone therapy is an established risk factor for breast cancer in postmenopausal women. We examined the less well-studied association between exogenous hormones and breast cancer in young women, who might use hormone therapy after gynaecological surgery or to relieve perimenopausal symptoms. Methods In this pooled cohort analysis, we investigated the relationship between exogenous hormones and breast cancer in young women using data from 10-13 prospective cohorts from North America, Europe, Asia, and Australia. The participating cohorts followed up women for incident breast cancer until age 55 years. We used cohort-stratified, multivariable-adjusted Cox proportional hazards regression to estimate hazard ratios (HRs) and 95% CI for associations of hormone therapy with incident young-onset breast cancer. We also estimated risk differences based on cumulative risk until age 55 years. Findings We included 459 476 women aged 16-54 years (mean 42 center dot 0 years [IQR 35 center dot 5-49 center dot 2]), of whom 8455 (2%) developed young-onset breast cancer (diagnosed before age 55 years; median follow-up 78 years [5 center dot 2-11 center dot 2]). Overall, 15% of participants reported using hormone therapy, with oestrogen plus progestin hormone therapy (6%) and unopposed oestrogen (5%) being the most common types. Cumulative risk of young-onset breast cancer was 41% in non-users. Hormone therapy of any type was not associated with incident young-onset breast cancer (HR 096 [95% CI 088 to 104]), but ever oestrogen hormone therapy use was inversely associated (086 [075 to 098]; risk difference-05% [-10 to-00]). The HR for ever oestrogen plus progestin hormone therapy and young-onset breast cancer was 110 (098 to 124), with positive associations observed for long-term use (118 [101 to 138] for >2 years) and use among women without hysterectomy or bilateral oophorectomy (115 [102 to 131]). Oestrogen hormone therapy and young-onset breast cancer association was similar for all breast cancer subtypes, but oestrogen plus progestin hormone therapy was more strongly associated with oestrogen receptor negative (144 [111 to 188]) and triple-negative disease (150 [102 to 220]) than with other subtypes. Interpretation Oestrogen hormone therapy use was inversely associated with young-onset breast cancer, and oestrogen plus progestin hormone therapy was associated with higher young-onset breast cancer incidence among women with intact uterus and ovaries. These findings largely parallel results from studies of hormone use and later-onset breast cancer and provide novel evidence for establishing clinical recommendations among younger women. | Many younger women use hormone therapy, but its effect on their risk of developing breast cancer is not well understood. This study was conducted to clarify the relationship between hormone therapy use and breast cancer risk in women under the age of 55. By combining data from many research groups, scientists found that using oestrogen-only therapy was linked to a slightly lower risk of breast cancer. However, using a combination of oestrogen and progestin was associated with a higher risk, especially for those who used it for longer than two years. These findings are important because they offer doctors and younger women clearer information about the potential risks of different hormone therapies, which can help them make more informed health decisions. | This study was conducted to better understand the potential connection between hormone therapy and the risk of developing breast cancer at a younger age. While prior research has shown a link in older women, less is known about this risk for younger women who may take hormones for various reasons. By analyzing data from nearly a half-million women, researchers found that using a combination of estrogen and progestin hormone therapy was linked to a higher risk of young-onset breast cancer, especially for those using it for longer periods. Interestingly, using estrogen-only hormone therapy was associated with a lower risk. These results provide important new information that can help doctors and patients make more informed decisions about hormone use and breast cancer risk in younger women. | This study looked at the relationship between using hormone therapy and getting breast cancer at a young age. We combined data from many studies across the world to get a better understanding. We found that using hormone therapy with estrogen alone may be linked to a slightly lower risk of young-onset breast cancer. However, using hormone therapy that contains both estrogen and progestin was associated with a higher risk, especially in women who had not had their uterus or ovaries removed. Our findings can help doctors and patients make more informed decisions about hormone therapy use in younger women, as the risks seem similar to those seen in older women. |
| CancerRes | 1 | 10.1016/S1470-2045(25)00211-10 | DeepSeek | Background Oestrogen plus progestin hormone therapy is an established risk factor for breast cancer in postmenopausal women. We examined the less well-studied association between exogenous hormones and breast cancer in young women, who might use hormone therapy after gynaecological surgery or to relieve perimenopausal symptoms. Methods In this pooled cohort analysis, we investigated the relationship between exogenous hormones and breast cancer in young women using data from 10-13 prospective cohorts from North America, Europe, Asia, and Australia. The participating cohorts followed up women for incident breast cancer until age 55 years. We used cohort-stratified, multivariable-adjusted Cox proportional hazards regression to estimate hazard ratios (HRs) and 95% CI for associations of hormone therapy with incident young-onset breast cancer. We also estimated risk differences based on cumulative risk until age 55 years. Findings We included 459 476 women aged 16-54 years (mean 42 center dot 0 years [IQR 35 center dot 5-49 center dot 2]), of whom 8455 (2%) developed young-onset breast cancer (diagnosed before age 55 years; median follow-up 78 years [5 center dot 2-11 center dot 2]). Overall, 15% of participants reported using hormone therapy, with oestrogen plus progestin hormone therapy (6%) and unopposed oestrogen (5%) being the most common types. Cumulative risk of young-onset breast cancer was 41% in non-users. Hormone therapy of any type was not associated with incident young-onset breast cancer (HR 096 [95% CI 088 to 104]), but ever oestrogen hormone therapy use was inversely associated (086 [075 to 098]; risk difference-05% [-10 to-00]). The HR for ever oestrogen plus progestin hormone therapy and young-onset breast cancer was 110 (098 to 124), with positive associations observed for long-term use (118 [101 to 138] for >2 years) and use among women without hysterectomy or bilateral oophorectomy (115 [102 to 131]). Oestrogen hormone therapy and young-onset breast cancer association was similar for all breast cancer subtypes, but oestrogen plus progestin hormone therapy was more strongly associated with oestrogen receptor negative (144 [111 to 188]) and triple-negative disease (150 [102 to 220]) than with other subtypes. Interpretation Oestrogen hormone therapy use was inversely associated with young-onset breast cancer, and oestrogen plus progestin hormone therapy was associated with higher young-onset breast cancer incidence among women with intact uterus and ovaries. These findings largely parallel results from studies of hormone use and later-onset breast cancer and provide novel evidence for establishing clinical recommendations among younger women. | This study looked at whether hormone therapy (HT) affects breast cancer risk in women under 55. Some women use HT after surgeries like hysterectomies or for menopause symptoms, but its effects on younger women aren’t well known. Researchers combined data from over 450,000 women across 13 countries, tracking who developed breast cancer before age 55. Most women (85%) didn’t use HT, and their average breast cancer risk was 4.1%. Overall, HT didn’t raise breast cancer risk, but women taking only estrogen had slightly lower risk. However, those taking estrogen plus progestin for more than 2 years—especially women who still had their uterus and ovaries—had a higher risk. These findings help doctors give better advice to younger women about hormone therapy and breast cancer. | This study looked at whether hormone therapy (like estrogen or estrogen plus progestin) increases the risk of breast cancer in women under 55. Researchers combined data from over 450,000 women across North America, Europe, Asia, and Australia. Most women did not develop breast cancer, but about 2% did before age 55. The study found that estrogen-only therapy was linked to a slightly lower risk of breast cancer, while estrogen plus progestin therapy—especially when used for more than two years—was tied to a higher risk, particularly in women who still had their uterus and ovaries. These results help doctors and younger women make safer choices about hormone therapy, especially for managing menopause symptoms or after gynecological surgery. The findings match what’s known about older women but provide new guidance for younger ones. | This study looked at whether hormone therapy (like estrogen or estrogen plus progestin) affects breast cancer risk in younger women (under 55). Researchers combined data from over 450,000 women across North America, Europe, Asia, and Australia. They found that, overall, hormone therapy did not increase breast cancer risk in young women. However, estrogen alone was linked to a slightly lower risk, while estrogen plus progestin was tied to a higher risk—especially in women who used it for more than two years or still had their uterus and ovaries. The risk was strongest for aggressive breast cancer types. These findings help doctors give better advice to younger women considering hormone therapy for menopause symptoms or other health needs. |
| CancerRes | 1 | 10.1016/S1470-2045(25)00211-11 | Grok | Background Oestrogen plus progestin hormone therapy is an established risk factor for breast cancer in postmenopausal women. We examined the less well-studied association between exogenous hormones and breast cancer in young women, who might use hormone therapy after gynaecological surgery or to relieve perimenopausal symptoms. Methods In this pooled cohort analysis, we investigated the relationship between exogenous hormones and breast cancer in young women using data from 10-13 prospective cohorts from North America, Europe, Asia, and Australia. The participating cohorts followed up women for incident breast cancer until age 55 years. We used cohort-stratified, multivariable-adjusted Cox proportional hazards regression to estimate hazard ratios (HRs) and 95% CI for associations of hormone therapy with incident young-onset breast cancer. We also estimated risk differences based on cumulative risk until age 55 years. Findings We included 459 476 women aged 16-54 years (mean 42 center dot 0 years [IQR 35 center dot 5-49 center dot 2]), of whom 8455 (2%) developed young-onset breast cancer (diagnosed before age 55 years; median follow-up 78 years [5 center dot 2-11 center dot 2]). Overall, 15% of participants reported using hormone therapy, with oestrogen plus progestin hormone therapy (6%) and unopposed oestrogen (5%) being the most common types. Cumulative risk of young-onset breast cancer was 41% in non-users. Hormone therapy of any type was not associated with incident young-onset breast cancer (HR 096 [95% CI 088 to 104]), but ever oestrogen hormone therapy use was inversely associated (086 [075 to 098]; risk difference-05% [-10 to-00]). The HR for ever oestrogen plus progestin hormone therapy and young-onset breast cancer was 110 (098 to 124), with positive associations observed for long-term use (118 [101 to 138] for >2 years) and use among women without hysterectomy or bilateral oophorectomy (115 [102 to 131]). Oestrogen hormone therapy and young-onset breast cancer association was similar for all breast cancer subtypes, but oestrogen plus progestin hormone therapy was more strongly associated with oestrogen receptor negative (144 [111 to 188]) and triple-negative disease (150 [102 to 220]) than with other subtypes. Interpretation Oestrogen hormone therapy use was inversely associated with young-onset breast cancer, and oestrogen plus progestin hormone therapy was associated with higher young-onset breast cancer incidence among women with intact uterus and ovaries. These findings largely parallel results from studies of hormone use and later-onset breast cancer and provide novel evidence for establishing clinical recommendations among younger women. | Hormone therapy helps some young women after surgery or with early menopause symptoms, but its link to breast cancer in them is not well understood, unlike in older women. Researchers wanted to check if using these hormones raises the risk of breast cancer before age 55. They combined data from many long-term studies involving nearly 460,000 women across continents, tracking them over years to spot new cases. The study found no overall connection between any hormone therapy and young breast cancer, but estrogen alone slightly lowered the risk. However, estrogen plus progestin raised the risk, especially with long use or in women who still have their uterus and ovaries, and more so for harder-to-treat types like triple-negative cancer. These results mirror findings in older women and can guide doctors to give safer advice to younger patients, helping prevent cases and shape better health rules for everyone. | Hormone therapy is a known risk for breast cancer in older women after menopause, but its effects on younger women are not well understood. These younger women might use it after surgery on their reproductive organs or to ease symptoms before menopause. Researchers wanted to see if using hormone therapy raises the chance of getting breast cancer before age 55. They combined data from many long-term studies of over 459,000 women from around the world and tracked who got breast cancer. They found that using estrogen alone lowered the risk a bit, but estrogen plus progestin raised it, especially with long use or in women who still have their uterus and ovaries. It was linked more to harder-to-treat types of breast cancer. These results mirror findings in older women and could help doctors make better guidelines for younger patients, improving care and prevention in the medical field. | Scientists are studying hormone therapy because it raises breast cancer risk in older women after menopause, but we know less about its effects in younger women who might use it after surgery or for early menopause symptoms. This research combined data from many long-term studies across the world, tracking over 459,000 women under 55 to see if hormone use links to breast cancer before that age. They found that using estrogen alone slightly lowers the risk of early breast cancer, while estrogen plus progestin raises it, especially with long-term use or in women who still have their uterus and ovaries. This type also ties more to harder-to-treat cancer types. These results mirror findings in older women and can help doctors give better advice to young women on hormone therapy, improving safety and guiding future research on cancer prevention. |
| CancerRes | 2 | 10.1016/S1470-2045(25)00139-2 | Open AI | Background Cell-free, circulating tumour DNA (ctDNA) is an established measure of minimal residual disease; however, it is not utilised in melanoma management. We investigated whether ctDNA measurements could predict survival outcomes during adjuvant targeted therapy or placebo treatment in stage III melanoma, thereby identifying patients at high risk and low risk of recurrence. Methods Analytically validated mutation-specific droplet digital PCR assays were used to measure BRAF(V600E) or BRAF(V600K) ctDNA in patients aged 18 years or older who were enrolled in the COMBI-AD trial, which was a double-blind, randomised, phase 3 study of oral dabrafenib (150 mg twice daily) plus oral trametinib (2 mg once daily) combination therapy versus two matched placebos in resected BRAF(V600)-mutant stage III melanoma. Patients were screened for enrolment between Jan 31, 2013, and Dec 11, 2014, had an Eastern Cooperative Oncology Group performance status of 0 or 1, and were randomly assigned (1:1) to the two treatment groups. The primary endpoint was recurrence-free survival, and the results from final analysis have been previously published and will not be described here. Biomarker analysis was a prespecified exploratory endpoint and performed in the intention-to-treat population. We compared associations between survival outcomes and baseline (post-resection) ctDNA copies per mL, tumour mutational burden and interferon gamma (IFNG) gene expression. In a subset of patients, ctDNA quantities during follow-up or at recurrence were measured. The trial is registered with ClinicalTrials.gov, NCT01682083, and has been completed. Findings Baseline plasma samples were available for 597 of 870 patients (331 male patients and 266 female patients) and samples for assessing the ctDNA positivity rate at landmark follow-up timepoints of 3 months, 6 months, 9 months, and 12 months after treatment initiation were available for 94 of 870 patients. Additionally, samples were available from 118 of 870 patients within a 2-month timeframe before or after clinical or radiographic recurrence. Median follow-up for the biomarker analyses was 60 months (IQR 39-66) in the combination therapy group and 58 months (21-66) for the placebo group. ctDNA was detectable in 79 (13%) of 597 baseline samples. ctDNA positivity rate and mutant copies per mL plasma were significantly higher in patients with higher disease substages. As a binary variable, ctDNA detection was associated with worse recurrence-free survival (placebo group: median 371 months [95% CI 239-689] vs 2441 months [1728-4313]; hazard ratio [HR] 291 [95% CI 199-425], p<00001); combination therapy group: median 1659 months [95% CI 1202-2680] vs 6811 months [5036-not reached]; HR 298 [195-454], p<00001) and overall survival (placebo group: median 3390 months [1396-not reached] vs not reached; HR 335 [201-555], p<00001); combination therapy group: median 4031 months [2490-not reached] vs not reached; HR 427 [250-727], p<00001) in the placebo group and combination therapy groups. Baseline ctDNA was more strongly associated with survival outcomes than IFNG gene expression or tumour mutational burden. Patients with adverse longitudinal ctDNA kinetics (molecular relapse or persistently positive) had markedly shorter median recurrence-free survival (831 months [95% CI 539-1220] and 532 months [279-not reached], respectively) compared with patients with favourable kinetics (ie, undetectable after positive baseline result: 1925 months [1639-not reached]; and durable undetectable: not reached [3844-not reached], p<00001). Interpretation Droplet digital PCR measurements of ctDNA to assess minimal residual disease before adjuvant targeted therapy and during follow-up can identify patients at high risk of early recurrence. Additional studies using ctDNA measurements to guide therapeutic interventions might lead to improvements in the management of resected stage III melanoma. | This study looked at whether a blood test that detects tiny fragments of cancer DNA, called circulating tumor DNA, could help predict if melanoma will return after surgery. We tested blood from people with stage III melanoma who had a specific BRAF gene change and were part of a large treatment trial. The test used a highly accurate method called droplet digital PCR to measure cancer DNA before treatment and during follow-up. We found that patients with detectable cancer DNA after surgery were more likely to have their cancer come back sooner and live for a shorter time, even if they received targeted therapy. Regular testing during follow-up also helped identify patients at higher risk earlier. These findings suggest that blood-based cancer DNA testing could become an important tool to guide care and monitor recovery for people with melanoma after surgery. | This study looked at a blood test that finds tiny pieces of cancer DNA in the blood of people with stage III melanoma, a serious type of skin cancer. After surgery to remove the cancer, some patients received targeted medicines and others received a placebo. Using a method called droplet digital PCR, the researchers measured cancer DNA before treatment and during follow-up. They found that patients who still had cancer DNA in their blood were more likely to have the cancer come back sooner and live for a shorter time than those without it. Tracking these DNA changes over time also helped show which patients were at higher risk. These findings suggest that measuring cancer DNA in the blood could help doctors identify patients who might need more intensive treatment or closer monitoring, leading to better outcomes in the future. | This study looked at whether a blood test can help predict if melanoma, a serious type of skin cancer, will return after surgery. The test measures tiny pieces of tumour DNA that can be found in the blood, called circulating tumour DNA. We studied patients with stage III melanoma who had surgery and were then given either a targeted cancer treatment or a placebo. Patients with tumour DNA in their blood after surgery were more likely to have their cancer return sooner and had shorter survival times. Regular testing over time also showed that changes in tumour DNA levels could warn of cancer coming back before scans did. These results suggest that measuring tumour DNA in the blood could help doctors find patients at higher risk of relapse and adjust treatment earlier, leading to more personalised and effective care. |
| CancerRes | 2 | 10.1016/S1470-2045(25)00139-3 | Claude | Background Cell-free, circulating tumour DNA (ctDNA) is an established measure of minimal residual disease; however, it is not utilised in melanoma management. We investigated whether ctDNA measurements could predict survival outcomes during adjuvant targeted therapy or placebo treatment in stage III melanoma, thereby identifying patients at high risk and low risk of recurrence. Methods Analytically validated mutation-specific droplet digital PCR assays were used to measure BRAF(V600E) or BRAF(V600K) ctDNA in patients aged 18 years or older who were enrolled in the COMBI-AD trial, which was a double-blind, randomised, phase 3 study of oral dabrafenib (150 mg twice daily) plus oral trametinib (2 mg once daily) combination therapy versus two matched placebos in resected BRAF(V600)-mutant stage III melanoma. Patients were screened for enrolment between Jan 31, 2013, and Dec 11, 2014, had an Eastern Cooperative Oncology Group performance status of 0 or 1, and were randomly assigned (1:1) to the two treatment groups. The primary endpoint was recurrence-free survival, and the results from final analysis have been previously published and will not be described here. Biomarker analysis was a prespecified exploratory endpoint and performed in the intention-to-treat population. We compared associations between survival outcomes and baseline (post-resection) ctDNA copies per mL, tumour mutational burden and interferon gamma (IFNG) gene expression. In a subset of patients, ctDNA quantities during follow-up or at recurrence were measured. The trial is registered with ClinicalTrials.gov, NCT01682083, and has been completed. Findings Baseline plasma samples were available for 597 of 870 patients (331 male patients and 266 female patients) and samples for assessing the ctDNA positivity rate at landmark follow-up timepoints of 3 months, 6 months, 9 months, and 12 months after treatment initiation were available for 94 of 870 patients. Additionally, samples were available from 118 of 870 patients within a 2-month timeframe before or after clinical or radiographic recurrence. Median follow-up for the biomarker analyses was 60 months (IQR 39-66) in the combination therapy group and 58 months (21-66) for the placebo group. ctDNA was detectable in 79 (13%) of 597 baseline samples. ctDNA positivity rate and mutant copies per mL plasma were significantly higher in patients with higher disease substages. As a binary variable, ctDNA detection was associated with worse recurrence-free survival (placebo group: median 371 months [95% CI 239-689] vs 2441 months [1728-4313]; hazard ratio [HR] 291 [95% CI 199-425], p<00001); combination therapy group: median 1659 months [95% CI 1202-2680] vs 6811 months [5036-not reached]; HR 298 [195-454], p<00001) and overall survival (placebo group: median 3390 months [1396-not reached] vs not reached; HR 335 [201-555], p<00001); combination therapy group: median 4031 months [2490-not reached] vs not reached; HR 427 [250-727], p<00001) in the placebo group and combination therapy groups. Baseline ctDNA was more strongly associated with survival outcomes than IFNG gene expression or tumour mutational burden. Patients with adverse longitudinal ctDNA kinetics (molecular relapse or persistently positive) had markedly shorter median recurrence-free survival (831 months [95% CI 539-1220] and 532 months [279-not reached], respectively) compared with patients with favourable kinetics (ie, undetectable after positive baseline result: 1925 months [1639-not reached]; and durable undetectable: not reached [3844-not reached], p<00001). Interpretation Droplet digital PCR measurements of ctDNA to assess minimal residual disease before adjuvant targeted therapy and during follow-up can identify patients at high risk of early recurrence. Additional studies using ctDNA measurements to guide therapeutic interventions might lead to improvements in the management of resected stage III melanoma. | Melanoma is a dangerous type of skin cancer that can spread to other parts of the body. Even after doctors remove all visible cancer through surgery, tiny cancer cells might still be hiding in the body and could cause the cancer to come back. This study looked at a new way to find these hidden cancer cells by testing blood samples for pieces of cancer DNA that float freely in the blood. The researchers studied 597 patients with stage III melanoma who had their tumors removed and were getting follow-up treatment. They found that patients who had cancer DNA in their blood were much more likely to have their cancer return quickly compared to patients without this DNA. The blood test was better at predicting cancer return than other current methods. This discovery is important because it could help doctors identify which patients need more aggressive treatment right after surgery and which patients are at lower risk. In the future, this simple blood test might help doctors make better treatment decisions and catch cancer return much earlier than current methods allow. | Melanoma is a serious type of skin cancer that can spread to other parts of the body. Even after surgery removes all visible cancer, some patients still have tiny amounts of cancer cells left behind that cannot be seen. These hidden cancer cells can cause the cancer to come back later. Scientists wanted to find a better way to predict which patients are most likely to have their cancer return after surgery. They tested blood samples from 597 melanoma patients to look for tiny pieces of cancer DNA floating in the blood. This DNA comes from cancer cells and can be measured using a special lab test. The researchers found that patients with detectable cancer DNA in their blood were much more likely to have their cancer return quickly compared to patients without detectable cancer DNA. This blood test could help doctors identify high-risk patients who might need more aggressive treatment and low-risk patients who might avoid unnecessary treatments. This simple blood test could improve care for melanoma patients by helping doctors make better treatment decisions. | Melanoma is a deadly skin cancer that can spread to other parts of the body. Even after doctors remove all visible cancer, tiny cancer cells may remain hidden and cause the disease to come back. This study looked at a new way to find these hidden cancer cells by testing blood samples for pieces of cancer DNA that break off from tumors. Researchers studied 597 patients with stage III melanoma who had surgery to remove their tumors. They tested blood samples before patients started taking cancer drugs or placebo pills. The team found that patients with cancer DNA in their blood were much more likely to have their cancer return quickly and had shorter survival times. Patients without cancer DNA in their blood stayed cancer-free much longer. This blood test could help doctors identify which patients need more aggressive treatment and closer monitoring. The findings suggest this simple blood test might become a valuable tool to improve care for melanoma patients after surgery. |
| CancerRes | 2 | 10.1016/S1470-2045(25)00139-4 | Gemini | Background Cell-free, circulating tumour DNA (ctDNA) is an established measure of minimal residual disease; however, it is not utilised in melanoma management. We investigated whether ctDNA measurements could predict survival outcomes during adjuvant targeted therapy or placebo treatment in stage III melanoma, thereby identifying patients at high risk and low risk of recurrence. Methods Analytically validated mutation-specific droplet digital PCR assays were used to measure BRAF(V600E) or BRAF(V600K) ctDNA in patients aged 18 years or older who were enrolled in the COMBI-AD trial, which was a double-blind, randomised, phase 3 study of oral dabrafenib (150 mg twice daily) plus oral trametinib (2 mg once daily) combination therapy versus two matched placebos in resected BRAF(V600)-mutant stage III melanoma. Patients were screened for enrolment between Jan 31, 2013, and Dec 11, 2014, had an Eastern Cooperative Oncology Group performance status of 0 or 1, and were randomly assigned (1:1) to the two treatment groups. The primary endpoint was recurrence-free survival, and the results from final analysis have been previously published and will not be described here. Biomarker analysis was a prespecified exploratory endpoint and performed in the intention-to-treat population. We compared associations between survival outcomes and baseline (post-resection) ctDNA copies per mL, tumour mutational burden and interferon gamma (IFNG) gene expression. In a subset of patients, ctDNA quantities during follow-up or at recurrence were measured. The trial is registered with ClinicalTrials.gov, NCT01682083, and has been completed. Findings Baseline plasma samples were available for 597 of 870 patients (331 male patients and 266 female patients) and samples for assessing the ctDNA positivity rate at landmark follow-up timepoints of 3 months, 6 months, 9 months, and 12 months after treatment initiation were available for 94 of 870 patients. Additionally, samples were available from 118 of 870 patients within a 2-month timeframe before or after clinical or radiographic recurrence. Median follow-up for the biomarker analyses was 60 months (IQR 39-66) in the combination therapy group and 58 months (21-66) for the placebo group. ctDNA was detectable in 79 (13%) of 597 baseline samples. ctDNA positivity rate and mutant copies per mL plasma were significantly higher in patients with higher disease substages. As a binary variable, ctDNA detection was associated with worse recurrence-free survival (placebo group: median 371 months [95% CI 239-689] vs 2441 months [1728-4313]; hazard ratio [HR] 291 [95% CI 199-425], p<00001); combination therapy group: median 1659 months [95% CI 1202-2680] vs 6811 months [5036-not reached]; HR 298 [195-454], p<00001) and overall survival (placebo group: median 3390 months [1396-not reached] vs not reached; HR 335 [201-555], p<00001); combination therapy group: median 4031 months [2490-not reached] vs not reached; HR 427 [250-727], p<00001) in the placebo group and combination therapy groups. Baseline ctDNA was more strongly associated with survival outcomes than IFNG gene expression or tumour mutational burden. Patients with adverse longitudinal ctDNA kinetics (molecular relapse or persistently positive) had markedly shorter median recurrence-free survival (831 months [95% CI 539-1220] and 532 months [279-not reached], respectively) compared with patients with favourable kinetics (ie, undetectable after positive baseline result: 1925 months [1639-not reached]; and durable undetectable: not reached [3844-not reached], p<00001). Interpretation Droplet digital PCR measurements of ctDNA to assess minimal residual disease before adjuvant targeted therapy and during follow-up can identify patients at high risk of early recurrence. Additional studies using ctDNA measurements to guide therapeutic interventions might lead to improvements in the management of resected stage III melanoma. | This study investigated a new way to predict which patients with resected stage III melanoma are most likely to have their cancer return after surgery. The research team measured tiny fragments of tumor DNA, called circulating tumor DNA, or ctDNA, in the blood of patients from a large clinical trial. By doing this, they found that patients with higher levels of this DNA in their blood were more likely to have a recurrence and had worse survival outcomes. The study shows that checking for ctDNA is a reliable way to identify patients at a very high risk of their cancer coming back. The findings suggest that doctors might one day use this simple blood test to guide treatment decisions and potentially improve patient outcomes. | This study explores whether a blood test can help doctors treat patients with melanoma, a type of skin cancer. The test looks for small pieces of cancer DNA, called circulating tumor DNA, in the blood of patients who have already had their tumors surgically removed. By using this test, researchers wanted to see if they could predict which patients were more likely to have their cancer return, even when they were receiving treatment. The findings show that detecting this cancer DNA in the blood is a strong sign that the cancer may come back, suggesting that this test could be a valuable tool for doctors. This new information may help doctors better understand a patient's risk and could lead to new ways of managing melanoma in the future. | This study explores how a new blood test can help doctors manage melanoma, a type of skin cancer. The test looks for tiny bits of cancer DNA, called circulating tumor DNA, or ctDNA, in the blood of patients who had stage three melanoma removed. The goal was to see if finding this cancer DNA could predict which patients were more likely to have the cancer come back, even after surgery. By following many patients over several years, the researchers found that those with detectable cancer DNA in their blood were at a much higher risk of their cancer returning and had lower survival rates. This new information could help doctors identify which patients need more aggressive treatment to prevent the cancer from coming back, ultimately improving patient outcomes. |
| CancerRes | 2 | 10.1016/S1470-2045(25)00139-5 | DeepSeek | Background Cell-free, circulating tumour DNA (ctDNA) is an established measure of minimal residual disease; however, it is not utilised in melanoma management. We investigated whether ctDNA measurements could predict survival outcomes during adjuvant targeted therapy or placebo treatment in stage III melanoma, thereby identifying patients at high risk and low risk of recurrence. Methods Analytically validated mutation-specific droplet digital PCR assays were used to measure BRAF(V600E) or BRAF(V600K) ctDNA in patients aged 18 years or older who were enrolled in the COMBI-AD trial, which was a double-blind, randomised, phase 3 study of oral dabrafenib (150 mg twice daily) plus oral trametinib (2 mg once daily) combination therapy versus two matched placebos in resected BRAF(V600)-mutant stage III melanoma. Patients were screened for enrolment between Jan 31, 2013, and Dec 11, 2014, had an Eastern Cooperative Oncology Group performance status of 0 or 1, and were randomly assigned (1:1) to the two treatment groups. The primary endpoint was recurrence-free survival, and the results from final analysis have been previously published and will not be described here. Biomarker analysis was a prespecified exploratory endpoint and performed in the intention-to-treat population. We compared associations between survival outcomes and baseline (post-resection) ctDNA copies per mL, tumour mutational burden and interferon gamma (IFNG) gene expression. In a subset of patients, ctDNA quantities during follow-up or at recurrence were measured. The trial is registered with ClinicalTrials.gov, NCT01682083, and has been completed. Findings Baseline plasma samples were available for 597 of 870 patients (331 male patients and 266 female patients) and samples for assessing the ctDNA positivity rate at landmark follow-up timepoints of 3 months, 6 months, 9 months, and 12 months after treatment initiation were available for 94 of 870 patients. Additionally, samples were available from 118 of 870 patients within a 2-month timeframe before or after clinical or radiographic recurrence. Median follow-up for the biomarker analyses was 60 months (IQR 39-66) in the combination therapy group and 58 months (21-66) for the placebo group. ctDNA was detectable in 79 (13%) of 597 baseline samples. ctDNA positivity rate and mutant copies per mL plasma were significantly higher in patients with higher disease substages. As a binary variable, ctDNA detection was associated with worse recurrence-free survival (placebo group: median 371 months [95% CI 239-689] vs 2441 months [1728-4313]; hazard ratio [HR] 291 [95% CI 199-425], p<00001); combination therapy group: median 1659 months [95% CI 1202-2680] vs 6811 months [5036-not reached]; HR 298 [195-454], p<00001) and overall survival (placebo group: median 3390 months [1396-not reached] vs not reached; HR 335 [201-555], p<00001); combination therapy group: median 4031 months [2490-not reached] vs not reached; HR 427 [250-727], p<00001) in the placebo group and combination therapy groups. Baseline ctDNA was more strongly associated with survival outcomes than IFNG gene expression or tumour mutational burden. Patients with adverse longitudinal ctDNA kinetics (molecular relapse or persistently positive) had markedly shorter median recurrence-free survival (831 months [95% CI 539-1220] and 532 months [279-not reached], respectively) compared with patients with favourable kinetics (ie, undetectable after positive baseline result: 1925 months [1639-not reached]; and durable undetectable: not reached [3844-not reached], p<00001). Interpretation Droplet digital PCR measurements of ctDNA to assess minimal residual disease before adjuvant targeted therapy and during follow-up can identify patients at high risk of early recurrence. Additional studies using ctDNA measurements to guide therapeutic interventions might lead to improvements in the management of resected stage III melanoma. | This study looked at whether a blood test that detects tiny amounts of cancer DNA (ctDNA) could predict if melanoma patients are at high or low risk of their cancer returning after surgery. Researchers tested blood samples from patients with stage III melanoma who were part of a clinical trial comparing a targeted drug combo (dabrafenib + trametinib) to a placebo. They found that patients with detectable ctDNA before treatment had a much higher chance of their cancer coming back and worse survival rates. Tracking ctDNA over time also helped spot patients whose cancer was likely to return early. These results suggest that ctDNA testing could help doctors identify high-risk patients who may need stronger treatment, while sparing low-risk patients from unnecessary therapy. More research is needed to confirm if using ctDNA tests can improve melanoma care. | This study looked at whether a blood test that detects tiny amounts of cancer DNA (ctDNA) could help predict if melanoma (a type of skin cancer) is likely to come back after surgery. Researchers tested blood samples from patients with stage III melanoma who received either a combination of two targeted drugs or a placebo. They found that patients with detectable ctDNA before treatment had a much higher risk of their cancer returning and worse survival rates compared to those without ctDNA. Tracking ctDNA over time also helped identify patients at high risk of relapse. These results suggest that ctDNA testing could help doctors spot patients who need stronger treatment early on, improving their chances of staying cancer-free. More research is needed to confirm how best to use this test in guiding patient care. | This study looked at whether a blood test that detects tiny amounts of cancer DNA (ctDNA) could help predict if melanoma patients would relapse after surgery. Researchers tested blood samples from patients with stage III melanoma who received either a targeted drug combo or a placebo. They found that patients with detectable ctDNA before treatment had a much higher risk of their cancer returning and worse survival rates. The test was better at predicting outcomes than other measures like tumor gene changes. Patients whose ctDNA stayed high or came back during treatment also had quicker relapses. These results suggest that checking ctDNA could help doctors identify high-risk patients early, possibly leading to better treatment decisions. More research is needed to confirm if adjusting therapy based on ctDNA can improve outcomes. |
| CancerRes | 2 | 10.1016/S1470-2045(25)00139-6 | Grok | Background Cell-free, circulating tumour DNA (ctDNA) is an established measure of minimal residual disease; however, it is not utilised in melanoma management. We investigated whether ctDNA measurements could predict survival outcomes during adjuvant targeted therapy or placebo treatment in stage III melanoma, thereby identifying patients at high risk and low risk of recurrence. Methods Analytically validated mutation-specific droplet digital PCR assays were used to measure BRAF(V600E) or BRAF(V600K) ctDNA in patients aged 18 years or older who were enrolled in the COMBI-AD trial, which was a double-blind, randomised, phase 3 study of oral dabrafenib (150 mg twice daily) plus oral trametinib (2 mg once daily) combination therapy versus two matched placebos in resected BRAF(V600)-mutant stage III melanoma. Patients were screened for enrolment between Jan 31, 2013, and Dec 11, 2014, had an Eastern Cooperative Oncology Group performance status of 0 or 1, and were randomly assigned (1:1) to the two treatment groups. The primary endpoint was recurrence-free survival, and the results from final analysis have been previously published and will not be described here. Biomarker analysis was a prespecified exploratory endpoint and performed in the intention-to-treat population. We compared associations between survival outcomes and baseline (post-resection) ctDNA copies per mL, tumour mutational burden and interferon gamma (IFNG) gene expression. In a subset of patients, ctDNA quantities during follow-up or at recurrence were measured. The trial is registered with ClinicalTrials.gov, NCT01682083, and has been completed. Findings Baseline plasma samples were available for 597 of 870 patients (331 male patients and 266 female patients) and samples for assessing the ctDNA positivity rate at landmark follow-up timepoints of 3 months, 6 months, 9 months, and 12 months after treatment initiation were available for 94 of 870 patients. Additionally, samples were available from 118 of 870 patients within a 2-month timeframe before or after clinical or radiographic recurrence. Median follow-up for the biomarker analyses was 60 months (IQR 39-66) in the combination therapy group and 58 months (21-66) for the placebo group. ctDNA was detectable in 79 (13%) of 597 baseline samples. ctDNA positivity rate and mutant copies per mL plasma were significantly higher in patients with higher disease substages. As a binary variable, ctDNA detection was associated with worse recurrence-free survival (placebo group: median 371 months [95% CI 239-689] vs 2441 months [1728-4313]; hazard ratio [HR] 291 [95% CI 199-425], p<00001); combination therapy group: median 1659 months [95% CI 1202-2680] vs 6811 months [5036-not reached]; HR 298 [195-454], p<00001) and overall survival (placebo group: median 3390 months [1396-not reached] vs not reached; HR 335 [201-555], p<00001); combination therapy group: median 4031 months [2490-not reached] vs not reached; HR 427 [250-727], p<00001) in the placebo group and combination therapy groups. Baseline ctDNA was more strongly associated with survival outcomes than IFNG gene expression or tumour mutational burden. Patients with adverse longitudinal ctDNA kinetics (molecular relapse or persistently positive) had markedly shorter median recurrence-free survival (831 months [95% CI 539-1220] and 532 months [279-not reached], respectively) compared with patients with favourable kinetics (ie, undetectable after positive baseline result: 1925 months [1639-not reached]; and durable undetectable: not reached [3844-not reached], p<00001). Interpretation Droplet digital PCR measurements of ctDNA to assess minimal residual disease before adjuvant targeted therapy and during follow-up can identify patients at high risk of early recurrence. Additional studies using ctDNA measurements to guide therapeutic interventions might lead to improvements in the management of resected stage III melanoma. | This study looks at whether tiny bits of cancer DNA floating in the blood can help predict if skin cancer will return in people with stage III melanoma after surgery. Researchers wanted to see if measuring this DNA could spot patients at high or low risk of the cancer coming back, while they got either real treatment or a fake pill in a big trial. They used a special test to check blood samples from over 500 patients before and during treatment. The results showed that finding this cancer DNA early on meant a much higher chance of the cancer returning soon and shorter survival, no matter the treatment. It was a better predictor than other tests. This could help doctors find high-risk patients faster and create better ways to treat them, possibly saving more lives in the future. | Melanoma is a serious skin cancer, and after surgery to remove it in stage III patients, some still face recurrence. This study looked at whether testing for tiny bits of tumor DNA in the blood could help predict who might stay cancer-free longer during follow-up treatments. Researchers used a precise blood test on samples from 597 patients in a major trial, where half got targeted drugs and half got placebos. They found that detecting tumor DNA before treatment meant shorter survival times without cancer returning, and it was a better predictor than other factors like gene activity. Tracking these DNA levels over time also spotted high-risk patients early. For the public, this simple blood test could guide doctors to give extra care to those at greater risk, potentially saving lives by catching cancer comeback sooner. | This study looks at using a blood test to find tiny bits of cancer DNA in people with stage III skin cancer called melanoma, after surgery. Researchers wanted to see if this test could spot who might have the cancer come back soon, even while getting extra treatment or a fake pill. They tested blood from over 500 patients in a big trial and found that those with cancer DNA in their blood at the start had much shorter time without cancer returning or living longer, compared to those without it. This worked better than other tests like gene activity or tumor changes. During check-ups, if the cancer DNA stayed or came back, patients did worse. These findings could help doctors pick out high-risk people early and try new ways to stop the cancer, making treatments better for everyone with this disease. |
| CancerRes | 3 | 10.1016/S1470-2045(24)00373-6 | Open AI | Background CAPItello-291 is an ongoing phase 3 trial in which capivasertib-fulvestrant significantly improved progression-free survival versus placebo-fulvestrant in patients with hormone receptor-positive, HER2-negative advanced breast cancer who had relapse or disease progression during or after aromatase inhibitor treatment, in both the overall population and in patients with PIK3CA, , AKT1, , or PTEN- altered tumours. This study further explored patient-reported health-related quality of life (HRQOL), functioning, symptoms, and symptom tolerability in CAPItello-291. Methods This phase 3, randomised, double-blind, placebo-controlled trial, which was conducted across 193 hospitals and cancer centres in 19 countries, enrolled women with any menopausal status or men, aged >= 18 years (>= 20 years in Japan), with hormone receptor-positive, HER2-negative locally advanced or metastatic breast cancer who had relapse or disease progression during or after treatment with an aromatase inhibitor, with or without previous cyclindependent kinase (CDK) 4 or 6 inhibitor therapy. Patients had an Eastern Cooperative Oncology Group/WHO performance score of 0 or 1 and could have received up to two previous lines of endocrine therapy and up to one previous line of chemotherapy for advanced disease. Patients were randomly assigned (1:1) using block randomisation (stratified according to the presence or absence of liver metastases, previous use of a CDK4/6 inhibitor [yes vs no], and geographical region) to receive oral capivasertib 400 mg (twice daily for 4 days, followed by 3 days off) plus intramuscular fulvestrant 500 mg (every 14 days for the first three injections, then every 28 days) or placebo with matching fulvestrant dosing. The dual primary endpoint of the trial was investigator-assessed progression-free survival assessed both in the overall population and among patients with PIK3CA, , AKT1, , or PTEN-altered tumours. The EORTC Quality of Life Questionnaire 30-item core module (QLQ-C30) and breast module (QLQ-BR23), Patient- Reported Outcomes version of the Common Terminology Criteria for Adverse Events (PRO-CTCAE), and Patient Global Impression of Treatment Tolerability (PGI-TT) questionnaires were used to assess patient-reported outcomes. Evaluation of EORTC QLQ-C30 and EORTC QLQ-BR23 were secondary endpoints and evaluation of PRO-CTCAE and PGI-TT were pre-defined exploratory endpoints, and these endpoints are the subject of analysis in this Article. Data were collected at baseline and prespecified timepoints. Patient-reported outcomes were analysed in all randomly assigned patients with an evaluable baseline assessment and at least one evaluable post-baseline assessment. Change from baseline was assessed using mixed model with repeated measures for EORTC QLQ-C30 and summarised for QLQ-BR23. Time to deterioration was described using the Kaplan-Meier method. PGI-TT and PRO-CTCAE responses were summarised at each treatment cycle. Patient-reported outcomes were not prospectively powered for statistical comparison. The trial is registered with ClinicalTrials.gov, NCT04305496. Findings Between June 2, 2020, and Oct 13, 2021, 901 patients were enrolled, of whom 708 patients were randomly assigned to receive capivasertib-fulvestrant (n=355) or placebo-fulvestrant (n=353). The median age of the patients was 59 years (IQR 51-67) in the capivasertib-fulvestrant group and 58 years (IQR 49-66) in the placebo-fulvestrant group. At data cutoff (Aug 15, 2022), the median duration of follow-up for progression-free survival in censored patients was 130 months (IQR 91-167) for capivasertib-fulvestrant and 127 months (IQR 20-164) for placebo- fulvestrant in the overall population. EORTC QLQ-C30 global health status/quality of life (GHS/QOL) scores were maintained from baseline and were similar between treatment groups throughout the study period (difference in mean change from baseline of -25 [95% CI -45 to -06] with capivasertib-fulvestrant vs -56 [-79 to -34] with placebo-fulvestrant; treatment difference 31 [95% CI 02 to 60]). Median time to deterioration in EORTC QLQ-C30 GHS/QOL was 249 months (95% CI 138 to not reached) in the capivasertib-fulvestrant group and 120 months (102 to 157) in the placebo-fulvestrant group (hazard ratio [HR] 070, 95% CI 053 to 092). Time to deterioration HRs for all EORTC QLQ-C30 and QLQ-BR23 subscale scores showed little difference between the treatment groups, except for diarrhoea, which was worse in the capivasertib-fulvestrant group than in the placebo-fulvestrant group (HR 275, 95% CI 201-381). In PRO-CTCAE symptom assessment, the proportion of patients reporting loose and watery stools frequently or almost constantly was 29% higher at cycle 1, day 15 in the capivasertib-fulvestrant group than in the placebo-fulvestrant group, decreasing at subsequent cycles. Other PRO-CTCAE-reported symptoms (rash, mouth or throat sores, itchy skin, and numbness or tingling in hands or feet) were absent or mild in most patients in both groups throughout treatment. According to the PGI-TT, most patients in both groups reported not at all or a little bit of bother from treatment side-effects. Interpretation Patient-reported outcomes from CAPItello-291 demonstrated that capivasertib-fulvestrant delayed time to deterioration of GHS/QOL and maintained other dimensions of HRQOL (except symptoms of diarrhoea) similarly to fulvestrant. With the clinical efficacy and manageable safety profile, these exploratory results further support the positive benefit-risk profile of capivasertib-fulvestrant in this population. Copyright (c) 2024 Elsevier Ltd. All rights reserved, including those for text and data mining, AI training, and similar technologies. | This study looked at whether adding a new medicine, capivasertib, to standard hormone therapy (fulvestrant) could help people with a type of advanced breast cancer that does not have the HER2 protein. Patients had already tried other treatments, but their cancer had returned or worsened. Researchers compared this combination to fulvestrant with a placebo. They also asked patients to report on their quality of life, symptoms, and how tolerable the treatment felt. The results showed that people taking capivasertib with fulvestrant stayed well for longer before their quality of life worsened, compared to those on fulvestrant alone. Overall quality of life remained similar between groups, except that diarrhoea was more common with capivasertib. Most other side effects were mild and not very bothersome. These findings suggest that adding capivasertib could provide extra benefit without greatly reducing quality of life, supporting its use as a treatment option for this group of patients. | This study looked at whether adding a new drug, capivasertib, to the standard treatment fulvestrant could help people with a certain type of advanced breast cancer live longer without the disease getting worse, while also maintaining their quality of life. Over 900 patients from 19 countries took part. They answered questionnaires about their overall health, daily functioning, and treatment side effects during the study. Results showed that the combination treatment delayed the time before patients’ quality of life worsened compared to standard treatment alone, without adding major new problems, except for more frequent diarrhea, which improved over time. Other side effects were generally mild. These findings suggest that capivasertib with fulvestrant may offer extra time without disease progression while allowing patients to maintain daily activities, supporting its use as a promising treatment option for this group of patients. | This study looked at whether adding a new medicine, capivasertib, to standard treatment with fulvestrant could help people with a certain type of advanced breast cancer live longer without their disease getting worse, while also maintaining their quality of life. The trial involved 901 patients in 19 countries. Participants reported on their daily well-being, symptoms, and how tolerable they found the treatment. Results showed that those who received capivasertib with fulvestrant kept their overall quality of life for longer than those who received fulvestrant alone, with the main difference being more frequent diarrhoea in the combination group. Other side effects were mostly mild and manageable. These findings suggest that the combination treatment can slow cancer progression without greatly affecting most aspects of daily life, supporting its potential as a valuable option for patients with this type of breast cancer. |
| CancerRes | 3 | 10.1016/S1470-2045(24)00373-7 | Claude | Background CAPItello-291 is an ongoing phase 3 trial in which capivasertib-fulvestrant significantly improved progression-free survival versus placebo-fulvestrant in patients with hormone receptor-positive, HER2-negative advanced breast cancer who had relapse or disease progression during or after aromatase inhibitor treatment, in both the overall population and in patients with PIK3CA, , AKT1, , or PTEN- altered tumours. This study further explored patient-reported health-related quality of life (HRQOL), functioning, symptoms, and symptom tolerability in CAPItello-291. Methods This phase 3, randomised, double-blind, placebo-controlled trial, which was conducted across 193 hospitals and cancer centres in 19 countries, enrolled women with any menopausal status or men, aged >= 18 years (>= 20 years in Japan), with hormone receptor-positive, HER2-negative locally advanced or metastatic breast cancer who had relapse or disease progression during or after treatment with an aromatase inhibitor, with or without previous cyclindependent kinase (CDK) 4 or 6 inhibitor therapy. Patients had an Eastern Cooperative Oncology Group/WHO performance score of 0 or 1 and could have received up to two previous lines of endocrine therapy and up to one previous line of chemotherapy for advanced disease. Patients were randomly assigned (1:1) using block randomisation (stratified according to the presence or absence of liver metastases, previous use of a CDK4/6 inhibitor [yes vs no], and geographical region) to receive oral capivasertib 400 mg (twice daily for 4 days, followed by 3 days off) plus intramuscular fulvestrant 500 mg (every 14 days for the first three injections, then every 28 days) or placebo with matching fulvestrant dosing. The dual primary endpoint of the trial was investigator-assessed progression-free survival assessed both in the overall population and among patients with PIK3CA, , AKT1, , or PTEN-altered tumours. The EORTC Quality of Life Questionnaire 30-item core module (QLQ-C30) and breast module (QLQ-BR23), Patient- Reported Outcomes version of the Common Terminology Criteria for Adverse Events (PRO-CTCAE), and Patient Global Impression of Treatment Tolerability (PGI-TT) questionnaires were used to assess patient-reported outcomes. Evaluation of EORTC QLQ-C30 and EORTC QLQ-BR23 were secondary endpoints and evaluation of PRO-CTCAE and PGI-TT were pre-defined exploratory endpoints, and these endpoints are the subject of analysis in this Article. Data were collected at baseline and prespecified timepoints. Patient-reported outcomes were analysed in all randomly assigned patients with an evaluable baseline assessment and at least one evaluable post-baseline assessment. Change from baseline was assessed using mixed model with repeated measures for EORTC QLQ-C30 and summarised for QLQ-BR23. Time to deterioration was described using the Kaplan-Meier method. PGI-TT and PRO-CTCAE responses were summarised at each treatment cycle. Patient-reported outcomes were not prospectively powered for statistical comparison. The trial is registered with ClinicalTrials.gov, NCT04305496. Findings Between June 2, 2020, and Oct 13, 2021, 901 patients were enrolled, of whom 708 patients were randomly assigned to receive capivasertib-fulvestrant (n=355) or placebo-fulvestrant (n=353). The median age of the patients was 59 years (IQR 51-67) in the capivasertib-fulvestrant group and 58 years (IQR 49-66) in the placebo-fulvestrant group. At data cutoff (Aug 15, 2022), the median duration of follow-up for progression-free survival in censored patients was 130 months (IQR 91-167) for capivasertib-fulvestrant and 127 months (IQR 20-164) for placebo- fulvestrant in the overall population. EORTC QLQ-C30 global health status/quality of life (GHS/QOL) scores were maintained from baseline and were similar between treatment groups throughout the study period (difference in mean change from baseline of -25 [95% CI -45 to -06] with capivasertib-fulvestrant vs -56 [-79 to -34] with placebo-fulvestrant; treatment difference 31 [95% CI 02 to 60]). Median time to deterioration in EORTC QLQ-C30 GHS/QOL was 249 months (95% CI 138 to not reached) in the capivasertib-fulvestrant group and 120 months (102 to 157) in the placebo-fulvestrant group (hazard ratio [HR] 070, 95% CI 053 to 092). Time to deterioration HRs for all EORTC QLQ-C30 and QLQ-BR23 subscale scores showed little difference between the treatment groups, except for diarrhoea, which was worse in the capivasertib-fulvestrant group than in the placebo-fulvestrant group (HR 275, 95% CI 201-381). In PRO-CTCAE symptom assessment, the proportion of patients reporting loose and watery stools frequently or almost constantly was 29% higher at cycle 1, day 15 in the capivasertib-fulvestrant group than in the placebo-fulvestrant group, decreasing at subsequent cycles. Other PRO-CTCAE-reported symptoms (rash, mouth or throat sores, itchy skin, and numbness or tingling in hands or feet) were absent or mild in most patients in both groups throughout treatment. According to the PGI-TT, most patients in both groups reported not at all or a little bit of bother from treatment side-effects. Interpretation Patient-reported outcomes from CAPItello-291 demonstrated that capivasertib-fulvestrant delayed time to deterioration of GHS/QOL and maintained other dimensions of HRQOL (except symptoms of diarrhoea) similarly to fulvestrant. With the clinical efficacy and manageable safety profile, these exploratory results further support the positive benefit-risk profile of capivasertib-fulvestrant in this population. Copyright (c) 2024 Elsevier Ltd. All rights reserved, including those for text and data mining, AI training, and similar technologies. | Breast cancer that spreads to other parts of the body is hard to treat. This study tested a new drug called capivasertib when combined with fulvestrant, a standard treatment. Researchers wanted to know if this new combination would help patients live better lives while fighting their cancer. They studied 708 women and men from 19 countries who had breast cancer that had grown despite other treatments. Half the patients got the new drug combination, and half got a fake pill with fulvestrant. The researchers asked patients how they felt about their quality of life, symptoms, and daily activities using special questionnaires. Results showed that patients taking the new combination kept their quality of life longer than those getting the fake pill. The main side effect was loose stools, which got better over time. Most patients said the treatment side effects did not bother them much. This study shows that the new combination helps patients feel good while fighting cancer, giving doctors a better treatment option for advanced breast cancer. | Advanced breast cancer often comes back even after treatment. This study looked at how patients felt when taking two different drug combinations. Researchers gave 708 women with a common type of breast cancer either capivasertib plus fulvestrant or a fake pill plus fulvestrant. They wanted to see if the new drug combo would help patients feel better and have fewer symptoms. Patients filled out surveys about their daily life, pain levels, and how bothersome their treatment was. The results showed that patients taking capivasertib stayed healthier for longer and kept their quality of life better than those taking the fake treatment. The main side effect was diarrhea, which happened more often with the new drug but got better over time. Most patients said their treatment did not bother them much. This research shows that capivasertib plus fulvestrant helps patients live better while fighting their cancer, giving doctors a new way to treat advanced breast cancer that focuses on both stopping the disease and keeping patients comfortable. | Breast cancer that feeds on hormones can spread to other parts of the body and become hard to treat. When common treatments stop working, doctors need new options. This study tested a new drug combo to see if it helps patients live longer without their cancer getting worse, and more importantly, if patients feel good while taking it. Researchers gave 708 women either the new drug combo (capivasertib plus fulvestrant) or a fake pill plus the standard drug. They asked patients to fill out forms about how they felt, their energy levels, and any side effects every few weeks. The results showed that patients taking the new combo felt just as good as those taking the standard treatment. Their quality of life stayed stable for longer - about 25 months versus 12 months. The main side effect was diarrhea, but most patients said the treatment side effects did not bother them much. This means the new drug combo not only helps fight cancer better but also lets patients maintain their daily lives and feel well during treatment. |
| CancerRes | 3 | 10.1016/S1470-2045(24)00373-8 | Gemini | Background CAPItello-291 is an ongoing phase 3 trial in which capivasertib-fulvestrant significantly improved progression-free survival versus placebo-fulvestrant in patients with hormone receptor-positive, HER2-negative advanced breast cancer who had relapse or disease progression during or after aromatase inhibitor treatment, in both the overall population and in patients with PIK3CA, , AKT1, , or PTEN- altered tumours. This study further explored patient-reported health-related quality of life (HRQOL), functioning, symptoms, and symptom tolerability in CAPItello-291. Methods This phase 3, randomised, double-blind, placebo-controlled trial, which was conducted across 193 hospitals and cancer centres in 19 countries, enrolled women with any menopausal status or men, aged >= 18 years (>= 20 years in Japan), with hormone receptor-positive, HER2-negative locally advanced or metastatic breast cancer who had relapse or disease progression during or after treatment with an aromatase inhibitor, with or without previous cyclindependent kinase (CDK) 4 or 6 inhibitor therapy. Patients had an Eastern Cooperative Oncology Group/WHO performance score of 0 or 1 and could have received up to two previous lines of endocrine therapy and up to one previous line of chemotherapy for advanced disease. Patients were randomly assigned (1:1) using block randomisation (stratified according to the presence or absence of liver metastases, previous use of a CDK4/6 inhibitor [yes vs no], and geographical region) to receive oral capivasertib 400 mg (twice daily for 4 days, followed by 3 days off) plus intramuscular fulvestrant 500 mg (every 14 days for the first three injections, then every 28 days) or placebo with matching fulvestrant dosing. The dual primary endpoint of the trial was investigator-assessed progression-free survival assessed both in the overall population and among patients with PIK3CA, , AKT1, , or PTEN-altered tumours. The EORTC Quality of Life Questionnaire 30-item core module (QLQ-C30) and breast module (QLQ-BR23), Patient- Reported Outcomes version of the Common Terminology Criteria for Adverse Events (PRO-CTCAE), and Patient Global Impression of Treatment Tolerability (PGI-TT) questionnaires were used to assess patient-reported outcomes. Evaluation of EORTC QLQ-C30 and EORTC QLQ-BR23 were secondary endpoints and evaluation of PRO-CTCAE and PGI-TT were pre-defined exploratory endpoints, and these endpoints are the subject of analysis in this Article. Data were collected at baseline and prespecified timepoints. Patient-reported outcomes were analysed in all randomly assigned patients with an evaluable baseline assessment and at least one evaluable post-baseline assessment. Change from baseline was assessed using mixed model with repeated measures for EORTC QLQ-C30 and summarised for QLQ-BR23. Time to deterioration was described using the Kaplan-Meier method. PGI-TT and PRO-CTCAE responses were summarised at each treatment cycle. Patient-reported outcomes were not prospectively powered for statistical comparison. The trial is registered with ClinicalTrials.gov, NCT04305496. Findings Between June 2, 2020, and Oct 13, 2021, 901 patients were enrolled, of whom 708 patients were randomly assigned to receive capivasertib-fulvestrant (n=355) or placebo-fulvestrant (n=353). The median age of the patients was 59 years (IQR 51-67) in the capivasertib-fulvestrant group and 58 years (IQR 49-66) in the placebo-fulvestrant group. At data cutoff (Aug 15, 2022), the median duration of follow-up for progression-free survival in censored patients was 130 months (IQR 91-167) for capivasertib-fulvestrant and 127 months (IQR 20-164) for placebo- fulvestrant in the overall population. EORTC QLQ-C30 global health status/quality of life (GHS/QOL) scores were maintained from baseline and were similar between treatment groups throughout the study period (difference in mean change from baseline of -25 [95% CI -45 to -06] with capivasertib-fulvestrant vs -56 [-79 to -34] with placebo-fulvestrant; treatment difference 31 [95% CI 02 to 60]). Median time to deterioration in EORTC QLQ-C30 GHS/QOL was 249 months (95% CI 138 to not reached) in the capivasertib-fulvestrant group and 120 months (102 to 157) in the placebo-fulvestrant group (hazard ratio [HR] 070, 95% CI 053 to 092). Time to deterioration HRs for all EORTC QLQ-C30 and QLQ-BR23 subscale scores showed little difference between the treatment groups, except for diarrhoea, which was worse in the capivasertib-fulvestrant group than in the placebo-fulvestrant group (HR 275, 95% CI 201-381). In PRO-CTCAE symptom assessment, the proportion of patients reporting loose and watery stools frequently or almost constantly was 29% higher at cycle 1, day 15 in the capivasertib-fulvestrant group than in the placebo-fulvestrant group, decreasing at subsequent cycles. Other PRO-CTCAE-reported symptoms (rash, mouth or throat sores, itchy skin, and numbness or tingling in hands or feet) were absent or mild in most patients in both groups throughout treatment. According to the PGI-TT, most patients in both groups reported not at all or a little bit of bother from treatment side-effects. Interpretation Patient-reported outcomes from CAPItello-291 demonstrated that capivasertib-fulvestrant delayed time to deterioration of GHS/QOL and maintained other dimensions of HRQOL (except symptoms of diarrhoea) similarly to fulvestrant. With the clinical efficacy and manageable safety profile, these exploratory results further support the positive benefit-risk profile of capivasertib-fulvestrant in this population. Copyright (c) 2024 Elsevier Ltd. All rights reserved, including those for text and data mining, AI training, and similar technologies. | In a study involving patients with advanced breast cancer, researchers wanted to understand how a new combination therapy called capivasertib-fulvestrant affected their quality of life, compared to the standard treatment of fulvestrant alone. The goal was to see if the new medicine, which was already known to help people live longer without their cancer getting worse, also allowed them to feel well and continue daily activities. The study found that patients taking the new combination therapy maintained a good quality of life and had a longer time before their health and well-being started to decline. While a side effect of diarrhea was more common with the new treatment, most patients found the side effects manageable. These results suggest that the new therapy not only helps fight the cancer but also allows patients to maintain a good quality of life, which is a key factor for doctors and patients when choosing a treatment plan. | A clinical trial called CAPItello-291 studied a new treatment for advanced breast cancer. The treatment, a combination of capivasertib and fulvestrant, has already been shown to help patients live longer without their cancer getting worse. This new part of the study focused on how the treatment affected patients' daily lives. Researchers wanted to understand if the new drug combination caused side effects that would lower a patient's quality of life. The study found that, while the new treatment did cause more diarrhea, it did not significantly worsen a patient's overall health or quality of life compared to the standard treatment. These findings are important because they show the new drug combination is not only effective but also tolerable, which helps doctors and patients make better treatment decisions. | In a study on advanced breast cancer, researchers wanted to find out if a new drug combination, capivasertib plus fulvestrant, could improve quality of life for patients. The initial research showed this new treatment slowed down cancer growth more effectively than the standard therapy. This part of the study focused on patient-reported outcomes to understand how the new treatment affects daily life, including symptoms and general well-being. The findings suggest that the new drug combination helps maintain a patient’s overall quality of life and delays its decline for a longer period. While the new treatment did cause more instances of diarrhea, other side effects were similar to the standard treatment. These results are important because they show that a more effective cancer treatment doesn't necessarily come at the cost of a significantly worse quality of life, which is a critical consideration for patients and doctors. |
| CancerRes | 3 | 10.1016/S1470-2045(24)00373-9 | DeepSeek | Background CAPItello-291 is an ongoing phase 3 trial in which capivasertib-fulvestrant significantly improved progression-free survival versus placebo-fulvestrant in patients with hormone receptor-positive, HER2-negative advanced breast cancer who had relapse or disease progression during or after aromatase inhibitor treatment, in both the overall population and in patients with PIK3CA, , AKT1, , or PTEN- altered tumours. This study further explored patient-reported health-related quality of life (HRQOL), functioning, symptoms, and symptom tolerability in CAPItello-291. Methods This phase 3, randomised, double-blind, placebo-controlled trial, which was conducted across 193 hospitals and cancer centres in 19 countries, enrolled women with any menopausal status or men, aged >= 18 years (>= 20 years in Japan), with hormone receptor-positive, HER2-negative locally advanced or metastatic breast cancer who had relapse or disease progression during or after treatment with an aromatase inhibitor, with or without previous cyclindependent kinase (CDK) 4 or 6 inhibitor therapy. Patients had an Eastern Cooperative Oncology Group/WHO performance score of 0 or 1 and could have received up to two previous lines of endocrine therapy and up to one previous line of chemotherapy for advanced disease. Patients were randomly assigned (1:1) using block randomisation (stratified according to the presence or absence of liver metastases, previous use of a CDK4/6 inhibitor [yes vs no], and geographical region) to receive oral capivasertib 400 mg (twice daily for 4 days, followed by 3 days off) plus intramuscular fulvestrant 500 mg (every 14 days for the first three injections, then every 28 days) or placebo with matching fulvestrant dosing. The dual primary endpoint of the trial was investigator-assessed progression-free survival assessed both in the overall population and among patients with PIK3CA, , AKT1, , or PTEN-altered tumours. The EORTC Quality of Life Questionnaire 30-item core module (QLQ-C30) and breast module (QLQ-BR23), Patient- Reported Outcomes version of the Common Terminology Criteria for Adverse Events (PRO-CTCAE), and Patient Global Impression of Treatment Tolerability (PGI-TT) questionnaires were used to assess patient-reported outcomes. Evaluation of EORTC QLQ-C30 and EORTC QLQ-BR23 were secondary endpoints and evaluation of PRO-CTCAE and PGI-TT were pre-defined exploratory endpoints, and these endpoints are the subject of analysis in this Article. Data were collected at baseline and prespecified timepoints. Patient-reported outcomes were analysed in all randomly assigned patients with an evaluable baseline assessment and at least one evaluable post-baseline assessment. Change from baseline was assessed using mixed model with repeated measures for EORTC QLQ-C30 and summarised for QLQ-BR23. Time to deterioration was described using the Kaplan-Meier method. PGI-TT and PRO-CTCAE responses were summarised at each treatment cycle. Patient-reported outcomes were not prospectively powered for statistical comparison. The trial is registered with ClinicalTrials.gov, NCT04305496. Findings Between June 2, 2020, and Oct 13, 2021, 901 patients were enrolled, of whom 708 patients were randomly assigned to receive capivasertib-fulvestrant (n=355) or placebo-fulvestrant (n=353). The median age of the patients was 59 years (IQR 51-67) in the capivasertib-fulvestrant group and 58 years (IQR 49-66) in the placebo-fulvestrant group. At data cutoff (Aug 15, 2022), the median duration of follow-up for progression-free survival in censored patients was 130 months (IQR 91-167) for capivasertib-fulvestrant and 127 months (IQR 20-164) for placebo- fulvestrant in the overall population. EORTC QLQ-C30 global health status/quality of life (GHS/QOL) scores were maintained from baseline and were similar between treatment groups throughout the study period (difference in mean change from baseline of -25 [95% CI -45 to -06] with capivasertib-fulvestrant vs -56 [-79 to -34] with placebo-fulvestrant; treatment difference 31 [95% CI 02 to 60]). Median time to deterioration in EORTC QLQ-C30 GHS/QOL was 249 months (95% CI 138 to not reached) in the capivasertib-fulvestrant group and 120 months (102 to 157) in the placebo-fulvestrant group (hazard ratio [HR] 070, 95% CI 053 to 092). Time to deterioration HRs for all EORTC QLQ-C30 and QLQ-BR23 subscale scores showed little difference between the treatment groups, except for diarrhoea, which was worse in the capivasertib-fulvestrant group than in the placebo-fulvestrant group (HR 275, 95% CI 201-381). In PRO-CTCAE symptom assessment, the proportion of patients reporting loose and watery stools frequently or almost constantly was 29% higher at cycle 1, day 15 in the capivasertib-fulvestrant group than in the placebo-fulvestrant group, decreasing at subsequent cycles. Other PRO-CTCAE-reported symptoms (rash, mouth or throat sores, itchy skin, and numbness or tingling in hands or feet) were absent or mild in most patients in both groups throughout treatment. According to the PGI-TT, most patients in both groups reported not at all or a little bit of bother from treatment side-effects. Interpretation Patient-reported outcomes from CAPItello-291 demonstrated that capivasertib-fulvestrant delayed time to deterioration of GHS/QOL and maintained other dimensions of HRQOL (except symptoms of diarrhoea) similarly to fulvestrant. With the clinical efficacy and manageable safety profile, these exploratory results further support the positive benefit-risk profile of capivasertib-fulvestrant in this population. Copyright (c) 2024 Elsevier Ltd. All rights reserved, including those for text and data mining, AI training, and similar technologies. | This study looked at how well a new drug combination (capivasertib + fulvestrant) worked for people with advanced breast cancer that grows with hormones but doesn’t respond to HER2 treatments. The trial involved 708 patients who had tried other therapies that stopped working. Researchers wanted to know if this combo helped patients live longer without their cancer worsening and if it affected their daily lives. Results showed that the capivasertib-fulvestrant mix kept patients’ quality of life stable for nearly twice as long (25 months vs. 12 months) compared to fulvestrant alone. While some patients had temporary diarrhea, most side effects (like rash or numbness) were mild. These findings suggest the new combo not only slows cancer growth but also helps patients feel better for longer, offering a promising option for this hard-to-treat cancer. | This study looked at whether adding the drug capivasertib to standard hormone therapy (fulvestrant) helps patients with advanced breast cancer that grew after previous treatment. Researchers wanted to know if this combo improved survival while keeping patients’ quality of life stable. Over 700 patients took part, with half receiving the new treatment and half receiving a placebo with fulvestrant. Results showed that those on capivasertib-fulvestrant lived longer without their cancer worsening and maintained their overall well-being similarly to the placebo group. However, they did experience more diarrhea. Most side effects were mild, and patients generally felt the treatment was manageable. These findings suggest capivasertib-fulvestrant could be a helpful option for advanced breast cancer patients, balancing effectiveness with quality of life. | This study looked at how well patients with advanced breast cancer (hormone receptor-positive, HER2-negative) felt while taking a new treatment, capivasertib plus fulvestrant, compared to fulvestrant alone. The goal was to see if the combo improved survival without making patients feel worse. Over 700 patients took part, reporting their quality of life, symptoms, and side effects through questionnaires. Results showed that the combo delayed worsening of overall health and quality of life compared to fulvestrant alone (24.9 vs. 12 months). While diarrhea was more common with the combo, most side effects (like rash or numbness) were mild. Importantly, patients on the combo lived longer without their cancer growing and generally felt as well as those on standard treatment. These findings suggest capivasertib-fulvestrant could be a promising option, balancing effectiveness with manageable side effects for advanced breast cancer patients. |
| CancerRes | 3 | 10.1016/S1470-2045(24)00373-10 | Grok | Background CAPItello-291 is an ongoing phase 3 trial in which capivasertib-fulvestrant significantly improved progression-free survival versus placebo-fulvestrant in patients with hormone receptor-positive, HER2-negative advanced breast cancer who had relapse or disease progression during or after aromatase inhibitor treatment, in both the overall population and in patients with PIK3CA, , AKT1, , or PTEN- altered tumours. This study further explored patient-reported health-related quality of life (HRQOL), functioning, symptoms, and symptom tolerability in CAPItello-291. Methods This phase 3, randomised, double-blind, placebo-controlled trial, which was conducted across 193 hospitals and cancer centres in 19 countries, enrolled women with any menopausal status or men, aged >= 18 years (>= 20 years in Japan), with hormone receptor-positive, HER2-negative locally advanced or metastatic breast cancer who had relapse or disease progression during or after treatment with an aromatase inhibitor, with or without previous cyclindependent kinase (CDK) 4 or 6 inhibitor therapy. Patients had an Eastern Cooperative Oncology Group/WHO performance score of 0 or 1 and could have received up to two previous lines of endocrine therapy and up to one previous line of chemotherapy for advanced disease. Patients were randomly assigned (1:1) using block randomisation (stratified according to the presence or absence of liver metastases, previous use of a CDK4/6 inhibitor [yes vs no], and geographical region) to receive oral capivasertib 400 mg (twice daily for 4 days, followed by 3 days off) plus intramuscular fulvestrant 500 mg (every 14 days for the first three injections, then every 28 days) or placebo with matching fulvestrant dosing. The dual primary endpoint of the trial was investigator-assessed progression-free survival assessed both in the overall population and among patients with PIK3CA, , AKT1, , or PTEN-altered tumours. The EORTC Quality of Life Questionnaire 30-item core module (QLQ-C30) and breast module (QLQ-BR23), Patient- Reported Outcomes version of the Common Terminology Criteria for Adverse Events (PRO-CTCAE), and Patient Global Impression of Treatment Tolerability (PGI-TT) questionnaires were used to assess patient-reported outcomes. Evaluation of EORTC QLQ-C30 and EORTC QLQ-BR23 were secondary endpoints and evaluation of PRO-CTCAE and PGI-TT were pre-defined exploratory endpoints, and these endpoints are the subject of analysis in this Article. Data were collected at baseline and prespecified timepoints. Patient-reported outcomes were analysed in all randomly assigned patients with an evaluable baseline assessment and at least one evaluable post-baseline assessment. Change from baseline was assessed using mixed model with repeated measures for EORTC QLQ-C30 and summarised for QLQ-BR23. Time to deterioration was described using the Kaplan-Meier method. PGI-TT and PRO-CTCAE responses were summarised at each treatment cycle. Patient-reported outcomes were not prospectively powered for statistical comparison. The trial is registered with ClinicalTrials.gov, NCT04305496. Findings Between June 2, 2020, and Oct 13, 2021, 901 patients were enrolled, of whom 708 patients were randomly assigned to receive capivasertib-fulvestrant (n=355) or placebo-fulvestrant (n=353). The median age of the patients was 59 years (IQR 51-67) in the capivasertib-fulvestrant group and 58 years (IQR 49-66) in the placebo-fulvestrant group. At data cutoff (Aug 15, 2022), the median duration of follow-up for progression-free survival in censored patients was 130 months (IQR 91-167) for capivasertib-fulvestrant and 127 months (IQR 20-164) for placebo- fulvestrant in the overall population. EORTC QLQ-C30 global health status/quality of life (GHS/QOL) scores were maintained from baseline and were similar between treatment groups throughout the study period (difference in mean change from baseline of -25 [95% CI -45 to -06] with capivasertib-fulvestrant vs -56 [-79 to -34] with placebo-fulvestrant; treatment difference 31 [95% CI 02 to 60]). Median time to deterioration in EORTC QLQ-C30 GHS/QOL was 249 months (95% CI 138 to not reached) in the capivasertib-fulvestrant group and 120 months (102 to 157) in the placebo-fulvestrant group (hazard ratio [HR] 070, 95% CI 053 to 092). Time to deterioration HRs for all EORTC QLQ-C30 and QLQ-BR23 subscale scores showed little difference between the treatment groups, except for diarrhoea, which was worse in the capivasertib-fulvestrant group than in the placebo-fulvestrant group (HR 275, 95% CI 201-381). In PRO-CTCAE symptom assessment, the proportion of patients reporting loose and watery stools frequently or almost constantly was 29% higher at cycle 1, day 15 in the capivasertib-fulvestrant group than in the placebo-fulvestrant group, decreasing at subsequent cycles. Other PRO-CTCAE-reported symptoms (rash, mouth or throat sores, itchy skin, and numbness or tingling in hands or feet) were absent or mild in most patients in both groups throughout treatment. According to the PGI-TT, most patients in both groups reported not at all or a little bit of bother from treatment side-effects. Interpretation Patient-reported outcomes from CAPItello-291 demonstrated that capivasertib-fulvestrant delayed time to deterioration of GHS/QOL and maintained other dimensions of HRQOL (except symptoms of diarrhoea) similarly to fulvestrant. With the clinical efficacy and manageable safety profile, these exploratory results further support the positive benefit-risk profile of capivasertib-fulvestrant in this population. Copyright (c) 2024 Elsevier Ltd. All rights reserved, including those for text and data mining, AI training, and similar technologies. | Breast cancer that responds to hormones but not to HER2 protein can be hard to treat when it spreads. This study tested if adding capivasertib to fulvestrant helps patients feel better and live with fewer symptoms. Researchers ran a large trial with 708 adults who had tried other treatments. They randomly gave half the group capivasertib plus fulvestrant and the other half a fake pill plus fulvestrant, without knowing which. Patients filled out surveys about their quality of life, daily functioning, and side effects like diarrhea or rash. Results showed the new combo kept overall health and well-being steady longer than fulvestrant alone, with only more diarrhea at first. Most side effects were mild, and patients weren't very bothered. This means the treatment could offer longer disease control without hurting daily life much, giving hope for better options for people with advanced breast cancer. | Breast cancer that responds to hormones but not to HER2 protein can be hard to treat after other therapies fail. This study looked at how a new drug combo, capivasertib plus fulvestrant, affects patients' daily life compared to fulvestrant alone. Researchers wanted to see if it keeps quality of life good while slowing cancer growth. They ran a big trial with over 700 women and some men, using surveys to track health, symptoms, and side effects. Results showed the combo maintained overall well-being and delayed its decline, with similar effects to the standard treatment except for more diarrhea at first. These findings suggest the new combo is tolerable and helpful, which could guide doctors to offer better options for advanced breast cancer patients, improving care and hope for many. | Breast cancer that responds to hormones but not to HER2 protein can be hard to treat when it spreads. This study looks at how a new drug combo, capivasertib plus fulvestrant, affects patients' daily life compared to fulvestrant alone. Researchers wanted to see if it keeps quality of life good while slowing cancer growth. They ran a big trial with 708 adults, giving half the new combo and half a fake pill with fulvestrant. Patients filled out surveys about health, symptoms, and side effects over time. Results showed the combo kept overall well-being steady and delayed its decline longer than the other treatment, though it caused more diarrhea at first. Other issues like rash or tiredness were mild. For people with this cancer, this means a better option to fight the disease without ruining life quality much, helping doctors choose treatments that balance health and comfort. |
| CancerRes | 4 | 10.1016/S1470-2045(25)00128-8 | Open AI | Background Infections are the second leading cause of death in patients with cancer and are often caused by resistant bacteria. However, the frequency of antimicrobial resistance (AMR) in outpatients with cancer is not well understood. We aimed to compare the frequency of AMR bacterial pathogens in outpatients with and without cancer. Methods This retrospective cohort study evaluated antimicrobial susceptibility of bacteria isolated from adults (aged >= 18 years) with and without cancer seeking care in 198 outpatient health-care settings in the USA. Data were collected using the BD Insights Research Database. Patients who were not prescribed cancer medications or not admitted to an inpatient cancer unit in the predefined period were categorised as patients without cancer. Patients were included in the cancer cohort if they received medication solely or sometimes indicated for cancer. Data on gender and race or ethnicity were not collected. Non-duplicate and non-contaminant pathogens collected from various samples (ie, blood, intra-abdominal, respiratory, urine, skin or wound, and other) in outpatients were used to assess the coprimary outcomes: overall and source-specific proportions of non-susceptible pathogen isolates with corresponding AMR odds ratios (ORs); and rates of AMR pathogens per 1000 isolates with corresponding AMR incidence rate ratio (IRR) in patients with and without cancer. Findings Data were collected between April 1, 2018, and Dec 31, 2022. 53 006 (3 center dot 2%) of 1 655 594 pathogens identified were from 27 421 patients with cancer and 1 602 588 (96 center dot 8%) were from 928 128 patients without cancer. For Pseudomonas aeruginosa, carbapenem non-susceptibility was higher in pathogen isolates from patients with cancer (816 [14 center dot 4%] of 5683) than patients without cancer (10 709 [11 center dot 3%] 94 419; OR 1 center dot 22 [95% CI 1 center dot 13-1 center dot 32]). For Enterobacterales, fluoroquinolone non-susceptibility was higher in pathogen isolates from patients with cancer (8662 [28 center dot 0%] of 30 867) than patients without cancer (238 479 [21 center dot 8%] of 1 095 996; OR 1 center dot 44 [1 center dot 40-1 center dot 47]), as was carbapenem non-susceptibility (472 [1 center dot 5%] of 30 867 vs 9165 [0 center dot 8%] of 1 095 996; OR 1 center dot 89 [1 center dot 72-2 center dot 07]), multidrug-resistant pathogens (2672 [8 center dot 7%] of 30 867 vs 48 962 [4 center dot 5%] of 1 095 996; OR 2 center dot 03 [1 center dot 95-2 center dot 11]), and extended-spectrum beta-lactamase producers (4343 [16 center dot 5%] of 26 327 vs 93 977 [9 center dot 4%] of 996 853; OR 1 center dot 96 [1 center dot 90-2 center dot 03]). For Staphylococcus aureus, meticillin resistance was higher in pathogen isolates from patients with cancer (4747 [53 center dot 0%] of 8959) than patients without cancer (129 291 [48 center dot 3%] of 267 520; OR 1 center dot 20 [1 center dot 15-1 center dot 25]). For Enterococcus spp, vancomycin resistance was higher in pathogen isolates from patients with cancer (1329 [18 center dot 6%] of 7145) than patients without cancer (12 333 [9 center dot 1%] of 135 772]; ORR 2 center dot 20 [2 center dot 06-2 center dot 34). The rates and corresponding IRRs of AMR pathogens per 1000 isolates was also higher in patients with cancer compared with patients without cancer, particularly for carbapenem non-susceptible P aeruginosa (IRR 2 center dot 06 [1 center dot 91-2 center dot 21]) and vancomycin-resistant enterococci (IRR 3 center dot 06 [2 center dot 89-3 center dot 24]). For all comparisons, p<0 center dot 0001. Interpretation AMR proportions and IRRs for most key pathogens were up to three-times higher in isolates from outpatients with cancer than those without cancer, highlighting the need for enhanced surveillance, infection prevention, and timely diagnostic stewardship to improve antibiotic prescribing in this population. Copyright (c) 2025 Elsevier Ltd. All rights reserved, including those for text and data mining, AI training, and similar technologies. | People with cancer are more likely to get serious infections, and these infections are often caused by bacteria that do not respond to common antibiotics. This study looked at lab results from over 1.6 million bacteria collected from patients in clinics across the United States. We compared people with cancer to those without cancer to see how often the bacteria were resistant to antibiotics. We found that resistance was much more common in patients with cancer—sometimes up to three times higher—especially for bacteria like Pseudomonas aeruginosa, Enterobacterales, Staphylococcus aureus, and Enterococcus. These findings show that patients with cancer face a greater risk from difficult-to-treat infections. Better infection control, faster diagnosis, and careful use of antibiotics are needed to protect these patients and improve their treatment outcomes. | People with cancer often face a higher risk of infections, which can be harder to treat if the germs are resistant to antibiotics. This study looked at over 1.6 million bacterial samples from adults in outpatient clinics across the United States to compare antibiotic resistance in people with and without cancer. We found that patients with cancer were more likely to have bacteria resistant to multiple important antibiotics, sometimes up to three times more often. This was seen in several common bacteria, including those that cause serious bloodstream and urinary infections. These results suggest that doctors should pay extra attention to infection prevention, quick diagnosis, and careful use of antibiotics in people with cancer. By improving how we track and respond to resistant infections, we can help protect this vulnerable group and guide better care in clinics. | People with cancer are more likely to get infections, and these infections can be harder to treat if the bacteria do not respond to common antibiotics. This study looked at bacteria from outpatients across many health centers in the United States to compare those with and without cancer. We found that drug-resistant bacteria were more common in people with cancer, sometimes up to three times higher, for several dangerous types of bacteria. These results mean that doctors need to be extra careful when treating infections in patients with cancer, using faster testing, better infection control, and smarter antibiotic choices. By understanding how often these resistant infections happen, health care providers can act sooner to prevent them and choose treatments that work, which may help save lives. |
| CancerRes | 4 | 10.1016/S1470-2045(25)00128-9 | Claude | Background Infections are the second leading cause of death in patients with cancer and are often caused by resistant bacteria. However, the frequency of antimicrobial resistance (AMR) in outpatients with cancer is not well understood. We aimed to compare the frequency of AMR bacterial pathogens in outpatients with and without cancer. Methods This retrospective cohort study evaluated antimicrobial susceptibility of bacteria isolated from adults (aged >= 18 years) with and without cancer seeking care in 198 outpatient health-care settings in the USA. Data were collected using the BD Insights Research Database. Patients who were not prescribed cancer medications or not admitted to an inpatient cancer unit in the predefined period were categorised as patients without cancer. Patients were included in the cancer cohort if they received medication solely or sometimes indicated for cancer. Data on gender and race or ethnicity were not collected. Non-duplicate and non-contaminant pathogens collected from various samples (ie, blood, intra-abdominal, respiratory, urine, skin or wound, and other) in outpatients were used to assess the coprimary outcomes: overall and source-specific proportions of non-susceptible pathogen isolates with corresponding AMR odds ratios (ORs); and rates of AMR pathogens per 1000 isolates with corresponding AMR incidence rate ratio (IRR) in patients with and without cancer. Findings Data were collected between April 1, 2018, and Dec 31, 2022. 53 006 (3 center dot 2%) of 1 655 594 pathogens identified were from 27 421 patients with cancer and 1 602 588 (96 center dot 8%) were from 928 128 patients without cancer. For Pseudomonas aeruginosa, carbapenem non-susceptibility was higher in pathogen isolates from patients with cancer (816 [14 center dot 4%] of 5683) than patients without cancer (10 709 [11 center dot 3%] 94 419; OR 1 center dot 22 [95% CI 1 center dot 13-1 center dot 32]). For Enterobacterales, fluoroquinolone non-susceptibility was higher in pathogen isolates from patients with cancer (8662 [28 center dot 0%] of 30 867) than patients without cancer (238 479 [21 center dot 8%] of 1 095 996; OR 1 center dot 44 [1 center dot 40-1 center dot 47]), as was carbapenem non-susceptibility (472 [1 center dot 5%] of 30 867 vs 9165 [0 center dot 8%] of 1 095 996; OR 1 center dot 89 [1 center dot 72-2 center dot 07]), multidrug-resistant pathogens (2672 [8 center dot 7%] of 30 867 vs 48 962 [4 center dot 5%] of 1 095 996; OR 2 center dot 03 [1 center dot 95-2 center dot 11]), and extended-spectrum beta-lactamase producers (4343 [16 center dot 5%] of 26 327 vs 93 977 [9 center dot 4%] of 996 853; OR 1 center dot 96 [1 center dot 90-2 center dot 03]). For Staphylococcus aureus, meticillin resistance was higher in pathogen isolates from patients with cancer (4747 [53 center dot 0%] of 8959) than patients without cancer (129 291 [48 center dot 3%] of 267 520; OR 1 center dot 20 [1 center dot 15-1 center dot 25]). For Enterococcus spp, vancomycin resistance was higher in pathogen isolates from patients with cancer (1329 [18 center dot 6%] of 7145) than patients without cancer (12 333 [9 center dot 1%] of 135 772]; ORR 2 center dot 20 [2 center dot 06-2 center dot 34). The rates and corresponding IRRs of AMR pathogens per 1000 isolates was also higher in patients with cancer compared with patients without cancer, particularly for carbapenem non-susceptible P aeruginosa (IRR 2 center dot 06 [1 center dot 91-2 center dot 21]) and vancomycin-resistant enterococci (IRR 3 center dot 06 [2 center dot 89-3 center dot 24]). For all comparisons, p<0 center dot 0001. Interpretation AMR proportions and IRRs for most key pathogens were up to three-times higher in isolates from outpatients with cancer than those without cancer, highlighting the need for enhanced surveillance, infection prevention, and timely diagnostic stewardship to improve antibiotic prescribing in this population. Copyright (c) 2025 Elsevier Ltd. All rights reserved, including those for text and data mining, AI training, and similar technologies. | Cancer patients often get infections that can be deadly, and these infections are getting harder to treat because germs are becoming resistant to medicines called antibiotics. Researchers wanted to find out if cancer patients who visit clinics and doctors' offices have more antibiotic-resistant germs than people without cancer. They looked at nearly two million germ samples from 198 medical centers across America over four years. They found that cancer patients had much higher rates of dangerous, drug-resistant germs compared to people without cancer. Some resistant germs were up to three times more common in cancer patients. This is important because it shows that cancer patients face extra risks from hard-to-treat infections when they visit doctors' offices. The findings suggest that doctors need better ways to prevent infections in cancer patients, faster tests to identify resistant germs, and smarter choices about which antibiotics to use. This research helps doctors understand they need special care plans to protect cancer patients from dangerous infections. | Cancer patients getting outpatient care face a bigger problem with drug-resistant germs than people without cancer. Researchers looked at over 1.6 million germ samples from 198 medical centers across America between 2018 and 2022. They wanted to find out how often cancer patients get infections that don't respond to common medicines. The study compared germ samples from about 27,000 cancer patients to nearly one million patients without cancer. Results showed that cancer patients were much more likely to have germs that resist treatment. Some dangerous germs were up to three times more resistant in cancer patients. For example, a germ called Enterococcus was twice as likely to resist vancomycin, a powerful antibiotic. Another germ, Pseudomonas, was more likely to resist carbapenem drugs. This research helps doctors understand that cancer patients need special care to prevent and treat infections. Better testing and smarter use of antibiotics could help protect these vulnerable patients from hard-to-treat infections. | Cancer patients visiting doctors' offices and clinics face a serious hidden problem: the germs that make them sick are much more likely to resist common antibiotics compared to people without cancer. Researchers studied over 1.6 million bacterial samples from nearly one million patients across 198 medical centers in America over four years. They compared infection patterns between cancer patients and healthy people to understand this growing threat. The study found that cancer patients had up to three times higher rates of drug-resistant bacteria, including dangerous germs like MRSA and superbugs that don't respond to multiple antibiotics. This happens because cancer treatments weaken the immune system and patients often receive many antibiotics, which helps resistant bacteria grow stronger. These findings are crucial because infections are the second leading cause of death in cancer patients. The research shows that doctors need better tools to quickly identify these resistant germs and choose the right antibiotics faster. This could save lives by ensuring cancer patients get effective treatment before their infections become life-threatening. |
| CancerRes | 4 | 10.1016/S1470-2045(25)00128-10 | Gemini | Background Infections are the second leading cause of death in patients with cancer and are often caused by resistant bacteria. However, the frequency of antimicrobial resistance (AMR) in outpatients with cancer is not well understood. We aimed to compare the frequency of AMR bacterial pathogens in outpatients with and without cancer. Methods This retrospective cohort study evaluated antimicrobial susceptibility of bacteria isolated from adults (aged >= 18 years) with and without cancer seeking care in 198 outpatient health-care settings in the USA. Data were collected using the BD Insights Research Database. Patients who were not prescribed cancer medications or not admitted to an inpatient cancer unit in the predefined period were categorised as patients without cancer. Patients were included in the cancer cohort if they received medication solely or sometimes indicated for cancer. Data on gender and race or ethnicity were not collected. Non-duplicate and non-contaminant pathogens collected from various samples (ie, blood, intra-abdominal, respiratory, urine, skin or wound, and other) in outpatients were used to assess the coprimary outcomes: overall and source-specific proportions of non-susceptible pathogen isolates with corresponding AMR odds ratios (ORs); and rates of AMR pathogens per 1000 isolates with corresponding AMR incidence rate ratio (IRR) in patients with and without cancer. Findings Data were collected between April 1, 2018, and Dec 31, 2022. 53 006 (3 center dot 2%) of 1 655 594 pathogens identified were from 27 421 patients with cancer and 1 602 588 (96 center dot 8%) were from 928 128 patients without cancer. For Pseudomonas aeruginosa, carbapenem non-susceptibility was higher in pathogen isolates from patients with cancer (816 [14 center dot 4%] of 5683) than patients without cancer (10 709 [11 center dot 3%] 94 419; OR 1 center dot 22 [95% CI 1 center dot 13-1 center dot 32]). For Enterobacterales, fluoroquinolone non-susceptibility was higher in pathogen isolates from patients with cancer (8662 [28 center dot 0%] of 30 867) than patients without cancer (238 479 [21 center dot 8%] of 1 095 996; OR 1 center dot 44 [1 center dot 40-1 center dot 47]), as was carbapenem non-susceptibility (472 [1 center dot 5%] of 30 867 vs 9165 [0 center dot 8%] of 1 095 996; OR 1 center dot 89 [1 center dot 72-2 center dot 07]), multidrug-resistant pathogens (2672 [8 center dot 7%] of 30 867 vs 48 962 [4 center dot 5%] of 1 095 996; OR 2 center dot 03 [1 center dot 95-2 center dot 11]), and extended-spectrum beta-lactamase producers (4343 [16 center dot 5%] of 26 327 vs 93 977 [9 center dot 4%] of 996 853; OR 1 center dot 96 [1 center dot 90-2 center dot 03]). For Staphylococcus aureus, meticillin resistance was higher in pathogen isolates from patients with cancer (4747 [53 center dot 0%] of 8959) than patients without cancer (129 291 [48 center dot 3%] of 267 520; OR 1 center dot 20 [1 center dot 15-1 center dot 25]). For Enterococcus spp, vancomycin resistance was higher in pathogen isolates from patients with cancer (1329 [18 center dot 6%] of 7145) than patients without cancer (12 333 [9 center dot 1%] of 135 772]; ORR 2 center dot 20 [2 center dot 06-2 center dot 34). The rates and corresponding IRRs of AMR pathogens per 1000 isolates was also higher in patients with cancer compared with patients without cancer, particularly for carbapenem non-susceptible P aeruginosa (IRR 2 center dot 06 [1 center dot 91-2 center dot 21]) and vancomycin-resistant enterococci (IRR 3 center dot 06 [2 center dot 89-3 center dot 24]). For all comparisons, p<0 center dot 0001. Interpretation AMR proportions and IRRs for most key pathogens were up to three-times higher in isolates from outpatients with cancer than those without cancer, highlighting the need for enhanced surveillance, infection prevention, and timely diagnostic stewardship to improve antibiotic prescribing in this population. Copyright (c) 2025 Elsevier Ltd. All rights reserved, including those for text and data mining, AI training, and similar technologies. | Having a strong immune system is important for fighting off infections. People with cancer often have weakened immune systems, which makes them more likely to get sick. When these patients get infections, the bacteria that cause them are more likely to be resistant to common antibiotics. This study looked at a large number of patient records from across the USA to compare bacteria found in people with and without cancer. The findings show that drug-resistant bacteria were much more common in outpatients with cancer. This is an important discovery because it highlights a serious risk for these patients and suggests that doctors need to be more careful with how they prevent and treat infections in people with cancer. The research community can use this information to develop better ways to test for and treat these difficult-to-treat infections. | People with cancer are more likely to get infections, which are the second leading cause of death for them. These infections can be hard to treat because the bacteria causing them are often resistant to common antibiotics. This study looked at a large group of people to see how often these resistant bacteria appeared in patients with and without cancer. The research found that antibiotic resistance was much more common in people with cancer, sometimes up to three times higher than in people without cancer. This is a significant discovery for the medical community, as it shows that doctors need to be extra careful when treating infections in cancer patients. The findings suggest that better strategies are needed to prevent infections and to make sure the right antibiotics are used to improve care for this vulnerable group of patients. | Infections are a major concern for people with cancer, often caused by bacteria that have become resistant to common antibiotics. This study was conducted to understand how frequently this antimicrobial resistance occurs in cancer patients who are not in the hospital. By looking at a large database of patient information from many different outpatient clinics, researchers compared the types of bacteria found in people with and without cancer. The study's findings showed that bacteria from cancer patients were significantly more resistant to antibiotics than bacteria from patients without cancer. For example, some antibiotic-resistant bacteria were found to be up to three times more common in the cancer patient group. These results highlight the need for healthcare providers to be more careful about preventing infections and prescribing antibiotics for people with cancer, as this group is at a higher risk of having difficult-to-treat infections. |
| CancerRes | 4 | 10.1016/S1470-2045(25)00128-11 | DeepSeek | Background Infections are the second leading cause of death in patients with cancer and are often caused by resistant bacteria. However, the frequency of antimicrobial resistance (AMR) in outpatients with cancer is not well understood. We aimed to compare the frequency of AMR bacterial pathogens in outpatients with and without cancer. Methods This retrospective cohort study evaluated antimicrobial susceptibility of bacteria isolated from adults (aged >= 18 years) with and without cancer seeking care in 198 outpatient health-care settings in the USA. Data were collected using the BD Insights Research Database. Patients who were not prescribed cancer medications or not admitted to an inpatient cancer unit in the predefined period were categorised as patients without cancer. Patients were included in the cancer cohort if they received medication solely or sometimes indicated for cancer. Data on gender and race or ethnicity were not collected. Non-duplicate and non-contaminant pathogens collected from various samples (ie, blood, intra-abdominal, respiratory, urine, skin or wound, and other) in outpatients were used to assess the coprimary outcomes: overall and source-specific proportions of non-susceptible pathogen isolates with corresponding AMR odds ratios (ORs); and rates of AMR pathogens per 1000 isolates with corresponding AMR incidence rate ratio (IRR) in patients with and without cancer. Findings Data were collected between April 1, 2018, and Dec 31, 2022. 53 006 (3 center dot 2%) of 1 655 594 pathogens identified were from 27 421 patients with cancer and 1 602 588 (96 center dot 8%) were from 928 128 patients without cancer. For Pseudomonas aeruginosa, carbapenem non-susceptibility was higher in pathogen isolates from patients with cancer (816 [14 center dot 4%] of 5683) than patients without cancer (10 709 [11 center dot 3%] 94 419; OR 1 center dot 22 [95% CI 1 center dot 13-1 center dot 32]). For Enterobacterales, fluoroquinolone non-susceptibility was higher in pathogen isolates from patients with cancer (8662 [28 center dot 0%] of 30 867) than patients without cancer (238 479 [21 center dot 8%] of 1 095 996; OR 1 center dot 44 [1 center dot 40-1 center dot 47]), as was carbapenem non-susceptibility (472 [1 center dot 5%] of 30 867 vs 9165 [0 center dot 8%] of 1 095 996; OR 1 center dot 89 [1 center dot 72-2 center dot 07]), multidrug-resistant pathogens (2672 [8 center dot 7%] of 30 867 vs 48 962 [4 center dot 5%] of 1 095 996; OR 2 center dot 03 [1 center dot 95-2 center dot 11]), and extended-spectrum beta-lactamase producers (4343 [16 center dot 5%] of 26 327 vs 93 977 [9 center dot 4%] of 996 853; OR 1 center dot 96 [1 center dot 90-2 center dot 03]). For Staphylococcus aureus, meticillin resistance was higher in pathogen isolates from patients with cancer (4747 [53 center dot 0%] of 8959) than patients without cancer (129 291 [48 center dot 3%] of 267 520; OR 1 center dot 20 [1 center dot 15-1 center dot 25]). For Enterococcus spp, vancomycin resistance was higher in pathogen isolates from patients with cancer (1329 [18 center dot 6%] of 7145) than patients without cancer (12 333 [9 center dot 1%] of 135 772]; ORR 2 center dot 20 [2 center dot 06-2 center dot 34). The rates and corresponding IRRs of AMR pathogens per 1000 isolates was also higher in patients with cancer compared with patients without cancer, particularly for carbapenem non-susceptible P aeruginosa (IRR 2 center dot 06 [1 center dot 91-2 center dot 21]) and vancomycin-resistant enterococci (IRR 3 center dot 06 [2 center dot 89-3 center dot 24]). For all comparisons, p<0 center dot 0001. Interpretation AMR proportions and IRRs for most key pathogens were up to three-times higher in isolates from outpatients with cancer than those without cancer, highlighting the need for enhanced surveillance, infection prevention, and timely diagnostic stewardship to improve antibiotic prescribing in this population. Copyright (c) 2025 Elsevier Ltd. All rights reserved, including those for text and data mining, AI training, and similar technologies. | This study looked at how often infections in cancer patients are caused by bacteria that don’t respond to common antibiotics (antimicrobial resistance, or AMR). Researchers compared lab test results from over 27,000 cancer patients and nearly 1 million non-cancer patients across U.S. clinics. They found that cancer patients were much more likely to have resistant infections—sometimes two to three times higher—especially for dangerous bacteria like Pseudomonas and Staphylococcus. These resistant infections make treatment harder and raise the risk of serious illness. The results show that cancer patients need better infection tracking, prevention, and smarter antibiotic use to keep them safe. This research helps doctors understand the growing AMR threat in vulnerable groups like cancer patients. | This study looked at how often infections caused by antibiotic-resistant bacteria happen in cancer patients visiting outpatient clinics compared to people without cancer. Researchers checked records from nearly 200 U.S. clinics between 2018 and 2022, analyzing over 1.6 million bacterial samples. They found that cancer patients were much more likely to have resistant infections—especially to strong antibiotics like carbapenems and vancomycin—sometimes up to three times higher than non-cancer patients. For example, resistant Staphylococcus and Enterococcus bacteria were more common in cancer patients. These findings matter because infections are a leading cause of death in cancer patients, and antibiotic resistance makes treatment harder. The study suggests better monitoring, prevention, and smarter antibiotic use to protect this high-risk group. | This study looked at how often antibiotic-resistant infections happen in cancer patients visiting clinics compared to people without cancer. Infections are a major threat to cancer patients, but we don’t fully know how common resistant bacteria are in outpatient settings. Researchers analyzed data from nearly 2 million bacterial samples collected from U.S. clinics between 2018 and 2022. They found that cancer patients were up to three times more likely to have infections resistant to common antibiotics, including those used as last-resort treatments. For example, resistance to carbapenems (a strong antibiotic) was higher in cancer patients for certain bacteria like Pseudomonas and Enterobacterales. These results show that cancer patients are at greater risk for hard-to-treat infections, even outside hospitals. The findings call for better infection tracking, prevention, and smarter antibiotic use to protect this vulnerable group. |
| CancerRes | 4 | 10.1016/S1470-2045(25)00128-12 | Grok | Background Infections are the second leading cause of death in patients with cancer and are often caused by resistant bacteria. However, the frequency of antimicrobial resistance (AMR) in outpatients with cancer is not well understood. We aimed to compare the frequency of AMR bacterial pathogens in outpatients with and without cancer. Methods This retrospective cohort study evaluated antimicrobial susceptibility of bacteria isolated from adults (aged >= 18 years) with and without cancer seeking care in 198 outpatient health-care settings in the USA. Data were collected using the BD Insights Research Database. Patients who were not prescribed cancer medications or not admitted to an inpatient cancer unit in the predefined period were categorised as patients without cancer. Patients were included in the cancer cohort if they received medication solely or sometimes indicated for cancer. Data on gender and race or ethnicity were not collected. Non-duplicate and non-contaminant pathogens collected from various samples (ie, blood, intra-abdominal, respiratory, urine, skin or wound, and other) in outpatients were used to assess the coprimary outcomes: overall and source-specific proportions of non-susceptible pathogen isolates with corresponding AMR odds ratios (ORs); and rates of AMR pathogens per 1000 isolates with corresponding AMR incidence rate ratio (IRR) in patients with and without cancer. Findings Data were collected between April 1, 2018, and Dec 31, 2022. 53 006 (3 center dot 2%) of 1 655 594 pathogens identified were from 27 421 patients with cancer and 1 602 588 (96 center dot 8%) were from 928 128 patients without cancer. For Pseudomonas aeruginosa, carbapenem non-susceptibility was higher in pathogen isolates from patients with cancer (816 [14 center dot 4%] of 5683) than patients without cancer (10 709 [11 center dot 3%] 94 419; OR 1 center dot 22 [95% CI 1 center dot 13-1 center dot 32]). For Enterobacterales, fluoroquinolone non-susceptibility was higher in pathogen isolates from patients with cancer (8662 [28 center dot 0%] of 30 867) than patients without cancer (238 479 [21 center dot 8%] of 1 095 996; OR 1 center dot 44 [1 center dot 40-1 center dot 47]), as was carbapenem non-susceptibility (472 [1 center dot 5%] of 30 867 vs 9165 [0 center dot 8%] of 1 095 996; OR 1 center dot 89 [1 center dot 72-2 center dot 07]), multidrug-resistant pathogens (2672 [8 center dot 7%] of 30 867 vs 48 962 [4 center dot 5%] of 1 095 996; OR 2 center dot 03 [1 center dot 95-2 center dot 11]), and extended-spectrum beta-lactamase producers (4343 [16 center dot 5%] of 26 327 vs 93 977 [9 center dot 4%] of 996 853; OR 1 center dot 96 [1 center dot 90-2 center dot 03]). For Staphylococcus aureus, meticillin resistance was higher in pathogen isolates from patients with cancer (4747 [53 center dot 0%] of 8959) than patients without cancer (129 291 [48 center dot 3%] of 267 520; OR 1 center dot 20 [1 center dot 15-1 center dot 25]). For Enterococcus spp, vancomycin resistance was higher in pathogen isolates from patients with cancer (1329 [18 center dot 6%] of 7145) than patients without cancer (12 333 [9 center dot 1%] of 135 772]; ORR 2 center dot 20 [2 center dot 06-2 center dot 34). The rates and corresponding IRRs of AMR pathogens per 1000 isolates was also higher in patients with cancer compared with patients without cancer, particularly for carbapenem non-susceptible P aeruginosa (IRR 2 center dot 06 [1 center dot 91-2 center dot 21]) and vancomycin-resistant enterococci (IRR 3 center dot 06 [2 center dot 89-3 center dot 24]). For all comparisons, p<0 center dot 0001. Interpretation AMR proportions and IRRs for most key pathogens were up to three-times higher in isolates from outpatients with cancer than those without cancer, highlighting the need for enhanced surveillance, infection prevention, and timely diagnostic stewardship to improve antibiotic prescribing in this population. Copyright (c) 2025 Elsevier Ltd. All rights reserved, including those for text and data mining, AI training, and similar technologies. | Infections kill many people with cancer, often due to bacteria that resist common drugs. But we don't know much about this problem in patients who get care outside hospitals. This study looked back at health records from over 1.6 million bacteria samples in U.S. outpatient clinics from 2018 to 2022. It compared drug resistance in adults with cancer to those without. Researchers found that resistant germs were much more common in cancer patients—up to three times higher for some types, like those fighting strong antibiotics. This means cancer outpatients face bigger risks from hard-to-treat infections. The results call for better tracking, prevention steps, and smarter drug choices to help these patients live longer and healthier. Scientists can use this to guide future work on fighting drug-resistant bugs in vulnerable groups. | People with cancer often die from infections caused by bacteria that resist antibiotics, but we don't know much about this problem in those getting care outside hospitals. This study looked at why this research matters: to compare how often these tough bacteria show up in outpatients with cancer versus those without. Researchers used a big US database from 2018 to 2022, checking over 1.6 million bacteria samples from adults in clinics. They found that cancer patients had up to three times more resistant germs, like those not killed by common drugs, in urine, blood, and other body parts. These results could help doctors watch for dangers better, prevent infections, and use antibiotics smarter. For everyday people, this means safer care for cancer fighters, cutting risks and saving lives. For scientists, it pushes for more studies on protecting this group. | People with cancer often get serious infections from bacteria that resist antibiotics, which is a big problem but not well studied in those getting care outside hospitals. This study looked back at health records from over 1 million adults in the US from 2018 to 2022 to compare how often these resistant germs appear in people with and without cancer. Researchers checked bacteria from blood, urine, wounds, and other samples, grouping patients by whether they took cancer drugs. They found that resistant bacteria, like those not killed by common antibiotics such as carbapenems or vancomycin, were up to three times more common in cancer patients. This means doctors need better ways to watch for, prevent, and treat these infections wisely to save lives and stop the spread of superbugs in everyday medical care. |
| CancerRes | 5 | 10.1016/S1470-2045(25)00006-4 | Open AI | Background The phase 2 randomised Young-PEARL study demonstrated that palbociclib plus exemestane with ovarian function suppression significantly prolonged progression-free survival compared with capecitabine in premenopausal women with hormone receptor-positive, HER2-negative metastatic breast cancer. Here, we report results of the protocol-specified secondary endpoint of overall survival. Methods Young-PEARL was a multicentre, randomised, open-label, phase 2 study conducted at 14 institutions in South Korea. Premenopausal women aged 19 years or older with histologically confirmed hormone receptor-positive, HER2-negative metastatic breast cancer that recurred or progressed during or after previous tamoxifen treatment, who were aromatase inhibitor naive, and had an Eastern Cooperative Oncology Group performance status of 0-2 were eligible. One previous line of chemotherapy was permitted in the metastatic setting. Eligible patients were randomly assigned (1:1), using block randomisation (block size of two) stratified by previous chemotherapy for metastatic breast cancer and presence of visceral metastasis, to receive either palbociclib (orally, 125 mg per day on a 3-weeks-on, 1-week off schedule) plus exemestane (orally 25 mg daily) with leuprorelin (subcutaneously 375 mg on day 1 of each 28-day cycle) or capecitabine (orally, 1250 mg/m(2) twice a day on a 2-weeks-on, 1-week-off schedule) until disease progression or unacceptable toxicity). The primary endpoint was progression-free survival. Overall survival was a secondary endpoint. All analyses were done in the modified intention-to-treat population (ie, included all patients randomly assigned to treatment who had at least one post-baseline CT scan and excluded those who did not receive study medication and who had any major violation of the eligible criteria). Safety was assessed in all patients who received any study treatment. This study is registered with ClinicalTrials.gov, NCT02592746, and is now complete. Findings Between June 15, 2016, and Dec 10, 2018, 189 patients were enrolled. 184 patients were randomly assigned to the palbociclib plus endocrine therapy group (n=92) or the capecitabine group (n=92), of whom 174 were included in the modified intention-to-treat population (n=90 in the palbociclib plus endocrine therapy group and n=84 in the capecitabine group). All patients were female and ethnicity data were not collected. As of data cutoff (Feb 29, 2024), median follow-up was 540 months (IQR 341-744). Median progression-free survival was 195 months (90% CI 143-222) for palbociclib plus endocrine therapy and 140 months (117-187) for capecitabine (hazard ratio 074 [90% CI 057-098]; one-sided log-rank p=0036). 52 (58%) of 90 patients in the palbociclib plus endocrine therapy group and 48 (57%) of 84 in the capecitabine group died, with a median overall survival of 548 months (95% CI 489-771) in the palbociclib plus endocrine therapy group versus 578 months (463-892) in the capecitabine group (hazard ratio 102 [95% CI 069-151]; p=092). The most common grade 3 or worse adverse event was neutropenia (59 [64%] of 92 in the palbociclib plus endocrine therapy group vs 15 [18%] of 85 in the capecitabine group) . No treatment-related deaths occurred. Interpretation With extended follow-up, palbociclib plus exemestane with ovarian function suppression continued to show a significant benefit in progression-free survival compared with capecitabine in premenopausal patients with hormone receptor-positive, HER2-negative metastatic breast cancer who had been previously treated with tamoxifen; however, no improvement in overall survival was seen. Given the progression-free survival benefit, the upfront use of palbociclib plus endocrine therapy is the preferred option for premenopausal women, although a capecitabine-first strategy might be an alternative treatment strategy for maintaining overall survival in resource-limited settings. Copyright (c) 2025 Elsevier Ltd. All rights reserved, including those for text and data mining, AI training, and similar technologies. | This study compared two treatment approaches for younger women who had breast cancer that had spread and tested positive for hormone receptors but negative for HER2. All women had already been treated with tamoxifen. One group received a targeted medicine called palbociclib with hormone therapy and ovarian suppression, while the other group received chemotherapy with capecitabine. Results showed that the palbociclib combination delayed cancer growth for longer than chemotherapy. However, overall survival time was similar between the two treatments. Side effects were different: palbociclib caused more low white blood cell counts, while capecitabine had other typical chemotherapy side effects. These findings suggest that palbociclib with hormone therapy is a strong first treatment option for these patients, especially when delaying cancer progression is important. In places where resources are limited, starting with chemotherapy may still be reasonable since overall survival was not reduced. | This study looked at two treatment options for younger women who have breast cancer that has spread and is sensitive to hormones but does not have the HER2 marker. All women had already been treated with tamoxifen. One group received palbociclib with hormone therapy and medicines to stop ovarian function. The other group received the chemotherapy drug capecitabine. Researchers found that the palbociclib combination kept the cancer from growing for longer than capecitabine, but it did not help women live longer overall. Side effects were different, with more low white blood cell counts in the palbociclib group. These results suggest that starting treatment with palbociclib plus hormone therapy may be a good choice for women in similar situations, while capecitabine may still be used when resources are limited. This information can guide doctors and patients in making treatment decisions. | This study looked at two treatment options for younger women who still have menstrual cycles and have a type of advanced breast cancer that grows in response to hormones but does not have excess HER2 protein. All women had tried tamoxifen before, but their cancer had returned or worsened. One group received palbociclib with hormone-blocking medicines, and the other group received the chemotherapy drug capecitabine. After more than four years of follow-up, the palbociclib group’s cancer took longer to grow compared to the capecitabine group. However, the length of life was similar in both groups. Side effects differed, with more cases of low white blood cells in the palbociclib group. These results suggest palbociclib plus hormone therapy may be a better first choice to delay cancer growth, while capecitabine could still be useful in places with fewer resources. |
| CancerRes | 5 | 10.1016/S1470-2045(25)00006-5 | Claude | Background The phase 2 randomised Young-PEARL study demonstrated that palbociclib plus exemestane with ovarian function suppression significantly prolonged progression-free survival compared with capecitabine in premenopausal women with hormone receptor-positive, HER2-negative metastatic breast cancer. Here, we report results of the protocol-specified secondary endpoint of overall survival. Methods Young-PEARL was a multicentre, randomised, open-label, phase 2 study conducted at 14 institutions in South Korea. Premenopausal women aged 19 years or older with histologically confirmed hormone receptor-positive, HER2-negative metastatic breast cancer that recurred or progressed during or after previous tamoxifen treatment, who were aromatase inhibitor naive, and had an Eastern Cooperative Oncology Group performance status of 0-2 were eligible. One previous line of chemotherapy was permitted in the metastatic setting. Eligible patients were randomly assigned (1:1), using block randomisation (block size of two) stratified by previous chemotherapy for metastatic breast cancer and presence of visceral metastasis, to receive either palbociclib (orally, 125 mg per day on a 3-weeks-on, 1-week off schedule) plus exemestane (orally 25 mg daily) with leuprorelin (subcutaneously 375 mg on day 1 of each 28-day cycle) or capecitabine (orally, 1250 mg/m(2) twice a day on a 2-weeks-on, 1-week-off schedule) until disease progression or unacceptable toxicity). The primary endpoint was progression-free survival. Overall survival was a secondary endpoint. All analyses were done in the modified intention-to-treat population (ie, included all patients randomly assigned to treatment who had at least one post-baseline CT scan and excluded those who did not receive study medication and who had any major violation of the eligible criteria). Safety was assessed in all patients who received any study treatment. This study is registered with ClinicalTrials.gov, NCT02592746, and is now complete. Findings Between June 15, 2016, and Dec 10, 2018, 189 patients were enrolled. 184 patients were randomly assigned to the palbociclib plus endocrine therapy group (n=92) or the capecitabine group (n=92), of whom 174 were included in the modified intention-to-treat population (n=90 in the palbociclib plus endocrine therapy group and n=84 in the capecitabine group). All patients were female and ethnicity data were not collected. As of data cutoff (Feb 29, 2024), median follow-up was 540 months (IQR 341-744). Median progression-free survival was 195 months (90% CI 143-222) for palbociclib plus endocrine therapy and 140 months (117-187) for capecitabine (hazard ratio 074 [90% CI 057-098]; one-sided log-rank p=0036). 52 (58%) of 90 patients in the palbociclib plus endocrine therapy group and 48 (57%) of 84 in the capecitabine group died, with a median overall survival of 548 months (95% CI 489-771) in the palbociclib plus endocrine therapy group versus 578 months (463-892) in the capecitabine group (hazard ratio 102 [95% CI 069-151]; p=092). The most common grade 3 or worse adverse event was neutropenia (59 [64%] of 92 in the palbociclib plus endocrine therapy group vs 15 [18%] of 85 in the capecitabine group) . No treatment-related deaths occurred. Interpretation With extended follow-up, palbociclib plus exemestane with ovarian function suppression continued to show a significant benefit in progression-free survival compared with capecitabine in premenopausal patients with hormone receptor-positive, HER2-negative metastatic breast cancer who had been previously treated with tamoxifen; however, no improvement in overall survival was seen. Given the progression-free survival benefit, the upfront use of palbociclib plus endocrine therapy is the preferred option for premenopausal women, although a capecitabine-first strategy might be an alternative treatment strategy for maintaining overall survival in resource-limited settings. Copyright (c) 2025 Elsevier Ltd. All rights reserved, including those for text and data mining, AI training, and similar technologies. | Breast cancer that spreads to other parts of the body is harder to treat, especially in younger women before menopause. Researchers wanted to find the best treatment for women with a type of breast cancer that needs hormones to grow. They compared two different treatments in 184 women from South Korea. One group received palbociclib pills combined with hormone-blocking drugs, while the other group received capecitabine chemotherapy pills. The study followed patients for over four years to see how long they lived and how long their cancer stayed under control. Women who received palbociclib had their cancer controlled for about five and a half months longer than those who received chemotherapy. However, both groups lived about the same amount of time overall. The palbociclib treatment caused more blood count problems but was generally safe. These results help doctors choose the best treatment for young women with this type of breast cancer. | Breast cancer that spreads to other parts of the body is harder to treat, especially in younger women before menopause. This study tested two different treatments for 184 women in South Korea with a specific type of breast cancer that feeds on hormones. Half the women got a combination of three drugs: palbociclib (a newer cancer pill), exemestane (a hormone blocker), and leuprorelin (a shot that stops ovaries from making hormones). The other half got capecitabine, a chemotherapy pill. Researchers wanted to see which treatment worked better and helped women live longer. They followed these women for over four years. The combination treatment delayed cancer growth for about 5.5 months longer than chemotherapy alone. However, both treatments helped women live about the same length of time overall. The combination caused more low white blood cell counts but was still safe. These results help doctors choose the best first treatment for younger women with this type of breast cancer. | Breast cancer that spreads to other parts of the body is harder to treat, especially in younger women before menopause. This study tested two different treatments for 184 young women whose breast cancer had a specific type called hormone receptor-positive, HER2-negative metastatic breast cancer. The researchers wanted to see which treatment worked better and helped women live longer. One group received a combination of three drugs: palbociclib, exemestane, and leuprorelin. The other group received a single drug called capecitabine. The study followed patients for over four years. Results showed that women who got the three-drug combination went longer without their cancer getting worse compared to those who got the single drug. However, both groups lived about the same amount of time overall. The three-drug treatment caused more side effects, especially low white blood cell counts. These findings help doctors choose the best treatment for young women with this type of breast cancer. |
| CancerRes | 5 | 10.1016/S1470-2045(25)00006-6 | Gemini | Background The phase 2 randomised Young-PEARL study demonstrated that palbociclib plus exemestane with ovarian function suppression significantly prolonged progression-free survival compared with capecitabine in premenopausal women with hormone receptor-positive, HER2-negative metastatic breast cancer. Here, we report results of the protocol-specified secondary endpoint of overall survival. Methods Young-PEARL was a multicentre, randomised, open-label, phase 2 study conducted at 14 institutions in South Korea. Premenopausal women aged 19 years or older with histologically confirmed hormone receptor-positive, HER2-negative metastatic breast cancer that recurred or progressed during or after previous tamoxifen treatment, who were aromatase inhibitor naive, and had an Eastern Cooperative Oncology Group performance status of 0-2 were eligible. One previous line of chemotherapy was permitted in the metastatic setting. Eligible patients were randomly assigned (1:1), using block randomisation (block size of two) stratified by previous chemotherapy for metastatic breast cancer and presence of visceral metastasis, to receive either palbociclib (orally, 125 mg per day on a 3-weeks-on, 1-week off schedule) plus exemestane (orally 25 mg daily) with leuprorelin (subcutaneously 375 mg on day 1 of each 28-day cycle) or capecitabine (orally, 1250 mg/m(2) twice a day on a 2-weeks-on, 1-week-off schedule) until disease progression or unacceptable toxicity). The primary endpoint was progression-free survival. Overall survival was a secondary endpoint. All analyses were done in the modified intention-to-treat population (ie, included all patients randomly assigned to treatment who had at least one post-baseline CT scan and excluded those who did not receive study medication and who had any major violation of the eligible criteria). Safety was assessed in all patients who received any study treatment. This study is registered with ClinicalTrials.gov, NCT02592746, and is now complete. Findings Between June 15, 2016, and Dec 10, 2018, 189 patients were enrolled. 184 patients were randomly assigned to the palbociclib plus endocrine therapy group (n=92) or the capecitabine group (n=92), of whom 174 were included in the modified intention-to-treat population (n=90 in the palbociclib plus endocrine therapy group and n=84 in the capecitabine group). All patients were female and ethnicity data were not collected. As of data cutoff (Feb 29, 2024), median follow-up was 540 months (IQR 341-744). Median progression-free survival was 195 months (90% CI 143-222) for palbociclib plus endocrine therapy and 140 months (117-187) for capecitabine (hazard ratio 074 [90% CI 057-098]; one-sided log-rank p=0036). 52 (58%) of 90 patients in the palbociclib plus endocrine therapy group and 48 (57%) of 84 in the capecitabine group died, with a median overall survival of 548 months (95% CI 489-771) in the palbociclib plus endocrine therapy group versus 578 months (463-892) in the capecitabine group (hazard ratio 102 [95% CI 069-151]; p=092). The most common grade 3 or worse adverse event was neutropenia (59 [64%] of 92 in the palbociclib plus endocrine therapy group vs 15 [18%] of 85 in the capecitabine group) . No treatment-related deaths occurred. Interpretation With extended follow-up, palbociclib plus exemestane with ovarian function suppression continued to show a significant benefit in progression-free survival compared with capecitabine in premenopausal patients with hormone receptor-positive, HER2-negative metastatic breast cancer who had been previously treated with tamoxifen; however, no improvement in overall survival was seen. Given the progression-free survival benefit, the upfront use of palbociclib plus endocrine therapy is the preferred option for premenopausal women, although a capecitabine-first strategy might be an alternative treatment strategy for maintaining overall survival in resource-limited settings. Copyright (c) 2025 Elsevier Ltd. All rights reserved, including those for text and data mining, AI training, and similar technologies. | This study, named Young-PEARL, looked at a new drug combination for premenopausal women with a specific type of advanced breast cancer. The goal was to see if the new treatment, which combines palbociclib and endocrine therapy, was better than a standard chemotherapy drug, capecitabine.  The research found that the new drug combination significantly delayed the cancer's progression, meaning it took longer for the disease to get worse. However, when the researchers looked at how long patients lived, they found that both treatments led to similar overall survival times. The new treatment caused more side effects, specifically a drop in white blood cells, but there were no treatment-related deaths in either group.  Because the palbociclib combination delayed the cancer's progression, doctors may prefer to use it as a first treatment. However, since both treatments resulted in similar survival times, using capecitabine first could be a valid alternative, especially in places where the new treatment is not easily accessible. | This study was conducted to see if a drug combination of palbociclib and a hormone therapy works better than another chemotherapy drug, capecitabine, for premenopausal women with a specific type of advanced breast cancer. The research found that the palbociclib combination helped patients live longer without their cancer getting worse, compared to capecitabine. However, after a longer follow-up period, both groups of patients lived for about the same total amount of time. The findings suggest that while the palbociclib combination can delay the cancer's progression, both treatments result in a similar long-term survival. This information is important for doctors and patients when deciding on the best initial treatment plan, especially in places where the cost of these drugs is a concern. | This study was conducted to find a better treatment for premenopausal women with a specific type of advanced breast cancer. The research compared two treatment plans: one using the drug palbociclib with hormone therapy, and another using chemotherapy with the drug capecitabine.  The study found that the palbociclib and hormone therapy combination significantly delayed the progression of the cancer. However, when researchers looked at how long patients lived overall, there was no major difference between the two groups. This means that while the palbociclib treatment helps to keep the disease in check for a longer period, it does not necessarily extend a patient's life more than the capecitabine treatment. The findings suggest that the palbociclib combination is a good first choice for these patients, but the capecitabine treatment could be a valuable alternative, particularly in situations where resources are limited. |
| CancerRes | 5 | 10.1016/S1470-2045(25)00006-7 | DeepSeek | Background The phase 2 randomised Young-PEARL study demonstrated that palbociclib plus exemestane with ovarian function suppression significantly prolonged progression-free survival compared with capecitabine in premenopausal women with hormone receptor-positive, HER2-negative metastatic breast cancer. Here, we report results of the protocol-specified secondary endpoint of overall survival. Methods Young-PEARL was a multicentre, randomised, open-label, phase 2 study conducted at 14 institutions in South Korea. Premenopausal women aged 19 years or older with histologically confirmed hormone receptor-positive, HER2-negative metastatic breast cancer that recurred or progressed during or after previous tamoxifen treatment, who were aromatase inhibitor naive, and had an Eastern Cooperative Oncology Group performance status of 0-2 were eligible. One previous line of chemotherapy was permitted in the metastatic setting. Eligible patients were randomly assigned (1:1), using block randomisation (block size of two) stratified by previous chemotherapy for metastatic breast cancer and presence of visceral metastasis, to receive either palbociclib (orally, 125 mg per day on a 3-weeks-on, 1-week off schedule) plus exemestane (orally 25 mg daily) with leuprorelin (subcutaneously 375 mg on day 1 of each 28-day cycle) or capecitabine (orally, 1250 mg/m(2) twice a day on a 2-weeks-on, 1-week-off schedule) until disease progression or unacceptable toxicity). The primary endpoint was progression-free survival. Overall survival was a secondary endpoint. All analyses were done in the modified intention-to-treat population (ie, included all patients randomly assigned to treatment who had at least one post-baseline CT scan and excluded those who did not receive study medication and who had any major violation of the eligible criteria). Safety was assessed in all patients who received any study treatment. This study is registered with ClinicalTrials.gov, NCT02592746, and is now complete. Findings Between June 15, 2016, and Dec 10, 2018, 189 patients were enrolled. 184 patients were randomly assigned to the palbociclib plus endocrine therapy group (n=92) or the capecitabine group (n=92), of whom 174 were included in the modified intention-to-treat population (n=90 in the palbociclib plus endocrine therapy group and n=84 in the capecitabine group). All patients were female and ethnicity data were not collected. As of data cutoff (Feb 29, 2024), median follow-up was 540 months (IQR 341-744). Median progression-free survival was 195 months (90% CI 143-222) for palbociclib plus endocrine therapy and 140 months (117-187) for capecitabine (hazard ratio 074 [90% CI 057-098]; one-sided log-rank p=0036). 52 (58%) of 90 patients in the palbociclib plus endocrine therapy group and 48 (57%) of 84 in the capecitabine group died, with a median overall survival of 548 months (95% CI 489-771) in the palbociclib plus endocrine therapy group versus 578 months (463-892) in the capecitabine group (hazard ratio 102 [95% CI 069-151]; p=092). The most common grade 3 or worse adverse event was neutropenia (59 [64%] of 92 in the palbociclib plus endocrine therapy group vs 15 [18%] of 85 in the capecitabine group) . No treatment-related deaths occurred. Interpretation With extended follow-up, palbociclib plus exemestane with ovarian function suppression continued to show a significant benefit in progression-free survival compared with capecitabine in premenopausal patients with hormone receptor-positive, HER2-negative metastatic breast cancer who had been previously treated with tamoxifen; however, no improvement in overall survival was seen. Given the progression-free survival benefit, the upfront use of palbociclib plus endocrine therapy is the preferred option for premenopausal women, although a capecitabine-first strategy might be an alternative treatment strategy for maintaining overall survival in resource-limited settings. Copyright (c) 2025 Elsevier Ltd. All rights reserved, including those for text and data mining, AI training, and similar technologies. | This study compared two treatments for premenopausal women with advanced breast cancer that grows in response to hormones (HR+) and does not involve the HER2 protein. Researchers tested whether a combination of palbociclib (a targeted drug) plus hormone therapy worked better than capecitabine (a chemotherapy drug) at helping patients live longer. While the palbociclib combo delayed cancer growth longer (19.5 vs. 14 months), both treatments allowed patients to live for a similar amount of time overall (around 55–58 months). Side effects differed, with palbociclib causing more low white blood cell counts. The findings suggest that palbociclib plus hormone therapy is a good first choice to control cancer longer, but capecitabine remains an option, especially where cost or resources are limited. | This study compared two treatments for premenopausal women with advanced breast cancer that is hormone-sensitive but HER2-negative. One group received palbociclib (a targeted drug) plus hormone therapy, while the other took capecitabine (chemotherapy). After about 4.5 years of follow-up, the palbociclib combo helped delay cancer growth longer (19.5 vs. 14 months) but did not extend overall survival (about 55 vs. 58 months). Side effects differed—palbociclib caused more low white blood cell counts, while capecitabine had fewer severe effects. Though survival was similar, palbociclib with hormone therapy is often preferred first because it slows cancer progression better. However, capecitabine remains a useful option where resources are limited. This helps doctors and patients choose treatments based on priorities like delaying cancer growth or managing side effects. | This study compared two treatments for premenopausal women with advanced breast cancer that grows in response to hormones (HR-positive, HER2-negative). Researchers tested whether a combination of palbociclib (a targeted drug) plus hormone therapy worked better than capecitabine (a chemotherapy drug) in helping patients live longer without their cancer worsening. While the palbociclib combo delayed cancer growth longer (19.5 vs. 14 months), both treatments allowed women to live for a similar amount of time overall (around 55-58 months). Side effects differed—palbociclib caused more low white blood cell counts, but capecitabine had other risks. The results suggest that palbociclib with hormone therapy may be a better first choice to control cancer longer, but capecitabine remains an option where resources are limited. This helps doctors and patients choose treatments based on their priorities. |
| CancerRes | 5 | 10.1016/S1470-2045(25)00006-8 | Grok | Background The phase 2 randomised Young-PEARL study demonstrated that palbociclib plus exemestane with ovarian function suppression significantly prolonged progression-free survival compared with capecitabine in premenopausal women with hormone receptor-positive, HER2-negative metastatic breast cancer. Here, we report results of the protocol-specified secondary endpoint of overall survival. Methods Young-PEARL was a multicentre, randomised, open-label, phase 2 study conducted at 14 institutions in South Korea. Premenopausal women aged 19 years or older with histologically confirmed hormone receptor-positive, HER2-negative metastatic breast cancer that recurred or progressed during or after previous tamoxifen treatment, who were aromatase inhibitor naive, and had an Eastern Cooperative Oncology Group performance status of 0-2 were eligible. One previous line of chemotherapy was permitted in the metastatic setting. Eligible patients were randomly assigned (1:1), using block randomisation (block size of two) stratified by previous chemotherapy for metastatic breast cancer and presence of visceral metastasis, to receive either palbociclib (orally, 125 mg per day on a 3-weeks-on, 1-week off schedule) plus exemestane (orally 25 mg daily) with leuprorelin (subcutaneously 375 mg on day 1 of each 28-day cycle) or capecitabine (orally, 1250 mg/m(2) twice a day on a 2-weeks-on, 1-week-off schedule) until disease progression or unacceptable toxicity). The primary endpoint was progression-free survival. Overall survival was a secondary endpoint. All analyses were done in the modified intention-to-treat population (ie, included all patients randomly assigned to treatment who had at least one post-baseline CT scan and excluded those who did not receive study medication and who had any major violation of the eligible criteria). Safety was assessed in all patients who received any study treatment. This study is registered with ClinicalTrials.gov, NCT02592746, and is now complete. Findings Between June 15, 2016, and Dec 10, 2018, 189 patients were enrolled. 184 patients were randomly assigned to the palbociclib plus endocrine therapy group (n=92) or the capecitabine group (n=92), of whom 174 were included in the modified intention-to-treat population (n=90 in the palbociclib plus endocrine therapy group and n=84 in the capecitabine group). All patients were female and ethnicity data were not collected. As of data cutoff (Feb 29, 2024), median follow-up was 540 months (IQR 341-744). Median progression-free survival was 195 months (90% CI 143-222) for palbociclib plus endocrine therapy and 140 months (117-187) for capecitabine (hazard ratio 074 [90% CI 057-098]; one-sided log-rank p=0036). 52 (58%) of 90 patients in the palbociclib plus endocrine therapy group and 48 (57%) of 84 in the capecitabine group died, with a median overall survival of 548 months (95% CI 489-771) in the palbociclib plus endocrine therapy group versus 578 months (463-892) in the capecitabine group (hazard ratio 102 [95% CI 069-151]; p=092). The most common grade 3 or worse adverse event was neutropenia (59 [64%] of 92 in the palbociclib plus endocrine therapy group vs 15 [18%] of 85 in the capecitabine group) . No treatment-related deaths occurred. Interpretation With extended follow-up, palbociclib plus exemestane with ovarian function suppression continued to show a significant benefit in progression-free survival compared with capecitabine in premenopausal patients with hormone receptor-positive, HER2-negative metastatic breast cancer who had been previously treated with tamoxifen; however, no improvement in overall survival was seen. Given the progression-free survival benefit, the upfront use of palbociclib plus endocrine therapy is the preferred option for premenopausal women, although a capecitabine-first strategy might be an alternative treatment strategy for maintaining overall survival in resource-limited settings. Copyright (c) 2025 Elsevier Ltd. All rights reserved, including those for text and data mining, AI training, and similar technologies. | This study looks at better ways to treat young women with breast cancer that has spread and responds to hormones but not to HER2 drugs. These women had already tried tamoxifen and needed new options. Researchers wanted to see if combining palbociclib with hormone therapy and ovary suppression works better than capecitabine chemotherapy for slowing cancer growth and helping patients live longer. They randomly gave the treatments to 174 women and followed them for about 4.5 years. The palbociclib mix helped patients stay cancer-free longer (19.5 months vs. 14 months), but both groups lived about the same time overall (around 55-58 months). Side effects like low white blood cells were more common with palbociclib, but no one died from treatment. These findings suggest the palbociclib combo is a top choice for delaying cancer return, while capecitabine could be a good backup in places with limited resources, helping doctors pick treatments that fit patients' needs and improve quality of life. | Breast cancer that has spread and is fueled by hormones but not HER2 protein can be hard to treat in younger women before menopause. This study aimed to compare two treatments: palbociclib combined with hormone therapy and ovary suppression versus capecitabine chemotherapy. Researchers wanted to see if the first option, which earlier showed longer time without cancer worsening, also helped patients live longer. They randomly assigned 174 women in South Korea to one group or the other and followed them for about 54 months. The palbociclib group had longer cancer control (19.5 months versus 14 months), but overall survival was similar (about 55 months for both). Side effects like low white blood cells were more common with palbociclib. These findings suggest starting with palbociclib for better disease control, but capecitabine could be a good choice in places with limited resources, helping doctors pick safer, effective options for women worldwide. | This study looked at better ways to treat young women before menopause who have a type of breast cancer that grows with hormones but not HER2, and has spread. Researchers wanted to see if using palbociclib with hormone therapy and ovary suppression helps patients live longer than using capecitabine chemotherapy. They tested this in 174 women in South Korea who had tried tamoxifen before. The results showed that palbociclib with hormone therapy delayed cancer growth by about 5 months more than capecitabine, but both groups lived about the same length of time, around 4-5 years. This means doctors can choose palbociclib first to slow the disease without harsh side effects, or start with capecitabine in places with limited resources to save lives similarly. These findings help guide safer, effective care for women worldwide. |
| CancCell | 1 | 10.1016/j.ccell.2025.03.018 | Open AI | Selecting optimal candidates for next-generation anti-human epidermal growth factor receptor 2 (HER2) antibody-drug conjugates (ADCs) remains challenging. We conduct a prespecified translational study to identify treatment biomarkers in SHR-A1811-treated HER2-positive breast cancer patients from the phase 2 neoadjuvant FASCINATE-N trial using DNA and RNA sequencing, computational pathology, and single-cell in situ spatial imaging. In the hormone receptor (HR)-negative subgroup, a higher proportion and more infiltration of immune cells (i.e., tumor-infiltrating lymphocytes [TILs]), particularly cytotoxic T cells, are associated with better treatment responses. In the HR-positive subgroup, the closeness and aggregation of HER2-strong-positive tumor cells, as opposed to a uniform distribution, are linked to a lower response rate and HER2 luminal-like (HER2-LUM) subtype, which more closely resembles HR+/HER2-breast cancer. In addition, we develop a clinically practical predictive model capable of predicting neoadjuvant treatment responses to SHR-A1811 and other novel ADCs based on clinicopathological characteristics and pathological images. | This study looks at why some people with HER2-positive breast cancer respond better than others to a new drug called SHR-A1811 before surgery. We studied tumor samples using advanced genetic tests, computer analysis of tissue images, and special cell-mapping techniques. In patients without hormone receptors, having more immune cells—especially certain white blood cells that attack cancer—was linked to better results. In patients with hormone receptors, tumors where HER2-strong cells were grouped together, rather than spread out, tended to respond less well. We also created an easy-to-use tool that can help doctors predict who is most likely to benefit from SHR-A1811 and similar drugs. These findings could guide more personalized treatment choices, helping patients get therapies that work best for them while avoiding less effective options. | This study looks at how a new targeted cancer drug, called SHR-A1811, works in people with HER2-positive breast cancer before surgery. Doctors want to find out which patients will benefit the most from this treatment. We studied tumor samples using advanced genetic tests and imaging to see how cancer cells and immune cells are arranged. In patients without hormone receptors, having more cancer-fighting immune cells was linked to better results. In patients with hormone receptors, tightly packed HER2-strong cancer cells were linked to lower success rates. Using these findings, we created an easy-to-use model that can help predict how well patients might respond to SHR-A1811 or similar new drugs. This research may help doctors choose the right patients for these treatments, improving success rates and avoiding unnecessary side effects. | This study looked at why some people with a type of breast cancer called HER2-positive respond better than others to a new drug called SHR-A1811, given before surgery. The drug is an antibody linked to a cancer-killing medicine that targets HER2-positive cancer cells. We studied tumor samples from patients using advanced genetic tests, computer-based image analysis, and special single-cell imaging. In patients whose tumors did not have hormone receptors, having more immune cells—especially cancer-fighting T cells—was linked to better responses. In patients whose tumors had hormone receptors, cancer cells grouped closely together were linked to poorer responses. From these findings, we created a practical tool to help doctors predict how well a patient may respond to SHR-A1811 and similar drugs before surgery. This research may guide more personalized treatment, so patients receive therapies most likely to work for them while avoiding ineffective options. |
| CancCell | 1 | 10.1016/j.ccell.2025.03.019 | Claude | Selecting optimal candidates for next-generation anti-human epidermal growth factor receptor 2 (HER2) antibody-drug conjugates (ADCs) remains challenging. We conduct a prespecified translational study to identify treatment biomarkers in SHR-A1811-treated HER2-positive breast cancer patients from the phase 2 neoadjuvant FASCINATE-N trial using DNA and RNA sequencing, computational pathology, and single-cell in situ spatial imaging. In the hormone receptor (HR)-negative subgroup, a higher proportion and more infiltration of immune cells (i.e., tumor-infiltrating lymphocytes [TILs]), particularly cytotoxic T cells, are associated with better treatment responses. In the HR-positive subgroup, the closeness and aggregation of HER2-strong-positive tumor cells, as opposed to a uniform distribution, are linked to a lower response rate and HER2 luminal-like (HER2-LUM) subtype, which more closely resembles HR+/HER2-breast cancer. In addition, we develop a clinically practical predictive model capable of predicting neoadjuvant treatment responses to SHR-A1811 and other novel ADCs based on clinicopathological characteristics and pathological images. | Breast cancer patients with high levels of a protein called HER2 can be treated with new targeted drugs called antibody-drug conjugates, but doctors need better ways to predict which patients will respond best to these treatments. Researchers studied patients receiving a new drug called SHR-A1811 before surgery to shrink their tumors. They used advanced computer technology to analyze tumor samples and look at how different cells were arranged within the tumors. They found that patients whose tumors had more immune cells fighting the cancer responded better to treatment, especially when hormone receptors were absent. However, when hormone receptors were present, patients whose HER2-positive cancer cells were clustered together in groups responded worse than those with evenly spread cancer cells. The clustered pattern made these tumors act more like a different, harder-to-treat type of breast cancer. Using this information, the researchers created a tool that can help doctors predict which patients will benefit most from these new targeted treatments, potentially improving treatment decisions and patient. | Breast cancer patients with high levels of a protein called HER2 can be treated with new targeted drugs called antibody-drug conjugates, but doctors need better ways to predict which patients will respond best to treatment. Researchers studied patients receiving a new drug called SHR-A1811 before surgery to see what factors make the treatment work better. They found that the response depends on hormone levels in the cancer. In patients without hormone-sensitive cancer, having more immune cells fighting the tumor led to better results. In patients with hormone-sensitive cancer, the way HER2-positive cancer cells are arranged matters - when these cells cluster together rather than spread evenly, the treatment works less well. The team also created a prediction tool using standard medical tests and images that can help doctors identify which patients are most likely to benefit from this type of treatment. This research could help doctors choose the best patients for these new targeted cancer drugs and improve treatment outcomes. | Scientists are working to find better ways to predict which breast cancer patients will respond best to a new targeted drug called SHR-A1811. This drug is designed to attack cancer cells that have high levels of a protein called HER2. The researchers studied tumor samples from 200 breast cancer patients who received this treatment before surgery. They used advanced imaging and computer analysis to examine how cancer cells and immune cells were arranged within the tumors. They discovered that the physical location and spacing of different cell types affects how well the treatment works. In patients whose tumors also had hormone receptors, the drug worked better when HER2-positive cancer cells were spread out rather than clustered together. In patients without hormone receptors, having more immune cells fighting the cancer led to better responses. Using these findings, the team created a simple prediction tool that doctors could use to identify which patients are most likely to benefit from this type of cancer treatment, potentially improving treatment outcomes. |
| CancCell | 1 | 10.1016/j.ccell.2025.03.020 | Gemini | Selecting optimal candidates for next-generation anti-human epidermal growth factor receptor 2 (HER2) antibody-drug conjugates (ADCs) remains challenging. We conduct a prespecified translational study to identify treatment biomarkers in SHR-A1811-treated HER2-positive breast cancer patients from the phase 2 neoadjuvant FASCINATE-N trial using DNA and RNA sequencing, computational pathology, and single-cell in situ spatial imaging. In the hormone receptor (HR)-negative subgroup, a higher proportion and more infiltration of immune cells (i.e., tumor-infiltrating lymphocytes [TILs]), particularly cytotoxic T cells, are associated with better treatment responses. In the HR-positive subgroup, the closeness and aggregation of HER2-strong-positive tumor cells, as opposed to a uniform distribution, are linked to a lower response rate and HER2 luminal-like (HER2-LUM) subtype, which more closely resembles HR+/HER2-breast cancer. In addition, we develop a clinically practical predictive model capable of predicting neoadjuvant treatment responses to SHR-A1811 and other novel ADCs based on clinicopathological characteristics and pathological images. | This study looks at a new drug, SHR-A1811, which combines an antibody with a cancer-fighting medicine to treat a type of breast cancer called HER2-positive. The main goal of this research is to figure out which patients will benefit most from this drug. We found that for some patients, having more immune cells in the tumor meant the treatment worked better. For others, how the cancer cells were arranged—whether they were close together or spread out—helped predict if the treatment would be successful. We also created a new tool that uses patient information and pictures of their tumors to better predict how they will respond to this and similar drugs. This work helps doctors choose the right treatment for each patient. | This research explores how a new drug, SHR-A1811, works in patients with HER2-positive breast cancer. The study aims to discover who will benefit most from this drug by looking at the details of their tumors. We found that patients with hormone receptor-negative tumors who have more immune cells surrounding the cancer cells respond better to treatment. In contrast, patients with hormone receptor-positive tumors tend to have a lower response if their HER2-positive cells are clustered together. Based on these findings, we developed a tool that uses patient information and tumor images to predict how well a patient might respond to this new drug. This will help doctors choose the best treatment for each patient. | Antibody-drug conjugates, like SHR-A1811, are a new type of medicine that helps fight cancer. However, it is not yet clear which patients with HER2-positive breast cancer will benefit the most from them. This study looked at breast cancer tissue to find out why some patients respond better to SHR-A1811 than others. The researchers found that in a certain type of breast cancer, a stronger immune response with more immune cells in the tumor was linked to better treatment results. For another type, having cancer cells that are clumped together instead of spread out was connected to a poorer response. This research helps us better understand which patients might be the best candidates for this treatment. It also provides a new way to predict how patients will respond using standard clinical and imaging information, which could help doctors choose the best treatment plan. |
| CancCell | 1 | 10.1016/j.ccell.2025.03.021 | DeepSeek | Selecting optimal candidates for next-generation anti-human epidermal growth factor receptor 2 (HER2) antibody-drug conjugates (ADCs) remains challenging. We conduct a prespecified translational study to identify treatment biomarkers in SHR-A1811-treated HER2-positive breast cancer patients from the phase 2 neoadjuvant FASCINATE-N trial using DNA and RNA sequencing, computational pathology, and single-cell in situ spatial imaging. In the hormone receptor (HR)-negative subgroup, a higher proportion and more infiltration of immune cells (i.e., tumor-infiltrating lymphocytes [TILs]), particularly cytotoxic T cells, are associated with better treatment responses. In the HR-positive subgroup, the closeness and aggregation of HER2-strong-positive tumor cells, as opposed to a uniform distribution, are linked to a lower response rate and HER2 luminal-like (HER2-LUM) subtype, which more closely resembles HR+/HER2-breast cancer. In addition, we develop a clinically practical predictive model capable of predicting neoadjuvant treatment responses to SHR-A1811 and other novel ADCs based on clinicopathological characteristics and pathological images. | This study explores why some HER2-positive breast cancer patients respond better to a new drug called SHR-A1811 before surgery. Researchers examined tumors using advanced imaging and genetic tests to find clues. They discovered that in hormone receptor (HR)-negative tumors, more immune cells inside the tumor—especially those that attack cancer—led to better treatment results. In HR-positive tumors, how HER2-positive cancer cells were grouped together mattered; clustered cells responded poorly, especially in a subtype resembling HR+/HER2- breast cancer. The team also created a tool to predict which patients will benefit from this drug and similar treatments. These findings could help doctors personalize therapy, improving outcomes for breast cancer patients. | This study looked at how well a new drug (SHR-A1811) works for HER2-positive breast cancer before surgery and what factors influence its success. Researchers analyzed tumors from patients using advanced imaging and genetic tests. They found that in hormone receptor-negative cancers, having more immune cells (especially those that attack tumors) helped the drug work better. In hormone receptor-positive cancers, the drug was less effective when HER2-positive tumor cells were clustered together instead of spread out. The team also created a tool to predict which patients would respond best to this drug and similar treatments. These findings could help doctors personalize therapy, giving the right drug to the right patient for better outcomes. | This study explores why some HER2-positive breast cancer patients respond better to a new drug called SHR-A1811, an antibody-drug conjugate (ADC). Researchers analyzed tumors from patients receiving the drug before surgery, using advanced imaging and genetic tests. They found that in hormone receptor-negative tumors, more immune cells—especially those that attack cancer—were linked to better treatment results. In hormone receptor-positive tumors, how HER2-positive cancer cells were clustered (not spread evenly) predicted a weaker response, especially in a subtype resembling other hard-to-treat breast cancers. The team also created a tool to help doctors predict which patients might benefit from this drug and similar future treatments. These findings could improve personalized care for HER2-positive breast cancer by matching the right patients to the most effective therapies. |
| CancCell | 1 | 10.1016/j.ccell.2025.03.022 | Grok | Selecting optimal candidates for next-generation anti-human epidermal growth factor receptor 2 (HER2) antibody-drug conjugates (ADCs) remains challenging. We conduct a prespecified translational study to identify treatment biomarkers in SHR-A1811-treated HER2-positive breast cancer patients from the phase 2 neoadjuvant FASCINATE-N trial using DNA and RNA sequencing, computational pathology, and single-cell in situ spatial imaging. In the hormone receptor (HR)-negative subgroup, a higher proportion and more infiltration of immune cells (i.e., tumor-infiltrating lymphocytes [TILs]), particularly cytotoxic T cells, are associated with better treatment responses. In the HR-positive subgroup, the closeness and aggregation of HER2-strong-positive tumor cells, as opposed to a uniform distribution, are linked to a lower response rate and HER2 luminal-like (HER2-LUM) subtype, which more closely resembles HR+/HER2-breast cancer. In addition, we develop a clinically practical predictive model capable of predicting neoadjuvant treatment responses to SHR-A1811 and other novel ADCs based on clinicopathological characteristics and pathological images. | Breast cancer with high levels of a protein called HER2 can be hard to treat, and picking the right patients for new drugs like SHR-A1811 is tricky. This study looks at why some patients respond better to this drug before surgery. Researchers used gene tests, computer analysis of tissue images, and close-up views of cells to study tumor samples from a trial. They found that in patients without hormone receptors, more immune cells fighting the cancer led to better results. In patients with hormone receptors, clustered HER2 cells meant poorer responses. The team created an easy-to-use tool to predict who will benefit based on patient details and images. This could help doctors choose the best treatments, improve outcomes for many women, and guide future research on similar drugs.3.1s | Breast cancer with high levels of a protein called HER2 can be hard to treat, and picking the right patients for new drugs like SHR-A1811 is tricky. This study looked at ways to find signs that predict how well the drug works before surgery. Researchers used gene tests, computer analysis of tissue images, and close-up views of cells to study tumor samples from patients in a trial. They found that in cancers without hormone receptors, more immune cells—especially killer T cells—near the tumor led to better results. In cancers with hormone receptors, clustered HER2 cells were linked to poorer responses and a subtype that acts more like non-HER2 cancers. The team created an easy-to-use model to predict outcomes for this drug and similar ones, which could help doctors choose the best treatments and improve care for people with breast cancer. | Breast cancer that tests positive for the HER2 protein can be tough to treat, and picking the right patients for new targeted drugs like SHR-A1811 is a big challenge. This study aimed to find clues in tumors that show who will respond well to this drug before surgery. Researchers used gene tests, computer analysis of tissue slides, and detailed cell imaging on samples from a clinical trial. They found that in patients without hormone receptors, more immune cells fighting the cancer led to better results. But in those with hormone receptors, clustered HER2 cells meant poorer responses and a cancer type more like hormone-positive cases. The team built an easy-to-use model to predict outcomes based on patient traits and images. These insights could help doctors choose the best treatments, improve success rates, and guide future research on similar drugs for more people. |
| CancCell | 2 | 10.1016/j.ccell.2025.03.028 | Open AI | Circular RNA (circRNA) is a class of noncoding RNA with regulatory potentials. Its role in the transdifferentiation of prostate and lung adenocarcinoma into neuroendocrine prostate cancer (NEPC) and small cell lung cancer (SCLC) remains unexplored. Here, we identified circRMST as an exceptionally abundant circRNA predominantly expressed in NEPC and SCLC, with strong conservation between humans and mice. Functional studies using shRNA, siRNA, CRISPR-Cas13, and Cas9 consistently demonstrate that circRMST is essential for tumor growth and the expression of ASCL1, a master regulator of neuroendocrine fate. Genetic knockout of Rmst in NEPC genetic engineered mouse models prevents neuroendocrine transdifferentiation, maintaining tumors in an adenocarcinoma state. Mechanistically, circRMST physically interacts with lineage transcription factors NKX2-1 and SOX2. Loss of circRMST induces NKX2-1 protein degradation through autophagy-lysosomal pathway and alters the genomic binding of SOX2, collectively leading to the loss of ASCL1 transcription. | Some prostate and lung cancers can change into a more aggressive type called neuroendocrine cancer, but how this happens is not well understood. In this study, we discovered a circular RNA called circRMST that is found at very high levels in these aggressive cancers in both people and mice. Using different genetic tools, we showed that circRMST is needed for tumor growth and for making ASCL1, a key protein that drives neuroendocrine cell changes. Removing circRMST in mouse models stopped the cancer from changing into the neuroendocrine type. We also found that circRMST works by controlling two important proteins, NKX2-1 and SOX2. Our findings reveal a new molecule that is essential for this cancer change, which may help scientists design new ways to detect or treat these hard-to-treat cancers. | Some cancers, like prostate and lung cancers, can change into a more aggressive form called neuroendocrine cancer, which is harder to treat. Our research looked at a special type of genetic material called circular RNA, which does not make proteins but can still control how cells behave. We found one circular RNA, called circRMST, that is present in large amounts in aggressive neuroendocrine cancers in both humans and mice. By blocking circRMST in laboratory and animal studies, we stopped cancer cells from changing into this dangerous form and slowed their growth. We also discovered that circRMST works by helping key proteins that control cell identity stay active. Without it, these proteins break down and lose their ability to switch the cancer’s type. This research could guide new treatments that target circRMST to prevent or slow aggressive cancer changes. | This study explored a special type of RNA, called circular RNA, and its role in how some prostate and lung cancers change into more aggressive forms. The researchers discovered that one circular RNA, named circRMST, is found in high amounts in these aggressive cancers in both humans and mice. By blocking circRMST in laboratory models, the cancers could no longer switch into the more dangerous type and stayed in their original state. The team found that circRMST works by interacting with two important proteins, NKX2-1 and SOX2, which control the identity of cancer cells. Removing circRMST caused one protein to break down and changed how the other worked, stopping the chain of events that leads to aggressive cancer growth. These findings could help scientists develop new ways to prevent or slow the progression of certain cancers by targeting circRMST. |
| CancCell | 2 | 10.1016/j.ccell.2025.03.029 | Claude | Circular RNA (circRNA) is a class of noncoding RNA with regulatory potentials. Its role in the transdifferentiation of prostate and lung adenocarcinoma into neuroendocrine prostate cancer (NEPC) and small cell lung cancer (SCLC) remains unexplored. Here, we identified circRMST as an exceptionally abundant circRNA predominantly expressed in NEPC and SCLC, with strong conservation between humans and mice. Functional studies using shRNA, siRNA, CRISPR-Cas13, and Cas9 consistently demonstrate that circRMST is essential for tumor growth and the expression of ASCL1, a master regulator of neuroendocrine fate. Genetic knockout of Rmst in NEPC genetic engineered mouse models prevents neuroendocrine transdifferentiation, maintaining tumors in an adenocarcinoma state. Mechanistically, circRMST physically interacts with lineage transcription factors NKX2-1 and SOX2. Loss of circRMST induces NKX2-1 protein degradation through autophagy-lysosomal pathway and alters the genomic binding of SOX2, collectively leading to the loss of ASCL1 transcription. | Cancer cells can sometimes change from one type to another, making them harder to treat. Scientists studied a special type of genetic material called circular RNA, specifically one named circRMST, to understand how certain cancers transform. They focused on prostate and lung cancers that change into more aggressive neuroendocrine forms. The researchers found that circRMST is very abundant in these transformed cancers and is essential for their growth. Using various laboratory techniques, they discovered that circRMST works like a molecular helper, partnering with specific proteins called transcription factors to control genes that drive cancer transformation. When they removed circRMST from cancer cells and mice, the tumors could not complete their dangerous transformation and remained in their original, less aggressive state. This research is important because it reveals a new target for treating these hard-to-cure cancers. Understanding how circRMST controls cancer transformation could lead to new medicines that prevent or reverse this deadly process, potentially saving many lives. | Cancer cells can change from one type to another, making them harder to treat. Scientists studied a special piece of genetic material called circular RMST that helps certain cancers transform into more aggressive forms. They found this circular RNA is very common in two deadly cancer types: neuroendocrine prostate cancer and small cell lung cancer. When researchers removed circular RMST from cancer cells in lab dishes and mice, the tumors could not change into these dangerous forms and stayed as less aggressive cancers instead. The circular RMST works by partnering with specific proteins that control which genes get turned on or off in cells. Without circular RMST, these partner proteins break down or stop working properly, preventing the cancer transformation. This discovery is important because it reveals a new target for treating these hard-to-cure cancers. By blocking circular RMST, doctors might be able to stop cancers from becoming more aggressive and deadly, giving patients better treatment options. | Cancer cells can sometimes change their identity, switching from one type to another in a process that helps them survive treatments. Scientists studied a special molecule called circular RMST that plays a key role when prostate and lung cancers transform into more aggressive forms called neuroendocrine cancers. These transformed cancers are much harder to treat and have worse outcomes for patients. The researchers found that circular RMST acts like a molecular coordinator, working with other important proteins to control this dangerous transformation. When they removed circular RMST from cancer cells in laboratory experiments and mouse models, the cancers could not complete their transformation and remained in their original, less aggressive state. The molecule works by protecting key proteins from being destroyed and helping them function properly to drive the cancer transformation. Understanding how circular RMST controls this process could lead to new treatments that prevent cancers from becoming more aggressive and harder to cure. |
| CancCell | 2 | 10.1016/j.ccell.2025.03.030 | Gemini | Circular RNA (circRNA) is a class of noncoding RNA with regulatory potentials. Its role in the transdifferentiation of prostate and lung adenocarcinoma into neuroendocrine prostate cancer (NEPC) and small cell lung cancer (SCLC) remains unexplored. Here, we identified circRMST as an exceptionally abundant circRNA predominantly expressed in NEPC and SCLC, with strong conservation between humans and mice. Functional studies using shRNA, siRNA, CRISPR-Cas13, and Cas9 consistently demonstrate that circRMST is essential for tumor growth and the expression of ASCL1, a master regulator of neuroendocrine fate. Genetic knockout of Rmst in NEPC genetic engineered mouse models prevents neuroendocrine transdifferentiation, maintaining tumors in an adenocarcinoma state. Mechanistically, circRMST physically interacts with lineage transcription factors NKX2-1 and SOX2. Loss of circRMST induces NKX2-1 protein degradation through autophagy-lysosomal pathway and alters the genomic binding of SOX2, collectively leading to the loss of ASCL1 transcription. | This research investigates a new type of genetic instruction, called a circular RNA, to understand how certain cancers change into a more aggressive form. We found a specific circular RNA, called circRMST, is very common in advanced prostate and lung cancers that have transformed into a neuroendocrine type. Our studies showed that removing circRMST stopped cancer growth and prevented this transformation. This happens because circRMST works together with other important proteins, called transcription factors, to control the main gene responsible for this cancer transformation. Ultimately, our findings reveal a crucial partnership that drives this aggressive cancer type, which could lead to new ways to diagnose and treat these diseases. | This research investigates how a specific type of RNA, called circular RMST, may help certain cancers change their identity. We found that circular RMST is very common in aggressive neuroendocrine prostate and small cell lung cancers. Through various experiments, we discovered that this circular RNA is essential for the growth of these cancer cells and for activating a key gene, ASCL1, which drives the cancer’s transformation. Our findings show that circular RMST works with other important proteins to activate ASCL1. By better understanding this process, future studies could find new ways to prevent or treat these aggressive cancers. This provides a new target for developing innovative therapies. | This research investigates how a specific type of RNA, called circular RMST, helps prostate and lung cancers change into a more aggressive form. This change is a process known as neuroendocrine transdifferentiation. We discovered that circular RMST is highly abundant in these aggressive cancers and is crucial for their growth. By removing circular RMST, we found that the cancer cells could no longer transform, remaining in a less dangerous state. We also found that this circular RNA works with other important proteins, called transcription factors, to control a key gene called ASCL1. The findings from this study help us understand how these cancers change, which could lead to new ways to stop this process and potentially develop new treatments. |
| CancCell | 2 | 10.1016/j.ccell.2025.03.031 | DeepSeek | Circular RNA (circRNA) is a class of noncoding RNA with regulatory potentials. Its role in the transdifferentiation of prostate and lung adenocarcinoma into neuroendocrine prostate cancer (NEPC) and small cell lung cancer (SCLC) remains unexplored. Here, we identified circRMST as an exceptionally abundant circRNA predominantly expressed in NEPC and SCLC, with strong conservation between humans and mice. Functional studies using shRNA, siRNA, CRISPR-Cas13, and Cas9 consistently demonstrate that circRMST is essential for tumor growth and the expression of ASCL1, a master regulator of neuroendocrine fate. Genetic knockout of Rmst in NEPC genetic engineered mouse models prevents neuroendocrine transdifferentiation, maintaining tumors in an adenocarcinoma state. Mechanistically, circRMST physically interacts with lineage transcription factors NKX2-1 and SOX2. Loss of circRMST induces NKX2-1 protein degradation through autophagy-lysosomal pathway and alters the genomic binding of SOX2, collectively leading to the loss of ASCL1 transcription. | Some cancers, like prostate and lung tumors, can change into a more aggressive type called neuroendocrine cancer, which is harder to treat. Scientists discovered a special circular RNA named circRMST that is very common in these aggressive cancers. They found that circRMST helps the cancer switch to this dangerous form by working with key proteins that control cell identity. When researchers blocked circRMST in mice, the tumors stayed in a less aggressive state. circRMST does this by sticking to important proteins like NKX2-1 and SOX2, which normally help the cancer transform. Without circRMST, these proteins break down or don’t work right, stopping the cancer from becoming neuroendocrine. This discovery could lead to new treatments that prevent cancers from turning into more deadly forms by targeting circRMST and its partners. | Some aggressive cancers, like certain prostate and lung cancers, can transform into a more dangerous type called neuroendocrine cancer. Scientists discovered a special circular RNA molecule, called circRMST, that plays a key role in this transformation. When they blocked circRMST in lab tests, the cancer cells stopped changing into neuroendocrine type and grew more slowly. They found that circRMST works by interacting with important proteins that control cell identity, keeping them stable and active. Without circRMST, these proteins break down or don’t work correctly, preventing the cancer from becoming more aggressive. This discovery helps explain how cancers evolve into harder-to-treat forms and could lead to new ways to stop this dangerous change in patients. | This study explores how a special type of RNA, called circRMST, helps certain cancers—like prostate and lung tumors—transform into aggressive neuroendocrine cancers (a harder-to-treat type). Researchers found that circRMST is very common in these cancers and works with key proteins (NKX2-1 and SOX2) to control cancer cell behavior. When they blocked circRMST in lab tests and mice, tumors stopped changing into neuroendocrine forms and grew more slowly. This discovery could lead to new treatments that prevent cancer progression by targeting circRMST or its partner proteins. Understanding this process may help doctors better tackle these tough cancers in the future. |
| CancCell | 2 | 10.1016/j.ccell.2025.03.032 | Grok | Circular RNA (circRNA) is a class of noncoding RNA with regulatory potentials. Its role in the transdifferentiation of prostate and lung adenocarcinoma into neuroendocrine prostate cancer (NEPC) and small cell lung cancer (SCLC) remains unexplored. Here, we identified circRMST as an exceptionally abundant circRNA predominantly expressed in NEPC and SCLC, with strong conservation between humans and mice. Functional studies using shRNA, siRNA, CRISPR-Cas13, and Cas9 consistently demonstrate that circRMST is essential for tumor growth and the expression of ASCL1, a master regulator of neuroendocrine fate. Genetic knockout of Rmst in NEPC genetic engineered mouse models prevents neuroendocrine transdifferentiation, maintaining tumors in an adenocarcinoma state. Mechanistically, circRMST physically interacts with lineage transcription factors NKX2-1 and SOX2. Loss of circRMST induces NKX2-1 protein degradation through autophagy-lysosomal pathway and alters the genomic binding of SOX2, collectively leading to the loss of ASCL1 transcription. | Prostate and lung cancers can change into more aggressive forms called neuroendocrine prostate cancer and small cell lung cancer. This study looks at why this happens and how a special loop-shaped RNA, called circular RMST, plays a key role. Researchers wanted to learn if this RNA helps drive the change and if stopping it could prevent it. They used tools to remove or block the RNA in lab tests and mice, and found that without it, the cancers stayed in their less harmful state. The RNA works by teaming up with proteins that control cell changes, keeping a master switch for the aggressive form turned on. These findings could help scientists develop new treatments to block this shift, giving hope for better ways to fight these tough cancers and improve patient lives. | Some cancers, like those in the prostate and lungs, can change into more aggressive types called neuroendocrine cancers. This study looks at why that happens and how a special molecule called circular RMST plays a key role. Researchers wanted to see if blocking this molecule could stop the change and slow cancer growth. They used tools to turn off circular RMST in lab tests and mice, and found it works with important proteins to drive the cancer shift. Without it, tumors stayed less harmful and grew slower. These findings could help scientists develop new ways to treat tough cancers, making therapies better for patients everywhere. | Prostate and lung cancers can change into more aggressive forms called neuroendocrine cancers, which are hard to treat. This study explores why this happens by focusing on a special loop-shaped molecule called circular RMST, which helps control genes without making proteins. Researchers wanted to see if circular RMST drives this harmful change and how it works. Using lab tools to block or remove it in cells and mice, they found that circular RMST teams up with key gene controllers to keep the cancer aggressive and growing. Without it, the cancers stay in their less harmful form and stop spreading. These findings could help scientists develop new ways to block this change, leading to better treatments that save lives by keeping cancers easier to manage. |
| CancCell | 3 | 10.1016/j.ccell.2025.02.019 | Open AI | PDGFRA is crucial to tumorigenesis and frequently genomically altered in high-grade glioma (HGG). In a comprehensive dataset of pediatric HGG (n = 261), we detect PDGFRA mutations and/or amplifications in 15% of cases, suggesting PDGFRA as a therapeutic target. We reveal that the PDGFRA/KIT inhibitor avapritinib shows (1) selectivity for PDGFRA inhibition, (2) distinct patterns of subcellular effects, (3) in vitro and in vivo activity in patient-derived HGG models, and (4) effective blood-brain barrier penetration in mice and humans. Furthermore, we report preliminary clinical real-world experience using avapritinib in pediatric and young adult patients with predominantly recurrent/refractory PDGFRA-altered HGG (n = 8). Our early data demonstrate that avapritinib is well tolerated and results in radiographic response in 3/7 cases, suggesting a potential role for avapritinib in the treatment of HGG with specific PDGFRA alterations. Overall, these translational results underscore the therapeutic potential of PDGFRA inhibition with avapritinib in HGG. | High-grade glioma is an aggressive brain cancer that is hard to treat. In some patients, the cancer cells have changes in a gene called PDGFRA, which helps the tumor grow. This study looked at avapritinib, a drug designed to block PDGFRA. We found that PDGFRA changes were present in about 15% of pediatric high-grade gliomas. Laboratory tests and animal studies showed that avapritinib can reach the brain, block PDGFRA activity, and slow tumor growth. We also reviewed early real-world use of avapritinib in eight young patients whose tumors had PDGFRA changes. The treatment was well tolerated, and three patients showed tumor shrinkage on scans. These results suggest that avapritinib may be a promising treatment option for certain high-grade gliomas, and they provide important information for future research and clinical trials. | High-grade gliomas are aggressive brain tumors that are very hard to treat. In some patients, these tumors have changes in a gene called PDGFRA, which may help the cancer grow. This study looked at a new treatment called avapritinib, which is designed to block PDGFRA activity. In lab tests and animal studies, avapritinib was able to reach the brain, target tumor cells, and slow their growth. We also studied a small group of children and young adults with this type of tumor. The medicine was generally well tolerated, and some patients showed signs of tumor shrinkage. These results suggest that avapritinib could be a promising option for patients whose tumors have PDGFRA changes. More research is needed to confirm these findings and to see if this treatment can help more people live longer and better. | High-grade gliomas are aggressive brain tumors that are very difficult to treat, especially in children and young adults. In some of these tumors, a gene called PDGFRA is changed in a way that helps the cancer grow. This study looked at whether a drug called avapritinib, which blocks PDGFRA, could be useful for these patients. Researchers tested the drug in the lab using tumor samples and animal models, and found it could reach the brain, slow tumor growth, and act specifically on PDGFRA changes. They also studied a small group of patients whose tumors had these changes and had returned after other treatments. Avapritinib was generally well tolerated and showed tumor shrinkage in some patients. These early results suggest that avapritinib could become a promising treatment option for people with high-grade gliomas that have PDGFRA changes, offering new hope for a disease with limited therapies. |
| CancCell | 3 | 10.1016/j.ccell.2025.02.020 | Claude | PDGFRA is crucial to tumorigenesis and frequently genomically altered in high-grade glioma (HGG). In a comprehensive dataset of pediatric HGG (n = 261), we detect PDGFRA mutations and/or amplifications in 15% of cases, suggesting PDGFRA as a therapeutic target. We reveal that the PDGFRA/KIT inhibitor avapritinib shows (1) selectivity for PDGFRA inhibition, (2) distinct patterns of subcellular effects, (3) in vitro and in vivo activity in patient-derived HGG models, and (4) effective blood-brain barrier penetration in mice and humans. Furthermore, we report preliminary clinical real-world experience using avapritinib in pediatric and young adult patients with predominantly recurrent/refractory PDGFRA-altered HGG (n = 8). Our early data demonstrate that avapritinib is well tolerated and results in radiographic response in 3/7 cases, suggesting a potential role for avapritinib in the treatment of HGG with specific PDGFRA alterations. Overall, these translational results underscore the therapeutic potential of PDGFRA inhibition with avapritinib in HGG. | Brain tumors called high-grade gliomas are among the most dangerous cancers, especially in children and young adults. These tumors are very hard to treat because most drugs cannot cross the blood-brain barrier to reach the tumor. Scientists found that a protein called PDGFRA helps these tumors grow and is changed in about 15% of patients with these brain cancers. This study tested a new drug called avapritinib that blocks PDGFRA and can cross into the brain. Researchers first tested the drug in lab models of these tumors and found it worked well to stop tumor growth. They also confirmed the drug reaches the brain in both mice and humans. Then they gave the drug to eight young patients whose tumors had returned after other treatments failed. The drug was safe and shrank tumors in three out of seven patients who could be evaluated. These results show that avapritinib could be a promising new treatment option for patients with these specific types of brain tumors. | Brain tumors called high-grade gliomas are among the most dangerous cancers, especially in children and young adults. These tumors often have changes in a protein called PDGFRA that helps them grow and spread. Scientists wanted to test whether a drug called avapritinib could stop these tumors by blocking PDGFRA. They studied 261 pediatric brain tumor samples and found that 15% had PDGFRA changes, making it a promising target for treatment. The researchers tested avapritinib in laboratory models and found it effectively reached the brain and slowed tumor growth. They also gave the drug to eight young patients with these specific brain tumors. The treatment was safe and caused tumors to shrink in three out of seven patients who could be evaluated. These encouraging results suggest that avapritinib could become a new treatment option for patients with high-grade gliomas that have PDGFRA changes, potentially improving outcomes for these challenging cancers. | Brain tumors called high-grade gliomas are very hard to treat, especially in children. Scientists found that a protein called PDGFRA helps these tumors grow and is changed in about 15% of these brain cancers. They tested a drug called avapritinib that blocks this protein to see if it could help patients. The researchers first studied the drug in lab dishes and mice with these tumors. They found that avapritinib works well against the tumors and can cross into the brain, which is important since many drugs cannot reach brain tumors. They then gave the drug to eight young patients whose tumors had come back after other treatments. The drug was safe and helped shrink tumors in three out of seven patients they could measure. This research shows that avapritinib might be a new treatment option for children and young adults with these specific types of brain tumors that have PDGFRA changes. |
| CancCell | 3 | 10.1016/j.ccell.2025.02.021 | Gemini | PDGFRA is crucial to tumorigenesis and frequently genomically altered in high-grade glioma (HGG). In a comprehensive dataset of pediatric HGG (n = 261), we detect PDGFRA mutations and/or amplifications in 15% of cases, suggesting PDGFRA as a therapeutic target. We reveal that the PDGFRA/KIT inhibitor avapritinib shows (1) selectivity for PDGFRA inhibition, (2) distinct patterns of subcellular effects, (3) in vitro and in vivo activity in patient-derived HGG models, and (4) effective blood-brain barrier penetration in mice and humans. Furthermore, we report preliminary clinical real-world experience using avapritinib in pediatric and young adult patients with predominantly recurrent/refractory PDGFRA-altered HGG (n = 8). Our early data demonstrate that avapritinib is well tolerated and results in radiographic response in 3/7 cases, suggesting a potential role for avapritinib in the treatment of HGG with specific PDGFRA alterations. Overall, these translational results underscore the therapeutic potential of PDGFRA inhibition with avapritinib in HGG. | This research explores new treatment options for a specific type of brain cancer called high-grade glioma. Many of these tumors have a change in a gene called PDGFRA, which helps the cancer grow. We studied a new drug, avapritinib, which is designed to block this gene's signal. Our laboratory and animal studies showed that this drug successfully targets the cancer cells and can even pass through the protective brain barrier. We also tested avapritinib in a small number of young patients, where it was well-tolerated and showed promising signs of shrinking tumors. These early findings suggest that avapritinib could be an effective new therapy for patients whose brain tumors have this specific genetic change. | This study explores a new way to treat a type of brain tumor called high-grade glioma, which is especially common in children. Researchers found that many of these tumors have a specific change, or "alteration," in a protein called PDGFRA. The purpose of this research was to see if a drug called avapritinib, which blocks this protein, could be an effective treatment. Using patient-derived tumor cells and animal models, the scientists showed that the drug can successfully enter the brain and slow down tumor growth. Additionally, they observed promising early results in a small group of patients, where the drug was well-tolerated and helped shrink tumors in some cases. These findings suggest that avapritinib could be a valuable new therapy for patients with these specific tumor alterations. | This research explores new treatment options for a type of aggressive brain tumor called high-grade glioma, which often has a specific genetic flaw related to a protein called PDGFRA. The study used a drug named avapritinib, which is designed to block the activity of this protein. Researchers first showed that this drug effectively targets the protein, can cross into the brain, and slowed tumor growth in lab models. The team also treated a small group of young patients with the tumor, finding that the drug was well-tolerated and showed promise in shrinking tumors in some cases. These findings suggest that avapritinib could be a valuable new therapy for patients with this specific genetic change in their brain tumors. |
| CancCell | 3 | 10.1016/j.ccell.2025.02.022 | DeepSeek | PDGFRA is crucial to tumorigenesis and frequently genomically altered in high-grade glioma (HGG). In a comprehensive dataset of pediatric HGG (n = 261), we detect PDGFRA mutations and/or amplifications in 15% of cases, suggesting PDGFRA as a therapeutic target. We reveal that the PDGFRA/KIT inhibitor avapritinib shows (1) selectivity for PDGFRA inhibition, (2) distinct patterns of subcellular effects, (3) in vitro and in vivo activity in patient-derived HGG models, and (4) effective blood-brain barrier penetration in mice and humans. Furthermore, we report preliminary clinical real-world experience using avapritinib in pediatric and young adult patients with predominantly recurrent/refractory PDGFRA-altered HGG (n = 8). Our early data demonstrate that avapritinib is well tolerated and results in radiographic response in 3/7 cases, suggesting a potential role for avapritinib in the treatment of HGG with specific PDGFRA alterations. Overall, these translational results underscore the therapeutic potential of PDGFRA inhibition with avapritinib in HGG. | High-grade gliomas (HGG) are aggressive brain tumors that sometimes have changes in a gene called PDGFRA, which helps them grow. This study looked at a drug called avapritinib, which blocks PDGFRA, to see if it could treat these tumors. Tests in lab-grown cells and mice showed that the drug works against HGG with PDGFRA changes and can reach the brain effectively. Early results from a small group of young patients with hard-to-treat HGG also showed that avapritinib was safe and helped shrink tumors in some cases. While more research is needed, these findings suggest that avapritinib could be a promising treatment for certain HGG patients with PDGFRA alterations. | This study looks at a type of aggressive brain tumor called high-grade glioma (HGG), which often has changes in a gene called PDGFRA. Researchers tested a drug called avapritinib, which targets these changes, to see if it could help treat the cancer. They found that avapritinib works against HGG in lab tests and animal studies, and it can also reach the brain effectively. Early results from a small group of young patients with hard-to-treat HGG showed that the drug was safe and helped shrink tumors in some cases. These findings suggest that avapritinib could be a promising treatment for HGG patients with PDGFRA changes, offering new hope where options are limited. | This study explores a new way to treat aggressive brain tumors called high-grade gliomas, especially those with changes in a gene called PDGFRA. About 15% of these tumors in children have PDGFRA problems, making it a good target for therapy. Researchers tested a drug called avapritinib, which blocks PDGFRA, and found it works well in lab tests and animal studies. It also reaches the brain effectively in mice and humans. Early results from a small group of young patients with hard-to-treat tumors show the drug is safe and helped shrink tumors in some cases. These findings suggest avapritinib could be a promising treatment for certain brain tumors with PDGFRA changes, offering hope for better options in the future. |
| CancCell | 3 | 10.1016/j.ccell.2025.02.023 | Grok | PDGFRA is crucial to tumorigenesis and frequently genomically altered in high-grade glioma (HGG). In a comprehensive dataset of pediatric HGG (n = 261), we detect PDGFRA mutations and/or amplifications in 15% of cases, suggesting PDGFRA as a therapeutic target. We reveal that the PDGFRA/KIT inhibitor avapritinib shows (1) selectivity for PDGFRA inhibition, (2) distinct patterns of subcellular effects, (3) in vitro and in vivo activity in patient-derived HGG models, and (4) effective blood-brain barrier penetration in mice and humans. Furthermore, we report preliminary clinical real-world experience using avapritinib in pediatric and young adult patients with predominantly recurrent/refractory PDGFRA-altered HGG (n = 8). Our early data demonstrate that avapritinib is well tolerated and results in radiographic response in 3/7 cases, suggesting a potential role for avapritinib in the treatment of HGG with specific PDGFRA alterations. Overall, these translational results underscore the therapeutic potential of PDGFRA inhibition with avapritinib in HGG. | High-grade brain tumors are serious cancers that often affect children and young adults. Changes in a gene called PDGFRA help these tumors grow and happen in about 15% of cases, so researchers wanted to find a better way to target this gene. They tested a drug named avapritinib to see if it could stop PDGFRA safely and effectively. In lab tests, animal studies, and early trials with eight patients, the drug showed it could reach the brain, was easy to tolerate, and shrank tumors in some people. These findings suggest avapritinib might offer new hope for treating these hard-to-cure brain cancers, encouraging more studies to help patients live longer and better. | This study explores a new way to treat high-grade glioma, a serious brain cancer, by targeting a gene called PDGFRA, which is often changed in these tumors. Researchers found that about 15% of children with this cancer have these gene changes, making it a good target for treatment. They tested a drug called avapritinib, which blocks PDGFRA and can reach the brain effectively. In lab tests and early patient trials, avapritinib was safe and shrank tumors in some cases. This research is important because it could lead to better treatments for people with this type of brain cancer, giving hope for improved outcomes. The findings may guide scientists to develop more targeted therapies, helping the research community find new ways to fight this disease. | High-grade gliomas are aggressive brain tumors that are hard to treat, especially in children. A protein called PDGFRA often changes in these tumors and helps them grow, so researchers studied if blocking it could help. They looked at tumor samples from 261 kids and found changes in this protein in 15 percent of cases. They tested a drug called avapritinib, which targets this protein, in lab models from patients, in animals, and in eight young people with tough-to-treat tumors. The drug worked well in tests, crossed into the brain easily, and was safe. It shrank tumors in three out of seven patients. This suggests avapritinib could become a new way to fight these brain tumors, giving hope for better treatments and inspiring more studies to improve care for affected families. |
| CancCell | 4 | 10.1016/j.ccell.2024.11.006 | Open AI | Anti-tumor immunity is crucial for high-grade serous ovarian cancer (HGSC) prognosis, yet its adaptation upon standard chemotherapy remains poorly understood. Here, we conduct spatial and molecular characterization of 117 HGSC samples collected before and after chemotherapy. Our single-cell and spatial analyses reveal increasingly versatile immune cell states forming spatiotemporally dynamic microcommunities. We describe Myelonets, networks of interconnected myeloid cells that contribute to CD8+ T cell exhaustion post-chemotherapy and show that M1/M2 polarization at the tumor-stroma interface is associated with CD8+ T cell exhaustion and exclusion, correlating with poor chemoresponse. Single-cell and spatial transcriptomics reveal prominent myeloid-T cell interactions via NECTIN2-TIGIT induced by chemotherapy. Targeting these interactions using a functional patient-derived immuno-oncology platform demonstrates that high NECTIN2-TIGIT signaling in matched tumors predicts responses to immune checkpoint blockade. Our discovery of clinically relevant myeloid-driven spatial T cell exhaustion unlocks immunotherapeutic strategies to unleash CD8+ T cell-mediated anti-tumor immunity in HGSC. | In ovarian cancer, the body’s immune system can help fight tumors, but this defense often weakens during treatment. We studied tumor samples from women with high-grade serous ovarian cancer before and after chemotherapy to see how immune cells changed. Using advanced methods, we found that chemotherapy can create clusters of myeloid immune cells that interact with and exhaust cancer-fighting T cells, making them less able to attack the tumor. We discovered specific signals, such as NECTIN2-TIGIT, that drive this exhaustion and may explain why some patients respond poorly to treatment. By testing drugs on patient-derived tumor models, we found that blocking these signals could restore T cell activity. These results highlight a potential new approach to boost the immune system during or after chemotherapy, which could improve treatment outcomes for women with ovarian cancer. | This study explores how chemotherapy changes the immune system’s response in high-grade serous ovarian cancer. Researchers examined tumor samples from patients before and after chemotherapy using advanced single-cell and spatial analysis. They found that chemotherapy can cause certain immune cells, called myeloid cells, to form tight networks that lead to the “exhaustion” of cancer-fighting T cells. This exhaustion happens in specific areas of the tumor and is linked to poorer treatment results. The team also discovered a key communication signal between myeloid cells and T cells, called NECTIN2-TIGIT, which becomes stronger after chemotherapy. Blocking this signal in lab models helped restore T cell activity. These findings may guide new treatments that combine chemotherapy with drugs to reactivate T cells, offering hope for improving outcomes in ovarian cancer patients. | This study looked at how the body’s immune system changes in women with high-grade serous ovarian cancer before and after chemotherapy. Researchers examined tumor samples at the single-cell level to see how immune cells interact and organize. They found that certain myeloid cells form close networks after treatment, which can weaken the cancer-fighting T cells in specific tumor areas. This effect was linked to poorer responses to chemotherapy. They also discovered a key signaling pathway between myeloid cells and T cells that may be blocked to restore immune function. These findings suggest new ways to combine chemotherapy with treatments that boost the immune system, which could improve outcomes for patients with this type of ovarian cancer. |
| CancCell | 4 | 10.1016/j.ccell.2024.11.007 | Claude | Anti-tumor immunity is crucial for high-grade serous ovarian cancer (HGSC) prognosis, yet its adaptation upon standard chemotherapy remains poorly understood. Here, we conduct spatial and molecular characterization of 117 HGSC samples collected before and after chemotherapy. Our single-cell and spatial analyses reveal increasingly versatile immune cell states forming spatiotemporally dynamic microcommunities. We describe Myelonets, networks of interconnected myeloid cells that contribute to CD8+ T cell exhaustion post-chemotherapy and show that M1/M2 polarization at the tumor-stroma interface is associated with CD8+ T cell exhaustion and exclusion, correlating with poor chemoresponse. Single-cell and spatial transcriptomics reveal prominent myeloid-T cell interactions via NECTIN2-TIGIT induced by chemotherapy. Targeting these interactions using a functional patient-derived immuno-oncology platform demonstrates that high NECTIN2-TIGIT signaling in matched tumors predicts responses to immune checkpoint blockade. Our discovery of clinically relevant myeloid-driven spatial T cell exhaustion unlocks immunotherapeutic strategies to unleash CD8+ T cell-mediated anti-tumor immunity in HGSC. | Ovarian cancer is hard to treat, and doctors need to understand how the body's immune system fights this disease. When patients get chemotherapy, their immune cells change in ways that doctors don't fully understand. This study looked at tissue samples from 117 ovarian cancer patients before and after chemotherapy treatment. The researchers used special tools to see how different immune cells work together in the tumor. They found that chemotherapy causes harmful immune cells called myeloid cells to form networks that make the helpful cancer-fighting T cells too tired to work properly. These tired T cells cannot kill cancer cells effectively. The study also discovered that certain proteins help these harmful interactions happen. When doctors blocked these proteins in lab tests, the cancer-fighting T cells worked better again. This research helps explain why some cancer treatments don't work well and points to new ways to help the immune system fight ovarian cancer more effectively. | Cancer treatments like chemotherapy can weaken the body's natural defense system against tumors. Scientists studied ovarian cancer samples from 117 patients before and after chemotherapy to understand how the immune system changes during treatment. They found that chemotherapy causes harmful immune cells called myeloid cells to form networks that tire out the good cancer-fighting cells called CD8+ T cells. These tired T cells become less able to attack cancer cells. The researchers discovered that two proteins, NECTIN2 and TIGIT, work together to cause this problem. When these proteins are highly active, patients respond better to a different type of cancer treatment called immune checkpoint blockade therapy. This research helps doctors understand why some cancer treatments stop working and points to new ways to boost the immune system's ability to fight ovarian cancer. The findings could lead to better treatment combinations that keep the immune system strong while fighting cancer. | Cancer treatments like chemotherapy are designed to kill cancer cells, but they can also affect the body's immune system in unexpected ways. This study looked at how chemotherapy changes the immune response in ovarian cancer, which is important because a strong immune system helps fight cancer. The researchers studied tissue samples from 117 ovarian cancer patients before and after chemotherapy to see what happened to their immune cells. They found that chemotherapy actually made it harder for the body's cancer-fighting cells (called T cells) to do their job. This happened because chemotherapy caused other immune cells (called myeloid cells) to form networks that tired out the T cells and stopped them from attacking the cancer. The researchers also discovered a specific communication pathway between these cells that could be targeted with new treatments. By understanding how chemotherapy weakens the immune response, doctors may be able to combine chemotherapy with immune-boosting treatments to help patients fight their cancer more effectively. |
| CancCell | 4 | 10.1016/j.ccell.2024.11.008 | Gemini | Anti-tumor immunity is crucial for high-grade serous ovarian cancer (HGSC) prognosis, yet its adaptation upon standard chemotherapy remains poorly understood. Here, we conduct spatial and molecular characterization of 117 HGSC samples collected before and after chemotherapy. Our single-cell and spatial analyses reveal increasingly versatile immune cell states forming spatiotemporally dynamic microcommunities. We describe Myelonets, networks of interconnected myeloid cells that contribute to CD8+ T cell exhaustion post-chemotherapy and show that M1/M2 polarization at the tumor-stroma interface is associated with CD8+ T cell exhaustion and exclusion, correlating with poor chemoresponse. Single-cell and spatial transcriptomics reveal prominent myeloid-T cell interactions via NECTIN2-TIGIT induced by chemotherapy. Targeting these interactions using a functional patient-derived immuno-oncology platform demonstrates that high NECTIN2-TIGIT signaling in matched tumors predicts responses to immune checkpoint blockade. Our discovery of clinically relevant myeloid-driven spatial T cell exhaustion unlocks immunotherapeutic strategies to unleash CD8+ T cell-mediated anti-tumor immunity in HGSC. | Chemotherapy is a common treatment for ovarian cancer, but we don't fully understand how it affects the body's immune response within the tumor. This study looked at tumor samples from patients before and after they received chemotherapy. We found that after treatment, certain immune cells called myeloid cells create a network that can make other key immune cells, known as T cells, tired or "exhausted." This exhaustion prevents the T cells from fighting the cancer effectively. Our research shows that targeting the communication between these myeloid and T cells could improve the effectiveness of other cancer treatments, potentially helping the body's immune system fight the cancer more successfully. This discovery offers a new way to develop better therapies for patients with ovarian cancer. | Chemotherapy is a common treatment for ovarian cancer, but we don't fully understand how it affects the body's own immune response to the tumor. This study looks closely at tumor samples from patients before and after chemotherapy to see how the immune cells change. We found that after treatment, certain immune cells called myeloid cells form new connections and suppress other immune cells, specifically CD8+ T cells, which are important for fighting cancer. This suppression happens in specific areas of the tumor and makes the T cells "exhausted" or less effective. Our research shows that blocking the signals between these myeloid and T cells could help wake up the immune system and make new treatments work better. This discovery may lead to new ways to use the immune system to fight ovarian cancer, especially for patients who don't respond well to current treatments. | This research explores how standard chemotherapy affects the immune system in ovarian cancer, specifically focusing on a type called high-grade serous ovarian cancer. We studied patient tumor samples both before and after chemotherapy to understand the changes that occur. Our findings show that chemotherapy causes certain immune cells, called myeloid cells, to form networks that make T cells—which are crucial for fighting cancer—become exhausted and less effective. We found a specific interaction between these myeloid cells and T cells that seems to be a key reason for this exhaustion. By understanding this process, we believe we can develop new treatment strategies. We found that targeting these interactions could potentially make existing immunotherapies more effective for patients, helping to unleash the body's own ability to fight the cancer more successfully. |
| CancCell | 4 | 10.1016/j.ccell.2024.11.009 | DeepSeek | Anti-tumor immunity is crucial for high-grade serous ovarian cancer (HGSC) prognosis, yet its adaptation upon standard chemotherapy remains poorly understood. Here, we conduct spatial and molecular characterization of 117 HGSC samples collected before and after chemotherapy. Our single-cell and spatial analyses reveal increasingly versatile immune cell states forming spatiotemporally dynamic microcommunities. We describe Myelonets, networks of interconnected myeloid cells that contribute to CD8+ T cell exhaustion post-chemotherapy and show that M1/M2 polarization at the tumor-stroma interface is associated with CD8+ T cell exhaustion and exclusion, correlating with poor chemoresponse. Single-cell and spatial transcriptomics reveal prominent myeloid-T cell interactions via NECTIN2-TIGIT induced by chemotherapy. Targeting these interactions using a functional patient-derived immuno-oncology platform demonstrates that high NECTIN2-TIGIT signaling in matched tumors predicts responses to immune checkpoint blockade. Our discovery of clinically relevant myeloid-driven spatial T cell exhaustion unlocks immunotherapeutic strategies to unleash CD8+ T cell-mediated anti-tumor immunity in HGSC. | Chemotherapy for ovarian cancer can weaken the body’s immune fight against tumors by causing certain immune cells (called myeloid cells) to form networks that exhaust helpful T cells. In this study, researchers examined 117 ovarian cancer samples before and after chemotherapy using advanced techniques to map how immune cells behave. They found that after treatment, myeloid cells interact with T cells in ways that shut them down, making the cancer harder to control. The team also discovered that blocking these interactions could help T cells work better, potentially improving immunotherapy responses. These findings open new ways to boost the immune system’s ability to fight ovarian cancer after chemotherapy. | Chemotherapy for ovarian cancer can weaken the body’s immune defense by causing certain immune cells (called myeloid cells) to form networks that block helpful T cells from fighting the tumor. In this study, researchers analyzed 117 ovarian cancer samples before and after chemotherapy and found that these myeloid networks push T cells into a "tired" state, making treatment less effective. They also discovered that a specific interaction (NECTIN2-TIGIT) between myeloid cells and T cells plays a key role in this process. By blocking this interaction in lab tests, they improved T cell function, suggesting a way to make immunotherapy work better for ovarian cancer patients. These findings could lead to new treatments that help the immune system fight cancer more effectively after chemotherapy. | This study explores why ovarian cancer often resists chemotherapy by looking at how the treatment affects the immune system. Researchers analyzed 117 tumor samples before and after chemo, using advanced methods to map immune cell behavior. They discovered that chemotherapy triggers harmful networks of immune cells (called Myelonets) that weaken cancer-fighting T cells, making treatment less effective. These myeloid cells block T cells by using signals like *NECTIN2-TIGIT*, which act like an "off switch" for immunity. But when scientists blocked these signals in lab tests, T cells regained strength—suggesting future therapies could combine chemo with immunotherapies to boost results. The findings help explain why some patients don’t respond to chemo and offer hope for better treatments by targeting these immune roadblocks. |
| CancCell | 4 | 10.1016/j.ccell.2024.11.010 | Grok | Anti-tumor immunity is crucial for high-grade serous ovarian cancer (HGSC) prognosis, yet its adaptation upon standard chemotherapy remains poorly understood. Here, we conduct spatial and molecular characterization of 117 HGSC samples collected before and after chemotherapy. Our single-cell and spatial analyses reveal increasingly versatile immune cell states forming spatiotemporally dynamic microcommunities. We describe Myelonets, networks of interconnected myeloid cells that contribute to CD8+ T cell exhaustion post-chemotherapy and show that M1/M2 polarization at the tumor-stroma interface is associated with CD8+ T cell exhaustion and exclusion, correlating with poor chemoresponse. Single-cell and spatial transcriptomics reveal prominent myeloid-T cell interactions via NECTIN2-TIGIT induced by chemotherapy. Targeting these interactions using a functional patient-derived immuno-oncology platform demonstrates that high NECTIN2-TIGIT signaling in matched tumors predicts responses to immune checkpoint blockade. Our discovery of clinically relevant myeloid-driven spatial T cell exhaustion unlocks immunotherapeutic strategies to unleash CD8+ T cell-mediated anti-tumor immunity in HGSC. | Ovarian cancer often relies on the body's immune system to fight tumors, but standard chemotherapy's effects on this defense are not well known. Researchers studied 117 tumor samples from before and after chemo using detailed cell and space mapping to see how immune cells change. They found that chemo creates networks of support cells that tire out key fighter T cells in specific spots, leading to weaker tumor attacks and poorer treatment results. These networks involve signals between cells that chemo boosts, but blocking them could revive T cells. This work helps scientists develop better immune-boosting therapies, predict who benefits from them, and improve outcomes for ovarian cancer patients by targeting these hidden weak spots in the body's fight against cancer. | Ovarian cancer is tough to treat, and while the body's immune system can fight it, we don't fully know how standard chemotherapy changes this defense. Researchers wanted to learn how immune cells adapt after chemo to find better ways to help patients. They studied 117 tumor samples from before and after treatment using tools that map cells and their interactions in detail. They found that chemo causes groups of immune cells called myeloid cells to form networks that tire out key fighter T cells in specific spots, leading to weaker attacks on cancer and poor responses in some cases. These tired T cells happen through signals that chemo boosts, but blocking them could predict and improve success with immune-boosting drugs. This work opens doors for new treatments that revive T cells, potentially saving more lives by combining chemo with targeted immune help. | High-grade serous ovarian cancer relies on the body's immune system to fight tumors, but we do not fully know how standard chemotherapy changes this defense. This study looks at tumor samples from before and after treatment to understand these shifts. Researchers used advanced tools to map immune cells and found that chemotherapy creates networks of myeloid cells that wear out key fighter T cells in specific areas, leading to weaker responses in some patients. They also discovered a signal between cells that blocks T cell action, which could predict how well new immune-boosting drugs work. These insights help scientists develop better ways to strengthen the immune attack against ovarian cancer, improving treatments for patients who do not respond well to current options. |
| CancCell | 5 | 10.1016/j.ccell.2025.03.007 | Open AI | Immune checkpoint inhibitors (ICIs) have improved outcomes of patients with many different cancers. These antibodies target molecules such as programmed cell death 1 (PD-1) or cytotoxic T lymphocyte associated protein 4 (CTLA-4) which normally function to limit immune activity. Treatment with ICIs reactivates T cells to destroy tumor cells in a highly specific manner, which in some patients, results in dramatic remissions and durable disease control. Over the last decade, much effort has been directed at characterizing factors that drive efficacy and resistance to ICI therapy. Food and Drug Administration (FDA)-approved biomarkers for ICI therapy have facilitated more judicious treatment of cancer patients and transformed the field of precision oncology. Yet, adaptive immunity against cancers is complex, and newer data have revealed the potential utility of other biomarkers. In this review, we discuss the utility of currently approved biomarkers and highlight how emerging biomarkers can further improve the identification of patients who benefit from ICIs. | Immune checkpoint inhibitors are a type of cancer treatment that help the body’s own immune system attack tumors. They work by blocking “brakes” on immune cells, allowing them to better find and destroy cancer cells. These treatments can lead to long-lasting benefits for some patients, but not everyone responds the same way. Doctors use certain signs, called biomarkers, to help decide who is most likely to benefit. Some biomarkers are already approved for use, but cancer and the immune system are complex, and researchers are finding new ones that may be even more helpful. This study reviews both the current approved biomarkers and promising new ones. Understanding and using these biomarkers more effectively could help doctors choose the right treatment for each patient, avoid unnecessary side effects, and improve cancer care in the future. | Immune checkpoint inhibitors are a type of cancer treatment that help the immune system recognize and attack cancer cells. While these treatments have led to major improvements for some patients, they do not work for everyone. Doctors use “biomarkers,” or measurable signs in the body, to help decide who is most likely to benefit. Some biomarkers are already approved for use, but they do not fully explain why certain patients respond better than others. This study reviews both the established biomarkers and new ones being researched, aiming to better predict treatment success. By understanding these indicators, doctors may be able to choose the right therapy for each patient, improve survival, and reduce unnecessary side effects. These insights could also guide future research and lead to more personalized cancer care, ensuring that each patient receives the treatment most likely to help them. | Immune checkpoint inhibitors are a type of cancer treatment that help the body’s own immune system attack tumors. They work by blocking certain “brakes” on immune cells, allowing them to find and destroy cancer cells. While some patients experience long-lasting benefits, not everyone responds in the same way. Doctors use special biological signals, called biomarkers, to predict which patients are most likely to benefit. This article explains both the well-established biomarkers already approved for use and new, emerging ones being studied. We describe how these biomarkers are found, what they tell us about a patient’s cancer, and how they could guide treatment choices. By improving the ability to match the right patient with the right treatment, these advances could lead to more effective care, fewer unnecessary side effects, and better outcomes for people with cancer. Our goal is to help guide future research and clinical decisions in this fast-changing field. |
| CancCell | 5 | 10.1016/j.ccell.2025.03.008 | Claude | Immune checkpoint inhibitors (ICIs) have improved outcomes of patients with many different cancers. These antibodies target molecules such as programmed cell death 1 (PD-1) or cytotoxic T lymphocyte associated protein 4 (CTLA-4) which normally function to limit immune activity. Treatment with ICIs reactivates T cells to destroy tumor cells in a highly specific manner, which in some patients, results in dramatic remissions and durable disease control. Over the last decade, much effort has been directed at characterizing factors that drive efficacy and resistance to ICI therapy. Food and Drug Administration (FDA)-approved biomarkers for ICI therapy have facilitated more judicious treatment of cancer patients and transformed the field of precision oncology. Yet, adaptive immunity against cancers is complex, and newer data have revealed the potential utility of other biomarkers. In this review, we discuss the utility of currently approved biomarkers and highlight how emerging biomarkers can further improve the identification of patients who benefit from ICIs. | Cancer treatment has been revolutionized by special medicines called immune checkpoint inhibitors, which help the body's own defense system fight cancer more effectively. These drugs work by removing the "brakes" that normally prevent immune cells from attacking tumors, allowing them to destroy cancer cells with great precision. While these treatments have led to remarkable recoveries in some patients, doctors have struggled to predict who will benefit most from them. Scientists have been working hard to find biological markers in patients' blood or tumors that can help identify the best candidates for these therapies. Currently approved tests have already improved how doctors choose treatments, making cancer care more personalized and effective. However, the immune system's fight against cancer is incredibly complex, and researchers continue discovering new markers that could help even more patients receive the right treatment at the right time. This ongoing research promises to make these powerful cancer-fighting drugs available to more people who need them. | Cancer treatments called immune checkpoint inhibitors help the body's own defense system fight tumors more effectively. These medicines work by removing natural brakes on immune cells, allowing them to better attack cancer. While these treatments have helped many patients live longer and healthier lives, doctors need better ways to predict which patients will benefit most from these therapies. Currently, doctors use specific tests to measure certain markers in tumors that help guide treatment decisions. However, the immune system's fight against cancer is very complex, involving many different factors that scientists are still learning about. This research aims to review the current methods doctors use to select patients for these immune-boosting treatments and explore new ways to identify which patients are most likely to respond well. By improving how doctors choose treatments, researchers hope to help more cancer patients receive the most effective therapy for their specific situation, leading to better outcomes and fewer unnecessary side effects from treatments that might not work for them. | Cancer treatments called immune checkpoint inhibitors help the body's own immune system fight cancer more effectively. These medicines work by removing the natural brakes on immune cells, allowing them to better attack and destroy cancer cells. While these treatments have helped many cancer patients live longer and healthier lives, doctors need better ways to predict which patients will benefit most from these therapies. Currently, doctors use several approved tests to help decide if a patient should receive these treatments, but these tests are not perfect. Scientists are working to discover new and better ways to identify patients who will respond well to immune checkpoint inhibitors. This research is important because it could help doctors choose the best treatment for each patient while avoiding unnecessary side effects for those unlikely to benefit. By improving how we select patients for these powerful cancer treatments, we can provide more personalized care and potentially save more lives while reducing healthcare costs. |
| CancCell | 5 | 10.1016/j.ccell.2025.03.009 | Gemini | Immune checkpoint inhibitors (ICIs) have improved outcomes of patients with many different cancers. These antibodies target molecules such as programmed cell death 1 (PD-1) or cytotoxic T lymphocyte associated protein 4 (CTLA-4) which normally function to limit immune activity. Treatment with ICIs reactivates T cells to destroy tumor cells in a highly specific manner, which in some patients, results in dramatic remissions and durable disease control. Over the last decade, much effort has been directed at characterizing factors that drive efficacy and resistance to ICI therapy. Food and Drug Administration (FDA)-approved biomarkers for ICI therapy have facilitated more judicious treatment of cancer patients and transformed the field of precision oncology. Yet, adaptive immunity against cancers is complex, and newer data have revealed the potential utility of other biomarkers. In this review, we discuss the utility of currently approved biomarkers and highlight how emerging biomarkers can further improve the identification of patients who benefit from ICIs. | New medicines called immune checkpoint inhibitors have shown great promise in treating many cancers by helping the body's own immune system fight the disease. However, these drugs do not work for every patient. This review explains how doctors currently use special tools, called biomarkers, to decide which patients are most likely to benefit from this treatment. The study's purpose is to examine both the established biomarkers that are already used in clinics and new ones that are being developed. By finding better biomarkers, doctors can more accurately select patients for treatment, avoid giving ineffective drugs, and ultimately improve the success of these important cancer therapies. | Cancer treatments called immune checkpoint inhibitors have shown great promise by helping a person's own immune system fight cancer. While these treatments have been very successful for some, they do not work for everyone. This study looks at the different biological markers that doctors currently use to decide which patients will benefit most from this therapy. The researchers also highlight new markers that are being discovered, which could further improve how we select patients for treatment. The goal is to help doctors make more informed decisions, ensuring the right patients receive this powerful therapy. Ultimately, this research will lead to better, more personalized cancer care for a wider range of people. | Cancer treatments called immune checkpoint inhibitors are powerful medicines that help a patient's own immune system find and destroy cancer cells. While these treatments are very successful for some people, they do not work for everyone. To help doctors decide who will benefit most, we use clues from the patient’s body, known as biomarkers. This research reviews the clues we already use and highlights new, promising ones that could make these treatments even more successful. By understanding which patients are most likely to respond, doctors can choose the best possible treatment for each person's cancer. This helps to improve how we use these important medicines and moves us closer to more personalized cancer care. |
| CancCell | 5 | 10.1016/j.ccell.2025.03.010 | DeepSeek | Immune checkpoint inhibitors (ICIs) have improved outcomes of patients with many different cancers. These antibodies target molecules such as programmed cell death 1 (PD-1) or cytotoxic T lymphocyte associated protein 4 (CTLA-4) which normally function to limit immune activity. Treatment with ICIs reactivates T cells to destroy tumor cells in a highly specific manner, which in some patients, results in dramatic remissions and durable disease control. Over the last decade, much effort has been directed at characterizing factors that drive efficacy and resistance to ICI therapy. Food and Drug Administration (FDA)-approved biomarkers for ICI therapy have facilitated more judicious treatment of cancer patients and transformed the field of precision oncology. Yet, adaptive immunity against cancers is complex, and newer data have revealed the potential utility of other biomarkers. In this review, we discuss the utility of currently approved biomarkers and highlight how emerging biomarkers can further improve the identification of patients who benefit from ICIs. | Immune checkpoint inhibitors (ICIs) are powerful cancer drugs that help the body’s immune system fight tumors. While these treatments work well for some patients, others don’t respond as strongly. Scientists have found certain markers—like PD-1 and CTLA-4—that help doctors predict who might benefit from ICI therapy. However, cancer immunity is complicated, and researchers are discovering new markers that could make these predictions even better. This review explains the currently approved markers and explores promising new ones. By improving how we match these treatments to the right patients, we can boost their effectiveness and help more people with cancer. | Cancer treatment has improved thanks to drugs called immune checkpoint inhibitors (ICIs), which help the immune system fight tumors. While some patients respond very well, others do not, so doctors use special markers (biomarkers) to predict who will benefit. This review explains the currently approved biomarkers and explores new ones that could make treatment more precise. By better matching patients with the right therapy, these findings may improve outcomes and reduce unnecessary side effects. This research is important because it could help more people get the most effective cancer treatment for their specific condition. | Cancer treatments called immune checkpoint inhibitors (ICIs) help the body’s immune system fight tumors, leading to long-term remission for some patients. Doctors use specific tests (biomarkers) to predict which patients will benefit most from these drugs, but cancer immunity is complex, and not all patients respond. This review explains how current biomarker tests guide treatment decisions and explores new, promising biomarkers that could help more patients receive the right therapy at the right time. By improving how we match patients with ICI treatments, these advances could lead to better outcomes and fewer side effects. |
| CancCell | 5 | 10.1016/j.ccell.2025.03.011 | Grok | Immune checkpoint inhibitors (ICIs) have improved outcomes of patients with many different cancers. These antibodies target molecules such as programmed cell death 1 (PD-1) or cytotoxic T lymphocyte associated protein 4 (CTLA-4) which normally function to limit immune activity. Treatment with ICIs reactivates T cells to destroy tumor cells in a highly specific manner, which in some patients, results in dramatic remissions and durable disease control. Over the last decade, much effort has been directed at characterizing factors that drive efficacy and resistance to ICI therapy. Food and Drug Administration (FDA)-approved biomarkers for ICI therapy have facilitated more judicious treatment of cancer patients and transformed the field of precision oncology. Yet, adaptive immunity against cancers is complex, and newer data have revealed the potential utility of other biomarkers. In this review, we discuss the utility of currently approved biomarkers and highlight how emerging biomarkers can further improve the identification of patients who benefit from ICIs. | Immune checkpoint inhibitors are drugs that help the body's immune system fight cancer by waking up T cells to attack tumors. This has led to big improvements for many cancer patients, but not everyone benefits, so doctors need better ways to pick who should get these treatments. In this review, the authors look at approved tests, like those from the Food and Drug Administration, that help guide treatment decisions, and explore new ones that could work even better. Their goal is to show how these markers can make cancer care more precise and effective. By highlighting these tools, the work could help researchers develop smarter ways to treat cancer, leading to longer lives and fewer side effects for more people. | Immune checkpoint inhibitors are drugs that help the body's immune system fight cancer by waking up T cells to attack tumors. This has led to big improvements for many patients, with some seeing long-lasting results. However, not everyone benefits, so doctors need ways to predict who will respond well. This review looks at approved tests, like those from the Food and Drug Administration, that guide treatment choices and make cancer care more precise. It also explores new signs in the body that could help spot even more patients who might succeed with these therapies. By sharing these insights, the work aims to help researchers and doctors pick the right treatments, saving lives and reducing side effects for people facing cancer. | Immune checkpoint inhibitors are drugs that help the body's immune system fight cancer by waking up T cells to attack tumors. This has led to big improvements for many cancer patients, with some seeing long-lasting results. However, not everyone benefits, so researchers are working to find better ways to predict who will respond well. This review looks at approved tests, like those from the Food and Drug Administration, that guide treatment choices, and explores new markers that could make decisions even smarter. By highlighting these tools, the work aims to help doctors pick the right patients for these therapies, leading to more successful outcomes and advancing cancer care for everyone. |

**Table 3s: GAI generated Lay abstracts and summaries (Test 1-3), using a “Generic Prompt” in the General purpose LLMs.**

| **Journal** | **Abstract** | **DOI** | **Arm** | **Original Abstract** | **Test 1** | **Test 2** | **Test 3** |
| --- | --- | --- | --- | --- | --- | --- | --- |
| JCO | 1 | 10.1200/JCO-24-02008 | Open AI | PURPOSETo our knowledge, the ACHILLES/TORG1834 trial is the first randomized study comparing afatinib and chemotherapy in patients with non-small cell lung cancer (NSCLC) harboring sensitizing uncommon epidermal growth factor receptor (EGFR) mutations.METHODSThis randomized, open-label study was performed at 51 Japanese institutions and recruited treatment-na & iuml;ve patients with nonsquamous NSCLC with uncommon EGFR mutations, excluding exon 20 insertions and T790M mutations. Patients were randomly assigned 2:1 to receive afatinib (30 or 40 mg orally, at the treating physician's discretion) or a combination of platinum (cisplatin or carboplatin) and pemetrexed, followed by pemetrexed maintenance. The primary end point was progression-free survival (PFS). Secondary end points included objective response rate (ORR), overall survival, and safety. A prespecified interim analysis was planned to provide clinically meaningful information promptly, along with a crossover recommendation if necessary.RESULTSA total of 109 patients were enrolled between March 2019 and February 2023. In the interim analysis, the Data and Safety Monitoring Committee recommended early study termination. The median PFS was significantly longer in patients receiving afatinib than in those undergoing chemotherapy (10.6 v 5.7 months; hazard ratio, 0.421 [95% CI, 0.251 to 0.706]; P = .0010). ORRs to afatinib were similar across the overall population and among participants with major uncommon (G719X, L861Q, and S768I), compound, and other mutations (61.7%, 55.8%, 72.7%, and 60.0%, respectively). The most common grade 3 or higher adverse events were diarrhea, paronychia, and rash for afatinib, and appetite loss and nausea for chemotherapy.CONCLUSIONAfatinib should be considered the standard initial therapy for patients with NSCLC with sensitizing uncommon EGFR mutations. | The ACHILLES/TORG1834 trial compared afatinib with chemotherapy in Japanese patients with a certain type of lung cancer (NSCLC) that has uncommon EGFR mutations. The study found that patients taking afatinib lived longer without their cancer worsening (10.6 months vs. 5.7 months) than those on chemotherapy. Response rates were high across different mutation types, and side effects differed between treatments. Based on these results, afatinib is recommended as the first treatment choice for these patients. | The ACHILLES/TORG1834 trial compared afatinib with chemotherapy in Japanese patients with a specific type of non-small cell lung cancer (NSCLC) caused by uncommon EGFR mutations. In this study, 109 untreated patients were randomly given either afatinib or chemotherapy. Results showed that afatinib nearly doubled the time before the cancer worsened (10.6 months vs 5.7 months) and had a high response rate across different mutation types. Side effects differed between treatments, but both were manageable. Researchers concluded that afatinib should be the first choice for treating these patients. | The ACHILLES/TORG1834 trial compared the drug afatinib with standard chemotherapy in patients in Japan who had a specific type of lung cancer (NSCLC) with uncommon EGFR mutations. In this study of 109 untreated patients, afatinib more than doubled the time before the cancer worsened compared to chemotherapy (10.6 vs. 5.7 months) and had similar response rates across different mutation types. Side effects differed between treatments, with diarrhea, nail infections, and rash more common with afatinib, and appetite loss and nausea more common with chemotherapy. The findings suggest afatinib should be the preferred first treatment for these patients. |
| JCO | 1 | 10.1200/JCO-24-02009 | Claude | PURPOSETo our knowledge, the ACHILLES/TORG1834 trial is the first randomized study comparing afatinib and chemotherapy in patients with non-small cell lung cancer (NSCLC) harboring sensitizing uncommon epidermal growth factor receptor (EGFR) mutations.METHODSThis randomized, open-label study was performed at 51 Japanese institutions and recruited treatment-na & iuml;ve patients with nonsquamous NSCLC with uncommon EGFR mutations, excluding exon 20 insertions and T790M mutations. Patients were randomly assigned 2:1 to receive afatinib (30 or 40 mg orally, at the treating physician's discretion) or a combination of platinum (cisplatin or carboplatin) and pemetrexed, followed by pemetrexed maintenance. The primary end point was progression-free survival (PFS). Secondary end points included objective response rate (ORR), overall survival, and safety. A prespecified interim analysis was planned to provide clinically meaningful information promptly, along with a crossover recommendation if necessary.RESULTSA total of 109 patients were enrolled between March 2019 and February 2023. In the interim analysis, the Data and Safety Monitoring Committee recommended early study termination. The median PFS was significantly longer in patients receiving afatinib than in those undergoing chemotherapy (10.6 v 5.7 months; hazard ratio, 0.421 [95% CI, 0.251 to 0.706]; P = .0010). ORRs to afatinib were similar across the overall population and among participants with major uncommon (G719X, L861Q, and S768I), compound, and other mutations (61.7%, 55.8%, 72.7%, and 60.0%, respectively). The most common grade 3 or higher adverse events were diarrhea, paronychia, and rash for afatinib, and appetite loss and nausea for chemotherapy.CONCLUSIONAfatinib should be considered the standard initial therapy for patients with NSCLC with sensitizing uncommon EGFR mutations. | This Japanese study compared two treatments for lung cancer patients with rare genetic mutations in the EGFR gene. Researchers gave 109 patients either afatinib (a targeted drug taken by mouth) or standard chemotherapy. The results showed that afatinib worked much better - patients taking afatinib went longer without their cancer getting worse (10.6 months versus 5.7 months) and had fewer severe side effects. Based on these findings, the researchers recommend afatinib as the preferred first treatment for lung cancer patients with these specific rare mutations. | This Japanese study compared two treatments for lung cancer patients with rare genetic mutations. Researchers gave 109 patients either a targeted drug called afatinib or standard chemotherapy. The results showed that afatinib worked much better - patients lived longer without their cancer getting worse (10.6 months vs 5.7 months) and had good response rates regardless of their specific mutation type. The study was stopped early because afatinib showed such clear benefits. Based on these findings, doctors should consider afatinib as the first treatment choice for lung cancer patients with these uncommon genetic mutations. | This Japanese study compared two treatments for lung cancer patients with rare genetic mutations called uncommon EGFR mutations. Researchers gave 109 patients either a targeted drug called afatinib or standard chemotherapy. The study found that afatinib worked much better - patients taking it went an average of 10.6 months before their cancer got worse, compared to only 5.7 months for those getting chemotherapy. About 62% of patients responded well to afatinib, and while it caused some side effects like diarrhea and skin problems, the results were so promising that the study was stopped early. The researchers concluded that afatinib should be the preferred first treatment for lung cancer patients with these specific rare mutations. |
| JCO | 1 | 10.1200/JCO-24-02010 | Gemini | PURPOSETo our knowledge, the ACHILLES/TORG1834 trial is the first randomized study comparing afatinib and chemotherapy in patients with non-small cell lung cancer (NSCLC) harboring sensitizing uncommon epidermal growth factor receptor (EGFR) mutations.METHODSThis randomized, open-label study was performed at 51 Japanese institutions and recruited treatment-na & iuml;ve patients with nonsquamous NSCLC with uncommon EGFR mutations, excluding exon 20 insertions and T790M mutations. Patients were randomly assigned 2:1 to receive afatinib (30 or 40 mg orally, at the treating physician's discretion) or a combination of platinum (cisplatin or carboplatin) and pemetrexed, followed by pemetrexed maintenance. The primary end point was progression-free survival (PFS). Secondary end points included objective response rate (ORR), overall survival, and safety. A prespecified interim analysis was planned to provide clinically meaningful information promptly, along with a crossover recommendation if necessary.RESULTSA total of 109 patients were enrolled between March 2019 and February 2023. In the interim analysis, the Data and Safety Monitoring Committee recommended early study termination. The median PFS was significantly longer in patients receiving afatinib than in those undergoing chemotherapy (10.6 v 5.7 months; hazard ratio, 0.421 [95% CI, 0.251 to 0.706]; P = .0010). ORRs to afatinib were similar across the overall population and among participants with major uncommon (G719X, L861Q, and S768I), compound, and other mutations (61.7%, 55.8%, 72.7%, and 60.0%, respectively). The most common grade 3 or higher adverse events were diarrhea, paronychia, and rash for afatinib, and appetite loss and nausea for chemotherapy.CONCLUSIONAfatinib should be considered the standard initial therapy for patients with NSCLC with sensitizing uncommon EGFR mutations. | In a recent study called ACHILLES/TORG1834, researchers compared afatinib to chemotherapy as a first treatment for patients with a specific type of lung cancer. The study found that patients who took afatinib lived significantly longer without their cancer getting worse compared to those who received chemotherapy. The median time was 10.6 months for afatinib and 5.7 months for chemotherapy. This positive result led the researchers to stop the study early and conclude that afatinib should be the standard first treatment for these patients. While both treatments had side effects, they were different, with afatinib causing more issues like diarrhea and rash, and chemotherapy causing more nausea and loss of appetite. | In a recent clinical trial called ACHILLES/TORG1834, researchers compared the effectiveness of the drug afatinib to standard chemotherapy for patients with a specific type of lung cancer called non-small cell lung cancer (NSCLC) that has uncommon mutations in the EGFR gene. The study found that patients who took afatinib lived significantly longer without their cancer getting worse compared to those who received chemotherapy, with a median progression-free survival of 10.6 months versus 5.7 months. Based on these strong results, researchers concluded that afatinib should be considered the new standard first treatment for patients with this specific form of lung cancer. | In a recent clinical trial called ACHILLES/TORG1834, researchers compared the effectiveness of the drug afatinib to standard chemotherapy for patients with a specific type of lung cancer called non-small cell lung cancer (NSCLC) that has rare EGFR gene mutations. The study was stopped early because afatinib was clearly more effective. Patients who received afatinib lived significantly longer without their cancer getting worse (10.6 months) compared to those who received chemotherapy (5.7 months). Afatinib also led to higher rates of tumor shrinkage. While both treatments had side effects, they were different for each group. The results show that afatinib should be the new standard first-line treatment for patients with these specific, uncommon EGFR mutations. |
| JCO | 1 | 10.1200/JCO-24-02011 | DeepSeek | PURPOSETo our knowledge, the ACHILLES/TORG1834 trial is the first randomized study comparing afatinib and chemotherapy in patients with non-small cell lung cancer (NSCLC) harboring sensitizing uncommon epidermal growth factor receptor (EGFR) mutations.METHODSThis randomized, open-label study was performed at 51 Japanese institutions and recruited treatment-na & iuml;ve patients with nonsquamous NSCLC with uncommon EGFR mutations, excluding exon 20 insertions and T790M mutations. Patients were randomly assigned 2:1 to receive afatinib (30 or 40 mg orally, at the treating physician's discretion) or a combination of platinum (cisplatin or carboplatin) and pemetrexed, followed by pemetrexed maintenance. The primary end point was progression-free survival (PFS). Secondary end points included objective response rate (ORR), overall survival, and safety. A prespecified interim analysis was planned to provide clinically meaningful information promptly, along with a crossover recommendation if necessary.RESULTSA total of 109 patients were enrolled between March 2019 and February 2023. In the interim analysis, the Data and Safety Monitoring Committee recommended early study termination. The median PFS was significantly longer in patients receiving afatinib than in those undergoing chemotherapy (10.6 v 5.7 months; hazard ratio, 0.421 [95% CI, 0.251 to 0.706]; P = .0010). ORRs to afatinib were similar across the overall population and among participants with major uncommon (G719X, L861Q, and S768I), compound, and other mutations (61.7%, 55.8%, 72.7%, and 60.0%, respectively). The most common grade 3 or higher adverse events were diarrhea, paronychia, and rash for afatinib, and appetite loss and nausea for chemotherapy.CONCLUSIONAfatinib should be considered the standard initial therapy for patients with NSCLC with sensitizing uncommon EGFR mutations. | The ACHILLES/TORG1834 trial compared afatinib to chemotherapy in untreated non-small cell lung cancer (NSCLC) patients with rare EGFR mutations (excluding exon 20 insertions and T790M). Afatinib significantly improved progression-free survival (10.6 vs. 5.7 months) and had better response rates than chemotherapy, with manageable side effects like diarrhea and rash. The trial was stopped early due to clear benefits, establishing afatinib as the preferred first treatment for NSCLC with these uncommon EGFR mutations. | The ACHILLES/TORG1834 trial compared afatinib to chemotherapy in untreated non-small cell lung cancer (NSCLC) patients with rare EGFR mutations (excluding exon 20 insertions and T790M). Afatinib significantly improved progression-free survival (10.6 vs. 5.7 months) and had better response rates across mutation types, though it caused more diarrhea and skin side effects, while chemotherapy led to appetite loss and nausea. The trial was stopped early due to clear benefits, establishing afatinib as the preferred first treatment for these patients. | The ACHILLES/TORG1834 trial compared afatinib (a targeted drug) to standard chemotherapy in untreated non-small cell lung cancer (NSCLC) patients with rare EGFR mutations. The study showed afatinib nearly doubled progression-free survival (10.6 vs. 5.7 months) and had higher response rates, with manageable side effects like diarrhea and rash. Due to these strong results, the trial was stopped early, and afatinib is now recommended as the first-line treatment for NSCLC with these uncommon EGFR mutations. |
| JCO | 1 | 10.1200/JCO-24-02012 | Grok | PURPOSETo our knowledge, the ACHILLES/TORG1834 trial is the first randomized study comparing afatinib and chemotherapy in patients with non-small cell lung cancer (NSCLC) harboring sensitizing uncommon epidermal growth factor receptor (EGFR) mutations.METHODSThis randomized, open-label study was performed at 51 Japanese institutions and recruited treatment-na & iuml;ve patients with nonsquamous NSCLC with uncommon EGFR mutations, excluding exon 20 insertions and T790M mutations. Patients were randomly assigned 2:1 to receive afatinib (30 or 40 mg orally, at the treating physician's discretion) or a combination of platinum (cisplatin or carboplatin) and pemetrexed, followed by pemetrexed maintenance. The primary end point was progression-free survival (PFS). Secondary end points included objective response rate (ORR), overall survival, and safety. A prespecified interim analysis was planned to provide clinically meaningful information promptly, along with a crossover recommendation if necessary.RESULTSA total of 109 patients were enrolled between March 2019 and February 2023. In the interim analysis, the Data and Safety Monitoring Committee recommended early study termination. The median PFS was significantly longer in patients receiving afatinib than in those undergoing chemotherapy (10.6 v 5.7 months; hazard ratio, 0.421 [95% CI, 0.251 to 0.706]; P = .0010). ORRs to afatinib were similar across the overall population and among participants with major uncommon (G719X, L861Q, and S768I), compound, and other mutations (61.7%, 55.8%, 72.7%, and 60.0%, respectively). The most common grade 3 or higher adverse events were diarrhea, paronychia, and rash for afatinib, and appetite loss and nausea for chemotherapy.CONCLUSIONAfatinib should be considered the standard initial therapy for patients with NSCLC with sensitizing uncommon EGFR mutations. | The ACHILLES/TORG1834 study, conducted at 51 Japanese centers, compared afatinib (a targeted therapy) with chemotherapy in 109 patients with non-small cell lung cancer (NSCLC) having uncommon EGFR mutations. Patients were randomly assigned to receive either afatinib or chemotherapy (platinum and pemetrexed). The study found that afatinib significantly extended progression-free survival (10.6 months vs. 5.7 months for chemotherapy) and had similar response rates across different mutation types. Due to these results, the study was stopped early, and afatinib was recommended as the standard initial treatment for NSCLC patients with these mutations, despite side effects like diarrhea and rash for afatinib, and appetite loss and nausea for chemotherapy. | The ACHILLES/TORG1834 study was the first to compare afatinib, a targeted drug, with chemotherapy in patients with non-small cell lung cancer (NSCLC) who have uncommon EGFR mutations. Conducted across 51 Japanese centers, the study involved 109 treatment-naïve patients randomly assigned to receive either afatinib or chemotherapy (platinum and pemetrexed). The results showed that afatinib significantly extended progression-free survival (10.6 months vs. 5.7 months for chemotherapy) and had similar response rates across different mutation types. Common side effects included diarrhea and rash for afatinib, and appetite loss and nausea for chemotherapy. The study concluded that afatinib should be the preferred initial treatment for these patients. | The ACHILLES/TORG1834 study, conducted at 51 Japanese institutions, compared afatinib (a targeted therapy) with chemotherapy in 109 patients with non-small cell lung cancer (NSCLC) who had uncommon EGFR mutations (excluding certain types). Patients were randomly assigned to receive either afatinib or chemotherapy, with afatinib doses chosen by doctors (30 or 40 mg). The study found that afatinib significantly extended the time before the cancer worsened (10.6 months vs. 5.7 months for chemotherapy) and had similar response rates across different mutation types. Side effects like diarrhea and rash were common with afatinib, while chemotherapy caused more appetite loss and nausea. The study concluded that afatinib should be the preferred initial treatment for these patients. |
| JCO | 2 | 10.1200/JCO-24-01270 | Open AI | PURPOSE Mobocertinib is an oral epidermal growth factor receptor (EGFR) tyrosine kinase inhibitor that targets EGFR exon 20 insertion (ex20ins) mutations in non-small cell lung cancer (NSCLC). This open-label, phase III trial (EXCLAIM-2, ClinicalTrials.gov identifier: NCT04129502) compared mobocertinib versus platinum-based chemotherapy as first-line treatment of EGFR ex20ins+ advanced/metastatic NSCLC. METHODS Patients with treatment-naive EGFR ex20ins+ locally advanced/metastatic NSCLC were randomly assigned 1:1 to mobocertinib 160 mg once daily or pemetrexed plus cisplatin or carboplatin every 3 weeks for four cycles followed by maintenance pemetrexed. The primary end point was progression-free survival (PFS) by blinded independent central review (BICR), with planned interim analysis (IA) after approximately 70% of 227 expected PFS events. RESULTS A total of 354 patients were randomly assigned (mobocertinib: n = 179; chemotherapy: n = 175). Baseline characteristics were balanced between arms. At IA (cutoff: April 4, 2023), the median PFS per BICR was 9.6 months in each treatment arm (hazard ratio [HR], 1.04 [95% CI, 0.77 to 1.39]; P = .803). The primary end point crossed the prespecified futility boundary (HR > 1). The confirmed objective response rate (95% CI) per BICR was 32% (26 to 40) with mobocertinib versus 30% (24 to 38) with chemotherapy; the median duration of response was 12.0 versus 8.4 months. Quality-of-life assessments indicated clinically meaningful delays in time to deterioration of lung cancer symptoms, cognitive function, and constipation with mobocertinib versus chemotherapy. Grade >= 3 adverse events in >5% of patients (mobocertinib, chemotherapy) were diarrhea (20%, 1%), anemia (6%, 10%), increased lipase (6%, 0%), and decreased neutrophil count (1%, 7%). CONCLUSION The EXCLAIM-2 trial did not meet its primary end point. The efficacy of mobocertinib was not superior to platinum-based chemotherapy for first-line treatment of patients with EGFR ex20ins+ advanced/metastatic NSCLC. | A phase III trial tested whether mobocertinib, a targeted oral drug, worked better than standard platinum-based chemotherapy as the first treatment for advanced non-small cell lung cancer with EGFR exon 20 insertion mutations. The study found no difference in how long patients lived without their cancer worsening (about 9.6 months for both groups) or in response rates. Mobocertinib did improve some quality-of-life measures but caused more diarrhea, while chemotherapy caused more anemia and low white blood cells. Overall, mobocertinib was not more effective than chemotherapy. | The EXCLAIM-2 trial tested whether mobocertinib, an oral drug targeting EGFR exon 20 insertion mutations, worked better than standard platinum-based chemotherapy for people with advanced or metastatic non-small cell lung cancer who had not yet been treated. In 354 patients, both treatments had the same median time before the cancer worsened (9.6 months) and similar response rates. Mobocertinib showed longer-lasting responses and better quality-of-life measures for some symptoms, but it did not outperform chemotherapy overall, so the main goal of the study was not met. | This study compared mobocertinib, an oral targeted cancer drug, with standard platinum-based chemotherapy as the first treatment for a specific type of advanced lung cancer (EGFR exon 20 insertion mutation). In 354 patients, both treatments had the same median time before the cancer worsened (9.6 months), and response rates were similar. However, mobocertinib patients had longer-lasting responses, better quality-of-life outcomes for some symptoms, and different side effect patterns. Overall, mobocertinib was not more effective than chemotherapy in stopping cancer growth. |
| JCO | 2 | 10.1200/JCO-24-01271 | Claude | PURPOSE Mobocertinib is an oral epidermal growth factor receptor (EGFR) tyrosine kinase inhibitor that targets EGFR exon 20 insertion (ex20ins) mutations in non-small cell lung cancer (NSCLC). This open-label, phase III trial (EXCLAIM-2, ClinicalTrials.gov identifier: NCT04129502) compared mobocertinib versus platinum-based chemotherapy as first-line treatment of EGFR ex20ins+ advanced/metastatic NSCLC. METHODS Patients with treatment-naive EGFR ex20ins+ locally advanced/metastatic NSCLC were randomly assigned 1:1 to mobocertinib 160 mg once daily or pemetrexed plus cisplatin or carboplatin every 3 weeks for four cycles followed by maintenance pemetrexed. The primary end point was progression-free survival (PFS) by blinded independent central review (BICR), with planned interim analysis (IA) after approximately 70% of 227 expected PFS events. RESULTS A total of 354 patients were randomly assigned (mobocertinib: n = 179; chemotherapy: n = 175). Baseline characteristics were balanced between arms. At IA (cutoff: April 4, 2023), the median PFS per BICR was 9.6 months in each treatment arm (hazard ratio [HR], 1.04 [95% CI, 0.77 to 1.39]; P = .803). The primary end point crossed the prespecified futility boundary (HR > 1). The confirmed objective response rate (95% CI) per BICR was 32% (26 to 40) with mobocertinib versus 30% (24 to 38) with chemotherapy; the median duration of response was 12.0 versus 8.4 months. Quality-of-life assessments indicated clinically meaningful delays in time to deterioration of lung cancer symptoms, cognitive function, and constipation with mobocertinib versus chemotherapy. Grade >= 3 adverse events in >5% of patients (mobocertinib, chemotherapy) were diarrhea (20%, 1%), anemia (6%, 10%), increased lipase (6%, 0%), and decreased neutrophil count (1%, 7%). CONCLUSION The EXCLAIM-2 trial did not meet its primary end point. The efficacy of mobocertinib was not superior to platinum-based chemotherapy for first-line treatment of patients with EGFR ex20ins+ advanced/metastatic NSCLC. | Mobocertinib is a pill-form cancer drug designed to treat a specific type of lung cancer with a particular genetic mutation. In this large clinical trial, researchers compared mobocertinib to standard chemotherapy as the first treatment for 354 patients with advanced lung cancer. Both treatments worked equally well, with patients living about 9.6 months before their cancer got worse, and about 30-32% of patients saw their tumors shrink. However, mobocertinib caused fewer serious blood-related side effects but more severe diarrhea compared to chemotherapy, and patients taking mobocertinib had better quality of life with less worsening of lung cancer symptoms. The study concluded that mobocertinib was not better than standard chemotherapy for treating this type of lung cancer. | This study tested a new oral cancer drug called mobocertinib against standard chemotherapy as the first treatment for people with a specific type of advanced lung cancer. Researchers randomly gave 354 patients either the new drug or chemotherapy and measured how long it took for their cancer to get worse. Both treatments worked equally well, with patients living about 9.6 months before their cancer progressed. The new drug didn't work better than chemotherapy, so the study didn't meet its main goal of proving the oral medication was superior to standard treatment. | This study tested a new lung cancer drug called mobocertinib against standard chemotherapy in patients with a specific type of lung cancer mutation. Researchers gave 354 patients either the new drug or chemotherapy as their first treatment and tracked how long it took for their cancer to get worse. Both treatments worked equally well - patients lived about 9.6 months without their cancer progressing, regardless of which treatment they received. The new drug didn't work better than chemotherapy, so the study failed to prove that mobocertinib should replace standard treatment for this type of lung cancer. |
| JCO | 2 | 10.1200/JCO-24-01272 | Gemini | PURPOSE Mobocertinib is an oral epidermal growth factor receptor (EGFR) tyrosine kinase inhibitor that targets EGFR exon 20 insertion (ex20ins) mutations in non-small cell lung cancer (NSCLC). This open-label, phase III trial (EXCLAIM-2, ClinicalTrials.gov identifier: NCT04129502) compared mobocertinib versus platinum-based chemotherapy as first-line treatment of EGFR ex20ins+ advanced/metastatic NSCLC. METHODS Patients with treatment-naive EGFR ex20ins+ locally advanced/metastatic NSCLC were randomly assigned 1:1 to mobocertinib 160 mg once daily or pemetrexed plus cisplatin or carboplatin every 3 weeks for four cycles followed by maintenance pemetrexed. The primary end point was progression-free survival (PFS) by blinded independent central review (BICR), with planned interim analysis (IA) after approximately 70% of 227 expected PFS events. RESULTS A total of 354 patients were randomly assigned (mobocertinib: n = 179; chemotherapy: n = 175). Baseline characteristics were balanced between arms. At IA (cutoff: April 4, 2023), the median PFS per BICR was 9.6 months in each treatment arm (hazard ratio [HR], 1.04 [95% CI, 0.77 to 1.39]; P = .803). The primary end point crossed the prespecified futility boundary (HR > 1). The confirmed objective response rate (95% CI) per BICR was 32% (26 to 40) with mobocertinib versus 30% (24 to 38) with chemotherapy; the median duration of response was 12.0 versus 8.4 months. Quality-of-life assessments indicated clinically meaningful delays in time to deterioration of lung cancer symptoms, cognitive function, and constipation with mobocertinib versus chemotherapy. Grade >= 3 adverse events in >5% of patients (mobocertinib, chemotherapy) were diarrhea (20%, 1%), anemia (6%, 10%), increased lipase (6%, 0%), and decreased neutrophil count (1%, 7%). CONCLUSION The EXCLAIM-2 trial did not meet its primary end point. The efficacy of mobocertinib was not superior to platinum-based chemotherapy for first-line treatment of patients with EGFR ex20ins+ advanced/metastatic NSCLC. | The EXCLAIM-2 trial compared the drug mobocertinib to standard chemotherapy as a first treatment for patients with a specific type of lung cancer. The study found that mobocertinib was not more effective than chemotherapy at preventing the cancer from getting worse. Both treatments had similar results in this regard, with patients living about 9.6 months before the cancer progressed. While mobocertinib did not improve survival rates, it did cause fewer serious side effects and delayed the worsening of lung cancer symptoms and other issues, such as constipation, compared to chemotherapy. | The EXCLAIM-2 trial compared the drug mobocertinib to standard platinum-based chemotherapy as a first treatment for patients with a specific type of lung cancer called EGFR exon 20 insertion-positive non-small cell lung cancer. The study found that mobocertinib was not more effective than chemotherapy in slowing the cancer's growth. Both treatments showed a similar duration of progression-free survival (9.6 months), and the percentage of patients whose tumors shrank was also comparable. While mobocertinib did not improve survival outcomes, it was associated with fewer severe side effects and seemed to delay the worsening of some symptoms and improve quality of life. | The EXCLAIM-2 trial compared the drug mobocertinib to standard chemotherapy as a first treatment for patients with a specific type of lung cancer. The study found that patients taking mobocertinib did not live longer without their cancer getting worse than those on chemotherapy. Both treatments had a similar effect on slowing down the disease. While mobocertinib didn't perform better in this main goal, it did show some benefits in improving patients' quality of life by delaying symptoms like shortness of breath and constipation. The trial concluded that mobocertinib is not a superior first-line treatment to chemotherapy for this condition. |
| JCO | 2 | 10.1200/JCO-24-01273 | DeepSeek | PURPOSE Mobocertinib is an oral epidermal growth factor receptor (EGFR) tyrosine kinase inhibitor that targets EGFR exon 20 insertion (ex20ins) mutations in non-small cell lung cancer (NSCLC). This open-label, phase III trial (EXCLAIM-2, ClinicalTrials.gov identifier: NCT04129502) compared mobocertinib versus platinum-based chemotherapy as first-line treatment of EGFR ex20ins+ advanced/metastatic NSCLC. METHODS Patients with treatment-naive EGFR ex20ins+ locally advanced/metastatic NSCLC were randomly assigned 1:1 to mobocertinib 160 mg once daily or pemetrexed plus cisplatin or carboplatin every 3 weeks for four cycles followed by maintenance pemetrexed. The primary end point was progression-free survival (PFS) by blinded independent central review (BICR), with planned interim analysis (IA) after approximately 70% of 227 expected PFS events. RESULTS A total of 354 patients were randomly assigned (mobocertinib: n = 179; chemotherapy: n = 175). Baseline characteristics were balanced between arms. At IA (cutoff: April 4, 2023), the median PFS per BICR was 9.6 months in each treatment arm (hazard ratio [HR], 1.04 [95% CI, 0.77 to 1.39]; P = .803). The primary end point crossed the prespecified futility boundary (HR > 1). The confirmed objective response rate (95% CI) per BICR was 32% (26 to 40) with mobocertinib versus 30% (24 to 38) with chemotherapy; the median duration of response was 12.0 versus 8.4 months. Quality-of-life assessments indicated clinically meaningful delays in time to deterioration of lung cancer symptoms, cognitive function, and constipation with mobocertinib versus chemotherapy. Grade >= 3 adverse events in >5% of patients (mobocertinib, chemotherapy) were diarrhea (20%, 1%), anemia (6%, 10%), increased lipase (6%, 0%), and decreased neutrophil count (1%, 7%). CONCLUSION The EXCLAIM-2 trial did not meet its primary end point. The efficacy of mobocertinib was not superior to platinum-based chemotherapy for first-line treatment of patients with EGFR ex20ins+ advanced/metastatic NSCLC. | The EXCLAIM-2 trial compared the oral drug mobocertinib to standard platinum-based chemotherapy as a first treatment for advanced lung cancer with a specific EGFR mutation (exon 20 insertion). The study found no significant difference in progression-free survival between the two treatments (both had a median of 9.6 months). While mobocertinib showed slightly better response duration and quality-of-life benefits (like slower symptom worsening), it also caused more severe side effects like diarrhea. The trial concluded that mobocertinib was not better than chemotherapy as a first-line treatment for this type of lung cancer. | The EXCLAIM-2 trial tested mobocertinib, a targeted pill for advanced lung cancer with EGFR exon 20 mutations, against standard platinum-based chemotherapy as a first treatment. The study found no significant difference in how long patients lived without their cancer worsening (9.6 months for both treatments). Mobocertinib showed slightly better symptom control and quality-of-life benefits but caused more side effects like diarrhea. Overall, it was not better than chemotherapy as an initial treatment for this type of lung cancer. | The EXCLAIM-2 trial compared the effectiveness of mobocertinib (a targeted pill) versus platinum-based chemotherapy as a first treatment for advanced lung cancer with a specific EGFR mutation (exon 20 insertion). The study found no difference in progression-free survival between the two treatments (both had a median of 9.6 months). While mobocertinib showed slightly longer response duration and better quality-of-life outcomes, it also caused more severe side effects like diarrhea. The trial concluded that mobocertinib was not better than chemotherapy as a first-line treatment for this type of lung cancer. |
| JCO | 2 | 10.1200/JCO-24-01274 | Grok | PURPOSE Mobocertinib is an oral epidermal growth factor receptor (EGFR) tyrosine kinase inhibitor that targets EGFR exon 20 insertion (ex20ins) mutations in non-small cell lung cancer (NSCLC). This open-label, phase III trial (EXCLAIM-2, ClinicalTrials.gov identifier: NCT04129502) compared mobocertinib versus platinum-based chemotherapy as first-line treatment of EGFR ex20ins+ advanced/metastatic NSCLC. METHODS Patients with treatment-naive EGFR ex20ins+ locally advanced/metastatic NSCLC were randomly assigned 1:1 to mobocertinib 160 mg once daily or pemetrexed plus cisplatin or carboplatin every 3 weeks for four cycles followed by maintenance pemetrexed. The primary end point was progression-free survival (PFS) by blinded independent central review (BICR), with planned interim analysis (IA) after approximately 70% of 227 expected PFS events. RESULTS A total of 354 patients were randomly assigned (mobocertinib: n = 179; chemotherapy: n = 175). Baseline characteristics were balanced between arms. At IA (cutoff: April 4, 2023), the median PFS per BICR was 9.6 months in each treatment arm (hazard ratio [HR], 1.04 [95% CI, 0.77 to 1.39]; P = .803). The primary end point crossed the prespecified futility boundary (HR > 1). The confirmed objective response rate (95% CI) per BICR was 32% (26 to 40) with mobocertinib versus 30% (24 to 38) with chemotherapy; the median duration of response was 12.0 versus 8.4 months. Quality-of-life assessments indicated clinically meaningful delays in time to deterioration of lung cancer symptoms, cognitive function, and constipation with mobocertinib versus chemotherapy. Grade >= 3 adverse events in >5% of patients (mobocertinib, chemotherapy) were diarrhea (20%, 1%), anemia (6%, 10%), increased lipase (6%, 0%), and decreased neutrophil count (1%, 7%). CONCLUSION The EXCLAIM-2 trial did not meet its primary end point. The efficacy of mobocertinib was not superior to platinum-based chemotherapy for first-line treatment of patients with EGFR ex20ins+ advanced/metastatic NSCLC. | The EXCLAIM-2 trial compared mobocertinib, a daily pill targeting specific EGFR mutations, to platinum-based chemotherapy as the first treatment for patients with advanced non-small cell lung cancer with EGFR exon 20 insertions. Involving 354 patients, the trial found no significant difference in progression-free survival (9.6 months for both treatments) and mobocertinib did not outperform chemotherapy, leading to the trial failing its main goal. Both treatments had similar response rates, but mobocertinib showed longer-lasting responses and better quality-of-life outcomes, with fewer severe side effects like low neutrophil counts, though it caused more diarrhea. | The EXCLAIM-2 phase III trial compared mobocertinib, a targeted oral drug, to platinum-based chemotherapy as the first treatment for patients with advanced non-small cell lung cancer (NSCLC) with EGFR exon 20 insertion mutations. The trial involved 354 patients split evenly between the two treatments. Both treatments showed similar progression-free survival (9.6 months) and response rates (32% for mobocertinib vs. 30% for chemotherapy), but mobocertinib did not outperform chemotherapy, failing to meet the trial’s main goal. Mobocertinib offered longer-lasting responses and better quality of life in some areas, like lung cancer symptoms and cognitive function, but had more frequent severe diarrhea, while chemotherapy caused more anemia and low neutrophil counts. | The EXCLAIM-2 trial compared mobocertinib, a targeted oral drug, to platinum-based chemotherapy as the first treatment for patients with advanced non-small cell lung cancer (NSCLC) with EGFR exon 20 insertion mutations. In the study, 354 patients were split evenly between the two treatments. Both treatments showed similar progression-free survival (9.6 months) and response rates (32% for mobocertinib vs. 30% for chemotherapy), but mobocertinib did not perform better than chemotherapy, failing to meet the trial’s main goal. Mobocertinib offered longer-lasting responses and better quality-of-life outcomes, like delayed worsening of lung cancer symptoms, but had more frequent severe diarrhea, while chemotherapy caused more anemia and low neutrophil counts. |
| JCO | 3 | 10.1200/JCO-24-01442 | Open AI | PURPOSETo evaluate the cost utility of a 9-month supervised exercise program for patients with metastatic breast cancer (mBC), compared with control (usual care, supplemented with general activity advice and an activity tracker). Evidence on the cost-effectiveness of exercise for patients with mBC is essential for implementation in clinical practice and is currently lacking.METHODSA cost-utility analysis was performed alongside the multinational PREFERABLE-EFFECT randomized controlled trial, conducted in 8 centers across Europe and Australia. Patients with mBC (N = 357) were randomly assigned to either a 9-month, twice-weekly, supervised exercise group (EG) or control group (CG). Costs of the exercise program were calculated through a bottom-up approach. Other health care resource use, productivity losses, and quality of life were collected using country-adapted, self-reported questionnaires. Analyses were conducted from a societal perspective with a time horizon of 9 months. Costs were collected and reported in 2021 Euros (1 = $1.18 US dollars).RESULTSCompared with the CG, EG resulted in a quality-adjusted life-year (QALY) gain of 0.013 (95% CI, -0.02 to 0.05) over a 9-month period. The mean costs of the exercise program were 1,696 per patient with one-on-one supervision (scenario 1) and 609 with one-on-four supervision (scenario 2). These costs were offset by savings in health care and productivity costs, resulting in mean total cost differences of -163 (scenario 1) and -1,249 (scenario 2) in favor of EG. The probability of supervised exercise being cost-effective was 65% in scenario 1 and 91% in scenario 2 at a willingness-to-pay threshold of 20,000 per QALY.CONCLUSIONExercise for patients with mBC increases quality of life, decreases costs, and is likely to be cost-effective. Group-based supervision is expected to have even higher cost-savings. Our positive findings can inform reimbursement of supervised exercise interventions for patients with mBC. | A study of 357 patients with metastatic breast cancer found that a 9-month, twice-weekly supervised exercise program improved quality of life and could save money compared to usual care. One-on-one sessions cost about €1,696 per patient, while group sessions cost about €609, with group supervision leading to bigger savings. Overall, exercise reduced healthcare and productivity costs and had a high chance of being cost-effective, especially in group settings, supporting its inclusion in standard care. | A 9-month supervised exercise program for people with metastatic breast cancer was tested against usual care in a large international study. The program slightly improved quality of life and, depending on how it was delivered, either reduced overall costs or nearly broke even. Group-based sessions (one trainer for four patients) were especially cost-saving. Overall, exercise was found to be likely cost-effective, supporting its use and potential reimbursement in cancer care. | A 9-month supervised exercise program for people with metastatic breast cancer was tested against usual care in a large international study. The program improved quality of life and, depending on whether sessions were one-on-one or in small groups, either slightly reduced or significantly lowered overall costs by saving on healthcare and productivity expenses. Group-based exercise was especially cost-effective, making it a promising option for improving patient well-being while reducing costs. |
| JCO | 3 | 10.1200/JCO-24-01443 | Claude | PURPOSETo evaluate the cost utility of a 9-month supervised exercise program for patients with metastatic breast cancer (mBC), compared with control (usual care, supplemented with general activity advice and an activity tracker). Evidence on the cost-effectiveness of exercise for patients with mBC is essential for implementation in clinical practice and is currently lacking.METHODSA cost-utility analysis was performed alongside the multinational PREFERABLE-EFFECT randomized controlled trial, conducted in 8 centers across Europe and Australia. Patients with mBC (N = 357) were randomly assigned to either a 9-month, twice-weekly, supervised exercise group (EG) or control group (CG). Costs of the exercise program were calculated through a bottom-up approach. Other health care resource use, productivity losses, and quality of life were collected using country-adapted, self-reported questionnaires. Analyses were conducted from a societal perspective with a time horizon of 9 months. Costs were collected and reported in 2021 Euros (1 = $1.18 US dollars).RESULTSCompared with the CG, EG resulted in a quality-adjusted life-year (QALY) gain of 0.013 (95% CI, -0.02 to 0.05) over a 9-month period. The mean costs of the exercise program were 1,696 per patient with one-on-one supervision (scenario 1) and 609 with one-on-four supervision (scenario 2). These costs were offset by savings in health care and productivity costs, resulting in mean total cost differences of -163 (scenario 1) and -1,249 (scenario 2) in favor of EG. The probability of supervised exercise being cost-effective was 65% in scenario 1 and 91% in scenario 2 at a willingness-to-pay threshold of 20,000 per QALY.CONCLUSIONExercise for patients with mBC increases quality of life, decreases costs, and is likely to be cost-effective. Group-based supervision is expected to have even higher cost-savings. Our positive findings can inform reimbursement of supervised exercise interventions for patients with mBC. | This study looked at whether a 9-month supervised exercise program for women with advanced breast cancer that has spread to other parts of the body is worth the cost. Researchers compared 357 patients who did the exercise program (working out twice a week with supervision) to those who received regular care plus basic activity advice. They found that the exercise program slightly improved quality of life and actually saved money overall - even though the program itself cost between €609-€1,696 per patient, it reduced other healthcare costs and work-related losses enough to more than make up for it. The program was likely cost-effective, especially when supervised in small groups rather than one-on-one, suggesting that insurance should consider covering these exercise programs for patients with advanced breast cancer. | This study looked at whether supervised exercise programs are worth the cost for patients with advanced breast cancer that has spread to other parts of the body. Researchers followed 357 patients for 9 months, comparing those who did a twice-weekly supervised exercise program to those who received usual care with basic activity advice. They found that the exercise program slightly improved patients' quality of life and actually saved money overall - even though the program itself cost between €609-€1,696 per patient, it reduced other healthcare costs and work productivity losses enough to more than make up for this expense. The study concluded that supervised exercise programs for advanced breast cancer patients are likely cost-effective and could justify insurance coverage, especially when done in small groups rather than one-on-one sessions. | Researchers studied whether supervised exercise programs are cost-effective for patients with advanced breast cancer that has spread to other parts of the body. They compared 357 patients who did a 9-month, twice-weekly exercise program with those who received usual care plus general activity advice. The exercise group had slightly better quality of life and actually saved money overall - between €163 to €1,249 per patient - because the cost of the exercise program was more than offset by reduced healthcare costs and fewer missed work days. The study found that supervised exercise is likely cost-effective, especially when done in small groups rather than one-on-one sessions, suggesting that insurance companies and healthcare systems should consider covering these programs for patients with advanced breast cancer. |
| JCO | 3 | 10.1200/JCO-24-01444 | Gemini | PURPOSETo evaluate the cost utility of a 9-month supervised exercise program for patients with metastatic breast cancer (mBC), compared with control (usual care, supplemented with general activity advice and an activity tracker). Evidence on the cost-effectiveness of exercise for patients with mBC is essential for implementation in clinical practice and is currently lacking.METHODSA cost-utility analysis was performed alongside the multinational PREFERABLE-EFFECT randomized controlled trial, conducted in 8 centers across Europe and Australia. Patients with mBC (N = 357) were randomly assigned to either a 9-month, twice-weekly, supervised exercise group (EG) or control group (CG). Costs of the exercise program were calculated through a bottom-up approach. Other health care resource use, productivity losses, and quality of life were collected using country-adapted, self-reported questionnaires. Analyses were conducted from a societal perspective with a time horizon of 9 months. Costs were collected and reported in 2021 Euros (1 = $1.18 US dollars).RESULTSCompared with the CG, EG resulted in a quality-adjusted life-year (QALY) gain of 0.013 (95% CI, -0.02 to 0.05) over a 9-month period. The mean costs of the exercise program were 1,696 per patient with one-on-one supervision (scenario 1) and 609 with one-on-four supervision (scenario 2). These costs were offset by savings in health care and productivity costs, resulting in mean total cost differences of -163 (scenario 1) and -1,249 (scenario 2) in favor of EG. The probability of supervised exercise being cost-effective was 65% in scenario 1 and 91% in scenario 2 at a willingness-to-pay threshold of 20,000 per QALY.CONCLUSIONExercise for patients with mBC increases quality of life, decreases costs, and is likely to be cost-effective. Group-based supervision is expected to have even higher cost-savings. Our positive findings can inform reimbursement of supervised exercise interventions for patients with mBC. | A study looked at the costs and benefits of a nine-month supervised exercise program for patients with metastatic breast cancer. Researchers compared patients who exercised with those who received usual care and found that the exercise group had a slightly better quality of life and lower overall costs. The savings came from reduced healthcare visits and fewer productivity losses. The study found that group exercise was even more cost-effective than one-on-one sessions. These results suggest that exercise for patients with metastatic breast cancer is a smart investment that can improve quality of life while reducing costs. | A recent study looked at whether a supervised exercise program is a good value for patients with metastatic breast cancer. The researchers compared a group that did a 9-month exercise program to a control group that received standard care. The results showed that the exercise program slightly improved the patients' quality of life and actually reduced overall costs because it led to fewer medical visits and lost workdays. When the exercise was done in a group setting, the cost savings were even greater. The study concluded that supervised exercise for these patients is likely to be a cost-effective and beneficial option that could be considered for insurance reimbursement. | A recent study looked at whether a supervised exercise program is a good value for patients with metastatic breast cancer. The research compared a nine-month exercise program to standard care. The findings showed that the exercise group had a slightly better quality of life and also saved money, mainly because they had fewer healthcare costs and missed less work. When the exercise was done in a group setting instead of one-on-one, the cost savings were even greater. Overall, the study concluded that supervised exercise is a worthwhile and cost-effective option that could be widely implemented for patients with metastatic breast cancer. |
| JCO | 3 | 10.1200/JCO-24-01445 | DeepSeek | PURPOSETo evaluate the cost utility of a 9-month supervised exercise program for patients with metastatic breast cancer (mBC), compared with control (usual care, supplemented with general activity advice and an activity tracker). Evidence on the cost-effectiveness of exercise for patients with mBC is essential for implementation in clinical practice and is currently lacking.METHODSA cost-utility analysis was performed alongside the multinational PREFERABLE-EFFECT randomized controlled trial, conducted in 8 centers across Europe and Australia. Patients with mBC (N = 357) were randomly assigned to either a 9-month, twice-weekly, supervised exercise group (EG) or control group (CG). Costs of the exercise program were calculated through a bottom-up approach. Other health care resource use, productivity losses, and quality of life were collected using country-adapted, self-reported questionnaires. Analyses were conducted from a societal perspective with a time horizon of 9 months. Costs were collected and reported in 2021 Euros (1 = $1.18 US dollars).RESULTSCompared with the CG, EG resulted in a quality-adjusted life-year (QALY) gain of 0.013 (95% CI, -0.02 to 0.05) over a 9-month period. The mean costs of the exercise program were 1,696 per patient with one-on-one supervision (scenario 1) and 609 with one-on-four supervision (scenario 2). These costs were offset by savings in health care and productivity costs, resulting in mean total cost differences of -163 (scenario 1) and -1,249 (scenario 2) in favor of EG. The probability of supervised exercise being cost-effective was 65% in scenario 1 and 91% in scenario 2 at a willingness-to-pay threshold of 20,000 per QALY.CONCLUSIONExercise for patients with mBC increases quality of life, decreases costs, and is likely to be cost-effective. Group-based supervision is expected to have even higher cost-savings. Our positive findings can inform reimbursement of supervised exercise interventions for patients with mBC. | This study looked at whether a 9-month supervised exercise program for patients with metastatic breast cancer (mBC) is worth the cost compared to usual care. The program improved quality of life slightly (0.013 QALYs) and saved money overall, especially when exercise sessions were done in groups (€1,249 savings per patient). Individual sessions also saved costs (€163) and were likely cost-effective (65-91% chance, depending on setup). The findings support covering supervised exercise for mBC patients, as it boosts well-being and reduces healthcare expenses. | This study looked at whether a 9-month supervised exercise program for patients with metastatic breast cancer (mBC) is cost-effective compared to usual care. The program improved quality of life slightly (0.013 QALYs) and reduced overall costs, especially when exercise was done in groups (saving €1,249 per patient). The chances of it being cost-effective were 65% with one-on-one supervision and 91% with group supervision. The findings suggest that supervised exercise is beneficial, affordable, and should be considered for coverage in health care. | This study looked at whether a 9-month supervised exercise program for patients with metastatic breast cancer (mBC) is worth the cost compared to usual care. The program improved quality of life slightly (0.013 QALYs) and saved money overall—€163 to €1,249 per patient—especially when exercise was done in small groups (1-on-4) instead of one-on-one. There was a 65-91% chance the program was cost-effective, making it a good option for patients. The findings support insurance coverage for supervised exercise in mBC care. |
| JCO | 3 | 10.1200/JCO-24-01446 | Grok | PURPOSETo evaluate the cost utility of a 9-month supervised exercise program for patients with metastatic breast cancer (mBC), compared with control (usual care, supplemented with general activity advice and an activity tracker). Evidence on the cost-effectiveness of exercise for patients with mBC is essential for implementation in clinical practice and is currently lacking.METHODSA cost-utility analysis was performed alongside the multinational PREFERABLE-EFFECT randomized controlled trial, conducted in 8 centers across Europe and Australia. Patients with mBC (N = 357) were randomly assigned to either a 9-month, twice-weekly, supervised exercise group (EG) or control group (CG). Costs of the exercise program were calculated through a bottom-up approach. Other health care resource use, productivity losses, and quality of life were collected using country-adapted, self-reported questionnaires. Analyses were conducted from a societal perspective with a time horizon of 9 months. Costs were collected and reported in 2021 Euros (1 = $1.18 US dollars).RESULTSCompared with the CG, EG resulted in a quality-adjusted life-year (QALY) gain of 0.013 (95% CI, -0.02 to 0.05) over a 9-month period. The mean costs of the exercise program were 1,696 per patient with one-on-one supervision (scenario 1) and 609 with one-on-four supervision (scenario 2). These costs were offset by savings in health care and productivity costs, resulting in mean total cost differences of -163 (scenario 1) and -1,249 (scenario 2) in favor of EG. The probability of supervised exercise being cost-effective was 65% in scenario 1 and 91% in scenario 2 at a willingness-to-pay threshold of 20,000 per QALY.CONCLUSIONExercise for patients with mBC increases quality of life, decreases costs, and is likely to be cost-effective. Group-based supervision is expected to have even higher cost-savings. Our positive findings can inform reimbursement of supervised exercise interventions for patients with mBC. | A study called PREFERABLE-EFFECT looked at whether a 9-month supervised exercise program for patients with metastatic breast cancer was worth the cost compared to usual care with general activity advice. The exercise program slightly improved quality of life and cost less overall, saving money on healthcare and work-related losses. When supervised one-on-one, the program cost €1,696 per patient, but in a group setting, it was only €609. The group-based program had a 91% chance of being cost-effective, making it a promising option for improving patient outcomes while reducing costs. | A study looked at whether a 9-month supervised exercise program for patients with metastatic breast cancer was worth the cost compared to usual care with general activity advice and a tracker. The exercise program slightly improved quality of life and cost less overall, mainly because it reduced healthcare and productivity losses. With one-on-one supervision, the program cost €1,696 per patient, but with group supervision (one trainer for four patients), it was only €609. The group-based option saved more money and had a 91% chance of being cost-effective, suggesting exercise programs could be a valuable, money-saving option for these patients. | A study called PREFERABLE-EFFECT looked at whether a 9-month supervised exercise program for patients with metastatic breast cancer is worth the cost compared to usual care with general activity advice and an activity tracker. The exercise program slightly improved quality of life and saved money overall, with costs of €1,696 per patient for one-on-one supervision and €609 for group supervision. These costs were offset by lower health care and productivity losses, making the program likely cost-effective, especially with group supervision, which had a 91% chance of being cost-effective at a €20,000 threshold per quality-adjusted life-year. |
| JCO | 4 | 10.1200/JCO-24-02087 | Open AI | PURPOSE Cyclin-dependent kinase 4/6 inhibitors (CDK4/6i) combined with endocrine therapy (ET) are the standard first-line treatment for hormone receptor-positive (HR+), human epidermal growth factor receptor 2-negative (HER2-) advanced breast cancer (ABC); however, disease progression occurs in almost all patients and additional treatment options are needed. Herein, we report outcomes of the postMONARCH trial investigating a switch in ET with/without CDK4/6 inhibition with abemaciclib after disease progression on CDK4/6i. METHODS This double-blind, randomized phase III study enrolled patients with disease progression on previous CDK4/6i plus aromatase inhibitor as initial therapy for advanced disease or recurrence on/after adjuvant CDK4/6i + ET. Patients were randomly assigned (1:1) to abemaciclib + fulvestrant or placebo + fulvestrant. The primary end point was investigator-assessed progression-free survival (PFS). Secondary end points included PFS by blinded independent central review, objective response rate (ORR), and safety. RESULTS This study randomly assigned 368 patients (abemaciclib + fulvestrant, n = 182 placebo + fulvestrant, n = 186). At the primary analysis (258 events), the hazard ratio (HR) was 0.73 (95% CI, 0.57 to 0.95; nominal P = .017), with median PFS 6.0 (95% CI, 5.6 to 8.6) versus 5.3 (95% CI, 3.7 to 5.6) months and 6-month PFS rates of 50% and 37% in the abemaciclib + fulvestrant and placebo + fulvestrant arms, respectively. These results were supported by BICR-assessed PFS (HR, 0.55 [95% CI, 0.39 to 0.77]; nominal P < .001). A consistent treatment effect was seen across major clinical and genomic subgroups, including with/without ESR1 or PIK3CA mutations. Among patients with measurable disease, investigator-assessed ORR was improved with abemaciclib + fulvestrant versus placebo + fulvestrant (17% v 7%; nominal P = .015). No new safety signals were observed, with findings consistent with the known safety profile of abemaciclib. CONCLUSION Abemaciclib + fulvestrant significantly improved PFS after disease progression on previous CDK4/6i + ET in patients with HR+, HER2- ABC, offering an additional targeted therapy option for these patients. | The postMONARCH trial studied patients with advanced HR+, HER2- breast cancer whose disease worsened after initial CDK4/6 inhibitor plus endocrine therapy. It compared switching to fulvestrant with or without adding the CDK4/6 inhibitor abemaciclib. Results showed that adding abemaciclib significantly delayed cancer progression (median 6.0 vs 5.3 months) and improved response rates, with benefits seen across different patient subgroups. Side effects were consistent with known safety data, suggesting this combination is a useful treatment option after prior CDK4/6 therapy fails. | A study tested adding the drug abemaciclib to fulvestrant in patients with advanced hormone-positive, HER2-negative breast cancer who had stopped responding to previous similar treatments. The combination helped slow cancer growth longer than fulvestrant alone, with more patients benefiting and no new safety problems. This shows abemaciclib plus fulvestrant can be a useful treatment option after the cancer progresses on earlier therapies. | This study tested a new treatment for advanced breast cancer patients whose disease got worse after standard therapy. It found that combining the drug abemaciclib with fulvestrant helped patients live longer without their cancer growing compared to fulvestrant alone. The treatment worked well in different patient groups and was safe, offering a new option for people with this type of breast cancer after their first treatment stops working. |
| JCO | 4 | 10.1200/JCO-24-02088 | Claude | PURPOSE Cyclin-dependent kinase 4/6 inhibitors (CDK4/6i) combined with endocrine therapy (ET) are the standard first-line treatment for hormone receptor-positive (HR+), human epidermal growth factor receptor 2-negative (HER2-) advanced breast cancer (ABC); however, disease progression occurs in almost all patients and additional treatment options are needed. Herein, we report outcomes of the postMONARCH trial investigating a switch in ET with/without CDK4/6 inhibition with abemaciclib after disease progression on CDK4/6i. METHODS This double-blind, randomized phase III study enrolled patients with disease progression on previous CDK4/6i plus aromatase inhibitor as initial therapy for advanced disease or recurrence on/after adjuvant CDK4/6i + ET. Patients were randomly assigned (1:1) to abemaciclib + fulvestrant or placebo + fulvestrant. The primary end point was investigator-assessed progression-free survival (PFS). Secondary end points included PFS by blinded independent central review, objective response rate (ORR), and safety. RESULTS This study randomly assigned 368 patients (abemaciclib + fulvestrant, n = 182 placebo + fulvestrant, n = 186). At the primary analysis (258 events), the hazard ratio (HR) was 0.73 (95% CI, 0.57 to 0.95; nominal P = .017), with median PFS 6.0 (95% CI, 5.6 to 8.6) versus 5.3 (95% CI, 3.7 to 5.6) months and 6-month PFS rates of 50% and 37% in the abemaciclib + fulvestrant and placebo + fulvestrant arms, respectively. These results were supported by BICR-assessed PFS (HR, 0.55 [95% CI, 0.39 to 0.77]; nominal P < .001). A consistent treatment effect was seen across major clinical and genomic subgroups, including with/without ESR1 or PIK3CA mutations. Among patients with measurable disease, investigator-assessed ORR was improved with abemaciclib + fulvestrant versus placebo + fulvestrant (17% v 7%; nominal P = .015). No new safety signals were observed, with findings consistent with the known safety profile of abemaciclib. CONCLUSION Abemaciclib + fulvestrant significantly improved PFS after disease progression on previous CDK4/6i + ET in patients with HR+, HER2- ABC, offering an additional targeted therapy option for these patients. | This study tested a new treatment combination for patients with advanced breast cancer whose disease had gotten worse despite standard therapy. Researchers gave 368 patients either abemaciclib plus fulvestrant or a placebo plus fulvestrant after their cancer had progressed on previous treatments. The results showed that patients who received the abemaciclib combination lived longer without their cancer getting worse (6 months versus 5.3 months) and had better response rates compared to those who got the placebo. The treatment was generally safe with no unexpected side effects, suggesting that abemaciclib plus fulvestrant could be a valuable new option for patients with this type of breast cancer when their initial treatments stop working. | This study tested a new treatment approach for patients with advanced breast cancer whose disease got worse despite taking standard drugs called CDK4/6 inhibitors. Researchers gave 368 patients either a combination of two drugs (abemaciclib + fulvestrant) or a placebo with fulvestrant to see which worked better. The results showed that patients who received the abemaciclib combination lived longer without their cancer progressing (6.0 months vs 5.3 months) and were more likely to see their tumors shrink. The treatment was generally safe with no unexpected side effects, giving doctors a new option for patients whose cancer stops responding to the usual first treatments. | This study tested a new treatment approach for patients with advanced breast cancer whose disease got worse despite taking standard medications (CDK4/6 inhibitors plus hormone therapy). Researchers gave 368 patients either a combination of two drugs (abemaciclib + fulvestrant) or a placebo plus one drug (fulvestrant). The results showed that patients who received both active drugs lived longer without their cancer progressing - about 6 months compared to 5.3 months for those on placebo. The combination treatment was safe and didn't cause unexpected side effects, suggesting it could be a useful new option for breast cancer patients when their current treatment stops working. |
| JCO | 4 | 10.1200/JCO-24-02089 | Gemini | PURPOSE Cyclin-dependent kinase 4/6 inhibitors (CDK4/6i) combined with endocrine therapy (ET) are the standard first-line treatment for hormone receptor-positive (HR+), human epidermal growth factor receptor 2-negative (HER2-) advanced breast cancer (ABC); however, disease progression occurs in almost all patients and additional treatment options are needed. Herein, we report outcomes of the postMONARCH trial investigating a switch in ET with/without CDK4/6 inhibition with abemaciclib after disease progression on CDK4/6i. METHODS This double-blind, randomized phase III study enrolled patients with disease progression on previous CDK4/6i plus aromatase inhibitor as initial therapy for advanced disease or recurrence on/after adjuvant CDK4/6i + ET. Patients were randomly assigned (1:1) to abemaciclib + fulvestrant or placebo + fulvestrant. The primary end point was investigator-assessed progression-free survival (PFS). Secondary end points included PFS by blinded independent central review, objective response rate (ORR), and safety. RESULTS This study randomly assigned 368 patients (abemaciclib + fulvestrant, n = 182 placebo + fulvestrant, n = 186). At the primary analysis (258 events), the hazard ratio (HR) was 0.73 (95% CI, 0.57 to 0.95; nominal P = .017), with median PFS 6.0 (95% CI, 5.6 to 8.6) versus 5.3 (95% CI, 3.7 to 5.6) months and 6-month PFS rates of 50% and 37% in the abemaciclib + fulvestrant and placebo + fulvestrant arms, respectively. These results were supported by BICR-assessed PFS (HR, 0.55 [95% CI, 0.39 to 0.77]; nominal P < .001). A consistent treatment effect was seen across major clinical and genomic subgroups, including with/without ESR1 or PIK3CA mutations. Among patients with measurable disease, investigator-assessed ORR was improved with abemaciclib + fulvestrant versus placebo + fulvestrant (17% v 7%; nominal P = .015). No new safety signals were observed, with findings consistent with the known safety profile of abemaciclib. CONCLUSION Abemaciclib + fulvestrant significantly improved PFS after disease progression on previous CDK4/6i + ET in patients with HR+, HER2- ABC, offering an additional targeted therapy option for these patients. | After initial treatment for advanced breast cancer with a CDK4/6 inhibitor and hormone therapy, the postMONARCH trial found that switching to a new treatment combination of abemaciclib and fulvestrant can slow cancer progression. In this study, patients who were given this new combination lived longer without their cancer getting worse compared to those who only received fulvestrant. The new treatment also helped shrink tumors in more patients. The safety risks were consistent with what is already known about abemaciclib. This new combination offers a potential additional treatment option for patients whose cancer has progressed after initial therapy. | After initial treatment for advanced breast cancer with a CDK4/6 inhibitor and hormone therapy, the postMONARCH trial found that a new treatment combination of abemaciclib and fulvestrant significantly slowed down cancer growth. In the study, patients who received this combination had a median progression-free survival of 6.0 months, compared to 5.3 months for those who received fulvestrant alone. The study also found that the new combination was generally safe, with side effects that were consistent with what is already known about abemaciclib. The results suggest that this treatment combination could be a new option for patients whose cancer has progressed after initial treatment. | Abemaciclib (Verzenio) and fulvestrant (Faslodex) were tested in the postMONARCH trial for patients with advanced breast cancer that had progressed after initial treatment with a different CDK4/6 inhibitor and endocrine therapy. The study found that patients who received abemaciclib plus fulvestrant lived longer without their cancer getting worse compared to those who received a placebo and fulvestrant. The combination of abemaciclib and fulvestrant also led to a higher rate of tumor shrinkage. These results suggest that this combination is an effective new treatment option for patients whose cancer has progressed after initial CDK4/6 inhibitor therapy. |
| JCO | 4 | 10.1200/JCO-24-02090 | DeepSeek | PURPOSE Cyclin-dependent kinase 4/6 inhibitors (CDK4/6i) combined with endocrine therapy (ET) are the standard first-line treatment for hormone receptor-positive (HR+), human epidermal growth factor receptor 2-negative (HER2-) advanced breast cancer (ABC); however, disease progression occurs in almost all patients and additional treatment options are needed. Herein, we report outcomes of the postMONARCH trial investigating a switch in ET with/without CDK4/6 inhibition with abemaciclib after disease progression on CDK4/6i. METHODS This double-blind, randomized phase III study enrolled patients with disease progression on previous CDK4/6i plus aromatase inhibitor as initial therapy for advanced disease or recurrence on/after adjuvant CDK4/6i + ET. Patients were randomly assigned (1:1) to abemaciclib + fulvestrant or placebo + fulvestrant. The primary end point was investigator-assessed progression-free survival (PFS). Secondary end points included PFS by blinded independent central review, objective response rate (ORR), and safety. RESULTS This study randomly assigned 368 patients (abemaciclib + fulvestrant, n = 182 placebo + fulvestrant, n = 186). At the primary analysis (258 events), the hazard ratio (HR) was 0.73 (95% CI, 0.57 to 0.95; nominal P = .017), with median PFS 6.0 (95% CI, 5.6 to 8.6) versus 5.3 (95% CI, 3.7 to 5.6) months and 6-month PFS rates of 50% and 37% in the abemaciclib + fulvestrant and placebo + fulvestrant arms, respectively. These results were supported by BICR-assessed PFS (HR, 0.55 [95% CI, 0.39 to 0.77]; nominal P < .001). A consistent treatment effect was seen across major clinical and genomic subgroups, including with/without ESR1 or PIK3CA mutations. Among patients with measurable disease, investigator-assessed ORR was improved with abemaciclib + fulvestrant versus placebo + fulvestrant (17% v 7%; nominal P = .015). No new safety signals were observed, with findings consistent with the known safety profile of abemaciclib. CONCLUSION Abemaciclib + fulvestrant significantly improved PFS after disease progression on previous CDK4/6i + ET in patients with HR+, HER2- ABC, offering an additional targeted therapy option for these patients. | The postMONARCH trial tested whether adding abemaciclib (a CDK4/6 inhibitor) to fulvestrant (hormone therapy) helps advanced breast cancer patients whose disease worsened after initial treatment with a similar drug. The study found that patients taking abemaciclib plus fulvestrant lived longer without their cancer growing (6 months vs. 5.3 months) compared to those on fulvestrant alone, with fewer side effects. This combo could be a new option for patients who progress on earlier CDK4/6 inhibitor therapy. | The postMONARCH trial tested whether adding abemaciclib (a CDK4/6 inhibitor) to fulvestrant (hormone therapy) helps patients with advanced HR+, HER2- breast cancer that had worsened after prior CDK4/6 inhibitor treatment. The study found that patients receiving abemaciclib plus fulvestrant lived longer without their cancer progressing (6.0 months) compared to those on fulvestrant alone (5.3 months), with a 17% response rate versus 7%. The treatment was safe, with no new side effects. This shows abemaciclib plus fulvestrant is a useful option after earlier CDK4/6 inhibitor treatment fails. | The postMONARCH trial tested whether adding abemaciclib (a CDK4/6 inhibitor) to fulvestrant (hormone therapy) could help patients with advanced HR+, HER2- breast cancer that had worsened after previous treatment with a different CDK4/6 inhibitor. The study found that patients receiving abemaciclib plus fulvestrant lived longer without their cancer growing (6 months vs. 5.3 months) compared to those on fulvestrant alone, with manageable side effects. This combination provides a new treatment option for patients whose cancer progresses after initial CDK4/6 inhibitor therapy. |
| JCO | 4 | 10.1200/JCO-24-02091 | Grok | PURPOSE Cyclin-dependent kinase 4/6 inhibitors (CDK4/6i) combined with endocrine therapy (ET) are the standard first-line treatment for hormone receptor-positive (HR+), human epidermal growth factor receptor 2-negative (HER2-) advanced breast cancer (ABC); however, disease progression occurs in almost all patients and additional treatment options are needed. Herein, we report outcomes of the postMONARCH trial investigating a switch in ET with/without CDK4/6 inhibition with abemaciclib after disease progression on CDK4/6i. METHODS This double-blind, randomized phase III study enrolled patients with disease progression on previous CDK4/6i plus aromatase inhibitor as initial therapy for advanced disease or recurrence on/after adjuvant CDK4/6i + ET. Patients were randomly assigned (1:1) to abemaciclib + fulvestrant or placebo + fulvestrant. The primary end point was investigator-assessed progression-free survival (PFS). Secondary end points included PFS by blinded independent central review, objective response rate (ORR), and safety. RESULTS This study randomly assigned 368 patients (abemaciclib + fulvestrant, n = 182 placebo + fulvestrant, n = 186). At the primary analysis (258 events), the hazard ratio (HR) was 0.73 (95% CI, 0.57 to 0.95; nominal P = .017), with median PFS 6.0 (95% CI, 5.6 to 8.6) versus 5.3 (95% CI, 3.7 to 5.6) months and 6-month PFS rates of 50% and 37% in the abemaciclib + fulvestrant and placebo + fulvestrant arms, respectively. These results were supported by BICR-assessed PFS (HR, 0.55 [95% CI, 0.39 to 0.77]; nominal P < .001). A consistent treatment effect was seen across major clinical and genomic subgroups, including with/without ESR1 or PIK3CA mutations. Among patients with measurable disease, investigator-assessed ORR was improved with abemaciclib + fulvestrant versus placebo + fulvestrant (17% v 7%; nominal P = .015). No new safety signals were observed, with findings consistent with the known safety profile of abemaciclib. CONCLUSION Abemaciclib + fulvestrant significantly improved PFS after disease progression on previous CDK4/6i + ET in patients with HR+, HER2- ABC, offering an additional targeted therapy option for these patients. | The postMONARCH trial studied whether switching to abemaciclib plus fulvestrant could help patients with advanced hormone receptor-positive, HER2-negative breast cancer whose disease worsened after initial treatment with a CDK4/6 inhibitor and endocrine therapy. In this study, 368 patients were split into two groups: one received abemaciclib with fulvestrant, and the other got a placebo with fulvestrant. The abemaciclib group had a longer time before their cancer progressed (6 months vs. 5.3 months), with 50% of patients progression-free at 6 months compared to 37% in the placebo group. The treatment also improved tumor response rates and showed consistent benefits across different patient groups, with no new safety concerns. This suggests abemaciclib plus fulvestrant is a useful option for these patients. | The postMONARCH trial studied whether switching to abemaciclib plus fulvestrant could help patients with hormone receptor-positive, HER2-negative advanced breast cancer who had disease progression after initial treatment with a CDK4/6 inhibitor and endocrine therapy. In this study, 368 patients were randomly assigned to receive either abemaciclib plus fulvestrant or a placebo plus fulvestrant. The abemaciclib group had a longer progression-free survival (6.0 months vs. 5.3 months) and a higher response rate (17% vs. 7%) compared to the placebo group, with consistent benefits across different patient subgroups. The treatment was safe, with no new side effects, making abemaciclib plus fulvestrant a promising option for these patients. | The postMONARCH trial studied whether switching to abemaciclib plus fulvestrant helps patients with hormone receptor-positive, HER2-negative advanced breast cancer who progressed after initial treatment with a CDK4/6 inhibitor and endocrine therapy. In this study, 368 patients were randomly assigned to receive either abemaciclib with fulvestrant or a placebo with fulvestrant. The abemaciclib group had a longer progression-free survival (6.0 months vs. 5.3 months) and a higher response rate (17% vs. 7%) compared to the placebo group, with consistent benefits across various patient subgroups. No new safety concerns were found, supporting abemaciclib plus fulvestrant as an effective treatment option for these patients. |
| JCO | 5 | 10.1200/JCO.24.00343 | Open AI | PURPOSE Guidelines recommend cardiovascular (CV) risk assessment and counseling for cancer survivors. This study evaluated the automated heart-health assessment (AH-HA) clinical decision support tool to promote provider-patient CV health (CVH) discussions in outpatient oncology. METHODS The AH-HA trial (WF-1804CD), coordinated by the Wake Forest National Cancer Institute Community Oncology Research Program Research Base, randomized practices to the AH-HA tool or usual care (UC) and enrolled survivors receiving routine care >= 6 months after curative cancer treatment. The tool displayed American Heart Association Life's Simple 7 CVH factors (BMI, physical activity, diet, smoking status, blood pressure, cholesterol, and glucose), populated from the electronic health record (EHR), alongside cancer treatments received with cardiotoxic potential. The primary end point was survivor-reported discussion of nonideal or missing CVH factors. A mixed-effects logistic regression model assessed the effect of AH-HA on CVH discussions, adjusting for practice. RESULTS Five UC and four AH-HA practices enrolled 645 survivors (82% breast, 8% endometrial, 5% colorectal, and 5% lymphoma, prostate, or multiple types) from October 1, 2020, to February 28, 2023. Most survivors were female (96%; 84% White/non-Hispanic, 8% Black; 3% Hispanic). Nearly all survivors (98%) in AH-HA practices reported a discussion for >= 1 nonideal or missing CVH factor compared with 55% in UC (P < .001). The average number of survivor-reported factors discussed was higher in AH-HA compared with UC (mean, 4.06 v 1.27; P < .001), as were EHR-documented discussions (3.83 v 0.77; P = .03). Survivors in AH-HA practices were also significantly more likely to report a recommendation to see a primary care provider (39%) compared with UC practices (25%, P = .02). Reported recommendations to see a cardiologist were low (approximately 6%) and did not differ between groups. CONCLUSION The AH-HA tool was effective at promoting CVH discussions during routine follow-up care for survivors and recommendations to consult primary care. | This study tested a new computer tool called AH-HA that helps doctors talk with cancer survivors about heart health risks. It showed that survivors whose doctors used the tool had more heart health discussions and were more often advised to see a primary care doctor compared to usual care. This means the tool helps improve important heart health conversations during cancer follow-up visits. | This study tested a tool called AH-HA that helps doctors talk with cancer survivors about heart health during regular check-ups. It showed important heart risk factors using patient records and cancer treatment history. Cancer survivors using this tool had more conversations about heart health with their doctors and were more often advised to see their primary care doctor than those getting usual care. The tool helped improve heart health discussions but didn’t change advice about seeing heart specialists. | This study tested a tool called AH-HA that helps doctors talk with cancer survivors about heart health risks during regular visits. The tool showed important heart health info from medical records and cancer treatments that could affect the heart. Compared to usual care, survivors using AH-HA had more heart health discussions and were more often advised to see a primary care doctor. This means the tool helps improve heart health conversations and follow-up care for cancer survivors. |
| JCO | 5 | 10.1200/JCO.24.00344 | Claude | PURPOSE Guidelines recommend cardiovascular (CV) risk assessment and counseling for cancer survivors. This study evaluated the automated heart-health assessment (AH-HA) clinical decision support tool to promote provider-patient CV health (CVH) discussions in outpatient oncology. METHODS The AH-HA trial (WF-1804CD), coordinated by the Wake Forest National Cancer Institute Community Oncology Research Program Research Base, randomized practices to the AH-HA tool or usual care (UC) and enrolled survivors receiving routine care >= 6 months after curative cancer treatment. The tool displayed American Heart Association Life's Simple 7 CVH factors (BMI, physical activity, diet, smoking status, blood pressure, cholesterol, and glucose), populated from the electronic health record (EHR), alongside cancer treatments received with cardiotoxic potential. The primary end point was survivor-reported discussion of nonideal or missing CVH factors. A mixed-effects logistic regression model assessed the effect of AH-HA on CVH discussions, adjusting for practice. RESULTS Five UC and four AH-HA practices enrolled 645 survivors (82% breast, 8% endometrial, 5% colorectal, and 5% lymphoma, prostate, or multiple types) from October 1, 2020, to February 28, 2023. Most survivors were female (96%; 84% White/non-Hispanic, 8% Black; 3% Hispanic). Nearly all survivors (98%) in AH-HA practices reported a discussion for >= 1 nonideal or missing CVH factor compared with 55% in UC (P < .001). The average number of survivor-reported factors discussed was higher in AH-HA compared with UC (mean, 4.06 v 1.27; P < .001), as were EHR-documented discussions (3.83 v 0.77; P = .03). Survivors in AH-HA practices were also significantly more likely to report a recommendation to see a primary care provider (39%) compared with UC practices (25%, P = .02). Reported recommendations to see a cardiologist were low (approximately 6%) and did not differ between groups. CONCLUSION The AH-HA tool was effective at promoting CVH discussions during routine follow-up care for survivors and recommendations to consult primary care. | This study tested a computer tool called AH-HA that helps doctors discuss heart health with cancer survivors during regular check-ups. The tool automatically pulls information from medical records about seven key heart health factors (like weight, exercise, diet, and blood pressure) and shows them alongside any cancer treatments that might affect the heart. Researchers compared cancer clinics using this tool to clinics providing usual care. They found that nearly all patients (98%) at clinics with the AH-HA tool discussed their heart health factors with their doctors, compared to only 55% at regular clinics. Patients using the tool also discussed more heart health topics overall and were more likely to be referred to their primary care doctor for follow-up. | This study tested a computer tool called AH-HA that helps doctors discuss heart health with cancer survivors during regular check-ups. The tool automatically pulls information from patient records about seven key heart health factors (like weight, exercise, diet, smoking, blood pressure, cholesterol, and blood sugar) and shows them alongside any cancer treatments that might affect the heart. Researchers compared cancer survivors whose doctors used this tool to those who received usual care. They found that nearly all patients (98%) whose doctors had the tool discussed at least one heart health issue, compared to only 55% of patients in usual care. The tool also led to more referrals to primary care doctors for heart health follow-up, showing it was effective at improving heart health conversations during cancer care. | This study tested a computer tool called AH-HA that helps doctors discuss heart health with cancer survivors during regular check-ups. The tool automatically pulls information from medical records about seven key heart health factors (like weight, exercise, diet, smoking, blood pressure, cholesterol, and blood sugar) and shows which cancer treatments might have affected the heart. Researchers compared cancer clinics that used this tool with those that didn't, involving 645 cancer survivors. They found that nearly all patients (98%) at clinics using the AH-HA tool had discussions about improving their heart health, compared to only 55% at regular clinics. The tool was successful at encouraging more heart health conversations and getting patients referred to their primary care doctors for follow-up care. |
| JCO | 5 | 10.1200/JCO.24.00345 | Gemini | PURPOSE Guidelines recommend cardiovascular (CV) risk assessment and counseling for cancer survivors. This study evaluated the automated heart-health assessment (AH-HA) clinical decision support tool to promote provider-patient CV health (CVH) discussions in outpatient oncology. METHODS The AH-HA trial (WF-1804CD), coordinated by the Wake Forest National Cancer Institute Community Oncology Research Program Research Base, randomized practices to the AH-HA tool or usual care (UC) and enrolled survivors receiving routine care >= 6 months after curative cancer treatment. The tool displayed American Heart Association Life's Simple 7 CVH factors (BMI, physical activity, diet, smoking status, blood pressure, cholesterol, and glucose), populated from the electronic health record (EHR), alongside cancer treatments received with cardiotoxic potential. The primary end point was survivor-reported discussion of nonideal or missing CVH factors. A mixed-effects logistic regression model assessed the effect of AH-HA on CVH discussions, adjusting for practice. RESULTS Five UC and four AH-HA practices enrolled 645 survivors (82% breast, 8% endometrial, 5% colorectal, and 5% lymphoma, prostate, or multiple types) from October 1, 2020, to February 28, 2023. Most survivors were female (96%; 84% White/non-Hispanic, 8% Black; 3% Hispanic). Nearly all survivors (98%) in AH-HA practices reported a discussion for >= 1 nonideal or missing CVH factor compared with 55% in UC (P < .001). The average number of survivor-reported factors discussed was higher in AH-HA compared with UC (mean, 4.06 v 1.27; P < .001), as were EHR-documented discussions (3.83 v 0.77; P = .03). Survivors in AH-HA practices were also significantly more likely to report a recommendation to see a primary care provider (39%) compared with UC practices (25%, P = .02). Reported recommendations to see a cardiologist were low (approximately 6%) and did not differ between groups. CONCLUSION The AH-HA tool was effective at promoting CVH discussions during routine follow-up care for survivors and recommendations to consult primary care. | A study looked at how well an electronic health record tool, called the AH-HA tool, helped doctors and cancer survivors talk about heart health. The tool automatically gathered information on seven key health factors, like BMI and blood pressure, for survivors in a community oncology setting. The study found that patients whose doctors used the AH-HA tool were much more likely to have a conversation about their heart health compared to those who received standard care. On average, the patients in the group using the tool discussed more health factors and were more likely to be told to see their primary care doctor. Overall, the AH-HA tool was very effective at promoting these important heart health discussions | The WF-1804CD study found that a new tool called AH-HA, which uses electronic health records to assess heart health, was very effective. The tool helped doctors and cancer survivors have more conversations about heart health, specifically about factors like BMI, diet, and physical activity. The study showed that nearly all survivors using the AH-HA tool had these discussions, compared to only 55% of those receiving standard care. The tool also led to more recommendations for survivors to see their primary care doctor. | The WF-1804CD study evaluated a tool called AH-HA, which is a clinical decision support tool in an electronic health record (EHR) designed to help doctors and patients talk about heart health. The study found that patients in practices using the AH-HA tool had significantly more discussions with their doctors about heart health factors like BMI and blood pressure compared to patients in practices that did not use the tool. Patients in the AH-HA group were also more likely to be recommended to see their primary care provider. These results suggest the AH-HA tool is effective at promoting heart health discussions for cancer survivors. |
| JCO | 5 | 10.1200/JCO.24.00346 | DeepSeek | PURPOSE Guidelines recommend cardiovascular (CV) risk assessment and counseling for cancer survivors. This study evaluated the automated heart-health assessment (AH-HA) clinical decision support tool to promote provider-patient CV health (CVH) discussions in outpatient oncology. METHODS The AH-HA trial (WF-1804CD), coordinated by the Wake Forest National Cancer Institute Community Oncology Research Program Research Base, randomized practices to the AH-HA tool or usual care (UC) and enrolled survivors receiving routine care >= 6 months after curative cancer treatment. The tool displayed American Heart Association Life's Simple 7 CVH factors (BMI, physical activity, diet, smoking status, blood pressure, cholesterol, and glucose), populated from the electronic health record (EHR), alongside cancer treatments received with cardiotoxic potential. The primary end point was survivor-reported discussion of nonideal or missing CVH factors. A mixed-effects logistic regression model assessed the effect of AH-HA on CVH discussions, adjusting for practice. RESULTS Five UC and four AH-HA practices enrolled 645 survivors (82% breast, 8% endometrial, 5% colorectal, and 5% lymphoma, prostate, or multiple types) from October 1, 2020, to February 28, 2023. Most survivors were female (96%; 84% White/non-Hispanic, 8% Black; 3% Hispanic). Nearly all survivors (98%) in AH-HA practices reported a discussion for >= 1 nonideal or missing CVH factor compared with 55% in UC (P < .001). The average number of survivor-reported factors discussed was higher in AH-HA compared with UC (mean, 4.06 v 1.27; P < .001), as were EHR-documented discussions (3.83 v 0.77; P = .03). Survivors in AH-HA practices were also significantly more likely to report a recommendation to see a primary care provider (39%) compared with UC practices (25%, P = .02). Reported recommendations to see a cardiologist were low (approximately 6%) and did not differ between groups. CONCLUSION The AH-HA tool was effective at promoting CVH discussions during routine follow-up care for survivors and recommendations to consult primary care. | This study tested an electronic tool (AH-HA) in cancer survivors to help doctors discuss heart health during routine check-ups. The tool showed key heart health factors pulled from patient records, like blood pressure and diet. Compared to usual care, survivors using AH-HA were far more likely to talk about heart health (98% vs. 55%) and received more advice to see a primary care doctor (39% vs. 25%). The tool worked well to encourage heart health conversations but didn’t increase referrals to cardiologists. Most participants were White, female breast cancer survivors. | This study tested an electronic tool (AH-HA) that helps cancer survivors and their doctors discuss heart health during follow-up visits. The tool automatically pulled heart-related health data (like blood pressure, diet, and activity levels) from patient records and highlighted areas needing attention. Compared to usual care, survivors using AH-HA were much more likely to talk with their doctors about heart health (98% vs. 55%) and received more advice to see a primary care provider. However, referrals to heart specialists were rare in both groups. The tool successfully improved heart-health conversations in cancer survivors. | This study tested an electronic tool (AH-HA) that helps doctors and cancer survivors talk about heart health during follow-up visits. The tool showed heart risk factors (like blood pressure, diet, and smoking) pulled from health records, alongside past cancer treatments that could affect the heart. Compared to usual care, survivors at clinics using AH-HA were much more likely to discuss heart health (98% vs. 55%) and got more advice to see a primary care doctor (39% vs. 25%). The tool worked well to encourage these important conversations. |
| JCO | 5 | 10.1200/JCO.24.00347 | Grok | PURPOSE Guidelines recommend cardiovascular (CV) risk assessment and counseling for cancer survivors. This study evaluated the automated heart-health assessment (AH-HA) clinical decision support tool to promote provider-patient CV health (CVH) discussions in outpatient oncology. METHODS The AH-HA trial (WF-1804CD), coordinated by the Wake Forest National Cancer Institute Community Oncology Research Program Research Base, randomized practices to the AH-HA tool or usual care (UC) and enrolled survivors receiving routine care >= 6 months after curative cancer treatment. The tool displayed American Heart Association Life's Simple 7 CVH factors (BMI, physical activity, diet, smoking status, blood pressure, cholesterol, and glucose), populated from the electronic health record (EHR), alongside cancer treatments received with cardiotoxic potential. The primary end point was survivor-reported discussion of nonideal or missing CVH factors. A mixed-effects logistic regression model assessed the effect of AH-HA on CVH discussions, adjusting for practice. RESULTS Five UC and four AH-HA practices enrolled 645 survivors (82% breast, 8% endometrial, 5% colorectal, and 5% lymphoma, prostate, or multiple types) from October 1, 2020, to February 28, 2023. Most survivors were female (96%; 84% White/non-Hispanic, 8% Black; 3% Hispanic). Nearly all survivors (98%) in AH-HA practices reported a discussion for >= 1 nonideal or missing CVH factor compared with 55% in UC (P < .001). The average number of survivor-reported factors discussed was higher in AH-HA compared with UC (mean, 4.06 v 1.27; P < .001), as were EHR-documented discussions (3.83 v 0.77; P = .03). Survivors in AH-HA practices were also significantly more likely to report a recommendation to see a primary care provider (39%) compared with UC practices (25%, P = .02). Reported recommendations to see a cardiologist were low (approximately 6%) and did not differ between groups. CONCLUSION The AH-HA tool was effective at promoting CVH discussions during routine follow-up care for survivors and recommendations to consult primary care. | The AH-HA trial tested a tool that uses electronic health records to help doctors discuss heart health with cancer survivors in community oncology practices. The tool showed survivors’ heart health factors, like diet and blood pressure, and highlighted cancer treatments that might affect the heart. In the study, 645 survivors were split between practices using the AH-HA tool and those using standard care. Survivors in AH-HA practices were much more likely to talk about heart health issues (98% vs. 55%) and had more factors discussed. They were also more likely to be advised to see a primary care doctor, though referrals to cardiologists were low and similar in both groups. The tool effectively encouraged heart health conversations during routine cancer follow-up visits. | The AH-HA trial tested a tool that uses electronic health records to show cancer survivors’ heart health risks during routine oncology visits. The study included 645 survivors, mostly women with breast cancer, across nine community oncology practices. Practices using the AH-HA tool had far more survivors (98%) discussing heart health issues like diet, exercise, or smoking compared to usual care (55%). Survivors in AH-HA practices also discussed more heart health factors and were more likely to be referred to primary care, though referrals to cardiologists remained low and similar in both groups. | The AH-HA trial tested a tool that uses electronic health records to help cancer survivors and their doctors discuss heart health during routine oncology visits. The study, conducted from 2020 to 2023, included 645 survivors, mostly breast cancer patients, across nine community practices. Practices using the AH-HA tool, which showed heart health factors like BMI, diet, and blood pressure, had 98% of survivors reporting discussions about heart health issues compared to 55% in usual care practices. Survivors in AH-HA practices also discussed more heart health factors and were more likely to be referred to primary care, though referrals to cardiologists were low and similar in both groups. |
| NatRevCan | 1 | 10.1038/s41568-025-00810-2 | Open AI | Phosphoinositide kinases, extending beyond the well-known phosphoinositide 3-kinase (PI3K), are key players in the dynamic and site-specific phosphorylation of lipid phosphoinositides. Unlike PI3Ks, phosphatidylinositol 4-kinases (PI4Ks) and phosphatidylinositol phosphate kinases (PIPKs) do not usually exhibit mutational alterations, but mostly show altered expression in tumours, orchestrating a broad spectrum of signalling, metabolic and immune processes, all of which are crucial in the pathogenesis of cancer. Dysregulation of PI4Ks and PIPKs has been associated with various malignancies, which has sparked considerable interest towards their therapeutic targeting. In this Review we summarize the current understanding of the lesser-studied phosphoinositide kinase families, PI4K and PIPK, focusing on their functions and relevance in cancer. In addition, we provide an overview of ongoing efforts driving the preclinical and clinical development of phosphoinositide kinase-targeting molecules. | Phosphoinositide kinases, especially types called PI4Ks and PIPKs, play important roles in controlling cell signals and functions linked to cancer. Unlike the well-known PI3K, these kinases don’t often have mutations but can have changed activity levels in tumors. This change affects many processes that help cancer grow and survive. Researchers are studying these kinases to better understand cancer and to develop new treatments targeting them. | Phosphoinositide kinases are enzymes that modify specific fats in cells to control important processes like signaling, metabolism, and immunity. While one type, PI3K, is well-known and often mutated in cancer, other types called PI4Ks and PIPKs usually change in how much they are made, not their structure. These changes can affect cancer development, making them important targets for new treatments. This review explains what we know about these less-studied kinases and the progress in developing drugs that target them for cancer therapy. | Phosphoinositide kinases, including lesser-known types like PI4Ks and PIPKs, help control important cell processes by modifying certain lipids. Unlike the well-known PI3K, these kinases usually don’t have mutations but show changes in how much they are made in cancer. These changes affect many cell signals and activities related to cancer growth. Scientists are studying these kinases more to understand their roles and to develop new cancer treatments targeting them. |
| NatRevCan | 1 | 10.1038/s41568-025-00810-3 | Claude | Phosphoinositide kinases, extending beyond the well-known phosphoinositide 3-kinase (PI3K), are key players in the dynamic and site-specific phosphorylation of lipid phosphoinositides. Unlike PI3Ks, phosphatidylinositol 4-kinases (PI4Ks) and phosphatidylinositol phosphate kinases (PIPKs) do not usually exhibit mutational alterations, but mostly show altered expression in tumours, orchestrating a broad spectrum of signalling, metabolic and immune processes, all of which are crucial in the pathogenesis of cancer. Dysregulation of PI4Ks and PIPKs has been associated with various malignancies, which has sparked considerable interest towards their therapeutic targeting. In this Review we summarize the current understanding of the lesser-studied phosphoinositide kinase families, PI4K and PIPK, focusing on their functions and relevance in cancer. In addition, we provide an overview of ongoing efforts driving the preclinical and clinical development of phosphoinositide kinase-targeting molecules. | Phosphoinositide kinases are enzymes that add phosphate groups to certain fats in cells, and they play important roles in cancer development. While the most famous one (PI3K) is often mutated in cancer, two other types called PI4K and PIPK aren't usually mutated but are made in abnormal amounts in tumors. These enzymes control many cellular processes including signaling, metabolism, and immune responses that are critical for cancer growth. Because they're so important in cancer, scientists are working to develop new drugs that can target these enzymes as potential cancer treatments. | Phosphoinositide kinases are enzymes that modify certain fats in cells by adding phosphate groups. While the well-known PI3K enzyme often has genetic mutations in cancer, two other types called PI4Ks and PIPKs usually don't have mutations but are produced in abnormal amounts in tumors. These enzymes control important cell processes including signaling, metabolism, and immune responses that are all involved in cancer development. Because PI4Ks and PIPKs are disrupted in many types of cancer, scientists are working to develop new drugs that target these enzymes as potential cancer treatments. | This text explains how certain enzymes called phosphoinositide kinases (specifically PI4K and PIPK types) play important roles in cancer development. Unlike the more famous PI3K enzymes that are often mutated in cancer, these enzymes typically become overactive in tumors without being directly damaged. When they don't work properly, they disrupt normal cell signaling, metabolism, and immune responses, which can lead to cancer. Because of their connection to various types of cancer, scientists are now actively researching and developing new drugs that target these enzymes as potential cancer treatments. |
| NatRevCan | 1 | 10.1038/s41568-025-00810-4 | Gemini | Phosphoinositide kinases, extending beyond the well-known phosphoinositide 3-kinase (PI3K), are key players in the dynamic and site-specific phosphorylation of lipid phosphoinositides. Unlike PI3Ks, phosphatidylinositol 4-kinases (PI4Ks) and phosphatidylinositol phosphate kinases (PIPKs) do not usually exhibit mutational alterations, but mostly show altered expression in tumours, orchestrating a broad spectrum of signalling, metabolic and immune processes, all of which are crucial in the pathogenesis of cancer. Dysregulation of PI4Ks and PIPKs has been associated with various malignancies, which has sparked considerable interest towards their therapeutic targeting. In this Review we summarize the current understanding of the lesser-studied phosphoinositide kinase families, PI4K and PIPK, focusing on their functions and relevance in cancer. In addition, we provide an overview of ongoing efforts driving the preclinical and clinical development of phosphoinositide kinase-targeting molecules. | This review article explains that certain enzymes called phosphoinositide kinases, specifically PI4Ks and PIPKs, are important in cancer development. Unlike the more famous PI3K enzyme, PI4Ks and PIPKs don't usually have genetic mutations in cancer cells. Instead, they become overactive or are present in higher amounts, which affects various cell processes like signaling, metabolism, and immune responses. This dysregulation contributes to cancer growth. The article explores how these enzymes work and their role in different types of cancer, and also discusses current and future efforts to develop drugs that target these specific kinases as a new way to treat cancer. | In the world of cancer research, a group of enzymes called phosphoinositide kinases are getting a lot of attention. While most people know about PI3K, this article focuses on two lesser-known members: PI4Ks and PIPKs. Unlike PI3K, these enzymes are not usually changed by mutations, but their levels are often altered in tumors. These changes can affect important cell processes like metabolism and immune responses, which helps cancer grow and spread. Scientists are now very interested in developing drugs that can target and block these enzymes as a new way to treat various cancers. | Phosphoinositide kinases, which are enzymes that modify fats in cells, are important in cancer. While most people know about phosphoinositide 3-kinase (PI3K), other types like PI4K and PIPK are also crucial. Instead of being mutated, these other kinases are often found in altered amounts in tumors, where they play a role in cell signaling, metabolism, and immune responses. Because these kinases are linked to different types of cancer, scientists are very interested in developing drugs that target them. The research described in the abstract focuses on understanding the function of PI4K and PIPK in cancer and provides an overview of current and future treatments that target these enzymes. |
| NatRevCan | 1 | 10.1038/s41568-025-00810-5 | DeepSeek | Phosphoinositide kinases, extending beyond the well-known phosphoinositide 3-kinase (PI3K), are key players in the dynamic and site-specific phosphorylation of lipid phosphoinositides. Unlike PI3Ks, phosphatidylinositol 4-kinases (PI4Ks) and phosphatidylinositol phosphate kinases (PIPKs) do not usually exhibit mutational alterations, but mostly show altered expression in tumours, orchestrating a broad spectrum of signalling, metabolic and immune processes, all of which are crucial in the pathogenesis of cancer. Dysregulation of PI4Ks and PIPKs has been associated with various malignancies, which has sparked considerable interest towards their therapeutic targeting. In this Review we summarize the current understanding of the lesser-studied phosphoinositide kinase families, PI4K and PIPK, focusing on their functions and relevance in cancer. In addition, we provide an overview of ongoing efforts driving the preclinical and clinical development of phosphoinositide kinase-targeting molecules. | This review discusses how certain enzymes called phosphoinositide kinases, especially PI4Ks and PIPKs, play important roles in cancer by affecting cell signaling, metabolism, and immune responses. Unlike the more studied PI3Ks, these enzymes are usually not mutated in tumors but are often overactive or overexpressed. Their dysregulation is linked to various cancers, making them promising targets for new therapies. The article summarizes their functions in cancer and highlights current efforts to develop drugs that block these enzymes. | This review discusses how certain enzymes called phosphoinositide kinases, especially PI4Ks and PIPKs (not just the well-known PI3K), play important roles in cancer by affecting cell signaling, metabolism, and immunity. While these enzymes aren’t usually mutated in tumors, their activity is often altered, contributing to cancer growth. Researchers are now exploring drugs that target these enzymes as potential new treatments for cancer. The article summarizes what we know about PI4Ks and PIPKs and highlights ongoing efforts to develop therapies against them. | This review discusses how certain enzymes called phosphoinositide kinases, especially PI4Ks and PIPKs, play important roles in cancer by affecting cell signaling, metabolism, and immune responses. While these enzymes are less studied than the well-known PI3K, they are often overactive in tumors and could be promising targets for new cancer treatments. The article summarizes their functions in cancer and highlights ongoing research into drugs that block these enzymes. |
| NatRevCan | 1 | 10.1038/s41568-025-00810-6 | Grok | Phosphoinositide kinases, extending beyond the well-known phosphoinositide 3-kinase (PI3K), are key players in the dynamic and site-specific phosphorylation of lipid phosphoinositides. Unlike PI3Ks, phosphatidylinositol 4-kinases (PI4Ks) and phosphatidylinositol phosphate kinases (PIPKs) do not usually exhibit mutational alterations, but mostly show altered expression in tumours, orchestrating a broad spectrum of signalling, metabolic and immune processes, all of which are crucial in the pathogenesis of cancer. Dysregulation of PI4Ks and PIPKs has been associated with various malignancies, which has sparked considerable interest towards their therapeutic targeting. In this Review we summarize the current understanding of the lesser-studied phosphoinositide kinase families, PI4K and PIPK, focusing on their functions and relevance in cancer. In addition, we provide an overview of ongoing efforts driving the preclinical and clinical development of phosphoinositide kinase-targeting molecules. | Phosphoinositide kinases, including lesser-known types like PI4Ks and PIPKs, play a big role in cancer by controlling how certain fats in cells are modified, affecting cell signaling, metabolism, and immune responses. Unlike other similar enzymes, these kinases usually don’t have genetic mutations but show changed activity levels in tumors. This makes them important in cancer development, and researchers are working on drugs to target them. This review explains their roles in cancer and updates on efforts to develop treatments targeting these enzymes. | Phosphoinositide kinases, including phosphatidylinositol 4-kinases (PI4Ks) and phosphatidylinositol phosphate kinases (PIPKs), play a big role in cancer by controlling how certain fats in cells are modified, which affects cell signaling, metabolism, and immune responses. Unlike other similar enzymes, these kinases don’t often have mutations but show changed levels in tumors, making them important in cancer development. This review explains their functions in cancer and highlights efforts to develop drugs targeting these kinases to treat cancer. | Phosphoinositide kinases, including PI4Ks and PIPKs, are enzymes that modify lipid molecules and play important roles in cancer development by influencing cell signaling, metabolism, and immune responses. Unlike PI3Ks, these kinases are not typically mutated but show altered levels in tumors. This review discusses their functions in cancer and highlights ongoing efforts to develop drugs targeting these enzymes for cancer treatment. |
| NatRevCan | 2 | 10.1038/s41568-024-00788-3 | Open AI | Tumours of the appendix - a vestigial digestive organ attached to the colon - are rare. Although we estimate that around 3,000 new appendiceal cancer cases are diagnosed annually in the USA, the challenges of accurately diagnosing and identifying this tumour type suggest that this number may underestimate true population incidence. In the current absence of disease-specific screening and diagnostic imaging modalities, or well-established risk factors, the incidental discovery of appendix tumours is often prompted by acute presentations mimicking appendicitis or when the tumour has already spread into the abdominal cavity - wherein the potential misclassification of appendiceal tumours as malignancies of the colon and ovaries also increases. Notwithstanding these diagnostic difficulties, our understanding of appendix carcinogenesis has advanced in recent years. However, there persist considerable challenges to accelerating the pace of research discoveries towards the path to improved treatments and cures for patients with this group of orphan malignancies. The premise of this Expert Recommendation article is to discuss the current state of the field, to delineate unique challenges for the study of appendiceal tumours, and to propose key priority research areas that will deliver a more complete picture of appendix carcinogenesis and metastasis. The Appendix Cancer Pseudomyxoma Peritonei (ACPMP) Research Foundation Scientific Think Tank delivered a consensus of core research priorities for appendiceal tumours that are poised to be ground-breaking and transformative for scientific discovery and innovation. On the basis of these six research areas, here, we define the first 'cells to society' research framework for appendix tumours. | Appendix tumors are rare and often hard to diagnose because they can look like other diseases or cancer types. About 3,000 cases are found yearly in the USA, but the true number might be higher. These tumors are usually discovered by accident during surgery or when the cancer has spread. Although research has improved our understanding of how these tumors develop, there are still many challenges in studying and treating them. Experts have identified six key research areas to better understand appendix cancer from its basic cell biology to its impact on society, aiming to improve diagnosis and treatment in the future. | Appendix tumors are rare and hard to diagnose, often discovered by accident or when symptoms look like appendicitis. Because there are no specific tests or clear risk factors, many cases might be missed or confused with other cancers. Although recent research has improved our understanding of how these tumors develop, much more work is needed to find better treatments. Experts have identified six key research areas to guide future studies, aiming to improve knowledge and care for people with appendix cancer. | Appendix tumors are rare and hard to diagnose because they often look like other illnesses like appendicitis or cancers of nearby organs. About 3,000 cases are found yearly in the USA, but this might be lower than the real number. There are no specific tests or clear risk factors for these tumors, which makes early detection difficult. Recent research has improved understanding of how these tumors develop, but many challenges remain in finding better treatments. Experts have identified six key research areas to help improve knowledge and care for appendix cancer patients, creating a new comprehensive plan to study these tumors from the smallest cells to the whole society. |
| NatRevCan | 2 | 10.1038/s41568-024-00788-4 | Claude | Tumours of the appendix - a vestigial digestive organ attached to the colon - are rare. Although we estimate that around 3,000 new appendiceal cancer cases are diagnosed annually in the USA, the challenges of accurately diagnosing and identifying this tumour type suggest that this number may underestimate true population incidence. In the current absence of disease-specific screening and diagnostic imaging modalities, or well-established risk factors, the incidental discovery of appendix tumours is often prompted by acute presentations mimicking appendicitis or when the tumour has already spread into the abdominal cavity - wherein the potential misclassification of appendiceal tumours as malignancies of the colon and ovaries also increases. Notwithstanding these diagnostic difficulties, our understanding of appendix carcinogenesis has advanced in recent years. However, there persist considerable challenges to accelerating the pace of research discoveries towards the path to improved treatments and cures for patients with this group of orphan malignancies. The premise of this Expert Recommendation article is to discuss the current state of the field, to delineate unique challenges for the study of appendiceal tumours, and to propose key priority research areas that will deliver a more complete picture of appendix carcinogenesis and metastasis. The Appendix Cancer Pseudomyxoma Peritonei (ACPMP) Research Foundation Scientific Think Tank delivered a consensus of core research priorities for appendiceal tumours that are poised to be ground-breaking and transformative for scientific discovery and innovation. On the basis of these six research areas, here, we define the first 'cells to society' research framework for appendix tumours. | Appendix cancer is a rare disease that affects about 3,000 Americans each year, though the actual number may be higher since it's hard to diagnose. The appendix is a small, unused organ connected to the large intestine. These tumors are often discovered by accident when patients have symptoms that look like appendicitis, or when the cancer has already spread throughout the belly. Because there are no specific screening tests or known risk factors, doctors sometimes mistake appendix cancer for colon or ovarian cancer. While scientists have learned more about how these cancers develop in recent years, research is still challenging because the disease is so uncommon. A group of experts has now identified six key research areas that could lead to major breakthroughs in understanding and treating appendix cancer, creating the first comprehensive research plan for this rare disease. | Appendix cancer is a rare disease affecting about 3,000 Americans yearly, though the actual number may be higher since it's hard to diagnose. The appendix is a small, unused organ connected to the large intestine. These cancers are often discovered by accident when patients think they have appendicitis or when the cancer has already spread throughout the abdomen, making them harder to identify and treat. While scientists have learned more about how appendix cancer develops in recent years, research progress remains slow due to the disease's rarity. This article discusses the current challenges in studying appendix tumors and outlines six key research priorities identified by cancer experts that could lead to breakthrough discoveries and better treatments for patients with this uncommon but serious disease. | Tumors in the appendix (a small organ attached to the large intestine) are very rare, with about 3,000 new cases diagnosed each year in the US, though the actual number may be higher since these cancers are hard to detect and diagnose. Because there are no screening tests or known risk factors, appendix tumors are usually discovered by accident when patients have symptoms that seem like appendicitis or when the cancer has already spread throughout the abdomen. While scientists have learned more about how these tumors develop in recent years, research progress remains slow due to the rarity of the disease. This article discusses current knowledge about appendix cancers and outlines six key research priorities identified by experts that could lead to major breakthroughs in understanding and treating these uncommon but serious cancers. |
| NatRevCan | 2 | 10.1038/s41568-024-00788-5 | Gemini | Tumours of the appendix - a vestigial digestive organ attached to the colon - are rare. Although we estimate that around 3,000 new appendiceal cancer cases are diagnosed annually in the USA, the challenges of accurately diagnosing and identifying this tumour type suggest that this number may underestimate true population incidence. In the current absence of disease-specific screening and diagnostic imaging modalities, or well-established risk factors, the incidental discovery of appendix tumours is often prompted by acute presentations mimicking appendicitis or when the tumour has already spread into the abdominal cavity - wherein the potential misclassification of appendiceal tumours as malignancies of the colon and ovaries also increases. Notwithstanding these diagnostic difficulties, our understanding of appendix carcinogenesis has advanced in recent years. However, there persist considerable challenges to accelerating the pace of research discoveries towards the path to improved treatments and cures for patients with this group of orphan malignancies. The premise of this Expert Recommendation article is to discuss the current state of the field, to delineate unique challenges for the study of appendiceal tumours, and to propose key priority research areas that will deliver a more complete picture of appendix carcinogenesis and metastasis. The Appendix Cancer Pseudomyxoma Peritonei (ACPMP) Research Foundation Scientific Think Tank delivered a consensus of core research priorities for appendiceal tumours that are poised to be ground-breaking and transformative for scientific discovery and innovation. On the basis of these six research areas, here, we define the first 'cells to society' research framework for appendix tumours. | Tumors of the appendix are rare and often difficult to diagnose, sometimes being mistaken for appendicitis or other cancers. This article proposes a new "cells to society" framework to guide research on appendiceal tumors. This framework is based on six key research areas identified by the Appendix Cancer Pseudomyxoma Peritonei (ACPMP) Research Foundation Scientific Think Tank, with the goal of accelerating discoveries and improving treatments for patients. | Tumors of the appendix are rare and often hard to diagnose. They are frequently discovered by chance during surgery for appendicitis or after the tumor has spread. Despite these difficulties, recent research has improved our understanding of how these tumors develop. A new "cells to society" framework has been created to guide future research and speed up the development of better treatments for patients with this rare cancer. | Tumors in the appendix are rare and difficult to diagnose, so the actual number of people with this cancer may be higher than currently thought. The tumors are often discovered by chance, sometimes after they have already spread. Although scientists have learned more about this cancer recently, it is still a challenge to turn these discoveries into better treatments. To speed up this process, a group of experts has created the first-ever "cells to society" research framework, which outlines six key areas for future research. |
| NatRevCan | 2 | 10.1038/s41568-024-00788-6 | DeepSeek | Tumours of the appendix - a vestigial digestive organ attached to the colon - are rare. Although we estimate that around 3,000 new appendiceal cancer cases are diagnosed annually in the USA, the challenges of accurately diagnosing and identifying this tumour type suggest that this number may underestimate true population incidence. In the current absence of disease-specific screening and diagnostic imaging modalities, or well-established risk factors, the incidental discovery of appendix tumours is often prompted by acute presentations mimicking appendicitis or when the tumour has already spread into the abdominal cavity - wherein the potential misclassification of appendiceal tumours as malignancies of the colon and ovaries also increases. Notwithstanding these diagnostic difficulties, our understanding of appendix carcinogenesis has advanced in recent years. However, there persist considerable challenges to accelerating the pace of research discoveries towards the path to improved treatments and cures for patients with this group of orphan malignancies. The premise of this Expert Recommendation article is to discuss the current state of the field, to delineate unique challenges for the study of appendiceal tumours, and to propose key priority research areas that will deliver a more complete picture of appendix carcinogenesis and metastasis. The Appendix Cancer Pseudomyxoma Peritonei (ACPMP) Research Foundation Scientific Think Tank delivered a consensus of core research priorities for appendiceal tumours that are poised to be ground-breaking and transformative for scientific discovery and innovation. On the basis of these six research areas, here, we define the first 'cells to society' research framework for appendix tumours. | This article discusses the challenges of studying rare appendix tumors, which are often misdiagnosed or discovered late due to unclear risk factors and lack of specific screening methods. Despite recent progress in understanding these cancers, research faces hurdles in improving diagnosis and treatment. The ACPMP Research Foundation identified six key research areas to advance knowledge and care for appendix tumors, leading to the creation of a comprehensive "cells to society" research framework to guide future breakthroughs. | This article discusses the challenges of studying rare appendix tumors, which are often misdiagnosed or discovered late due to a lack of specific screening methods and unclear risk factors. While research has improved our understanding of these cancers, progress in treatment remains slow. The ACPMP Research Foundation identified six key research areas to advance knowledge and improve patient outcomes. The article proposes a "cells to society" framework to guide future studies, aiming to better understand appendix cancer development and spread. | This article discusses appendix tumors, which are rare and often misdiagnosed, leading to underestimated case numbers. Diagnosing these tumors is challenging because they are usually found incidentally during appendicitis-like symptoms or after spreading, and they can be confused with colon or ovarian cancers. While research has improved our understanding, progress in treatment remains slow. The article highlights key research priorities identified by experts to better understand and treat these tumors, proposing a "cells to society" framework to advance scientific discoveries and patient care. |
| NatRevCan | 2 | 10.1038/s41568-024-00788-7 | Grok | Tumours of the appendix - a vestigial digestive organ attached to the colon - are rare. Although we estimate that around 3,000 new appendiceal cancer cases are diagnosed annually in the USA, the challenges of accurately diagnosing and identifying this tumour type suggest that this number may underestimate true population incidence. In the current absence of disease-specific screening and diagnostic imaging modalities, or well-established risk factors, the incidental discovery of appendix tumours is often prompted by acute presentations mimicking appendicitis or when the tumour has already spread into the abdominal cavity - wherein the potential misclassification of appendiceal tumours as malignancies of the colon and ovaries also increases. Notwithstanding these diagnostic difficulties, our understanding of appendix carcinogenesis has advanced in recent years. However, there persist considerable challenges to accelerating the pace of research discoveries towards the path to improved treatments and cures for patients with this group of orphan malignancies. The premise of this Expert Recommendation article is to discuss the current state of the field, to delineate unique challenges for the study of appendiceal tumours, and to propose key priority research areas that will deliver a more complete picture of appendix carcinogenesis and metastasis. The Appendix Cancer Pseudomyxoma Peritonei (ACPMP) Research Foundation Scientific Think Tank delivered a consensus of core research priorities for appendiceal tumours that are poised to be ground-breaking and transformative for scientific discovery and innovation. On the basis of these six research areas, here, we define the first 'cells to society' research framework for appendix tumours. | Appendiceal tumors, rare cancers of the appendix, are hard to diagnose and often found by chance during procedures for other conditions, with about 3,000 new cases yearly in the USA, though this may be an underestimate. Challenges include the lack of specific screening tools, unclear risk factors, and frequent misdiagnosis as colon or ovarian cancer. Recent progress in understanding these tumors has been made, but more research is needed to improve treatments. This article outlines a new "cells to society" research framework, based on six key areas identified by the Appendix Cancer Pseudomyxoma Peritonei Research Foundation, to advance scientific discoveries and transform care for patients with these rare cancers. | Appendix tumors are rare cancers, with about 3,000 new cases diagnosed each year in the USA, though this number may be higher due to challenges in accurate diagnosis. Often found by chance during symptoms like appendicitis or when the cancer has spread, these tumors are hard to detect because there are no specific screening tools or clear risk factors. Recent progress has improved our understanding of how these tumors form, but more research is needed to develop better treatments. This article discusses the current state of appendix tumor research and outlines six key research priorities, forming the first "cells to society" framework to guide future discoveries and improve patient outcomes. | Appendiceal tumors, rare cancers found in the appendix, are often hard to diagnose due to limited screening tools and unclear risk factors, with about 3,000 new cases yearly in the USA, though this may be an underestimate. These tumors are frequently discovered by chance during appendicitis-like episodes or after spreading to the abdominal cavity, where they may be mistaken for colon or ovarian cancers. Despite recent progress in understanding how these tumors form, challenges remain in speeding up research for better treatments. This article outlines a new "cells to society" research framework, based on six key priorities identified by the ACPMP Research Foundation, to advance scientific discovery and improve outcomes for patients with these rare cancers. |
| NatRevCan | 3 | 10.1038/s41568-025-00841-9 | Open AI | In the ongoing battle against cancer, the natural world provides promising inspiration for designing novel therapeutic strategies. The field of comparative oncology offers a valuable source of such inspiration. By combining evolutionary biology, ecology, veterinary medicine and clinical oncology, comparative oncology aims to better understand cancer, especially by highlighting taxa that are strongly resistant or susceptible to cancer and to identify the molecular and cellular mechanisms underlying the remarkable cancer resistance of some taxa. Such studies hold profound implications for human cancer research and treatment, and increase the probability of detecting therapeutic avenues that are non-toxic to healthy cells and tissues. This Perspective underscores the importance of comparative oncology, emphasizes its relevance, and showcases recent breakthroughs in identifying natural cancer resistance mechanisms and opportunities for clinical translation. We advocate for a better integration of cancer research on non-conventional model species into oncology and we urge enhanced cooperation between clinicians and comparative oncologists to advance cancer prevention or treatment strategies. | Comparative oncology studies how different animals naturally resist or get cancer, using ideas from biology and medicine. By learning from species that rarely get cancer, scientists hope to discover new, safer ways to treat cancer in people. This approach can help find treatments that don’t harm healthy cells, and working together with doctors can improve cancer prevention and care. | Comparative oncology studies how different animals naturally resist or get cancer, combining biology, ecology, and medicine to learn new ways to fight cancer. By understanding why some species rarely get cancer, researchers hope to find safer, more effective treatments for humans. This approach encourages doctors and scientists to work together using insights from nature to improve cancer prevention and care. | Comparative oncology studies how different animals naturally resist or get cancer to help us understand the disease better. By learning from nature and combining knowledge from biology, ecology, and medicine, this field aims to find new, safer cancer treatments for humans. It encourages more teamwork between doctors and researchers studying animals to improve cancer prevention and care. |
| NatRevCan | 3 | 10.1038/s41568-025-00841-10 | Claude | In the ongoing battle against cancer, the natural world provides promising inspiration for designing novel therapeutic strategies. The field of comparative oncology offers a valuable source of such inspiration. By combining evolutionary biology, ecology, veterinary medicine and clinical oncology, comparative oncology aims to better understand cancer, especially by highlighting taxa that are strongly resistant or susceptible to cancer and to identify the molecular and cellular mechanisms underlying the remarkable cancer resistance of some taxa. Such studies hold profound implications for human cancer research and treatment, and increase the probability of detecting therapeutic avenues that are non-toxic to healthy cells and tissues. This Perspective underscores the importance of comparative oncology, emphasizes its relevance, and showcases recent breakthroughs in identifying natural cancer resistance mechanisms and opportunities for clinical translation. We advocate for a better integration of cancer research on non-conventional model species into oncology and we urge enhanced cooperation between clinicians and comparative oncologists to advance cancer prevention or treatment strategies. | Scientists are studying how different animals naturally resist cancer to find new ways to treat the disease in humans. This field, called comparative oncology, looks at why some animals almost never get cancer while others are more likely to develop it. By understanding the biological mechanisms that protect certain animals from cancer, researchers hope to develop safer treatments that target cancer cells without harming healthy tissue. The authors argue that doctors and animal cancer researchers should work together more closely to discover these natural anti-cancer strategies and turn them into real medical treatments for people. | Scientists are studying how different animals deal with cancer to help find better treatments for humans. Some animals are naturally very good at fighting off cancer, while others get it easily. By looking at what makes these cancer-resistant animals special - like the molecules and cells that protect them - researchers hope to discover new ways to treat human cancer that won't harm healthy parts of the body. This field, called comparative oncology, combines knowledge from evolution, animal medicine, and cancer research. The authors believe that doctors and animal researchers should work together more closely to develop these nature-inspired cancer treatments. | Scientists are studying how different animals in nature fight off cancer to help develop better treatments for humans. This field, called comparative oncology, looks at why some animals rarely get cancer while others get it more often. By understanding the natural ways animals protect themselves from cancer, researchers hope to create new therapies that can kill cancer cells without harming healthy parts of the body. The authors argue that doctors and animal cancer researchers should work together more closely to turn these natural cancer-fighting discoveries into treatments that could help human patients. |
| NatRevCan | 3 | 10.1038/s41568-025-00841-11 | Gemini | In the ongoing battle against cancer, the natural world provides promising inspiration for designing novel therapeutic strategies. The field of comparative oncology offers a valuable source of such inspiration. By combining evolutionary biology, ecology, veterinary medicine and clinical oncology, comparative oncology aims to better understand cancer, especially by highlighting taxa that are strongly resistant or susceptible to cancer and to identify the molecular and cellular mechanisms underlying the remarkable cancer resistance of some taxa. Such studies hold profound implications for human cancer research and treatment, and increase the probability of detecting therapeutic avenues that are non-toxic to healthy cells and tissues. This Perspective underscores the importance of comparative oncology, emphasizes its relevance, and showcases recent breakthroughs in identifying natural cancer resistance mechanisms and opportunities for clinical translation. We advocate for a better integration of cancer research on non-conventional model species into oncology and we urge enhanced cooperation between clinicians and comparative oncologists to advance cancer prevention or treatment strategies. | Comparative oncology studies cancer in different animal species to find out why some animals are highly resistant to it. By combining knowledge from different fields like evolutionary biology and veterinary medicine, this research aims to discover new ways to prevent and treat human cancer. The goal is to find new therapies that are less harmful to healthy cells and tissues, and the field encourages closer collaboration between doctors and scientists to achieve these goals. | Comparative oncology is a field that studies cancer in different animal species to understand why some animals are more resistant to it than others. By combining knowledge from veterinary medicine, evolutionary biology, and human oncology, researchers hope to discover new ways to prevent and treat cancer in people. The goal is to find new therapies that are less toxic to healthy cells and tissues by learning from how nature has already solved some of these problems. This approach encourages collaboration between clinicians and scientists to speed up the development of new cancer treatments. | Comparative oncology studies how different species get cancer, or in some cases, don't get cancer. By looking at animals that are naturally resistant to cancer, like certain bats or naked mole rats, researchers can discover new ways to fight the disease in humans. This field combines evolutionary biology, veterinary medicine, and human oncology to find treatments that are less toxic than current options. The goal is to better integrate these findings into cancer research and encourage collaboration between doctors and comparative oncologists to develop new strategies for preventing and treating cancer. |
| NatRevCan | 3 | 10.1038/s41568-025-00841-12 | DeepSeek | In the ongoing battle against cancer, the natural world provides promising inspiration for designing novel therapeutic strategies. The field of comparative oncology offers a valuable source of such inspiration. By combining evolutionary biology, ecology, veterinary medicine and clinical oncology, comparative oncology aims to better understand cancer, especially by highlighting taxa that are strongly resistant or susceptible to cancer and to identify the molecular and cellular mechanisms underlying the remarkable cancer resistance of some taxa. Such studies hold profound implications for human cancer research and treatment, and increase the probability of detecting therapeutic avenues that are non-toxic to healthy cells and tissues. This Perspective underscores the importance of comparative oncology, emphasizes its relevance, and showcases recent breakthroughs in identifying natural cancer resistance mechanisms and opportunities for clinical translation. We advocate for a better integration of cancer research on non-conventional model species into oncology and we urge enhanced cooperation between clinicians and comparative oncologists to advance cancer prevention or treatment strategies. | Comparative oncology studies how different animals resist or get cancer to find new ways to fight the disease in humans. By looking at species with natural cancer resistance, scientists can discover safe and effective treatments that don’t harm healthy cells. This field combines evolutionary biology, ecology, and medicine to improve cancer research. The article calls for more collaboration between doctors and researchers working on unconventional animal models to develop better prevention and treatment options. | This article highlights how studying cancer in different animals (comparative oncology) can help improve human cancer research and treatments. By looking at species that are highly resistant or prone to cancer, scientists can uncover natural mechanisms that fight the disease. These discoveries could lead to safer, more effective therapies. The authors encourage stronger collaboration between doctors and researchers working on unconventional animal models to accelerate progress in cancer prevention and treatment. | Comparative oncology studies cancer across different animal species to find new ways to fight the disease. By looking at animals that are highly resistant or vulnerable to cancer, scientists can uncover natural mechanisms that protect against tumors. This approach could lead to safer, more effective treatments for humans. The field encourages collaboration between doctors and researchers to explore these natural defenses and improve cancer therapies. The goal is to turn discoveries from nature into real-world medical solutions. |
| NatRevCan | 3 | 10.1038/s41568-025-00841-13 | Grok | In the ongoing battle against cancer, the natural world provides promising inspiration for designing novel therapeutic strategies. The field of comparative oncology offers a valuable source of such inspiration. By combining evolutionary biology, ecology, veterinary medicine and clinical oncology, comparative oncology aims to better understand cancer, especially by highlighting taxa that are strongly resistant or susceptible to cancer and to identify the molecular and cellular mechanisms underlying the remarkable cancer resistance of some taxa. Such studies hold profound implications for human cancer research and treatment, and increase the probability of detecting therapeutic avenues that are non-toxic to healthy cells and tissues. This Perspective underscores the importance of comparative oncology, emphasizes its relevance, and showcases recent breakthroughs in identifying natural cancer resistance mechanisms and opportunities for clinical translation. We advocate for a better integration of cancer research on non-conventional model species into oncology and we urge enhanced cooperation between clinicians and comparative oncologists to advance cancer prevention or treatment strategies. | Comparative oncology is a field that studies cancer across different species to find new ways to fight the disease. By looking at animals that are either very resistant or prone to cancer, scientists can learn about the biological mechanisms behind cancer resistance. This research could lead to new, safer cancer treatments for humans. The article highlights the importance of this approach, recent discoveries, and the need for closer collaboration between doctors and researchers studying cancer in various species to improve cancer prevention and treatment. | Comparative oncology is a field that studies cancer by looking at different animals to find new ways to fight the disease. By combining knowledge from evolution, ecology, veterinary medicine, and human cancer research, scientists explore why some animals are very resistant to cancer while others are not. These discoveries help identify safe and effective cancer treatments for humans. This approach encourages collaboration between doctors and researchers to improve cancer prevention and treatment strategies using insights from nature. | Comparative oncology studies cancer by looking at different animals to find out why some are very resistant to it while others are not. By combining knowledge from evolution, ecology, veterinary medicine, and cancer research, scientists aim to discover new, safe ways to treat cancer in humans. This approach highlights unique biological mechanisms in animals that could lead to better cancer prevention and treatment strategies. The text calls for more collaboration between doctors and researchers studying non-traditional animals to advance cancer research. |
| NatRevCan | 4 | 10.1038/s41568-025-00831-w | Open AI | The nervous and immune systems have co-evolved to detect and respond to internal and external threats, working together to restore homeostasis after tissue injury or infection. Sharing several receptors and ligands, they engage in direct cross-talk that substantially influences disease development. The emerging field of cancer neuro-immunity focuses on the intricate interactions between the nervous system, immune responses and tumour growth. Additional findings have revealed that nerve fibres infiltrating peripheral tumours can release neuromodulatory factors that shape both immune cell behaviour and tumour progression. Conversely, tumour-infiltrating immune cells can modify the activity of local neurons, including pain-transmitting nociceptive sensory neurons. Beyond sensory fibres, sympathetic signalling can foster immunosuppression by recruiting myeloid-derived suppressor cells and promoting T cell exhaustion. This Review summarizes current evidence on how neuronal signalling regulates peripheral antitumour immune responses within the tumour microenvironment. We describe the complex, reciprocal interactions among neurons, immune cells and malignant cells, highlighting the key parts played by the peripheral nervous system in modulating immunity against cancer. By understanding this neuro-immune axis, novel therapeutic approaches may be uncovered to strengthen antitumour immunity and enhance responses to existing cancer treatments. | The nervous and immune systems work closely together to protect the body from injury and disease, including cancer. In cancer, nerves growing into tumors can affect how immune cells behave and how the tumor grows, while immune cells can also influence nerve activity. Some nerve signals can weaken the immune system’s ability to fight cancer. This field, called cancer neuro-immunity, studies these complex interactions to help develop new treatments that boost the immune response against tumors. | The nervous and immune systems work closely together to detect dangers and heal the body after injury or infection. In cancer, nerve cells and immune cells interact in ways that affect tumor growth and the body’s ability to fight the cancer. Nerves can influence immune cells to either help or harm the fight against tumors, and immune cells can also affect nerve activity. Understanding these complex connections between nerves, immune cells, and cancer may lead to new treatments that better support the immune system to fight cancer. | The nervous and immune systems work closely together to detect and respond to threats like injury or infection, and this teamwork also affects how cancer grows. Nerves in tumors can send signals that change immune cell behavior and tumor development, while immune cells can affect nerve activity. Certain nerve signals can weaken the immune response against cancer. Studying these interactions helps us understand how nerves influence the body's fight against tumors and may lead to new treatments that boost the immune system to better fight cancer. |
| NatRevCan | 4 | 10.1038/s41568-025-00831-w | Claude | The nervous and immune systems have co-evolved to detect and respond to internal and external threats, working together to restore homeostasis after tissue injury or infection. Sharing several receptors and ligands, they engage in direct cross-talk that substantially influences disease development. The emerging field of cancer neuro-immunity focuses on the intricate interactions between the nervous system, immune responses and tumour growth. Additional findings have revealed that nerve fibres infiltrating peripheral tumours can release neuromodulatory factors that shape both immune cell behaviour and tumour progression. Conversely, tumour-infiltrating immune cells can modify the activity of local neurons, including pain-transmitting nociceptive sensory neurons. Beyond sensory fibres, sympathetic signalling can foster immunosuppression by recruiting myeloid-derived suppressor cells and promoting T cell exhaustion. This Review summarizes current evidence on how neuronal signalling regulates peripheral antitumour immune responses within the tumour microenvironment. We describe the complex, reciprocal interactions among neurons, immune cells and malignant cells, highlighting the key parts played by the peripheral nervous system in modulating immunity against cancer. By understanding this neuro-immune axis, novel therapeutic approaches may be uncovered to strengthen antitumour immunity and enhance responses to existing cancer treatments. | The nervous system (which controls our body's responses) and immune system (which fights disease) work closely together, especially in cancer. When cancer tumors grow, nerve fibers can actually help the cancer by releasing chemicals that weaken immune responses and help tumors spread. At the same time, immune cells fighting the cancer can affect how nerves work, including those that sense pain. This creates a complex back-and-forth relationship between nerves, immune cells, and cancer cells that often favors the tumor's growth. Scientists are studying these connections because understanding how nerves and immunity interact in cancer could lead to new treatments that boost the body's ability to fight tumors. | The nervous system (which controls our body's functions) and immune system (which fights disease) work together to protect us from threats like cancer. Scientists have discovered that nerve fibers in tumors can release chemicals that affect both immune cells and cancer growth, while immune cells can also influence nearby nerves, including those that transmit pain. Some nerve signals can actually help tumors grow by weakening the immune response and making immune cells less effective at fighting cancer. By understanding how nerves and immune cells communicate with each other in and around tumors, researchers hope to develop new treatments that could boost the body's natural ability to fight cancer and make existing cancer therapies work better. | The nervous system and immune system work together like partners to protect the body from threats and heal injuries. In cancer, this partnership becomes complicated - nerves that grow into tumors can release chemicals that either help or hurt the body's ability to fight cancer cells. At the same time, immune cells in tumors can affect how these nerves work, including those that sense pain. Some nerve signals can actually weaken the immune system's cancer-fighting abilities by attracting unhelpful immune cells and wearing out the good ones. Scientists are studying these complex interactions between nerves, immune cells, and cancer cells because understanding how they communicate could lead to new treatments that boost the body's natural ability to fight cancer. |
| NatRevCan | 4 | 10.1038/s41568-025-00831-w | Gemini | The nervous and immune systems have co-evolved to detect and respond to internal and external threats, working together to restore homeostasis after tissue injury or infection. Sharing several receptors and ligands, they engage in direct cross-talk that substantially influences disease development. The emerging field of cancer neuro-immunity focuses on the intricate interactions between the nervous system, immune responses and tumour growth. Additional findings have revealed that nerve fibres infiltrating peripheral tumours can release neuromodulatory factors that shape both immune cell behaviour and tumour progression. Conversely, tumour-infiltrating immune cells can modify the activity of local neurons, including pain-transmitting nociceptive sensory neurons. Beyond sensory fibres, sympathetic signalling can foster immunosuppression by recruiting myeloid-derived suppressor cells and promoting T cell exhaustion. This Review summarizes current evidence on how neuronal signalling regulates peripheral antitumour immune responses within the tumour microenvironment. We describe the complex, reciprocal interactions among neurons, immune cells and malignant cells, highlighting the key parts played by the peripheral nervous system in modulating immunity against cancer. By understanding this neuro-immune axis, novel therapeutic approaches may be uncovered to strengthen antitumour immunity and enhance responses to existing cancer treatments. | The nervous and immune systems communicate with each other, and this interaction, known as neuro-immunity, plays a crucial role in cancer. Nerves within tumors can release signals that affect the behavior of immune cells and promote tumor growth. Conversely, immune cells can also modify the activity of these nerves. This complex communication can suppress the body's natural anti-tumor immune response. By better understanding this neuro-immune axis, scientists hope to discover new ways to improve cancer treatments. | The body's nervous and immune systems, which typically work together to fight off threats and heal injuries, also have a significant impact on cancer. This is because nerves can grow into tumors and release signals that affect how immune cells behave and how the tumor grows. For example, some nerve signals can weaken the immune system's ability to fight the cancer. On the other hand, immune cells within the tumor can also influence the nerves, including those that transmit pain. Understanding this complex relationship between nerves, immune cells, and cancer cells could lead to new treatments that boost the immune system's ability to attack tumors. | The abstract discusses how the nervous system and the immune system work together to influence cancer growth. Nerves within tumors can release substances that change how immune cells behave and affect how the tumor progresses. Similarly, immune cells in the tumor can alter the activity of local nerves. Additionally, the nervous system's sympathetic signals can weaken the immune response against cancer. Understanding this connection, called the neuro-immune axis, could lead to new ways to treat cancer and improve current therapies. |
| NatRevCan | 4 | 10.1038/s41568-025-00831-w | DeepSeek | The nervous and immune systems have co-evolved to detect and respond to internal and external threats, working together to restore homeostasis after tissue injury or infection. Sharing several receptors and ligands, they engage in direct cross-talk that substantially influences disease development. The emerging field of cancer neuro-immunity focuses on the intricate interactions between the nervous system, immune responses and tumour growth. Additional findings have revealed that nerve fibres infiltrating peripheral tumours can release neuromodulatory factors that shape both immune cell behaviour and tumour progression. Conversely, tumour-infiltrating immune cells can modify the activity of local neurons, including pain-transmitting nociceptive sensory neurons. Beyond sensory fibres, sympathetic signalling can foster immunosuppression by recruiting myeloid-derived suppressor cells and promoting T cell exhaustion. This Review summarizes current evidence on how neuronal signalling regulates peripheral antitumour immune responses within the tumour microenvironment. We describe the complex, reciprocal interactions among neurons, immune cells and malignant cells, highlighting the key parts played by the peripheral nervous system in modulating immunity against cancer. By understanding this neuro-immune axis, novel therapeutic approaches may be uncovered to strengthen antitumour immunity and enhance responses to existing cancer treatments. | The nervous and immune systems work together to respond to threats and heal injuries, but in cancer, their interaction can sometimes help tumors grow. Nerves in and around tumors release signals that affect immune cells, weakening their ability to fight cancer. At the same time, immune cells can change nerve activity, sometimes causing pain. This review explains how nerves and immune cells communicate in cancer, creating an environment that supports tumor growth. Understanding this connection could lead to new treatments that boost the immune system’s ability to fight cancer. | The nervous and immune systems work together to protect the body, but in cancer, their interaction can sometimes help tumors grow. Nerves in and around tumors release signals that affect immune cells, weakening their ability to fight cancer. At the same time, immune cells can change nerve activity, influencing pain and tumor spread. This review explains how nerve signals control immune responses in cancer and explores how blocking harmful nerve-immune interactions could lead to new treatments. | This review explores how the nervous and immune systems interact in cancer. Nerves in and around tumors release signals that can affect immune cells and tumor growth, while immune cells can also change nerve activity, sometimes causing pain. For example, stress-related nerves can weaken the immune response, helping tumors escape detection. Understanding these connections could lead to new treatments to boost the immune system’s ability to fight cancer. |
| NatRevCan | 4 | 10.1038/s41568-025-00831-w | Grok | The nervous and immune systems have co-evolved to detect and respond to internal and external threats, working together to restore homeostasis after tissue injury or infection. Sharing several receptors and ligands, they engage in direct cross-talk that substantially influences disease development. The emerging field of cancer neuro-immunity focuses on the intricate interactions between the nervous system, immune responses and tumour growth. Additional findings have revealed that nerve fibres infiltrating peripheral tumours can release neuromodulatory factors that shape both immune cell behaviour and tumour progression. Conversely, tumour-infiltrating immune cells can modify the activity of local neurons, including pain-transmitting nociceptive sensory neurons. Beyond sensory fibres, sympathetic signalling can foster immunosuppression by recruiting myeloid-derived suppressor cells and promoting T cell exhaustion. This Review summarizes current evidence on how neuronal signalling regulates peripheral antitumour immune responses within the tumour microenvironment. We describe the complex, reciprocal interactions among neurons, immune cells and malignant cells, highlighting the key parts played by the peripheral nervous system in modulating immunity against cancer. By understanding this neuro-immune axis, novel therapeutic approaches may be uncovered to strengthen antitumour immunity and enhance responses to existing cancer treatments. | The nervous and immune systems work together to protect the body, and their interactions play a big role in cancer development. In the tumor environment, nerve fibers release chemicals that affect immune cells and tumor growth, while immune cells can influence nerve activity, including pain signals. For example, signals from the nervous system can weaken the immune response by attracting cells that suppress immunity and exhausting T cells. This review explains how these complex interactions between nerves, immune cells, and cancer cells shape the body's fight against cancer, suggesting that understanding these connections could lead to new ways to boost cancer treatments. | The nervous and immune systems work together to protect the body and maintain balance, especially in response to injury or infection, and they share many communication signals. In cancer, the new field of cancer neuro-immunity explores how nerve fibers in tumors release chemicals that affect immune cells and tumor growth, while immune cells can influence nerve activity, including pain-sensing nerves. For example, signals from the nervous system can weaken the immune response by attracting cells that suppress immunity or exhausting T cells. This review explains these complex interactions between nerves, immune cells, and cancer cells, suggesting that understanding these connections could lead to new ways to boost the immune system’s fight against cancer and improve existing treatments. | The nervous and immune systems work together to protect the body and maintain balance after injury or infection, and their interactions play a big role in cancer development. In the tumor microenvironment, nerve fibers release signals that affect immune cells and tumor growth, while immune cells can influence nerve activity, including pain-sensing neurons. Sympathetic nerve signals may weaken the immune response by attracting certain cells and exhausting others. Understanding these connections between nerves, immune cells, and cancer cells could lead to new ways to boost the body’s ability to fight cancer and improve existing treatments. |
| NatRevCan | 5 | 10.1038/s41568-025-00829-5 | Open AI | Sex is a significant determinant of cancer incidence and outcome. The effects of sexual differentiation on normal and cancer biology underly this epidemiology. The resultant sex differences in therapeutic target pathways and processes provide a foundation for developing more personalized cancer treatments. However, our efforts at personalization cannot stop there. Humans also have gender, and sex and gender are highly interactive in individuation. Thus, we will also need to consider how gender-sex interactions (GSI) affect cancer biology and clinical parameters such as the timing of diagnoses, clinical trial enrolment, and the completeness of efficacy and toxicity data. Ignoring the effects of GSI can compromise the quality of basic biological and clinical data and the conclusions drawn from them. This is not to say that GSI will always have a significant effect or any effect at all in every cancer study. Rather, it is to say that we know enough about GSI and human cancer to anticipate measurable differences when GSI are considered in research, enabling us to experimentally determine whether their effects are significant. Here, I delve deeply into GSI and cancer, as this approach to treatment personalization holds great promise to benefit all patients with cancer. | Sex plays an important role in how often cancer occurs and how it progresses, because biological differences between males and females affect cancer development and treatment responses. But beyond biological sex, a person’s gender and how it interacts with sex also influence cancer diagnosis, treatment, and research outcomes. Paying attention to these gender-sex interactions can improve the accuracy of cancer studies and help create more personalized and effective treatments for everyone. | Sex plays a big role in how often cancer happens and how it progresses, because male and female biology affects cancer differently. Understanding these differences helps create more personalized treatments. But it’s also important to consider gender—how people identify and live—as it interacts with sex in complex ways that can affect cancer diagnosis, treatment, and research results. Ignoring these sex-gender interactions can lead to less accurate data and conclusions. Paying attention to both sex and gender together can improve cancer care for everyone. | Sex plays a big role in how often people get cancer and how they respond to treatment, because biological differences affect cancer development and therapy. But it’s not just sex—gender, which interacts closely with sex, also influences cancer in important ways, like when cancer is diagnosed and how patients respond to treatments. Paying attention to how sex and gender work together can help improve cancer research and lead to better, more personalized care for everyone. |
| NatRevCan | 5 | 10.1038/s41568-025-00829-6 | Claude | Sex is a significant determinant of cancer incidence and outcome. The effects of sexual differentiation on normal and cancer biology underly this epidemiology. The resultant sex differences in therapeutic target pathways and processes provide a foundation for developing more personalized cancer treatments. However, our efforts at personalization cannot stop there. Humans also have gender, and sex and gender are highly interactive in individuation. Thus, we will also need to consider how gender-sex interactions (GSI) affect cancer biology and clinical parameters such as the timing of diagnoses, clinical trial enrolment, and the completeness of efficacy and toxicity data. Ignoring the effects of GSI can compromise the quality of basic biological and clinical data and the conclusions drawn from them. This is not to say that GSI will always have a significant effect or any effect at all in every cancer study. Rather, it is to say that we know enough about GSI and human cancer to anticipate measurable differences when GSI are considered in research, enabling us to experimentally determine whether their effects are significant. Here, I delve deeply into GSI and cancer, as this approach to treatment personalization holds great promise to benefit all patients with cancer. | This text explains that both biological sex (male/female) and gender identity significantly affect how cancer develops, progresses, and responds to treatment. While researchers already know that men and women can experience cancer differently, the author argues that we also need to study how sex and gender work together to influence cancer outcomes. This includes looking at factors like when people get diagnosed, who participates in clinical trials, and how treatments work for different groups. By better understanding these combined sex-gender effects, doctors could create more personalized and effective cancer treatments for all patients. | This text explains that both biological sex (male/female) and gender (social identity) work together to influence how cancer develops, progresses, and responds to treatment. While doctors already know that men and women can have different cancer experiences, the author argues that researchers need to also study how gender and sex interact with each other to affect things like when people get diagnosed, whether they join clinical trials, and how well treatments work. By understanding these combined effects, doctors could develop better personalized cancer treatments that help all patients more effectively. | This text explains that both biological sex (male/female) and gender identity significantly affect how cancer develops and how well treatments work. While doctors are starting to create more personalized cancer treatments based on biological differences between men and women, they also need to consider how sex and gender work together to influence things like when people get diagnosed, who participates in medical studies, and how well treatments actually work. The author argues that ignoring these combined sex-gender effects can lead to incomplete or misleading research results, and that studying these interactions more carefully could help improve cancer treatment for everyone. |
| NatRevCan | 5 | 10.1038/s41568-025-00829-7 | Gemini | Sex is a significant determinant of cancer incidence and outcome. The effects of sexual differentiation on normal and cancer biology underly this epidemiology. The resultant sex differences in therapeutic target pathways and processes provide a foundation for developing more personalized cancer treatments. However, our efforts at personalization cannot stop there. Humans also have gender, and sex and gender are highly interactive in individuation. Thus, we will also need to consider how gender-sex interactions (GSI) affect cancer biology and clinical parameters such as the timing of diagnoses, clinical trial enrolment, and the completeness of efficacy and toxicity data. Ignoring the effects of GSI can compromise the quality of basic biological and clinical data and the conclusions drawn from them. This is not to say that GSI will always have a significant effect or any effect at all in every cancer study. Rather, it is to say that we know enough about GSI and human cancer to anticipate measurable differences when GSI are considered in research, enabling us to experimentally determine whether their effects are significant. Here, I delve deeply into GSI and cancer, as this approach to treatment personalization holds great promise to benefit all patients with cancer. | Sex and gender both play a significant role in how cancer develops and how patients respond to treatment. The article explains that while biological sex differences are important, we also need to consider gender—a person's social and cultural identity—because of how these two factors interact. These gender-sex interactions can affect the biology of cancer as well as clinical aspects, like the timing of a diagnosis and participation in clinical trials. By studying these interactions, researchers believe they can develop more effective, personalized cancer treatments for everyone. | The text explains that both a person's biological sex and their social gender can impact cancer. It highlights that sex-based differences in biology affect how cancer develops and how patients respond to treatment. The author argues that simply considering sex isn't enough. We also need to understand how gender—the social and behavioral aspects—interacts with biological sex. These interactions, referred to as GSI, can influence things like when people are diagnosed, who enrolls in clinical trials, and the quality of data collected. By studying these interactions, we can create more personalized and effective cancer treatments for everyone. | Sex and gender both play a significant role in how cancer develops and how patients respond to treatment. The article explains that while biological sex differences are important, we also need to consider gender—a person's social and cultural identity—because of how these two factors interact. These gender-sex interactions can affect the biology of cancer as well as clinical aspects, like the timing of a diagnosis and participation in clinical trials. By studying these interactions, researchers believe they can develop more effective, personalized cancer treatments for everyone. |
| NatRevCan | 5 | 10.1038/s41568-025-00829-8 | DeepSeek | Sex is a significant determinant of cancer incidence and outcome. The effects of sexual differentiation on normal and cancer biology underly this epidemiology. The resultant sex differences in therapeutic target pathways and processes provide a foundation for developing more personalized cancer treatments. However, our efforts at personalization cannot stop there. Humans also have gender, and sex and gender are highly interactive in individuation. Thus, we will also need to consider how gender-sex interactions (GSI) affect cancer biology and clinical parameters such as the timing of diagnoses, clinical trial enrolment, and the completeness of efficacy and toxicity data. Ignoring the effects of GSI can compromise the quality of basic biological and clinical data and the conclusions drawn from them. This is not to say that GSI will always have a significant effect or any effect at all in every cancer study. Rather, it is to say that we know enough about GSI and human cancer to anticipate measurable differences when GSI are considered in research, enabling us to experimentally determine whether their effects are significant. Here, I delve deeply into GSI and cancer, as this approach to treatment personalization holds great promise to benefit all patients with cancer. | This text explains that both biological sex (like male or female) and gender (social and cultural roles) affect cancer risk, treatment response, and outcomes. While sex influences cancer biology, gender can impact factors like diagnosis timing and treatment access. The interaction between sex and gender (GSI) is important to consider in cancer research and care to improve personalized treatments and ensure better results for all patients. Ignoring these differences could lead to incomplete or biased data, so studying GSI helps make cancer research more accurate and effective. | This text explains that both biological sex (like male or female) and gender (social and cultural roles) influence cancer risk, treatment, and outcomes. Sex affects how cancer develops and responds to therapies, while gender can impact diagnosis timing, clinical trial participation, and treatment side effects. The author argues that studying how sex and gender interact can improve personalized cancer care, leading to better results for all patients. Ignoring these differences could harm research quality and patient outcomes. While not every study will find major effects, understanding these interactions can help tailor more effective treatments. | This text explains that both biological sex (like male or female) and gender (social and cultural roles) influence cancer risk, progression, and treatment outcomes. While sex affects cancer biology, gender can impact diagnosis timing, treatment access, and side effects. Ignoring these differences can lead to incomplete or biased research and care. By studying how sex and gender interact, scientists can develop more personalized and effective cancer treatments for everyone. The author argues that understanding these interactions is crucial for better cancer research and patient care. |
| NatRevCan | 5 | 10.1038/s41568-025-00829-9 | Grok | Sex is a significant determinant of cancer incidence and outcome. The effects of sexual differentiation on normal and cancer biology underly this epidemiology. The resultant sex differences in therapeutic target pathways and processes provide a foundation for developing more personalized cancer treatments. However, our efforts at personalization cannot stop there. Humans also have gender, and sex and gender are highly interactive in individuation. Thus, we will also need to consider how gender-sex interactions (GSI) affect cancer biology and clinical parameters such as the timing of diagnoses, clinical trial enrolment, and the completeness of efficacy and toxicity data. Ignoring the effects of GSI can compromise the quality of basic biological and clinical data and the conclusions drawn from them. This is not to say that GSI will always have a significant effect or any effect at all in every cancer study. Rather, it is to say that we know enough about GSI and human cancer to anticipate measurable differences when GSI are considered in research, enabling us to experimentally determine whether their effects are significant. Here, I delve deeply into GSI and cancer, as this approach to treatment personalization holds great promise to benefit all patients with cancer. | Sex and gender play important roles in how cancer develops and how it affects people. Biological differences between males and females, combined with societal gender influences, impact cancer rates, treatment responses, and outcomes. Considering these sex and gender interactions can help create more personalized cancer treatments and improve research by accounting for their effects on diagnosis, clinical trials, and treatment data. While not always significant, exploring these factors can lead to better care for all cancer patients. | Sex and gender significantly influence cancer development and treatment outcomes due to biological differences and their interactions in individuals. These differences affect cancer rates, treatment pathways, and clinical factors like diagnosis timing and trial participation. Considering how sex and gender interact can improve the quality of cancer research and lead to more personalized treatments, though their impact may vary across studies. Exploring these interactions offers a promising approach to better cancer care for everyone. | Sex differences play a big role in how often cancer occurs and its outcomes, stemming from biological factors that influence treatment targets and processes for more tailored care. However, people also have gender identities, which interact with sex to shape individual experiences, affecting things like when cancer is diagnosed, who joins clinical trials, and the accuracy of data on drug effectiveness and side effects. Overlooking these gender-sex interactions can weaken research quality, but by studying them, we can better understand their impact and improve cancer treatments for everyone. |
| CancerRes | 1 | 10.1158/0008-5472.CAN-24-3174 | Open AI | Perineural invasion (PNI) is a pivotal prognostic factor in pancreatic cancer, associated with aggressive tumor behavior and adverse patient outcomes. The recognized clinical impact of PNI highlights the need to better understand the molecular mechanisms underlying PNI-induced phenotypes. In this study, we isolated PNI-associated cancer-associated fibroblasts (pCAF), which demonstrated a markedly enhanced capacity to promote neural invasion in pancreatic cancer compared with non-PNI-associated CAFs. Single-cell, high-throughput sequencing and metabolomics data showed a significant upregulation of glycolysis in pCAFs, fostering a high-lactate tumor microenvironment conducive to cancer progression. pCAF-derived lactate was absorbed by tumor cells, facilitating histone H3K18 lactylation. The lactate-induced epigenetic modification activated the transcription of neural invasion-associated genes, such as L1CAM and SLIT1, thereby driving PNI in pancreatic cancer. Further exploration of metabolic reprogramming in pCAFs revealed enhanced acetylation of the glycolytic enzyme GAPDH, which correlated with increased enzymatic activity and glycolytic flux. Targeting GAPDH and lactylation modifications significantly inhibited neural invasion in a genetically engineered mouse model. Clinical data suggested that high levels of H3K18 lactylation correlate with severe PNI and poorer patient prognosis. Together, these findings provide critical insights into the role of CAFs in promoting PNI of pancreatic cancer, highlighting glycolytic reprogramming and lactate-driven histone modifications as potential therapeutic targets for PDAC.Significance: Targeting cancer-associated fibroblast metabolism or histone lactylation in pancreatic cancer cells to reverse epigenetic remodeling induced by lactate accumulation in the tumor microenvironment are potential therapeutic strategies to inhibit perineural invasion. | Perineural invasion (PNI) in pancreatic cancer makes the disease more aggressive and harder to treat. This study found that special cells called cancer-associated fibroblasts (pCAFs) near nerves produce lots of lactate through increased glycolysis. The lactate changes how tumor genes behave by modifying histones, which helps cancer spread along nerves. Blocking the enzyme GAPDH or the lactate-related changes slowed down this nerve invasion in mice. These discoveries suggest new ways to treat pancreatic cancer by targeting these metabolic and gene changes in the tumor environment. | Perineural invasion (PNI) in pancreatic cancer is linked to worse outcomes because cancer-associated fibroblasts (pCAFs) near nerves help tumors grow and invade by producing lots of lactate through increased sugar metabolism. This lactate changes how cancer cells turn on certain genes that promote nerve invasion. By blocking key enzymes and these lactate-driven changes, researchers were able to reduce nerve invasion in mice, suggesting new treatments could focus on stopping these metabolic and gene changes to help patients. | In pancreatic cancer, a harmful process called perineural invasion (PNI) helps tumors spread and worsen patient outcomes. This study found that special cells called cancer-associated fibroblasts (pCAFs) near nerves produce lots of lactate through increased glycolysis. This lactate changes tumor cells by modifying their genes, making them more likely to invade nerves. Blocking key enzymes involved in this process reduced nerve invasion in mice. These findings suggest that targeting how these fibroblasts produce energy or how lactate changes tumor cells could be a new way to treat pancreatic cancer and stop it from spreading along nerves. |
| CancerRes | 1 | 10.1158/0008-5472.CAN-24-3175 | Claude | Perineural invasion (PNI) is a pivotal prognostic factor in pancreatic cancer, associated with aggressive tumor behavior and adverse patient outcomes. The recognized clinical impact of PNI highlights the need to better understand the molecular mechanisms underlying PNI-induced phenotypes. In this study, we isolated PNI-associated cancer-associated fibroblasts (pCAF), which demonstrated a markedly enhanced capacity to promote neural invasion in pancreatic cancer compared with non-PNI-associated CAFs. Single-cell, high-throughput sequencing and metabolomics data showed a significant upregulation of glycolysis in pCAFs, fostering a high-lactate tumor microenvironment conducive to cancer progression. pCAF-derived lactate was absorbed by tumor cells, facilitating histone H3K18 lactylation. The lactate-induced epigenetic modification activated the transcription of neural invasion-associated genes, such as L1CAM and SLIT1, thereby driving PNI in pancreatic cancer. Further exploration of metabolic reprogramming in pCAFs revealed enhanced acetylation of the glycolytic enzyme GAPDH, which correlated with increased enzymatic activity and glycolytic flux. Targeting GAPDH and lactylation modifications significantly inhibited neural invasion in a genetically engineered mouse model. Clinical data suggested that high levels of H3K18 lactylation correlate with severe PNI and poorer patient prognosis. Together, these findings provide critical insights into the role of CAFs in promoting PNI of pancreatic cancer, highlighting glycolytic reprogramming and lactate-driven histone modifications as potential therapeutic targets for PDAC.Significance: Targeting cancer-associated fibroblast metabolism or histone lactylation in pancreatic cancer cells to reverse epigenetic remodeling induced by lactate accumulation in the tumor microenvironment are potential therapeutic strategies to inhibit perineural invasion. | This study looked at how pancreatic cancer spreads along nerves, which leads to worse outcomes for patients. Researchers found that certain cells called cancer-associated fibroblasts (CAFs) near nerves produce high amounts of lactate, a sugar byproduct. Cancer cells absorb this lactate, which changes how their genes work by modifying proteins called histones. These genetic changes turn on genes that help cancer spread along nerves. The researchers discovered they could block this nerve invasion in lab mice by targeting either the sugar-processing enzymes in CAFs or the histone modifications in cancer cells. Patients with high levels of these histone changes had worse disease progression, suggesting that targeting this lactate-driven process could be a new way to treat pancreatic cancer and prevent it from spreading along nerves. | This research found that certain support cells called cancer-associated fibroblasts (CAFs) near nerves in pancreatic cancer tumors help cancer spread along nerve pathways, which makes the disease more aggressive. These special CAFs produce high amounts of a sugar-breakdown product called lactate, which cancer cells absorb and use to turn on genes that help them invade nerves. The study showed that blocking either the sugar breakdown process in these support cells or the way cancer cells use lactate could prevent nerve invasion in lab models. Patients with high levels of this lactate-driven gene activation had worse outcomes, suggesting these processes could be targeted for new pancreatic cancer treatments. | Researchers found that in pancreatic cancer, special cells called cancer-associated fibroblasts help cancer spread along nerves, which makes the disease more aggressive and harder to treat. These fibroblasts produce high amounts of a sugar byproduct called lactate, which cancer cells then absorb and use to turn on genes that help them invade nerves. The study showed that blocking either the production of lactate or preventing cancer cells from using it could stop this nerve invasion in laboratory models. This discovery suggests new ways to treat pancreatic cancer by targeting these metabolic processes, potentially improving outcomes for patients whose cancer spreads along nerve pathways. |
| CancerRes | 1 | 10.1158/0008-5472.CAN-24-3176 | Gemini | Perineural invasion (PNI) is a pivotal prognostic factor in pancreatic cancer, associated with aggressive tumor behavior and adverse patient outcomes. The recognized clinical impact of PNI highlights the need to better understand the molecular mechanisms underlying PNI-induced phenotypes. In this study, we isolated PNI-associated cancer-associated fibroblasts (pCAF), which demonstrated a markedly enhanced capacity to promote neural invasion in pancreatic cancer compared with non-PNI-associated CAFs. Single-cell, high-throughput sequencing and metabolomics data showed a significant upregulation of glycolysis in pCAFs, fostering a high-lactate tumor microenvironment conducive to cancer progression. pCAF-derived lactate was absorbed by tumor cells, facilitating histone H3K18 lactylation. The lactate-induced epigenetic modification activated the transcription of neural invasion-associated genes, such as L1CAM and SLIT1, thereby driving PNI in pancreatic cancer. Further exploration of metabolic reprogramming in pCAFs revealed enhanced acetylation of the glycolytic enzyme GAPDH, which correlated with increased enzymatic activity and glycolytic flux. Targeting GAPDH and lactylation modifications significantly inhibited neural invasion in a genetically engineered mouse model. Clinical data suggested that high levels of H3K18 lactylation correlate with severe PNI and poorer patient prognosis. Together, these findings provide critical insights into the role of CAFs in promoting PNI of pancreatic cancer, highlighting glycolytic reprogramming and lactate-driven histone modifications as potential therapeutic targets for PDAC.Significance: Targeting cancer-associated fibroblast metabolism or histone lactylation in pancreatic cancer cells to reverse epigenetic remodeling induced by lactate accumulation in the tumor microenvironment are potential therapeutic strategies to inhibit perineural invasion. | In simple terms, this study found that special cells called cancer-associated fibroblasts (CAFs) help pancreatic cancer spread to nerves, a process called perineural invasion (PNI). These specific CAFs, found near the nerves, have a high-energy metabolism that produces a lot of lactate. This lactate is then absorbed by the cancer cells, where it changes their DNA by a process called lactylation. This change in the cancer cells' DNA turns on genes that specifically help the cancer invade nerves. Blocking this process, either by targeting the CAFs' metabolism or by stopping the lactylation in the cancer cells, could be a new way to treat pancreatic cancer and prevent its aggressive spread. | Cancer-associated fibroblasts, or CAFs, that are near nerves in pancreatic cancer have a metabolism that's very different from other CAFs. These specialized CAFs, called pCAFs, produce a lot of lactate, creating a high-lactate environment around the tumor. The cancer cells then absorb this lactate, which causes a change called lactylation to occur on their histones. This process acts like a switch, turning on genes that help cancer cells invade nerves. Since this nerve invasion leads to a more aggressive tumor, targeting the lactate production in the CAFs or the lactylation process in the cancer cells could be a new way to treat pancreatic cancer. | Cancer-associated fibroblasts (CAFs) are a type of cell that can help tumors grow. A specific kind of CAF, found near nerves, called pCAFs, helps pancreatic cancer spread to nerves. These pCAFs switch their metabolism to produce a lot of lactate, which is a type of lactic acid. This lactate is then absorbed by the cancer cells and changes their epigenetic markers—essentially turning on genes that make the cancer more aggressive and able to invade nerves. The study found that blocking this process, either by targeting the metabolism of the pCAFs or the changes in the cancer cells, can stop this nerve invasion. This suggests that these pathways could be new targets for treating pancreatic cancer. |
| CancerRes | 1 | 10.1158/0008-5472.CAN-24-3177 | DeepSeek | Perineural invasion (PNI) is a pivotal prognostic factor in pancreatic cancer, associated with aggressive tumor behavior and adverse patient outcomes. The recognized clinical impact of PNI highlights the need to better understand the molecular mechanisms underlying PNI-induced phenotypes. In this study, we isolated PNI-associated cancer-associated fibroblasts (pCAF), which demonstrated a markedly enhanced capacity to promote neural invasion in pancreatic cancer compared with non-PNI-associated CAFs. Single-cell, high-throughput sequencing and metabolomics data showed a significant upregulation of glycolysis in pCAFs, fostering a high-lactate tumor microenvironment conducive to cancer progression. pCAF-derived lactate was absorbed by tumor cells, facilitating histone H3K18 lactylation. The lactate-induced epigenetic modification activated the transcription of neural invasion-associated genes, such as L1CAM and SLIT1, thereby driving PNI in pancreatic cancer. Further exploration of metabolic reprogramming in pCAFs revealed enhanced acetylation of the glycolytic enzyme GAPDH, which correlated with increased enzymatic activity and glycolytic flux. Targeting GAPDH and lactylation modifications significantly inhibited neural invasion in a genetically engineered mouse model. Clinical data suggested that high levels of H3K18 lactylation correlate with severe PNI and poorer patient prognosis. Together, these findings provide critical insights into the role of CAFs in promoting PNI of pancreatic cancer, highlighting glycolytic reprogramming and lactate-driven histone modifications as potential therapeutic targets for PDAC.Significance: Targeting cancer-associated fibroblast metabolism or histone lactylation in pancreatic cancer cells to reverse epigenetic remodeling induced by lactate accumulation in the tumor microenvironment are potential therapeutic strategies to inhibit perineural invasion. | This study found that certain cancer-associated fibroblasts (pCAFs) in pancreatic cancer produce high levels of lactate, which creates an environment that helps cancer spread along nerves (perineural invasion, PNI). The lactate is taken up by cancer cells, causing epigenetic changes that activate genes linked to nerve invasion, like L1CAM and SLIT1. The pCAFs boost lactate production by increasing glycolysis, partly through modifications to the enzyme GAPDH. Blocking GAPDH or lactylation reduced nerve invasion in mice, and high lactylation levels in patients were linked to worse outcomes. The findings suggest that targeting fibroblast metabolism or lactate-driven epigenetic changes could be new ways to treat pancreatic cancer. | This study found that certain cancer-associated fibroblasts (pCAFs) in pancreatic cancer create a high-lactate environment, which helps cancer cells invade nearby nerves—a process called perineural invasion (PNI). The lactate from pCAFs is taken up by cancer cells, causing epigenetic changes that activate genes linked to nerve invasion. The researchers also discovered that pCAFs boost lactate production by increasing glycolysis, driven by modifications to the enzyme GAPDH. Blocking GAPDH or histone lactylation reduced nerve invasion in mice, and high levels of these changes in patients were linked to worse outcomes. The findings suggest that targeting pCAF metabolism or lactate-induced epigenetic changes could be new ways to treat aggressive pancreatic cancer. | This study found that certain fibroblasts (pCAFs) in pancreatic cancer create a high-lactate environment, which helps cancer cells invade nerves—a process linked to worse outcomes. The lactate from pCAFs is taken up by cancer cells, causing epigenetic changes that activate genes driving nerve invasion. Blocking lactate production or its effects reduced nerve invasion in mice, and high lactate levels in patients were tied to more aggressive cancer. Targeting these metabolic and epigenetic pathways could lead to new treatments for pancreatic cancer. |
| CancerRes | 1 | 10.1158/0008-5472.CAN-24-3178 | Grok | Perineural invasion (PNI) is a pivotal prognostic factor in pancreatic cancer, associated with aggressive tumor behavior and adverse patient outcomes. The recognized clinical impact of PNI highlights the need to better understand the molecular mechanisms underlying PNI-induced phenotypes. In this study, we isolated PNI-associated cancer-associated fibroblasts (pCAF), which demonstrated a markedly enhanced capacity to promote neural invasion in pancreatic cancer compared with non-PNI-associated CAFs. Single-cell, high-throughput sequencing and metabolomics data showed a significant upregulation of glycolysis in pCAFs, fostering a high-lactate tumor microenvironment conducive to cancer progression. pCAF-derived lactate was absorbed by tumor cells, facilitating histone H3K18 lactylation. The lactate-induced epigenetic modification activated the transcription of neural invasion-associated genes, such as L1CAM and SLIT1, thereby driving PNI in pancreatic cancer. Further exploration of metabolic reprogramming in pCAFs revealed enhanced acetylation of the glycolytic enzyme GAPDH, which correlated with increased enzymatic activity and glycolytic flux. Targeting GAPDH and lactylation modifications significantly inhibited neural invasion in a genetically engineered mouse model. Clinical data suggested that high levels of H3K18 lactylation correlate with severe PNI and poorer patient prognosis. Together, these findings provide critical insights into the role of CAFs in promoting PNI of pancreatic cancer, highlighting glycolytic reprogramming and lactate-driven histone modifications as potential therapeutic targets for PDAC.Significance: Targeting cancer-associated fibroblast metabolism or histone lactylation in pancreatic cancer cells to reverse epigenetic remodeling induced by lactate accumulation in the tumor microenvironment are potential therapeutic strategies to inhibit perineural invasion. | In pancreatic cancer, cancer-associated fibroblasts (pCAFs) linked to perineural invasion (PNI) create a high-lactate environment by boosting glycolysis, which promotes aggressive cancer spread along nerves. These pCAFs produce lactate that tumor cells use to modify histones (H3K18 lactylation), activating genes like L1CAM and SLIT1 that drive PNI. Enhanced activity of the enzyme GAPDH in pCAFs further increases this lactate production. Studies in mice showed that targeting GAPDH or histone lactylation reduced PNI, and high H3K18 lactylation in patients was linked to worse outcomes, suggesting these processes as potential treatment targets. | In pancreatic cancer, cancer-associated fibroblasts (pCAFs) linked to perineural invasion (PNI), where cancer spreads along nerves, create a high-lactate environment by boosting glycolysis. This lactate is taken up by tumor cells, triggering a modification called histone H3K18 lactylation, which turns on genes like L1CAM and SLIT1 that drive nerve invasion. The study found that pCAFs enhance the activity of a glycolytic enzyme, GAPDH, increasing lactate production. Blocking GAPDH or lactylation reduced nerve invasion in mice, and high H3K18 lactylation levels in patients were linked to worse PNI and outcomes, suggesting new treatment targets to stop cancer spread. | In pancreatic cancer, cancer-associated fibroblasts (pCAFs) linked to perineural invasion (PNI), where cancer spreads along nerves, create a high-lactate environment by boosting glycolysis. This lactate is taken up by cancer cells, triggering a process called histone H3K18 lactylation that turns on genes like L1CAM and SLIT1, which drive nerve invasion. The study found that targeting a key enzyme, GAPDH, or the lactylation process in pCAFs reduced nerve invasion in mice and could be a promising treatment strategy, as high H3K18 lactylation levels in patients were linked to worse outcomes. |
| CancerRes | 2 | 10.1158/0008-5472.CAN-24-1880 | Open AI | Black individuals experience worse survival after a diagnosis of high-grade serous ovarian carcinoma (HGSC) than White individuals and are underrepresented in ovarian cancer research. To date, the understanding of the molecular and genomic heterogeneity of HGSC is based primarily on the evaluation of tumors from White individuals. In the present study, we performed whole-exome sequencing on HGSC samples from 211 Black patients to identify significantly mutated genes and characterize mutational signatures, assessing their distributions by gene expression subtypes. The occurrence and frequency of somatic mutations and signatures by self-reported race were compared with historic data from The Cancer Genome Atlas (TCGA). Despite technical differences (e.g., formalin-fixed vs. fresh-frozen tissue), the distribution of mutations and their variant classifications for major HGSC genes were nearly identical across study populations. However, de novo significantly mutated gene analysis identified genes not previously reported in TCGA analysis, including the oncogene KRAS and the potential tumor suppressor OBSCN. The prevalence of the homologous recombination deficiency signature was higher among Black individuals with the immunoreactive gene expression subtype compared with the mesenchymal and proliferative subtypes. These findings were confirmed by comparing the data from Black patients with those from 123 White patients with identical tissue collection and processing. Overall, this study suggests that, although most features of HGSC tumor phenotypes are similar in Black and White populations, there may be clinically relevant differences. If validated, these phenotypes may be important for clinical decision-making and would have been missed by characterizing tumors from White individuals only.Significance: Elucidation of the somatic mutational landscape of high-grade serous ovarian carcinoma in Black individuals, who experience poor survival and are underrepresented in research, could inform patient prognosis and enable precision medicine opportunities. | Black women with high-grade serous ovarian cancer (HGSC) tend to have worse survival rates but are rarely included in research. This study analyzed tumors from 211 Black patients and found that while many genetic features of the cancer are similar between Black and White patients, some important differences exist, including unique gene mutations and certain mutation patterns. These differences could affect how the cancer behaves and responds to treatment. Understanding these unique traits in Black patients may help improve their care and lead to more personalized treatments. | Black women with high-grade serous ovarian cancer tend to have worse survival than White women, but most research has focused on tumors from White patients. This study analyzed tumors from Black patients and found that while many genetic features are similar between Black and White patients, there are some important differences in certain mutated genes and mutation patterns. These differences might affect treatment and outcomes, showing the need to include more diverse groups in research to improve care for everyone. | Black individuals with high-grade serous ovarian cancer (HGSC) tend to have worse survival rates than White individuals but have been underrepresented in research. This study analyzed tumors from Black patients to understand their genetic differences and found some unique gene changes not seen in White patients. While many tumor features were similar between Black and White groups, certain genetic differences might affect treatment and outcomes. Studying tumors from Black patients is important to improve care and develop more precise treatments for everyone. |
| CancerRes | 2 | 10.1158/0008-5472.CAN-24-1881 | Claude | Black individuals experience worse survival after a diagnosis of high-grade serous ovarian carcinoma (HGSC) than White individuals and are underrepresented in ovarian cancer research. To date, the understanding of the molecular and genomic heterogeneity of HGSC is based primarily on the evaluation of tumors from White individuals. In the present study, we performed whole-exome sequencing on HGSC samples from 211 Black patients to identify significantly mutated genes and characterize mutational signatures, assessing their distributions by gene expression subtypes. The occurrence and frequency of somatic mutations and signatures by self-reported race were compared with historic data from The Cancer Genome Atlas (TCGA). Despite technical differences (e.g., formalin-fixed vs. fresh-frozen tissue), the distribution of mutations and their variant classifications for major HGSC genes were nearly identical across study populations. However, de novo significantly mutated gene analysis identified genes not previously reported in TCGA analysis, including the oncogene KRAS and the potential tumor suppressor OBSCN. The prevalence of the homologous recombination deficiency signature was higher among Black individuals with the immunoreactive gene expression subtype compared with the mesenchymal and proliferative subtypes. These findings were confirmed by comparing the data from Black patients with those from 123 White patients with identical tissue collection and processing. Overall, this study suggests that, although most features of HGSC tumor phenotypes are similar in Black and White populations, there may be clinically relevant differences. If validated, these phenotypes may be important for clinical decision-making and would have been missed by characterizing tumors from White individuals only.Significance: Elucidation of the somatic mutational landscape of high-grade serous ovarian carcinoma in Black individuals, who experience poor survival and are underrepresented in research, could inform patient prognosis and enable precision medicine opportunities. | This study looked at genetic differences in a deadly type of ovarian cancer between Black and White patients. Researchers analyzed tumor samples from 211 Black patients and found that while most genetic features of the cancer were similar between racial groups, there were some important differences. Black patients had mutations in certain genes (like KRAS) that weren't commonly found in previous studies that mostly included White patients. They also found that Black patients with one particular type of tumor showed more signs of DNA repair problems. These findings suggest that studying cancer genetics in diverse populations could help doctors make better treatment decisions, since relying only on data from White patients might miss important genetic differences that could affect how well treatments work for Black patients. | This study examined tumor samples from 211 Black women with a serious type of ovarian cancer to understand the genetic changes in their tumors, since most previous cancer research has focused on White patients. While the researchers found that Black and White women's tumors were very similar overall, they discovered some important differences - including certain genes that hadn't been identified before and different patterns of DNA damage repair problems in Black women's tumors. These findings suggest that including more diverse patient populations in cancer research could lead to better, more personalized treatments for all patients, especially since Black women with ovarian cancer tend to have worse outcomes than White women. | This study examined the genetic characteristics of a deadly type of ovarian cancer in Black women, who have worse survival rates and are rarely included in cancer research. Researchers analyzed tumor DNA from 211 Black patients and found that while most genetic features were similar to those seen in White patients, there were some important differences - including different mutated genes and patterns of DNA damage that could affect treatment decisions. The findings suggest that studying tumors from diverse patient populations is crucial because relying only on data from White patients might miss genetic differences that could help doctors provide better, more personalized treatments for Black women with ovarian cancer. |
| CancerRes | 2 | 10.1158/0008-5472.CAN-24-1882 | Gemini | Black individuals experience worse survival after a diagnosis of high-grade serous ovarian carcinoma (HGSC) than White individuals and are underrepresented in ovarian cancer research. To date, the understanding of the molecular and genomic heterogeneity of HGSC is based primarily on the evaluation of tumors from White individuals. In the present study, we performed whole-exome sequencing on HGSC samples from 211 Black patients to identify significantly mutated genes and characterize mutational signatures, assessing their distributions by gene expression subtypes. The occurrence and frequency of somatic mutations and signatures by self-reported race were compared with historic data from The Cancer Genome Atlas (TCGA). Despite technical differences (e.g., formalin-fixed vs. fresh-frozen tissue), the distribution of mutations and their variant classifications for major HGSC genes were nearly identical across study populations. However, de novo significantly mutated gene analysis identified genes not previously reported in TCGA analysis, including the oncogene KRAS and the potential tumor suppressor OBSCN. The prevalence of the homologous recombination deficiency signature was higher among Black individuals with the immunoreactive gene expression subtype compared with the mesenchymal and proliferative subtypes. These findings were confirmed by comparing the data from Black patients with those from 123 White patients with identical tissue collection and processing. Overall, this study suggests that, although most features of HGSC tumor phenotypes are similar in Black and White populations, there may be clinically relevant differences. If validated, these phenotypes may be important for clinical decision-making and would have been missed by characterizing tumors from White individuals only.Significance: Elucidation of the somatic mutational landscape of high-grade serous ovarian carcinoma in Black individuals, who experience poor survival and are underrepresented in research, could inform patient prognosis and enable precision medicine opportunities. | Researchers studied the genetics of a serious type of ovarian cancer in Black women to see if there were differences compared to White women. Previous research on this cancer was mostly done on White individuals, even though Black individuals tend to have worse outcomes. The study found that while many of the cancer's genetic features were similar between the two groups, there were some important differences.  Specifically, the study identified certain gene mutations, such as KRAS and OBSCN, that had not been previously highlighted in similar studies of White individuals. They also found that a specific genetic signature, called homologous recombination deficiency, was more common in a certain subtype of tumors in Black individuals. These findings suggest that there might be unique, clinically relevant differences in the biology of this cancer in Black individuals. If these differences are validated, they could lead to more personalized and effective treatments. | A recent study of ovarian cancer in Black individuals found that while most of the genetic features of the tumors were similar to those in White individuals, some important differences were discovered. Researchers analyzed tumors from 211 Black patients and identified new genes, including KRAS and OBSCN, that had not been previously linked to this type of cancer. The study also found a higher prevalence of a specific genetic signature in Black patients with a certain tumor subtype. These findings suggest that the genetic makeup of these tumors may differ in ways that could be important for making clinical decisions and developing more personalized treatments for Black patients, who have historically been underrepresented in research. | A recent study focused on high-grade serous ovarian carcinoma (HGSC), a type of ovarian cancer, found that Black individuals experience worse survival rates and are underrepresented in research. The study, which analyzed tumor samples from **211 Black patients**, aimed to understand the genomic characteristics of the disease in this population. It compared its findings to historical data, which primarily came from White patients. The research found that while many features of the tumors were similar across both Black and White individuals, there were some notable differences. For example, the study identified specific gene mutations, like **KRAS** and **OBSCN**, that had not been previously reported in studies of White patients. Additionally, the study found a higher prevalence of a specific mutational signature related to homologous recombination deficiency in Black individuals with the immunoreactive gene expression subtype. These findings suggest there may be clinically significant differences in HGSC between racial groups, which could impact treatment and patient outcomes. |
| CancerRes | 2 | 10.1158/0008-5472.CAN-24-1883 | DeepSeek | Black individuals experience worse survival after a diagnosis of high-grade serous ovarian carcinoma (HGSC) than White individuals and are underrepresented in ovarian cancer research. To date, the understanding of the molecular and genomic heterogeneity of HGSC is based primarily on the evaluation of tumors from White individuals. In the present study, we performed whole-exome sequencing on HGSC samples from 211 Black patients to identify significantly mutated genes and characterize mutational signatures, assessing their distributions by gene expression subtypes. The occurrence and frequency of somatic mutations and signatures by self-reported race were compared with historic data from The Cancer Genome Atlas (TCGA). Despite technical differences (e.g., formalin-fixed vs. fresh-frozen tissue), the distribution of mutations and their variant classifications for major HGSC genes were nearly identical across study populations. However, de novo significantly mutated gene analysis identified genes not previously reported in TCGA analysis, including the oncogene KRAS and the potential tumor suppressor OBSCN. The prevalence of the homologous recombination deficiency signature was higher among Black individuals with the immunoreactive gene expression subtype compared with the mesenchymal and proliferative subtypes. These findings were confirmed by comparing the data from Black patients with those from 123 White patients with identical tissue collection and processing. Overall, this study suggests that, although most features of HGSC tumor phenotypes are similar in Black and White populations, there may be clinically relevant differences. If validated, these phenotypes may be important for clinical decision-making and would have been missed by characterizing tumors from White individuals only.Significance: Elucidation of the somatic mutational landscape of high-grade serous ovarian carcinoma in Black individuals, who experience poor survival and are underrepresented in research, could inform patient prognosis and enable precision medicine opportunities. | This study examined the genetic differences in high-grade serous ovarian cancer (HGSC) between Black and White individuals, as Black patients often have worse survival rates and are underrepresented in research. By analyzing tumors from 211 Black patients, researchers found that while most genetic mutations were similar to those in White patients (based on past data), some unique mutations—like in the KRAS and OBSCN genes—were more common in Black patients. They also discovered differences in how certain tumor subtypes responded to treatment. These findings suggest that while HGSC is mostly similar across races, there may be key genetic differences that could affect treatment decisions, highlighting the need for more inclusive research to improve care for all patients. | This study explored genetic differences in high-grade serous ovarian cancer (HGSC) between Black and White individuals, as Black patients often have worse survival rates and are underrepresented in research. By analyzing tumors from 211 Black patients, researchers found that while most genetic mutations were similar to those in White patients (based on past data), some unique mutations were identified in Black patients, including in the KRAS oncogene and OBSCN tumor suppressor. Additionally, Black patients with a specific immune-related tumor subtype had more DNA repair defects, which could affect treatment response. The findings suggest that while HGSC is genetically similar across races, certain differences may impact outcomes and treatment, highlighting the need for more inclusive research to improve personalized care for all patients. | This study explored genetic differences in high-grade serous ovarian cancer (HGSC) between Black and White individuals, as Black patients often have worse survival rates and are underrepresented in research. By analyzing tumors from 211 Black patients, researchers found that while most genetic mutations were similar to those in White patients (based on past studies), some unique mutations were identified in Black patients, including in the KRAS oncogene and OBSCN tumor suppressor. Additionally, Black patients with a specific immune-related tumor subtype showed more signs of DNA repair defects. Comparing these findings to 123 White patients confirmed these differences. The study suggests that while HGSC is mostly similar across races, certain genetic variations in Black patients could impact treatment and survival, highlighting the need for more inclusive research to improve personalized care. |
| CancerRes | 2 | 10.1158/0008-5472.CAN-24-1884 | Grok | Black individuals experience worse survival after a diagnosis of high-grade serous ovarian carcinoma (HGSC) than White individuals and are underrepresented in ovarian cancer research. To date, the understanding of the molecular and genomic heterogeneity of HGSC is based primarily on the evaluation of tumors from White individuals. In the present study, we performed whole-exome sequencing on HGSC samples from 211 Black patients to identify significantly mutated genes and characterize mutational signatures, assessing their distributions by gene expression subtypes. The occurrence and frequency of somatic mutations and signatures by self-reported race were compared with historic data from The Cancer Genome Atlas (TCGA). Despite technical differences (e.g., formalin-fixed vs. fresh-frozen tissue), the distribution of mutations and their variant classifications for major HGSC genes were nearly identical across study populations. However, de novo significantly mutated gene analysis identified genes not previously reported in TCGA analysis, including the oncogene KRAS and the potential tumor suppressor OBSCN. The prevalence of the homologous recombination deficiency signature was higher among Black individuals with the immunoreactive gene expression subtype compared with the mesenchymal and proliferative subtypes. These findings were confirmed by comparing the data from Black patients with those from 123 White patients with identical tissue collection and processing. Overall, this study suggests that, although most features of HGSC tumor phenotypes are similar in Black and White populations, there may be clinically relevant differences. If validated, these phenotypes may be important for clinical decision-making and would have been missed by characterizing tumors from White individuals only.Significance: Elucidation of the somatic mutational landscape of high-grade serous ovarian carcinoma in Black individuals, who experience poor survival and are underrepresented in research, could inform patient prognosis and enable precision medicine opportunities. | This study analyzed the genetic makeup of high-grade serous ovarian carcinoma (HGSC) in 211 Black patients using whole-exome sequencing to identify key gene mutations and patterns, comparing them to data from White patients in The Cancer Genome Atlas (TCGA). While most genetic features of HGSC were similar between Black and White patients, the study found unique mutations in genes like KRAS and OBSCN in Black patients, along with a higher prevalence of a specific DNA repair defect in Black patients with certain tumor subtypes. These differences, not seen in studies of White patients alone, could impact treatment decisions and highlight the importance of including diverse populations in cancer research. | This study analyzed the DNA of ovarian cancer tumors from 211 Black patients to understand their genetic features compared to data from mostly White patients in The Cancer Genome Atlas. While most tumor characteristics were similar between Black and White patients, the study found unique gene mutations in Black patients, including in the KRAS and OBSCN genes, and a higher presence of a specific DNA repair defect in certain tumor subtypes. These differences, confirmed by comparing with 123 White patients, suggest that studying diverse populations could reveal important insights for better diagnosis and treatment of ovarian cancer in Black individuals. | This study analyzed the genetic makeup of high-grade serous ovarian carcinoma (HGSC) in 211 Black patients using whole-exome sequencing to identify key gene mutations and patterns, comparing them to data from White patients in The Cancer Genome Atlas. While most genetic features were similar between Black and White patients, the study found unique mutations in genes like KRAS and OBSCN, and a higher prevalence of a specific DNA repair defect in Black patients with a certain tumor subtype. These differences, not seen in prior studies focused on White patients, suggest that including Black patients in research could reveal important insights for better diagnosis and treatment. |
| CancerRes | 3 | 10.1158/0008-5472.CAN-24-1518 | Open AI | Early detection of lung cancer is important for improving patient survival rates. Liquid biopsy using whole-genome sequencing of cell-free DNA (cfDNA) offers a promising avenue for lung cancer screening, providing a potential alternative or complementary approach to current screening modalities. Here, we aimed to develop and validate an approach by integrating fragment and genomic features of cfDNA to enhance lung cancer detection accuracy across diverse populations. Deep learning-based classifiers were trained using comprehensive cfDNA fragmentomic features from participants in multi-institutional studies, including a Korean discovery dataset (218 patients with lung cancer and 2,559 controls), a Korean validation dataset (111 patients with lung cancer and 1,136 controls), and an independent Caucasian validation cohort (50 patients with lung cancer and 50 controls). In the discovery dataset, classifiers using fragment end motif by size, a feature that captures both fragment end motif and size profiles, outperformed standalone fragment end motif and fragment size classifiers, achieving an area under the curve (AUC) of 0.917. The ensemble classifier integrating fragment end motif by size and genomic coverage achieved an improved performance, with an AUC of 0.937. This performance extended to the Korean validation dataset and demonstrated ethnic generalizability in the Caucasian validation cohort. Overall, the development of a deep learning-based classifier integrating cfDNA fragmentomic and genomic features in this study highlights the potential for accurate lung cancer detection across diverse populations.Significance: Evaluating fragment-based features and genomic coverage in cell-free DNA offers an accurate lung cancer screening method, promising improvements in early cancer detection and addressing challenges associated with current screening methods. | This study developed a new method to detect lung cancer early by analyzing tiny pieces of DNA floating in the blood, using advanced computer learning. By combining different DNA features, the method showed high accuracy in identifying lung cancer in people from different ethnic backgrounds. This approach could improve lung cancer screening and help catch the disease sooner than current tests. | Early detection of lung cancer helps save lives, and a new blood test using cell-free DNA (cfDNA) shows promise for this. Researchers developed a deep learning method that analyzes tiny DNA fragments in the blood to better detect lung cancer. They tested it on large groups from Korea and the U.S. and found it accurately identified lung cancer across different ethnic groups. This approach could improve lung cancer screening and catch cancer earlier than current methods. | This study shows that early lung cancer can be detected accurately by analyzing tiny pieces of DNA floating in the blood, using a method called liquid biopsy. By combining patterns of DNA fragments and their genetic information with deep learning (a type of artificial intelligence), researchers created a test that worked well in different groups of people, including Koreans and Caucasians. This new approach could help find lung cancer earlier and improve survival rates, offering a promising alternative to current screening methods. |
| CancerRes | 3 | 10.1158/0008-5472.CAN-24-1519 | Claude | Early detection of lung cancer is important for improving patient survival rates. Liquid biopsy using whole-genome sequencing of cell-free DNA (cfDNA) offers a promising avenue for lung cancer screening, providing a potential alternative or complementary approach to current screening modalities. Here, we aimed to develop and validate an approach by integrating fragment and genomic features of cfDNA to enhance lung cancer detection accuracy across diverse populations. Deep learning-based classifiers were trained using comprehensive cfDNA fragmentomic features from participants in multi-institutional studies, including a Korean discovery dataset (218 patients with lung cancer and 2,559 controls), a Korean validation dataset (111 patients with lung cancer and 1,136 controls), and an independent Caucasian validation cohort (50 patients with lung cancer and 50 controls). In the discovery dataset, classifiers using fragment end motif by size, a feature that captures both fragment end motif and size profiles, outperformed standalone fragment end motif and fragment size classifiers, achieving an area under the curve (AUC) of 0.917. The ensemble classifier integrating fragment end motif by size and genomic coverage achieved an improved performance, with an AUC of 0.937. This performance extended to the Korean validation dataset and demonstrated ethnic generalizability in the Caucasian validation cohort. Overall, the development of a deep learning-based classifier integrating cfDNA fragmentomic and genomic features in this study highlights the potential for accurate lung cancer detection across diverse populations.Significance: Evaluating fragment-based features and genomic coverage in cell-free DNA offers an accurate lung cancer screening method, promising improvements in early cancer detection and addressing challenges associated with current screening methods. | This research developed a new blood test for detecting lung cancer early using a technique called liquid biopsy. Instead of tissue samples, scientists analyzed small pieces of DNA floating freely in the blood (called cell-free DNA) using advanced computer learning methods. They tested this approach on over 4,000 people from Korean and Caucasian populations, including both cancer patients and healthy individuals. The blood test was highly accurate at identifying lung cancer, correctly detecting it about 94% of the time. This method could provide a simpler, less invasive alternative to current lung cancer screening approaches and may help catch the disease earlier when treatment is most effective. | Researchers developed a new blood test that could help detect lung cancer early by analyzing tiny pieces of DNA floating freely in the blood. They used artificial intelligence to examine specific patterns in this DNA from over 4,000 people across different ethnic groups (Korean and Caucasian populations). The test was able to accurately identify lung cancer cases about 94% of the time by looking at both the size and shape of DNA fragments as well as how much DNA was present. This approach could offer a simpler, more accessible alternative to current lung cancer screening methods and potentially save more lives through earlier detection across diverse populations. | This research developed a new blood test for detecting lung cancer early using artificial intelligence. Scientists analyzed DNA fragments floating freely in blood samples from over 4,000 people across Korean and Caucasian populations. They trained computer programs to recognize patterns in these DNA fragments that indicate lung cancer. The best approach combined information about fragment patterns and genetic coverage, achieving high accuracy (over 90%) in identifying lung cancer patients. This method could provide a simpler, more accessible screening option compared to current lung cancer detection methods, potentially helping doctors catch the disease earlier when treatment is most effective. |
| CancerRes | 3 | 10.1158/0008-5472.CAN-24-1520 | Gemini | Early detection of lung cancer is important for improving patient survival rates. Liquid biopsy using whole-genome sequencing of cell-free DNA (cfDNA) offers a promising avenue for lung cancer screening, providing a potential alternative or complementary approach to current screening modalities. Here, we aimed to develop and validate an approach by integrating fragment and genomic features of cfDNA to enhance lung cancer detection accuracy across diverse populations. Deep learning-based classifiers were trained using comprehensive cfDNA fragmentomic features from participants in multi-institutional studies, including a Korean discovery dataset (218 patients with lung cancer and 2,559 controls), a Korean validation dataset (111 patients with lung cancer and 1,136 controls), and an independent Caucasian validation cohort (50 patients with lung cancer and 50 controls). In the discovery dataset, classifiers using fragment end motif by size, a feature that captures both fragment end motif and size profiles, outperformed standalone fragment end motif and fragment size classifiers, achieving an area under the curve (AUC) of 0.917. The ensemble classifier integrating fragment end motif by size and genomic coverage achieved an improved performance, with an AUC of 0.937. This performance extended to the Korean validation dataset and demonstrated ethnic generalizability in the Caucasian validation cohort. Overall, the development of a deep learning-based classifier integrating cfDNA fragmentomic and genomic features in this study highlights the potential for accurate lung cancer detection across diverse populations.Significance: Evaluating fragment-based features and genomic coverage in cell-free DNA offers an accurate lung cancer screening method, promising improvements in early cancer detection and addressing challenges associated with current screening methods. | The abstract describes a new method for detecting lung cancer early using a blood test. The method analyzes fragments of DNA from a person's blood, looking at both the size and specific patterns at the ends of the fragments, as well as where the fragments came from in the genome. By using a deep learning classifier, the researchers were able to accurately detect lung cancer in a study involving Korean and Caucasian participants, showing the method works across different populations. This approach offers a promising way to improve lung cancer screening. | Scientists developed a new method for detecting lung cancer early using a blood test. They analyzed cell-free DNA (cfDNA), which are tiny pieces of DNA circulating in the blood, from a large group of people. By using a deep learning model, they looked at the unique patterns and sizes of these DNA fragments. This approach was successful in finding lung cancer in both Korean and Caucasian populations, showing its potential for accurate and early detection in diverse groups. The method, which combines different DNA features, performed better than looking at just one feature alone. | Scientists are working on a new way to detect lung cancer early using a simple blood test. They analyzed DNA fragments from the blood, called cell-free DNA (cfDNA), of people from both Korean and Caucasian backgrounds. By using a special computer program called a deep learning classifier, they found that combining information about the size and patterns of these DNA fragments, along with where they came from in the genome, was highly effective at identifying lung cancer. This new method performed well in different groups of people, suggesting it could be a promising tool for accurate, early lung cancer screening across diverse populations. |
| CancerRes | 3 | 10.1158/0008-5472.CAN-24-1521 | DeepSeek | Early detection of lung cancer is important for improving patient survival rates. Liquid biopsy using whole-genome sequencing of cell-free DNA (cfDNA) offers a promising avenue for lung cancer screening, providing a potential alternative or complementary approach to current screening modalities. Here, we aimed to develop and validate an approach by integrating fragment and genomic features of cfDNA to enhance lung cancer detection accuracy across diverse populations. Deep learning-based classifiers were trained using comprehensive cfDNA fragmentomic features from participants in multi-institutional studies, including a Korean discovery dataset (218 patients with lung cancer and 2,559 controls), a Korean validation dataset (111 patients with lung cancer and 1,136 controls), and an independent Caucasian validation cohort (50 patients with lung cancer and 50 controls). In the discovery dataset, classifiers using fragment end motif by size, a feature that captures both fragment end motif and size profiles, outperformed standalone fragment end motif and fragment size classifiers, achieving an area under the curve (AUC) of 0.917. The ensemble classifier integrating fragment end motif by size and genomic coverage achieved an improved performance, with an AUC of 0.937. This performance extended to the Korean validation dataset and demonstrated ethnic generalizability in the Caucasian validation cohort. Overall, the development of a deep learning-based classifier integrating cfDNA fragmentomic and genomic features in this study highlights the potential for accurate lung cancer detection across diverse populations.Significance: Evaluating fragment-based features and genomic coverage in cell-free DNA offers an accurate lung cancer screening method, promising improvements in early cancer detection and addressing challenges associated with current screening methods. | This study developed a new blood test using cell-free DNA (cfDNA) to detect lung cancer early. By combining information about DNA fragment patterns (like size and end motifs) with genomic data, a deep learning model was trained and tested on diverse groups, including Korean and Caucasian participants. The method showed high accuracy in identifying lung cancer, even across different ethnicities, and performed better than using fragment or genomic data alone. This approach could improve early lung cancer detection, offering a simpler alternative to current screening methods like CT scans. | This study developed a new method to detect lung cancer early by analyzing DNA fragments in blood (cfDNA). Using deep learning, researchers combined information about the size and end patterns of these fragments with genomic data, creating a highly accurate test. The method was tested in Korean and Caucasian groups, showing strong performance across different populations. This approach could improve lung cancer screening, offering a simpler and more effective alternative to current methods. | This study developed a new blood test using cell-free DNA (cfDNA) to detect lung cancer early. By combining information about DNA fragment patterns (like size and end motifs) with genomic data, a deep learning model achieved high accuracy in identifying lung cancer across different groups, including Korean and Caucasian populations. The approach showed better performance than using fragment features alone, offering a promising, less invasive alternative to current screening methods like CT scans. This could help improve early detection and survival rates for lung cancer patients. |
| CancerRes | 3 | 10.1158/0008-5472.CAN-24-1522 | Grok | Early detection of lung cancer is important for improving patient survival rates. Liquid biopsy using whole-genome sequencing of cell-free DNA (cfDNA) offers a promising avenue for lung cancer screening, providing a potential alternative or complementary approach to current screening modalities. Here, we aimed to develop and validate an approach by integrating fragment and genomic features of cfDNA to enhance lung cancer detection accuracy across diverse populations. Deep learning-based classifiers were trained using comprehensive cfDNA fragmentomic features from participants in multi-institutional studies, including a Korean discovery dataset (218 patients with lung cancer and 2,559 controls), a Korean validation dataset (111 patients with lung cancer and 1,136 controls), and an independent Caucasian validation cohort (50 patients with lung cancer and 50 controls). In the discovery dataset, classifiers using fragment end motif by size, a feature that captures both fragment end motif and size profiles, outperformed standalone fragment end motif and fragment size classifiers, achieving an area under the curve (AUC) of 0.917. The ensemble classifier integrating fragment end motif by size and genomic coverage achieved an improved performance, with an AUC of 0.937. This performance extended to the Korean validation dataset and demonstrated ethnic generalizability in the Caucasian validation cohort. Overall, the development of a deep learning-based classifier integrating cfDNA fragmentomic and genomic features in this study highlights the potential for accurate lung cancer detection across diverse populations.Significance: Evaluating fragment-based features and genomic coverage in cell-free DNA offers an accurate lung cancer screening method, promising improvements in early cancer detection and addressing challenges associated with current screening methods. | Researchers developed a new method to detect lung cancer early using a blood test called liquid biopsy, which analyzes cell-free DNA (cfDNA) with whole-genome sequencing. By combining features like fragment end motifs and sizes with genomic data, they trained deep learning models on datasets from Korean and Caucasian populations. The approach, particularly the model integrating fragment end motif by size and genomic coverage, showed high accuracy (AUC of 0.937) in identifying lung cancer across diverse groups, offering a promising, less invasive alternative or complement to current screening methods. | Researchers developed a new method to detect lung cancer early using a blood test called liquid biopsy, which analyzes cell-free DNA (cfDNA) from a person's blood. By combining information about the size and patterns of DNA fragments with other genetic features, they created a deep learning model that accurately identifies lung cancer. The model was tested on diverse groups, including Korean and Caucasian populations, and showed strong results with high accuracy (AUC of 0.937). This approach could improve early lung cancer detection and complement existing screening methods. | Researchers developed a new method to detect lung cancer early using a blood test called liquid biopsy, which analyzes cell-free DNA (cfDNA). By combining features like fragment end motifs, fragment sizes, and genomic coverage with deep learning, they created a classifier that accurately identifies lung cancer. Tested on diverse groups from Korea and a Caucasian cohort, the method achieved high accuracy (AUC of 0.937) and shows promise as a reliable, non-invasive screening tool to improve early lung cancer detection and patient outcomes. |
| CancerRes | 4 | 10.1158/0008-5472.CAN-25-0988 | Open AI | Therapy resistance is a significant cause of death in patients treated with targeted cancer therapy in diverse oncogene-driven cancers. A better understanding of resistance mechanisms can lay the foundation for improving existing and developing new therapies. A recent elegant study published in Nature Chemical Biology sheds light on a new resistance mechanism. The authors define a novel role for ARAF, a member of the RAF protein family (ARAF, BRAF, and CRAF), that is distinct from its previously understood role as a RAS effector and MEK protein kinase in the MAPK pathway. They describe how ARAF sequesters active RAS at the plasma membrane in phase-separated condensates to sustain signaling and prevent inactivation by the RAS GTPase-activating protein neurofibromin 1. This study underscores emerging roles for biomolecular condensates in cancer and highlights important implications for disrupting protein condensates to address treatment resistance to RAS (and RAS pathway)-targeted therapies. The study also illuminates evolutionary functional distinction between the RAF proteins and indicates unique biology for ARAF in normal physiology and disease. | A new study found that the protein ARAF, part of the RAF family, can help cancer cells resist targeted treatments by trapping active RAS proteins at the cell membrane, keeping cancer growth signals turned on. This process happens in special protein clusters called biomolecular condensates and prevents RAS from being switched off. The discovery reveals a unique role for ARAF, different from other RAF proteins, and suggests that disrupting these condensates could help overcome treatment resistance in RAS-related cancers. | A new study found that ARAF, a protein in the RAF family, can help cancer cells resist targeted treatments by trapping active RAS proteins in special clusters on the cell membrane. This keeps cancer growth signals active and prevents them from being shut off. The discovery shows that disrupting these protein clusters could be a new way to overcome resistance to RAS-targeted therapies, and it reveals unique functions for ARAF compared to other RAF proteins. | A new study in Nature Chemical Biology found that ARAF, a protein in the RAF family, can help cancer cells resist targeted treatments. Unlike its usual role in a known signaling pathway, ARAF traps active RAS proteins in special clusters at the cell membrane, keeping them active and protecting them from being turned off. This discovery reveals a unique function for ARAF, shows how these protein clusters can drive cancer, and suggests that breaking them apart could be a way to overcome treatment resistance. |
| CancerRes | 4 | 10.1158/0008-5472.CAN-25-0989 | Claude | Therapy resistance is a significant cause of death in patients treated with targeted cancer therapy in diverse oncogene-driven cancers. A better understanding of resistance mechanisms can lay the foundation for improving existing and developing new therapies. A recent elegant study published in Nature Chemical Biology sheds light on a new resistance mechanism. The authors define a novel role for ARAF, a member of the RAF protein family (ARAF, BRAF, and CRAF), that is distinct from its previously understood role as a RAS effector and MEK protein kinase in the MAPK pathway. They describe how ARAF sequesters active RAS at the plasma membrane in phase-separated condensates to sustain signaling and prevent inactivation by the RAS GTPase-activating protein neurofibromin 1. This study underscores emerging roles for biomolecular condensates in cancer and highlights important implications for disrupting protein condensates to address treatment resistance to RAS (and RAS pathway)-targeted therapies. The study also illuminates evolutionary functional distinction between the RAF proteins and indicates unique biology for ARAF in normal physiology and disease. | Cancer treatments that target specific cancer-causing proteins often stop working because cancer cells develop resistance to them, leading to patient deaths. A new study found that a protein called ARAF helps cancer cells resist treatment in an unexpected way - instead of just passing along growth signals like scientists thought, ARAF actually traps and protects the cancer-driving RAS protein at the cell surface, keeping it active even when the cell tries to shut it down. This discovery reveals a new weakness that researchers could potentially target to make cancer treatments work better and last longer. | Cancer treatments that target specific proteins often stop working because cancer cells develop resistance, which can lead to patient death. Researchers recently discovered a new way this resistance happens: a protein called ARAF creates special clusters at the cell surface that protect cancer-promoting RAS proteins from being turned off. This finding helps explain why some cancer treatments fail and could lead to new approaches for overcoming treatment resistance by breaking up these protective protein clusters. | When cancer patients receive targeted treatments, their tumors often become resistant to the drugs, which can be deadly. Scientists recently discovered a new way this resistance happens. They found that a protein called ARAF acts like a protective shield around cancer-promoting signals at the cell surface, keeping them active and preventing the cell's natural shutdown mechanisms from working. This discovery helps explain why some cancer treatments stop working and could lead to better ways to overcome drug resistance in the future. |
| CancerRes | 4 | 10.1158/0008-5472.CAN-25-0990 | Gemini | Therapy resistance is a significant cause of death in patients treated with targeted cancer therapy in diverse oncogene-driven cancers. A better understanding of resistance mechanisms can lay the foundation for improving existing and developing new therapies. A recent elegant study published in Nature Chemical Biology sheds light on a new resistance mechanism. The authors define a novel role for ARAF, a member of the RAF protein family (ARAF, BRAF, and CRAF), that is distinct from its previously understood role as a RAS effector and MEK protein kinase in the MAPK pathway. They describe how ARAF sequesters active RAS at the plasma membrane in phase-separated condensates to sustain signaling and prevent inactivation by the RAS GTPase-activating protein neurofibromin 1. This study underscores emerging roles for biomolecular condensates in cancer and highlights important implications for disrupting protein condensates to address treatment resistance to RAS (and RAS pathway)-targeted therapies. The study also illuminates evolutionary functional distinction between the RAF proteins and indicates unique biology for ARAF in normal physiology and disease. | The provided text describes a new way cancer cells resist treatment. A protein called ARAF, which is part of the RAS pathway, creates tiny clumps called condensates. These condensates trap active RAS protein at the cell's membrane, keeping it from being deactivated. This sustained activity of RAS signaling allows cancer to continue growing even when targeted therapies are used. This discovery suggests that disrupting these condensates could be a new strategy to overcome therapy resistance in cancer. | A recent study published in Nature Chemical Biology uncovered a new way that cancer cells can become resistant to therapy. It focuses on the ARAF protein, which helps keep the cancer-promoting RAS protein active. Instead of just being a normal part of the signaling pathway, ARAF traps active RAS proteins at the cell's membrane in protective droplets called condensates. This prevents RAS from being turned off, allowing it to continue promoting cancer growth despite treatment. This discovery highlights how targeting these condensates could be a new way to overcome drug resistance in cancers driven by the RAS pathway. | A new study has found a reason why some cancer therapies stop working. It turns out that a protein called ARAF helps cancer cells resist treatment by trapping another protein, RAS, in special clumps called condensates. This keeps the RAS protein active and helps the cancer grow. This discovery suggests that disrupting these condensates could be a new way to fight cancer and overcome drug resistance. |
| CancerRes | 4 | 10.1158/0008-5472.CAN-25-0991 | DeepSeek | Therapy resistance is a significant cause of death in patients treated with targeted cancer therapy in diverse oncogene-driven cancers. A better understanding of resistance mechanisms can lay the foundation for improving existing and developing new therapies. A recent elegant study published in Nature Chemical Biology sheds light on a new resistance mechanism. The authors define a novel role for ARAF, a member of the RAF protein family (ARAF, BRAF, and CRAF), that is distinct from its previously understood role as a RAS effector and MEK protein kinase in the MAPK pathway. They describe how ARAF sequesters active RAS at the plasma membrane in phase-separated condensates to sustain signaling and prevent inactivation by the RAS GTPase-activating protein neurofibromin 1. This study underscores emerging roles for biomolecular condensates in cancer and highlights important implications for disrupting protein condensates to address treatment resistance to RAS (and RAS pathway)-targeted therapies. The study also illuminates evolutionary functional distinction between the RAF proteins and indicates unique biology for ARAF in normal physiology and disease. | A recent study reveals a new way cancer cells resist targeted therapies. Researchers found that the protein ARAF, part of the RAF family, forms clumps (condensates) at the cell membrane that trap active RAS, keeping it turned on and preventing its shutdown. This helps cancer cells survive treatment. The discovery highlights how these protein clumps play a role in drug resistance and suggests targeting them could improve therapies for RAS-driven cancers. It also shows that ARAF has a unique function compared to other RAF proteins. | This study reveals a new way cancer cells resist targeted therapies. Researchers found that the protein ARAF, part of the RAF family, forms liquid-like clusters (condensates) at the cell membrane, trapping active RAS and keeping it turned on—even when it should be shut off by other proteins. This helps cancer cells survive treatment. The discovery highlights how these condensates play a key role in drug resistance and suggests that disrupting them could improve therapies targeting RAS and related pathways. It also shows that ARAF has a unique function compared to other RAF proteins in both health and disease. | A recent study reveals a new way cancer cells resist targeted therapies. Researchers found that the protein ARAF, part of the RAF family, forms liquid-like clusters (condensates) at the cell membrane, trapping active RAS and keeping it turned on—even when it should be shut off by other proteins. This helps cancer cells survive treatment. The discovery highlights how these condensates play a role in drug resistance and suggests that disrupting them could improve therapies targeting RAS-driven cancers. It also shows that ARAF has a unique function compared to other RAF proteins in both health and disease. |
| CancerRes | 4 | 10.1158/0008-5472.CAN-25-0992 | Grok | Therapy resistance is a significant cause of death in patients treated with targeted cancer therapy in diverse oncogene-driven cancers. A better understanding of resistance mechanisms can lay the foundation for improving existing and developing new therapies. A recent elegant study published in Nature Chemical Biology sheds light on a new resistance mechanism. The authors define a novel role for ARAF, a member of the RAF protein family (ARAF, BRAF, and CRAF), that is distinct from its previously understood role as a RAS effector and MEK protein kinase in the MAPK pathway. They describe how ARAF sequesters active RAS at the plasma membrane in phase-separated condensates to sustain signaling and prevent inactivation by the RAS GTPase-activating protein neurofibromin 1. This study underscores emerging roles for biomolecular condensates in cancer and highlights important implications for disrupting protein condensates to address treatment resistance to RAS (and RAS pathway)-targeted therapies. The study also illuminates evolutionary functional distinction between the RAF proteins and indicates unique biology for ARAF in normal physiology and disease. | A recent study in Nature Chemical Biology reveals a new way cancer cells resist targeted therapies by focusing on ARAF, a protein in the RAF family. Unlike its known role in helping RAS activate the MAPK pathway, ARAF traps active RAS in special clusters at the cell membrane, called condensates. These clusters keep RAS signaling strong and block a protein that normally turns RAS off. This discovery highlights how these condensates contribute to cancer treatment resistance and suggests that breaking them up could improve therapies targeting RAS and its pathways, while also showing ARAF’s unique role compared to other RAF proteins. | A recent study in Nature Chemical Biology reveals a new way cancer cells resist treatment by focusing on ARAF, a protein in the RAF family. Unlike its known role in helping RAS activate the MAPK pathway, ARAF traps active RAS in special clusters at the cell membrane, called condensates. These clusters keep RAS signaling active and block a protein called neurofibromin 1 from turning it off. This discovery highlights how these condensates contribute to cancer drug resistance and suggests targeting them could improve treatments for cancers driven by RAS and related pathways, while also showing ARAF’s unique role compared to other RAF proteins. | A recent study in Nature Chemical Biology reveals a new way cancer cells resist targeted therapies by focusing on ARAF, a protein in the RAF family. Unlike its usual role in the MAPK pathway, ARAF traps active RAS proteins in special clusters at the cell membrane, called condensates, to keep cancer signaling active and block a protein that normally turns RAS off. This discovery highlights how these condensates contribute to cancer treatment resistance and suggests that breaking them up could improve therapies targeting RAS and its pathways, while also showing ARAF’s unique role compared to other RAF proteins. |
| CancerRes | 5 | 10.1158/0008-5472.CAN-24-2290 | Open AI | Non-coding RNA transcribed from active enhancers, known as enhancer RNA (eRNA), is a critical element in gene regulation with a highly specific expression pattern in the regulatory networks of tumor-infiltrating cells. Therefore, eRNA signatures could potentially be applied to represent antitumor immune cells and to improve cancer immunotherapy. In this study, we identified thousands of eRNAs that were significantly correlated with infiltrating immune cell abundance in more than 10,000 patient samples across a variety of cancer types. The expression of these eRNAs was mediated by transcription factors with high expression in antitumor immune cells, as identified through single-cell assays. An eRNA immunotherapy signature (eRIS) developed using the antitumor eRNAs was highly associated with the objective response rate of immunotherapy and was elevated in patients who benefited from immune checkpoint blockade treatment. In comparison with a signature based on protein-coding genes, the eRIS was more effective in predicting the response to immunotherapy. Integration of the eRIS with pharmacogenomic data revealed hundreds of anticancer drugs that have the potential to enhance immunotherapy efficacy. Finally, treatment of a mouse model of IDH-mutant glioma with the histone deacetylase inhibitor vorinostat improved the effects of anti-PD-1 immunotherapy through increased abundance of infiltrating immune cells. Taken together, this study developed an eRIS with demonstrated efficacy in predicting immunotherapy response and used the eRIS to identify a series of effective combination drugs, thus highlighting the clinical utility of the eRIS in immunotherapy enhancement.Significance: An eRNA immunotherapy signature developed using antitumor eRNAs in tumor-infiltrating immune cells improves the prediction of response to immunotherapy and identifies a series of effective drug combinations to enhance immunotherapy efficacy. | Researchers found that certain non-coding RNAs called enhancer RNAs (eRNAs), made by active enhancers in immune cells within tumors, can help predict how well cancer patients will respond to immunotherapy. By studying over 10,000 patient samples, they created an eRNA immunotherapy signature (eRIS) that outperformed traditional protein-coding gene markers in predicting treatment success. The eRIS also helped identify many drugs that could boost immunotherapy, and in a mouse model, one such drug improved the effectiveness of anti-PD-1 treatment by increasing immune cell activity. | Researchers found that special molecules called enhancer RNAs (eRNAs) in tumor-infiltrating immune cells can help predict how well cancer patients will respond to immunotherapy. They created an eRNA-based score, called eRIS, which worked better than traditional gene-based methods in predicting treatment success. Using eRIS, they also identified many existing drugs that could boost immunotherapy effects, and in mouse experiments, one such drug improved immune response against cancer. This work suggests eRIS could guide both treatment choices and effective drug combinations for cancer patients. | This study found that certain enhancer RNAs (eRNAs), which help control gene activity, are strongly linked to the presence of immune cells that fight cancer. By analyzing over 10,000 cancer patient samples, researchers created an eRNA immunotherapy signature (eRIS) that can predict how well patients will respond to immunotherapy, doing better than traditional gene-based methods. They also used eRIS to identify many drugs that could boost immunotherapy results, and in mouse experiments, one such drug (vorinostat) improved treatment by increasing cancer-fighting immune cells. |
| CancerRes | 5 | 10.1158/0008-5472.CAN-24-2291 | Claude | Non-coding RNA transcribed from active enhancers, known as enhancer RNA (eRNA), is a critical element in gene regulation with a highly specific expression pattern in the regulatory networks of tumor-infiltrating cells. Therefore, eRNA signatures could potentially be applied to represent antitumor immune cells and to improve cancer immunotherapy. In this study, we identified thousands of eRNAs that were significantly correlated with infiltrating immune cell abundance in more than 10,000 patient samples across a variety of cancer types. The expression of these eRNAs was mediated by transcription factors with high expression in antitumor immune cells, as identified through single-cell assays. An eRNA immunotherapy signature (eRIS) developed using the antitumor eRNAs was highly associated with the objective response rate of immunotherapy and was elevated in patients who benefited from immune checkpoint blockade treatment. In comparison with a signature based on protein-coding genes, the eRIS was more effective in predicting the response to immunotherapy. Integration of the eRIS with pharmacogenomic data revealed hundreds of anticancer drugs that have the potential to enhance immunotherapy efficacy. Finally, treatment of a mouse model of IDH-mutant glioma with the histone deacetylase inhibitor vorinostat improved the effects of anti-PD-1 immunotherapy through increased abundance of infiltrating immune cells. Taken together, this study developed an eRIS with demonstrated efficacy in predicting immunotherapy response and used the eRIS to identify a series of effective combination drugs, thus highlighting the clinical utility of the eRIS in immunotherapy enhancement.Significance: An eRNA immunotherapy signature developed using antitumor eRNAs in tumor-infiltrating immune cells improves the prediction of response to immunotherapy and identifies a series of effective drug combinations to enhance immunotherapy efficacy. | Researchers discovered that certain RNA molecules called enhancer RNAs (eRNAs) can help predict whether cancer patients will respond well to immunotherapy treatments. These eRNAs are produced by immune cells that fight tumors and have unique patterns in different patients. The scientists created a signature using these eRNAs that was better at predicting immunotherapy success than traditional methods based on protein-coding genes. They also used this signature to identify hundreds of existing drugs that could potentially be combined with immunotherapy to make it more effective, and tested one such combination successfully in mice with brain tumors. | Researchers developed a new way to predict how well cancer patients will respond to immunotherapy by studying special RNA molecules called enhancer RNAs (eRNAs) found in immune cells that fight tumors. They analyzed over 10,000 patient samples and created an "eRNA immunotherapy signature" that was better at predicting treatment success than traditional methods using protein-coding genes. This signature also helped them identify hundreds of existing cancer drugs that could be combined with immunotherapy to make it more effective. When they tested one such drug combination in mice with brain tumors, it significantly improved the immunotherapy results, suggesting this approach could lead to better personalized cancer treatments for patients. | Researchers discovered that certain RNA molecules called enhancer RNAs (eRNAs) can help predict how well cancer patients will respond to immunotherapy treatments. These eRNAs are produced by immune cells that fight tumors, and their levels can indicate whether a patient's immune system is ready to respond to treatment. The scientists created a scoring system based on these eRNAs that was better at predicting treatment success than previous methods using regular genes. They also used this system to identify existing drugs that could be combined with immunotherapy to make it more effective, and tested one such combination successfully in mice with brain tumors. |
| CancerRes | 5 | 10.1158/0008-5472.CAN-24-2292 | Gemini | Non-coding RNA transcribed from active enhancers, known as enhancer RNA (eRNA), is a critical element in gene regulation with a highly specific expression pattern in the regulatory networks of tumor-infiltrating cells. Therefore, eRNA signatures could potentially be applied to represent antitumor immune cells and to improve cancer immunotherapy. In this study, we identified thousands of eRNAs that were significantly correlated with infiltrating immune cell abundance in more than 10,000 patient samples across a variety of cancer types. The expression of these eRNAs was mediated by transcription factors with high expression in antitumor immune cells, as identified through single-cell assays. An eRNA immunotherapy signature (eRIS) developed using the antitumor eRNAs was highly associated with the objective response rate of immunotherapy and was elevated in patients who benefited from immune checkpoint blockade treatment. In comparison with a signature based on protein-coding genes, the eRIS was more effective in predicting the response to immunotherapy. Integration of the eRIS with pharmacogenomic data revealed hundreds of anticancer drugs that have the potential to enhance immunotherapy efficacy. Finally, treatment of a mouse model of IDH-mutant glioma with the histone deacetylase inhibitor vorinostat improved the effects of anti-PD-1 immunotherapy through increased abundance of infiltrating immune cells. Taken together, this study developed an eRIS with demonstrated efficacy in predicting immunotherapy response and used the eRIS to identify a series of effective combination drugs, thus highlighting the clinical utility of the eRIS in immunotherapy enhancement.Significance: An eRNA immunotherapy signature developed using antitumor eRNAs in tumor-infiltrating immune cells improves the prediction of response to immunotherapy and identifies a series of effective drug combinations to enhance immunotherapy efficacy. | Scientists discovered a new way to predict how well cancer immunotherapy will work by studying a special type of RNA called enhancer RNA (eRNA). They found specific eRNAs that are linked to the presence of immune cells fighting a tumor. This new eRNA immunotherapy signature (eRIS) was better at predicting which patients would benefit from treatment than previous methods. Using this signature, they also identified drugs that could be combined with immunotherapy to make it more effective. For example, in a mouse study, combining an existing drug (vorinostat) with immunotherapy improved the results by increasing the number of tumor-fighting immune cells. This shows that the eRIS can help doctors better predict treatment success and find new drug combinations to improve outcomes for cancer patients. | Scientists created a new way to predict how well cancer patients will respond to immunotherapy. They did this by looking at a specific type of non-coding RNA, called enhancer RNA (eRNA), which comes from immune cells that have infiltrated the tumor. This new eRNA immunotherapy signature (eRIS) was better at predicting treatment success than previous methods. Using this signature, they found several existing drugs that could potentially be combined with immunotherapy to make it even more effective. They tested one of these combinations, a drug called vorinostat with anti-PD-1 therapy, in mice with brain tumors and found it improved the treatment's results by increasing the number of immune cells in the tumor. This research shows that eRNA can be a valuable tool for improving cancer treatment. | In this study, researchers found that certain pieces of non-coding RNA, called enhancer RNAs (eRNAs), can act as a reliable signature for predicting how well a patient will respond to cancer immunotherapy. They created an eRNA immunotherapy signature (eRIS) by analyzing thousands of patient samples. This signature was better at predicting treatment success than methods based on protein-coding genes. By using the eRIS, they identified hundreds of drugs that could potentially be combined with immunotherapy to improve its effectiveness. For example, they found that a drug called vorinostat, when used with anti-PD-1 therapy, helped increase the number of immune cells fighting a tumor in a mouse model. This research shows that eRNA signatures are a powerful tool for predicting immunotherapy outcomes and finding new combination therapies. |
| CancerRes | 5 | 10.1158/0008-5472.CAN-24-2293 | DeepSeek | Non-coding RNA transcribed from active enhancers, known as enhancer RNA (eRNA), is a critical element in gene regulation with a highly specific expression pattern in the regulatory networks of tumor-infiltrating cells. Therefore, eRNA signatures could potentially be applied to represent antitumor immune cells and to improve cancer immunotherapy. In this study, we identified thousands of eRNAs that were significantly correlated with infiltrating immune cell abundance in more than 10,000 patient samples across a variety of cancer types. The expression of these eRNAs was mediated by transcription factors with high expression in antitumor immune cells, as identified through single-cell assays. An eRNA immunotherapy signature (eRIS) developed using the antitumor eRNAs was highly associated with the objective response rate of immunotherapy and was elevated in patients who benefited from immune checkpoint blockade treatment. In comparison with a signature based on protein-coding genes, the eRIS was more effective in predicting the response to immunotherapy. Integration of the eRIS with pharmacogenomic data revealed hundreds of anticancer drugs that have the potential to enhance immunotherapy efficacy. Finally, treatment of a mouse model of IDH-mutant glioma with the histone deacetylase inhibitor vorinostat improved the effects of anti-PD-1 immunotherapy through increased abundance of infiltrating immune cells. Taken together, this study developed an eRIS with demonstrated efficacy in predicting immunotherapy response and used the eRIS to identify a series of effective combination drugs, thus highlighting the clinical utility of the eRIS in immunotherapy enhancement.Significance: An eRNA immunotherapy signature developed using antitumor eRNAs in tumor-infiltrating immune cells improves the prediction of response to immunotherapy and identifies a series of effective drug combinations to enhance immunotherapy efficacy. | This study found that a type of non-coding RNA called enhancer RNA (eRNA), which is active in immune cells within tumors, can help predict how well cancer patients will respond to immunotherapy. Researchers created an eRNA-based signature (eRIS) that outperformed protein-coding gene signatures in identifying patients likely to benefit from treatments like immune checkpoint blockers. They also used eRIS to discover hundreds of drugs that could boost immunotherapy effectiveness, including vorinostat, which improved anti-PD-1 treatment in mice by increasing immune cell activity. These findings suggest eRIS could guide better immunotherapy strategies for cancer patients. | This study found that a type of non-coding RNA called enhancer RNA (eRNA), produced in immune cells within tumors, can help predict how well cancer patients will respond to immunotherapy. Researchers created an eRNA-based signature (eRIS) that outperformed protein-coding gene signatures in identifying patients likely to benefit from immune checkpoint blockade. They also used this signature to discover hundreds of potential drug combinations—like the HDAC inhibitor vorinostat—that could boost immunotherapy effectiveness, as shown in a mouse glioma model. This eRIS tool could improve personalized cancer treatment by guiding immunotherapy decisions and combination therapies. | This study found that a type of non-coding RNA called enhancer RNA (eRNA), produced in immune cells within tumors, can help predict how well cancer patients will respond to immunotherapy. By analyzing eRNAs from over 10,000 patients, researchers created a signature (eRIS) that outperformed protein-based markers in identifying who would benefit from treatments like immune checkpoint blockers. They also used this signature to discover hundreds of drugs—like vorinostat—that could boost immunotherapy effectiveness, successfully testing one in mice with brain tumors. This eRIS tool could improve personalized cancer treatment by guiding therapy choices and drug combinations. |
| CancerRes | 5 | 10.1158/0008-5472.CAN-24-2294 | Grok | Non-coding RNA transcribed from active enhancers, known as enhancer RNA (eRNA), is a critical element in gene regulation with a highly specific expression pattern in the regulatory networks of tumor-infiltrating cells. Therefore, eRNA signatures could potentially be applied to represent antitumor immune cells and to improve cancer immunotherapy. In this study, we identified thousands of eRNAs that were significantly correlated with infiltrating immune cell abundance in more than 10,000 patient samples across a variety of cancer types. The expression of these eRNAs was mediated by transcription factors with high expression in antitumor immune cells, as identified through single-cell assays. An eRNA immunotherapy signature (eRIS) developed using the antitumor eRNAs was highly associated with the objective response rate of immunotherapy and was elevated in patients who benefited from immune checkpoint blockade treatment. In comparison with a signature based on protein-coding genes, the eRIS was more effective in predicting the response to immunotherapy. Integration of the eRIS with pharmacogenomic data revealed hundreds of anticancer drugs that have the potential to enhance immunotherapy efficacy. Finally, treatment of a mouse model of IDH-mutant glioma with the histone deacetylase inhibitor vorinostat improved the effects of anti-PD-1 immunotherapy through increased abundance of infiltrating immune cells. Taken together, this study developed an eRIS with demonstrated efficacy in predicting immunotherapy response and used the eRIS to identify a series of effective combination drugs, thus highlighting the clinical utility of the eRIS in immunotherapy enhancement.Significance: An eRNA immunotherapy signature developed using antitumor eRNAs in tumor-infiltrating immune cells improves the prediction of response to immunotherapy and identifies a series of effective drug combinations to enhance immunotherapy efficacy. | This study explored enhancer RNAs (eRNAs), which are special molecules that help control genes and are linked to immune cells fighting tumors. By analyzing over 10,000 patient samples from various cancers, researchers identified eRNAs that are active in antitumor immune cells and created an eRNA immunotherapy signature (eRIS). This signature predicts how well patients will respond to immunotherapy better than traditional gene-based methods. The study also found that combining immunotherapy with certain drugs, like the histone deacetylase inhibitor vorinostat, boosts immune cell activity and improves treatment outcomes in a mouse model of brain cancer, showing the potential of eRIS to enhance cancer immunotherapy. | This study explored enhancer RNAs (eRNAs), which are molecules that help control genes and are linked to immune cells fighting tumors. Researchers analyzed over 10,000 patient samples across different cancers and found eRNAs that were strongly tied to these immune cells. They created an eRNA immunotherapy signature (eRIS) that predicts how well immunotherapy works better than protein-based signatures. The eRIS also helped identify drugs that could boost immunotherapy. In a mouse model of brain cancer, a drug called vorinostat improved immunotherapy by increasing immune cell activity, showing the potential of eRIS to guide better cancer treatments. | Researchers studied enhancer RNAs (eRNAs), which are molecules that help control genes and are linked to immune cells fighting cancer. They analyzed over 10,000 patient samples across different cancers and found eRNAs that are active in antitumor immune cells. Using these, they created an eRNA immunotherapy signature (eRIS) that predicts how well cancer patients respond to immunotherapy better than traditional gene-based methods. The eRIS also helped identify drugs that could boost immunotherapy effectiveness. In a mouse model of brain cancer, combining a drug called vorinostat with immunotherapy increased immune cell activity, showing that eRIS could guide better treatment strategies. |
| CancerRes | 1 | 10.1016/S1470-2045(25)00211-7 | Open AI | Background Oestrogen plus progestin hormone therapy is an established risk factor for breast cancer in postmenopausal women. We examined the less well-studied association between exogenous hormones and breast cancer in young women, who might use hormone therapy after gynaecological surgery or to relieve perimenopausal symptoms. Methods In this pooled cohort analysis, we investigated the relationship between exogenous hormones and breast cancer in young women using data from 10-13 prospective cohorts from North America, Europe, Asia, and Australia. The participating cohorts followed up women for incident breast cancer until age 55 years. We used cohort-stratified, multivariable-adjusted Cox proportional hazards regression to estimate hazard ratios (HRs) and 95% CI for associations of hormone therapy with incident young-onset breast cancer. We also estimated risk differences based on cumulative risk until age 55 years. Findings We included 459 476 women aged 16-54 years (mean 42 center dot 0 years [IQR 35 center dot 5-49 center dot 2]), of whom 8455 (2%) developed young-onset breast cancer (diagnosed before age 55 years; median follow-up 78 years [5 center dot 2-11 center dot 2]). Overall, 15% of participants reported using hormone therapy, with oestrogen plus progestin hormone therapy (6%) and unopposed oestrogen (5%) being the most common types. Cumulative risk of young-onset breast cancer was 41% in non-users. Hormone therapy of any type was not associated with incident young-onset breast cancer (HR 096 [95% CI 088 to 104]), but ever oestrogen hormone therapy use was inversely associated (086 [075 to 098]; risk difference-05% [-10 to-00]). The HR for ever oestrogen plus progestin hormone therapy and young-onset breast cancer was 110 (098 to 124), with positive associations observed for long-term use (118 [101 to 138] for >2 years) and use among women without hysterectomy or bilateral oophorectomy (115 [102 to 131]). Oestrogen hormone therapy and young-onset breast cancer association was similar for all breast cancer subtypes, but oestrogen plus progestin hormone therapy was more strongly associated with oestrogen receptor negative (144 [111 to 188]) and triple-negative disease (150 [102 to 220]) than with other subtypes. Interpretation Oestrogen hormone therapy use was inversely associated with young-onset breast cancer, and oestrogen plus progestin hormone therapy was associated with higher young-onset breast cancer incidence among women with intact uterus and ovaries. These findings largely parallel results from studies of hormone use and later-onset breast cancer and provide novel evidence for establishing clinical recommendations among younger women. | This large study of nearly 460,000 women aged 16–54 found that overall hormone therapy use was not linked to higher rates of breast cancer before age 55. However, women who used oestrogen-only therapy had a slightly lower risk, while long-term use of oestrogen plus progestin—especially in women with an intact uterus and ovaries—was linked to a higher risk, particularly for more aggressive breast cancer types. The results are similar to patterns seen in older women and may help guide treatment decisions for younger women. | This large international study followed nearly 460,000 women under age 55 to explore how hormone therapy affects breast cancer risk in younger women. Overall, hormone therapy did not increase the risk of developing breast cancer before age 55. In fact, estrogen-only therapy was linked to a slightly lower risk, while estrogen plus progestin therapy showed a higher risk, especially for women with intact uterus and ovaries, for long-term use, and for more aggressive cancer types. These results are similar to patterns seen in older women and may help guide treatment decisions for younger patients. | This large international study followed over 450,000 women under age 55 to see if hormone therapy affects the risk of early-onset breast cancer. Overall, hormone therapy use was not linked to higher breast cancer risk. Oestrogen-only therapy was linked to a slightly lower risk, while oestrogen plus progestin therapy was linked to a higher risk—especially with long-term use and in women who still had their uterus and ovaries. The increased risk was more pronounced for aggressive cancer types like triple-negative breast cancer. These results are similar to findings in older women and may help guide treatment choices for younger women. |
| CancerRes | 1 | 10.1016/S1470-2045(25)00211-8 | Claude | Background Oestrogen plus progestin hormone therapy is an established risk factor for breast cancer in postmenopausal women. We examined the less well-studied association between exogenous hormones and breast cancer in young women, who might use hormone therapy after gynaecological surgery or to relieve perimenopausal symptoms. Methods In this pooled cohort analysis, we investigated the relationship between exogenous hormones and breast cancer in young women using data from 10-13 prospective cohorts from North America, Europe, Asia, and Australia. The participating cohorts followed up women for incident breast cancer until age 55 years. We used cohort-stratified, multivariable-adjusted Cox proportional hazards regression to estimate hazard ratios (HRs) and 95% CI for associations of hormone therapy with incident young-onset breast cancer. We also estimated risk differences based on cumulative risk until age 55 years. Findings We included 459 476 women aged 16-54 years (mean 42 center dot 0 years [IQR 35 center dot 5-49 center dot 2]), of whom 8455 (2%) developed young-onset breast cancer (diagnosed before age 55 years; median follow-up 78 years [5 center dot 2-11 center dot 2]). Overall, 15% of participants reported using hormone therapy, with oestrogen plus progestin hormone therapy (6%) and unopposed oestrogen (5%) being the most common types. Cumulative risk of young-onset breast cancer was 41% in non-users. Hormone therapy of any type was not associated with incident young-onset breast cancer (HR 096 [95% CI 088 to 104]), but ever oestrogen hormone therapy use was inversely associated (086 [075 to 098]; risk difference-05% [-10 to-00]). The HR for ever oestrogen plus progestin hormone therapy and young-onset breast cancer was 110 (098 to 124), with positive associations observed for long-term use (118 [101 to 138] for >2 years) and use among women without hysterectomy or bilateral oophorectomy (115 [102 to 131]). Oestrogen hormone therapy and young-onset breast cancer association was similar for all breast cancer subtypes, but oestrogen plus progestin hormone therapy was more strongly associated with oestrogen receptor negative (144 [111 to 188]) and triple-negative disease (150 [102 to 220]) than with other subtypes. Interpretation Oestrogen hormone therapy use was inversely associated with young-onset breast cancer, and oestrogen plus progestin hormone therapy was associated with higher young-onset breast cancer incidence among women with intact uterus and ovaries. These findings largely parallel results from studies of hormone use and later-onset breast cancer and provide novel evidence for establishing clinical recommendations among younger women. | This large study looked at whether hormone therapy increases breast cancer risk in women under 55. Researchers followed nearly 460,000 women from multiple countries and found that taking estrogen alone actually seemed to slightly lower breast cancer risk. However, taking estrogen combined with progestin (another hormone) increased breast cancer risk, especially when used for more than 2 years or in women who still had their uterus and ovaries. The combined hormone therapy was particularly linked to more aggressive types of breast cancer. These results are similar to what's already known about hormone therapy in older women after menopause, helping doctors make better recommendations for younger women considering hormone treatment. | This large study looked at how hormone therapy affects breast cancer risk in women under 55. Researchers followed nearly 460,000 women and found that taking estrogen alone actually slightly reduced breast cancer risk, while taking estrogen combined with progestin (another hormone) increased risk, especially when used for more than 2 years or in women who still had their uterus and ovaries. The estrogen-progestin combination was particularly linked to more aggressive types of breast cancer. Overall, about 4% of women in the study developed breast cancer before age 55, and these findings are similar to what's already known about hormone therapy and breast cancer in older women. | This large study looked at how hormone therapy affects breast cancer risk in women under 55. Researchers followed nearly 460,000 women from multiple countries and found that 2% developed breast cancer before age 55. Women who used estrogen-only hormone therapy actually had a slightly lower risk of breast cancer. However, women who used combined estrogen plus progestin therapy (especially for more than 2 years or those who still had their uterus and ovaries) had a higher risk of developing breast cancer, particularly aggressive types like triple-negative breast cancer. These findings are similar to what's already known about hormone therapy and breast cancer in older women, helping doctors make better treatment recommendations for younger women. |
| CancerRes | 1 | 10.1016/S1470-2045(25)00211-9 | Gemini | Background Oestrogen plus progestin hormone therapy is an established risk factor for breast cancer in postmenopausal women. We examined the less well-studied association between exogenous hormones and breast cancer in young women, who might use hormone therapy after gynaecological surgery or to relieve perimenopausal symptoms. Methods In this pooled cohort analysis, we investigated the relationship between exogenous hormones and breast cancer in young women using data from 10-13 prospective cohorts from North America, Europe, Asia, and Australia. The participating cohorts followed up women for incident breast cancer until age 55 years. We used cohort-stratified, multivariable-adjusted Cox proportional hazards regression to estimate hazard ratios (HRs) and 95% CI for associations of hormone therapy with incident young-onset breast cancer. We also estimated risk differences based on cumulative risk until age 55 years. Findings We included 459 476 women aged 16-54 years (mean 42 center dot 0 years [IQR 35 center dot 5-49 center dot 2]), of whom 8455 (2%) developed young-onset breast cancer (diagnosed before age 55 years; median follow-up 78 years [5 center dot 2-11 center dot 2]). Overall, 15% of participants reported using hormone therapy, with oestrogen plus progestin hormone therapy (6%) and unopposed oestrogen (5%) being the most common types. Cumulative risk of young-onset breast cancer was 41% in non-users. Hormone therapy of any type was not associated with incident young-onset breast cancer (HR 096 [95% CI 088 to 104]), but ever oestrogen hormone therapy use was inversely associated (086 [075 to 098]; risk difference-05% [-10 to-00]). The HR for ever oestrogen plus progestin hormone therapy and young-onset breast cancer was 110 (098 to 124), with positive associations observed for long-term use (118 [101 to 138] for >2 years) and use among women without hysterectomy or bilateral oophorectomy (115 [102 to 131]). Oestrogen hormone therapy and young-onset breast cancer association was similar for all breast cancer subtypes, but oestrogen plus progestin hormone therapy was more strongly associated with oestrogen receptor negative (144 [111 to 188]) and triple-negative disease (150 [102 to 220]) than with other subtypes. Interpretation Oestrogen hormone therapy use was inversely associated with young-onset breast cancer, and oestrogen plus progestin hormone therapy was associated with higher young-onset breast cancer incidence among women with intact uterus and ovaries. These findings largely parallel results from studies of hormone use and later-onset breast cancer and provide novel evidence for establishing clinical recommendations among younger women. | The study looked at how hormone therapy affects the risk of breast cancer in younger women (under age 55). Researchers found that using hormone therapy containing only estrogen was linked to a slightly lower risk of breast cancer. However, using hormone therapy that combines both estrogen and progestin was linked to a higher risk, especially for women who used it for more than two years and still had their uterus and ovaries. This increased risk was particularly noticeable for more aggressive types of breast cancer, such as triple-negative breast cancer. | This study, which looked at a large number of young women, found that using hormone therapy with just estrogen was associated with a lower risk of breast cancer. However, using hormone therapy that combined estrogen and progestin was linked to a higher risk of breast cancer, especially in women who still had their uterus and ovaries. This increased risk was particularly notable for certain aggressive breast cancer types, such as estrogen receptor-negative and triple-negative breast cancer. | This study, which looked at data from nearly half a million women under the age of 55, found that the use of hormone therapy is linked to the risk of developing breast cancer. Specifically, using estrogen-only hormone therapy was associated with a lower risk of young-onset breast cancer. However, using a combination of estrogen and progestin hormone therapy was associated with a higher risk, particularly for those with a uterus and ovaries and for those who used it for more than two years. This combination therapy was also found to be more strongly linked to specific, aggressive types of breast cancer, such as triple-negative breast cancer. These findings are similar to what is known about hormone therapy and later-onset breast cancer, and they provide important information for doctors and younger women to consider. |
| CancerRes | 1 | 10.1016/S1470-2045(25)00211-10 | DeepSeek | Background Oestrogen plus progestin hormone therapy is an established risk factor for breast cancer in postmenopausal women. We examined the less well-studied association between exogenous hormones and breast cancer in young women, who might use hormone therapy after gynaecological surgery or to relieve perimenopausal symptoms. Methods In this pooled cohort analysis, we investigated the relationship between exogenous hormones and breast cancer in young women using data from 10-13 prospective cohorts from North America, Europe, Asia, and Australia. The participating cohorts followed up women for incident breast cancer until age 55 years. We used cohort-stratified, multivariable-adjusted Cox proportional hazards regression to estimate hazard ratios (HRs) and 95% CI for associations of hormone therapy with incident young-onset breast cancer. We also estimated risk differences based on cumulative risk until age 55 years. Findings We included 459 476 women aged 16-54 years (mean 42 center dot 0 years [IQR 35 center dot 5-49 center dot 2]), of whom 8455 (2%) developed young-onset breast cancer (diagnosed before age 55 years; median follow-up 78 years [5 center dot 2-11 center dot 2]). Overall, 15% of participants reported using hormone therapy, with oestrogen plus progestin hormone therapy (6%) and unopposed oestrogen (5%) being the most common types. Cumulative risk of young-onset breast cancer was 41% in non-users. Hormone therapy of any type was not associated with incident young-onset breast cancer (HR 096 [95% CI 088 to 104]), but ever oestrogen hormone therapy use was inversely associated (086 [075 to 098]; risk difference-05% [-10 to-00]). The HR for ever oestrogen plus progestin hormone therapy and young-onset breast cancer was 110 (098 to 124), with positive associations observed for long-term use (118 [101 to 138] for >2 years) and use among women without hysterectomy or bilateral oophorectomy (115 [102 to 131]). Oestrogen hormone therapy and young-onset breast cancer association was similar for all breast cancer subtypes, but oestrogen plus progestin hormone therapy was more strongly associated with oestrogen receptor negative (144 [111 to 188]) and triple-negative disease (150 [102 to 220]) than with other subtypes. Interpretation Oestrogen hormone therapy use was inversely associated with young-onset breast cancer, and oestrogen plus progestin hormone therapy was associated with higher young-onset breast cancer incidence among women with intact uterus and ovaries. These findings largely parallel results from studies of hormone use and later-onset breast cancer and provide novel evidence for establishing clinical recommendations among younger women. | This study looked at whether hormone therapy (HT) increases breast cancer risk in women under 55. It pooled data from over 450,000 women across multiple countries and found that overall, HT did not raise breast cancer risk. However, estrogen-only HT was linked to a slightly lower risk, while estrogen-plus-progestin HT was tied to a higher risk—especially with long-term use or in women who still had their uterus and ovaries. The risk was also stronger for certain aggressive breast cancer types. These results align with findings in older women and can help guide HT recommendations for younger women. | This study looked at whether hormone therapy (HT) increases breast cancer risk in women under 55. It pooled data from over 450,000 women across multiple countries and found that overall, HT did not raise breast cancer risk. However, estrogen-only HT was linked to a slightly lower risk, while estrogen-progestin HT was tied to a higher risk—especially with long-term use or in women who still had their uterus and ovaries. The risk was strongest for certain aggressive breast cancer types. These findings align with older women’s HT risks and can help guide younger women’s treatment choices. | This study looked at whether hormone therapy (HT) increases breast cancer risk in women under 55. It pooled data from over 450,000 women across multiple countries and found that overall, HT did not raise breast cancer risk. However, estrogen-only HT was linked to a slightly lower risk, while estrogen-progestin HT was tied to a higher risk—especially with long-term use or in women who still had their uterus and ovaries. The risk was strongest for aggressive breast cancer types (like triple-negative). These findings suggest that, similar to older women, younger women using combined HT may face a small increased risk of breast cancer. |
| CancerRes | 1 | 10.1016/S1470-2045(25)00211-11 | Grok | Background Oestrogen plus progestin hormone therapy is an established risk factor for breast cancer in postmenopausal women. We examined the less well-studied association between exogenous hormones and breast cancer in young women, who might use hormone therapy after gynaecological surgery or to relieve perimenopausal symptoms. Methods In this pooled cohort analysis, we investigated the relationship between exogenous hormones and breast cancer in young women using data from 10-13 prospective cohorts from North America, Europe, Asia, and Australia. The participating cohorts followed up women for incident breast cancer until age 55 years. We used cohort-stratified, multivariable-adjusted Cox proportional hazards regression to estimate hazard ratios (HRs) and 95% CI for associations of hormone therapy with incident young-onset breast cancer. We also estimated risk differences based on cumulative risk until age 55 years. Findings We included 459 476 women aged 16-54 years (mean 42 center dot 0 years [IQR 35 center dot 5-49 center dot 2]), of whom 8455 (2%) developed young-onset breast cancer (diagnosed before age 55 years; median follow-up 78 years [5 center dot 2-11 center dot 2]). Overall, 15% of participants reported using hormone therapy, with oestrogen plus progestin hormone therapy (6%) and unopposed oestrogen (5%) being the most common types. Cumulative risk of young-onset breast cancer was 41% in non-users. Hormone therapy of any type was not associated with incident young-onset breast cancer (HR 096 [95% CI 088 to 104]), but ever oestrogen hormone therapy use was inversely associated (086 [075 to 098]; risk difference-05% [-10 to-00]). The HR for ever oestrogen plus progestin hormone therapy and young-onset breast cancer was 110 (098 to 124), with positive associations observed for long-term use (118 [101 to 138] for >2 years) and use among women without hysterectomy or bilateral oophorectomy (115 [102 to 131]). Oestrogen hormone therapy and young-onset breast cancer association was similar for all breast cancer subtypes, but oestrogen plus progestin hormone therapy was more strongly associated with oestrogen receptor negative (144 [111 to 188]) and triple-negative disease (150 [102 to 220]) than with other subtypes. Interpretation Oestrogen hormone therapy use was inversely associated with young-onset breast cancer, and oestrogen plus progestin hormone therapy was associated with higher young-onset breast cancer incidence among women with intact uterus and ovaries. These findings largely parallel results from studies of hormone use and later-onset breast cancer and provide novel evidence for establishing clinical recommendations among younger women. | A large study of 459,476 women aged 16-54 from multiple countries found that hormone therapy affects the risk of breast cancer in young women differently depending on the type used. Overall, hormone therapy didn’t significantly change the risk of breast cancer before age 55, but using only oestrogen slightly lowered the risk, while oestrogen plus progestin increased the risk, especially in women with an intact uterus and ovaries or those using it for over two years. This increase was stronger for certain aggressive breast cancer types. These findings help guide hormone therapy recommendations for younger women. | A large study of 459,476 women aged 16-54 from multiple countries found that hormone therapy affects the risk of breast cancer in young women differently depending on the type used. Overall, hormone therapy didn’t significantly change the risk of breast cancer before age 55, but using only oestrogen slightly lowered the risk, while oestrogen plus progestin slightly increased it, especially in women with long-term use or those who still had their uterus and ovaries. The increased risk was stronger for certain aggressive breast cancer types. These findings help guide hormone therapy recommendations for younger women. | A study involving 459,476 women aged 16-54 from various global cohorts found that hormone therapy's impact on young-onset breast cancer (diagnosed before age 55) varies by type. Overall, hormone therapy didn’t significantly increase breast cancer risk, but estrogen-only therapy slightly lowered it, while estrogen plus progestin therapy slightly raised it, especially in women with long-term use or those who hadn’t had a hysterectomy or ovary removal. The risk was higher for certain breast cancer types, like estrogen receptor-negative and triple-negative cancers. These findings help guide hormone therapy recommendations for younger women. |
| CancerRes | 2 | 10.1016/S1470-2045(25)00139-2 | Open AI | Background Cell-free, circulating tumour DNA (ctDNA) is an established measure of minimal residual disease; however, it is not utilised in melanoma management. We investigated whether ctDNA measurements could predict survival outcomes during adjuvant targeted therapy or placebo treatment in stage III melanoma, thereby identifying patients at high risk and low risk of recurrence. Methods Analytically validated mutation-specific droplet digital PCR assays were used to measure BRAF(V600E) or BRAF(V600K) ctDNA in patients aged 18 years or older who were enrolled in the COMBI-AD trial, which was a double-blind, randomised, phase 3 study of oral dabrafenib (150 mg twice daily) plus oral trametinib (2 mg once daily) combination therapy versus two matched placebos in resected BRAF(V600)-mutant stage III melanoma. Patients were screened for enrolment between Jan 31, 2013, and Dec 11, 2014, had an Eastern Cooperative Oncology Group performance status of 0 or 1, and were randomly assigned (1:1) to the two treatment groups. The primary endpoint was recurrence-free survival, and the results from final analysis have been previously published and will not be described here. Biomarker analysis was a prespecified exploratory endpoint and performed in the intention-to-treat population. We compared associations between survival outcomes and baseline (post-resection) ctDNA copies per mL, tumour mutational burden and interferon gamma (IFNG) gene expression. In a subset of patients, ctDNA quantities during follow-up or at recurrence were measured. The trial is registered with ClinicalTrials.gov, NCT01682083, and has been completed. Findings Baseline plasma samples were available for 597 of 870 patients (331 male patients and 266 female patients) and samples for assessing the ctDNA positivity rate at landmark follow-up timepoints of 3 months, 6 months, 9 months, and 12 months after treatment initiation were available for 94 of 870 patients. Additionally, samples were available from 118 of 870 patients within a 2-month timeframe before or after clinical or radiographic recurrence. Median follow-up for the biomarker analyses was 60 months (IQR 39-66) in the combination therapy group and 58 months (21-66) for the placebo group. ctDNA was detectable in 79 (13%) of 597 baseline samples. ctDNA positivity rate and mutant copies per mL plasma were significantly higher in patients with higher disease substages. As a binary variable, ctDNA detection was associated with worse recurrence-free survival (placebo group: median 371 months [95% CI 239-689] vs 2441 months [1728-4313]; hazard ratio [HR] 291 [95% CI 199-425], p<00001); combination therapy group: median 1659 months [95% CI 1202-2680] vs 6811 months [5036-not reached]; HR 298 [195-454], p<00001) and overall survival (placebo group: median 3390 months [1396-not reached] vs not reached; HR 335 [201-555], p<00001); combination therapy group: median 4031 months [2490-not reached] vs not reached; HR 427 [250-727], p<00001) in the placebo group and combination therapy groups. Baseline ctDNA was more strongly associated with survival outcomes than IFNG gene expression or tumour mutational burden. Patients with adverse longitudinal ctDNA kinetics (molecular relapse or persistently positive) had markedly shorter median recurrence-free survival (831 months [95% CI 539-1220] and 532 months [279-not reached], respectively) compared with patients with favourable kinetics (ie, undetectable after positive baseline result: 1925 months [1639-not reached]; and durable undetectable: not reached [3844-not reached], p<00001). Interpretation Droplet digital PCR measurements of ctDNA to assess minimal residual disease before adjuvant targeted therapy and during follow-up can identify patients at high risk of early recurrence. Additional studies using ctDNA measurements to guide therapeutic interventions might lead to improvements in the management of resected stage III melanoma. | This study looked at whether measuring tiny fragments of tumor DNA in the blood (ctDNA) could help predict which patients with stage III melanoma are more likely to have their cancer return after surgery. Using a highly sensitive test, researchers found that patients with detectable ctDNA before or during treatment had a much higher risk of cancer coming back sooner and shorter overall survival compared to those without detectable ctDNA. These blood tests were better at predicting outcomes than other biomarkers, and tracking ctDNA over time gave valuable information about a patient’s risk. The findings suggest ctDNA testing could help doctors identify high-risk patients and improve melanoma treatment strategies. | This study looked at whether measuring circulating tumor DNA (ctDNA) in the blood could help predict which patients with stage III melanoma are more likely to have their cancer return after surgery. Researchers measured ctDNA in patients who received either targeted therapy or a placebo. They found that patients with detectable ctDNA after surgery had a higher risk of early recurrence and worse overall survival, while those with undetectable ctDNA did much better. Tracking ctDNA over time also helped identify patients whose disease was returning. These results suggest that ctDNA testing could be a useful tool to guide treatment and follow-up in melanoma patients. | This study found that measuring circulating tumor DNA (ctDNA) in the blood of patients with stage III melanoma can help predict who is more likely to have the cancer come back after surgery. Patients with detectable ctDNA before or during treatment had shorter times without recurrence and lower overall survival, while those with no detectable ctDNA did better. ctDNA levels were a stronger predictor of outcomes than other measures like tumor mutations or gene expression. These results suggest that ctDNA testing could help identify high-risk patients and guide treatment decisions after melanoma surgery. |
| CancerRes | 2 | 10.1016/S1470-2045(25)00139-3 | Claude | Background Cell-free, circulating tumour DNA (ctDNA) is an established measure of minimal residual disease; however, it is not utilised in melanoma management. We investigated whether ctDNA measurements could predict survival outcomes during adjuvant targeted therapy or placebo treatment in stage III melanoma, thereby identifying patients at high risk and low risk of recurrence. Methods Analytically validated mutation-specific droplet digital PCR assays were used to measure BRAF(V600E) or BRAF(V600K) ctDNA in patients aged 18 years or older who were enrolled in the COMBI-AD trial, which was a double-blind, randomised, phase 3 study of oral dabrafenib (150 mg twice daily) plus oral trametinib (2 mg once daily) combination therapy versus two matched placebos in resected BRAF(V600)-mutant stage III melanoma. Patients were screened for enrolment between Jan 31, 2013, and Dec 11, 2014, had an Eastern Cooperative Oncology Group performance status of 0 or 1, and were randomly assigned (1:1) to the two treatment groups. The primary endpoint was recurrence-free survival, and the results from final analysis have been previously published and will not be described here. Biomarker analysis was a prespecified exploratory endpoint and performed in the intention-to-treat population. We compared associations between survival outcomes and baseline (post-resection) ctDNA copies per mL, tumour mutational burden and interferon gamma (IFNG) gene expression. In a subset of patients, ctDNA quantities during follow-up or at recurrence were measured. The trial is registered with ClinicalTrials.gov, NCT01682083, and has been completed. Findings Baseline plasma samples were available for 597 of 870 patients (331 male patients and 266 female patients) and samples for assessing the ctDNA positivity rate at landmark follow-up timepoints of 3 months, 6 months, 9 months, and 12 months after treatment initiation were available for 94 of 870 patients. Additionally, samples were available from 118 of 870 patients within a 2-month timeframe before or after clinical or radiographic recurrence. Median follow-up for the biomarker analyses was 60 months (IQR 39-66) in the combination therapy group and 58 months (21-66) for the placebo group. ctDNA was detectable in 79 (13%) of 597 baseline samples. ctDNA positivity rate and mutant copies per mL plasma were significantly higher in patients with higher disease substages. As a binary variable, ctDNA detection was associated with worse recurrence-free survival (placebo group: median 371 months [95% CI 239-689] vs 2441 months [1728-4313]; hazard ratio [HR] 291 [95% CI 199-425], p<00001); combination therapy group: median 1659 months [95% CI 1202-2680] vs 6811 months [5036-not reached]; HR 298 [195-454], p<00001) and overall survival (placebo group: median 3390 months [1396-not reached] vs not reached; HR 335 [201-555], p<00001); combination therapy group: median 4031 months [2490-not reached] vs not reached; HR 427 [250-727], p<00001) in the placebo group and combination therapy groups. Baseline ctDNA was more strongly associated with survival outcomes than IFNG gene expression or tumour mutational burden. Patients with adverse longitudinal ctDNA kinetics (molecular relapse or persistently positive) had markedly shorter median recurrence-free survival (831 months [95% CI 539-1220] and 532 months [279-not reached], respectively) compared with patients with favourable kinetics (ie, undetectable after positive baseline result: 1925 months [1639-not reached]; and durable undetectable: not reached [3844-not reached], p<00001). Interpretation Droplet digital PCR measurements of ctDNA to assess minimal residual disease before adjuvant targeted therapy and during follow-up can identify patients at high risk of early recurrence. Additional studies using ctDNA measurements to guide therapeutic interventions might lead to improvements in the management of resected stage III melanoma. | This study looked at whether measuring tiny pieces of tumor DNA floating in the blood (called ctDNA) could predict which melanoma patients would have their cancer come back after surgery. Researchers tested blood samples from nearly 600 patients with stage III melanoma who were receiving either targeted therapy drugs or placebo pills. They found that patients who had detectable tumor DNA in their blood were much more likely to have their cancer return quickly and had shorter survival times, regardless of whether they received the active drugs or placebo. The blood test was better at predicting outcomes than other tumor markers, suggesting it could help doctors identify high-risk patients who might need more aggressive treatment after surgery. | This study looked at whether measuring tiny pieces of tumor DNA floating in the blood (called ctDNA) could help predict which melanoma patients were more likely to have their cancer come back after surgery. Researchers tested blood samples from nearly 600 patients with stage III melanoma who were receiving either a combination drug treatment or placebo pills. They found that patients who had detectable tumor DNA in their blood after surgery were much more likely to have their cancer return and had shorter survival times, regardless of whether they received the actual drugs or placebo. The study suggests that this blood test could be a useful tool to identify high-risk patients who might need closer monitoring or different treatment approaches after melanoma surgery. | This study looked at whether measuring tiny pieces of tumor DNA floating in the blood (called ctDNA) could help predict which melanoma patients are most likely to have their cancer come back after surgery. Researchers tested blood samples from nearly 600 patients with stage III melanoma who were receiving either targeted therapy drugs or placebo pills. They found that patients who had detectable tumor DNA in their blood after surgery were much more likely to have their cancer return quickly and had shorter survival times, regardless of which treatment they received. The blood test was better at predicting outcomes than other methods, suggesting it could help doctors identify high-risk patients who might need more intensive monitoring or different treatments. |
| CancerRes | 2 | 10.1016/S1470-2045(25)00139-4 | Gemini | Background Cell-free, circulating tumour DNA (ctDNA) is an established measure of minimal residual disease; however, it is not utilised in melanoma management. We investigated whether ctDNA measurements could predict survival outcomes during adjuvant targeted therapy or placebo treatment in stage III melanoma, thereby identifying patients at high risk and low risk of recurrence. Methods Analytically validated mutation-specific droplet digital PCR assays were used to measure BRAF(V600E) or BRAF(V600K) ctDNA in patients aged 18 years or older who were enrolled in the COMBI-AD trial, which was a double-blind, randomised, phase 3 study of oral dabrafenib (150 mg twice daily) plus oral trametinib (2 mg once daily) combination therapy versus two matched placebos in resected BRAF(V600)-mutant stage III melanoma. Patients were screened for enrolment between Jan 31, 2013, and Dec 11, 2014, had an Eastern Cooperative Oncology Group performance status of 0 or 1, and were randomly assigned (1:1) to the two treatment groups. The primary endpoint was recurrence-free survival, and the results from final analysis have been previously published and will not be described here. Biomarker analysis was a prespecified exploratory endpoint and performed in the intention-to-treat population. We compared associations between survival outcomes and baseline (post-resection) ctDNA copies per mL, tumour mutational burden and interferon gamma (IFNG) gene expression. In a subset of patients, ctDNA quantities during follow-up or at recurrence were measured. The trial is registered with ClinicalTrials.gov, NCT01682083, and has been completed. Findings Baseline plasma samples were available for 597 of 870 patients (331 male patients and 266 female patients) and samples for assessing the ctDNA positivity rate at landmark follow-up timepoints of 3 months, 6 months, 9 months, and 12 months after treatment initiation were available for 94 of 870 patients. Additionally, samples were available from 118 of 870 patients within a 2-month timeframe before or after clinical or radiographic recurrence. Median follow-up for the biomarker analyses was 60 months (IQR 39-66) in the combination therapy group and 58 months (21-66) for the placebo group. ctDNA was detectable in 79 (13%) of 597 baseline samples. ctDNA positivity rate and mutant copies per mL plasma were significantly higher in patients with higher disease substages. As a binary variable, ctDNA detection was associated with worse recurrence-free survival (placebo group: median 371 months [95% CI 239-689] vs 2441 months [1728-4313]; hazard ratio [HR] 291 [95% CI 199-425], p<00001); combination therapy group: median 1659 months [95% CI 1202-2680] vs 6811 months [5036-not reached]; HR 298 [195-454], p<00001) and overall survival (placebo group: median 3390 months [1396-not reached] vs not reached; HR 335 [201-555], p<00001); combination therapy group: median 4031 months [2490-not reached] vs not reached; HR 427 [250-727], p<00001) in the placebo group and combination therapy groups. Baseline ctDNA was more strongly associated with survival outcomes than IFNG gene expression or tumour mutational burden. Patients with adverse longitudinal ctDNA kinetics (molecular relapse or persistently positive) had markedly shorter median recurrence-free survival (831 months [95% CI 539-1220] and 532 months [279-not reached], respectively) compared with patients with favourable kinetics (ie, undetectable after positive baseline result: 1925 months [1639-not reached]; and durable undetectable: not reached [3844-not reached], p<00001). Interpretation Droplet digital PCR measurements of ctDNA to assess minimal residual disease before adjuvant targeted therapy and during follow-up can identify patients at high risk of early recurrence. Additional studies using ctDNA measurements to guide therapeutic interventions might lead to improvements in the management of resected stage III melanoma. | Circulating tumor DNA, or ctDNA, is a type of DNA from cancer cells that circulates in the blood. This study found that measuring ctDNA in patients with resected stage III melanoma (a severe form of skin cancer) can predict who is more likely to have their cancer come back. The study used a highly sensitive test called droplet digital PCR to measure ctDNA levels both before and during treatment. The results showed that patients with detectable ctDNA at the start of the study, or whose ctDNA levels remained high over time, had a significantly higher risk of their cancer returning and shorter survival. This suggests that monitoring ctDNA could be a valuable tool for identifying patients who need closer monitoring or more aggressive treatment. | In a study on patients with a specific type of advanced melanoma that had been surgically removed, researchers looked at whether circulating tumor DNA (ctDNA) could predict how well patients would do after treatment. They found that finding ctDNA in the blood before treatment was a strong sign of a worse outcome, with patients being more likely to have their cancer return and having a shorter survival time. This was true for patients who received either the active combination therapy or a placebo. The study concluded that using this blood test to find ctDNA can help identify patients at high risk of their cancer coming back early. This suggests that ctDNA could be a useful tool for guiding future treatment decisions in melanoma. | Researchers in the COMBI-AD trial studied the use of a blood test for circulating tumor DNA (ctDNA) to predict how well patients with resected stage III melanoma would do. They found that patients who had detectable levels of ctDNA in their blood after surgery had a significantly higher risk of their cancer returning and shorter overall survival. This was true whether they received a placebo or the combination therapy of dabrafenib and trametinib. The study also showed that patients whose ctDNA levels remained detectable or reappeared after being undetectable had a very high risk of the cancer returning quickly. These findings suggest that monitoring ctDNA could be a valuable tool to identify patients who are at high risk of recurrence, potentially leading to better-tailored treatment strategies. |
| CancerRes | 2 | 10.1016/S1470-2045(25)00139-5 | DeepSeek | Background Cell-free, circulating tumour DNA (ctDNA) is an established measure of minimal residual disease; however, it is not utilised in melanoma management. We investigated whether ctDNA measurements could predict survival outcomes during adjuvant targeted therapy or placebo treatment in stage III melanoma, thereby identifying patients at high risk and low risk of recurrence. Methods Analytically validated mutation-specific droplet digital PCR assays were used to measure BRAF(V600E) or BRAF(V600K) ctDNA in patients aged 18 years or older who were enrolled in the COMBI-AD trial, which was a double-blind, randomised, phase 3 study of oral dabrafenib (150 mg twice daily) plus oral trametinib (2 mg once daily) combination therapy versus two matched placebos in resected BRAF(V600)-mutant stage III melanoma. Patients were screened for enrolment between Jan 31, 2013, and Dec 11, 2014, had an Eastern Cooperative Oncology Group performance status of 0 or 1, and were randomly assigned (1:1) to the two treatment groups. The primary endpoint was recurrence-free survival, and the results from final analysis have been previously published and will not be described here. Biomarker analysis was a prespecified exploratory endpoint and performed in the intention-to-treat population. We compared associations between survival outcomes and baseline (post-resection) ctDNA copies per mL, tumour mutational burden and interferon gamma (IFNG) gene expression. In a subset of patients, ctDNA quantities during follow-up or at recurrence were measured. The trial is registered with ClinicalTrials.gov, NCT01682083, and has been completed. Findings Baseline plasma samples were available for 597 of 870 patients (331 male patients and 266 female patients) and samples for assessing the ctDNA positivity rate at landmark follow-up timepoints of 3 months, 6 months, 9 months, and 12 months after treatment initiation were available for 94 of 870 patients. Additionally, samples were available from 118 of 870 patients within a 2-month timeframe before or after clinical or radiographic recurrence. Median follow-up for the biomarker analyses was 60 months (IQR 39-66) in the combination therapy group and 58 months (21-66) for the placebo group. ctDNA was detectable in 79 (13%) of 597 baseline samples. ctDNA positivity rate and mutant copies per mL plasma were significantly higher in patients with higher disease substages. As a binary variable, ctDNA detection was associated with worse recurrence-free survival (placebo group: median 371 months [95% CI 239-689] vs 2441 months [1728-4313]; hazard ratio [HR] 291 [95% CI 199-425], p<00001); combination therapy group: median 1659 months [95% CI 1202-2680] vs 6811 months [5036-not reached]; HR 298 [195-454], p<00001) and overall survival (placebo group: median 3390 months [1396-not reached] vs not reached; HR 335 [201-555], p<00001); combination therapy group: median 4031 months [2490-not reached] vs not reached; HR 427 [250-727], p<00001) in the placebo group and combination therapy groups. Baseline ctDNA was more strongly associated with survival outcomes than IFNG gene expression or tumour mutational burden. Patients with adverse longitudinal ctDNA kinetics (molecular relapse or persistently positive) had markedly shorter median recurrence-free survival (831 months [95% CI 539-1220] and 532 months [279-not reached], respectively) compared with patients with favourable kinetics (ie, undetectable after positive baseline result: 1925 months [1639-not reached]; and durable undetectable: not reached [3844-not reached], p<00001). Interpretation Droplet digital PCR measurements of ctDNA to assess minimal residual disease before adjuvant targeted therapy and during follow-up can identify patients at high risk of early recurrence. Additional studies using ctDNA measurements to guide therapeutic interventions might lead to improvements in the management of resected stage III melanoma. | This study looked at whether detecting tiny bits of tumor DNA (ctDNA) in blood could predict cancer recurrence in stage III melanoma patients after surgery. Using a sensitive test (droplet digital PCR), researchers found ctDNA in 13% of patients before starting treatment. Those with ctDNA had much worse outcomes, relapsing faster and surviving shorter, whether they received placebo or targeted therapy (dabrafenib + trametinib). Tracking ctDNA over time also helped identify high-risk patients, as those with persistent or rising ctDNA relapsed sooner. The results suggest ctDNA testing could help spot patients needing stronger treatment after surgery. | This study looked at whether detecting tiny bits of tumor DNA (ctDNA) in blood could predict cancer recurrence in stage III melanoma patients after surgery and adjuvant therapy. Using a sensitive test (droplet digital PCR), researchers found that patients with detectable ctDNA before treatment or during follow-up had a much higher risk of their cancer returning and worse survival outcomes compared to those without ctDNA. The results suggest that monitoring ctDNA could help identify high-risk patients early, potentially guiding treatment decisions to improve outcomes. | This study looked at whether detecting tiny amounts of tumor DNA (ctDNA) in blood could predict cancer recurrence in stage III melanoma patients after surgery and adjuvant therapy. Using a sensitive test (droplet digital PCR), researchers found that patients with detectable ctDNA before treatment had a higher risk of their cancer returning and worse survival outcomes compared to those without ctDNA. Tracking ctDNA levels over time also helped identify patients at high risk of early relapse. The results suggest ctDNA testing could be a useful tool to guide treatment decisions in melanoma. |
| CancerRes | 2 | 10.1016/S1470-2045(25)00139-6 | Grok | Background Cell-free, circulating tumour DNA (ctDNA) is an established measure of minimal residual disease; however, it is not utilised in melanoma management. We investigated whether ctDNA measurements could predict survival outcomes during adjuvant targeted therapy or placebo treatment in stage III melanoma, thereby identifying patients at high risk and low risk of recurrence. Methods Analytically validated mutation-specific droplet digital PCR assays were used to measure BRAF(V600E) or BRAF(V600K) ctDNA in patients aged 18 years or older who were enrolled in the COMBI-AD trial, which was a double-blind, randomised, phase 3 study of oral dabrafenib (150 mg twice daily) plus oral trametinib (2 mg once daily) combination therapy versus two matched placebos in resected BRAF(V600)-mutant stage III melanoma. Patients were screened for enrolment between Jan 31, 2013, and Dec 11, 2014, had an Eastern Cooperative Oncology Group performance status of 0 or 1, and were randomly assigned (1:1) to the two treatment groups. The primary endpoint was recurrence-free survival, and the results from final analysis have been previously published and will not be described here. Biomarker analysis was a prespecified exploratory endpoint and performed in the intention-to-treat population. We compared associations between survival outcomes and baseline (post-resection) ctDNA copies per mL, tumour mutational burden and interferon gamma (IFNG) gene expression. In a subset of patients, ctDNA quantities during follow-up or at recurrence were measured. The trial is registered with ClinicalTrials.gov, NCT01682083, and has been completed. Findings Baseline plasma samples were available for 597 of 870 patients (331 male patients and 266 female patients) and samples for assessing the ctDNA positivity rate at landmark follow-up timepoints of 3 months, 6 months, 9 months, and 12 months after treatment initiation were available for 94 of 870 patients. Additionally, samples were available from 118 of 870 patients within a 2-month timeframe before or after clinical or radiographic recurrence. Median follow-up for the biomarker analyses was 60 months (IQR 39-66) in the combination therapy group and 58 months (21-66) for the placebo group. ctDNA was detectable in 79 (13%) of 597 baseline samples. ctDNA positivity rate and mutant copies per mL plasma were significantly higher in patients with higher disease substages. As a binary variable, ctDNA detection was associated with worse recurrence-free survival (placebo group: median 371 months [95% CI 239-689] vs 2441 months [1728-4313]; hazard ratio [HR] 291 [95% CI 199-425], p<00001); combination therapy group: median 1659 months [95% CI 1202-2680] vs 6811 months [5036-not reached]; HR 298 [195-454], p<00001) and overall survival (placebo group: median 3390 months [1396-not reached] vs not reached; HR 335 [201-555], p<00001); combination therapy group: median 4031 months [2490-not reached] vs not reached; HR 427 [250-727], p<00001) in the placebo group and combination therapy groups. Baseline ctDNA was more strongly associated with survival outcomes than IFNG gene expression or tumour mutational burden. Patients with adverse longitudinal ctDNA kinetics (molecular relapse or persistently positive) had markedly shorter median recurrence-free survival (831 months [95% CI 539-1220] and 532 months [279-not reached], respectively) compared with patients with favourable kinetics (ie, undetectable after positive baseline result: 1925 months [1639-not reached]; and durable undetectable: not reached [3844-not reached], p<00001). Interpretation Droplet digital PCR measurements of ctDNA to assess minimal residual disease before adjuvant targeted therapy and during follow-up can identify patients at high risk of early recurrence. Additional studies using ctDNA measurements to guide therapeutic interventions might lead to improvements in the management of resected stage III melanoma. | In a study called COMBI-AD, researchers used a special test called droplet digital PCR to measure tiny pieces of tumor DNA (ctDNA) in the blood of 597 patients with stage III melanoma after surgery. They checked if these DNA pieces could predict how long patients would stay cancer-free and survive while receiving either a combination of two drugs (dabrafenib and trametinib) or a placebo. They found that patients with detectable ctDNA at the start had a much higher chance of their cancer returning sooner and lower overall survival compared to those without detectable ctDNA. This test was better at predicting outcomes than other measures like tumor mutation burden or immune gene activity. The results suggest that checking ctDNA could help identify patients at high risk of cancer returning, which might guide better treatment choices in the future. | In a study involving patients with stage III melanoma who had their tumors surgically removed, researchers used a sensitive test called droplet digital PCR to measure circulating tumor DNA (ctDNA) in the blood to predict survival outcomes. The study, part of the COMBI-AD trial, included 597 patients treated with either a combination of dabrafenib and trametinib or a placebo. They found that patients with detectable ctDNA at the start had a higher risk of cancer returning and worse survival rates compared to those without detectable ctDNA. The test also showed that patients whose ctDNA levels increased or remained high during follow-up were more likely to have their cancer return sooner. This suggests that ctDNA testing could help identify high-risk patients who might need different treatments to prevent recurrence. | In a study involving patients with stage III melanoma, researchers used a precise test called droplet digital PCR to measure circulating tumour DNA (ctDNA) in blood samples to predict survival outcomes. The study, part of the COMBI-AD trial, included 597 patients who received either a combination of dabrafenib and trametinib or a placebo after surgery. They found that patients with detectable ctDNA before treatment had a higher risk of cancer returning and worse survival rates compared to those without detectable ctDNA. Tracking ctDNA over time also helped identify patients at risk of early recurrence, suggesting that ctDNA testing could guide future treatment to improve outcomes for these patients. |
| CancerRes | 3 | 10.1016/S1470-2045(24)00373-6 | Open AI | Background CAPItello-291 is an ongoing phase 3 trial in which capivasertib-fulvestrant significantly improved progression-free survival versus placebo-fulvestrant in patients with hormone receptor-positive, HER2-negative advanced breast cancer who had relapse or disease progression during or after aromatase inhibitor treatment, in both the overall population and in patients with PIK3CA, , AKT1, , or PTEN- altered tumours. This study further explored patient-reported health-related quality of life (HRQOL), functioning, symptoms, and symptom tolerability in CAPItello-291. Methods This phase 3, randomised, double-blind, placebo-controlled trial, which was conducted across 193 hospitals and cancer centres in 19 countries, enrolled women with any menopausal status or men, aged >= 18 years (>= 20 years in Japan), with hormone receptor-positive, HER2-negative locally advanced or metastatic breast cancer who had relapse or disease progression during or after treatment with an aromatase inhibitor, with or without previous cyclindependent kinase (CDK) 4 or 6 inhibitor therapy. Patients had an Eastern Cooperative Oncology Group/WHO performance score of 0 or 1 and could have received up to two previous lines of endocrine therapy and up to one previous line of chemotherapy for advanced disease. Patients were randomly assigned (1:1) using block randomisation (stratified according to the presence or absence of liver metastases, previous use of a CDK4/6 inhibitor [yes vs no], and geographical region) to receive oral capivasertib 400 mg (twice daily for 4 days, followed by 3 days off) plus intramuscular fulvestrant 500 mg (every 14 days for the first three injections, then every 28 days) or placebo with matching fulvestrant dosing. The dual primary endpoint of the trial was investigator-assessed progression-free survival assessed both in the overall population and among patients with PIK3CA, , AKT1, , or PTEN-altered tumours. The EORTC Quality of Life Questionnaire 30-item core module (QLQ-C30) and breast module (QLQ-BR23), Patient- Reported Outcomes version of the Common Terminology Criteria for Adverse Events (PRO-CTCAE), and Patient Global Impression of Treatment Tolerability (PGI-TT) questionnaires were used to assess patient-reported outcomes. Evaluation of EORTC QLQ-C30 and EORTC QLQ-BR23 were secondary endpoints and evaluation of PRO-CTCAE and PGI-TT were pre-defined exploratory endpoints, and these endpoints are the subject of analysis in this Article. Data were collected at baseline and prespecified timepoints. Patient-reported outcomes were analysed in all randomly assigned patients with an evaluable baseline assessment and at least one evaluable post-baseline assessment. Change from baseline was assessed using mixed model with repeated measures for EORTC QLQ-C30 and summarised for QLQ-BR23. Time to deterioration was described using the Kaplan-Meier method. PGI-TT and PRO-CTCAE responses were summarised at each treatment cycle. Patient-reported outcomes were not prospectively powered for statistical comparison. The trial is registered with ClinicalTrials.gov, NCT04305496. Findings Between June 2, 2020, and Oct 13, 2021, 901 patients were enrolled, of whom 708 patients were randomly assigned to receive capivasertib-fulvestrant (n=355) or placebo-fulvestrant (n=353). The median age of the patients was 59 years (IQR 51-67) in the capivasertib-fulvestrant group and 58 years (IQR 49-66) in the placebo-fulvestrant group. At data cutoff (Aug 15, 2022), the median duration of follow-up for progression-free survival in censored patients was 130 months (IQR 91-167) for capivasertib-fulvestrant and 127 months (IQR 20-164) for placebo- fulvestrant in the overall population. EORTC QLQ-C30 global health status/quality of life (GHS/QOL) scores were maintained from baseline and were similar between treatment groups throughout the study period (difference in mean change from baseline of -25 [95% CI -45 to -06] with capivasertib-fulvestrant vs -56 [-79 to -34] with placebo-fulvestrant; treatment difference 31 [95% CI 02 to 60]). Median time to deterioration in EORTC QLQ-C30 GHS/QOL was 249 months (95% CI 138 to not reached) in the capivasertib-fulvestrant group and 120 months (102 to 157) in the placebo-fulvestrant group (hazard ratio [HR] 070, 95% CI 053 to 092). Time to deterioration HRs for all EORTC QLQ-C30 and QLQ-BR23 subscale scores showed little difference between the treatment groups, except for diarrhoea, which was worse in the capivasertib-fulvestrant group than in the placebo-fulvestrant group (HR 275, 95% CI 201-381). In PRO-CTCAE symptom assessment, the proportion of patients reporting loose and watery stools frequently or almost constantly was 29% higher at cycle 1, day 15 in the capivasertib-fulvestrant group than in the placebo-fulvestrant group, decreasing at subsequent cycles. Other PRO-CTCAE-reported symptoms (rash, mouth or throat sores, itchy skin, and numbness or tingling in hands or feet) were absent or mild in most patients in both groups throughout treatment. According to the PGI-TT, most patients in both groups reported not at all or a little bit of bother from treatment side-effects. Interpretation Patient-reported outcomes from CAPItello-291 demonstrated that capivasertib-fulvestrant delayed time to deterioration of GHS/QOL and maintained other dimensions of HRQOL (except symptoms of diarrhoea) similarly to fulvestrant. With the clinical efficacy and manageable safety profile, these exploratory results further support the positive benefit-risk profile of capivasertib-fulvestrant in this population. Copyright (c) 2024 Elsevier Ltd. All rights reserved, including those for text and data mining, AI training, and similar technologies. | In the CAPItello-291 trial, adding capivasertib to fulvestrant for patients with advanced hormone receptor-positive, HER2-negative breast cancer helped delay disease progression and generally maintained patients’ quality of life compared with fulvestrant alone. While most side effects were mild and well-tolerated, diarrhea was more common with capivasertib but tended to improve over time. Overall, patients reported that treatment side effects caused little bother, supporting capivasertib-fulvestrant as an effective and manageable option for this type of breast cancer. | The CAPItello-291 trial tested capivasertib plus fulvestrant in patients with advanced hormone receptor-positive, HER2-negative breast cancer who had relapsed after prior treatment. The study found that this combination improved the time patients lived without their cancer getting worse and maintained overall quality of life compared with placebo plus fulvestrant. Most side effects were mild, though diarrhea was more common with capivasertib. Overall, patients tolerated the treatment well, supporting its positive benefit-risk profile. | The CAPItello-291 trial tested capivasertib plus fulvestrant in patients with advanced hormone receptor-positive, HER2-negative breast cancer and found it improved time without disease progression compared to fulvestrant alone. The study also looked at patients’ quality of life, symptoms, and how tolerable the treatment was. Results showed that overall health and quality of life were maintained with capivasertib-fulvestrant, except for more diarrhea early in treatment, which mostly improved over time. Most patients reported only mild side effects, supporting that capivasertib-fulvestrant is effective and generally well tolerated. |
| CancerRes | 3 | 10.1016/S1470-2045(24)00373-7 | Claude | Background CAPItello-291 is an ongoing phase 3 trial in which capivasertib-fulvestrant significantly improved progression-free survival versus placebo-fulvestrant in patients with hormone receptor-positive, HER2-negative advanced breast cancer who had relapse or disease progression during or after aromatase inhibitor treatment, in both the overall population and in patients with PIK3CA, , AKT1, , or PTEN- altered tumours. This study further explored patient-reported health-related quality of life (HRQOL), functioning, symptoms, and symptom tolerability in CAPItello-291. Methods This phase 3, randomised, double-blind, placebo-controlled trial, which was conducted across 193 hospitals and cancer centres in 19 countries, enrolled women with any menopausal status or men, aged >= 18 years (>= 20 years in Japan), with hormone receptor-positive, HER2-negative locally advanced or metastatic breast cancer who had relapse or disease progression during or after treatment with an aromatase inhibitor, with or without previous cyclindependent kinase (CDK) 4 or 6 inhibitor therapy. Patients had an Eastern Cooperative Oncology Group/WHO performance score of 0 or 1 and could have received up to two previous lines of endocrine therapy and up to one previous line of chemotherapy for advanced disease. Patients were randomly assigned (1:1) using block randomisation (stratified according to the presence or absence of liver metastases, previous use of a CDK4/6 inhibitor [yes vs no], and geographical region) to receive oral capivasertib 400 mg (twice daily for 4 days, followed by 3 days off) plus intramuscular fulvestrant 500 mg (every 14 days for the first three injections, then every 28 days) or placebo with matching fulvestrant dosing. The dual primary endpoint of the trial was investigator-assessed progression-free survival assessed both in the overall population and among patients with PIK3CA, , AKT1, , or PTEN-altered tumours. The EORTC Quality of Life Questionnaire 30-item core module (QLQ-C30) and breast module (QLQ-BR23), Patient- Reported Outcomes version of the Common Terminology Criteria for Adverse Events (PRO-CTCAE), and Patient Global Impression of Treatment Tolerability (PGI-TT) questionnaires were used to assess patient-reported outcomes. Evaluation of EORTC QLQ-C30 and EORTC QLQ-BR23 were secondary endpoints and evaluation of PRO-CTCAE and PGI-TT were pre-defined exploratory endpoints, and these endpoints are the subject of analysis in this Article. Data were collected at baseline and prespecified timepoints. Patient-reported outcomes were analysed in all randomly assigned patients with an evaluable baseline assessment and at least one evaluable post-baseline assessment. Change from baseline was assessed using mixed model with repeated measures for EORTC QLQ-C30 and summarised for QLQ-BR23. Time to deterioration was described using the Kaplan-Meier method. PGI-TT and PRO-CTCAE responses were summarised at each treatment cycle. Patient-reported outcomes were not prospectively powered for statistical comparison. The trial is registered with ClinicalTrials.gov, NCT04305496. Findings Between June 2, 2020, and Oct 13, 2021, 901 patients were enrolled, of whom 708 patients were randomly assigned to receive capivasertib-fulvestrant (n=355) or placebo-fulvestrant (n=353). The median age of the patients was 59 years (IQR 51-67) in the capivasertib-fulvestrant group and 58 years (IQR 49-66) in the placebo-fulvestrant group. At data cutoff (Aug 15, 2022), the median duration of follow-up for progression-free survival in censored patients was 130 months (IQR 91-167) for capivasertib-fulvestrant and 127 months (IQR 20-164) for placebo- fulvestrant in the overall population. EORTC QLQ-C30 global health status/quality of life (GHS/QOL) scores were maintained from baseline and were similar between treatment groups throughout the study period (difference in mean change from baseline of -25 [95% CI -45 to -06] with capivasertib-fulvestrant vs -56 [-79 to -34] with placebo-fulvestrant; treatment difference 31 [95% CI 02 to 60]). Median time to deterioration in EORTC QLQ-C30 GHS/QOL was 249 months (95% CI 138 to not reached) in the capivasertib-fulvestrant group and 120 months (102 to 157) in the placebo-fulvestrant group (hazard ratio [HR] 070, 95% CI 053 to 092). Time to deterioration HRs for all EORTC QLQ-C30 and QLQ-BR23 subscale scores showed little difference between the treatment groups, except for diarrhoea, which was worse in the capivasertib-fulvestrant group than in the placebo-fulvestrant group (HR 275, 95% CI 201-381). In PRO-CTCAE symptom assessment, the proportion of patients reporting loose and watery stools frequently or almost constantly was 29% higher at cycle 1, day 15 in the capivasertib-fulvestrant group than in the placebo-fulvestrant group, decreasing at subsequent cycles. Other PRO-CTCAE-reported symptoms (rash, mouth or throat sores, itchy skin, and numbness or tingling in hands or feet) were absent or mild in most patients in both groups throughout treatment. According to the PGI-TT, most patients in both groups reported not at all or a little bit of bother from treatment side-effects. Interpretation Patient-reported outcomes from CAPItello-291 demonstrated that capivasertib-fulvestrant delayed time to deterioration of GHS/QOL and maintained other dimensions of HRQOL (except symptoms of diarrhoea) similarly to fulvestrant. With the clinical efficacy and manageable safety profile, these exploratory results further support the positive benefit-risk profile of capivasertib-fulvestrant in this population. Copyright (c) 2024 Elsevier Ltd. All rights reserved, including those for text and data mining, AI training, and similar technologies. | This study looked at the quality of life for breast cancer patients taking part in a clinical trial called CAPItello-291. The trial tested whether adding a new drug called capivasertib to the standard treatment (fulvestrant) would help women with hormone-positive, HER2-negative advanced breast cancer that had gotten worse despite previous treatment. Researchers asked 708 patients to fill out questionnaires about their symptoms, daily functioning, and overall well-being throughout treatment. They found that patients receiving the combination treatment maintained their quality of life better and took longer to experience a decline in their overall health status compared to those getting the standard treatment alone. The main side effect was diarrhea, which was more common with the combination treatment but was generally manageable. Overall, the results suggest that adding capivasertib to standard treatment not only helps control the cancer better but also helps patients maintain their quality of life. | This study looked at how patients felt while taking a new breast cancer treatment called capivasertib-fulvestrant compared to a standard treatment. The researchers surveyed 708 women with hormone receptor-positive, HER2-negative advanced breast cancer to understand their quality of life, symptoms, and how well they tolerated the treatments. They found that patients taking the new combination treatment maintained their quality of life longer than those on standard treatment - it took about 25 months for their quality of life to worsen compared to 12 months with standard treatment. The main side effect was diarrhea, which was more common with the new treatment but manageable, and most patients in both groups said the side effects didn't bother them much. Overall, the results suggest the new treatment helps patients live better for longer while effectively treating their cancer. | This study looked at how patients with advanced breast cancer felt while receiving a new combination treatment (capivasertib plus fulvestrant) compared to the standard treatment (fulvestrant alone). The researchers used questionnaires to measure patients' quality of life, symptoms, and how bothersome the treatment side effects were. They found that patients receiving the combination treatment maintained their quality of life just as well as those on standard treatment, and actually took longer before their quality of life got worse. The main difference was that patients on the combination treatment experienced more diarrhea, but most patients in both groups reported that treatment side effects were only mildly bothersome. Overall, the study showed that while the combination treatment was more effective at controlling cancer, it didn't significantly worsen patients' day-to-day wellbeing compared to standard treatment. |
| CancerRes | 3 | 10.1016/S1470-2045(24)00373-8 | Gemini | Background CAPItello-291 is an ongoing phase 3 trial in which capivasertib-fulvestrant significantly improved progression-free survival versus placebo-fulvestrant in patients with hormone receptor-positive, HER2-negative advanced breast cancer who had relapse or disease progression during or after aromatase inhibitor treatment, in both the overall population and in patients with PIK3CA, , AKT1, , or PTEN- altered tumours. This study further explored patient-reported health-related quality of life (HRQOL), functioning, symptoms, and symptom tolerability in CAPItello-291. Methods This phase 3, randomised, double-blind, placebo-controlled trial, which was conducted across 193 hospitals and cancer centres in 19 countries, enrolled women with any menopausal status or men, aged >= 18 years (>= 20 years in Japan), with hormone receptor-positive, HER2-negative locally advanced or metastatic breast cancer who had relapse or disease progression during or after treatment with an aromatase inhibitor, with or without previous cyclindependent kinase (CDK) 4 or 6 inhibitor therapy. Patients had an Eastern Cooperative Oncology Group/WHO performance score of 0 or 1 and could have received up to two previous lines of endocrine therapy and up to one previous line of chemotherapy for advanced disease. Patients were randomly assigned (1:1) using block randomisation (stratified according to the presence or absence of liver metastases, previous use of a CDK4/6 inhibitor [yes vs no], and geographical region) to receive oral capivasertib 400 mg (twice daily for 4 days, followed by 3 days off) plus intramuscular fulvestrant 500 mg (every 14 days for the first three injections, then every 28 days) or placebo with matching fulvestrant dosing. The dual primary endpoint of the trial was investigator-assessed progression-free survival assessed both in the overall population and among patients with PIK3CA, , AKT1, , or PTEN-altered tumours. The EORTC Quality of Life Questionnaire 30-item core module (QLQ-C30) and breast module (QLQ-BR23), Patient- Reported Outcomes version of the Common Terminology Criteria for Adverse Events (PRO-CTCAE), and Patient Global Impression of Treatment Tolerability (PGI-TT) questionnaires were used to assess patient-reported outcomes. Evaluation of EORTC QLQ-C30 and EORTC QLQ-BR23 were secondary endpoints and evaluation of PRO-CTCAE and PGI-TT were pre-defined exploratory endpoints, and these endpoints are the subject of analysis in this Article. Data were collected at baseline and prespecified timepoints. Patient-reported outcomes were analysed in all randomly assigned patients with an evaluable baseline assessment and at least one evaluable post-baseline assessment. Change from baseline was assessed using mixed model with repeated measures for EORTC QLQ-C30 and summarised for QLQ-BR23. Time to deterioration was described using the Kaplan-Meier method. PGI-TT and PRO-CTCAE responses were summarised at each treatment cycle. Patient-reported outcomes were not prospectively powered for statistical comparison. The trial is registered with ClinicalTrials.gov, NCT04305496. Findings Between June 2, 2020, and Oct 13, 2021, 901 patients were enrolled, of whom 708 patients were randomly assigned to receive capivasertib-fulvestrant (n=355) or placebo-fulvestrant (n=353). The median age of the patients was 59 years (IQR 51-67) in the capivasertib-fulvestrant group and 58 years (IQR 49-66) in the placebo-fulvestrant group. At data cutoff (Aug 15, 2022), the median duration of follow-up for progression-free survival in censored patients was 130 months (IQR 91-167) for capivasertib-fulvestrant and 127 months (IQR 20-164) for placebo- fulvestrant in the overall population. EORTC QLQ-C30 global health status/quality of life (GHS/QOL) scores were maintained from baseline and were similar between treatment groups throughout the study period (difference in mean change from baseline of -25 [95% CI -45 to -06] with capivasertib-fulvestrant vs -56 [-79 to -34] with placebo-fulvestrant; treatment difference 31 [95% CI 02 to 60]). Median time to deterioration in EORTC QLQ-C30 GHS/QOL was 249 months (95% CI 138 to not reached) in the capivasertib-fulvestrant group and 120 months (102 to 157) in the placebo-fulvestrant group (hazard ratio [HR] 070, 95% CI 053 to 092). Time to deterioration HRs for all EORTC QLQ-C30 and QLQ-BR23 subscale scores showed little difference between the treatment groups, except for diarrhoea, which was worse in the capivasertib-fulvestrant group than in the placebo-fulvestrant group (HR 275, 95% CI 201-381). In PRO-CTCAE symptom assessment, the proportion of patients reporting loose and watery stools frequently or almost constantly was 29% higher at cycle 1, day 15 in the capivasertib-fulvestrant group than in the placebo-fulvestrant group, decreasing at subsequent cycles. Other PRO-CTCAE-reported symptoms (rash, mouth or throat sores, itchy skin, and numbness or tingling in hands or feet) were absent or mild in most patients in both groups throughout treatment. According to the PGI-TT, most patients in both groups reported not at all or a little bit of bother from treatment side-effects. Interpretation Patient-reported outcomes from CAPItello-291 demonstrated that capivasertib-fulvestrant delayed time to deterioration of GHS/QOL and maintained other dimensions of HRQOL (except symptoms of diarrhoea) similarly to fulvestrant. With the clinical efficacy and manageable safety profile, these exploratory results further support the positive benefit-risk profile of capivasertib-fulvestrant in this population. Copyright (c) 2024 Elsevier Ltd. All rights reserved, including those for text and data mining, AI training, and similar technologies. | In a study called CAPItello-291, researchers found that adding the drug capivasertib to the standard treatment fulvestrant helped people with a specific type of advanced breast cancer live longer without their cancer getting worse. The study also looked at how the treatment affected patients' daily lives. They discovered that while the combination of capivasertib and fulvestrant caused more diarrhea, it didn't negatively impact their overall quality of life. In fact, patients on the new combination treatment experienced a longer delay before their general health and quality of life started to decline compared to those who only received fulvestrant. These findings suggest that the benefits of the new treatment outweigh the side effects, making it a good option for these patients. | In a study called CAPItello-291, researchers found that the combination of capivasertib and fulvestrant improved progression-free survival for patients with a specific type of advanced breast cancer. The study also looked at how the treatment affected patients' quality of life. The results showed that while the capivasertib-fulvestrant combination caused more frequent diarrhea, it didn't negatively impact other aspects of a patient's overall quality of life or their ability to handle the side effects of the treatment. In fact, patients on the new drug combination had a longer time before their overall quality of life started to get worse compared to those on the placebo. These findings suggest the new treatment has a positive balance of benefits and risks for patients. | Based on the provided abstract from the CAPItello-291 trial, adding capivasertib to fulvestrant treatment for advanced breast cancer patients delayed the time it took for their overall quality of life to worsen, without negatively affecting most other aspects of their health-related quality of life. The only significant side effect was an increase in diarrhea. The study concludes that the combination of capivasertib and fulvestrant has a positive balance of benefits and risks for this group of patients. |
| CancerRes | 3 | 10.1016/S1470-2045(24)00373-9 | DeepSeek | Background CAPItello-291 is an ongoing phase 3 trial in which capivasertib-fulvestrant significantly improved progression-free survival versus placebo-fulvestrant in patients with hormone receptor-positive, HER2-negative advanced breast cancer who had relapse or disease progression during or after aromatase inhibitor treatment, in both the overall population and in patients with PIK3CA, , AKT1, , or PTEN- altered tumours. This study further explored patient-reported health-related quality of life (HRQOL), functioning, symptoms, and symptom tolerability in CAPItello-291. Methods This phase 3, randomised, double-blind, placebo-controlled trial, which was conducted across 193 hospitals and cancer centres in 19 countries, enrolled women with any menopausal status or men, aged >= 18 years (>= 20 years in Japan), with hormone receptor-positive, HER2-negative locally advanced or metastatic breast cancer who had relapse or disease progression during or after treatment with an aromatase inhibitor, with or without previous cyclindependent kinase (CDK) 4 or 6 inhibitor therapy. Patients had an Eastern Cooperative Oncology Group/WHO performance score of 0 or 1 and could have received up to two previous lines of endocrine therapy and up to one previous line of chemotherapy for advanced disease. Patients were randomly assigned (1:1) using block randomisation (stratified according to the presence or absence of liver metastases, previous use of a CDK4/6 inhibitor [yes vs no], and geographical region) to receive oral capivasertib 400 mg (twice daily for 4 days, followed by 3 days off) plus intramuscular fulvestrant 500 mg (every 14 days for the first three injections, then every 28 days) or placebo with matching fulvestrant dosing. The dual primary endpoint of the trial was investigator-assessed progression-free survival assessed both in the overall population and among patients with PIK3CA, , AKT1, , or PTEN-altered tumours. The EORTC Quality of Life Questionnaire 30-item core module (QLQ-C30) and breast module (QLQ-BR23), Patient- Reported Outcomes version of the Common Terminology Criteria for Adverse Events (PRO-CTCAE), and Patient Global Impression of Treatment Tolerability (PGI-TT) questionnaires were used to assess patient-reported outcomes. Evaluation of EORTC QLQ-C30 and EORTC QLQ-BR23 were secondary endpoints and evaluation of PRO-CTCAE and PGI-TT were pre-defined exploratory endpoints, and these endpoints are the subject of analysis in this Article. Data were collected at baseline and prespecified timepoints. Patient-reported outcomes were analysed in all randomly assigned patients with an evaluable baseline assessment and at least one evaluable post-baseline assessment. Change from baseline was assessed using mixed model with repeated measures for EORTC QLQ-C30 and summarised for QLQ-BR23. Time to deterioration was described using the Kaplan-Meier method. PGI-TT and PRO-CTCAE responses were summarised at each treatment cycle. Patient-reported outcomes were not prospectively powered for statistical comparison. The trial is registered with ClinicalTrials.gov, NCT04305496. Findings Between June 2, 2020, and Oct 13, 2021, 901 patients were enrolled, of whom 708 patients were randomly assigned to receive capivasertib-fulvestrant (n=355) or placebo-fulvestrant (n=353). The median age of the patients was 59 years (IQR 51-67) in the capivasertib-fulvestrant group and 58 years (IQR 49-66) in the placebo-fulvestrant group. At data cutoff (Aug 15, 2022), the median duration of follow-up for progression-free survival in censored patients was 130 months (IQR 91-167) for capivasertib-fulvestrant and 127 months (IQR 20-164) for placebo- fulvestrant in the overall population. EORTC QLQ-C30 global health status/quality of life (GHS/QOL) scores were maintained from baseline and were similar between treatment groups throughout the study period (difference in mean change from baseline of -25 [95% CI -45 to -06] with capivasertib-fulvestrant vs -56 [-79 to -34] with placebo-fulvestrant; treatment difference 31 [95% CI 02 to 60]). Median time to deterioration in EORTC QLQ-C30 GHS/QOL was 249 months (95% CI 138 to not reached) in the capivasertib-fulvestrant group and 120 months (102 to 157) in the placebo-fulvestrant group (hazard ratio [HR] 070, 95% CI 053 to 092). Time to deterioration HRs for all EORTC QLQ-C30 and QLQ-BR23 subscale scores showed little difference between the treatment groups, except for diarrhoea, which was worse in the capivasertib-fulvestrant group than in the placebo-fulvestrant group (HR 275, 95% CI 201-381). In PRO-CTCAE symptom assessment, the proportion of patients reporting loose and watery stools frequently or almost constantly was 29% higher at cycle 1, day 15 in the capivasertib-fulvestrant group than in the placebo-fulvestrant group, decreasing at subsequent cycles. Other PRO-CTCAE-reported symptoms (rash, mouth or throat sores, itchy skin, and numbness or tingling in hands or feet) were absent or mild in most patients in both groups throughout treatment. According to the PGI-TT, most patients in both groups reported not at all or a little bit of bother from treatment side-effects. Interpretation Patient-reported outcomes from CAPItello-291 demonstrated that capivasertib-fulvestrant delayed time to deterioration of GHS/QOL and maintained other dimensions of HRQOL (except symptoms of diarrhoea) similarly to fulvestrant. With the clinical efficacy and manageable safety profile, these exploratory results further support the positive benefit-risk profile of capivasertib-fulvestrant in this population. Copyright (c) 2024 Elsevier Ltd. All rights reserved, including those for text and data mining, AI training, and similar technologies. | The CAPItello-291 trial tested capivasertib combined with fulvestrant in patients with advanced hormone receptor-positive, HER2-negative breast cancer. The study found that while the combination improved progression-free survival, it also maintained patients' overall quality of life compared to fulvestrant alone, except for increased diarrhea. Most side effects were mild, and patients generally tolerated the treatment well. These results support capivasertib-fulvestrant as a beneficial option for this group. | The CAPItello-291 trial tested capivasertib plus fulvestrant versus placebo plus fulvestrant in patients with advanced hormone receptor-positive, HER2-negative breast cancer. The study found that while the combination improved survival, it also maintained patients' overall quality of life, except for increased diarrhea. Most side effects were mild, and patients generally reported minimal bother from treatment. These results support capivasertib-fulvestrant as a beneficial option with manageable side effects for this group. | The CAPItello-291 trial tested capivasertib combined with fulvestrant in patients with advanced hormone receptor-positive, HER2-negative breast cancer that worsened after prior treatment. The study found that while the combination improved progression-free survival, it also maintained patients' overall quality of life compared to fulvestrant alone, except for increased diarrhea. Most side effects were mild, and patients reported minimal bother from treatment. These results support capivasertib-fulvestrant as a beneficial option with a manageable safety profile for this group. |
| CancerRes | 3 | 10.1016/S1470-2045(24)00373-10 | Grok | Background CAPItello-291 is an ongoing phase 3 trial in which capivasertib-fulvestrant significantly improved progression-free survival versus placebo-fulvestrant in patients with hormone receptor-positive, HER2-negative advanced breast cancer who had relapse or disease progression during or after aromatase inhibitor treatment, in both the overall population and in patients with PIK3CA, , AKT1, , or PTEN- altered tumours. This study further explored patient-reported health-related quality of life (HRQOL), functioning, symptoms, and symptom tolerability in CAPItello-291. Methods This phase 3, randomised, double-blind, placebo-controlled trial, which was conducted across 193 hospitals and cancer centres in 19 countries, enrolled women with any menopausal status or men, aged >= 18 years (>= 20 years in Japan), with hormone receptor-positive, HER2-negative locally advanced or metastatic breast cancer who had relapse or disease progression during or after treatment with an aromatase inhibitor, with or without previous cyclindependent kinase (CDK) 4 or 6 inhibitor therapy. Patients had an Eastern Cooperative Oncology Group/WHO performance score of 0 or 1 and could have received up to two previous lines of endocrine therapy and up to one previous line of chemotherapy for advanced disease. Patients were randomly assigned (1:1) using block randomisation (stratified according to the presence or absence of liver metastases, previous use of a CDK4/6 inhibitor [yes vs no], and geographical region) to receive oral capivasertib 400 mg (twice daily for 4 days, followed by 3 days off) plus intramuscular fulvestrant 500 mg (every 14 days for the first three injections, then every 28 days) or placebo with matching fulvestrant dosing. The dual primary endpoint of the trial was investigator-assessed progression-free survival assessed both in the overall population and among patients with PIK3CA, , AKT1, , or PTEN-altered tumours. The EORTC Quality of Life Questionnaire 30-item core module (QLQ-C30) and breast module (QLQ-BR23), Patient- Reported Outcomes version of the Common Terminology Criteria for Adverse Events (PRO-CTCAE), and Patient Global Impression of Treatment Tolerability (PGI-TT) questionnaires were used to assess patient-reported outcomes. Evaluation of EORTC QLQ-C30 and EORTC QLQ-BR23 were secondary endpoints and evaluation of PRO-CTCAE and PGI-TT were pre-defined exploratory endpoints, and these endpoints are the subject of analysis in this Article. Data were collected at baseline and prespecified timepoints. Patient-reported outcomes were analysed in all randomly assigned patients with an evaluable baseline assessment and at least one evaluable post-baseline assessment. Change from baseline was assessed using mixed model with repeated measures for EORTC QLQ-C30 and summarised for QLQ-BR23. Time to deterioration was described using the Kaplan-Meier method. PGI-TT and PRO-CTCAE responses were summarised at each treatment cycle. Patient-reported outcomes were not prospectively powered for statistical comparison. The trial is registered with ClinicalTrials.gov, NCT04305496. Findings Between June 2, 2020, and Oct 13, 2021, 901 patients were enrolled, of whom 708 patients were randomly assigned to receive capivasertib-fulvestrant (n=355) or placebo-fulvestrant (n=353). The median age of the patients was 59 years (IQR 51-67) in the capivasertib-fulvestrant group and 58 years (IQR 49-66) in the placebo-fulvestrant group. At data cutoff (Aug 15, 2022), the median duration of follow-up for progression-free survival in censored patients was 130 months (IQR 91-167) for capivasertib-fulvestrant and 127 months (IQR 20-164) for placebo- fulvestrant in the overall population. EORTC QLQ-C30 global health status/quality of life (GHS/QOL) scores were maintained from baseline and were similar between treatment groups throughout the study period (difference in mean change from baseline of -25 [95% CI -45 to -06] with capivasertib-fulvestrant vs -56 [-79 to -34] with placebo-fulvestrant; treatment difference 31 [95% CI 02 to 60]). Median time to deterioration in EORTC QLQ-C30 GHS/QOL was 249 months (95% CI 138 to not reached) in the capivasertib-fulvestrant group and 120 months (102 to 157) in the placebo-fulvestrant group (hazard ratio [HR] 070, 95% CI 053 to 092). Time to deterioration HRs for all EORTC QLQ-C30 and QLQ-BR23 subscale scores showed little difference between the treatment groups, except for diarrhoea, which was worse in the capivasertib-fulvestrant group than in the placebo-fulvestrant group (HR 275, 95% CI 201-381). In PRO-CTCAE symptom assessment, the proportion of patients reporting loose and watery stools frequently or almost constantly was 29% higher at cycle 1, day 15 in the capivasertib-fulvestrant group than in the placebo-fulvestrant group, decreasing at subsequent cycles. Other PRO-CTCAE-reported symptoms (rash, mouth or throat sores, itchy skin, and numbness or tingling in hands or feet) were absent or mild in most patients in both groups throughout treatment. According to the PGI-TT, most patients in both groups reported not at all or a little bit of bother from treatment side-effects. Interpretation Patient-reported outcomes from CAPItello-291 demonstrated that capivasertib-fulvestrant delayed time to deterioration of GHS/QOL and maintained other dimensions of HRQOL (except symptoms of diarrhoea) similarly to fulvestrant. With the clinical efficacy and manageable safety profile, these exploratory results further support the positive benefit-risk profile of capivasertib-fulvestrant in this population. Copyright (c) 2024 Elsevier Ltd. All rights reserved, including those for text and data mining, AI training, and similar technologies. | The CAPItello-291 study tested a new treatment combining capivasertib and fulvestrant for patients with hormone receptor-positive, HER2-negative advanced breast cancer who had cancer progression after aromatase inhibitor treatment. This phase 3 trial, involving 708 patients, showed that capivasertib-fulvestrant extended progression-free survival compared to placebo-fulvestrant. Patient-reported outcomes, measured with questionnaires, indicated that quality of life was maintained similarly in both groups, though the capivasertib group experienced a longer delay before quality of life worsened (24.9 months vs. 12.0 months). Diarrhea was more common with capivasertib, especially early on, but other symptoms like rash or itching were generally mild. Overall, the treatment was tolerable, supporting its potential as an effective option with manageable side effects. | The CAPItello-291 phase 3 trial studied capivasertib combined with fulvestrant in patients with hormone receptor-positive, HER2-negative advanced breast cancer who had disease progression after aromatase inhibitor treatment. The trial, involving 708 patients across 19 countries, showed that capivasertib-fulvestrant extended progression-free survival compared to placebo-fulvestrant. Patient-reported outcomes, measured using questionnaires, indicated that quality of life was maintained similarly in both groups, though the capivasertib group experienced a longer time before quality of life worsened (24.9 vs. 12.0 months). Diarrhea was more common with capivasertib, but other symptoms like rash or itchy skin were generally mild, and most patients reported minimal bother from side effects, supporting the treatment’s favorable benefit-risk profile. | The CAPItello-291 phase 3 trial studied capivasertib combined with fulvestrant in patients with hormone receptor-positive, HER2-negative advanced breast cancer who had disease progression after aromatase inhibitor treatment. The trial, involving 708 patients across 19 countries, showed that capivasertib-fulvestrant extended progression-free survival compared to placebo-fulvestrant. Patient-reported outcomes, measured using questionnaires, indicated that quality of life was maintained similarly in both groups, though the capivasertib group experienced a longer delay before quality of life worsened (24.9 vs. 12.0 months). Diarrhea was more frequent with capivasertib, but other symptoms like rash or itchy skin were generally mild. Overall, the treatment was well-tolerated, supporting its positive benefit-risk profile. |
| CancerRes | 4 | 10.1016/S1470-2045(25)00128-8 | Open AI | Background Infections are the second leading cause of death in patients with cancer and are often caused by resistant bacteria. However, the frequency of antimicrobial resistance (AMR) in outpatients with cancer is not well understood. We aimed to compare the frequency of AMR bacterial pathogens in outpatients with and without cancer. Methods This retrospective cohort study evaluated antimicrobial susceptibility of bacteria isolated from adults (aged >= 18 years) with and without cancer seeking care in 198 outpatient health-care settings in the USA. Data were collected using the BD Insights Research Database. Patients who were not prescribed cancer medications or not admitted to an inpatient cancer unit in the predefined period were categorised as patients without cancer. Patients were included in the cancer cohort if they received medication solely or sometimes indicated for cancer. Data on gender and race or ethnicity were not collected. Non-duplicate and non-contaminant pathogens collected from various samples (ie, blood, intra-abdominal, respiratory, urine, skin or wound, and other) in outpatients were used to assess the coprimary outcomes: overall and source-specific proportions of non-susceptible pathogen isolates with corresponding AMR odds ratios (ORs); and rates of AMR pathogens per 1000 isolates with corresponding AMR incidence rate ratio (IRR) in patients with and without cancer. Findings Data were collected between April 1, 2018, and Dec 31, 2022. 53 006 (3 center dot 2%) of 1 655 594 pathogens identified were from 27 421 patients with cancer and 1 602 588 (96 center dot 8%) were from 928 128 patients without cancer. For Pseudomonas aeruginosa, carbapenem non-susceptibility was higher in pathogen isolates from patients with cancer (816 [14 center dot 4%] of 5683) than patients without cancer (10 709 [11 center dot 3%] 94 419; OR 1 center dot 22 [95% CI 1 center dot 13-1 center dot 32]). For Enterobacterales, fluoroquinolone non-susceptibility was higher in pathogen isolates from patients with cancer (8662 [28 center dot 0%] of 30 867) than patients without cancer (238 479 [21 center dot 8%] of 1 095 996; OR 1 center dot 44 [1 center dot 40-1 center dot 47]), as was carbapenem non-susceptibility (472 [1 center dot 5%] of 30 867 vs 9165 [0 center dot 8%] of 1 095 996; OR 1 center dot 89 [1 center dot 72-2 center dot 07]), multidrug-resistant pathogens (2672 [8 center dot 7%] of 30 867 vs 48 962 [4 center dot 5%] of 1 095 996; OR 2 center dot 03 [1 center dot 95-2 center dot 11]), and extended-spectrum beta-lactamase producers (4343 [16 center dot 5%] of 26 327 vs 93 977 [9 center dot 4%] of 996 853; OR 1 center dot 96 [1 center dot 90-2 center dot 03]). For Staphylococcus aureus, meticillin resistance was higher in pathogen isolates from patients with cancer (4747 [53 center dot 0%] of 8959) than patients without cancer (129 291 [48 center dot 3%] of 267 520; OR 1 center dot 20 [1 center dot 15-1 center dot 25]). For Enterococcus spp, vancomycin resistance was higher in pathogen isolates from patients with cancer (1329 [18 center dot 6%] of 7145) than patients without cancer (12 333 [9 center dot 1%] of 135 772]; ORR 2 center dot 20 [2 center dot 06-2 center dot 34). The rates and corresponding IRRs of AMR pathogens per 1000 isolates was also higher in patients with cancer compared with patients without cancer, particularly for carbapenem non-susceptible P aeruginosa (IRR 2 center dot 06 [1 center dot 91-2 center dot 21]) and vancomycin-resistant enterococci (IRR 3 center dot 06 [2 center dot 89-3 center dot 24]). For all comparisons, p<0 center dot 0001. Interpretation AMR proportions and IRRs for most key pathogens were up to three-times higher in isolates from outpatients with cancer than those without cancer, highlighting the need for enhanced surveillance, infection prevention, and timely diagnostic stewardship to improve antibiotic prescribing in this population. Copyright (c) 2025 Elsevier Ltd. All rights reserved, including those for text and data mining, AI training, and similar technologies. | A study of over 1.6 million bacterial samples from U.S. outpatients found that people with cancer are more likely to have infections caused by antibiotic-resistant bacteria than those without cancer. Resistance was higher across several key pathogens, including Pseudomonas, Enterobacterales, Staphylococcus aureus, and Enterococcus, with rates up to three times greater in cancer patients. This shows the need for better monitoring, infection prevention, and careful use of antibiotics in outpatient cancer care. | A study of over 1.6 million bacterial samples from US outpatients found that people with cancer had much higher rates of antibiotic-resistant infections than those without cancer. Key bacteria like Pseudomonas, Enterobacterales, Staphylococcus aureus, and Enterococcus showed significantly more resistance to common antibiotics in cancer patients. These findings suggest that outpatients with cancer are at greater risk of hard-to-treat infections, emphasizing the need for better infection monitoring, prevention, and careful antibiotic use in this group. | A study of over 1.6 million bacterial infections in U.S. outpatients found that people with cancer had much higher rates of antibiotic-resistant bacteria than those without cancer. Resistant strains of Pseudomonas, Enterobacterales, Staphylococcus aureus, and Enterococcus were all more common in cancer patients, sometimes two to three times higher. This shows that cancer patients are at greater risk for hard-to-treat infections, emphasizing the need for better monitoring, infection prevention, and careful use of antibiotics in this group. |
| CancerRes | 4 | 10.1016/S1470-2045(25)00128-9 | Claude | Background Infections are the second leading cause of death in patients with cancer and are often caused by resistant bacteria. However, the frequency of antimicrobial resistance (AMR) in outpatients with cancer is not well understood. We aimed to compare the frequency of AMR bacterial pathogens in outpatients with and without cancer. Methods This retrospective cohort study evaluated antimicrobial susceptibility of bacteria isolated from adults (aged >= 18 years) with and without cancer seeking care in 198 outpatient health-care settings in the USA. Data were collected using the BD Insights Research Database. Patients who were not prescribed cancer medications or not admitted to an inpatient cancer unit in the predefined period were categorised as patients without cancer. Patients were included in the cancer cohort if they received medication solely or sometimes indicated for cancer. Data on gender and race or ethnicity were not collected. Non-duplicate and non-contaminant pathogens collected from various samples (ie, blood, intra-abdominal, respiratory, urine, skin or wound, and other) in outpatients were used to assess the coprimary outcomes: overall and source-specific proportions of non-susceptible pathogen isolates with corresponding AMR odds ratios (ORs); and rates of AMR pathogens per 1000 isolates with corresponding AMR incidence rate ratio (IRR) in patients with and without cancer. Findings Data were collected between April 1, 2018, and Dec 31, 2022. 53 006 (3 center dot 2%) of 1 655 594 pathogens identified were from 27 421 patients with cancer and 1 602 588 (96 center dot 8%) were from 928 128 patients without cancer. For Pseudomonas aeruginosa, carbapenem non-susceptibility was higher in pathogen isolates from patients with cancer (816 [14 center dot 4%] of 5683) than patients without cancer (10 709 [11 center dot 3%] 94 419; OR 1 center dot 22 [95% CI 1 center dot 13-1 center dot 32]). For Enterobacterales, fluoroquinolone non-susceptibility was higher in pathogen isolates from patients with cancer (8662 [28 center dot 0%] of 30 867) than patients without cancer (238 479 [21 center dot 8%] of 1 095 996; OR 1 center dot 44 [1 center dot 40-1 center dot 47]), as was carbapenem non-susceptibility (472 [1 center dot 5%] of 30 867 vs 9165 [0 center dot 8%] of 1 095 996; OR 1 center dot 89 [1 center dot 72-2 center dot 07]), multidrug-resistant pathogens (2672 [8 center dot 7%] of 30 867 vs 48 962 [4 center dot 5%] of 1 095 996; OR 2 center dot 03 [1 center dot 95-2 center dot 11]), and extended-spectrum beta-lactamase producers (4343 [16 center dot 5%] of 26 327 vs 93 977 [9 center dot 4%] of 996 853; OR 1 center dot 96 [1 center dot 90-2 center dot 03]). For Staphylococcus aureus, meticillin resistance was higher in pathogen isolates from patients with cancer (4747 [53 center dot 0%] of 8959) than patients without cancer (129 291 [48 center dot 3%] of 267 520; OR 1 center dot 20 [1 center dot 15-1 center dot 25]). For Enterococcus spp, vancomycin resistance was higher in pathogen isolates from patients with cancer (1329 [18 center dot 6%] of 7145) than patients without cancer (12 333 [9 center dot 1%] of 135 772]; ORR 2 center dot 20 [2 center dot 06-2 center dot 34). The rates and corresponding IRRs of AMR pathogens per 1000 isolates was also higher in patients with cancer compared with patients without cancer, particularly for carbapenem non-susceptible P aeruginosa (IRR 2 center dot 06 [1 center dot 91-2 center dot 21]) and vancomycin-resistant enterococci (IRR 3 center dot 06 [2 center dot 89-3 center dot 24]). For all comparisons, p<0 center dot 0001. Interpretation AMR proportions and IRRs for most key pathogens were up to three-times higher in isolates from outpatients with cancer than those without cancer, highlighting the need for enhanced surveillance, infection prevention, and timely diagnostic stewardship to improve antibiotic prescribing in this population. Copyright (c) 2025 Elsevier Ltd. All rights reserved, including those for text and data mining, AI training, and similar technologies. | This study looked at antibiotic-resistant bacteria in cancer patients compared to people without cancer who were receiving outpatient care (not hospitalized). Researchers analyzed over 1.6 million bacterial samples from nearly a million patients across 198 U.S. healthcare facilities between 2018 and 2022. They found that cancer patients had significantly higher rates of antibiotic-resistant infections - sometimes up to three times higher - including dangerous bacteria like MRSA and drug-resistant E. coli. Since infections are already the second leading cause of death in cancer patients, these higher resistance rates are concerning and suggest that cancer patients need better infection monitoring, prevention strategies, and more careful antibiotic prescribing to protect this vulnerable population. | This study looked at antibiotic-resistant bacteria in cancer patients who receive outpatient care (not hospitalized). Researchers compared bacteria samples from over 27,000 cancer patients to samples from more than 900,000 patients without cancer at 198 U.S. healthcare facilities between 2018-2022. They found that cancer patients had significantly higher rates of antibiotic-resistant infections - up to three times higher for some dangerous bacteria like vancomycin-resistant enterococci and carbapenem-resistant Pseudomonas. This means common antibiotics don't work as well against infections in cancer patients, making these infections harder to treat. The researchers concluded that cancer patients need better infection monitoring, prevention strategies, and more careful antibiotic prescribing to address this increased risk of drug-resistant infections. | This study looked at antibiotic-resistant bacteria in cancer patients versus non-cancer patients who were treated as outpatients (not hospitalized). Researchers analyzed over 1.6 million bacterial samples from nearly 1 million patients across 198 U.S. healthcare facilities between 2018 and 2022. They found that cancer patients had significantly higher rates of antibiotic-resistant infections - up to three times higher for some dangerous bacteria like vancomycin-resistant enterococci and carbapenem-resistant Pseudomonas. The results show that cancer patients face a much greater threat from hard-to-treat bacterial infections, which means doctors need better infection prevention strategies and more careful antibiotic prescribing for this vulnerable population. |
| CancerRes | 4 | 10.1016/S1470-2045(25)00128-10 | Gemini | Background Infections are the second leading cause of death in patients with cancer and are often caused by resistant bacteria. However, the frequency of antimicrobial resistance (AMR) in outpatients with cancer is not well understood. We aimed to compare the frequency of AMR bacterial pathogens in outpatients with and without cancer. Methods This retrospective cohort study evaluated antimicrobial susceptibility of bacteria isolated from adults (aged >= 18 years) with and without cancer seeking care in 198 outpatient health-care settings in the USA. Data were collected using the BD Insights Research Database. Patients who were not prescribed cancer medications or not admitted to an inpatient cancer unit in the predefined period were categorised as patients without cancer. Patients were included in the cancer cohort if they received medication solely or sometimes indicated for cancer. Data on gender and race or ethnicity were not collected. Non-duplicate and non-contaminant pathogens collected from various samples (ie, blood, intra-abdominal, respiratory, urine, skin or wound, and other) in outpatients were used to assess the coprimary outcomes: overall and source-specific proportions of non-susceptible pathogen isolates with corresponding AMR odds ratios (ORs); and rates of AMR pathogens per 1000 isolates with corresponding AMR incidence rate ratio (IRR) in patients with and without cancer. Findings Data were collected between April 1, 2018, and Dec 31, 2022. 53 006 (3 center dot 2%) of 1 655 594 pathogens identified were from 27 421 patients with cancer and 1 602 588 (96 center dot 8%) were from 928 128 patients without cancer. For Pseudomonas aeruginosa, carbapenem non-susceptibility was higher in pathogen isolates from patients with cancer (816 [14 center dot 4%] of 5683) than patients without cancer (10 709 [11 center dot 3%] 94 419; OR 1 center dot 22 [95% CI 1 center dot 13-1 center dot 32]). For Enterobacterales, fluoroquinolone non-susceptibility was higher in pathogen isolates from patients with cancer (8662 [28 center dot 0%] of 30 867) than patients without cancer (238 479 [21 center dot 8%] of 1 095 996; OR 1 center dot 44 [1 center dot 40-1 center dot 47]), as was carbapenem non-susceptibility (472 [1 center dot 5%] of 30 867 vs 9165 [0 center dot 8%] of 1 095 996; OR 1 center dot 89 [1 center dot 72-2 center dot 07]), multidrug-resistant pathogens (2672 [8 center dot 7%] of 30 867 vs 48 962 [4 center dot 5%] of 1 095 996; OR 2 center dot 03 [1 center dot 95-2 center dot 11]), and extended-spectrum beta-lactamase producers (4343 [16 center dot 5%] of 26 327 vs 93 977 [9 center dot 4%] of 996 853; OR 1 center dot 96 [1 center dot 90-2 center dot 03]). For Staphylococcus aureus, meticillin resistance was higher in pathogen isolates from patients with cancer (4747 [53 center dot 0%] of 8959) than patients without cancer (129 291 [48 center dot 3%] of 267 520; OR 1 center dot 20 [1 center dot 15-1 center dot 25]). For Enterococcus spp, vancomycin resistance was higher in pathogen isolates from patients with cancer (1329 [18 center dot 6%] of 7145) than patients without cancer (12 333 [9 center dot 1%] of 135 772]; ORR 2 center dot 20 [2 center dot 06-2 center dot 34). The rates and corresponding IRRs of AMR pathogens per 1000 isolates was also higher in patients with cancer compared with patients without cancer, particularly for carbapenem non-susceptible P aeruginosa (IRR 2 center dot 06 [1 center dot 91-2 center dot 21]) and vancomycin-resistant enterococci (IRR 3 center dot 06 [2 center dot 89-3 center dot 24]). For all comparisons, p<0 center dot 0001. Interpretation AMR proportions and IRRs for most key pathogens were up to three-times higher in isolates from outpatients with cancer than those without cancer, highlighting the need for enhanced surveillance, infection prevention, and timely diagnostic stewardship to improve antibiotic prescribing in this population. Copyright (c) 2025 Elsevier Ltd. All rights reserved, including those for text and data mining, AI training, and similar technologies. | Based on the provided abstract, a study comparing antimicrobial resistance (AMR) in outpatients with and without cancer found that patients with cancer had a higher frequency of resistant bacteria. Specifically, the study found that the proportion of non-susceptible pathogens was up to three times higher in patients with cancer. For example, carbapenem non-susceptibility in Pseudomonas aeruginosa was 14.4% in patients with cancer compared to 11.3% in those without. Similarly, vancomycin resistance in Enterococcus spp was 18.6% in the cancer group versus 9.1% in the non-cancer group. These findings highlight the need for improved infection control and antibiotic management for cancer patients. | A recent study looked at how common antibiotic-resistant bacteria are in outpatients with and without cancer. Researchers found that patients with cancer had a much higher frequency of these resistant bacteria compared to those without cancer. For instance, the study found that for certain types of bacteria, the resistance rates were up to three times higher in cancer patients. This suggests a greater need for monitoring and careful antibiotic use to prevent infections in this group. | A study analyzing data from outpatients with and without cancer found that antimicrobial resistance (AMR) is significantly more common in cancer patients. Researchers compared the rates of resistant bacteria in over 27,000 cancer patients to over 928,000 patients without cancer. They found that for various common bacteria, the odds of a pathogen being resistant to antibiotics were up to three times higher in samples from cancer patients. This finding suggests that there is a need for better infection prevention and more careful use of antibiotics in this patient group. |
| CancerRes | 4 | 10.1016/S1470-2045(25)00128-11 | DeepSeek | Background Infections are the second leading cause of death in patients with cancer and are often caused by resistant bacteria. However, the frequency of antimicrobial resistance (AMR) in outpatients with cancer is not well understood. We aimed to compare the frequency of AMR bacterial pathogens in outpatients with and without cancer. Methods This retrospective cohort study evaluated antimicrobial susceptibility of bacteria isolated from adults (aged >= 18 years) with and without cancer seeking care in 198 outpatient health-care settings in the USA. Data were collected using the BD Insights Research Database. Patients who were not prescribed cancer medications or not admitted to an inpatient cancer unit in the predefined period were categorised as patients without cancer. Patients were included in the cancer cohort if they received medication solely or sometimes indicated for cancer. Data on gender and race or ethnicity were not collected. Non-duplicate and non-contaminant pathogens collected from various samples (ie, blood, intra-abdominal, respiratory, urine, skin or wound, and other) in outpatients were used to assess the coprimary outcomes: overall and source-specific proportions of non-susceptible pathogen isolates with corresponding AMR odds ratios (ORs); and rates of AMR pathogens per 1000 isolates with corresponding AMR incidence rate ratio (IRR) in patients with and without cancer. Findings Data were collected between April 1, 2018, and Dec 31, 2022. 53 006 (3 center dot 2%) of 1 655 594 pathogens identified were from 27 421 patients with cancer and 1 602 588 (96 center dot 8%) were from 928 128 patients without cancer. For Pseudomonas aeruginosa, carbapenem non-susceptibility was higher in pathogen isolates from patients with cancer (816 [14 center dot 4%] of 5683) than patients without cancer (10 709 [11 center dot 3%] 94 419; OR 1 center dot 22 [95% CI 1 center dot 13-1 center dot 32]). For Enterobacterales, fluoroquinolone non-susceptibility was higher in pathogen isolates from patients with cancer (8662 [28 center dot 0%] of 30 867) than patients without cancer (238 479 [21 center dot 8%] of 1 095 996; OR 1 center dot 44 [1 center dot 40-1 center dot 47]), as was carbapenem non-susceptibility (472 [1 center dot 5%] of 30 867 vs 9165 [0 center dot 8%] of 1 095 996; OR 1 center dot 89 [1 center dot 72-2 center dot 07]), multidrug-resistant pathogens (2672 [8 center dot 7%] of 30 867 vs 48 962 [4 center dot 5%] of 1 095 996; OR 2 center dot 03 [1 center dot 95-2 center dot 11]), and extended-spectrum beta-lactamase producers (4343 [16 center dot 5%] of 26 327 vs 93 977 [9 center dot 4%] of 996 853; OR 1 center dot 96 [1 center dot 90-2 center dot 03]). For Staphylococcus aureus, meticillin resistance was higher in pathogen isolates from patients with cancer (4747 [53 center dot 0%] of 8959) than patients without cancer (129 291 [48 center dot 3%] of 267 520; OR 1 center dot 20 [1 center dot 15-1 center dot 25]). For Enterococcus spp, vancomycin resistance was higher in pathogen isolates from patients with cancer (1329 [18 center dot 6%] of 7145) than patients without cancer (12 333 [9 center dot 1%] of 135 772]; ORR 2 center dot 20 [2 center dot 06-2 center dot 34). The rates and corresponding IRRs of AMR pathogens per 1000 isolates was also higher in patients with cancer compared with patients without cancer, particularly for carbapenem non-susceptible P aeruginosa (IRR 2 center dot 06 [1 center dot 91-2 center dot 21]) and vancomycin-resistant enterococci (IRR 3 center dot 06 [2 center dot 89-3 center dot 24]). For all comparisons, p<0 center dot 0001. Interpretation AMR proportions and IRRs for most key pathogens were up to three-times higher in isolates from outpatients with cancer than those without cancer, highlighting the need for enhanced surveillance, infection prevention, and timely diagnostic stewardship to improve antibiotic prescribing in this population. Copyright (c) 2025 Elsevier Ltd. All rights reserved, including those for text and data mining, AI training, and similar technologies. | This study compared antibiotic-resistant infections in outpatients with and without cancer in the U.S. from 2018 to 2022. It found that cancer patients had higher rates of resistant bacteria, including harder-to-treat Pseudomonas, Enterobacterales, Staphylococcus aureus, and Enterococcus. For example, resistance to carbapenems (a strong antibiotic) was 1.5 times higher in cancer patients for some bacteria, and vancomycin-resistant Enterococcus was over twice as common. These results show that cancer patients are at greater risk for resistant infections, highlighting the need for better infection control and smarter antibiotic use in this vulnerable group. | This study compared antibiotic-resistant infections in outpatients with and without cancer in the U.S. from 2018 to 2022. It found that cancer patients had higher rates of resistant bacteria, including harder-to-treat Pseudomonas, Enterobacterales, Staphylococcus aureus, and Enterococcus. For example, resistance to carbapenems and vancomycin was 1.2 to 3 times more common in cancer patients. These results show that cancer outpatients face greater risks from antibiotic-resistant infections, calling for better monitoring, prevention, and smarter antibiotic use to protect them. | This study compared antibiotic-resistant infections in outpatients with and without cancer in the U.S. from 2018 to 2022. It found that cancer patients had higher rates of resistant bacteria, including harder-to-treat Pseudomonas, Enterobacterales, Staphylococcus aureus, and Enterococcus. For example, carbapenem-resistant Pseudomonas and vancomycin-resistant Enterococcus were 2–3 times more common in cancer patients. These results show that cancer outpatients face greater risks from antibiotic-resistant infections, calling for better monitoring, prevention, and smarter antibiotic use to protect this vulnerable group. |
| CancerRes | 4 | 10.1016/S1470-2045(25)00128-12 | Grok | Background Infections are the second leading cause of death in patients with cancer and are often caused by resistant bacteria. However, the frequency of antimicrobial resistance (AMR) in outpatients with cancer is not well understood. We aimed to compare the frequency of AMR bacterial pathogens in outpatients with and without cancer. Methods This retrospective cohort study evaluated antimicrobial susceptibility of bacteria isolated from adults (aged >= 18 years) with and without cancer seeking care in 198 outpatient health-care settings in the USA. Data were collected using the BD Insights Research Database. Patients who were not prescribed cancer medications or not admitted to an inpatient cancer unit in the predefined period were categorised as patients without cancer. Patients were included in the cancer cohort if they received medication solely or sometimes indicated for cancer. Data on gender and race or ethnicity were not collected. Non-duplicate and non-contaminant pathogens collected from various samples (ie, blood, intra-abdominal, respiratory, urine, skin or wound, and other) in outpatients were used to assess the coprimary outcomes: overall and source-specific proportions of non-susceptible pathogen isolates with corresponding AMR odds ratios (ORs); and rates of AMR pathogens per 1000 isolates with corresponding AMR incidence rate ratio (IRR) in patients with and without cancer. Findings Data were collected between April 1, 2018, and Dec 31, 2022. 53 006 (3 center dot 2%) of 1 655 594 pathogens identified were from 27 421 patients with cancer and 1 602 588 (96 center dot 8%) were from 928 128 patients without cancer. For Pseudomonas aeruginosa, carbapenem non-susceptibility was higher in pathogen isolates from patients with cancer (816 [14 center dot 4%] of 5683) than patients without cancer (10 709 [11 center dot 3%] 94 419; OR 1 center dot 22 [95% CI 1 center dot 13-1 center dot 32]). For Enterobacterales, fluoroquinolone non-susceptibility was higher in pathogen isolates from patients with cancer (8662 [28 center dot 0%] of 30 867) than patients without cancer (238 479 [21 center dot 8%] of 1 095 996; OR 1 center dot 44 [1 center dot 40-1 center dot 47]), as was carbapenem non-susceptibility (472 [1 center dot 5%] of 30 867 vs 9165 [0 center dot 8%] of 1 095 996; OR 1 center dot 89 [1 center dot 72-2 center dot 07]), multidrug-resistant pathogens (2672 [8 center dot 7%] of 30 867 vs 48 962 [4 center dot 5%] of 1 095 996; OR 2 center dot 03 [1 center dot 95-2 center dot 11]), and extended-spectrum beta-lactamase producers (4343 [16 center dot 5%] of 26 327 vs 93 977 [9 center dot 4%] of 996 853; OR 1 center dot 96 [1 center dot 90-2 center dot 03]). For Staphylococcus aureus, meticillin resistance was higher in pathogen isolates from patients with cancer (4747 [53 center dot 0%] of 8959) than patients without cancer (129 291 [48 center dot 3%] of 267 520; OR 1 center dot 20 [1 center dot 15-1 center dot 25]). For Enterococcus spp, vancomycin resistance was higher in pathogen isolates from patients with cancer (1329 [18 center dot 6%] of 7145) than patients without cancer (12 333 [9 center dot 1%] of 135 772]; ORR 2 center dot 20 [2 center dot 06-2 center dot 34). The rates and corresponding IRRs of AMR pathogens per 1000 isolates was also higher in patients with cancer compared with patients without cancer, particularly for carbapenem non-susceptible P aeruginosa (IRR 2 center dot 06 [1 center dot 91-2 center dot 21]) and vancomycin-resistant enterococci (IRR 3 center dot 06 [2 center dot 89-3 center dot 24]). For all comparisons, p<0 center dot 0001. Interpretation AMR proportions and IRRs for most key pathogens were up to three-times higher in isolates from outpatients with cancer than those without cancer, highlighting the need for enhanced surveillance, infection prevention, and timely diagnostic stewardship to improve antibiotic prescribing in this population. Copyright (c) 2025 Elsevier Ltd. All rights reserved, including those for text and data mining, AI training, and similar technologies. | This study compared bacterial infections resistant to antibiotics in outpatients with and without cancer across 198 U.S. healthcare settings from April 2018 to December 2022. It found that bacteria from patients with cancer were more likely to resist common antibiotics, such as carbapenems, fluoroquinolones, and vancomycin, compared to those without cancer. For example, resistance was higher for pathogens like Pseudomonas aeruginosa, Enterobacterales, Staphylococcus aureus, and Enterococcus spp., with resistance rates up to three times higher in cancer patients. These findings suggest a need for better monitoring, infection prevention, and careful antibiotic use in outpatients with cancer. | A study conducted from April 2018 to December 2022 in the USA compared bacterial infections in outpatients with and without cancer to understand antimicrobial resistance (AMR). It found that bacteria from cancer patients were more resistant to antibiotics, including carbapenems, fluoroquinolones, and vancomycin, with resistance rates up to three times higher than in patients without cancer. This suggests that cancer patients face a higher risk of hard-to-treat infections, emphasizing the need for better monitoring, prevention, and careful antibiotic use in this group. | This study compared bacterial infections in outpatients with and without cancer in the USA from 2018 to 2022, focusing on antimicrobial resistance (AMR). It found that bacteria from patients with cancer were more likely to resist common antibiotics, including carbapenems, fluoroquinolones, and vancomycin, with resistance rates up to three times higher than in patients without cancer. This suggests that outpatients with cancer face a higher risk of hard-to-treat infections, emphasizing the need for better monitoring, infection prevention, and careful antibiotic use in this group. |
| CancerRes | 5 | 10.1016/S1470-2045(25)00006-4 | Open AI | Background The phase 2 randomised Young-PEARL study demonstrated that palbociclib plus exemestane with ovarian function suppression significantly prolonged progression-free survival compared with capecitabine in premenopausal women with hormone receptor-positive, HER2-negative metastatic breast cancer. Here, we report results of the protocol-specified secondary endpoint of overall survival. Methods Young-PEARL was a multicentre, randomised, open-label, phase 2 study conducted at 14 institutions in South Korea. Premenopausal women aged 19 years or older with histologically confirmed hormone receptor-positive, HER2-negative metastatic breast cancer that recurred or progressed during or after previous tamoxifen treatment, who were aromatase inhibitor naive, and had an Eastern Cooperative Oncology Group performance status of 0-2 were eligible. One previous line of chemotherapy was permitted in the metastatic setting. Eligible patients were randomly assigned (1:1), using block randomisation (block size of two) stratified by previous chemotherapy for metastatic breast cancer and presence of visceral metastasis, to receive either palbociclib (orally, 125 mg per day on a 3-weeks-on, 1-week off schedule) plus exemestane (orally 25 mg daily) with leuprorelin (subcutaneously 375 mg on day 1 of each 28-day cycle) or capecitabine (orally, 1250 mg/m(2) twice a day on a 2-weeks-on, 1-week-off schedule) until disease progression or unacceptable toxicity). The primary endpoint was progression-free survival. Overall survival was a secondary endpoint. All analyses were done in the modified intention-to-treat population (ie, included all patients randomly assigned to treatment who had at least one post-baseline CT scan and excluded those who did not receive study medication and who had any major violation of the eligible criteria). Safety was assessed in all patients who received any study treatment. This study is registered with ClinicalTrials.gov, NCT02592746, and is now complete. Findings Between June 15, 2016, and Dec 10, 2018, 189 patients were enrolled. 184 patients were randomly assigned to the palbociclib plus endocrine therapy group (n=92) or the capecitabine group (n=92), of whom 174 were included in the modified intention-to-treat population (n=90 in the palbociclib plus endocrine therapy group and n=84 in the capecitabine group). All patients were female and ethnicity data were not collected. As of data cutoff (Feb 29, 2024), median follow-up was 540 months (IQR 341-744). Median progression-free survival was 195 months (90% CI 143-222) for palbociclib plus endocrine therapy and 140 months (117-187) for capecitabine (hazard ratio 074 [90% CI 057-098]; one-sided log-rank p=0036). 52 (58%) of 90 patients in the palbociclib plus endocrine therapy group and 48 (57%) of 84 in the capecitabine group died, with a median overall survival of 548 months (95% CI 489-771) in the palbociclib plus endocrine therapy group versus 578 months (463-892) in the capecitabine group (hazard ratio 102 [95% CI 069-151]; p=092). The most common grade 3 or worse adverse event was neutropenia (59 [64%] of 92 in the palbociclib plus endocrine therapy group vs 15 [18%] of 85 in the capecitabine group) . No treatment-related deaths occurred. Interpretation With extended follow-up, palbociclib plus exemestane with ovarian function suppression continued to show a significant benefit in progression-free survival compared with capecitabine in premenopausal patients with hormone receptor-positive, HER2-negative metastatic breast cancer who had been previously treated with tamoxifen; however, no improvement in overall survival was seen. Given the progression-free survival benefit, the upfront use of palbociclib plus endocrine therapy is the preferred option for premenopausal women, although a capecitabine-first strategy might be an alternative treatment strategy for maintaining overall survival in resource-limited settings. Copyright (c) 2025 Elsevier Ltd. All rights reserved, including those for text and data mining, AI training, and similar technologies. | The Young-PEARL study tested two treatments for premenopausal women with hormone receptor-positive, HER2-negative metastatic breast cancer: palbociclib plus exemestane with ovarian suppression versus capecitabine. The study found that palbociclib with endocrine therapy significantly delayed disease progression compared to capecitabine, but it did not improve overall survival. Side effects were more common with palbociclib, especially low white blood cell counts, but no treatment-related deaths occurred. This suggests that palbociclib plus endocrine therapy is preferred for delaying progression, while capecitabine could be considered when resources are limited. | The Young-PEARL study tested palbociclib plus exemestane with ovarian suppression versus capecitabine in premenopausal women with a specific type of advanced breast cancer. The treatment with palbociclib and endocrine therapy helped women live longer without the cancer getting worse, but it did not increase overall survival compared with capecitabine. The main side effect was low white blood cell counts, and no treatment-related deaths occurred. Overall, palbociclib plus endocrine therapy is preferred to delay cancer progression, though capecitabine may still be used in some settings. | The Young-PEARL study tested treatments in premenopausal women with hormone receptor-positive, HER2-negative metastatic breast cancer. It found that palbociclib plus exemestane with ovarian suppression helped patients live longer without their cancer worsening compared with capecitabine, but it did not improve overall survival. Both treatments were generally safe, though palbociclib caused more cases of low white blood cell counts. Because it delays disease progression, palbociclib plus endocrine therapy is usually preferred, but capecitabine may be an option where resources are limited. |
| CancerRes | 5 | 10.1016/S1470-2045(25)00006-5 | Claude | Background The phase 2 randomised Young-PEARL study demonstrated that palbociclib plus exemestane with ovarian function suppression significantly prolonged progression-free survival compared with capecitabine in premenopausal women with hormone receptor-positive, HER2-negative metastatic breast cancer. Here, we report results of the protocol-specified secondary endpoint of overall survival. Methods Young-PEARL was a multicentre, randomised, open-label, phase 2 study conducted at 14 institutions in South Korea. Premenopausal women aged 19 years or older with histologically confirmed hormone receptor-positive, HER2-negative metastatic breast cancer that recurred or progressed during or after previous tamoxifen treatment, who were aromatase inhibitor naive, and had an Eastern Cooperative Oncology Group performance status of 0-2 were eligible. One previous line of chemotherapy was permitted in the metastatic setting. Eligible patients were randomly assigned (1:1), using block randomisation (block size of two) stratified by previous chemotherapy for metastatic breast cancer and presence of visceral metastasis, to receive either palbociclib (orally, 125 mg per day on a 3-weeks-on, 1-week off schedule) plus exemestane (orally 25 mg daily) with leuprorelin (subcutaneously 375 mg on day 1 of each 28-day cycle) or capecitabine (orally, 1250 mg/m(2) twice a day on a 2-weeks-on, 1-week-off schedule) until disease progression or unacceptable toxicity). The primary endpoint was progression-free survival. Overall survival was a secondary endpoint. All analyses were done in the modified intention-to-treat population (ie, included all patients randomly assigned to treatment who had at least one post-baseline CT scan and excluded those who did not receive study medication and who had any major violation of the eligible criteria). Safety was assessed in all patients who received any study treatment. This study is registered with ClinicalTrials.gov, NCT02592746, and is now complete. Findings Between June 15, 2016, and Dec 10, 2018, 189 patients were enrolled. 184 patients were randomly assigned to the palbociclib plus endocrine therapy group (n=92) or the capecitabine group (n=92), of whom 174 were included in the modified intention-to-treat population (n=90 in the palbociclib plus endocrine therapy group and n=84 in the capecitabine group). All patients were female and ethnicity data were not collected. As of data cutoff (Feb 29, 2024), median follow-up was 540 months (IQR 341-744). Median progression-free survival was 195 months (90% CI 143-222) for palbociclib plus endocrine therapy and 140 months (117-187) for capecitabine (hazard ratio 074 [90% CI 057-098]; one-sided log-rank p=0036). 52 (58%) of 90 patients in the palbociclib plus endocrine therapy group and 48 (57%) of 84 in the capecitabine group died, with a median overall survival of 548 months (95% CI 489-771) in the palbociclib plus endocrine therapy group versus 578 months (463-892) in the capecitabine group (hazard ratio 102 [95% CI 069-151]; p=092). The most common grade 3 or worse adverse event was neutropenia (59 [64%] of 92 in the palbociclib plus endocrine therapy group vs 15 [18%] of 85 in the capecitabine group) . No treatment-related deaths occurred. Interpretation With extended follow-up, palbociclib plus exemestane with ovarian function suppression continued to show a significant benefit in progression-free survival compared with capecitabine in premenopausal patients with hormone receptor-positive, HER2-negative metastatic breast cancer who had been previously treated with tamoxifen; however, no improvement in overall survival was seen. Given the progression-free survival benefit, the upfront use of palbociclib plus endocrine therapy is the preferred option for premenopausal women, although a capecitabine-first strategy might be an alternative treatment strategy for maintaining overall survival in resource-limited settings. Copyright (c) 2025 Elsevier Ltd. All rights reserved, including those for text and data mining, AI training, and similar technologies. | This study followed premenopausal women with a specific type of breast cancer (hormone receptor-positive, HER2-negative) that had spread to other parts of the body. Researchers compared two treatment approaches: one group received a combination of palbociclib (a targeted therapy drug) plus hormone therapy, while the other group received capecitabine (a chemotherapy drug). The combination therapy was better at delaying cancer progression - patients stayed cancer-free for about 19.5 months compared to 14 months with chemotherapy alone. However, when looking at overall survival (how long patients lived), both treatments performed similarly, with patients living about 55-58 months on average. The combination therapy caused more severe side effects like low white blood cell counts, but the progression-free survival benefit makes it the preferred first treatment option for this group of patients. | This study followed premenopausal women with a specific type of breast cancer (hormone receptor-positive, HER2-negative) that had spread to other parts of their body. Researchers compared two treatment approaches: one using a combination of palbociclib (a targeted cancer drug) plus hormone therapy, and another using capecitabine (a chemotherapy drug). While the combination treatment was better at delaying cancer progression (keeping the cancer stable for about 5.5 months longer), it didn't help patients live longer overall - both groups had similar survival times of around 4-5 years. The combination treatment did cause more severe side effects, particularly low white blood cell counts. The researchers concluded that while the combination therapy is still the preferred first treatment because it delays progression, using chemotherapy first might be a reasonable alternative, especially in areas with limited healthcare resources. | This study followed premenopausal women with a specific type of breast cancer (hormone receptor-positive, HER2-negative) that had spread to other parts of the body. Researchers compared two treatment approaches: one group received a combination of palbociclib (a targeted drug), exemestane (a hormone therapy), and ovarian suppression, while the other group received capecitabine (a chemotherapy drug). The results showed that the combination treatment helped delay cancer progression for about 5.5 months longer than chemotherapy alone (19.5 months vs 14 months). However, when looking at overall survival - how long patients lived - both treatments performed similarly, with patients living about the same amount of time regardless of which treatment they received. The combination treatment caused more severe drops in white blood cell counts, but both treatments were generally safe with no treatment-related deaths. |
| CancerRes | 5 | 10.1016/S1470-2045(25)00006-6 | Gemini | Background The phase 2 randomised Young-PEARL study demonstrated that palbociclib plus exemestane with ovarian function suppression significantly prolonged progression-free survival compared with capecitabine in premenopausal women with hormone receptor-positive, HER2-negative metastatic breast cancer. Here, we report results of the protocol-specified secondary endpoint of overall survival. Methods Young-PEARL was a multicentre, randomised, open-label, phase 2 study conducted at 14 institutions in South Korea. Premenopausal women aged 19 years or older with histologically confirmed hormone receptor-positive, HER2-negative metastatic breast cancer that recurred or progressed during or after previous tamoxifen treatment, who were aromatase inhibitor naive, and had an Eastern Cooperative Oncology Group performance status of 0-2 were eligible. One previous line of chemotherapy was permitted in the metastatic setting. Eligible patients were randomly assigned (1:1), using block randomisation (block size of two) stratified by previous chemotherapy for metastatic breast cancer and presence of visceral metastasis, to receive either palbociclib (orally, 125 mg per day on a 3-weeks-on, 1-week off schedule) plus exemestane (orally 25 mg daily) with leuprorelin (subcutaneously 375 mg on day 1 of each 28-day cycle) or capecitabine (orally, 1250 mg/m(2) twice a day on a 2-weeks-on, 1-week-off schedule) until disease progression or unacceptable toxicity). The primary endpoint was progression-free survival. Overall survival was a secondary endpoint. All analyses were done in the modified intention-to-treat population (ie, included all patients randomly assigned to treatment who had at least one post-baseline CT scan and excluded those who did not receive study medication and who had any major violation of the eligible criteria). Safety was assessed in all patients who received any study treatment. This study is registered with ClinicalTrials.gov, NCT02592746, and is now complete. Findings Between June 15, 2016, and Dec 10, 2018, 189 patients were enrolled. 184 patients were randomly assigned to the palbociclib plus endocrine therapy group (n=92) or the capecitabine group (n=92), of whom 174 were included in the modified intention-to-treat population (n=90 in the palbociclib plus endocrine therapy group and n=84 in the capecitabine group). All patients were female and ethnicity data were not collected. As of data cutoff (Feb 29, 2024), median follow-up was 540 months (IQR 341-744). Median progression-free survival was 195 months (90% CI 143-222) for palbociclib plus endocrine therapy and 140 months (117-187) for capecitabine (hazard ratio 074 [90% CI 057-098]; one-sided log-rank p=0036). 52 (58%) of 90 patients in the palbociclib plus endocrine therapy group and 48 (57%) of 84 in the capecitabine group died, with a median overall survival of 548 months (95% CI 489-771) in the palbociclib plus endocrine therapy group versus 578 months (463-892) in the capecitabine group (hazard ratio 102 [95% CI 069-151]; p=092). The most common grade 3 or worse adverse event was neutropenia (59 [64%] of 92 in the palbociclib plus endocrine therapy group vs 15 [18%] of 85 in the capecitabine group) . No treatment-related deaths occurred. Interpretation With extended follow-up, palbociclib plus exemestane with ovarian function suppression continued to show a significant benefit in progression-free survival compared with capecitabine in premenopausal patients with hormone receptor-positive, HER2-negative metastatic breast cancer who had been previously treated with tamoxifen; however, no improvement in overall survival was seen. Given the progression-free survival benefit, the upfront use of palbociclib plus endocrine therapy is the preferred option for premenopausal women, although a capecitabine-first strategy might be an alternative treatment strategy for maintaining overall survival in resource-limited settings. Copyright (c) 2025 Elsevier Ltd. All rights reserved, including those for text and data mining, AI training, and similar technologies. | In a study comparing two treatments for a specific type of breast cancer in premenopausal women, a combination of palbociclib and hormone therapy was found to slow the progression of the disease significantly more than a capecitabine-only treatment. However, after an extended follow-up, there was no difference in overall survival between the two groups. The most common severe side effect of the palbociclib and hormone therapy combination was a low white blood cell count. Due to the benefit in delaying cancer progression, the combination treatment is generally preferred, although the capecitabine-only treatment could be a good alternative, especially in places where resources are limited. | In a study called Young-PEARL, researchers compared two treatments for premenopausal women with a specific type of advanced breast cancer. The first group received a combination of medications (palbociclib plus endocrine therapy), while the second group received capecitabine. The study found that the palbociclib combination significantly delayed the time it took for the cancer to progress. However, when it came to overall survival, there was no significant difference between the two treatments. The most common severe side effect in the palbociclib group was a low white blood cell count (neutropenia). Based on these results, doctors generally prefer the palbociclib combination because it keeps the cancer from progressing for a longer period, but capecitabine is also a good option, especially where resources may be limited, as it provides a similar overall survival outcome. | In a study comparing two treatments for a specific type of breast cancer in premenopausal women, a combination of palbociclib plus endocrine therapy was found to be more effective at slowing the cancer's progression than the drug capecitabine. The study followed 174 women and found that those who took palbociclib plus endocrine therapy had a longer time before their disease worsened (a median of 19.5 months) compared to those who took capecitabine (a median of 14.0 months). However, despite this benefit, both groups of women lived for about the same amount of time overall. The most common severe side effect for the palbociclib group was a low white blood cell count, called neutropenia. The researchers concluded that the palbociclib combination is the preferred initial treatment due to the progression-free survival benefit. |
| CancerRes | 5 | 10.1016/S1470-2045(25)00006-7 | DeepSeek | Background The phase 2 randomised Young-PEARL study demonstrated that palbociclib plus exemestane with ovarian function suppression significantly prolonged progression-free survival compared with capecitabine in premenopausal women with hormone receptor-positive, HER2-negative metastatic breast cancer. Here, we report results of the protocol-specified secondary endpoint of overall survival. Methods Young-PEARL was a multicentre, randomised, open-label, phase 2 study conducted at 14 institutions in South Korea. Premenopausal women aged 19 years or older with histologically confirmed hormone receptor-positive, HER2-negative metastatic breast cancer that recurred or progressed during or after previous tamoxifen treatment, who were aromatase inhibitor naive, and had an Eastern Cooperative Oncology Group performance status of 0-2 were eligible. One previous line of chemotherapy was permitted in the metastatic setting. Eligible patients were randomly assigned (1:1), using block randomisation (block size of two) stratified by previous chemotherapy for metastatic breast cancer and presence of visceral metastasis, to receive either palbociclib (orally, 125 mg per day on a 3-weeks-on, 1-week off schedule) plus exemestane (orally 25 mg daily) with leuprorelin (subcutaneously 375 mg on day 1 of each 28-day cycle) or capecitabine (orally, 1250 mg/m(2) twice a day on a 2-weeks-on, 1-week-off schedule) until disease progression or unacceptable toxicity). The primary endpoint was progression-free survival. Overall survival was a secondary endpoint. All analyses were done in the modified intention-to-treat population (ie, included all patients randomly assigned to treatment who had at least one post-baseline CT scan and excluded those who did not receive study medication and who had any major violation of the eligible criteria). Safety was assessed in all patients who received any study treatment. This study is registered with ClinicalTrials.gov, NCT02592746, and is now complete. Findings Between June 15, 2016, and Dec 10, 2018, 189 patients were enrolled. 184 patients were randomly assigned to the palbociclib plus endocrine therapy group (n=92) or the capecitabine group (n=92), of whom 174 were included in the modified intention-to-treat population (n=90 in the palbociclib plus endocrine therapy group and n=84 in the capecitabine group). All patients were female and ethnicity data were not collected. As of data cutoff (Feb 29, 2024), median follow-up was 540 months (IQR 341-744). Median progression-free survival was 195 months (90% CI 143-222) for palbociclib plus endocrine therapy and 140 months (117-187) for capecitabine (hazard ratio 074 [90% CI 057-098]; one-sided log-rank p=0036). 52 (58%) of 90 patients in the palbociclib plus endocrine therapy group and 48 (57%) of 84 in the capecitabine group died, with a median overall survival of 548 months (95% CI 489-771) in the palbociclib plus endocrine therapy group versus 578 months (463-892) in the capecitabine group (hazard ratio 102 [95% CI 069-151]; p=092). The most common grade 3 or worse adverse event was neutropenia (59 [64%] of 92 in the palbociclib plus endocrine therapy group vs 15 [18%] of 85 in the capecitabine group) . No treatment-related deaths occurred. Interpretation With extended follow-up, palbociclib plus exemestane with ovarian function suppression continued to show a significant benefit in progression-free survival compared with capecitabine in premenopausal patients with hormone receptor-positive, HER2-negative metastatic breast cancer who had been previously treated with tamoxifen; however, no improvement in overall survival was seen. Given the progression-free survival benefit, the upfront use of palbociclib plus endocrine therapy is the preferred option for premenopausal women, although a capecitabine-first strategy might be an alternative treatment strategy for maintaining overall survival in resource-limited settings. Copyright (c) 2025 Elsevier Ltd. All rights reserved, including those for text and data mining, AI training, and similar technologies. | The Young-PEARL study compared two treatments for premenopausal women with hormone-sensitive, HER2-negative metastatic breast cancer: palbociclib (a targeted therapy) plus hormone therapy versus capecitabine (chemotherapy). While palbociclib combined with hormone therapy delayed cancer progression longer than capecitabine (19.5 vs. 14 months), both treatments showed similar overall survival (around 55-58 months). Side effects differed, with palbociclib causing more neutropenia. Though palbociclib is preferred upfront for delaying progression, capecitabine remains an alternative where resources are limited. No treatment-related deaths occurred. | The Young-PEARL study compared two treatments for premenopausal women with hormone-sensitive, HER2-negative metastatic breast cancer: palbociclib (a targeted therapy) plus hormone-blocking drugs versus chemotherapy (capecitabine). The study found that palbociclib combo delayed cancer progression longer (19.5 vs. 14 months) but did not improve overall survival (54.8 vs. 57.8 months). Side effects differed, with more neutropenia in the palbociclib group. While palbociclib is preferred upfront for delaying progression, capecitabine remains an alternative where resources are limited. | The Young-PEARL study compared two treatments for premenopausal women with hormone receptor-positive, HER2-negative metastatic breast cancer: palbociclib plus endocrine therapy (exemestane and ovarian suppression) versus capecitabine chemotherapy. While palbociclib plus endocrine therapy improved progression-free survival (19.5 vs. 14.0 months) compared to capecitabine, there was no difference in overall survival (54.8 vs. 57.8 months). Side effects like neutropenia were more common with palbociclib, but no treatment-related deaths occurred. The study suggests palbociclib plus endocrine therapy is a preferred first option, though capecitabine remains a reasonable alternative, especially where resources are limited. |
| CancerRes | 5 | 10.1016/S1470-2045(25)00006-8 | Grok | Background The phase 2 randomised Young-PEARL study demonstrated that palbociclib plus exemestane with ovarian function suppression significantly prolonged progression-free survival compared with capecitabine in premenopausal women with hormone receptor-positive, HER2-negative metastatic breast cancer. Here, we report results of the protocol-specified secondary endpoint of overall survival. Methods Young-PEARL was a multicentre, randomised, open-label, phase 2 study conducted at 14 institutions in South Korea. Premenopausal women aged 19 years or older with histologically confirmed hormone receptor-positive, HER2-negative metastatic breast cancer that recurred or progressed during or after previous tamoxifen treatment, who were aromatase inhibitor naive, and had an Eastern Cooperative Oncology Group performance status of 0-2 were eligible. One previous line of chemotherapy was permitted in the metastatic setting. Eligible patients were randomly assigned (1:1), using block randomisation (block size of two) stratified by previous chemotherapy for metastatic breast cancer and presence of visceral metastasis, to receive either palbociclib (orally, 125 mg per day on a 3-weeks-on, 1-week off schedule) plus exemestane (orally 25 mg daily) with leuprorelin (subcutaneously 375 mg on day 1 of each 28-day cycle) or capecitabine (orally, 1250 mg/m(2) twice a day on a 2-weeks-on, 1-week-off schedule) until disease progression or unacceptable toxicity). The primary endpoint was progression-free survival. Overall survival was a secondary endpoint. All analyses were done in the modified intention-to-treat population (ie, included all patients randomly assigned to treatment who had at least one post-baseline CT scan and excluded those who did not receive study medication and who had any major violation of the eligible criteria). Safety was assessed in all patients who received any study treatment. This study is registered with ClinicalTrials.gov, NCT02592746, and is now complete. Findings Between June 15, 2016, and Dec 10, 2018, 189 patients were enrolled. 184 patients were randomly assigned to the palbociclib plus endocrine therapy group (n=92) or the capecitabine group (n=92), of whom 174 were included in the modified intention-to-treat population (n=90 in the palbociclib plus endocrine therapy group and n=84 in the capecitabine group). All patients were female and ethnicity data were not collected. As of data cutoff (Feb 29, 2024), median follow-up was 540 months (IQR 341-744). Median progression-free survival was 195 months (90% CI 143-222) for palbociclib plus endocrine therapy and 140 months (117-187) for capecitabine (hazard ratio 074 [90% CI 057-098]; one-sided log-rank p=0036). 52 (58%) of 90 patients in the palbociclib plus endocrine therapy group and 48 (57%) of 84 in the capecitabine group died, with a median overall survival of 548 months (95% CI 489-771) in the palbociclib plus endocrine therapy group versus 578 months (463-892) in the capecitabine group (hazard ratio 102 [95% CI 069-151]; p=092). The most common grade 3 or worse adverse event was neutropenia (59 [64%] of 92 in the palbociclib plus endocrine therapy group vs 15 [18%] of 85 in the capecitabine group) . No treatment-related deaths occurred. Interpretation With extended follow-up, palbociclib plus exemestane with ovarian function suppression continued to show a significant benefit in progression-free survival compared with capecitabine in premenopausal patients with hormone receptor-positive, HER2-negative metastatic breast cancer who had been previously treated with tamoxifen; however, no improvement in overall survival was seen. Given the progression-free survival benefit, the upfront use of palbociclib plus endocrine therapy is the preferred option for premenopausal women, although a capecitabine-first strategy might be an alternative treatment strategy for maintaining overall survival in resource-limited settings. Copyright (c) 2025 Elsevier Ltd. All rights reserved, including those for text and data mining, AI training, and similar technologies. | The Young-PEARL study compared two treatments for premenopausal women with hormone receptor-positive, HER2-negative metastatic breast cancer who had previously used tamoxifen. In this phase 2 trial, 184 women were split into two groups: one received palbociclib plus exemestane and leuprorelin, while the other got capecitabine. After about 54 months, the palbociclib group had longer progression-free survival (19.5 months vs. 14 months), meaning their cancer was controlled longer. However, overall survival was similar (54.8 months for palbociclib vs. 57.8 months for capecitabine). Neutropenia was the most common severe side effect, more frequent in the palbociclib group. The study suggests palbociclib with endocrine therapy is better for delaying cancer progression, but capecitabine could be an option where resources are limited. | The Young-PEARL study compared two treatments for premenopausal women with hormone receptor-positive, HER2-negative metastatic breast cancer who had previously used tamoxifen. In this study, 184 women were split into two groups: one received palbociclib plus exemestane with ovarian suppression, and the other got capecitabine. After a median follow-up of 54 months, the palbociclib group had longer progression-free survival (19.5 months vs. 14 months), meaning the disease was controlled for longer. However, overall survival was similar between the groups (54.8 months for palbociclib vs. 57.8 months for capecitabine). Neutropenia was the most common serious side effect, more frequent in the palbociclib group. The study suggests palbociclib with endocrine therapy is better for delaying disease progression, but capecitabine could be an option in resource-limited settings. | The Young-PEARL study compared two treatments for premenopausal women with hormone receptor-positive, HER2-negative metastatic breast cancer who had previously used tamoxifen. Patients were given either palbociclib plus exemestane and leuprorelin (to suppress ovarian function) or capecitabine. The study found that palbociclib with endocrine therapy extended the time before the cancer progressed (19.5 months vs. 14 months) compared to capecitabine, but both groups had similar overall survival (about 55-58 months). Neutropenia was the most common severe side effect, more frequent with palbociclib. The results suggest palbociclib with endocrine therapy is better for delaying cancer progression, but capecitabine could be an alternative in resource-limited settings. |
| CancCell | 1 | 10.1016/j.ccell.2025.03.018 | Open AI | Selecting optimal candidates for next-generation anti-human epidermal growth factor receptor 2 (HER2) antibody-drug conjugates (ADCs) remains challenging. We conduct a prespecified translational study to identify treatment biomarkers in SHR-A1811-treated HER2-positive breast cancer patients from the phase 2 neoadjuvant FASCINATE-N trial using DNA and RNA sequencing, computational pathology, and single-cell in situ spatial imaging. In the hormone receptor (HR)-negative subgroup, a higher proportion and more infiltration of immune cells (i.e., tumor-infiltrating lymphocytes [TILs]), particularly cytotoxic T cells, are associated with better treatment responses. In the HR-positive subgroup, the closeness and aggregation of HER2-strong-positive tumor cells, as opposed to a uniform distribution, are linked to a lower response rate and HER2 luminal-like (HER2-LUM) subtype, which more closely resembles HR+/HER2-breast cancer. In addition, we develop a clinically practical predictive model capable of predicting neoadjuvant treatment responses to SHR-A1811 and other novel ADCs based on clinicopathological characteristics and pathological images. | Researchers studied HER2-positive breast cancer patients treated with a new drug, SHR-A1811, to find markers that predict who will respond best. They found that in hormone receptor–negative tumors, more immune cells, especially killer T cells, meant better treatment response. In hormone receptor–positive tumors, tumors with tightly clustered HER2-strong cells responded worse, especially in a subtype similar to HR+/HER2- breast cancer. The team also created a practical model using patient and tumor data to predict treatment responses to SHR-A1811 and similar drugs. | Researchers studied HER2-positive breast cancer patients treated with a new drug called SHR-A1811 to find markers that predict who will respond best. They found that in hormone receptor–negative tumors, more immune cells, especially killer T cells, were linked to better responses. In hormone receptor–positive tumors, tightly packed HER2-strong cells were linked to poorer responses. Using these findings, they created a practical model that can predict how patients might respond to SHR-A1811 and similar drugs based on their tumor features and images. | Researchers studied how to predict which HER2-positive breast cancer patients respond best to a new drug, SHR-A1811. They found that in hormone receptor–negative patients, having more immune cells, especially cytotoxic T cells, leads to better responses. In hormone receptor–positive patients, tumor cells that cluster together instead of spreading out are linked to poorer responses. Using these findings, they created a practical model that can predict how patients might respond to SHR-A1811 and similar therapies based on patient characteristics and tumor images. |
| CancCell | 1 | 10.1016/j.ccell.2025.03.019 | Claude | Selecting optimal candidates for next-generation anti-human epidermal growth factor receptor 2 (HER2) antibody-drug conjugates (ADCs) remains challenging. We conduct a prespecified translational study to identify treatment biomarkers in SHR-A1811-treated HER2-positive breast cancer patients from the phase 2 neoadjuvant FASCINATE-N trial using DNA and RNA sequencing, computational pathology, and single-cell in situ spatial imaging. In the hormone receptor (HR)-negative subgroup, a higher proportion and more infiltration of immune cells (i.e., tumor-infiltrating lymphocytes [TILs]), particularly cytotoxic T cells, are associated with better treatment responses. In the HR-positive subgroup, the closeness and aggregation of HER2-strong-positive tumor cells, as opposed to a uniform distribution, are linked to a lower response rate and HER2 luminal-like (HER2-LUM) subtype, which more closely resembles HR+/HER2-breast cancer. In addition, we develop a clinically practical predictive model capable of predicting neoadjuvant treatment responses to SHR-A1811 and other novel ADCs based on clinicopathological characteristics and pathological images. | This study looked at how to better predict which breast cancer patients will respond well to a new targeted drug called SHR-A1811. Researchers analyzed tumor samples from patients with HER2-positive breast cancer and found that the drug works differently depending on hormone receptor status. In hormone receptor-negative patients, having more immune cells attacking the tumor predicted better treatment results. In hormone receptor-positive patients, tumors where HER2-positive cancer cells were clumped together (rather than spread evenly) responded worse to treatment. The researchers also created a prediction tool using standard lab tests and tumor images that could help doctors identify which patients are most likely to benefit from this type of cancer treatment. | This study looked at ways to predict which breast cancer patients would respond best to a new targeted drug called SHR-A1811. The researchers studied patients with HER2-positive breast cancer and found that the drug works differently depending on the patient's hormone receptor status. In patients without hormone receptors, having more immune cells fighting the tumor led to better treatment results. In patients with hormone receptors, the drug worked less well when HER2-positive cancer cells were clustered together rather than spread out evenly. The team also created a practical tool that doctors could use to predict how well patients might respond to this and similar cancer drugs based on standard tests and tissue samples. | Scientists studied a new cancer drug called SHR-A1811 to find better ways to predict which breast cancer patients would respond well to treatment. They looked at tumor samples from patients with HER2-positive breast cancer and found that patients with more immune cells attacking the tumor tended to respond better to the drug, especially in certain types of breast cancer. However, in hormone-positive breast cancers, patients whose cancer cells were clustered together in clumps rather than spread out evenly were less likely to respond to treatment. The researchers also created a prediction tool that doctors could use to help decide which patients might benefit most from this type of cancer therapy. |
| CancCell | 1 | 10.1016/j.ccell.2025.03.020 | Gemini | Selecting optimal candidates for next-generation anti-human epidermal growth factor receptor 2 (HER2) antibody-drug conjugates (ADCs) remains challenging. We conduct a prespecified translational study to identify treatment biomarkers in SHR-A1811-treated HER2-positive breast cancer patients from the phase 2 neoadjuvant FASCINATE-N trial using DNA and RNA sequencing, computational pathology, and single-cell in situ spatial imaging. In the hormone receptor (HR)-negative subgroup, a higher proportion and more infiltration of immune cells (i.e., tumor-infiltrating lymphocytes [TILs]), particularly cytotoxic T cells, are associated with better treatment responses. In the HR-positive subgroup, the closeness and aggregation of HER2-strong-positive tumor cells, as opposed to a uniform distribution, are linked to a lower response rate and HER2 luminal-like (HER2-LUM) subtype, which more closely resembles HR+/HER2-breast cancer. In addition, we develop a clinically practical predictive model capable of predicting neoadjuvant treatment responses to SHR-A1811 and other novel ADCs based on clinicopathological characteristics and pathological images. | Researchers looked at how a new drug called SHR-A1811, an antibody-drug conjugate (ADC), worked in patients with HER2-positive breast cancer. They found that the drug's effectiveness depended on the characteristics of the tumor. For patients with hormone receptor (HR)-negative tumors, a strong immune response with many immune cells in the tumor was linked to better outcomes. However, in patients with HR-positive tumors, a cluster of HER2-strong-positive tumor cells was associated with a lower response to the drug. The study also developed a tool to help predict which patients will respond well to this drug and similar new treatments, using a combination of patient information and images of their tumors. | In a study of the experimental drug SHR-A1811 for HER2-positive breast cancer, researchers looked at what makes the treatment effective. They found that in patients with hormone receptor-negative cancer, a strong immune response with many immune cells, especially cytotoxic T cells, led to better results. However, in patients with hormone receptor-positive cancer, the way tumor cells were grouped together played a role; when these cells were clustered tightly, the treatment was less effective. Based on these findings, the researchers created a model that can help predict which patients are most likely to respond to this type of drug. | Researchers studied a new drug called SHR-A1811 for treating HER2-positive breast cancer. They found that the drug's effectiveness depends on the specific characteristics of the tumor. For patients with hormone receptor (HR)-negative tumors, a better response to the drug was linked to a higher presence of immune cells, especially cytotoxic T cells, within the tumor. In contrast, for patients with HR-positive tumors, a lower response was associated with HER2-positive cancer cells being clustered together rather than spread out. The study also created a model to help doctors predict which patients will respond well to this drug, using information from patient records and tumor images. |
| CancCell | 1 | 10.1016/j.ccell.2025.03.021 | DeepSeek | Selecting optimal candidates for next-generation anti-human epidermal growth factor receptor 2 (HER2) antibody-drug conjugates (ADCs) remains challenging. We conduct a prespecified translational study to identify treatment biomarkers in SHR-A1811-treated HER2-positive breast cancer patients from the phase 2 neoadjuvant FASCINATE-N trial using DNA and RNA sequencing, computational pathology, and single-cell in situ spatial imaging. In the hormone receptor (HR)-negative subgroup, a higher proportion and more infiltration of immune cells (i.e., tumor-infiltrating lymphocytes [TILs]), particularly cytotoxic T cells, are associated with better treatment responses. In the HR-positive subgroup, the closeness and aggregation of HER2-strong-positive tumor cells, as opposed to a uniform distribution, are linked to a lower response rate and HER2 luminal-like (HER2-LUM) subtype, which more closely resembles HR+/HER2-breast cancer. In addition, we develop a clinically practical predictive model capable of predicting neoadjuvant treatment responses to SHR-A1811 and other novel ADCs based on clinicopathological characteristics and pathological images. | This study explores factors that influence how well the antibody-drug conjugate (ADC) drug SHR-A1811 works in HER2-positive breast cancer patients before surgery. Researchers found that in hormone receptor (HR)-negative tumors, more immune cells (especially cytotoxic T cells) were linked to better treatment responses. In HR-positive tumors, tightly clustered HER2-positive cancer cells (rather than evenly spread) were associated with poorer outcomes and a subtype resembling HR+/HER2- breast cancer. The study also created a practical model to predict treatment success using patient data and pathology images. | This study explores factors that influence how well the antibody-drug conjugate (ADC) SHR-A1811 works in HER2-positive breast cancer patients before surgery. Researchers found that in hormone receptor (HR)-negative patients, more immune cells (especially cytotoxic T cells) in the tumor were linked to better treatment responses. In HR-positive patients, tumors with clustered HER2-strong cells (instead of evenly spread ones) responded poorly and resembled HR+/HER2- breast cancer. The study also created a practical model to predict treatment success using patient data and tumor images. | This study explored factors that influence how well the antibody-drug conjugate (ADC) drug SHR-A1811 works in HER2-positive breast cancer patients before surgery. Researchers found that in hormone receptor (HR)-negative patients, higher levels of immune cells, especially those that attack tumors, were linked to better treatment responses. In HR-positive patients, tumors with tightly clustered HER2-positive cells (rather than spread out) responded poorly and resembled less aggressive breast cancer types. The team also created a practical tool to predict treatment success using patient data and tumor images. |
| CancCell | 1 | 10.1016/j.ccell.2025.03.022 | Grok | Selecting optimal candidates for next-generation anti-human epidermal growth factor receptor 2 (HER2) antibody-drug conjugates (ADCs) remains challenging. We conduct a prespecified translational study to identify treatment biomarkers in SHR-A1811-treated HER2-positive breast cancer patients from the phase 2 neoadjuvant FASCINATE-N trial using DNA and RNA sequencing, computational pathology, and single-cell in situ spatial imaging. In the hormone receptor (HR)-negative subgroup, a higher proportion and more infiltration of immune cells (i.e., tumor-infiltrating lymphocytes [TILs]), particularly cytotoxic T cells, are associated with better treatment responses. In the HR-positive subgroup, the closeness and aggregation of HER2-strong-positive tumor cells, as opposed to a uniform distribution, are linked to a lower response rate and HER2 luminal-like (HER2-LUM) subtype, which more closely resembles HR+/HER2-breast cancer. In addition, we develop a clinically practical predictive model capable of predicting neoadjuvant treatment responses to SHR-A1811 and other novel ADCs based on clinicopathological characteristics and pathological images. | A study explored how well a new drug, SHR-A1811, works for treating HER2-positive breast cancer before surgery, focusing on identifying factors that predict its effectiveness. In patients with hormone receptor-negative cancer, more immune cells, especially cytotoxic T cells, in the tumor were linked to better treatment results. In hormone receptor-positive patients, tumors with tightly packed HER2-positive cells responded less well and resembled a less responsive cancer subtype. The study also created a simple model using clinical data and tumor images to predict how well SHR-A1811 and similar drugs might work. | In a study of the new drug SHR-A1811 for treating HER2-positive breast cancer before surgery, researchers found that patients with hormone receptor (HR)-negative tumors responded better when their tumors had more immune cells, especially cytotoxic T cells. For HR-positive patients, tumors with tightly clustered HER2-positive cells were less responsive and resembled a less aggressive breast cancer type. The study also created a simple model using clinical data and tumor images to predict how well SHR-A1811 and similar drugs might work for different patients. | This study explores how to identify which patients with HER2-positive breast cancer will respond best to a new treatment called SHR-A1811, an antibody-drug conjugate, in a clinical trial. By analyzing DNA, RNA, tissue images, and immune cell activity, researchers found that in patients without hormone receptors (HR-negative), more immune cells, especially cytotoxic T cells, in the tumor area led to better treatment results. In patients with hormone receptors (HR-positive), tumors with tightly packed HER2-positive cells responded less well and resembled a different breast cancer type. The study also created a practical model to predict how well patients will respond to SHR-A1811 and similar treatments based on clinical data and tissue images. |
| CancCell | 2 | 10.1016/j.ccell.2025.03.028 | Open AI | Circular RNA (circRNA) is a class of noncoding RNA with regulatory potentials. Its role in the transdifferentiation of prostate and lung adenocarcinoma into neuroendocrine prostate cancer (NEPC) and small cell lung cancer (SCLC) remains unexplored. Here, we identified circRMST as an exceptionally abundant circRNA predominantly expressed in NEPC and SCLC, with strong conservation between humans and mice. Functional studies using shRNA, siRNA, CRISPR-Cas13, and Cas9 consistently demonstrate that circRMST is essential for tumor growth and the expression of ASCL1, a master regulator of neuroendocrine fate. Genetic knockout of Rmst in NEPC genetic engineered mouse models prevents neuroendocrine transdifferentiation, maintaining tumors in an adenocarcinoma state. Mechanistically, circRMST physically interacts with lineage transcription factors NKX2-1 and SOX2. Loss of circRMST induces NKX2-1 protein degradation through autophagy-lysosomal pathway and alters the genomic binding of SOX2, collectively leading to the loss of ASCL1 transcription. | Researchers discovered a circular RNA called circRMST that is highly active in aggressive neuroendocrine forms of prostate and lung cancer. This RNA is crucial for tumor growth and helps control ASCL1, a key protein that drives cancer cells to become neuroendocrine. Removing circRMST in mouse models stops this transformation, keeping tumors in their original, less aggressive form. It works by interacting with other proteins (NKX2-1 and SOX2) to control gene activity, and without it, these proteins break down or lose their function, blocking the cancer’s neuroendocrine changes. | Researchers discovered that a specific circular RNA called circRMST is highly active in aggressive forms of prostate and lung cancer (NEPC and SCLC) and is crucial for these cancers to grow and change their cell type. Experiments in cells and mice show that without circRMST, tumors stay in a less dangerous adenocarcinoma state. CircRMST works by interacting with key proteins (NKX2-1 and SOX2) that control cancer cell identity, and its absence disrupts these proteins, stopping the expression of ASCL1, a major driver of neuroendocrine cancer traits. | Researchers discovered that a circular RNA called circRMST is highly present in certain aggressive cancers—neuroendocrine prostate cancer (NEPC) and small cell lung cancer (SCLC). This RNA is crucial for tumor growth and for turning regular cancer cells into neuroendocrine-type cells by helping control key proteins like ASCL1, NKX2-1, and SOX2. Removing circRMST in mice stops this transformation, keeping tumors in their original form. Essentially, circRMST acts like a switch that drives cancer cells to become more dangerous. |
| CancCell | 2 | 10.1016/j.ccell.2025.03.029 | Claude | Circular RNA (circRNA) is a class of noncoding RNA with regulatory potentials. Its role in the transdifferentiation of prostate and lung adenocarcinoma into neuroendocrine prostate cancer (NEPC) and small cell lung cancer (SCLC) remains unexplored. Here, we identified circRMST as an exceptionally abundant circRNA predominantly expressed in NEPC and SCLC, with strong conservation between humans and mice. Functional studies using shRNA, siRNA, CRISPR-Cas13, and Cas9 consistently demonstrate that circRMST is essential for tumor growth and the expression of ASCL1, a master regulator of neuroendocrine fate. Genetic knockout of Rmst in NEPC genetic engineered mouse models prevents neuroendocrine transdifferentiation, maintaining tumors in an adenocarcinoma state. Mechanistically, circRMST physically interacts with lineage transcription factors NKX2-1 and SOX2. Loss of circRMST induces NKX2-1 protein degradation through autophagy-lysosomal pathway and alters the genomic binding of SOX2, collectively leading to the loss of ASCL1 transcription. | Scientists discovered a special type of RNA called circRMST that plays a crucial role in how certain cancers change their identity. When prostate and lung cancers transform into more aggressive neuroendocrine cancers, circRMST helps control this dangerous switch by interacting with key proteins that regulate cell identity. When researchers removed circRMST from cancer cells, the tumors couldn't complete this harmful transformation and stayed as less aggressive adenocarcinomas instead. This finding suggests that circRMST could be an important target for developing new cancer treatments, since blocking it might prevent these cancers from becoming more dangerous forms. | Scientists discovered a special type of RNA molecule called circRMST that plays a crucial role in how certain prostate and lung cancers change into more aggressive forms. This RNA molecule is found in high amounts in these aggressive cancer types and is necessary for the cancers to grow and maintain their dangerous characteristics. When researchers removed or blocked circRMST in laboratory studies, the cancers lost their ability to transform into the more aggressive forms and remained as less dangerous adenocarcinomas. The circRMST works by interacting with specific proteins that control which genes are turned on or off in cancer cells, essentially acting like a master switch that helps maintain the cancer's aggressive behavior. | Circular RNA (circRNA) is a type of genetic material that helps control how cells function. Scientists discovered that a specific circRNA called circRMST plays a crucial role in how certain prostate and lung cancers change into more aggressive forms called neuroendocrine cancers. This circRNA is highly active in these aggressive cancer types and is essential for their growth. When researchers removed circRMST from cancer cells, the tumors couldn't transform into the dangerous neuroendocrine type and remained as less aggressive adenocarcinomas instead. The circRMST works by interacting with key proteins that control cell identity, and without it, these proteins break down or stop working properly, preventing the cancer transformation. |
| CancCell | 2 | 10.1016/j.ccell.2025.03.030 | Gemini | Circular RNA (circRNA) is a class of noncoding RNA with regulatory potentials. Its role in the transdifferentiation of prostate and lung adenocarcinoma into neuroendocrine prostate cancer (NEPC) and small cell lung cancer (SCLC) remains unexplored. Here, we identified circRMST as an exceptionally abundant circRNA predominantly expressed in NEPC and SCLC, with strong conservation between humans and mice. Functional studies using shRNA, siRNA, CRISPR-Cas13, and Cas9 consistently demonstrate that circRMST is essential for tumor growth and the expression of ASCL1, a master regulator of neuroendocrine fate. Genetic knockout of Rmst in NEPC genetic engineered mouse models prevents neuroendocrine transdifferentiation, maintaining tumors in an adenocarcinoma state. Mechanistically, circRMST physically interacts with lineage transcription factors NKX2-1 and SOX2. Loss of circRMST induces NKX2-1 protein degradation through autophagy-lysosomal pathway and alters the genomic binding of SOX2, collectively leading to the loss of ASCL1 transcription. | A recently discovered type of RNA, called circRMST, plays a crucial role in transforming prostate and lung cancer cells into a more aggressive, neuroendocrine form of cancer. This circRMST is highly abundant in these aggressive cancer types and is essential for their growth. It works by interacting with and protecting specific proteins, NKX2-1 and SOX2, which are vital for turning on the gene ASCL1. This gene is the master switch for this dangerous transformation. Without circRMST, the NKX2-1 protein is destroyed, and the SOX2 protein can't do its job properly, which ultimately prevents the cancer cells from becoming neuroendocrine. | A recently discovered type of RNA, called circRMST, plays a key role in transforming certain types of cancer, specifically prostate and lung adenocarcinoma, into more aggressive forms known as neuroendocrine prostate cancer (NEPC) and small cell lung cancer (SCLC). The study found that circRMST is very common in these aggressive cancers and is essential for their growth. It works by interacting with two proteins, NKX2-1 and SOX2, which are crucial for the production of another protein called ASCL1. ASCL1 is a master regulator that drives the neuroendocrine transformation. By getting rid of circRMST, researchers were able to stop this transformation, keeping the cancers in a less aggressive state. | Circular RMST is a special type of RNA that is very common in two kinds of cancer: neuroendocrine prostate cancer and small cell lung cancer. This study found that circRMST is crucial for the growth of these tumors and for activating a key gene called ASCL1, which helps these cancers change into a neuroendocrine state. It works by interacting with other proteins (NKX2-1 and SOX2). When circRMST is removed, it leads to the destruction of NKX2-1 and changes how SOX2 works, which in turn stops the ASCL1 gene from being turned on. This shows that circRMST is a key player in how these cancers develop. |
| CancCell | 2 | 10.1016/j.ccell.2025.03.031 | DeepSeek | Circular RNA (circRNA) is a class of noncoding RNA with regulatory potentials. Its role in the transdifferentiation of prostate and lung adenocarcinoma into neuroendocrine prostate cancer (NEPC) and small cell lung cancer (SCLC) remains unexplored. Here, we identified circRMST as an exceptionally abundant circRNA predominantly expressed in NEPC and SCLC, with strong conservation between humans and mice. Functional studies using shRNA, siRNA, CRISPR-Cas13, and Cas9 consistently demonstrate that circRMST is essential for tumor growth and the expression of ASCL1, a master regulator of neuroendocrine fate. Genetic knockout of Rmst in NEPC genetic engineered mouse models prevents neuroendocrine transdifferentiation, maintaining tumors in an adenocarcinoma state. Mechanistically, circRMST physically interacts with lineage transcription factors NKX2-1 and SOX2. Loss of circRMST induces NKX2-1 protein degradation through autophagy-lysosomal pathway and alters the genomic binding of SOX2, collectively leading to the loss of ASCL1 transcription. | This study found that a circular RNA called circRMST plays a key role in transforming certain prostate and lung cancers into aggressive neuroendocrine types (NEPC and SCLC). circRMST is highly active in these cancers and works with important proteins (NKX2-1 and SOX2) to control genes that drive this dangerous change. When researchers blocked circRMST, the tumors stayed in a less aggressive form, suggesting that targeting circRMST could be a new way to treat these cancers. | This study discovered a circular RNA called circRMST that plays a key role in transforming certain prostate and lung cancers into aggressive neuroendocrine types (NEPC and SCLC). circRMST is highly active in these cancers and works with specific proteins (NKX2-1 and SOX2) to control a critical gene (ASCL1) that drives neuroendocrine features. When researchers blocked circRMST, the tumors stayed in a less aggressive form. The findings suggest circRMST could be a target for future cancer treatments. | This study found that a circular RNA called circRMST plays a key role in transforming certain prostate and lung cancers into aggressive neuroendocrine types (NEPC and SCLC). circRMST is highly active in these cancers and works with important proteins (NKX2-1 and SOX2) to control genes that drive this dangerous change. When researchers blocked circRMST, the tumors stayed in a less aggressive form, suggesting circRMST is crucial for cancer progression. The findings highlight how circular RNAs can influence cancer development by interacting with critical proteins. |
| CancCell | 2 | 10.1016/j.ccell.2025.03.032 | Grok | Circular RNA (circRNA) is a class of noncoding RNA with regulatory potentials. Its role in the transdifferentiation of prostate and lung adenocarcinoma into neuroendocrine prostate cancer (NEPC) and small cell lung cancer (SCLC) remains unexplored. Here, we identified circRMST as an exceptionally abundant circRNA predominantly expressed in NEPC and SCLC, with strong conservation between humans and mice. Functional studies using shRNA, siRNA, CRISPR-Cas13, and Cas9 consistently demonstrate that circRMST is essential for tumor growth and the expression of ASCL1, a master regulator of neuroendocrine fate. Genetic knockout of Rmst in NEPC genetic engineered mouse models prevents neuroendocrine transdifferentiation, maintaining tumors in an adenocarcinoma state. Mechanistically, circRMST physically interacts with lineage transcription factors NKX2-1 and SOX2. Loss of circRMST induces NKX2-1 protein degradation through autophagy-lysosomal pathway and alters the genomic binding of SOX2, collectively leading to the loss of ASCL1 transcription. | The study identifies a circular RNA called circRMST, which is highly present in neuroendocrine prostate cancer (NEPC) and small cell lung cancer (SCLC), and is crucial for their development. circRMST works with key proteins, ASCL1, NKX2-1, and SOX2, to help cancer cells change into a neuroendocrine type. When circRMST is removed using genetic tools, it stops this transformation, keeping tumors as adenocarcinomas. Specifically, without circRMST, NKX2-1 is broken down and SOX2’s activity changes, which prevents ASCL1 production, slowing tumor growth. | The study explores a circular RNA called circRMST, which is highly present in neuroendocrine prostate cancer (NEPC) and small cell lung cancer (SCLC). Researchers found that circRMST is crucial for tumor growth and helps control a key protein, ASCL1, which drives the development of these cancers. By using genetic tools to remove circRMST in mice, the cancer cells stayed in a less aggressive state instead of turning into NEPC. The study shows that circRMST works by interacting with two proteins, NKX2-1 and SOX2, affecting their function and stability, which in turn influences ASCL1 and cancer progression. | The study explores a type of noncoding RNA called circRMST, which is highly present in aggressive forms of prostate and lung cancers that transform into neuroendocrine tumors (NEPC and SCLC). Researchers found that circRMST is crucial for tumor growth and helps control a key protein, ASCL1, that drives this transformation. By using genetic tools to remove circRMST in mouse models, the tumors stayed in a less aggressive state. CircRMST works by interacting with two proteins, NKX2-1 and SOX2, and without it, NKX2-1 is broken down and SOX2’s activity changes, stopping the production of ASCL1 and preventing the cancer from becoming more aggressive. |
| CancCell | 3 | 10.1016/j.ccell.2025.02.019 | Open AI | PDGFRA is crucial to tumorigenesis and frequently genomically altered in high-grade glioma (HGG). In a comprehensive dataset of pediatric HGG (n = 261), we detect PDGFRA mutations and/or amplifications in 15% of cases, suggesting PDGFRA as a therapeutic target. We reveal that the PDGFRA/KIT inhibitor avapritinib shows (1) selectivity for PDGFRA inhibition, (2) distinct patterns of subcellular effects, (3) in vitro and in vivo activity in patient-derived HGG models, and (4) effective blood-brain barrier penetration in mice and humans. Furthermore, we report preliminary clinical real-world experience using avapritinib in pediatric and young adult patients with predominantly recurrent/refractory PDGFRA-altered HGG (n = 8). Our early data demonstrate that avapritinib is well tolerated and results in radiographic response in 3/7 cases, suggesting a potential role for avapritinib in the treatment of HGG with specific PDGFRA alterations. Overall, these translational results underscore the therapeutic potential of PDGFRA inhibition with avapritinib in HGG. | PDGFRA, a gene often changed in high-grade gliomas (HGG), is important for tumor growth. About 15% of pediatric HGG cases have PDGFRA changes, making it a potential treatment target. The drug avapritinib, which blocks PDGFRA, works well in lab and animal models, reaches the brain effectively, and is safe in early use with children and young adults. Some patients showed tumor shrinkage, suggesting avapritinib could be a promising therapy for HGG with PDGFRA alterations. | PDGFRA, a gene often changed in high-grade gliomas (HGG), helps tumors grow. About 15% of pediatric HGG cases have PDGFRA changes, making it a possible treatment target. The drug avapritinib blocks PDGFRA, works well in lab and animal models, crosses the blood-brain barrier, and shows early positive effects in some young patients with PDGFRA-altered HGG. These findings suggest avapritinib could be a promising therapy for this type of brain cancer. | Researchers found that changes in the PDGFRA gene are common in some high-grade brain tumors in children. They tested a drug called avapritinib, which specifically targets PDGFRA, and found it can reach the brain, work in lab and animal models, and is safe in early patient use. In a small group of patients with PDGFRA-altered tumors, some showed tumor shrinkage, suggesting avapritinib could be a promising treatment for these brain cancers. |
| CancCell | 3 | 10.1016/j.ccell.2025.02.020 | Claude | PDGFRA is crucial to tumorigenesis and frequently genomically altered in high-grade glioma (HGG). In a comprehensive dataset of pediatric HGG (n = 261), we detect PDGFRA mutations and/or amplifications in 15% of cases, suggesting PDGFRA as a therapeutic target. We reveal that the PDGFRA/KIT inhibitor avapritinib shows (1) selectivity for PDGFRA inhibition, (2) distinct patterns of subcellular effects, (3) in vitro and in vivo activity in patient-derived HGG models, and (4) effective blood-brain barrier penetration in mice and humans. Furthermore, we report preliminary clinical real-world experience using avapritinib in pediatric and young adult patients with predominantly recurrent/refractory PDGFRA-altered HGG (n = 8). Our early data demonstrate that avapritinib is well tolerated and results in radiographic response in 3/7 cases, suggesting a potential role for avapritinib in the treatment of HGG with specific PDGFRA alterations. Overall, these translational results underscore the therapeutic potential of PDGFRA inhibition with avapritinib in HGG. | Scientists studied a gene called PDGFRA that plays an important role in causing brain tumors in children. They found that about 15% of children with these aggressive brain tumors have changes in this gene. The researchers tested a drug called avapritinib and discovered it can effectively block this gene, cross into the brain, and showed promising results in both lab studies and a small group of 8 young patients. Three out of seven patients who could be evaluated showed their tumors shrinking on brain scans, suggesting this drug could be a new treatment option for children with this specific type of brain tumor. | This research studied a protein called PDGFRA that helps brain tumors grow and is commonly altered in aggressive childhood brain cancers called high-grade gliomas. Scientists found that 15% of these tumors have PDGFRA changes, making it a promising treatment target. They tested a drug called avapritinib, which blocks PDGFRA, and found it works well in lab studies, reaches the brain effectively, and shows early promise in treating 8 young patients with these specific brain tumors - with 3 out of 7 patients showing tumor shrinkage and the treatment being well-tolerated. | This research studied a protein called PDGFRA that plays an important role in brain cancer development, particularly in high-grade gliomas (aggressive brain tumors) in children. The scientists found that about 15% of these brain tumors have changes in the PDGFRA gene, making it a potential target for treatment. They tested a drug called avapritinib and discovered it effectively blocks PDGFRA, crosses into the brain well, and works against these tumors in laboratory studies and animal models. When they gave avapritinib to 8 young patients with these specific brain tumors, the drug was safe and caused tumors to shrink in 3 out of 7 patients, suggesting this targeted therapy could be a promising new treatment option for children with this type of brain cancer. |
| CancCell | 3 | 10.1016/j.ccell.2025.02.021 | Gemini | PDGFRA is crucial to tumorigenesis and frequently genomically altered in high-grade glioma (HGG). In a comprehensive dataset of pediatric HGG (n = 261), we detect PDGFRA mutations and/or amplifications in 15% of cases, suggesting PDGFRA as a therapeutic target. We reveal that the PDGFRA/KIT inhibitor avapritinib shows (1) selectivity for PDGFRA inhibition, (2) distinct patterns of subcellular effects, (3) in vitro and in vivo activity in patient-derived HGG models, and (4) effective blood-brain barrier penetration in mice and humans. Furthermore, we report preliminary clinical real-world experience using avapritinib in pediatric and young adult patients with predominantly recurrent/refractory PDGFRA-altered HGG (n = 8). Our early data demonstrate that avapritinib is well tolerated and results in radiographic response in 3/7 cases, suggesting a potential role for avapritinib in the treatment of HGG with specific PDGFRA alterations. Overall, these translational results underscore the therapeutic potential of PDGFRA inhibition with avapritinib in HGG. | Researchers investigated the potential of a drug called avapritinib to treat a type of brain tumor called high-grade glioma (HGG) that has a specific genetic change, or alteration, in the PDGFRA gene. They found that this alteration is common in about 15% of pediatric HGG cases. The study showed that avapritinib effectively targets these tumors in lab and animal models, and it can successfully cross the blood-brain barrier to reach the brain. Early clinical results from a small group of patients showed that the drug was well-tolerated and led to a positive response in some cases, highlighting its potential as a new treatment for HGG with PDGFRA alterations. | Researchers studied a type of brain tumor called high-grade glioma (HGG) and found that a specific gene called PDGFRA is often altered in these tumors. This suggests that targeting this gene could be a way to treat the disease. They then tested a drug called avapritinib, which inhibits the PDGFRA gene, and found that it was effective in lab and animal models of HGG. The drug also seemed to reach the brain effectively. In a small group of patients, avapritinib was well-tolerated and showed promising results, shrinking tumors in some cases. These findings suggest that avapritinib could be a potential new treatment for HGG with specific PDGFRA alterations. | A new study shows promise for a drug called avapritinib in treating a type of aggressive brain cancer called high-grade glioma (HGG). The study focused on patients whose tumors have specific genetic changes in the PDGFRA gene. Researchers found that these changes are present in about 15% of pediatric HGG cases. The drug was shown to be effective in lab tests and animal models, and importantly, it can cross the blood-brain barrier to reach the tumor. Early clinical data from eight patients showed that avapritinib was well-tolerated and helped shrink tumors in some cases. These findings suggest that avapritinib could be a valuable new treatment option for HGG patients with these specific genetic alterations. |
| CancCell | 3 | 10.1016/j.ccell.2025.02.022 | DeepSeek | PDGFRA is crucial to tumorigenesis and frequently genomically altered in high-grade glioma (HGG). In a comprehensive dataset of pediatric HGG (n = 261), we detect PDGFRA mutations and/or amplifications in 15% of cases, suggesting PDGFRA as a therapeutic target. We reveal that the PDGFRA/KIT inhibitor avapritinib shows (1) selectivity for PDGFRA inhibition, (2) distinct patterns of subcellular effects, (3) in vitro and in vivo activity in patient-derived HGG models, and (4) effective blood-brain barrier penetration in mice and humans. Furthermore, we report preliminary clinical real-world experience using avapritinib in pediatric and young adult patients with predominantly recurrent/refractory PDGFRA-altered HGG (n = 8). Our early data demonstrate that avapritinib is well tolerated and results in radiographic response in 3/7 cases, suggesting a potential role for avapritinib in the treatment of HGG with specific PDGFRA alterations. Overall, these translational results underscore the therapeutic potential of PDGFRA inhibition with avapritinib in HGG. | This study shows that avapritinib, a drug targeting PDGFRA (a gene often mutated or amplified in aggressive brain tumors), could be a promising treatment for high-grade gliomas (HGG). Research in lab models and early results from eight young patients with recurring PDGFRA-altered HGG suggest the drug is well-tolerated, reaches the brain effectively, and may shrink tumors in some cases. These findings highlight avapritinib’s potential as a targeted therapy for certain HGG patients with PDGFRA changes. | This study shows that avapritinib, a drug that blocks PDGFRA (a protein often changed in aggressive brain tumors), could be a promising treatment for high-grade gliomas (HGG) with PDGFRA mutations or amplifications. In lab tests and early patient cases (including kids and young adults with recurring tumors), avapritinib effectively targeted the tumor, crossed into the brain, and was well tolerated, with some patients showing improvement. The findings suggest avapritinib may help treat HGG with specific PDGFRA changes. | This study shows that avapritinib, a drug targeting the PDGFRA protein, could be a promising treatment for high-grade gliomas (HGGs) with PDGFRA mutations or amplifications, which are found in about 15% of pediatric cases. Avapritinib effectively blocks PDGFRA, works in lab and animal models, and crosses the blood-brain barrier. Early results from eight young patients with recurrent or treatment-resistant HGGs show the drug is well-tolerated and helped shrink tumors in three out of seven cases. These findings suggest avapritinib may be a useful therapy for HGGs with specific PDGFRA changes. |
| CancCell | 3 | 10.1016/j.ccell.2025.02.023 | Grok | PDGFRA is crucial to tumorigenesis and frequently genomically altered in high-grade glioma (HGG). In a comprehensive dataset of pediatric HGG (n = 261), we detect PDGFRA mutations and/or amplifications in 15% of cases, suggesting PDGFRA as a therapeutic target. We reveal that the PDGFRA/KIT inhibitor avapritinib shows (1) selectivity for PDGFRA inhibition, (2) distinct patterns of subcellular effects, (3) in vitro and in vivo activity in patient-derived HGG models, and (4) effective blood-brain barrier penetration in mice and humans. Furthermore, we report preliminary clinical real-world experience using avapritinib in pediatric and young adult patients with predominantly recurrent/refractory PDGFRA-altered HGG (n = 8). Our early data demonstrate that avapritinib is well tolerated and results in radiographic response in 3/7 cases, suggesting a potential role for avapritinib in the treatment of HGG with specific PDGFRA alterations. Overall, these translational results underscore the therapeutic potential of PDGFRA inhibition with avapritinib in HGG. | Researchers found that a protein called PDGFRA, which helps tumors grow, is altered in about 15% of high-grade glioma (HGG) cases, a type of aggressive brain cancer. They tested a drug called avapritinib, which targets PDGFRA, and showed it works well in lab and animal studies, can cross into the brain, and is safe for use. In a small group of young patients with PDGFRA-altered HGG, avapritinib was well-tolerated and shrank tumors in 3 out of 7 cases, suggesting it could be a promising treatment for this type of brain cancer. | In a study of 261 pediatric high-grade glioma (HGG) cases, researchers found that 15% had changes in the PDGFRA gene, making it a potential target for treatment. They tested avapritinib, a drug that blocks PDGFRA, and found it works well in lab and animal tests, can cross the blood-brain barrier, and showed promising results in 3 out of 7 young patients with PDGFRA-altered HGG, suggesting it could be a helpful treatment option. | In a study of 261 pediatric high-grade glioma (HGG) cases, researchers found that 15% had changes in the PDGFRA gene, making it a potential target for treatment. They tested avapritinib, a drug that blocks PDGFRA and KIT proteins, and found it effectively targets PDGFRA, shows unique effects inside cells, works in lab and animal models, and can cross the blood-brain barrier in mice and humans. In a small group of 8 young patients with PDGFRA-altered HGG, avapritinib was well tolerated and showed tumor shrinkage in 3 out of 7 cases, suggesting it could be a promising treatment for HGG with PDGFRA changes. |
| CancCell | 4 | 10.1016/j.ccell.2024.11.006 | Open AI | Anti-tumor immunity is crucial for high-grade serous ovarian cancer (HGSC) prognosis, yet its adaptation upon standard chemotherapy remains poorly understood. Here, we conduct spatial and molecular characterization of 117 HGSC samples collected before and after chemotherapy. Our single-cell and spatial analyses reveal increasingly versatile immune cell states forming spatiotemporally dynamic microcommunities. We describe Myelonets, networks of interconnected myeloid cells that contribute to CD8+ T cell exhaustion post-chemotherapy and show that M1/M2 polarization at the tumor-stroma interface is associated with CD8+ T cell exhaustion and exclusion, correlating with poor chemoresponse. Single-cell and spatial transcriptomics reveal prominent myeloid-T cell interactions via NECTIN2-TIGIT induced by chemotherapy. Targeting these interactions using a functional patient-derived immuno-oncology platform demonstrates that high NECTIN2-TIGIT signaling in matched tumors predicts responses to immune checkpoint blockade. Our discovery of clinically relevant myeloid-driven spatial T cell exhaustion unlocks immunotherapeutic strategies to unleash CD8+ T cell-mediated anti-tumor immunity in HGSC. | Researchers studied how the immune system in high-grade serous ovarian cancer (HGSC) changes after chemotherapy. They found that immune cells form dynamic communities, and networks of myeloid cells—called Myelonets—can make CD8+ T cells exhausted, reducing their ability to fight the tumor. Certain patterns of immune cell interactions, especially involving NECTIN2-TIGIT, predict how well patients respond to immune therapies. These findings suggest new ways to boost anti-tumor immunity and improve treatment outcomes in HGSC. | Researchers studied high-grade serous ovarian cancer (HGSC) before and after chemotherapy and found that immune cells in tumors change in complex ways. Networks of myeloid cells, called Myelonets, contribute to the weakening of CD8+ T cells, especially near the tumor-stroma interface, which is linked to poor chemotherapy response. They discovered that chemotherapy increases interactions between myeloid cells and T cells through NECTIN2-TIGIT, and targeting this pathway could improve immune therapy effectiveness, offering new ways to boost the body’s anti-cancer immune response. | Researchers studied high-grade serous ovarian cancer (HGSC) before and after chemotherapy to understand how the immune system reacts. They found that networks of myeloid cells, called Myelonets, cause exhaustion of CD8+ T cells, especially at the tumor-stroma boundary, which is linked to poor response to chemotherapy. Chemotherapy also increases specific myeloid-T cell interactions (NECTIN2-TIGIT), and blocking these signals can improve immune responses. This work shows that targeting these myeloid-driven interactions could help boost the body’s ability to fight HGSC. |
| CancCell | 4 | 10.1016/j.ccell.2024.11.007 | Claude | Anti-tumor immunity is crucial for high-grade serous ovarian cancer (HGSC) prognosis, yet its adaptation upon standard chemotherapy remains poorly understood. Here, we conduct spatial and molecular characterization of 117 HGSC samples collected before and after chemotherapy. Our single-cell and spatial analyses reveal increasingly versatile immune cell states forming spatiotemporally dynamic microcommunities. We describe Myelonets, networks of interconnected myeloid cells that contribute to CD8+ T cell exhaustion post-chemotherapy and show that M1/M2 polarization at the tumor-stroma interface is associated with CD8+ T cell exhaustion and exclusion, correlating with poor chemoresponse. Single-cell and spatial transcriptomics reveal prominent myeloid-T cell interactions via NECTIN2-TIGIT induced by chemotherapy. Targeting these interactions using a functional patient-derived immuno-oncology platform demonstrates that high NECTIN2-TIGIT signaling in matched tumors predicts responses to immune checkpoint blockade. Our discovery of clinically relevant myeloid-driven spatial T cell exhaustion unlocks immunotherapeutic strategies to unleash CD8+ T cell-mediated anti-tumor immunity in HGSC. | This study looked at how the immune system in ovarian cancer patients changes after chemotherapy treatment. Researchers found that chemotherapy causes immune cells called myeloid cells to form networks that weaken the cancer-fighting T cells, making them less effective at attacking tumors. They discovered that certain protein interactions (NECTIN2-TIGIT) increase after chemotherapy and contribute to this immune suppression. However, the study also found that measuring these protein levels could help predict which patients might respond well to immunotherapy treatments that help reactivate the immune system to fight cancer more effectively. | Scientists studied how the immune system responds to ovarian cancer before and after chemotherapy treatment. They found that chemotherapy causes immune cells called myeloid cells to form networks that make cancer-fighting T cells become exhausted and less effective. The researchers discovered that when these myeloid cells interact with T cells through specific proteins (NECTIN2-TIGIT), it weakens the immune response against the tumor. However, this same interaction can help predict which patients might benefit from immunotherapy treatments that boost the immune system's ability to fight cancer. | This study looked at how the immune system in ovarian cancer patients changes after chemotherapy treatment. Researchers found that chemotherapy causes immune cells to form complex networks that actually make it harder for the body's cancer-fighting T cells to work properly. They discovered that certain immune cells create connections that exhaust and block the T cells from attacking tumors effectively. However, they also identified specific protein interactions that could be targeted with new immunotherapy treatments to help restore the immune system's ability to fight ovarian cancer. This research could lead to better combination treatments that use both chemotherapy and immunotherapy together. |
| CancCell | 4 | 10.1016/j.ccell.2024.11.008 | Gemini | Anti-tumor immunity is crucial for high-grade serous ovarian cancer (HGSC) prognosis, yet its adaptation upon standard chemotherapy remains poorly understood. Here, we conduct spatial and molecular characterization of 117 HGSC samples collected before and after chemotherapy. Our single-cell and spatial analyses reveal increasingly versatile immune cell states forming spatiotemporally dynamic microcommunities. We describe Myelonets, networks of interconnected myeloid cells that contribute to CD8+ T cell exhaustion post-chemotherapy and show that M1/M2 polarization at the tumor-stroma interface is associated with CD8+ T cell exhaustion and exclusion, correlating with poor chemoresponse. Single-cell and spatial transcriptomics reveal prominent myeloid-T cell interactions via NECTIN2-TIGIT induced by chemotherapy. Targeting these interactions using a functional patient-derived immuno-oncology platform demonstrates that high NECTIN2-TIGIT signaling in matched tumors predicts responses to immune checkpoint blockade. Our discovery of clinically relevant myeloid-driven spatial T cell exhaustion unlocks immunotherapeutic strategies to unleash CD8+ T cell-mediated anti-tumor immunity in HGSC. | Chemotherapy for a type of ovarian cancer called high-grade serous ovarian cancer (HGSC) can have a negative effect on the immune system's ability to fight the tumor. This study found that after chemotherapy, certain immune cells called myeloid cells create specific networks that cause T cells (a type of white blood cell that fights cancer) to become exhausted. This exhaustion, which happens in particular areas of the tumor, is linked to a poor response to chemotherapy. The researchers discovered a specific interaction, or signal, between these myeloid and T cells that chemotherapy triggers. They suggest that blocking this signal could be a new way to help T cells fight the cancer, potentially improving treatment for patients with HGSC. | Chemotherapy for ovarian cancer changes the tumor's environment, causing certain immune cells (myeloid cells) to become organized into networks called "Myelonets." These networks cause the cancer-fighting T cells to become exhausted and less effective. The researchers found that a specific interaction between myeloid and T cells, called NECTIN2-TIGIT signaling, is a key part of this process. They also discovered that high levels of this signaling predict which patients might respond well to a type of treatment called immune checkpoint blockade, which could help re-activate the exhausted T cells and improve anti-tumor immunity. | Chemotherapy for ovarian cancer changes the environment around tumors, causing certain immune cells called **myeloid cells** to make cancer-fighting **T cells** tired and ineffective. This process, which is linked to a specific cell signal called NECTIN2-TIGIT, helps explain why some patients don't respond well to chemotherapy. The discovery suggests that targeting this signal could lead to new treatments that help the body's T cells fight the cancer more effectively |
| CancCell | 4 | 10.1016/j.ccell.2024.11.009 | DeepSeek | Anti-tumor immunity is crucial for high-grade serous ovarian cancer (HGSC) prognosis, yet its adaptation upon standard chemotherapy remains poorly understood. Here, we conduct spatial and molecular characterization of 117 HGSC samples collected before and after chemotherapy. Our single-cell and spatial analyses reveal increasingly versatile immune cell states forming spatiotemporally dynamic microcommunities. We describe Myelonets, networks of interconnected myeloid cells that contribute to CD8+ T cell exhaustion post-chemotherapy and show that M1/M2 polarization at the tumor-stroma interface is associated with CD8+ T cell exhaustion and exclusion, correlating with poor chemoresponse. Single-cell and spatial transcriptomics reveal prominent myeloid-T cell interactions via NECTIN2-TIGIT induced by chemotherapy. Targeting these interactions using a functional patient-derived immuno-oncology platform demonstrates that high NECTIN2-TIGIT signaling in matched tumors predicts responses to immune checkpoint blockade. Our discovery of clinically relevant myeloid-driven spatial T cell exhaustion unlocks immunotherapeutic strategies to unleash CD8+ T cell-mediated anti-tumor immunity in HGSC. | This study explores how chemotherapy affects the immune system in ovarian cancer. Researchers analyzed tumor samples before and after treatment and found that chemotherapy triggers a network of myeloid cells (called Myelonets) that cause CD8+ T cells—key fighters against cancer—to become exhausted and less effective. These changes, especially near the tumor-stroma border, are linked to poor treatment response. The study also identified a specific interaction (NECTIN2-TIGIT) between myeloid and T cells that worsens exhaustion. Blocking this interaction could help restore T cell function and improve immunotherapy outcomes in ovarian cancer. | This study explores how chemotherapy affects the immune system in ovarian cancer. Researchers analyzed tumor samples before and after treatment and found that chemotherapy triggers a network of myeloid cells (called Myelonets) that cause CD8+ T cells (key immune fighters) to become exhausted, especially near the tumor-stroma border. They identified a specific interaction (NECTIN2-TIGIT) between myeloid and T cells that worsens exhaustion. Blocking this interaction could help restore T cell function and improve responses to immunotherapy. The findings suggest new ways to enhance treatment by targeting these immune-suppressing mechanisms. | This study explores how chemotherapy affects the immune system in ovarian cancer patients. Researchers analyzed tumor samples before and after treatment and found that chemotherapy triggers a network of myeloid cells (called Myelonets) that cause CD8+ T cells—key fighters against cancer—to become exhausted and less effective. These changes, along with certain immune signals (like NECTIN2-TIGIT), lead to poor treatment responses. However, blocking these signals could help restore T cell function and improve immunotherapy outcomes. The findings highlight new ways to boost the immune system’s ability to fight ovarian cancer after chemotherapy. |
| CancCell | 4 | 10.1016/j.ccell.2024.11.010 | Grok | Anti-tumor immunity is crucial for high-grade serous ovarian cancer (HGSC) prognosis, yet its adaptation upon standard chemotherapy remains poorly understood. Here, we conduct spatial and molecular characterization of 117 HGSC samples collected before and after chemotherapy. Our single-cell and spatial analyses reveal increasingly versatile immune cell states forming spatiotemporally dynamic microcommunities. We describe Myelonets, networks of interconnected myeloid cells that contribute to CD8+ T cell exhaustion post-chemotherapy and show that M1/M2 polarization at the tumor-stroma interface is associated with CD8+ T cell exhaustion and exclusion, correlating with poor chemoresponse. Single-cell and spatial transcriptomics reveal prominent myeloid-T cell interactions via NECTIN2-TIGIT induced by chemotherapy. Targeting these interactions using a functional patient-derived immuno-oncology platform demonstrates that high NECTIN2-TIGIT signaling in matched tumors predicts responses to immune checkpoint blockade. Our discovery of clinically relevant myeloid-driven spatial T cell exhaustion unlocks immunotherapeutic strategies to unleash CD8+ T cell-mediated anti-tumor immunity in HGSC. | In a study of 117 high-grade serous ovarian cancer samples, researchers found that chemotherapy changes the immune system’s behavior in the tumor area, making immune cells like T cells less effective. They identified networks of myeloid cells, called Myelonets, that interact with T cells and cause them to become exhausted after chemotherapy, especially at the tumor’s edge, which is linked to poor treatment response. Using advanced analysis and a patient-derived testing platform, they discovered that a specific interaction between molecules (NECTIN2-TIGIT) increases after chemotherapy and can predict how well immunotherapy might work. This finding opens new possibilities for improving immune-based treatments for ovarian cancer. | This study explores how chemotherapy affects the immune system in high-grade serous ovarian cancer (HGSC) by analyzing 117 tumor samples before and after treatment. Using advanced single-cell and spatial analysis, researchers found that chemotherapy increases interactions between immune cells, particularly myeloid cells, which form networks called Myelonets that exhaust CD8+ T cells, weakening the body’s ability to fight the cancer. They identified a specific interaction between NECTIN2 and TIGIT proteins that drives this exhaustion, especially at the tumor-stroma interface, and is linked to poor chemotherapy response. By targeting this interaction using a patient-derived testing platform, the study shows that high NECTIN2-TIGIT signaling predicts how well tumors respond to immunotherapy, offering new ways to improve immune-based treatments for HGSC. | This study explores how chemotherapy affects the immune system in high-grade serous ovarian cancer (HGSC) by analyzing 117 tumor samples before and after treatment. Using advanced single-cell and spatial analysis, researchers found that chemotherapy leads to dynamic immune cell interactions, particularly involving myeloid cells that form networks called Myelonets, which cause T cell exhaustion and reduce their ability to fight cancer. They also identified that specific myeloid-T cell interactions (via NECTIN2-TIGIT) increase after chemotherapy and are linked to poor treatment response. By targeting these interactions, the study suggests new immunotherapy strategies to boost T cell activity and improve outcomes in HGSC. |
| CancCell | 5 | 10.1016/j.ccell.2025.03.007 | Open AI | Immune checkpoint inhibitors (ICIs) have improved outcomes of patients with many different cancers. These antibodies target molecules such as programmed cell death 1 (PD-1) or cytotoxic T lymphocyte associated protein 4 (CTLA-4) which normally function to limit immune activity. Treatment with ICIs reactivates T cells to destroy tumor cells in a highly specific manner, which in some patients, results in dramatic remissions and durable disease control. Over the last decade, much effort has been directed at characterizing factors that drive efficacy and resistance to ICI therapy. Food and Drug Administration (FDA)-approved biomarkers for ICI therapy have facilitated more judicious treatment of cancer patients and transformed the field of precision oncology. Yet, adaptive immunity against cancers is complex, and newer data have revealed the potential utility of other biomarkers. In this review, we discuss the utility of currently approved biomarkers and highlight how emerging biomarkers can further improve the identification of patients who benefit from ICIs. | Immune checkpoint inhibitors (ICIs) are cancer treatments that reactivate the immune system to attack tumors by targeting molecules like PD-1 and CTLA-4. They can lead to strong, lasting remissions in some patients. Researchers have studied why these treatments work or fail, and FDA-approved biomarkers help guide who should receive them. Newer research is exploring additional biomarkers to better identify patients who are likely to benefit from ICIs. | Immune checkpoint inhibitors (ICIs) are cancer treatments that help the immune system’s T cells attack tumors by blocking molecules that normally slow immune activity. They can lead to strong, lasting remissions in some patients. Scientists are studying why ICIs work well for some people and not others, using biomarkers—biological signs that guide treatment. FDA-approved biomarkers already help doctors choose the right patients for ICIs, and new biomarkers may further improve patient selection and outcomes. | Immune checkpoint inhibitors (ICIs) are cancer treatments that help the immune system’s T cells attack tumors by blocking molecules like PD-1 and CTLA-4 that usually slow immune activity. These drugs can lead to strong, long-lasting remissions in some patients. Scientists have been studying why ICIs work for some people and not others, and FDA-approved biomarkers now help doctors choose the right patients for treatment. New research suggests additional biomarkers could further improve how patients who will benefit from ICIs are identified. |
| CancCell | 5 | 10.1016/j.ccell.2025.03.008 | Claude | Immune checkpoint inhibitors (ICIs) have improved outcomes of patients with many different cancers. These antibodies target molecules such as programmed cell death 1 (PD-1) or cytotoxic T lymphocyte associated protein 4 (CTLA-4) which normally function to limit immune activity. Treatment with ICIs reactivates T cells to destroy tumor cells in a highly specific manner, which in some patients, results in dramatic remissions and durable disease control. Over the last decade, much effort has been directed at characterizing factors that drive efficacy and resistance to ICI therapy. Food and Drug Administration (FDA)-approved biomarkers for ICI therapy have facilitated more judicious treatment of cancer patients and transformed the field of precision oncology. Yet, adaptive immunity against cancers is complex, and newer data have revealed the potential utility of other biomarkers. In this review, we discuss the utility of currently approved biomarkers and highlight how emerging biomarkers can further improve the identification of patients who benefit from ICIs. | Immune checkpoint inhibitors are cancer drugs that work by removing the "brakes" on the body's immune system, allowing it to better recognize and attack cancer cells. These medications have helped many cancer patients achieve long-lasting remissions by targeting specific proteins that normally prevent immune cells from being too active. Doctors now use special tests called biomarkers to identify which patients are most likely to benefit from these treatments, making cancer care more personalized and effective, though researchers continue working to develop even better ways to predict treatment success. | Immune checkpoint inhibitors are a type of cancer treatment that work by removing the brakes on the body's immune system, allowing it to better fight cancer cells. These drugs target specific proteins that normally prevent immune cells from attacking tumors. When successful, this treatment can lead to significant tumor shrinkage and long-lasting cancer control in some patients. Scientists have been working to identify which patients are most likely to benefit from these treatments by studying various biological markers, and while some approved tests already help doctors make better treatment decisions, researchers continue to discover new ways to predict which patients will respond best to these immunotherapies. | Immune checkpoint inhibitors (ICIs) are a type of cancer treatment that work by removing the "brakes" on the body's immune system, allowing it to better recognize and attack cancer cells. These drugs have helped many cancer patients achieve long-lasting remissions by reactivating immune cells called T cells to specifically target tumors. Scientists have been working to identify biomarkers - biological indicators that can predict which patients are most likely to respond to this treatment. While some FDA-approved biomarkers already help doctors make better treatment decisions, researchers continue to discover new markers that could further improve the ability to identify patients who will benefit most from immune checkpoint inhibitor therapy. |
| CancCell | 5 | 10.1016/j.ccell.2025.03.009 | Gemini | Immune checkpoint inhibitors (ICIs) have improved outcomes of patients with many different cancers. These antibodies target molecules such as programmed cell death 1 (PD-1) or cytotoxic T lymphocyte associated protein 4 (CTLA-4) which normally function to limit immune activity. Treatment with ICIs reactivates T cells to destroy tumor cells in a highly specific manner, which in some patients, results in dramatic remissions and durable disease control. Over the last decade, much effort has been directed at characterizing factors that drive efficacy and resistance to ICI therapy. Food and Drug Administration (FDA)-approved biomarkers for ICI therapy have facilitated more judicious treatment of cancer patients and transformed the field of precision oncology. Yet, adaptive immunity against cancers is complex, and newer data have revealed the potential utility of other biomarkers. In this review, we discuss the utility of currently approved biomarkers and highlight how emerging biomarkers can further improve the identification of patients who benefit from ICIs. | Immune checkpoint inhibitors (ICIs) are a type of cancer treatment that helps the body's immune system fight off cancer cells. These treatments have shown great promise in improving patient outcomes, but they don't work for everyone. To help doctors decide which patients will benefit most, scientists have identified certain "biomarkers" that can predict a patient's response to ICI therapy. This research review discusses both the established biomarkers that are already used by doctors and new, emerging biomarkers that could further improve how we select patients for this treatment. | Immune checkpoint inhibitors (ICIs) are a type of cancer treatment that helps the body's own immune system fight off cancer. While these treatments have been very successful for many patients, they don't work for everyone. Scientists are using biomarkers, which are biological signs, to predict who will respond best to these therapies. The FDA has already approved some biomarkers to help doctors decide on treatment. However, new research is constantly uncovering additional, emerging biomarkers that could make it even easier to identify patients who will benefit most from these life-saving drugs. | Immune checkpoint inhibitors (ICIs) are a type of cancer treatment that helps the body's own immune system fight tumors. While they have been very successful for many patients, it's not clear who will respond best to them. Scientists have identified certain biomarkers, or biological indicators, that can help predict who will benefit from these treatments. Some of these biomarkers are already approved for clinical use, but researchers are constantly discovering new ones. By using these established and emerging biomarkers, doctors hope to get even better at identifying the patients who are most likely to respond to ICI therapy. |
| CancCell | 5 | 10.1016/j.ccell.2025.03.010 | DeepSeek | Immune checkpoint inhibitors (ICIs) have improved outcomes of patients with many different cancers. These antibodies target molecules such as programmed cell death 1 (PD-1) or cytotoxic T lymphocyte associated protein 4 (CTLA-4) which normally function to limit immune activity. Treatment with ICIs reactivates T cells to destroy tumor cells in a highly specific manner, which in some patients, results in dramatic remissions and durable disease control. Over the last decade, much effort has been directed at characterizing factors that drive efficacy and resistance to ICI therapy. Food and Drug Administration (FDA)-approved biomarkers for ICI therapy have facilitated more judicious treatment of cancer patients and transformed the field of precision oncology. Yet, adaptive immunity against cancers is complex, and newer data have revealed the potential utility of other biomarkers. In this review, we discuss the utility of currently approved biomarkers and highlight how emerging biomarkers can further improve the identification of patients who benefit from ICIs. | Immune checkpoint inhibitors (ICIs) are cancer treatments that boost the immune system to attack tumors by targeting proteins like PD-1 or CTLA-4. While some patients see long-lasting benefits, not everyone responds, so researchers have been studying biomarkers to predict who will benefit. Currently, FDA-approved biomarkers help guide treatment decisions, but new discoveries suggest additional markers could improve patient selection. This review explores both established and emerging biomarkers to better match ICI therapy to the right patients. | Immune checkpoint inhibitors (ICIs) are cancer treatments that boost the immune system to attack tumors by targeting proteins like PD-1 or CTLA-4. While some patients see long-lasting benefits, not everyone responds, so researchers have developed biomarkers to predict who will benefit most. Currently approved biomarkers help guide treatment decisions, but newer studies suggest additional markers could improve patient selection. This review explores both established and emerging biomarkers to better personalize ICI therapy. | Immune checkpoint inhibitors (ICIs) are cancer treatments that boost the immune system to attack tumors by targeting proteins like PD-1 or CTLA-4. While some patients see long-lasting results, not everyone responds to these therapies. Currently, FDA-approved biomarkers help doctors decide which patients might benefit, but research is uncovering new biomarkers to better predict treatment success. This review explores both established and emerging biomarkers to improve personalized cancer care. |
| CancCell | 5 | 10.1016/j.ccell.2025.03.011 | Grok | Immune checkpoint inhibitors (ICIs) have improved outcomes of patients with many different cancers. These antibodies target molecules such as programmed cell death 1 (PD-1) or cytotoxic T lymphocyte associated protein 4 (CTLA-4) which normally function to limit immune activity. Treatment with ICIs reactivates T cells to destroy tumor cells in a highly specific manner, which in some patients, results in dramatic remissions and durable disease control. Over the last decade, much effort has been directed at characterizing factors that drive efficacy and resistance to ICI therapy. Food and Drug Administration (FDA)-approved biomarkers for ICI therapy have facilitated more judicious treatment of cancer patients and transformed the field of precision oncology. Yet, adaptive immunity against cancers is complex, and newer data have revealed the potential utility of other biomarkers. In this review, we discuss the utility of currently approved biomarkers and highlight how emerging biomarkers can further improve the identification of patients who benefit from ICIs. | Immune checkpoint inhibitors (ICIs) are drugs that help the immune system fight cancer by targeting molecules like PD-1 and CTLA-4, which normally calm down immune activity. By blocking these molecules, ICIs activate T cells to attack tumor cells, leading to significant tumor shrinkage and long-term cancer control in some patients. Over the past ten years, researchers have worked to identify factors that make ICIs effective or cause resistance. FDA-approved biomarkers have improved cancer treatment by helping doctors choose the right patients for ICIs, and new biomarkers are being explored to further enhance patient selection and treatment success. | Immune checkpoint inhibitors (ICIs) are drugs that help the immune system fight cancer by targeting molecules like PD-1 or CTLA-4, which normally dampen immune activity. These treatments can lead to significant cancer remission in some patients by reactivating T cells to attack tumors. While FDA-approved biomarkers have improved patient selection for ICI therapy, new research is exploring additional biomarkers to better identify who will benefit from these treatments, enhancing precision in cancer care. | Immune checkpoint inhibitors (ICIs) are drugs that help the immune system fight cancer by targeting molecules like PD-1 or CTLA-4, which normally calm immune activity. By reactivating T cells, ICIs allow the body to attack tumor cells effectively, leading to significant and lasting cancer control in some patients. Over the past ten years, researchers have studied what makes ICIs work or fail, leading to FDA-approved biomarkers that guide better treatment choices in precision oncology. This review discusses these approved biomarkers and explores new ones that could help identify which patients will benefit most from ICIs. |

**Table 4s: GAI generated Lay abstracts and summaries (Test 1-3), using Task specific LLM-powered tool (Pub2Post)**

| **Journal** | **Abstract** | **DOI** | **Arm** | **Original Abstract** | **Test 1** | **Test 2** | **Test 3** |
| --- | --- | --- | --- | --- | --- | --- | --- |
| JCO | 1 | 10.1200/JCO-24-02007 | Pub2Post | PURPOSETo our knowledge, the ACHILLES/TORG1834 trial is the first randomized study comparing afatinib and chemotherapy in patients with non-small cell lung cancer (NSCLC) harboring sensitizing uncommon epidermal growth factor receptor (EGFR) mutations.METHODSThis randomized, open-label study was performed at 51 Japanese institutions and recruited treatment-na & iuml;ve patients with nonsquamous NSCLC with uncommon EGFR mutations, excluding exon 20 insertions and T790M mutations. Patients were randomly assigned 2:1 to receive afatinib (30 or 40 mg orally, at the treating physician's discretion) or a combination of platinum (cisplatin or carboplatin) and pemetrexed, followed by pemetrexed maintenance. The primary end point was progression-free survival (PFS). Secondary end points included objective response rate (ORR), overall survival, and safety. A prespecified interim analysis was planned to provide clinically meaningful information promptly, along with a crossover recommendation if necessary.RESULTSA total of 109 patients were enrolled between March 2019 and February 2023. In the interim analysis, the Data and Safety Monitoring Committee recommended early study termination. The median PFS was significantly longer in patients receiving afatinib than in those undergoing chemotherapy (10.6 v 5.7 months; hazard ratio, 0.421 [95% CI, 0.251 to 0.706]; P = .0010). ORRs to afatinib were similar across the overall population and among participants with major uncommon (G719X, L861Q, and S768I), compound, and other mutations (61.7%, 55.8%, 72.7%, and 60.0%, respectively). The most common grade 3 or higher adverse events were diarrhea, paronychia, and rash for afatinib, and appetite loss and nausea for chemotherapy.CONCLUSIONAfatinib should be considered the standard initial therapy for patients with NSCLC with sensitizing uncommon EGFR mutations. | Background/Introduction:  This study looked at how well two kinds of medicine work for a type of lung cancer that has rare gene changes. One kind is called afatinib, a pill that blocks a signal helping cancer grow. The other is a mix of strong drugs called chemo. People in the study had a type of lung cancer called non-small cell lung cancer. They had not taken any treatment before. All of them had rare forms of a gene called epidermal growth factor receptor, which helps cancer grow.  Methods:  The study took place in many hospitals in Japan. People were split into 2 groups. One big group got afatinib pills. The smaller group got chemo, which used drugs given by a doctor. These people had to keep taking their given treatment until their cancer got worse. The main thing checked was how long the treatment stopped the cancer from getting worse. The study also looked at how many people had their cancer shrink and how safe each treatment was.   Findings/Results:  A little over one hundred people joined the study. After some time, the people checking the study said the group with afatinib was doing much better. So, they stopped the study early to protect others. On average, people who took afatinib had their cancer stay the same for about twice as long as those who got chemo. Afatinib worked well even for people who had different rare gene forms. Some people got side effects like loose poop, nail pain near fingers, or red skin. People who got chemo felt sick and had less wish to eat.  Conclusion:  Afatinib worked better than chemo in these people. It lasted longer. It also helped more people. Afatinib should be the first choice for people with this kind of lung cancer and these rare gene forms.  Short Summary:  This study looked at people with a kind of lung cancer that had rare gene changes. The goal was to see which treatment worked better and was safer. One group took afatinib pills; the other got chemo with strong drugs. People who took the pill had their cancer stay small longer and felt better than those who got chemo. The pill caused fewer hard side effects. Because of this, doctors now say the pill should be the first choice for treatment. More studies may not be needed now since the results were very clear. | Background/Introduction  This study looked at people with a kind of lung cancer called non-small cell lung cancer. These people had rare changes, called mutations, in a part of their cells known as the epidermal growth factor receptor. These changes can help the cancer grow. The goal was to see which treatment worked better—afatinib (a drug taken by mouth) or regular chemotherapy (a mix of other drugs). This study was the first of its kind to look at these two choices in this group of people.  Methods  People from many hospitals in Japan took part in this study. They had not been treated for their lung cancer before. Each person had the special gene change, but they did not have two other common changes linked to poor response to treatment. They were split into two groups. One group got afatinib, and the other got the usual chemotherapy mix. Doctors watched how long the cancer stayed the same, how well the treatment worked, and what side effects happened. The study was planned to stop early if strong results were found.  Findings/Results  A little over a hundred people joined the study. It lasted for a few years. Later, doctors saw that people who took afatinib stayed free of cancer growth for more time than those on chemotherapy. Also, afatinib seemed to work well across all the different rare gene changes. People on afatinib mostly had tummy problems and rashes. People on chemo mostly felt sick and did not want to eat. Because afatinib worked better, the study was ended early.  Conclusion  Afatinib helped people the most in this group. Based on what was seen, afatinib should be the first choice for people with this kind of lung cancer. Future studies may look more at longer survival and use in other groups.  Short Summary  This study tested afatinib, a drug for lung cancer, in people who had rare gene changes. These people had not been treated before. Afatinib worked better than usual chemotherapy and helped people stay well longer. People had some side effects, but these were okay to manage. Because afatinib worked so well, the study was stopped early. The study shows afatinib is the best first choice for these patients, and more studies may happen in the future. | Background/Introduction:  Some people with lung cancer have a change in their body called a mutation. One kind of lung cancer is called non-small cell lung cancer. Some mutations in this cancer are not common. Doctors use different drugs to help treat lung cancer. This study looked to see if one drug called afatinib works better than old-style chemo for people with these rare changes.  Methods:  The study was done in Japan. Adults with rare lung cancer changes took part. The people had not had any treatment before. Half of them took afatinib as a pill. The others got chemo drugs through a needle. They were put into the groups by chance to keep it fair. The study wanted to see how long the cancer stayed the same or got worse, how many people got better, and if the drugs were safe to use.  Findings/Results:  In a few years, over one hundred people joined. A safety team looked at the first set of results and found that afatinib worked a lot better. People who took afatinib had their cancer stay the same or grow much slower than those who got chemo. Chemo worked, too, but not as well. Afatinib helped people with different rare changes, not just one kind. The bad effects with afatinib were things like tummy runs, skin problems, and nail pain. Chemo caused sick feelings and made people not feel like eating.  Conclusion:  People with this kind of lung cancer do better if they take afatinib first instead of chemo. This drug should now be the first choice for doctors to use.   Short Summary:  This study looked at people in Japan with a rare type of lung cancer change. The goal was to see if an oral drug called afatinib helped more than older chemo treatments. Afatinib helped more people, kept cancer from growing longer, and worked well for several types of rare changes. Chemo worked too, but not as well. The side effects from afatinib were mostly stomach and skin related. The study found afatinib should be the first drug used for these patients. Based on the good results, follow-up studies may not be needed for the same question. |
| JCO | 2 | 10.1200/JCO-24-01269 | Pub2Post | PURPOSE Mobocertinib is an oral epidermal growth factor receptor (EGFR) tyrosine kinase inhibitor that targets EGFR exon 20 insertion (ex20ins) mutations in non-small cell lung cancer (NSCLC). This open-label, phase III trial (EXCLAIM-2, ClinicalTrials.gov identifier: NCT04129502) compared mobocertinib versus platinum-based chemotherapy as first-line treatment of EGFR ex20ins+ advanced/metastatic NSCLC. METHODS Patients with treatment-naive EGFR ex20ins+ locally advanced/metastatic NSCLC were randomly assigned 1:1 to mobocertinib 160 mg once daily or pemetrexed plus cisplatin or carboplatin every 3 weeks for four cycles followed by maintenance pemetrexed. The primary end point was progression-free survival (PFS) by blinded independent central review (BICR), with planned interim analysis (IA) after approximately 70% of 227 expected PFS events. RESULTS A total of 354 patients were randomly assigned (mobocertinib: n = 179; chemotherapy: n = 175). Baseline characteristics were balanced between arms. At IA (cutoff: April 4, 2023), the median PFS per BICR was 9.6 months in each treatment arm (hazard ratio [HR], 1.04 [95% CI, 0.77 to 1.39]; P = .803). The primary end point crossed the prespecified futility boundary (HR > 1). The confirmed objective response rate (95% CI) per BICR was 32% (26 to 40) with mobocertinib versus 30% (24 to 38) with chemotherapy; the median duration of response was 12.0 versus 8.4 months. Quality-of-life assessments indicated clinically meaningful delays in time to deterioration of lung cancer symptoms, cognitive function, and constipation with mobocertinib versus chemotherapy. Grade >= 3 adverse events in >5% of patients (mobocertinib, chemotherapy) were diarrhea (20%, 1%), anemia (6%, 10%), increased lipase (6%, 0%), and decreased neutrophil count (1%, 7%). CONCLUSION The EXCLAIM-2 trial did not meet its primary end point. The efficacy of mobocertinib was not superior to platinum-based chemotherapy for first-line treatment of patients with EGFR ex20ins+ advanced/metastatic NSCLC. | Background/Introduction:  This study looked at people with a type of lung cancer called non-small cell lung cancer. These people had a rare change in a gene known as EGFR exon twenty insertion. This gene change helps the cancer grow. One group got a drug called mobocertinib, taken by mouth each day. The other group got strong medicines through a drip, called platinum chemo. The goal was to see if mobocertinib worked better than chemo as the first treatment.  Methods:  People in the study had not been treated before and had cancer that had spread. Half took mobocertinib pills every day. The others got chemo every few weeks. Some of those stayed on one of the chemo drugs afterwards. Doctors checked how long it took before the cancer got worse. They also looked at how people's bodies reacted to the drugs and how the people felt during treatment.  Findings/Results:  The number of people was about the same in both groups. On average, the cancer took the same amount of time to get worse in both groups. The chance of the tumors shrinking was about the same too. People on mobocertinib felt better for longer with fewer lung problems and trouble going to the bathroom. But mobocertinib caused more cases of very bad loose stools. Chemo led to more people getting tired and having low blood counts.  Conclusion:  The pill mobocertinib did not work better than chemo for people with this lung cancer type. Even though some people felt better on mobocertinib, the cancer did not stay away longer. More research may be needed to find better first treatments for these patients.  Short Summary:  This study compared a new lung cancer pill, mobocertinib, to common chemo in people with a rare gene change. Both treatment groups had the same time before the cancer worsened. People taking the pill felt better in some ways but also had more bad stomach issues. In the end, the pill did not stop the cancer better than chemo. New studies may help find better options. | Background/Introduction:  Lung cancer is a disease where bad cells grow in the lungs. Some people have a special kind called non-small cell lung cancer. These people might carry a change in a tiny part of their body’s instructions, called EGFR exon twenty insertion. That change can make the cancer grow fast. Doctors wanted to see if a new pill drug called mobocertinib works better than old-style chemo drugs to help these people live longer without the cancer getting worse. The old-style chemo drugs often use strong medicines given with a needle.  Methods:  The study included people with this special kind of lung cancer. They had not started any treatment before. Half of the group got the new pill drug each day. The other half got chemo every few weeks, followed by a lighter chemo. Doctors looked at how long people stayed free from worse cancer. This was checked by doctors who did not know what drug the person got, to keep things fair.  Findings/Results:  Both groups stayed free from worse cancer for about the same time. The new pill drug did not help people more than usual chemo. A few more people had a helpful change in their cancer with the pill drug than with chemo. Also, people taking the pill drug waited longer before they felt worse from cancer in the lung, had trouble thinking, or had hard stools. Some people had bad effects like runny bowels with the new pill, while people on chemo had low blood count and tiredness.  Conclusion:  The study showed that the pill drug mobocertinib did not work better than usual chemo. So, for this kind of lung cancer, the new drug is not a better choice than the old one. More studies may be needed to learn new ways to treat this cancer better.  Short Summary:  The study looked at people with a type of lung cancer caused by a gene change. It compared a new pill drug to usual chemo. Both worked the same for stopping worse cancer. The new pill helped people feel better longer but had some bad effects. In the end, the pill was not better than chemo. More research may be planned to find better ways to treat this kind of cancer. | Background/Introduction:  A kind of lung cancer called non-small cell lung cancer can happen in people when a part of their body’s cells changes in a special way. Some of these changes are called EGFR exon 20 insertions. This makes the cancer hard to treat. Doctors wanted to know if a new pill called mobocertinib works better at the start than the older kind of drug treatment, called chemotherapy, for this type of lung cancer.  Methods:  People with this type of lung cancer, who had not yet gotten any treatment, were split into two groups. One group took mobocertinib by mouth daily. The other group got chemotherapy through a drip every few weeks. The researchers looked to see how long people lived without the cancer growing and how well each treatment worked.  Findings/Results:  Each group had almost the same number of people. The results showed that both treatments stopped cancer from growing for about the same time. The number of people who showed improvement was close in both groups. But, the people who took the pill stayed better for longer before their cancer came back. Also, the pill helped people feel better for longer, with fewer problems in breathing and thinking. However, some people on the pill had more side effects like runny tummy or changes in blood tests.  Conclusion:  The study showed that the new pill did not work better overall than the usual drip medicines. So, the pill is not a clear winner to start with for this kind of lung cancer. Still, the pill helped some people feel better for longer, and some effects lasted longer. More studies may help find out who could gain the most from this pill.  Short Summary:  Doctors tested if a pill named mobocertinib helps people with a hard-to-treat lung cancer more than old-style drip treatment. Both treatments worked about the same to stop the cancer from growing. The pill helped people feel better longer but had some more side effects like runny tummy. The new pill does not clearly work better, but it could help some people. More checking may help learn who should use it first. |
| JCO | 3 | 10.1200/JCO-24-01441 | Pub2Post | PURPOSETo evaluate the cost utility of a 9-month supervised exercise program for patients with metastatic breast cancer (mBC), compared with control (usual care, supplemented with general activity advice and an activity tracker). Evidence on the cost-effectiveness of exercise for patients with mBC is essential for implementation in clinical practice and is currently lacking.METHODSA cost-utility analysis was performed alongside the multinational PREFERABLE-EFFECT randomized controlled trial, conducted in 8 centers across Europe and Australia. Patients with mBC (N = 357) were randomly assigned to either a 9-month, twice-weekly, supervised exercise group (EG) or control group (CG). Costs of the exercise program were calculated through a bottom-up approach. Other health care resource use, productivity losses, and quality of life were collected using country-adapted, self-reported questionnaires. Analyses were conducted from a societal perspective with a time horizon of 9 months. Costs were collected and reported in 2021 Euros (1 = $1.18 US dollars).RESULTSCompared with the CG, EG resulted in a quality-adjusted life-year (QALY) gain of 0.013 (95% CI, -0.02 to 0.05) over a 9-month period. The mean costs of the exercise program were 1,696 per patient with one-on-one supervision (scenario 1) and 609 with one-on-four supervision (scenario 2). These costs were offset by savings in health care and productivity costs, resulting in mean total cost differences of -163 (scenario 1) and -1,249 (scenario 2) in favor of EG. The probability of supervised exercise being cost-effective was 65% in scenario 1 and 91% in scenario 2 at a willingness-to-pay threshold of 20,000 per QALY.CONCLUSIONExercise for patients with mBC increases quality of life, decreases costs, and is likely to be cost-effective. Group-based supervision is expected to have even higher cost-savings. Our positive findings can inform reimbursement of supervised exercise interventions for patients with mBC. | Background/Introduction  This study looked at how helpful a workout plan is for people with spread breast cancer. Spread breast cancer means the disease has moved to other parts of the body. Many people with this type of cancer feel weak and tired. Doctors think moving more might help them feel better. But they do not know for sure if the cost of setting up these exercise plans is worth it. This study wanted to see if the health gains from exercise are worth the money spent.  Methods  The study picked people with spread breast cancer from many places in Europe and Australia. Some people did a workout plan with a coach. They moved their bodies two times each week for almost a year. The other people got the usual advice to stay active. These two groups were studied and compared. The study checked how good each person felt and how much it cost to care for them. They also looked at things like time missed from work because of being sick. All money counts were based on amounts from a few years ago.  Findings/Results  People who joined the workout plan felt a little better in health than those who only got advice. It cost some money to run the workout plan, but the people in the workout group needed a bit less health care and missed less work. That helped save money in other areas. The plan with a group coach saved more money than having one coach per person. Most of the time, the workout plan cost less than what people would be okay paying for feeling better.  Conclusion  The study found that gentle, guided movement helps people with spread breast cancer feel a bit better. It may also cost less in the long run. Having a coach help a few people at the same time saves even more money. Based on the results, doctors can now say that adding coached exercise may be a smart idea. Using this plan may be good for both health and for saving money. More studies may come to see if this also works in other places and for more people.  Short Summary  People with spread breast cancer often feel weak and tired. This study found that gentle workouts with a coach helped them feel a bit better and may even lower health costs. Sharing one coach across a small group saved even more money. This plan could be a smart and helpful way to care for these patients. It may also guide future work on better and cheaper care. | Background/Introduction  This study looked at women who have breast cancer that has spread to other parts of their body. Doctors wanted to know if helping these women exercise in a planned way would help them feel better and if it would also save money. So far, there has not been enough proof that this kind of exercise is worth the cost. This study tried to find out if it really helps and if it is not too costly.  Methods  More than three hundred women from eight places in Europe and Australia took part in this study. Each woman was put in one of two groups. One group got help with exercise from a trainer two times each week for nine months. The other group was told to be active and given a step tracker. The people in charge checked how the programs affected how the women felt and how much money they spent on health or missed work during those months. The costs were counted in money used in Europe.  Findings/Results  The women who got help with exercise felt a little better in daily life than those who did not. They also needed less help from doctors and missed less work. The one-on-one sessions cost more at first, but this money was balanced by saving money in other places, like fewer doctor visits. The group sessions with one trainer and four women cost even less and saved even more money. So, exercising with help seemed like a good way to help women feel better without costing more.  Conclusion  Helping women with this kind of cancer to keep moving with a trainer can help them feel better and may also save money in the long run. When done as a group instead of one-on-one, the savings are even higher. This study shows why health providers might want to pay for these programs in the future.  Short Summary  This study looked at women with breast cancer that has spread. One group did guided workouts while the other got tips to stay active. The women who had guided workouts felt a bit better and needed less doctor care. Group exercise saved more money than solo sessions. Helping these women move more can improve their lives and save costs. These findings may help health leaders decide to support such programs. Further studies may build on this. | Background/Introduction:  This study looked at whether a special workout plan can help people who have breast cancer that has spread to other parts of the body. These people often feel tired or weak, and doctors don’t yet know if planned exercise saves money or helps enough to be worth using in clinics.  Methods:  People with advanced breast cancer joined this study across different places in Europe and Australia. Some took part in a workout plan with a coach twice each week for almost one year. Others got usual care with health tips and an activity watch. The team then added up all health costs, time away from work, and how people felt about their life during that time.  Findings/Results:  People who did the workouts felt a bit better in their daily life. The money spent on these workouts was balanced out by lower spending on doctor visits and sick leave from work. When the workout coach helped a small group at once, the cost was lower, and the savings were bigger. The chance that these workouts save enough money and bring good results was high, especially in small group sessions.  Conclusion:  This study shows that workouts with a coach help people with breast cancer feel a bit better. This care also may end up costing less overall. If the workouts are done in small groups, they save even more. This means doctors may soon offer such workout plans to help more patients.  Short Summary:  The study looked at people with breast cancer that had spread. Some of them joined planned workouts, while others got usual care. The workouts helped people feel a bit better and saved money, especially when done in groups. Doctors may start using these kinds of group plans more in the future. |
| JCO | 4 | 10.1200/JCO-24-02086 | Pub2Post | PURPOSE Cyclin-dependent kinase 4/6 inhibitors (CDK4/6i) combined with endocrine therapy (ET) are the standard first-line treatment for hormone receptor-positive (HR+), human epidermal growth factor receptor 2-negative (HER2-) advanced breast cancer (ABC); however, disease progression occurs in almost all patients and additional treatment options are needed. Herein, we report outcomes of the postMONARCH trial investigating a switch in ET with/without CDK4/6 inhibition with abemaciclib after disease progression on CDK4/6i. METHODS This double-blind, randomized phase III study enrolled patients with disease progression on previous CDK4/6i plus aromatase inhibitor as initial therapy for advanced disease or recurrence on/after adjuvant CDK4/6i + ET. Patients were randomly assigned (1:1) to abemaciclib + fulvestrant or placebo + fulvestrant. The primary end point was investigator-assessed progression-free survival (PFS). Secondary end points included PFS by blinded independent central review, objective response rate (ORR), and safety. RESULTS This study randomly assigned 368 patients (abemaciclib + fulvestrant, n = 182 placebo + fulvestrant, n = 186). At the primary analysis (258 events), the hazard ratio (HR) was 0.73 (95% CI, 0.57 to 0.95; nominal P = .017), with median PFS 6.0 (95% CI, 5.6 to 8.6) versus 5.3 (95% CI, 3.7 to 5.6) months and 6-month PFS rates of 50% and 37% in the abemaciclib + fulvestrant and placebo + fulvestrant arms, respectively. These results were supported by BICR-assessed PFS (HR, 0.55 [95% CI, 0.39 to 0.77]; nominal P < .001). A consistent treatment effect was seen across major clinical and genomic subgroups, including with/without ESR1 or PIK3CA mutations. Among patients with measurable disease, investigator-assessed ORR was improved with abemaciclib + fulvestrant versus placebo + fulvestrant (17% v 7%; nominal P = .015). No new safety signals were observed, with findings consistent with the known safety profile of abemaciclib. CONCLUSION Abemaciclib + fulvestrant significantly improved PFS after disease progression on previous CDK4/6i + ET in patients with HR+, HER2- ABC, offering an additional targeted therapy option for these patients. | Background/Introduction:  People with a certain type of breast cancer often start with a mix of hormone therapy and special drugs that slow cancer growth. But these drugs stop working over time. So, new treatments for when the cancer gets worse are important. This study looked at whether a mix of two other drugs worked better when the earlier treatment had stopped helping.  Methods:  The study included women with a kind of breast cancer that does not have a certain protein called HER2 and does react to hormones. All of them had already tried one kind of treatment that no longer worked. They were split into two groups by chance. One group got a mix of two drugs called abemaciclib and fulvestrant. The other group got a pretend drug (called a placebo) and fulvestrant. The doctors mainly watched how long it took for the cancer to get worse again.  Findings/Results:  The group that got the two real drugs had more time before their cancer got worse. More of these women also saw their tumors shrink a bit. This happened even in women with changes in certain genes. The side effects were what doctors expected from past use of these drugs. There were no big surprises in the safety of the treatment.  Conclusion:  This mix of drugs helped slow down the cancer better than the older mix after the first treatment stopped helping. It worked for many types of the cancer and was safe. This could give doctors a new way to treat this kind of cancer. More studies may follow to learn even more.  Short Summary:  Women with breast cancer that grew after one kind of drug treatment were tested with a new drug pair. This new mix helped keep the cancer from getting worse for a longer time. It worked for different kinds of patients and did not cause new safety concerns. This mix may become a better choice for women when the first drugs no longer help. Future studies may look at more ways it can be used. | Background/Introduction:  Many women with a certain type of breast cancer first get a mix of hormone medicines and special drugs called cell-blockers. But after some time, the cancer may still get worse. These women then need new treatment choices. This study looked into whether using abemaciclib (a cancer drug that stops some cell growth) with fulvestrant (a hormone-blocking medicine) could help, after that first treatment stops working.  Methods:  Women whose cancer had gotten worse after earlier drug treatment joined this study. The study gave these women one of two treatments: some got abemaciclib plus fulvestrant, and some got just fulvestrant with a fake pill. The women were picked by chance for one of the two groups. The doctors wanted to see how long it took for cancer to get worse again. They also checked how many women’s tumors got smaller and if the treatment was safe.  Findings/Results:  About half of all the women got the drug combo with abemaciclib. The women who got both drugs stayed better a bit longer before the cancer got worse again. More women in the combo group had their tumors shrink compared to the other group. The treatment worked well for many groups of women, even with different gene changes. The side effects were pretty much what doctors already knew could happen with this drug and there were no big new safety problems.  Conclusion:  The drug combo with abemaciclib helped women with this kind of breast cancer stay well a bit longer and helped some women’s tumors shrink. So, these two drugs together offer a new option after the first treatment stops helping.   Short Summary:  This study tested if two drugs—abemaciclib and fulvestrant—could help women whose breast cancer came back after their first treatment. Half the women got both drugs, and they stayed well a bit longer than those who only got one drug. More of them even had smaller tumors. The treatment worked even in women with gene changes. No new safety problems showed up. The results show these two drugs can be a helpful choice. The study did not say if more research is coming, but the findings may lead to it. | Background/Introduction  People with a certain type of breast cancer often get help from two kinds of medicine: hormone blockers and drugs that stop cancer cell growth. But over time, in most cases, the cancer gets worse, and these people need a new treatment. A study called postMONARCH tested if changing the hormone medicine and adding a drug called abemaciclib could help those whose cancer worsened after the first treatment.  Methods  In this study, people whose breast cancer worsened after earlier treatment were split into two groups. One group got abemaciclib with another hormone drug called fulvestrant. The other group got a fake drug (a placebo) with fulvestrant. Neither the people in the study nor the doctors knew who got which. The study looked at how long these people lived without their cancer getting worse. It also looked at how many people had their cancer shrink and if the drugs caused any problems.  Findings/Results  Over three hundred people were in the study. About half got abemaciclib with fulvestrant, and the rest got the placebo with fulvestrant. People who took abemaciclib stayed free from cancer growth longer than people who did not. More people in the abemaciclib group also saw their tumors get smaller. The new drug did not cause more side effects than what doctors already knew about it.  Conclusion  This study showed that adding abemaciclib to fulvestrant helped slow the cancer in people whose illness got worse after earlier care. This gives doctors and patients a new choice for this kind of breast cancer.  Short Summary  This study looked at people with a type of breast cancer that grew worse after early treatment. It tested adding the drug abemaciclib to another hormone drug called fulvestrant. People who got the mix of these two drugs did better than those who got only the hormone drug. Their cancer took longer to grow and more saw their tumors shrink. The new drug was safe and gives patients a better option. More research may be done, but this one shows promise. |
| JCO | 5 | 10.1200/JCO.24.00342 | Pub2Post | PURPOSE Guidelines recommend cardiovascular (CV) risk assessment and counseling for cancer survivors. This study evaluated the automated heart-health assessment (AH-HA) clinical decision support tool to promote provider-patient CV health (CVH) discussions in outpatient oncology. METHODS The AH-HA trial (WF-1804CD), coordinated by the Wake Forest National Cancer Institute Community Oncology Research Program Research Base, randomized practices to the AH-HA tool or usual care (UC) and enrolled survivors receiving routine care >= 6 months after curative cancer treatment. The tool displayed American Heart Association Life's Simple 7 CVH factors (BMI, physical activity, diet, smoking status, blood pressure, cholesterol, and glucose), populated from the electronic health record (EHR), alongside cancer treatments received with cardiotoxic potential. The primary end point was survivor-reported discussion of nonideal or missing CVH factors. A mixed-effects logistic regression model assessed the effect of AH-HA on CVH discussions, adjusting for practice. RESULTS Five UC and four AH-HA practices enrolled 645 survivors (82% breast, 8% endometrial, 5% colorectal, and 5% lymphoma, prostate, or multiple types) from October 1, 2020, to February 28, 2023. Most survivors were female (96%; 84% White/non-Hispanic, 8% Black; 3% Hispanic). Nearly all survivors (98%) in AH-HA practices reported a discussion for >= 1 nonideal or missing CVH factor compared with 55% in UC (P < .001). The average number of survivor-reported factors discussed was higher in AH-HA compared with UC (mean, 4.06 v 1.27; P < .001), as were EHR-documented discussions (3.83 v 0.77; P = .03). Survivors in AH-HA practices were also significantly more likely to report a recommendation to see a primary care provider (39%) compared with UC practices (25%, P = .02). Reported recommendations to see a cardiologist were low (approximately 6%) and did not differ between groups. CONCLUSION The AH-HA tool was effective at promoting CVH discussions during routine follow-up care for survivors and recommendations to consult primary care. | Background/Introduction:  People who had cancer in the past face a higher risk of heart problems. Doctors suggest that these people get heart health checks and talk with their care team about it. This study looked at a new tool that helps doctors and patients talk about heart health during cancer follow-up visits. The tool is built into the patient’s medical chart on the computer.  Methods:  This study took place in cancer clinics. Some doctors used the heart health tool, while others gave usual care. People who had finished their cancer treatment at least half a year ago joined the study. The tool showed key things about heart health, like weight, food, movement, blood pressure, blood sugar, and smoking. It also showed past cancer care that could be hard on the heart. The study checked if people and doctors talked more about these heart health risks when the tool was used.  Findings/Results:  Most of the people in this study had breast cancer, and many were white women. In the groups that used the heart tool, almost all people said they talked with their doctor about at least one heart health issue. In the usual care group, only about half had this kind of talk. People in the tool group talked about more heart health topics and were more likely to be told to visit their regular doctor. Talks about seeing a heart doctor were low in both groups.  Conclusion:  The heart health tool helped people and their cancer doctors talk more about heart risks during follow-up care. It also helped people get advice to visit their regular doctor. This shows the tool works well and may help others in the future.  Short Summary:  This study tested a heart health tool that helps cancer patients and doctors talk about staying heart-healthy. Most people had finished cancer care and joined regular visits. The tool helped bring up talks on weight, food, movement, blood pressure, and more. People using the tool had better talks and got more advice to follow up with their own doctor. This tool may help keep people healthier after cancer. More studies may look at how well it works over time. | Background/Introduction:  People who have beaten cancer can still face heart problems later on. Doctors suggest that these people should check their heart health often. But these talks about heart health do not always happen during visits. A tool called AH-HA was made to help doctors and patients talk more about heart health.  Methods:  This study looked at how well the AH-HA tool worked. Some cancer care offices used the tool, and others did not. The people in the study were all done with cancer treatment for at least half a year. The tool showed heart health facts like weight, how active a person is, what they eat, if they smoke, and other things. It also showed any cancer treatments that might harm the heart. The main thing the study looked at was if patients said they talked with their doctor about problems or missing notes about their heart health.  Findings/Results:  People in offices that used the tool were much more likely to talk with their doctor about heart health issues than those at offices without the tool. Most people using the tool talked about at least one heart health concern, while very few did in regular offices. People with the AH-HA tool also talked about more heart topics. Doctors were more likely to suggest these people visit their family doctor. Only a small number were told to see a heart specialist, and this was the same in both groups.  Conclusion:  The AH-HA tool helped doctors and patients talk more about heart health after cancer care. It also helped doctors give better advice to visit a family doctor. The study shows that tools like this can make a big difference and should be used more.  Short Summary:  This study looked at a tool made to help doctors talk to people who had cancer about their heart health. People using the tool had many more heart talks with their doctors than those who did not. The tool also helped doctors give better advice, like seeing a family doctor. These talks can help keep survivors healthy. More use of the tool is a good idea. | Background/Introduction:  People who survive cancer need to take care of their heart health, but many don't talk to their doctors about it. This study looked at a new computer tool that helps doctors and patients speak about heart health during visits. The goal was to see if the tool works better than normal care.  Methods:  This study looked at people who had finished cancer care at least half a year ago. They were part of different doctor’s offices. Some offices used the new tool. Others did not, and gave regular care. The tool showed heart risk signs like weight, how much they move, what they eat, if they smoke, blood pressure, fat in the blood, and blood sugar. The tool also showed past cancer treatments that might hurt the heart. Doctors and patients saw this on a screen.  Findings/Results:  Most people in the group with the tool said they talked with their doctor about at least one heart health issue. Far fewer people in the regular care group had this kind of talk. People with the tool talked about more heart health issues than those who did not have the tool. Doctors who used the tool also wrote down more talks about heart facts in the patient records. More people using the tool were told to go see their main health doctor, but only a small group in both parts were told to see a heart doctor.  Conclusion:  The tool helped doctors and patients talk more about heart health. It worked well during normal check-ups for cancer survivors. It also helped get people to see their regular doctor to work on heart issues.  Short Summary:  This study looked at people who beat cancer and checked if a new tool helped doctors talk more about heart health. The tool showed heart risks and past cancer care side effects. It helped make heart talks happen more often. People using the tool had more full visits and were often told to check in with their main doctor. The study shows this tool helps keep survivors’ hearts safe and strong. More studies may happen soon to look at long-term effects. |
| NatRevCan | 1 | 10.1038/s41568-025-00810-1 | Pub2Post | Phosphoinositide kinases, extending beyond the well-known phosphoinositide 3-kinase (PI3K), are key players in the dynamic and site-specific phosphorylation of lipid phosphoinositides. Unlike PI3Ks, phosphatidylinositol 4-kinases (PI4Ks) and phosphatidylinositol phosphate kinases (PIPKs) do not usually exhibit mutational alterations, but mostly show altered expression in tumours, orchestrating a broad spectrum of signalling, metabolic and immune processes, all of which are crucial in the pathogenesis of cancer. Dysregulation of PI4Ks and PIPKs has been associated with various malignancies, which has sparked considerable interest towards their therapeutic targeting. In this Review we summarize the current understanding of the lesser-studied phosphoinositide kinase families, PI4K and PIPK, focusing on their functions and relevance in cancer. In addition, we provide an overview of ongoing efforts driving the preclinical and clinical development of phosphoinositide kinase-targeting molecules. | Background/Introduction: Some tiny cell parts called kinases help control how cells grow and work. A few of these kinases deal with fat-like parts in cells and are called phosphoinositide kinases. Some kinds of these kinases, which were not well studied before, seem to be very active in many types of cancer. This means they might be doing things that help cancer grow and spread.  Methods: The researchers looked at past work done by other scientists to gather more facts. They studied how these specific kinases act inside the body. They also looked at how these kinases may help cancer happen. The goal was to see if these kinases could be used to find or treat cancer.  Findings/Results: The research showed that two types of these kinases, called phosphatidylinositol four-kinases and phosphatidylinositol phosphate kinases, don’t change because of gene mistakes. Still, they act too much inside cancer cells. This affects how the body signals, uses energy, and fights illness. These acts can lead to many types of cancer. Because of this, new lab work and people tests are now trying to stop these kinases with special drugs.  Conclusion: These lesser-known kinases have a big part in cancer. Even if they don’t have gene errors, they still cause trouble when they act too much. Many are now trying new ways to stop them from working so hard. These ideas may help treat cancer in the future.  Short Summary: Some cell helpers called kinase groups are more active in cancer. Two special types work on fat-like parts in cells and are not usually changed by genes, but they still make cancer worse when they act a lot. Scientists are now trying new drugs to stop them. This may help treat cancer. More studies are being done to see how well this works. | Background/Introduction:  This study looks at a group of small tools in the body called phosphoinositide kinases. These tools help send signals in cells. One well-known type is PI3K, already linked to cancer. But this paper focuses on other types, less studied, called PI4K and PIPK. These other types don’t often change shape like some cancer-causing ones. Instead, their levels go up or down in tumors. When this happens, they may cause changes in cell work, body energy use, or how the body fights germs. This can help cancer grow.  Methods:  This is a review paper. That means the authors did not test on people or animals. Instead, they looked at past work by others. They picked many papers that talk about the role of these less-known kinases in cancer. They put this work together to explain better how these tools work and how they might help treat cancer in the future.  Findings/Results:  The authors found that PI4Ks and PIPKs are found at odd levels in many kinds of tumors. These changes make the cells act in the wrong way. This helps cancer grow or stay in the body longer. Scientists now think these tools could be good new targets for cancer drugs. Work is already going on to test these ideas with trial drugs. Some are still being tested in labs while others are moving to early tests in people.  Conclusion:  PI4Ks and PIPKs, two lesser-known tools in the body, help support cancer in quiet ways. Even though they don’t change shape like some cancer genes, their strange levels are important. Because of this, they might help us find new ways to treat cancer. More research is needed to learn the safest and best ways to use these tools to stop or slow down cancer.  Short Summary:  This paper reviewed older studies on two lesser-known body tools, PI4K and PIPK, which send signals in cells. These tools act strange in tumor cells and may help cancer grow. While they don’t change shape like old-known cancer tools, their odd levels can cause problems. Many scientists are now looking at these tools to see if new cancer drugs might stop them. Work is still ongoing to test these ideas, but early signs show they could play a big part in future cancer care. | Background/Introduction:  Cells in our body use fats and signals to talk to each other and grow. Some tiny tools in cells, called phosphoinositide kinases, help control these fats. One group of these tools, called phosphoinositide 3-kinase, is already known to be important in cancer. But there are other groups, like phosphatidylinositol 4-kinases and phosphatidylinositol phosphate kinases, that we don’t know as much about. These are not often changed by broken genes, but their levels go up or down in cancer. When that happens, it can hurt how the body works, like its signals, energy use, and defense cells. This may help cancer grow.  Methods:  In this study, the team read many past reports to learn more about how these lesser-known cell tools work. They looked at lab tests and early-stage human tests. They focused on the role of these tools in how cancer grows and spreads. They also explored ways to block these tools to try to stop cancer.  Findings/Results:  The researchers found that when these cell tools act in the wrong way, they can help some cancers grow. The tools affect many steps that cells use to live, grow, and move. Turning down these tools in experiments sometimes helped fight cancer cells. Because of this, some drug makers are now testing new ways to block these cell tools.  Conclusion:  This study says that these lesser-known cell tools are very important in cancer. Even if genes in them do not change much, their wrong activity still helps cancer grow. There are new drug ideas that could stop these tools. These may help patients if used with other cancer drugs. More studies on people are needed before they can be used in health centers.  Short Summary:  This study looked into lesser-known tools in cells that help manage fat-based signals. These tools, which don’t change much through broken genes, still often act wrong in cancer. When they do, they help cancer cells grow and spread. Some new drug ideas may stop these tools and help treat cancer. More research is needed to prove this. |
| NatRevCan | 2 | 10.1038/s41568-024-00788-2 | Pub2Post | Tumours of the appendix - a vestigial digestive organ attached to the colon - are rare. Although we estimate that around 3,000 new appendiceal cancer cases are diagnosed annually in the USA, the challenges of accurately diagnosing and identifying this tumour type suggest that this number may underestimate true population incidence. In the current absence of disease-specific screening and diagnostic imaging modalities, or well-established risk factors, the incidental discovery of appendix tumours is often prompted by acute presentations mimicking appendicitis or when the tumour has already spread into the abdominal cavity - wherein the potential misclassification of appendiceal tumours as malignancies of the colon and ovaries also increases. Notwithstanding these diagnostic difficulties, our understanding of appendix carcinogenesis has advanced in recent years. However, there persist considerable challenges to accelerating the pace of research discoveries towards the path to improved treatments and cures for patients with this group of orphan malignancies. The premise of this Expert Recommendation article is to discuss the current state of the field, to delineate unique challenges for the study of appendiceal tumours, and to propose key priority research areas that will deliver a more complete picture of appendix carcinogenesis and metastasis. The Appendix Cancer Pseudomyxoma Peritonei (ACPMP) Research Foundation Scientific Think Tank delivered a consensus of core research priorities for appendiceal tumours that are poised to be ground-breaking and transformative for scientific discovery and innovation. On the basis of these six research areas, here, we define the first 'cells to society' research framework for appendix tumours. | Background/Introduction  The appendix is a small part of the gut with no clear use. When it gets sick with cancer, it is hard to find out. Doctors find very few of these cases each year in the United States, but the real number might be higher. Right now, there are no special checks to find this kind of cancer early. Most of the time, doctors find it by accident, when someone has belly pain or when the cancer has already spread. Some appendix cancer cases even get mixed up with other kinds like colon or ovary cancer. Over time, we have learned more about how this cancer grows, but it's still hard to make new and better treatments.   Methods  A group of experts came together to talk about what makes appendix cancer hard to study. They also shared ideas on what future studies should look at. This group was brought together by a group called the Appendix Cancer Pseudomyxoma Peritonei Research Foundation. After talking and sharing their views, the experts made a plan to guide future studies. They grouped this plan into six main ideas that deal with different parts of the problem — from tiny body cells to how people and groups are affected.   Findings/Results  The experts found key roadblocks that slow down science on appendix cancer. These include trouble with finding the cancer early, not having enough people to study, and the fact that this type of cancer is not well known. They also pointed out areas where more study can have a big effect. This includes better ways to find the disease, smart ways to treat it, and how to help patients and their families deal with it.   Conclusion  Appendix cancer is rare and tough to spot early. It’s often found by accident and may be mistaken for other diseases. A new plan with six goals has now been created to help guide future study on how this cancer grows and spreads. The hope is that, by doing more of the right studies, new ways to treat and maybe cure the disease will come sooner. Many more studies will likely need to follow this plan to help researchers and doctors.   Short Summary  Appendix cancer is hard to spot and often found late. Experts made a new plan with six main goals to guide future study. These goals focus on how the cancer starts, how to treat it, and how it affects people. The study points out that better checks and care are needed. More follow-up work will help bring better answers for patients. | Background/Introduction:  The appendix is a small part of the gut that people do not need. Tumors can grow there, but this is rare. Experts guess that only a few thousand people in the United States are told they have this kind of tumor each year. But this guess may be too small because these tumors are hard to find. There are no special tools to check for these tumors, and most people only learn they have one during an emergency or when the cancer has already spread. Sometimes these tumors are mixed up with other cancers, such as those in the large gut or in the ovaries. Even though it's hard to spot, our knowledge about these tumors has grown in recent years. Still, more work needs to be done to find better ways to help people.  Methods:  A group of health experts came together and shared ideas. They discussed what we know about these appendix tumors and the problems keeping progress slow. They wanted to find the most important questions about how these tumors grow and spread. From this, they built a plan for research that starts from looking at cells and goes all the way to how people and communities are affected.  Findings/Results:  The expert group chose six big ideas to focus the research. These ideas aim to help us better understand how appendix tumors start, grow, and move to other body parts. Their plan looks at many parts of life, like body changes, health care, and even people’s lives in the real world. The main goal is to speed up research and come up with better care and support for people with this disease.  Conclusion:  With this new plan, we hope to learn much more about these rare tumors. It will take different kinds of studies that look at both body cells and how people live. This idea could lead to new ways to find, treat, or even stop the disease. The group hopes this research will help people get better care and may lead to future studies.  Short Summary:  This paper talks about rare tumors found in the appendix. These are hard to detect and are often found by accident. Health experts worked together to find the most important questions about this disease. They picked six big ideas to guide the research. Their plan looks at both the body and how people live with cancer. This could help speed up new findings and help improve care for people. More research is likely in the future. | Background/Introduction  The appendix is a small pouch near the large gut. Sometimes it grows cancer, though this is very rare. Each year, only a small group of people in the United States are told they have appendix cancer. But many cases may be missed or confused with other cancers, like those from the gut or female parts. These tumors are hard to find early because there are no clear tests or signs. Often, people find out by chance when they feel pain like appendicitis or the cancer has spread in the belly. Even with these problems, doctors have learned more about how this cancer begins. But there is still a lot we do not know. This makes it hard to find better ways to help patients.  Methods  A group of experts worked together to study this cancer more closely. They belong to a group that helps with research on appendix cancer. They talked about the biggest problems in the field. Then they picked six top ideas to study further. They used these ideas to make a new plan to help guide future research from basic cell work to how it affects people and homes.  Findings/Results  The experts said we need to study many parts of appendix cancer. This includes how it starts, how it spreads, and which groups of people may get it more. They also said we need to make better tools to study it and improve ways to tell it apart from other cancers. They pointed out that more people need to know about this rare cancer so it can be found earlier. The group made a plan for six key areas of study. These areas cover both tiny body parts (cells) and large ideas (like care access and family impact). Their plan can help make new tools, better treatments, and raise hope for faster progress.  Conclusion  Appendix cancer is often not found on time and has been hard to study. This group of experts gave a helpful list of what we do not know and what we must learn. Their new research plan will help us get better at finding, treating, and understanding this rare cancer. The plan can also help people living with the cancer and may spark new studies in the future. With this map, science can move faster and in the right path.  Short Summary  Appendix cancer is rare and hard to spot early. Many people only learn they have it when it causes belly pain or has already spread. Experts met and made a list of six key study areas. These cover how the cancer grows, spreads, and how to better care for people with it. Their plan helps guide research from tiny body parts to home and care life. This could bring new tools, better care, and more hope for people with appendix cancer. More studies might follow to build on this work. |
| NatRevCan | 3 | 10.1038/s41568-025-00841-8 | Pub2Post | In the ongoing battle against cancer, the natural world provides promising inspiration for designing novel therapeutic strategies. The field of comparative oncology offers a valuable source of such inspiration. By combining evolutionary biology, ecology, veterinary medicine and clinical oncology, comparative oncology aims to better understand cancer, especially by highlighting taxa that are strongly resistant or susceptible to cancer and to identify the molecular and cellular mechanisms underlying the remarkable cancer resistance of some taxa. Such studies hold profound implications for human cancer research and treatment, and increase the probability of detecting therapeutic avenues that are non-toxic to healthy cells and tissues. This Perspective underscores the importance of comparative oncology, emphasizes its relevance, and showcases recent breakthroughs in identifying natural cancer resistance mechanisms and opportunities for clinical translation. We advocate for a better integration of cancer research on non-conventional model species into oncology and we urge enhanced cooperation between clinicians and comparative oncologists to advance cancer prevention or treatment strategies. | Background/Introduction:  Some plants and animals do not get sick with cancer as often as people do. Scientists want to learn why. They hope this can help them find safer and better ways to stop or treat cancer in people. A field called comparative oncology — which means studying cancer in different living things — helps with this. It brings together ideas from nature, health care for animals, human medicine, and how living things grow and change over time. This study looks at how this field can help doctors discover new ways to fight cancer.  Methods:  The study brings together work from many fields: how living things grow and change (evolution), how they live in the world (ecology), animal health, and human cancer care. Scientists look at animals that do not often get cancer and try to find out why. They also study animals that get sick more often. They focus on what is happening inside the cells and how the body protects itself from cancer.  Findings/Results:  Some animals hardly ever get cancer, which is strange because their bodies are large or they live a long time. These animals have special ways to protect their cells. Scientists have started to find how these ways work. These clues may lead to treatments that stop cancer without hurting the rest of the body. This research could help make safer drugs for people.  Conclusion:  More teamwork is needed between doctors who treat people and those who study animals. Working together can help create new ways to stop or treat cancer. Learning from animals that resist cancer well could give people safer care. This study says we should pay more attention to animals that are not used much in labs and study how they fight off cancer.  Short Summary:  This study explores how learning from animals and nature can help us better stop or treat cancer. Some animals rarely get cancer, and finding out why can guide doctors to make safer treatments for people. The study says doctors and scientists should work more closely together. They also suggest using more types of animals in research, not just the usual ones, so we can learn more. | Background/Introduction  Some living things rarely get cancer, while others get it often. Doctors want to know why. They look at animals who do not get cancer to help them learn new ways to stop or treat cancer in people. This kind of study is called "comparative oncology." It uses ideas from how animals grow, live, and get sick. It also mixes animal and human medicine. The goal is to find out why some animals fight cancer so well.   Methods  Doctors and scientists studied many animals. They looked at how cancer acts in those animals and how their bodies keep cancer away. Then, they used this new learning to think of new ways to treat cancer in people. They work together and share what they find.  Findings/Results  Some animals show strong ways to keep from getting cancer. By learning what their bodies do, doctors found new ways that might help treat cancer in people. These ways may be safe and not harm the good parts of the body. Some of the things scientists found might be used one day in hospitals to help people.   Conclusion  Looking at how animals fight cancer helps doctors find new answers. This kind of work can lead to better care for people. It is best when doctors of animals and people work as one team. They hope to work more like this in the future.   Short Summary  Scientists want to learn more about cancer by studying animals. They found that some animals do not get cancer much and want to know why. This study helps find new ways to treat cancer in people that do not hurt the rest of the body. Doctors from human and animal care work together. They plan to keep working on this to find better care for people with cancer. | Background/Introduction  Some animals in nature get sick with cancer less often than others. Scientists want to learn why this happens. This can help them think of new and better ways to treat or stop cancer in people. A group of scientists is using a way called “comparative oncology.” This means they look at cancer in both animals and people. They try to see which animals fight cancer well and how they do it.   Methods  The scientists used ideas from many fields, like how living things change over time (this is called evolution), how living things interact in nature (this is called ecology), and how animal doctors and people doctors treat sickness. They looked closely at animals that almost never get cancer. They checked what happens to the tiny parts in their bodies to better understand how they stay healthy.   Findings/Results  These scientists found many animals that have strong ways to stop cancer. Some animals seem to have cells, which are the basic building blocks of every body, that fight off cancer fast. Others stop it before it even starts. These ways do not hurt healthy body parts. Their findings give clues on how to make cancer drugs that do not cause harm to the rest of the body.   Conclusion  Looking at cancer in animals helps doctors come up with better tools to treat people. This study shows it is very helpful to work with all sorts of animals, not just the few often used in labs. It also shows that human doctors and animal doctors need to team up more. By doing this, they can find safer and quicker ways to stop cancer.   Short Summary  Scientists studied animals that do not get cancer often. They used many tools to find out how these animals avoid the disease. Their work may help doctors treat people without hurting good parts of the body. They say that doctors for people and animals must work together. More studies like this could lead to better cancer care in the future. |
| NatRevCan | 4 | 10.1038/s41568-025-00831-w | Pub2Post | The nervous and immune systems have co-evolved to detect and respond to internal and external threats, working together to restore homeostasis after tissue injury or infection. Sharing several receptors and ligands, they engage in direct cross-talk that substantially influences disease development. The emerging field of cancer neuro-immunity focuses on the intricate interactions between the nervous system, immune responses and tumour growth. Additional findings have revealed that nerve fibres infiltrating peripheral tumours can release neuromodulatory factors that shape both immune cell behaviour and tumour progression. Conversely, tumour-infiltrating immune cells can modify the activity of local neurons, including pain-transmitting nociceptive sensory neurons. Beyond sensory fibres, sympathetic signalling can foster immunosuppression by recruiting myeloid-derived suppressor cells and promoting T cell exhaustion. This Review summarizes current evidence on how neuronal signalling regulates peripheral antitumour immune responses within the tumour microenvironment. We describe the complex, reciprocal interactions among neurons, immune cells and malignant cells, highlighting the key parts played by the peripheral nervous system in modulating immunity against cancer. By understanding this neuro-immune axis, novel therapeutic approaches may be uncovered to strengthen antitumour immunity and enhance responses to existing cancer treatments. | Background/Introduction:  Our body has two key systems: the nerve system and the defense system. The nerve system helps us feel things and move. The defense system fights off germs. These two systems also work together when the body gets hurt or sick. In people with cancer, these systems send signals to each other. This signal-sharing can help or hurt how the cancer grows. Nerve cells can enter tumors and change how defense cells act. The defense cells can also change how nerve cells work, like those that make us feel pain. This teamwork is what we call "nerve-defense talk" in cancer.  Methods:  Experts looked at what many older and new studies say about how nerve and defense systems affect cancer. They focused on how signals from nerve cells change what happens to tumors and defense cells near those tumors. Scientists also studied how this back-and-forth talking between cells might help or stop the tumor from growing. They checked what roles both nerve and defense cells play around the tumor.  Findings/Results:  The study found that nerve cells can give off certain body chemicals that reach the tumor. These chemicals can make defense cells weaker or tired. This makes it harder for the body to fight off the cancer. Some nerve cells also help defense cells that stop the defense system from working well. On the other hand, the defense cells inside the tumor can change how nerve cells near them act. Some of these nerve cells are the ones that help us feel pain. These talks between nerve and defense cells can make it easier for the tumor to grow.  Conclusion:  The body’s nerve and defense systems work together in more ways than we have known before. This two-way talk helps tumors grow or can stop the body from fighting the cancer well. If we understand this better, we may find new ways to help the body fight cancer by stopping or changing these signals. This may help people respond better to cancer care in the future.  Short Summary:  This study looks at how the body's nerve and defense systems talk to each other when someone has cancer. Nerve cells and defense cells send signals to one another that can help or hurt the fight against tumors. The nerve signals can weaken defense cells, and defense cells inside tumors can affect nerve cells too, especially those linked to pain. Finding new ways to stop these bad signals might help us care for people with cancer better. More studies will likely explore this in the future. | Background/Introduction:  Our brain and body defenses (called the nervous and immune systems) help us fight germs and fix damage. They talk to each other to help us heal. Experts now study how these two systems work together in cancer. It turns out, nerves in and around tumors can affect how our body fights back. In the same way, cells that fight cancer can send messages to nerves, and they might change how we feel pain. Some nerve signals can even block the body’s fight against cancer. This story is about how nerves and immune cells interact inside the areas near tumors. The goal is to learn how this talk between cells can help or hurt us when we treat cancer.  Methods:  The team looked at different studies done by many other scientists. They studied past tests and reports about how nerves and immune cells work in cancer. They paid close attention to changes inside and around tumors. The review focused on the ways that nerve signals and cancer-fighting cells interact.  Findings/Results:  The team found that nerves give off tiny messengers that can change how cancer and immune cells behave. Some of these messengers help cancer grow or weaken the body’s defense. Also, some cancer-fighting cells can change how nerves act, even those that feel pain. Other nerve types send signals that help block the body's defenses from working well. These complex talks between nerves and immune cells help shape how fast cancer grows or how well the body can fight it.  Conclusion:  Learning more about how nerves and immune cells work together can help us treat cancer better. If we can block harmful signals or boost helpful ones, doctors can make better tools to help the body fight cancer. These ideas may help improve how current cancer treatments work. Experts think more studies will be done in the future to find better ways to use this nerve-immune link in cancer care.  Short Summary:  This study looked at how nerves and body defense cells talk to each other when cancer is in the body. The aim was to see how this talk affects how cancer grows and how the body fights it. The review found that nerve signals can help or hurt the body’s defenses. The team hopes that by better understanding this, future treatments can help people fight cancer more strongly. Doctors may use this knowledge to make current treatments work even better, and more studies will likely happen next. | Background/Introduction  The brain and body’s germ fighters work together to keep people safe from harm. They help the body heal from cuts or bugs. These two parts — the nerve system and the germ-fighting system — can talk to each other and shape how sickness grows. When people have cancer, this talk between nerves and germ fighters can change how fast the cancer grows. New studies show how nerves in or near cancer spots send out tiny helpers that change how the cancer and germ-fighters act. Germ-fighters in cancer areas can also change how nerves work, even nerves that bring pain. This big teamwork plays a big role in how the body fights off cancer.   Methods  The writers of the study looked at many old and new papers. They found facts about how nerves and germ-fighters act inside and near cancer. They paid close care to nerves outside the brain and spine, since those seem to help or stop the body from fighting cancer.   Findings/Results  They found that nerves near cancer do more than send signals. These nerves release tiny helpers that can make germ fighters act in new ways. Some of these changes slow down the fight against cancer. Some germ fighters can even stop working right. Also, germ fighters can change nerve signals, even those that bring pain. The nerves that help the body deal with fear or stress can also make it harder for germ fighters to work well.   Conclusion  The body’s nerves and germ fighters both help control how cancer grows. This back-and-forth between them can help or hurt cancer care. By learning how they work together, doctors may find new ways to help the body fight cancer. This may also help other cancer care work better.   Short Summary  This study looks at how nerves and germ fighters work together in people with cancer. The goal was to learn how they help or hurt the fight against cancer. The team found that nerves near cancer send out stuff that can stop germ fighters from working right. At the same time, germ fighters can change how nerves act, even making pain feel different. These changes can slow down the body’s fight against cancer. The study shows how both teams shape what happens to cancer in the body. Knowing this could help doctors come up with new ways to treat cancer. More study is likely needed to find the best ways to use this new info. |
| NatRevCan | 5 | 10.1038/s41568-025-00829-4 | Pub2Post | Sex is a significant determinant of cancer incidence and outcome. The effects of sexual differentiation on normal and cancer biology underly this epidemiology. The resultant sex differences in therapeutic target pathways and processes provide a foundation for developing more personalized cancer treatments. However, our efforts at personalization cannot stop there. Humans also have gender, and sex and gender are highly interactive in individuation. Thus, we will also need to consider how gender-sex interactions (GSI) affect cancer biology and clinical parameters such as the timing of diagnoses, clinical trial enrolment, and the completeness of efficacy and toxicity data. Ignoring the effects of GSI can compromise the quality of basic biological and clinical data and the conclusions drawn from them. This is not to say that GSI will always have a significant effect or any effect at all in every cancer study. Rather, it is to say that we know enough about GSI and human cancer to anticipate measurable differences when GSI are considered in research, enabling us to experimentally determine whether their effects are significant. Here, I delve deeply into GSI and cancer, as this approach to treatment personalization holds great promise to benefit all patients with cancer. | **Background/Introduction**  Men and women often get different types of cancer and may respond in different ways to treatment. This happens in part because of how their bodies grow and develop differently. These body differences change how cancer starts and grows. These differences can also change how well cancer treatment works. But there’s more to people than just being male or female. People also have "gender," which means how they feel, act, and are treated in the world. Together, the way someone’s body is built and their gender can change when they see a doctor, if they get into a trial for a new treatment, and what kind of care they receive.  **Methods**  The writer looked at research about how body type (male or female) and gender interact and affect cancer. He dug deep into the differences that can change how cancer looks, how fast it grows, and how treatment works. The writer also looked into how these differences may be left out of studies and why that can be a problem.  **Findings/Results**  The study showed that both body type and gender can change cancer care and research in key ways. For example, it can affect when someone finds out they have cancer, how well they do in a trial, and how much help or harm they get from new drugs. Not paying attention to these differences can mess up results and slow down good care.  **Conclusion**  To help all people with cancer do better, both body type and gender must be looked at closely. These two things work together and may make a big change, or sometimes not much, but we should always check. Taking these into account can lead to better, fairer care and help science learn more from every study.  **Short Summary**  The study looked at how being male or female and one's gender identity work together to change cancer risks, results, and treatment. These two parts of a person can change when they find out they have cancer and how well they do with care. The author says we need to always think about both parts when studying cancer to make sure the science is fair and helps more people. This may lead to better care plans for each person. The writer thinks more work will follow this idea in future studies. | **Background/Introduction**  Boys and girls often get sick in different ways. This is true for cancer too. Some of the ways that cancer grows and spreads are not the same in boys and girls. These body changes start when we are kids and become more clear as we get older. These changes help doctors find better ways to treat cancer. But it is not only about being male or female. How someone sees and lives out their gender (like being a boy or girl in society) also plays a role. When doctors do not think about both body sex and lived gender, they can miss big clues.  **Methods**  The researcher looked at many past studies on cancer. They wanted to see if thinking about sex and gender together helped people understand cancer better. These ideas are called gender–sex interactions. The researcher looked at how these ideas may change how fast cancer is found, who joins a cancer study, or how well treatments work. The goal was to learn how these ideas can help doctors give the best care to each person.  **Findings/Results**  The review showed that both sex (body parts from birth) and gender (how someone lives their role in society) can change how cancer acts. These things can also change how strong a treatment is or how someone feels during treatment. Some cancers may be missed if doctors do not think about gender and sex together. Some people may get sicker than others or not even be asked to join a study. The data used in cancer work gets more useful when it includes this mix of gender and sex.  **Conclusion**  We need to think about both the body and the lived role of people when we treat cancer. If doctors use this full view, they may help more people. Not all cancers may show changes from gender–sex ideas. But researchers should keep them in mind to see if they make a big change. Looking at both sex and gender helps us treat each person in the best way. This can help all people with cancer have better care. More study in this area should be done.  **Short Summary**  Boys and girls show and feel cancer in different ways. This is not just about body parts from birth, but also how they live their gender. Past research shows these ideas matter when finding and treating cancer. If doctors use both in their plans, they can help more people. This study says we need to look at both for the best cancer care. Future studies should keep thinking about this. | **Background/Introduction**  Boys and girls can face different risks when it comes to getting and dealing with some types of cancer. These differences come from how our bodies grow and change with sex, which means being male or female from birth. This also changes how cancer behaves and how it can be treated in different people. On top of this, people have something called gender. This is how we feel or see ourselves, like as a man, woman, or something else. Gender and sex mix together in many ways. When this blend is not studied in cancer care, doctors may miss important facts and this may hurt the chance of helping patients the best way.   **Methods**  The writer looked closely at other studies and facts about cancer to see how sex and gender mix and change cancer. He wanted to find out how this mix could change things like how fast cancer is found, who joins cancer tests, and how much we know about drug effects. He shared ideas on how we can do better by looking at both sex and gender in cancer science.   **Findings/Results**  The report found that not looking at both sex and gender can cause wrong or weak ideas in cancer work. For example, if we study only men or only women, we might not spot all the side effects or know which drugs work best. Boys and girls may not even join drug tests in the same way. But if we do pay careful attention to gender and sex mix, we might be able to better tell who cancer may hit, how fast it grows, and which cure can work better.   **Conclusion**  So, it is very clear that both sex and gender work together when it comes to cancer. We should think about them both when we study cancer. If we do, we can help all people better, no matter who they are. Learning more in this way will give people the best chance to beat cancer. More study is still needed, but this can help improve care for everyone.   **Short Summary**  This research shows that cancer acts different in boys and girls. Part of this comes from how their bodies are made (called sex), and the other part comes from how they see their role in life (called gender). These two things often mix and affect how cancer starts and how well care works. If studies forget about this mix, they may miss key facts or give weak results. But if we do pay attention to both, care and tests can be better and fair for all. The writer says that more tests that include both ideas are needed to help all sick people in the right way. |
| CancerRes | 1 | 10.1158/0008-5472.CAN-24-3173 | Pub2Post | Perineural invasion (PNI) is a pivotal prognostic factor in pancreatic cancer, associated with aggressive tumor behavior and adverse patient outcomes. The recognized clinical impact of PNI highlights the need to better understand the molecular mechanisms underlying PNI-induced phenotypes. In this study, we isolated PNI-associated cancer-associated fibroblasts (pCAF), which demonstrated a markedly enhanced capacity to promote neural invasion in pancreatic cancer compared with non-PNI-associated CAFs. Single-cell, high-throughput sequencing and metabolomics data showed a significant upregulation of glycolysis in pCAFs, fostering a high-lactate tumor microenvironment conducive to cancer progression. pCAF-derived lactate was absorbed by tumor cells, facilitating histone H3K18 lactylation. The lactate-induced epigenetic modification activated the transcription of neural invasion-associated genes, such as L1CAM and SLIT1, thereby driving PNI in pancreatic cancer. Further exploration of metabolic reprogramming in pCAFs revealed enhanced acetylation of the glycolytic enzyme GAPDH, which correlated with increased enzymatic activity and glycolytic flux. Targeting GAPDH and lactylation modifications significantly inhibited neural invasion in a genetically engineered mouse model. Clinical data suggested that high levels of H3K18 lactylation correlate with severe PNI and poorer patient prognosis. Together, these findings provide critical insights into the role of CAFs in promoting PNI of pancreatic cancer, highlighting glycolytic reprogramming and lactate-driven histone modifications as potential therapeutic targets for PDAC.Significance: Targeting cancer-associated fibroblast metabolism or histone lactylation in pancreatic cancer cells to reverse epigenetic remodeling induced by lactate accumulation in the tumor microenvironment are potential therapeutic strategies to inhibit perineural invasion. | Background/Introduction:  Pancreatic cancer often spreads along tiny nerves inside the body. This makes the disease harder to treat and shortens lives. This nerve spread is called perineural invasion. The study looked at some helper cells called fibroblasts. These cells live in the tumor and help it grow. These fibroblasts were found to be more active in cases where the cancer spread through nerves. The goal was to better understand how these helper cells make the cancer worse.  Methods:  The study looked closely at the fibroblasts taken from people with pancreatic cancer. Some of these fibroblasts came from spots where cancer had spread into nerves, while others came from places where it had not. Researchers looked at how much sugar these cells used and what waste they made, including a type of acid called lactate. They also looked at how this lactate changed the cancer cells, even at the gene level. Tests were also done in lab animals.  Findings/Results:  The helper cells from nerve-spread areas made more lactate. This lactate changed how the cancer cells worked. It turned on certain parts of the genes that help cancer move into nerves. This showed that when fibroblasts make more lactate, they help the cancer invade the nerves. One small part of the cell that makes energy, called an enzyme, was also more active in these fibroblasts. When this part was stopped, the cancer could not spread into the nerves as much. In real people, when cells had more of this lactate change, they were sicker and had worse results.  Conclusion:  The fibroblasts play a big part in how pancreatic cancer grows into nerves. They do this by making too much lactate, which then changes cancer cell genes. If we block this process, it may help stop the nerve-spread of cancer. This gives a new way to think about treatment and more studies may follow.  Short Summary:  This study looked at people with pancreatic cancer. It found that helper cells called fibroblasts can make the cancer worse by helping it travel into nerves. These cells make a kind of acid waste called lactate, which changes how cancer genes work. When researchers blocked this change, the cancer slowed down. This points to a new way we might treat this type of cancer in the future. | Background/Introduction:  Doctors know that nerve spread in a certain type of gut cancer makes things much worse for people. But they still do not fully know how this spread happens. This study looked at a group of helper cells called cancer helper cells. These cells live near the cancer and were found more often around nerves in bad tumors. The goal was to find out how these cells help cancer move into nerves.  Methods:  The team took helper cells from cancer with nerve spread and studied them. They checked how these cells work by looking at all the tiny parts inside them. They also checked the sugar use and waste made by these cells. They wanted to see what the cancer eats and how it changes the body around it. They used both lab tests and mouse models to check what happens when they block some parts of this process.  Findings/Results:  The helper cells made more sugar go through a process that makes energy fast. This made a lot of waste called lactate, a type of acid. Cancer used this waste to change some key parts inside their cells. These changes helped the cancer move into nearby nerves. When the team blocked this, the cancer had more trouble reaching nerves. Mice with this type of cancer were better off when the team blocked some of the helper cells' work. Also, people who had worse nerve spread had more of this change inside their cancer cells.  Conclusion:  The study showed that helper cells feed the cancer with waste that makes it stronger and meaner. This waste tells the cancer how to spread into nerves. By stopping how the helper cells make this waste or by stopping how the cancer uses it, we might find new ways to fight the disease.  Short Summary:  This study explored how some helper cells in a gut cancer made a waste product that helped cancer spread to nearby nerves. These cells used more sugar and made a lot of this waste product. The cancer used it to grow and spread faster. The team found that blocking this sugar-use process or the way the cancer cells changed helped stop the cancer from spreading. People whose cancer cells used this waste more had worse health. More studies may help find better ways to treat or stop this type of cancer. | Background / Introduction  Some people with a type of cancer in the tummy called pancreatic cancer get worse when the cancer grows near or into nerves. This is called perineural invasion, or PNI. People with PNI often have more pain and do not live as long. Doctors want to know why PNI makes the cancer grow faster and spreads. This study looks at a special type of helper cell in the tumor, called a cancer helper fibroblast. These cells may make the cancer act more mean and hurtful to nerves.  Methods  The scientists looked closely at the cancer helper cells taken from tumors with and without nerve growth. They used new tools that look deep inside single cells to see how the cells work. They looked at how these cells use sugar for fuel and what happens when they make more of a waste called lactate. The team tested mice with cancer to see if stopping this process can help. They also checked results from people with this cancer to see if what they found in mice would match real patients.  Findings / Results  Cancer helper cells in tumors with nerve growth used more sugar and made more lactate. Lactate is a waste, like the sour stuff found in milk. The cancer cells took in the lactate. This changed how the cells acted by turning certain genes on. One gene, called L1CAM, helps cancer grow into nerves. When scientists blocked how cancer cells use lactate, the cancer stopped growing into nerves. In the mice, doing this worked well. In people, those who had more changes from the lactate in their cells had worse cancer and shorter lives.  Conclusion  Helper cells in a cancer tumor make the area around the cancer rich in lactate. This helps the cancer grow toward and into nerves. If we stop this from happening, we might stop the cancer from spreading in this way. This study gives hope for new ways to treat this problem. The team might study this again to see how well these ideas can help real people in the future.  Short Summary  Doctors studied why pancreatic cancer grows into nerves and makes people sicker. They found that helper cells in the cancer make a sour waste called lactate. This waste changes the cancer and makes it grow into nerves. When they blocked how the cancer uses this waste, the cancer did not spread to the nerves. This may help make new treatments for people with this type of cancer. More studies may be done to see how this could work in real life. |
| CancerRes | 2 | 10.1158/0008-5472.CAN-24-1879 | Pub2Post | Black individuals experience worse survival after a diagnosis of high-grade serous ovarian carcinoma (HGSC) than White individuals and are underrepresented in ovarian cancer research. To date, the understanding of the molecular and genomic heterogeneity of HGSC is based primarily on the evaluation of tumors from White individuals. In the present study, we performed whole-exome sequencing on HGSC samples from 211 Black patients to identify significantly mutated genes and characterize mutational signatures, assessing their distributions by gene expression subtypes. The occurrence and frequency of somatic mutations and signatures by self-reported race were compared with historic data from The Cancer Genome Atlas (TCGA). Despite technical differences (e.g., formalin-fixed vs. fresh-frozen tissue), the distribution of mutations and their variant classifications for major HGSC genes were nearly identical across study populations. However, de novo significantly mutated gene analysis identified genes not previously reported in TCGA analysis, including the oncogene KRAS and the potential tumor suppressor OBSCN. The prevalence of the homologous recombination deficiency signature was higher among Black individuals with the immunoreactive gene expression subtype compared with the mesenchymal and proliferative subtypes. These findings were confirmed by comparing the data from Black patients with those from 123 White patients with identical tissue collection and processing. Overall, this study suggests that, although most features of HGSC tumor phenotypes are similar in Black and White populations, there may be clinically relevant differences. If validated, these phenotypes may be important for clinical decision-making and would have been missed by characterizing tumors from White individuals only.Significance: Elucidation of the somatic mutational landscape of high-grade serous ovarian carcinoma in Black individuals, who experience poor survival and are underrepresented in research, could inform patient prognosis and enable precision medicine opportunities. | Background/Introduction:  Black women who get a type of ovary cancer often do not live as long as white women. These women have not been part of many past cancer studies. Most past studies looked only at white women. So, we do not know much about how this cancer acts in Black women. This study looked closely at this cancer in Black women to see if it is different in some way.  Methods:  Doctors studied small bits of tissue taken from over two hundred Black women who had this type of ovary cancer. They used a lab method called whole-exome sequencing. This test checks all the genes that can make proteins to find changes that might lead to cancer. They also looked at how certain genes were active and grouped the cancer types by these gene changes. They then compared the cancers in Black women with past data from white women.  Findings/Results:  Even though the cancer looked mostly the same in both groups, they did find some new things in the Black women’s samples. Some new genes had changes that had not been seen before. One gene helps cancer grow, another may stop cancer from growing. In one group of Black women, a certain type of gene damage was more common. This was not as common in other groups. The test results from both Black women and white women were checked and matched to be sure.  Conclusion:  The main parts of this type of ovary cancer are alike in both white and Black women. But there are a few small differences in Black women that could really matter for how doctors treat them. If doctors know this, it may help them pick better care or drugs. If we just look at white women’s samples, we might miss these points. More studies are likely needed to be sure.  Short Summary:  This study looked at a type of ovary cancer in more than two hundred Black women. Researchers used a lab test to check the genes and see how the cancer acts. Most cancer traits were the same across groups. But some gene changes were new and found only in Black women. One gene helps cancer grow, another may slow it. These new facts may help doctors treat cancer in Black women better. More work will help check these early facts. | Background/Introduction:  Black women with a certain kind of ovarian cancer often do not live as long as white women. But most cancer studies have not included many Black women. This means doctors may not have full facts to help treat them well. This study looked closely at the genes in cancer tumors from many Black women to learn more about how their tumors work and how they may be different from those in white women.  Methods:  The study checked the full set of genes in tumor samples from over two hundred Black women. The gene changes were looked at closely. The researchers compared the types of gene changes with past data from white women. They also grouped the tumors by how active their genes were and used the same lab methods to compare tumors from both groups.  Findings/Results:  Most of the gene changes seen in Black women were the same as in white women. But a few new gene changes were found in the tumors from Black women. One of these genes may help cancer grow faster, and another might help stop cancer, but only if it works right. One special kind of gene change linked to how the body fixes broken genes was found more often in tumors from Black women with a certain gene pattern. These results were checked by comparing the tumors from both groups using the same tools and ways.  Conclusion:  Even though tumors in both Black and white women look mostly the same, some small gene changes may still make a big difference. These small changes might help doctors choose better treatments for each person. If other studies find the same results, this could help build fairer and better ways to treat all women with this cancer.  Short Summary:  This study looked at tumors from many Black women with a certain deadly type of ovarian cancer. It found most tumors had the same gene changes as those in white women, but some new changes were seen only in the Black group. One gene that can help cancer grow and one that can stop cancer were found with differences. A special type of gene issue that causes problems with fixing broken genes was also higher in a smaller group of Black women. These findings may help doctors make better choices for care in the future. More work is needed to confirm these results. | Background/Introduction:  Black women who get a kind of tough ovarian cancer often do worse than White women. They are also not often part of cancer research. Most past research looked at tumors from White women, which means we may not know the full picture.  Methods:  In this study, doctors looked closely at the DNA from the tumors of over two hundred Black women. They wanted to see which genes were changed in bad ways. They also checked how these changes acted in different tumor types. Then they compared their results to old samples from White women to see if there were any big differences.  Findings/Results:  For the most part, the gene changes were the same in both Black and White women. But in the Black women’s tumors, some gene changes not seen before showed up. One of them helps cancer grow. Another one might stop cancer from growing. For one tumor type found in some Black women, certain gene marks that show the body’s ability to fix itself were more common.  Conclusion:  This study found that while tumors in both Black and White women look alike in many ways, there are some things found only in Black women. These new findings might help doctors choose better treatments in the future. If we had only looked at White women, we might have missed these key signs.  Short Summary:  This study looked at a tough type of ovarian cancer in Black women, a group often left out of research. It found that while most tumor traits are the same in Black and White women, Black women had a few new gene changes. Some of these might help us choose better care. These results show the need to study all groups, not just some. Future studies could help confirm this and guide doctors in choosing the best plans for each person. |
| CancerRes | 3 | 10.1158/0008-5472.CAN-24-1517 | Pub2Post | Early detection of lung cancer is important for improving patient survival rates. Liquid biopsy using whole-genome sequencing of cell-free DNA (cfDNA) offers a promising avenue for lung cancer screening, providing a potential alternative or complementary approach to current screening modalities. Here, we aimed to develop and validate an approach by integrating fragment and genomic features of cfDNA to enhance lung cancer detection accuracy across diverse populations. Deep learning-based classifiers were trained using comprehensive cfDNA fragmentomic features from participants in multi-institutional studies, including a Korean discovery dataset (218 patients with lung cancer and 2,559 controls), a Korean validation dataset (111 patients with lung cancer and 1,136 controls), and an independent Caucasian validation cohort (50 patients with lung cancer and 50 controls). In the discovery dataset, classifiers using fragment end motif by size, a feature that captures both fragment end motif and size profiles, outperformed standalone fragment end motif and fragment size classifiers, achieving an area under the curve (AUC) of 0.917. The ensemble classifier integrating fragment end motif by size and genomic coverage achieved an improved performance, with an AUC of 0.937. This performance extended to the Korean validation dataset and demonstrated ethnic generalizability in the Caucasian validation cohort. Overall, the development of a deep learning-based classifier integrating cfDNA fragmentomic and genomic features in this study highlights the potential for accurate lung cancer detection across diverse populations.Significance: Evaluating fragment-based features and genomic coverage in cell-free DNA offers an accurate lung cancer screening method, promising improvements in early cancer detection and addressing challenges associated with current screening methods. | Background/Introduction  Doctors need to find lung cancer very early to help people live longer and get better care. Some blood tests can find signs of cancer without needing surgery or scans. This study looked at tiny bits of DNA that float in the blood. DNA holds the body’s code. When cells die, they drop pieces of DNA into the blood. Cancer cells drop different kinds of DNA pieces. The team wanted to try a new way to spot these cancer DNA bits better.   Methods  The team studied over a few thousand people, some with lung cancer and some without. They used a computer system that learns from large sets of data. This system looked at the size and ends of the DNA pieces. It also looked at which parts of the DNA showed up more or less often in these pieces. The team tested this on people from Korea and another group with light skin color from a separate country to make sure the test worked for many groups.   Findings/Results  The new way to spot cancer worked better than older ones that only looked at part of the DNA bits. When they used both size and end shape of the DNA along with which parts of the body’s code showed up, they found more cancers earlier and more correctly. It worked well on both groups of people, no matter where they are from or the color of their skin.   Conclusion  The new test using bits of DNA in the blood seems to be a better and easier way to find lung cancer early. It picked out sick people from healthy ones well, even with different skin colors and places they live. This may help make cancer care better and faster. More follow-up work could help make this blood test something doctors use often.   Short Summary  This study worked on a better way to find lung cancer early using blood instead of scans or surgery. It looked at tiny DNA pieces from dead cells floating in blood. People with and without cancer gave blood for the test. A smart computer learned from the shapes and sizes of the DNA and where the code came from. This method found cancer better than older ways and worked well in people with different skin color and from different places. This could help doctors find cancer sooner and help people faster. More work is likely to be done on this method. | Background/Introduction:  Lung cancer is a sickness that makes it hard for people to get well if found too late. Doctors want to find it early to help people live longer. Right now, tests for lung cancer are not always easy or right. A new way to look for cancer uses tiny bits of DNA – genetic code – that float in the blood. These bits come from cells and can help show if someone has cancer without needing to cut or poke the body. This study looked at how different parts and sizes of these blood DNA bits could help find lung cancer.  Methods:  The researchers tested samples from people in two countries. They used special computer tools that learn from data, kind of like how voice assistants learn from talking. These tools looked at the ends and sizes of the DNA bits. The goal was to see if these tools could tell the difference between people with lung cancer and people without it. People from Korea and people of white skin from other places joined this study.  Findings/Results:  The study found that the computer tools using both the size and the ends of the DNA bits worked better than just using one part. These tools were good at finding lung cancer in Korean people and also in white people from other places. This means the test works well for people from different backgrounds.  Conclusion:  This new test that checks blood for lung cancer using DNA bits and smart computer tools might help find cancer early. It works for many types of people and might be better than older tests. More checks may be done in the future to make this test even better.  Short Summary:  Doctors want to find lung cancer early to help patients live longer. This study used a smart computer tool to look at tiny pieces of DNA in blood. These pieces come from cells and can help show if a person has cancer. The test worked well in both Korean people and people with white skin. It may offer a better, easier way to check for lung cancer without using hard or painful tests. More work might be done to improve it. | Background/Introduction:  Finding lung cancer early helps more people survive. Doctors now test blood using a method called “liquid biopsy.” This checks for small bits of DNA in blood. DNA is a code carried by all the cells in our body. When we get sick with cancer, tiny bits of DNA float in the blood. This study looked at how the shape and size of those DNA bits can help find lung cancer early and better.  Methods:  The study made a new computer tool. This tool looked at the DNA bits in blood from people with and without lung cancer. It used a smart system called "deep learning" to find patterns. The tool was tested using blood from people in different groups, including people living in one country in Asia and people with European background.  Findings/Results:  The study found that using both the shape and size of the DNA bits gave better results than using each part by itself. The tool worked well for people in both groups. The tool found those with lung cancer more often and more correctly. It worked the best when it used both shape and size with extra gene-related parts.  Conclusion:  This new tool can help find lung cancer from blood better than before. It works for people from different countries. It may help more people get care sooner and live longer. More studies can help learn more and make this idea even better.  Short Summary:  This study tested a new way to find lung cancer early using blood tests. It looked at the shape and size of tiny DNA bits in blood. A smart computer tool read these patterns. It worked well on many kinds of people, not just one group. This new test found people with lung cancer better than older tests. The tool may help people from all places get help faster. More work on this tool is planned. |
| CancerRes | 4 | 10.1158/0008-5472.CAN-25-0987 | Pub2Post | Therapy resistance is a significant cause of death in patients treated with targeted cancer therapy in diverse oncogene-driven cancers. A better understanding of resistance mechanisms can lay the foundation for improving existing and developing new therapies. A recent elegant study published in Nature Chemical Biology sheds light on a new resistance mechanism. The authors define a novel role for ARAF, a member of the RAF protein family (ARAF, BRAF, and CRAF), that is distinct from its previously understood role as a RAS effector and MEK protein kinase in the MAPK pathway. They describe how ARAF sequesters active RAS at the plasma membrane in phase-separated condensates to sustain signaling and prevent inactivation by the RAS GTPase-activating protein neurofibromin 1. This study underscores emerging roles for biomolecular condensates in cancer and highlights important implications for disrupting protein condensates to address treatment resistance to RAS (and RAS pathway)-targeted therapies. The study also illuminates evolutionary functional distinction between the RAF proteins and indicates unique biology for ARAF in normal physiology and disease. | Background/Introduction:  Some people with cancer get medicine that targets certain bad parts of their cells. But after some time, these medicines stop working. This makes it harder to treat the cancer. A study found a new reason why the medicine may stop working. This may help make better cancer care in the future.  Methods:  The study looked at a part inside the cell called ARAF. This part is from a family of cell helpers used in sending cell messages. ARAF does more than we thought before. The team found that ARAF keeps a bad protein, called RAS, busy by trapping it near the cell wall inside small blobs. These blobs stop RAS from being turned off by another helper, called neurofibromin. They used lab tools to see this happen in cells.  Findings/Results:  The study showed that ARAF traps the RAS protein and keeps it turned on. The small blobs in the cell help with this. When this happens, cancer can keep growing even with medicine. This can explain why some cancer drugs stop working over time. Also, ARAF works in its own special way that is not like the others in its group.  Conclusion:  This study found that small blobs inside cells, called condensates, play a big part in how cancer fights back against medicine. ARAF has a big job in this. This new finding may help doctors and scientists make new medicines that work better. It also shows ARAF may act in new ways in both sick and healthy cells.  Short Summary:  Some people with cancer do not get better with their medicine because the cancer finds ways to fight back. This study found that a cell part named ARAF helps a bad protein, RAS, stay turned on by trapping it in tiny blobs near the cell wall. These blobs stop RAS from being shut down. This helps the cancer grow even when treated. These new facts may help in creating better care that still works even when the cancer changes. | **Background/Introduction:**  When people get sick with cancer, they may get special medicine made just for their type of cancer. But sometimes, the cancer stops listening to the medicine and starts to grow again. Scientists wanted to know why this happens. A new study found something special that may help explain it. They looked at a protein called ARAF, which most folks have never heard of. This protein is in the same family as others that help control how cells grow. Before, scientists thought ARAF only acted in one way. But now they see it can do something new that may help cancer fight off medicine.  **Methods:**  The researchers used science tools to look closely at how ARAF moves and acts in cells. They found that ARAF makes sticky spots, like tiny clumps, in a part of the cell close to the outside edge. These clumps trap another protein called RAS in a very active state. RAS helps cells grow, and when it's stuck like this, it keeps sending strong grow signals. The team also looked at how these clumps stop a cell helper named neurofibromin 1 from calming RAS down.  **Findings/Results:**  The researchers saw that ARAF holds RAS in these sticky clumps to keep it awake and working all the time. This stops medicine from turning off RAS like it's supposed to. This means that even if a person gets the right medicine, the cancer can still grow because RAS never really turns off. These clumps are called "condensates." They may be one key reason why cancer fights off drugs that target RAS.   **Conclusion:**  This study shows that these small clumps in the cell are a big reason why cancer medicine stops working. ARAF is not just a helper for RAS but also keeps it going in a new way. This new idea about how cells act may help doctors figure out better ways to stop cancer from fighting back. The study shows that breaking up these clumps may be a smart way to make medicine work better.  **Short Summary:**  Doctors give special drugs to stop cancer, but sometimes they stop working. A new study found that a protein named ARAF helps cancer keep growing by trapping another protein called RAS in small clumps inside cells. These clumps help RAS stay very active and ignore the medicine. The study shows that ARAF plays a bigger role in cancer than once thought. This can help scientists make better drugs in the future. | **Background/Introduction**  Sometimes, cancer drugs stop working. This happens in many people with cancers caused by a certain group of broken genes. Learning why this happens can help doctors make better drugs. A new study looked at one of the body’s helper proteins called ARAF. This protein is part of a family that helps send messages inside cells. Before now, scientists thought ARAF only had one job. But this study shows it may do something new.  **Methods**  Scientists used lab research and special tests to watch what ARAF does in cells. They looked at how it behaves with another group called RAS, which turns cancer on. RAS normally gets turned off by another helper. But ARAF was seen keeping RAS turned on by holding it at the edge of the cell.  **Findings/Results**  The scientists saw that ARAF pulls RAS into small liquid areas in the cell's edge. These tiny areas are like bubbles where RAS can stay active. This keeps RAS from being shut down by another helper protein. Because RAS stays on, some cancers grow, and some drugs do not work anymore.  **Conclusion**  This study shows that ARAF has a new role that helps cancer grow by keeping RAS switched on. This helps explain why some people do not get better with certain cancer drugs. The study says that breaking up these tiny bubbles might help new drugs work better. This finding also shows that ARAF works in a special way that is different from other helper proteins like it. Future studies may look at how to stop ARAF from helping cancer grow.  **Short Summary**  Cancer drugs sometimes stop working. A study found that a helper protein called ARAF keeps cancer turned on by holding another protein, called RAS, in tiny bubble-like places in cells. This stops RAS from being shut off, which can lead to cancer drug failure. The study helps show why this happens and gives ideas for new ways to treat cancer. Future research may try to stop these cell bubbles to help cancer drugs work again. |
| CancerRes | 5 | 10.1158/0008-5472.CAN-24-2289 | Pub2Post | Non-coding RNA transcribed from active enhancers, known as enhancer RNA (eRNA), is a critical element in gene regulation with a highly specific expression pattern in the regulatory networks of tumor-infiltrating cells. Therefore, eRNA signatures could potentially be applied to represent antitumor immune cells and to improve cancer immunotherapy. In this study, we identified thousands of eRNAs that were significantly correlated with infiltrating immune cell abundance in more than 10,000 patient samples across a variety of cancer types. The expression of these eRNAs was mediated by transcription factors with high expression in antitumor immune cells, as identified through single-cell assays. An eRNA immunotherapy signature (eRIS) developed using the antitumor eRNAs was highly associated with the objective response rate of immunotherapy and was elevated in patients who benefited from immune checkpoint blockade treatment. In comparison with a signature based on protein-coding genes, the eRIS was more effective in predicting the response to immunotherapy. Integration of the eRIS with pharmacogenomic data revealed hundreds of anticancer drugs that have the potential to enhance immunotherapy efficacy. Finally, treatment of a mouse model of IDH-mutant glioma with the histone deacetylase inhibitor vorinostat improved the effects of anti-PD-1 immunotherapy through increased abundance of infiltrating immune cells. Taken together, this study developed an eRIS with demonstrated efficacy in predicting immunotherapy response and used the eRIS to identify a series of effective combination drugs, thus highlighting the clinical utility of the eRIS in immunotherapy enhancement.Significance: An eRNA immunotherapy signature developed using antitumor eRNAs in tumor-infiltrating immune cells improves the prediction of response to immunotherapy and identifies a series of effective drug combinations to enhance immunotherapy efficacy. | Background/Introduction:  Tiny pieces of RNA, called enhancer RNA, help cells turn certain genes on or off. These pieces show up a lot in certain cells found inside tumors. Scientists think these RNA pieces might help tell doctors which patients will do well with cancer treatment that uses the body’s own immune system. That's why this study looked at how these RNA pieces are linked to immune cells in tumors and how they might help find better ways to treat cancer.  Methods:  The researchers looked at RNA from many patients with different kinds of cancer. They studied samples from over ten thousand people. They wanted to find which RNA pieces were in immune cells that try to fight tumors. They used special tools to see which genes were active in single cells. Then, they created a group of RNA pieces tied to good immune responses. They called this group an RNA signature. The team also checked how well this RNA group matched patient responses to cancer treatment. Finally, they tested one treatment mix in mice to see if it helped immune cells fight brain tumors.  Findings/Results:  The team found many RNA pieces that showed up more when there were more immune cells in tumors. These pieces only showed up when certain helper parts in the cell (called transcription factors) were working. When patients’ tumors had more of these special RNA pieces, they were more likely to get better from immune-based treatment. This new RNA group was better at guessing who would get better than other gene tests. The team also saw that many cancer drugs might work better when used with immune therapy. In mice with brain cancer, adding one drug made the immune treatment work much better.  Conclusion:  The new RNA group helps tell which patients might do well with immune-based cancer treatment. It can also help find drug pairs that fight cancer better together. This could help doctors treat cancer in a smarter way. More research may look at how else this RNA group can help improve cancer care.  Short Summary:  Scientists studied small RNA pieces that help turn genes on inside cancer-fighting cells. They found that patients with more of these RNA pieces in their tumors were more likely to get better from immune-based cancer treatments. They also found that these RNA pieces can help pick better drug combos. In mice, one drug made immune treatment work better. This new RNA group might help doctors decide who should get certain treatments and which drugs to mix together. More studies might be done to learn more. | Background/Introduction:  Some parts of our body, called cells, can become sick and grow into a lump called a tumor. These tumors can have special helpers called immune cells that try to fight the sickness. There is a kind of message in our body called enhancer RNA — it helps turn genes on or off. Scientists found that this message is common when immune cells are trying to fight cancer. So, they thought that watching these messages might help us learn more about these immune cells and if a cancer patient will get better with a type of medicine called immunotherapy. Immunotherapy is a way to help our body’s soldiers (immune cells) fight disease better.   Methods:  The scientists looked at many sick people with different kinds of cancer. They studied the messages made by their cells, especially the enhancer RNA. They checked which ones were strong when good immune cells were found in the tumor. They also used special tools that check what each tiny cell is doing, one at a time. A new list — called enhancer RNA immunotherapy signature — was made using the messages that showed strong work by the immune cells. They also matched this list with many different cancer medicines to find ones that could help immunotherapy work better. Then they tested one of the medicines in mice with brain sickness like human cancer.   Findings/Results:  The scientists found that many enhancer RNA messages were stronger when many helpful immune cells were in the tumor. These strong messages were turned on by special tools in the cells called transcription factors. Patients who got better with immunotherapy had more of these messages. The new enhancer RNA list helped tell which people would get better with treatment, even better than using other gene lists. When they used this RNA list to test many cancer drugs, they found many that may help immunotherapy work even better. One of these, called vorinostat, helped sick mice get better when used with another drug that helps the immune cells.  Conclusion:  The scientists made a useful list of RNA messages that showed who might get better from immunotherapy. This list also helped find new medicine pairs that can help our body fight cancer more strongly. The study shows this could help make treatment better soon, and more studies may happen to test more medicines with this idea.  Short Summary:  The study looked at signs in cells, called enhancer RNA, that help guess if cancer patients will get better with immunotherapy. By checking more than ten thousand people, the scientists made a helpful list of these signs. This list worked better than old tools to pick who may do well with treatment. It also helped find many new medicine partners that might help the body fight cancer. They tried one of these in mice, and it worked well. The results are hopeful for new ways to beat cancer, and more tests will likely be done. | Background/Introduction:  Some parts of our body's cells make tiny pieces of genetic material called enhancer RNA, or eRNA. These come from special parts of our genes and help turn other genes on or off. In people with cancer, these eRNAs can tell us how active the immune system is inside a tumor. That makes them useful for finding out whether cancer treatment that helps the immune system work better might be helpful.   Methods:  The scientists looked at many thousands of samples from people with different types of cancer. They searched for eRNAs that show up when there are lots of immune cells in tumors. They then checked which “switches” in the cell were helping turn these eRNAs on. Using this, they built a guide called the eRNA immunotherapy signature, or eRIS. This helped them guess which people might respond well to cancer treatments that help the immune system. They also used cell data to match drugs that could make these treatments work better.   Findings/Results:  The study found many eRNAs linked with strong immune responses in tumors. The eRIS guide worked better than older tools to guess who would benefit from treatment that helps the immune system fight cancer. The guide also pointed to many drug options that could make the treatments work even better. When the scientists gave one of these drugs, called vorinostat, to mice with brain tumors along with immune treatment, the mice got better results.   Conclusion:  The scientists made a helpful guide using eRNAs to tell who may benefit from immune-based cancer treatment. They also found drugs that could help make the treatment work better. This shows the eRIS guide may help find better ways to treat cancer in people.   Short Summary:  The study looked at small gene helpers called eRNAs found in the immune cells near tumors. These helpers can identify which people might get better from immune-based cancer treatments. The team made a new tool, the eRIS, from these helpers to better guess treatment results. This new tool worked better than older ones. It also helped them find drugs that could improve these treatments. One of these drugs helped mice with brain cancer. The study shows this tool may help doctors choose the best treatments for people with cancer and find treatments that work even better when mixed with others. More studies may happen in the future. |
| CancerRes | 1 | 10.1016/S1470-2045(25)00211-6 | Pub2Post | Background Oestrogen plus progestin hormone therapy is an established risk factor for breast cancer in postmenopausal women. We examined the less well-studied association between exogenous hormones and breast cancer in young women, who might use hormone therapy after gynaecological surgery or to relieve perimenopausal symptoms. Methods In this pooled cohort analysis, we investigated the relationship between exogenous hormones and breast cancer in young women using data from 10-13 prospective cohorts from North America, Europe, Asia, and Australia. The participating cohorts followed up women for incident breast cancer until age 55 years. We used cohort-stratified, multivariable-adjusted Cox proportional hazards regression to estimate hazard ratios (HRs) and 95% CI for associations of hormone therapy with incident young-onset breast cancer. We also estimated risk differences based on cumulative risk until age 55 years. Findings We included 459 476 women aged 16-54 years (mean 42 center dot 0 years [IQR 35 center dot 5-49 center dot 2]), of whom 8455 (2%) developed young-onset breast cancer (diagnosed before age 55 years; median follow-up 78 years [5 center dot 2-11 center dot 2]). Overall, 15% of participants reported using hormone therapy, with oestrogen plus progestin hormone therapy (6%) and unopposed oestrogen (5%) being the most common types. Cumulative risk of young-onset breast cancer was 41% in non-users. Hormone therapy of any type was not associated with incident young-onset breast cancer (HR 096 [95% CI 088 to 104]), but ever oestrogen hormone therapy use was inversely associated (086 [075 to 098]; risk difference-05% [-10 to-00]). The HR for ever oestrogen plus progestin hormone therapy and young-onset breast cancer was 110 (098 to 124), with positive associations observed for long-term use (118 [101 to 138] for >2 years) and use among women without hysterectomy or bilateral oophorectomy (115 [102 to 131]). Oestrogen hormone therapy and young-onset breast cancer association was similar for all breast cancer subtypes, but oestrogen plus progestin hormone therapy was more strongly associated with oestrogen receptor negative (144 [111 to 188]) and triple-negative disease (150 [102 to 220]) than with other subtypes. Interpretation Oestrogen hormone therapy use was inversely associated with young-onset breast cancer, and oestrogen plus progestin hormone therapy was associated with higher young-onset breast cancer incidence among women with intact uterus and ovaries. These findings largely parallel results from studies of hormone use and later-onset breast cancer and provide novel evidence for establishing clinical recommendations among younger women. | Background/Introduction:  Doctors know that some older women who take hormone pills after menopause face a higher chance of getting breast cancer. But little is known about how these hormone pills affect younger women. Some younger women take these hormones because they had surgery or have signs of early menopause. This study looked at how these pills might be linked to breast cancer in younger women.  Methods:  The study collected health data from many groups of women from across the world, including North America, Europe, Asia, and Australia. These women were followed until they were in their early fifties. The study grouped the women by where they were from and looked at those who had used hormone pills. The two kinds of pills studied were those that had both oestrogen and progestin, and those with only oestrogen. The study compared how many women got breast cancer in each group.  Findings/Results:  Almost half a million women took part. Most were in their early forties when the study began. A small number of these women got breast cancer before they turned fifty-five. About one in seven had used hormone pills. When researchers checked, they found that using any hormone pill did not clearly raise the chance of getting breast cancer for younger women. In fact, women who used pills with only oestrogen may have had a slightly lower chance. But for women who used pills with both oestrogen and progestin for a long time, or who still had their womb and ovaries, the chance of getting breast cancer may have gone up. This was more true for certain types of breast cancer that are harder to treat.  Conclusion:  Hormone pills with only oestrogen did not lead to more breast cancer in younger women and may even lower the risk. But pills with both oestrogen and progestin may raise the chance of breast cancer, mostly in women who have not had surgery to remove their womb or ovaries. These results are close to what past studies found in older women. The new information may help doctors give better advice to younger women who are thinking about taking hormone pills.  Short Summary:  The study looked at how hormone pills affect breast cancer in younger women. These pills are sometimes used after surgery or to ease signs of early menopause. The study followed many women from around the world. It found that oestrogen pills alone did not raise the risk of breast cancer, and may even lower it. But using both oestrogen and progestin, especially for a long time or in women with a womb and ovaries, may raise the risk. This gives doctors a better idea of who should or should not use these pills. More studies may be done in the future. | Background/Introduction:  Doctors know that some older women who take hormones after menopause may have a higher chance of getting breast cancer. But it is less known how taking these hormones might affect younger women who sometimes take them after surgery or when their bodies begin to change before menopause. This study looked into how these hormones might be linked to breast cancer in younger women.  Methods:  The study used past health information from groups of women in different parts of the world, such as North America, Europe, Asia, and Australia. All the women were followed until they were about middle age. The study checked whether using hormones increased their chance of breast cancer. Two kinds of hormone treatments were checked: one with two hormone types, called estrogen and progestin, and one with only estrogen.  Findings/Results:  The study included almost half a million women who were between their teen years and middle age. A small part of these women got breast cancer while they were still young. Around a small share of them had used hormone treatments, and two common types were most used: one that had both estrogen and progestin, and one with only estrogen. The study found that using only estrogen might lower the chance of getting breast cancer. But taking both estrogen and progestin might raise the chance, especially for women who still had their womb and ovaries. Women who used both hormones for a long time had a higher risk. This risk was stronger for certain types of breast cancer that grow faster and are harder to treat.  Conclusion:  Young women who used only estrogen had a slightly lower risk of getting breast cancer. But those who used both estrogen and progestin had a higher chance, especially if they had not had surgery to remove their womb or ovaries. These results are alike to past studies in older women, and they may help doctors give better advice to younger women thinking about using hormone pills. More research may be needed to confirm these findings.  Short Summary:  This study looked at younger women who used hormone treatments, often for signs of early menopause or after surgery. It found that only-estrogen treatment may lower their breast cancer risk, while using both estrogen and progestin may raise the risk, mostly in women who still have their womb and ovaries. Women who used both hormones for a long time were more likely to get fast-growing types of breast cancer. These findings could help guide how doctors talk with younger women about hormone use. More studies may happen in the future to learn more. | Background/Introduction:  Most people know that taking hormone medicine after menopause can raise the risk of breast cancer. But we do not know as much about how this medicine may affect younger women. These young women may take hormones for health reasons, like dealing with signs that come before menopause or after surgery to remove female parts. This study wanted to see how taking these hormones might link to breast cancer in these younger women.  Methods:  The study used health data from large groups of women living in places like North America, Europe, Asia, and Australia. The women were followed to see if they got breast cancer before they turned middle-aged. The study looked at women who took hormone medicine and those who did not. It compared their chances of getting breast cancer. The study looked at the kind of hormones used and how long they were taken.  Findings/Results:  The study included many women between their mid-teens and early fifties. Most of them were in their early forties. A few of them got breast cancer while they were still young. Only a small part of the group took hormone medicine. Two kinds of hormone medicine were most common. One was a mix of two female body hormones, and the other had just one. Women who never took the medicine had a small chance of getting breast cancer, but it was still a risk. The study did not show a strong link between taking either kind of hormone medicine and getting breast cancer early. But there was a slight lower risk in those who used the single hormone only. The study showed that using the mix of hormones for a long time or using it with a working womb and ovaries may raise the risk. The mix type was more linked to harder-to-treat types of breast cancer.  Conclusion:  Using the one-hormone type may lower a young woman’s chance of getting breast cancer. Taking the two-hormone mix, however, may raise the chance of getting breast cancer—especially if taken for a long time or if the woman still has her womb and ovaries. These results are much like what doctors already know about older women and hormone use. This new info can help doctors guide younger women better. More studies may be done later to learn more.  Short Summary:  This study looked at younger women who took hormone medicine, often for health reasons. Some used a mix of two female hormones, while others used just one. The study found no big link between these hormones and getting breast cancer early, but using just one hormone may lower the risk a little. Using the mix of two may raise the risk, mostly for certain breast cancer types and when taken for a long time. Doctors may use this new info when helping younger women make safe choices. More studies may come in the future. |
| CancerRes | 2 | 10.1016/S1470-2045(25)00139-1 | Pub2Post | Background Cell-free, circulating tumour DNA (ctDNA) is an established measure of minimal residual disease; however, it is not utilised in melanoma management. We investigated whether ctDNA measurements could predict survival outcomes during adjuvant targeted therapy or placebo treatment in stage III melanoma, thereby identifying patients at high risk and low risk of recurrence. Methods Analytically validated mutation-specific droplet digital PCR assays were used to measure BRAF(V600E) or BRAF(V600K) ctDNA in patients aged 18 years or older who were enrolled in the COMBI-AD trial, which was a double-blind, randomised, phase 3 study of oral dabrafenib (150 mg twice daily) plus oral trametinib (2 mg once daily) combination therapy versus two matched placebos in resected BRAF(V600)-mutant stage III melanoma. Patients were screened for enrolment between Jan 31, 2013, and Dec 11, 2014, had an Eastern Cooperative Oncology Group performance status of 0 or 1, and were randomly assigned (1:1) to the two treatment groups. The primary endpoint was recurrence-free survival, and the results from final analysis have been previously published and will not be described here. Biomarker analysis was a prespecified exploratory endpoint and performed in the intention-to-treat population. We compared associations between survival outcomes and baseline (post-resection) ctDNA copies per mL, tumour mutational burden and interferon gamma (IFNG) gene expression. In a subset of patients, ctDNA quantities during follow-up or at recurrence were measured. The trial is registered with ClinicalTrials.gov, NCT01682083, and has been completed. Findings Baseline plasma samples were available for 597 of 870 patients (331 male patients and 266 female patients) and samples for assessing the ctDNA positivity rate at landmark follow-up timepoints of 3 months, 6 months, 9 months, and 12 months after treatment initiation were available for 94 of 870 patients. Additionally, samples were available from 118 of 870 patients within a 2-month timeframe before or after clinical or radiographic recurrence. Median follow-up for the biomarker analyses was 60 months (IQR 39-66) in the combination therapy group and 58 months (21-66) for the placebo group. ctDNA was detectable in 79 (13%) of 597 baseline samples. ctDNA positivity rate and mutant copies per mL plasma were significantly higher in patients with higher disease substages. As a binary variable, ctDNA detection was associated with worse recurrence-free survival (placebo group: median 371 months [95% CI 239-689] vs 2441 months [1728-4313]; hazard ratio [HR] 291 [95% CI 199-425], p<00001); combination therapy group: median 1659 months [95% CI 1202-2680] vs 6811 months [5036-not reached]; HR 298 [195-454], p<00001) and overall survival (placebo group: median 3390 months [1396-not reached] vs not reached; HR 335 [201-555], p<00001); combination therapy group: median 4031 months [2490-not reached] vs not reached; HR 427 [250-727], p<00001) in the placebo group and combination therapy groups. Baseline ctDNA was more strongly associated with survival outcomes than IFNG gene expression or tumour mutational burden. Patients with adverse longitudinal ctDNA kinetics (molecular relapse or persistently positive) had markedly shorter median recurrence-free survival (831 months [95% CI 539-1220] and 532 months [279-not reached], respectively) compared with patients with favourable kinetics (ie, undetectable after positive baseline result: 1925 months [1639-not reached]; and durable undetectable: not reached [3844-not reached], p<00001). Interpretation Droplet digital PCR measurements of ctDNA to assess minimal residual disease before adjuvant targeted therapy and during follow-up can identify patients at high risk of early recurrence. Additional studies using ctDNA measurements to guide therapeutic interventions might lead to improvements in the management of resected stage III melanoma. | Background/Introduction:  Doctors know that when cancer is cut out, tiny bits can stay in the blood. These are called bits of tumour DNA. This study looked at people with skin cancer (called melanoma) to see if these tiny bits can help tell who might get sick again after treatment.   Methods:  The study included people with a type of skin cancer that had been taken out with surgery. Everyone took pills or fake pills, but no one knew which one they got. The study checked the blood for the tumour bits using a very tiny test (kind of like looking for a needle in a haystack). The blood was checked before and after treatment.   Findings/Results:  Not everyone had the tumour bits in their blood. The ones who did were more likely to get sick again sooner. People with fewer or no tumour bits stayed well much longer. Doctors saw that these tests were better at showing risk than some other cancer clues.   Conclusion:  Looking at tumour bits in the blood can help spot folks more likely to get sick again after skin cancer removal. This test may help doctors plan better care. More tests like this could help people stay well for longer.   Short Summary:  This study checked if tiny cancer bits in the blood could show who might get sick again after skin cancer was taken out. People with more of these bits were more likely to have cancer return. The test helped doctors see who needed closer care. More studies may help doctors use this tool to better treat skin cancer in the future. | Background/Introduction:  People with a type of skin cancer called stage three melanoma often have surgery to take out the tumor. However, the cancer can come back later. This study looked at a tiny piece of tumor material in the blood, called tumor DNA, to see if it could show whether the cancer might come back. These pieces are very small and float in the blood after cancer surgery. They might help doctors tell who needs more help to stop the cancer from returning.  Methods:  People with stage three melanoma who had a certain gene change (called BRAFV600) joined this study. Everyone got either a mix of two pills or a pretend treatment. Both groups were checked for tumor DNA in their blood after surgery. Doctors looked to see if this blood test could help guess who was more likely to stay cancer-free. They also looked at other things in the body, like how active a certain defense gene was. Some people in the study gave more blood samples after the start to help learn more.  Findings/Results:  Some blood from just over half of the people was good enough to study. A small group had tumor DNA still in their blood after the tumor was taken out. These people were more likely to have the cancer come back sooner. This was true even if they got the real medicine. If the small tumor pieces stayed in the blood over time, the chance of the cancer coming back went up. People who had no tumor bits left in their blood after starting the medicine had much better outcomes. This blood test gave better clues about what might happen than other ways of checking.  Conclusion:  Finding these tiny cancer pieces in the blood after surgery can help show who might have the cancer come back. This test may help doctors decide who needs more care. Future studies may see if using this blood test can help choose the best way to treat the cancer.  Short Summary:  This study looked at people with skin cancer who had surgery. Doctors used a blood test to look for small bits of cancer left in the blood. If these pieces stayed in the blood, the cancer was more likely to come back. People who had no tumor bits after starting medicine did much better. The test was better than other checks and may help pick the right care in the future. This could lead to better ways to help people stay free of cancer longer. Future research is likely to focus on this test. | Background/Introduction:  When people have skin cancer called melanoma, doctors try to remove all of it with surgery. But even after surgery, some tiny bits of cancer might still stay in the body. These small pieces are too small to see and can grow again later. Scientists call this "minimal residual disease." In this study, the team wanted to know if a blood test could help spot people whose cancer might come back. The blood test looks for tiny bits of cancer DNA floating in the blood.  Methods:  Adults who had their melanoma removed took part in a big research trial. They were split into two groups. One group got two cancer drugs. The other group got fake pills. The study used a test called droplet digital PCR. This test looked for a change in the cancer DNA called BRAFV600. Blood was tested before and after treatment to check if this changed DNA was in the blood. Doctors watched to see how long people stayed cancer-free. They also looked at how long they lived.  Findings/Results:  Some people had this cancer DNA in their blood before the treatment started. These people were more likely to have their cancer come back fast. Others did not have the cancer DNA in their blood and did better. Those who had the DNA but it went away after treatment stayed cancer-free longer. If the DNA stayed or came back later, cancer returned sooner. This blood test did a better job of showing who might stay well than other tests doctors often use.  Conclusion:  This simple blood test may help doctors know who might get sick again. If they know early, they can try other treatments or watch those patients more closely. More research may help test if using the blood test to guide treatment will help people with this type of cancer.  Short Summary:  This study looked at people with a type of skin cancer who had surgery to remove it. Some got cancer pills and some did not. A special blood test looked for tiny signs of cancer that might come back. People who had these signs in their blood were more likely to get sick again. The test seems to show who might be at risk. Using this test could help doctors treat people better after surgery. More studies are needed to see what changes to treatment might help most. |
| CancerRes | 3 | 10.1016/S1470-2045(24)00373-5 | Pub2Post | Background CAPItello-291 is an ongoing phase 3 trial in which capivasertib-fulvestrant significantly improved progression-free survival versus placebo-fulvestrant in patients with hormone receptor-positive, HER2-negative advanced breast cancer who had relapse or disease progression during or after aromatase inhibitor treatment, in both the overall population and in patients with PIK3CA, , AKT1, , or PTEN- altered tumours. This study further explored patient-reported health-related quality of life (HRQOL), functioning, symptoms, and symptom tolerability in CAPItello-291. Methods This phase 3, randomised, double-blind, placebo-controlled trial, which was conducted across 193 hospitals and cancer centres in 19 countries, enrolled women with any menopausal status or men, aged >= 18 years (>= 20 years in Japan), with hormone receptor-positive, HER2-negative locally advanced or metastatic breast cancer who had relapse or disease progression during or after treatment with an aromatase inhibitor, with or without previous cyclindependent kinase (CDK) 4 or 6 inhibitor therapy. Patients had an Eastern Cooperative Oncology Group/WHO performance score of 0 or 1 and could have received up to two previous lines of endocrine therapy and up to one previous line of chemotherapy for advanced disease. Patients were randomly assigned (1:1) using block randomisation (stratified according to the presence or absence of liver metastases, previous use of a CDK4/6 inhibitor [yes vs no], and geographical region) to receive oral capivasertib 400 mg (twice daily for 4 days, followed by 3 days off) plus intramuscular fulvestrant 500 mg (every 14 days for the first three injections, then every 28 days) or placebo with matching fulvestrant dosing. The dual primary endpoint of the trial was investigator-assessed progression-free survival assessed both in the overall population and among patients with PIK3CA, , AKT1, , or PTEN-altered tumours. The EORTC Quality of Life Questionnaire 30-item core module (QLQ-C30) and breast module (QLQ-BR23), Patient- Reported Outcomes version of the Common Terminology Criteria for Adverse Events (PRO-CTCAE), and Patient Global Impression of Treatment Tolerability (PGI-TT) questionnaires were used to assess patient-reported outcomes. Evaluation of EORTC QLQ-C30 and EORTC QLQ-BR23 were secondary endpoints and evaluation of PRO-CTCAE and PGI-TT were pre-defined exploratory endpoints, and these endpoints are the subject of analysis in this Article. Data were collected at baseline and prespecified timepoints. Patient-reported outcomes were analysed in all randomly assigned patients with an evaluable baseline assessment and at least one evaluable post-baseline assessment. Change from baseline was assessed using mixed model with repeated measures for EORTC QLQ-C30 and summarised for QLQ-BR23. Time to deterioration was described using the Kaplan-Meier method. PGI-TT and PRO-CTCAE responses were summarised at each treatment cycle. Patient-reported outcomes were not prospectively powered for statistical comparison. The trial is registered with ClinicalTrials.gov, NCT04305496. Findings Between June 2, 2020, and Oct 13, 2021, 901 patients were enrolled, of whom 708 patients were randomly assigned to receive capivasertib-fulvestrant (n=355) or placebo-fulvestrant (n=353). The median age of the patients was 59 years (IQR 51-67) in the capivasertib-fulvestrant group and 58 years (IQR 49-66) in the placebo-fulvestrant group. At data cutoff (Aug 15, 2022), the median duration of follow-up for progression-free survival in censored patients was 130 months (IQR 91-167) for capivasertib-fulvestrant and 127 months (IQR 20-164) for placebo- fulvestrant in the overall population. EORTC QLQ-C30 global health status/quality of life (GHS/QOL) scores were maintained from baseline and were similar between treatment groups throughout the study period (difference in mean change from baseline of -25 [95% CI -45 to -06] with capivasertib-fulvestrant vs -56 [-79 to -34] with placebo-fulvestrant; treatment difference 31 [95% CI 02 to 60]). Median time to deterioration in EORTC QLQ-C30 GHS/QOL was 249 months (95% CI 138 to not reached) in the capivasertib-fulvestrant group and 120 months (102 to 157) in the placebo-fulvestrant group (hazard ratio [HR] 070, 95% CI 053 to 092). Time to deterioration HRs for all EORTC QLQ-C30 and QLQ-BR23 subscale scores showed little difference between the treatment groups, except for diarrhoea, which was worse in the capivasertib-fulvestrant group than in the placebo-fulvestrant group (HR 275, 95% CI 201-381). In PRO-CTCAE symptom assessment, the proportion of patients reporting loose and watery stools frequently or almost constantly was 29% higher at cycle 1, day 15 in the capivasertib-fulvestrant group than in the placebo-fulvestrant group, decreasing at subsequent cycles. Other PRO-CTCAE-reported symptoms (rash, mouth or throat sores, itchy skin, and numbness or tingling in hands or feet) were absent or mild in most patients in both groups throughout treatment. According to the PGI-TT, most patients in both groups reported not at all or a little bit of bother from treatment side-effects. Interpretation Patient-reported outcomes from CAPItello-291 demonstrated that capivasertib-fulvestrant delayed time to deterioration of GHS/QOL and maintained other dimensions of HRQOL (except symptoms of diarrhoea) similarly to fulvestrant. With the clinical efficacy and manageable safety profile, these exploratory results further support the positive benefit-risk profile of capivasertib-fulvestrant in this population. Copyright (c) 2024 Elsevier Ltd. All rights reserved, including those for text and data mining, AI training, and similar technologies. | Background/Introduction:  This study looked at people with a type of breast cancer that is not too rare. These people had cancer that had spread or come back after certain drugs stopped working. A new combo of two drugs was tested. These drugs are called capivasertib and fulvestrant. The study checked how people felt and if side effects from this treatment were hard to deal with.  Methods:  The study was done all around the world. It included grown-ups with breast cancer. These people had cancer that was tough to treat. They had tried other cancer drugs before. The people were split into two main groups. One group got the new drug mix. The other got just a look-alike drug plus the old treatment. The people did not know which group they were in. They answered health surveys often. These surveys asked how they felt, their side effects, and how much the side effects bothered them.  Findings/Results:  The study looked at more than eight hundred people. Most people were women around their late fifties. Those who got the drug mix felt better for longer. They said their health stayed the same for more time when taking capivasertib and fulvestrant. One problem was that more people had loose stool in the drug mix group. But other issues, like skin itching or sore mouth, did not get worse. Most people said the new drugs did not bother them too much.  Conclusion:  People with breast cancer who got the new mix stayed feeling good for longer. Their side effects were not too bad—only gut problems showed up more at first, but they got better later. This shows the mix is safe and can help people feel better longer. Doctors think this drug mix can help more people with this kind of breast cancer. More work may be done in the future to learn more.  Short Summary:  This study tested two drugs to help people with a common kind of breast cancer. Most people in the study were women who had tried other drugs before. The drug mix helped them feel better for more time. It caused some gut trouble at first, but other side effects stayed mild. Most people did not feel too bothered by treatment. The results show this drug mix may be a good choice for helping people with tough-to-treat breast cancer. | Background/Introduction:  This study looks at a drug mix called capivasertib with fulvestrant. These drugs are tested in people with a certain type of breast cancer that grows even after past treatment. Doctors wanted to see how the patients feel during treatment, such as how they handle side effects and how the drugs change daily life.  Methods:  The study took place in many hospitals across many countries. Adults with the right kind of breast cancer took part, including both women and men. These people had already tried one or two other treatments that did not stop the cancer. Patients got either the study drug or a fake pill, so no one knew who got what. Everyone also got fulvestrant shots. The people in the study told doctors how they felt using forms that asked about symptoms and side effects. These answers helped to see how well people felt and if they could handle the treatment.  Findings/Results:  A lot of patients joined the study. Some got the drug mix and some got the fake pill. People taking capivasertib with fulvestrant did not feel much worse in general health or daily life during the study. Both groups had about the same experience, except that people on the drug mix had more loose stools, mostly early in the treatment. Most people said side effects were not too bad. Other problems like rash, sore mouth, or numb spots were mostly small and did not bother much.  Conclusion:  This study showed that the mix of capivasertib and fulvestrant helped slow down the return of worse health in people with this breast cancer. Most people felt about the same as those who got the fake drug, and side effects were mostly mild. These facts suggest the drug mix works well and is safe for many patients.  Short Summary:  This study looked at a mix of two drugs for adults with a kind of breast cancer that came back after past treatments. The goal was to see how well patients felt on the drugs and if they could handle them. Most people taking the drug mix felt much like those who got a fake pill. They also had fewer signs of getting worse for a longer time. One side effect, loose stools, was more common with the drug mix, but many people were not bothered much. In the end, the study suggests that the mix works and is safe. More future studies are likely. | Background/Introduction:  This study looked at how people with a kind of breast cancer felt while taking a new mix of two drugs. This kind of breast cancer grows with help from certain body chemicals. One of the drugs (capivasertib) is meant to stop cancer from growing. The other drug (fulvestrant) is already used for breast cancer. The goal was to learn not only if these drugs could stop the cancer from getting worse but also how people felt while on the drugs.  Methods:  Women and men with this breast cancer, who were adults and had seen their cancer come back or get worse after using a common treatment called aromatase inhibitors, joined the study. Some people got the new drug mix. The others got a lookalike pill with fulvestrant. Both the doctors and the people in the study did not know who got which treatment, making the test fair. They talked to the people taking part and asked how they felt, how their daily life was going, what problems they had from the drug, and how much those problems bothered them. These talks went on through the study.  Findings/Results:  The study found that people taking the new drug mix felt okay for a longer time before starting to feel worse. Their answers showed they stayed close to how they felt at the start and stayed active. One downside for more people who took the new mix was having loose stools, which went down over time. Other side effects, like skin rash, sore mouth, or feel of pins and needles in hands or feet, were light for most. Many people in both groups said the drug side effects did not bother them or only bothered them a little.  Conclusion:  Most people taking the new drug mix still felt good during the treatment and were not too bothered by side effects. The new drug mix worked to stop cancer from growing for longer, and people could go about their daily lives. Even though more people got loose stools, it got better later. These results show the new mix is helpful and safe for people with this kind of breast cancer.  Short Summary:  The study checked how people with a special kind of breast cancer felt while on a new drug mix. People who had this mix stayed well longer and felt mostly okay. A few people had loose stools, but it got better. Other side effects were mild and did not bother most. The drug mix helped slow the cancer and kept people feeling alright. This shows the mix can help and is not hard to manage. |
| CancerRes | 4 | 10.1016/S1470-2045(25)00128-7 | Pub2Post | Background Infections are the second leading cause of death in patients with cancer and are often caused by resistant bacteria. However, the frequency of antimicrobial resistance (AMR) in outpatients with cancer is not well understood. We aimed to compare the frequency of AMR bacterial pathogens in outpatients with and without cancer. Methods This retrospective cohort study evaluated antimicrobial susceptibility of bacteria isolated from adults (aged >= 18 years) with and without cancer seeking care in 198 outpatient health-care settings in the USA. Data were collected using the BD Insights Research Database. Patients who were not prescribed cancer medications or not admitted to an inpatient cancer unit in the predefined period were categorised as patients without cancer. Patients were included in the cancer cohort if they received medication solely or sometimes indicated for cancer. Data on gender and race or ethnicity were not collected. Non-duplicate and non-contaminant pathogens collected from various samples (ie, blood, intra-abdominal, respiratory, urine, skin or wound, and other) in outpatients were used to assess the coprimary outcomes: overall and source-specific proportions of non-susceptible pathogen isolates with corresponding AMR odds ratios (ORs); and rates of AMR pathogens per 1000 isolates with corresponding AMR incidence rate ratio (IRR) in patients with and without cancer. Findings Data were collected between April 1, 2018, and Dec 31, 2022. 53 006 (3 center dot 2%) of 1 655 594 pathogens identified were from 27 421 patients with cancer and 1 602 588 (96 center dot 8%) were from 928 128 patients without cancer. For Pseudomonas aeruginosa, carbapenem non-susceptibility was higher in pathogen isolates from patients with cancer (816 [14 center dot 4%] of 5683) than patients without cancer (10 709 [11 center dot 3%] 94 419; OR 1 center dot 22 [95% CI 1 center dot 13-1 center dot 32]). For Enterobacterales, fluoroquinolone non-susceptibility was higher in pathogen isolates from patients with cancer (8662 [28 center dot 0%] of 30 867) than patients without cancer (238 479 [21 center dot 8%] of 1 095 996; OR 1 center dot 44 [1 center dot 40-1 center dot 47]), as was carbapenem non-susceptibility (472 [1 center dot 5%] of 30 867 vs 9165 [0 center dot 8%] of 1 095 996; OR 1 center dot 89 [1 center dot 72-2 center dot 07]), multidrug-resistant pathogens (2672 [8 center dot 7%] of 30 867 vs 48 962 [4 center dot 5%] of 1 095 996; OR 2 center dot 03 [1 center dot 95-2 center dot 11]), and extended-spectrum beta-lactamase producers (4343 [16 center dot 5%] of 26 327 vs 93 977 [9 center dot 4%] of 996 853; OR 1 center dot 96 [1 center dot 90-2 center dot 03]). For Staphylococcus aureus, meticillin resistance was higher in pathogen isolates from patients with cancer (4747 [53 center dot 0%] of 8959) than patients without cancer (129 291 [48 center dot 3%] of 267 520; OR 1 center dot 20 [1 center dot 15-1 center dot 25]). For Enterococcus spp, vancomycin resistance was higher in pathogen isolates from patients with cancer (1329 [18 center dot 6%] of 7145) than patients without cancer (12 333 [9 center dot 1%] of 135 772]; ORR 2 center dot 20 [2 center dot 06-2 center dot 34). The rates and corresponding IRRs of AMR pathogens per 1000 isolates was also higher in patients with cancer compared with patients without cancer, particularly for carbapenem non-susceptible P aeruginosa (IRR 2 center dot 06 [1 center dot 91-2 center dot 21]) and vancomycin-resistant enterococci (IRR 3 center dot 06 [2 center dot 89-3 center dot 24]). For all comparisons, p<0 center dot 0001. Interpretation AMR proportions and IRRs for most key pathogens were up to three-times higher in isolates from outpatients with cancer than those without cancer, highlighting the need for enhanced surveillance, infection prevention, and timely diagnostic stewardship to improve antibiotic prescribing in this population. Copyright (c) 2025 Elsevier Ltd. All rights reserved, including those for text and data mining, AI training, and similar technologies. | Background/Introduction:  People with cancer often get sick from bugs that don’t die when given common drugs. These hard-to-treat bugs are called drug-strong germs. For people with cancer who get treatment outside the hospital, we don’t know how often they get these strong germs. This study looked at how often these bugs show up in people with and without cancer who see doctors outside of hospitals.  Methods:  The study used medical records from lots of clinics in the United States. It looked at grown-ups who got treated outside of hospitals. The researchers checked samples from body parts like blood, pee, skin, and lungs. They looked at how often strong germs were found in each group. People were put in the cancer group if they had cancer drugs. Others went in the group without cancer.  Findings/Results:  The study checked germs from many people. A small part had cancer, while most did not. People with cancer had more strong bugs than people without cancer. For example, one type of bug called Pseudomonas was more often drug-strong in people with cancer. Other bugs, like Staphylococcus and Enterococcus, also showed more drug resistance in the cancer group. More kinds of strong germs were found in people with cancer across many sample types.  Conclusion:  People with cancer who get care outside of hospitals have more trouble with strong bugs. It is clear they need more careful checks, better ways to stop bug spread, and fast, smart ways to pick the right drugs.  Short Summary:  The study looked at how often hard-to-kill germs showed up in people with and without cancer who visited clinics. People with cancer had these strong bugs more often than others. The study shows we need better ways to find and stop these germs early in people with cancer. This can help doctors choose the right treatment sooner. More studies may help improve care for these patients. | Background/Introduction:  People with cancer often get sick with infections. These infections can be very serious and are often caused by germs that no longer respond to common drugs. Doctors do not know much about how often this happens to people with cancer who are treated outside of a hospital. This study looked at how often these tough-to-treat germs show up in these people compared to other patients.  Methods:  The study looked at health records from many clinics across the United States. It focused on grown-ups with and without cancer who got care outside the hospital. The research team studied germs taken from places like blood, pee, wounds, or lungs. They checked if these germs could still be treated with common drugs. This helped them find out if the germs in cancer patients were harder to treat than in others.  Findings/Results:  Over a few years, the team studied samples from many people, including both those with and without cancer. They found that germs from people with cancer were more likely to no longer respond to many common drugs. This was true for different kinds of germs and for samples taken from many body parts. The germs from people with cancer were almost twice as likely, and in some cases three times as likely, to be hard to treat.  Conclusion:  The study showed that people with cancer are more likely to get infections from germs that cannot be easily treated. This is a big problem. It means doctors need to watch these patients more closely, work on stopping these infections early, and make smarter choices about which drugs to give them.  Short Summary:  This study looked at how often germs stop working with usual medicine in people with cancer compared to those without cancer. It found that people with cancer who are treated outside the hospital get more infections with germs that do not respond well to drugs. The germs were tougher to treat in many ways. This shows we need better steps to watch, stop, and treat these infections early in cancer patients. More research may help doctors find better ways to care for these people. | Background/Introduction:  People with cancer often get sick from germs. These germs can be harder to kill with medicine. This is a big problem. The goal of this study was to see how often these tough germs show up in people with cancer who are not in the hospital, and to see if that happens more than in people without cancer.  Methods:  This study looked at past health records from many health places in the United States. It checked the germs found in grown-ups who had cancer and those who didn’t. The study only used germs from real infections and threw out wrong or mixed-up ones. They looked at samples from things like blood, pee, skin, and more. They wanted to know how often these germs did not die from usual medicines.  Findings/Results:  The study took place over a few years. It found that people with cancer had more hard-to-treat germs than people without cancer. These germs were more likely to not respond to certain strong medicines. For example, some germs in people with cancer were about two or three times more likely to fight off the medicines than in people without cancer.  Conclusion:  People with cancer who are not staying in a hospital still have germs that are harder to treat compared to others. This means it's very important to watch these cases closely, stop the spread of germs, and pick the right medicine early. It also shows we need more smart steps in fighting germ problems in people with cancer.  Short Summary:  This study looked at grown-ups in the United States who had infections. Some had cancer, and some did not. The study wanted to see which group had more germs that medicine could not kill. It found that people with cancer had more of these tough germs. This shows a need for better ways to stop and treat these germs in cancer patients. Follow-up work could help stop bad outcomes before they happen. |
| CancerRes | 5 | 10.1016/S1470-2045(25)00006-3 | Pub2Post | Background The phase 2 randomised Young-PEARL study demonstrated that palbociclib plus exemestane with ovarian function suppression significantly prolonged progression-free survival compared with capecitabine in premenopausal women with hormone receptor-positive, HER2-negative metastatic breast cancer. Here, we report results of the protocol-specified secondary endpoint of overall survival. Methods Young-PEARL was a multicentre, randomised, open-label, phase 2 study conducted at 14 institutions in South Korea. Premenopausal women aged 19 years or older with histologically confirmed hormone receptor-positive, HER2-negative metastatic breast cancer that recurred or progressed during or after previous tamoxifen treatment, who were aromatase inhibitor naive, and had an Eastern Cooperative Oncology Group performance status of 0-2 were eligible. One previous line of chemotherapy was permitted in the metastatic setting. Eligible patients were randomly assigned (1:1), using block randomisation (block size of two) stratified by previous chemotherapy for metastatic breast cancer and presence of visceral metastasis, to receive either palbociclib (orally, 125 mg per day on a 3-weeks-on, 1-week off schedule) plus exemestane (orally 25 mg daily) with leuprorelin (subcutaneously 375 mg on day 1 of each 28-day cycle) or capecitabine (orally, 1250 mg/m(2) twice a day on a 2-weeks-on, 1-week-off schedule) until disease progression or unacceptable toxicity). The primary endpoint was progression-free survival. Overall survival was a secondary endpoint. All analyses were done in the modified intention-to-treat population (ie, included all patients randomly assigned to treatment who had at least one post-baseline CT scan and excluded those who did not receive study medication and who had any major violation of the eligible criteria). Safety was assessed in all patients who received any study treatment. This study is registered with ClinicalTrials.gov, NCT02592746, and is now complete. Findings Between June 15, 2016, and Dec 10, 2018, 189 patients were enrolled. 184 patients were randomly assigned to the palbociclib plus endocrine therapy group (n=92) or the capecitabine group (n=92), of whom 174 were included in the modified intention-to-treat population (n=90 in the palbociclib plus endocrine therapy group and n=84 in the capecitabine group). All patients were female and ethnicity data were not collected. As of data cutoff (Feb 29, 2024), median follow-up was 540 months (IQR 341-744). Median progression-free survival was 195 months (90% CI 143-222) for palbociclib plus endocrine therapy and 140 months (117-187) for capecitabine (hazard ratio 074 [90% CI 057-098]; one-sided log-rank p=0036). 52 (58%) of 90 patients in the palbociclib plus endocrine therapy group and 48 (57%) of 84 in the capecitabine group died, with a median overall survival of 548 months (95% CI 489-771) in the palbociclib plus endocrine therapy group versus 578 months (463-892) in the capecitabine group (hazard ratio 102 [95% CI 069-151]; p=092). The most common grade 3 or worse adverse event was neutropenia (59 [64%] of 92 in the palbociclib plus endocrine therapy group vs 15 [18%] of 85 in the capecitabine group) . No treatment-related deaths occurred. Interpretation With extended follow-up, palbociclib plus exemestane with ovarian function suppression continued to show a significant benefit in progression-free survival compared with capecitabine in premenopausal patients with hormone receptor-positive, HER2-negative metastatic breast cancer who had been previously treated with tamoxifen; however, no improvement in overall survival was seen. Given the progression-free survival benefit, the upfront use of palbociclib plus endocrine therapy is the preferred option for premenopausal women, although a capecitabine-first strategy might be an alternative treatment strategy for maintaining overall survival in resource-limited settings. Copyright (c) 2025 Elsevier Ltd. All rights reserved, including those for text and data mining, AI training, and similar technologies. | Background/Introduction:  This study looked at young women who had breast cancer that spread to other parts of the body. Their type of breast cancer grows with the help of female hormones. These women were still getting their monthly periods. The study wanted to see which treatment helped better: one with a mix of pills that stops cancer from growing, or a type of chemo pill.  Methods:  Women who had this kind of breast cancer were picked to join the study. They had to be adults who tried one kind of medicine before. The researchers split the women into two groups—one got the new pill mix (palbociclib with two others), and the other got the chemo pill (capecitabine). Doctors checked how long the cancer stayed away and how long the women lived. The women went to several clinics in South Korea for the study.  Findings/Results:  A little under two hundred women joined. One group got the new pill mix, and the other got the chemo pill. The group that took the new pills had their cancer stay quiet for a few more months. Both groups lived for about the same amount of time. The new pill group had more cases of low white blood cells, which help fight germs. But no one died because of the treatment.  Conclusion:  The new pill mix helped keep the cancer away longer, but both treatments led to about the same life span. Doctors may pick the new pills when they want to stop the cancer’s return. But the chemo pill can still be used where other drugs are not easy to get.   Short Summary:  The study tested two treatments on young women with a fast-spreading type of hormone-driven breast cancer. One group got a new drug mix, and the other took a chemo pill. The new drug kept cancer away a bit longer, but both groups lived a similar amount of time. The new drug had more side effects, like fewer white blood cells. Chemo may work just as well where money or care choices are limited. More studies on long-term impact may be done later. | Background/Introduction:  This study looked at young women with breast cancer that had spread in the body. The type of breast cancer in these women grows with help from hormones. The women had not gone through menopause yet. The study compared two ways to treat them. One way used two pills named palbociclib and exemestane, along with a shot that stops the ovaries. The other way used a pill called capecitabine. The goal was to see which treatment worked better and if one helped the women live longer.  Methods:  The study took place in many hospitals in South Korea. Young women who still had their periods and had this kind of breast cancer joined. They had tried one other kind of cancer drug before, but not certain hormone pills. They were put into two groups by a fair system, like flipping a coin. One group got palbociclib and exemestane with a shot to turn off their ovaries. The other group got capecitabine. Doctors watched the women to see how long the cancer stayed away and how long they lived overall. They also watched for bad side effects.  Findings/Results:  The study included close to two hundred women. About half got each type of treatment. All were women, and race was not asked. Doctors followed the women for almost five years. The group getting palbociclib and exemestane had the cancer stay away for a bit longer than the women taking capecitabine. But, both groups lived about the same amount of time. The most common bad side effect in the palbociclib group was a drop in white blood cells, which fight germs. No one died because of the treatment.  Conclusion:  The treatment with palbociclib, exemestane, and ovary-blocking shots helped the cancer stay away longer. But it did not help women live longer than when they took capecitabine. Still, the group had more time without cancer growing, so this might be a better first choice. In places where money or drugs are hard to get, capecitabine could still be used. No more studies were said to be planned.  Short Summary:  This study tested two treatments in young women whose breast cancer had spread and needed hormone support to grow. One group took a mix of pills and a shot to stop their ovaries. The other group took a different pill. The first group had more time before the cancer came back. But both groups lived about the same. The pill-and-shot mix may be better for stopping the cancer early, and the other way may still be okay where choices are limited. | Background/Introduction:  This study looked at how well two cancer treatments worked for young women with a kind of breast cancer that had spread (called “metastatic breast cancer”). All the women were still having periods (called “premenopausal”) and had a type of breast cancer that reacts to hormones. One treatment, called palbociclib with hormone pills and a shot that stops the ovaries from working, was compared to another called capecitabine, which is a form of chemo. The goal was to see which helped the women live longer without the cancer getting worse.  Methods:  The study took place across many hospitals in South Korea. Women who already tried another common breast cancer treatment called tamoxifen, but whose cancer came back or got worse, took part. They were split by chance into two groups. One group got palbociclib with hormone pills and a shot to stop the ovaries from working. The other group got capecitabine chemo pills. Both groups kept getting medicine until their cancer got worse or they had strong side effects. The main thing the study looked at was how long the women stayed well before the cancer grew. It also checked how long they lived in total. The study followed each woman closely and made sure they all had the needed checkups.  Findings/Results:  Close to two hundred women joined the study. Half of them got the palbociclib combo and the other half got capecitabine. The study ran for more than four years. The researchers found that the palbociclib combo helped women stay well for more months without their cancer growing, compared to the capecitabine group. But the total time the women lived was pretty much the same between both groups. A common strong side effect in the palbociclib group was that their body made fewer white blood cells, which help fight germs. But no one died from the treatment.  Conclusion:  The cancer slowed down for more time when young women took palbociclib with hormone therapy and an ovary-blocking shot, compared to just taking capecitabine chemo. But the overall time they lived was not better with one over the other. So palbociclib combo may be better if the goal is to keep the cancer from growing fast, while capecitabine may still be an option if money or medicine supply is a problem. The study is now done.  Short Summary:  This study looked at young women with breast cancer that had spread. It tested if a combo of a cancer pill, hormone pills, and a shot to stop the ovaries from working helped more than a chemo pill. The combo helped stop the cancer from growing as fast, but both groups lived about the same. The combo may be a better first choice to keep women well longer, but the chemo pill still works if money or supply is tight. No one died from side effects. |
| CancCell | 1 | 10.1016/j.ccell.2025.03.017 | Pub2Post | Selecting optimal candidates for next-generation anti-human epidermal growth factor receptor 2 (HER2) antibody-drug conjugates (ADCs) remains challenging. We conduct a prespecified translational study to identify treatment biomarkers in SHR-A1811-treated HER2-positive breast cancer patients from the phase 2 neoadjuvant FASCINATE-N trial using DNA and RNA sequencing, computational pathology, and single-cell in situ spatial imaging. In the hormone receptor (HR)-negative subgroup, a higher proportion and more infiltration of immune cells (i.e., tumor-infiltrating lymphocytes [TILs]), particularly cytotoxic T cells, are associated with better treatment responses. In the HR-positive subgroup, the closeness and aggregation of HER2-strong-positive tumor cells, as opposed to a uniform distribution, are linked to a lower response rate and HER2 luminal-like (HER2-LUM) subtype, which more closely resembles HR+/HER2-breast cancer. In addition, we develop a clinically practical predictive model capable of predicting neoadjuvant treatment responses to SHR-A1811 and other novel ADCs based on clinicopathological characteristics and pathological images. | **Background/Introduction:**  This study looked at a new type of breast cancer drug used before surgery. The drug is called SHR-A1811. It is a kind of cancer drug that joins a special protein (called an antibody) with a killing chemical. This combo helps the drug find and attack only the cancer cells that have a protein called HER2. HER2 is found more in some breast cancer types. But the drug does not work the same for every patient. The goal was to learn who might get the best results from this drug.  **Methods:**  The doctors looked at old data from a past study. They worked with people who had breast cancer with too much HER2. They used special tools to check how the cancer cells and immune cells (body defense cells) looked and worked. They also used pictures of the cells and tested the genes (parts of the cell that tell it what to do). The study looked at what helped the drug work better in different people.  **Findings/Results:**  For people with a type of cancer that does not use hormones, the drug worked best when more body defense cells were in the tumor. These cells — especially ones called "killer T cells" — helped fight the cancer. But in cancers that do use hormones, if the cancer cells with high HER2 were clumped too close together, the drug did not work as well. This group showed signs that looked more like a different kind of breast cancer that acts slower.  **Conclusion:**  The team was able to come up with a way to guess how the new drug will work for each patient. They used cell pictures and data to help doctors pick the right people for this treatment. This might help make care better and faster. More work may be done in the future to test the guessing tool even more.  **Short Summary:**  This study looked at a new breast cancer drug that connects a smart helper (called an antibody) to a strong cancer-killing part. Doctors studied people with too much HER2 in breast cancer cells to see who got the most help from the drug. Those with more helpful defense cells in the tumor had better results. But, in other types of breast cancer, the cells being bunched up made the drug not work as well. The team made a tool to help pick who may benefit most from the drug. More studies could follow. | Background/Introduction  Doctors are still trying to figure out who will benefit most from a type of drug that links a special protein-targeting medicine to cancer-killing drugs. This study looks at people with a kind of breast cancer that has high levels of a protein called HER2. These drugs may help more when given before the main cancer treatment. The goal was to find signs in the body that show if this new drug will work well.  Methods  The study looked at people with HER2-positive breast cancer. Before other treatment, these people got a new linked drug called SHR-A1811. The team studied samples from the tumors using many tools, such as gene testing, computer images, and very detailed views of each cell in the tumor. The goal was to match changes in the tumor with how well the drug worked.  Findings/Results  In people whose tumors did not have extra hormone signals, the drug worked better when there were more immune cells—these are the body’s fighter cells—inside the tumor. A certain fighter type, called killer T cells, seemed to help most. In people whose tumors did show hormone signals, the drug worked less well when the cancer cells were bunched close together with more HER2 signals. These tumors looked like another type of breast cancer that usually does not respond well to this kind of medicine. Doctors made a model to guess how well this drug or others like it will work, using pictures and past health signs.  Conclusion  This new drug worked better in tumors with many cancer-fighting cells. But in some cases where the tumor cells were all packed in tight with high HER2, the drug did not help so much. A new model may help doctors guess early if the drug will help or not. This could make choosing the right care easier in the future.  Short Summary  This study looked at a new drug used before the main treatment for people with HER2-type breast cancer. The drug worked better when the patients’ tumors had many immune cells, which help fight cancer. It did not work as well when tumor cells were close together with high HER2 levels. A new tool was made to help tell if the drug could help each patient. More studies may help improve care choices for breast cancer in the future. | Background/Introduction:  This study looked at a new kind of drug for women with a type of breast cancer called "HER2-positive." This type of breast cancer has too much of a protein called HER2. The new drug is a mix of an antibody and a cancer drug, which means it can find the cancer and give the drug right where it's needed. But not all people with this cancer respond the same way to the drug. The goal of this study was to find out who might benefit most from the treatment.  Methods:  The researchers studied people with HER2-positive breast cancer. They looked at their cancer samples in many ways—like reading their genes, looking at their cells one by one, and studying pictures of their tumors. They wanted to see if certain things in the body could help guess if the treatment would work. The people were part of a larger test of the drug, taken before they had surgery.  Findings/Results:  People with a kind of cancer that does not have hormone signals did better when they had more immune cells that fight cancer, like T-cells, inside the tumor. People whose tumors had many strong HER2 cells clumped in one spot did not do as well. This kind of tumor looked more like another type of breast cancer that usually does not have too much HER2. The team used these findings to build a tool to help doctors guess how well this drug might work for someone.  Conclusion:  This study helped show important signs in tumors that can tell how well this new drug might work. It also gave doctors a tool to better pick who should get this drug before surgery. This could help more people get the right treatment and skip drugs that may not help them. The team may do more studies to make the tool even better.  Short Summary:  This study looked at women with a type of breast cancer called HER2-positive. It tested a new treatment that uses a mix of proteins and drugs to fight the cancer. The team found that some women, especially those without hormone signals and with more immune cells, responded better. Others, whose tumors were tightly packed with cancer cells, did worse. They built a tool to help doctors guess who this treatment will help most. If it works well in the real world, it could help doctors give the best drug to the right people. More tests may come in the future. |
| CancCell | 2 | 10.1016/j.ccell.2025.03.027 | Pub2Post | Circular RNA (circRNA) is a class of noncoding RNA with regulatory potentials. Its role in the transdifferentiation of prostate and lung adenocarcinoma into neuroendocrine prostate cancer (NEPC) and small cell lung cancer (SCLC) remains unexplored. Here, we identified circRMST as an exceptionally abundant circRNA predominantly expressed in NEPC and SCLC, with strong conservation between humans and mice. Functional studies using shRNA, siRNA, CRISPR-Cas13, and Cas9 consistently demonstrate that circRMST is essential for tumor growth and the expression of ASCL1, a master regulator of neuroendocrine fate. Genetic knockout of Rmst in NEPC genetic engineered mouse models prevents neuroendocrine transdifferentiation, maintaining tumors in an adenocarcinoma state. Mechanistically, circRMST physically interacts with lineage transcription factors NKX2-1 and SOX2. Loss of circRMST induces NKX2-1 protein degradation through autophagy-lysosomal pathway and alters the genomic binding of SOX2, collectively leading to the loss of ASCL1 transcription. | Background/Introduction:  Some kinds of cancer can change into types that grow faster and are harder to treat. This study looked at two kinds: prostate cancer and lung cancer. These can turn into more dangerous forms called small cell lung cancer and neuroendocrine prostate cancer. A small piece inside the cell, called circular RNA (this is a kind of material that helps control what the cell does), may play a big part in this change. The study focused on one special circular RNA called circRMST. Scientists wanted to know how this little piece helps the cancer become worse.  Methods:  Researchers tested this special circular RNA in mice and human cancer cells. They used many tools to block it or cut it out from cancer cells. These tools have names like short hairpin RNA, small interfering RNA, and CRISPR—those are ways to turn off or remove tiny parts of cell data. They also watched how proteins inside the cancer cells changed when the circular RNA was missing.  Findings/Results:  The researchers found that many of these bad cancer cells had a lot of this special circular RNA. When they stopped the circular RNA, the fast-growing cancer cells could not grow well. They also found that a key protein, one that says, “act like a nerve cell,” was not made anymore when the circular RNA was gone. The cancer stayed in its slower form and did not change into the faster, more harmful kind. The circular RNA worked with other tools inside the cell that act like switches. Without it, these switches would break down or stop working.  Conclusion:  The study shows that this circular RNA is needed for the cancer to grow faster and get worse. Taking it away helps stop the cancer from changing into the more dangerous type. Future studies might try to block this RNA to create new treatments and help keep people safe from the more harmful cancers.  Short Summary:  The study looked at how some slow-growing cancers change into faster, more harmful types. It found that a small cell part, called circular RNA, helps this bad change happen. By removing it from the cells, the cancer stayed in the slower and safer stage. The results show that this circular RNA is key to the change and may be used to stop cancer from getting worse. Future tests may help find ways to block it and treat this kind of cancer better. | Background/Introduction  Some cancers from the lung and prostate can change into a different kind called "neuroendocrine" cancer, which is harder to treat. This change happens through a process called "transdifferentiation," where one cell type turns into another. Scientists found a small piece of genetic material called circRMST in these cancers. circRMST is a type of RNA, which helps cells make proteins. But this one does not make proteins; instead, it helps control other genes. Before now, no one knew what role circRMST played in this cancer switch.  Methods  The study looked at animals and cell groups in labs. Scientists used tools to turn off circRMST and watched what happened. These tools (called shRNA, siRNA, CRISPR-Cas13, and Cas9) help change or remove small parts of a cell’s genes. They also used mice made to grow cancers like humans do. The team watched how these mice grew their tumors when circRMST was either present or removed.  Findings/Results  The study showed that circRMST is needed to help some cancers grow and become more dangerous. When circRMST was taken away, the cancers did not change into the harder-to-treat type. The tumors stayed in their first form. The team also learned that circRMST works with two helpers called transcription factors. These are like keys that turn genes on or off. Without circRMST, these keys do not work right, and the cancer cannot change into the tough type.  Conclusion  The research showed that circRMST helps cancer cells change into the more harmful type. When it’s removed, the cells stay in their first, less dangerous form. This means circRMST might be a new way to stop these cancers from getting worse. More studies may be done later to see how to use this in patients.  Short Summary  Some lung and prostate cancers change into harder-to-treat forms. A tiny piece of genetic material called circRMST helps this change happen. Scientists found that taking circRMST away stops the cancer from changing. This was tested in cancer cells in labs and in mice. The cancer stayed in its first form when circRMST was not there. This gives hope for new ways to stop cancer from getting worse. More research might follow to learn how to use this for new treatments in people. | **Background/Introduction**  Some types of cancer in the lung and prostate can change into a more deadly kind called neuroendocrine cancer. This change is hard to stop and makes treatment harder. Some small round molecules called circular ribonucleic acid, or circRNA, may be part of this process. These tiny molecules are not used by cells to make proteins but instead help control how cells work. One of these molecules, circRMST, may play a big role in helping cancer cells change into the tougher kind in lung and prostate cancer.  **Methods**  Scientists looked at many cancer samples from both people and mice. They checked which cells had lots of circRMST. They shut off this molecule using lab tools to see what would happen to the cancer growth. These tools helped stop the circRMST from working in cancer samples. They also looked at how this RNA worked with other important cell parts called transcription factors. Transcription factors are like light switches that turn genes on or off to tell the cell what to do.  **Findings/Results**  The researchers found a lot of the circRMST in the tougher types of prostate and lung cancer. When they turned off circRMST, the cancer slowed down, and the cells stopped changing into the more harmful type. This circRNA worked closely with other parts of the cell that help it change type. Without circRMST, one of these helper proteins, NKX2-1, broke down faster, and another, SOX2, could not do its job right. This made the cancer cells stay in their old form and not switch into the more dangerous kind.  **Conclusion**  The study shows that circRMST helps cancer grow and change into a harder-to-treat form. Blocking this little round RNA may keep the cancer in a less harmful state. These findings help us learn more about how some cancers become worse. Future studies may look into new ways to stop this RNA to help treat cancer better.  **Short Summary**  This study looked at a tiny circular molecule called circRMST in lung and prostate cancer. It helps these cancers change into a tougher form. Turning it off made the cancer grow less and stay in a safer form. This gives hope that blocking it may help in the future. More research may follow. |
| CancCell | 3 | 10.1016/j.ccell.2025.02.018 | Pub2Post | PDGFRA is crucial to tumorigenesis and frequently genomically altered in high-grade glioma (HGG). In a comprehensive dataset of pediatric HGG (n = 261), we detect PDGFRA mutations and/or amplifications in 15% of cases, suggesting PDGFRA as a therapeutic target. We reveal that the PDGFRA/KIT inhibitor avapritinib shows (1) selectivity for PDGFRA inhibition, (2) distinct patterns of subcellular effects, (3) in vitro and in vivo activity in patient-derived HGG models, and (4) effective blood-brain barrier penetration in mice and humans. Furthermore, we report preliminary clinical real-world experience using avapritinib in pediatric and young adult patients with predominantly recurrent/refractory PDGFRA-altered HGG (n = 8). Our early data demonstrate that avapritinib is well tolerated and results in radiographic response in 3/7 cases, suggesting a potential role for avapritinib in the treatment of HGG with specific PDGFRA alterations. Overall, these translational results underscore the therapeutic potential of PDGFRA inhibition with avapritinib in HGG. | Background/Introduction  Some kids and teens get a type of brain cancer called high-grade glioma. This cancer grows fast and is hard to treat. In some of these cancers, a part of the body called PDGFRA (a gene that helps cells grow) does not work right. When this gene changes, it can help the tumor get worse. In this study, the goal was to see if a drug called avapritinib can help treat brain tumors with these gene changes.  Methods  Doctors looked at tissue from kids and teens who had this type of brain cancer. They searched for the PDGFRA gene changes. Then they tested avapritinib on cells in labs and in mice. Some young people with this cancer also got the drug. Their doctors looked to see if the tumors got smaller and if the kids felt okay taking the drug.  Findings/Results  The broken PDGFRA gene was found in a small part of the group. The drug worked well in lab tests and got into the brain, which is hard for many drugs to do. In real kids who got the treatment, most felt okay while taking it. In a few cases, the cancer shrank when the kids took the drug.  Conclusion  This study showed that avapritinib could help treat some brain cancers when the PDGFRA gene is not working right. It worked in labs and also helped some kids. It did not cause bad side effects in most kids. This means the drug might be useful for kids with these tumors. More studies may help to learn more in the future.  Short Summary  Doctors studied a brain cancer in kids and teens that grows fast. A gene that helps cells grow, called PDGFRA, can be broken in some of these cancers. A medicine called avapritinib was checked to see if it could help. The drug worked well in labs, went into the brain, and helped some kids whose tumors had this gene problem. It did not cause strong side effects for most. This study shows the drug might help some patients and that more tests could happen later on. | Background/Introduction  Some kids and teens get very bad brain tumors called high-grade gliomas. These tumors often grow fast and are hard to treat. A change in a body part called the PDGFRA gene is often found in these tumors. This gene helps cells grow too much when it has a problem. The study wanted to see if a drug called avapritinib could help fight these tumors.  Methods  The study looked at brain tumor samples from many young patients. The researchers checked if the tumors had problems in the PDGFRA gene. They gave the new medicine avapritinib to mice and some patients with this type of brain cancer. They looked at how well the drug worked and if patients felt okay while on it.  Findings/Results  The researchers found that the PDGFRA gene did not work right in about one out of every seven tumor samples. The drug avapritinib worked well in lab tests and in mice with brain cancer. It could even pass through a part of the body called the blood-brain barrier, which is hard for many drugs. In a small group of kids and teens with tough brain cancer, some got better pictures on brain scans after taking the drug. Most could take the medicine without feeling very sick.  Conclusion  This study shows that avapritinib might help treat certain brain tumors in young people that have problems in the PDGFRA gene. The early results are hopeful, but more tests with more patients are needed to make sure the drug works well and is safe.  Short Summary  Doctors studied a new drug called avapritinib to treat hard-to-fight brain tumors in kids and teens. These tumors had a trouble spot in a gene called PDGFRA, which helps the cancer grow. The drug showed good results in lab and mouse tests. Some young people with tough cancer felt better, and the medicine did not cause strong side effects. Researchers think this drug could help, and more studies will be done to learn more. | Background/Introduction:  Some children and teens get a type of brain cancer called high-grade glioma. This form of cancer can grow fast and is hard to treat. Many of these tumors have changes in a gene called PDGFRA, which helps cells grow. If this gene does not work right, it can help cancer grow. Doctors are trying new ways to treat this kind of cancer. A new drug called avapritinib may help stop the bad changes in the PDGFRA gene.  Methods:  To check if avapritinib works, lab tests were done using cancer cells from real patients. These tests looked at how well the drug could stop the bad gene from working. Then, the drug was given to mice and a small number of young people with this type of brain cancer. The doctors also looked at if the drug could reach the brain and if it caused any bad side effects.  Findings/Results:  The lab tests showed that avapritinib was good at stopping the PDGFRA gene from helping cancer grow. The tests also found that the drug could get into the brain in mice and people. It worked on cancer cells in the lab and in mice. When used with real patients, most of them handled the drug well. For a few patients, their brain scans even showed the cancer shrank after taking the drug.  Conclusion:  Avapritinib may help treat this fast-growing brain cancer by stopping the bad PDGFRA gene. It seems safe, can reach the brain, and may slow or shrink the cancer in some young people. More studies will be needed to see if it helps more patients and to learn the best way to use it.  Short Summary:  Some fast-growing brain cancers in kids and teens are linked to a bad gene called PDGFRA. A new drug called avapritinib was tested in lab and animal studies and showed it could stop this gene from helping cancer grow. When tried in a few young people, the drug got into the brain and was safe to use. Some even had smaller tumors after taking the drug. More tests are planned to see how well it works for more people. |
| CancCell | 4 | 10.1016/j.ccell.2024.11.005 | Pub2Post | Anti-tumor immunity is crucial for high-grade serous ovarian cancer (HGSC) prognosis, yet its adaptation upon standard chemotherapy remains poorly understood. Here, we conduct spatial and molecular characterization of 117 HGSC samples collected before and after chemotherapy. Our single-cell and spatial analyses reveal increasingly versatile immune cell states forming spatiotemporally dynamic microcommunities. We describe Myelonets, networks of interconnected myeloid cells that contribute to CD8+ T cell exhaustion post-chemotherapy and show that M1/M2 polarization at the tumor-stroma interface is associated with CD8+ T cell exhaustion and exclusion, correlating with poor chemoresponse. Single-cell and spatial transcriptomics reveal prominent myeloid-T cell interactions via NECTIN2-TIGIT induced by chemotherapy. Targeting these interactions using a functional patient-derived immuno-oncology platform demonstrates that high NECTIN2-TIGIT signaling in matched tumors predicts responses to immune checkpoint blockade. Our discovery of clinically relevant myeloid-driven spatial T cell exhaustion unlocks immunotherapeutic strategies to unleash CD8+ T cell-mediated anti-tumor immunity in HGSC. | Background/Introduction:  The immune system helps our body fight cancer. In a type of ovary cancer, this fight is very important. But, it is not clear how the body's line of defense changes after common cancer drugs.   Methods:  The team looked closely at samples from many people with this cancer. They studied small bits of the tumor before and after the people got the cancer drugs. Using special tools, they watched how tiny fighter cells from the immune system worked and changed over time.  Findings/Results:  They saw that after the drug treatment, helper cells called "myeloid cells" got in the way of the body’s fighter cells, called "CD8 T cells". These fighter cells became weak and tired. The team saw a group of these blocker cells forming networks, which made it harder for fighter cells to do their job. The more tired the fighter cells got, the worse the people responded to treatment. The study also found a pair of cell signals that stop the fighter cells from working. They tested stopping these signals and found some tumors did better.  Conclusion:  The body's defense cells can become weak from the drugs meant to fight cancer. This weakness is caused in part by blocker cells. Knowing this can help doctors try new ways to boost the fighter cells again and help with better treatment. Future research may explore these better ways.  Short Summary:  This study looked at how the body’s defense changes after drug treatment for a kind of ovary cancer. The goal was to see why some people do not get better. The team found that after the drugs, helper cells in the body formed groups that stopped fighter cells from working. These fighter cells became tired, which led to poorer results. They also found a signal between cells that could be blocked to help the body fight again. This shows how doctors may one day give better care with new types of treatment. More studies could come next. | Background/Introduction:  Ovarian cancer, which affects the female parts inside the belly, is hard to treat. The body’s own guards, called immune cells, are very important in fighting this cancer. One type of these guards, called T cells, helps destroy the bad cells. But when people get strong anti-cancer drugs (chemotherapy), these T cells can become tired and stop working well. Doctors and scientists don’t yet fully know how these treatments change the guards or where in the body this happens.  Methods:  The researchers looked at tissue from people with ovarian cancer. They checked the samples from before and after treatment. They used special tools that let them see each immune cell on its own and find out how the cells talk to each other in the body. They mostly looked at cells called myeloid cells—which are helper guards that give signals to other cells—and how they affect T cells.  Findings/Results:  After treatment, more groups of helper guards (myeloid cells) grew near the cancer. These groups were linked to tired-out killer T cells. In areas near the tumor, the helper guards showed different styles. Some were more bossy, and some were more chill. But both made the killer T cells stop fighting. The study also found a special way that the helper and killer cells talk to each other called NECTIN2-TIGIT. When this talking gets strong, it lowers the T cells' power. The team learned that if this signal is strong, a certain medicine might help better by waking the T cells up to fight again.  Conclusion:  The study shows that the strong drugs used to treat ovarian cancer might make the body’s own guards weak in the wrong places. By finding which guards and cell talks are causing the problem, doctors may one day give people a mix of treatments to keep the killer T cells strong. New care steps may come in the future to help the body's guards stay on the job longer.  Short Summary:  This study looked at how strong anti-cancer drugs affect how the body fights ovarian cancer. It found that helper guard cells gather in new places after treatment and wear out the main killer cells. A special kind of cell chatter, NECTIN2-TIGIT, makes this worse. When doctors know where and how this happens, they may stop it using special plans or drugs. This could help the body fight cancer better. Follow-up work will likely help make better treatments. | Background/Introduction  Doctors try to help people with a type of cancer called high-grade serous ovarian cancer. This kind of cancer can be hard to treat. The body’s immune system, which helps fight off sickness, also fights cancer. But it is still not clear what happens to those immune cells after people get regular cancer drugs.   Methods  To learn more, the researchers looked at over one hundred cancer samples. These came from people before and after they got the cancer drugs. They used very detailed tools to study small parts of the tumors and single immune cells. This helped them understand where different cells are and what they are doing.  Findings/Results  The study found that immune cells change a lot after the drugs are given. A group of immune cells called myeloid cells (these help call other cells to fight sickness) formed tight places inside the cancer. These cells made it hard for good fighting cells, called CD8+ T cells, to work right. These T cells became tired and stopped fighting cancer. Where some immune cells meet the tumor edges, they looked different and kept T cells out. This was a bad sign showing cancer might not get better from treatment. The researchers also found a special signal between the myeloid cells and T cells that starts after the drugs are given. This signal may explain why the T cells become so weak. They tested ideas in lab models built from human tumors to see what might work to stop this.  Conclusion  The study showed how cancer drugs can hurt the immune system’s fight. T cells become tired because of how myeloid cells act. But now that this is known, doctors can try new ways to stop that. Blocking the signal that hurts the T cells could help future care. More study is needed.  Short Summary  This study looked at a hard-to-treat cancer in the ovaries. It showed how cancer drugs weaken the body's defender cells. A type of helper cell builds spots inside the cancer that block strong killing cells called CD8+ T cells. These killing cells then get too tired to help. The study found a way these bad changes start and showed that blocking them could help. This could lead to better new drugs. More work on this is planned. |
| CancCell | 5 | 10.1016/j.ccell.2025.03.006 | Pub2Post | Immune checkpoint inhibitors (ICIs) have improved outcomes of patients with many different cancers. These antibodies target molecules such as programmed cell death 1 (PD-1) or cytotoxic T lymphocyte associated protein 4 (CTLA-4) which normally function to limit immune activity. Treatment with ICIs reactivates T cells to destroy tumor cells in a highly specific manner, which in some patients, results in dramatic remissions and durable disease control. Over the last decade, much effort has been directed at characterizing factors that drive efficacy and resistance to ICI therapy. Food and Drug Administration (FDA)-approved biomarkers for ICI therapy have facilitated more judicious treatment of cancer patients and transformed the field of precision oncology. Yet, adaptive immunity against cancers is complex, and newer data have revealed the potential utility of other biomarkers. In this review, we discuss the utility of currently approved biomarkers and highlight how emerging biomarkers can further improve the identification of patients who benefit from ICIs. | Background/Introduction:  New treatments called immune checkpoint blockers help the body’s defense system fight cancer. These treatments work by stopping brakes on the immune cells called T cells so they can attack cancer better. Some people with cancer do very well with these drugs. But not all people get the same good results. Experts want to understand who gets the most help from this treatment.  Methods:  Experts looked at many clues, called markers, in the body that may help tell who will do well with these drugs. Some of these markers are already approved by the United States Food and Drug group. The team also studied new markers that are not yet approved but might help in the future.  Findings/Results:  The study found that some markers can already help doctors choose the right people to get these drugs. The new possible markers may make it even easier to find the best people for the treatment. But the way the body fights cancer is hard to understand, and more work is needed.  Conclusion:  Using markers helps doctors choose who may get well with these drugs. While the old markers work well, new ones might make things better. Experts want to keep looking for better ways to help more people.  Short Summary:  This study looked at body clues called markers to help find which people will do best with new cancer drugs that help the immune system. Some markers are already used by doctors, while others are new but may help in the future. The goal is to give the right drug to the right person. More work is needed, but these findings give hope. | Background/Introduction:  Doctors have found new ways to help the body fight cancer. One way is called immune checkpoint inhibitor treatment. This means using special medicine that tells the body’s defense cells to wake up and go after cancer. These drugs stop chemicals in the body that make the defense cells slow or rest. When these defense cells are awake, they can help some people fight off cancer for a long time. Scientists want to know which people will get the most help from this treatment, so they look for special signs in the body, called markers. These signs can help pick the right treatment for each person.  Methods:  Doctors and researchers have been working for many years to understand what helps or blocks this type of cancer care from working. They look at different parts of the body and blood to find these helpful signs. Some markers are now approved for doctors to use, so they can choose the treatment that works best for each cancer patient. Scientists also look at new markers that may work even better. In this review, researchers studied both the old and new signs to see how they help.  Findings/Results:  Some of the approved body signs are useful in some people, but not everyone. New tests and body signs have been found that may point to how well this treatment will work. These new signs might help more people in the future. But body defenses are tricky, and many things can affect how the body reacts.  Conclusion:  Doctors have better tools now to pick the right treatment for cancer using these body signs. The new signs could help even more people. Still, more science work is needed to understand how all of these signs work together. Learning more can help doctors treat cancer better. So, follow-up science work is needed and already planned.  Short Summary:  This study looks at how doctors find the best cancer care for each person using special signs in the body. These signs tell doctors if a person might do well with a drug that wakes up body defense cells. Some signs are already approved and used, but new ones may work better. The study found that these signs can help make cancer care better. But more research is needed to fully understand them and to help more people in the future. | Background/Introduction: Doctors use special drugs to help the body's immune system fight cancer. These drugs are called immune checkpoint blockers. They help the immune system find and kill cancer cells. These drugs have worked well for many people with different types of cancer. But not everyone gets better from them. To try to help more people, scientists look for clues in the body called markers. Markers help doctors guess who might get better with these drugs and who might not.  Methods: Scientists studied past research and reports. They looked into both new and known body signs that help predict who will do well with these cancer drugs. They also talked about how these signs work and how they help doctors choose the best treatment for each person.  Findings/Results: Some signs that were already used worked well in helping pick the right patients. These signs are now used often by doctors. But the body and its fight against cancer are very tricky. So, new signs are also being looked at. These new signs may help spot even more people who will get better with these drugs. This could make the treatment work better for more people.  Conclusion: The way the body fights cancer is hard to understand. Simple signs used today are helpful, but better ones are on the way. These new signs could help doctors treat people more carefully. More work is needed to make sure these new signs are safe and useful. Future studies will help learn more.  Short Summary: Some people with cancer get special drugs that help their bodies fight the sickness. These drugs do not help everyone, so scientists look for signs in the body to know who will get better. Some signs work well and are already used, while new ones are being tested. These signs help doctors give the right care to the right people. There is still more to learn, and more studies will help doctors in the future. |

**Tabel 5s: Comparison of Readability Scores, Indexes and Text Statistics between GAI-Generated Lay Abstracts and Summaries across Three Consecutive Tests, using a “Specific Prompt” in the Generic LLMs.** This table provides a detailed comparison of GAI-generated lay abstracts and summaries (LASs) across three consecutive tests. The analysis encompasses readability scores, readability indexes, and additional text-level statistics, including average sentence length, total word count, syllable count per word, and the proportion of complex words. Readability scores measure how easily a text can be understood, with higher values indicating greater accessibility. In contrast, readability indexes operate inversely, where lower values reflect higher accessibility. Supporting statistics such as sentence length and word complexity offer further insight into the linguistic characteristics of the texts.

|  |  | **Specific Prompt used in:** | | | | |
| --- | --- | --- | --- | --- | --- | --- |
|  |  | **ChatGPT 5** | **Claude** | **Gemeni** | **DeepSeek** | **Grok** |
| **FLESCH KINCAID READING EASE** | **Test 1** | 43.9 (38.9-50.0) | 54.4 (49.3-64.2) | 44.2 (38.1-53.0) | 50.3 (43.8-56.9) | 58.4 (51.9-60.5) |
|  | **Test 2** | 45.5 (39.1-50.4) | 52.6 (46.0-58.7) | 47.0 (40.5-50.6) | 47.4 (38.1-53.7) | 55.6 (51.7-60.8) |
|  | **Test 3** | 47.8 (40.1-52.0) | 53.2 (46.0-61.3) | 45.0 (36.1-51.3) | 45.6 (39.6-52.3) | 53.2 (46.7-58.7) |
|  |  |  |  |  |  |  |
|  | ***P value*** | *0.79* | *0.36* | *0.6* | *0.36* | *0.29* |
|  |  |  |  |  |  |  |
| **FLESCH KINCAID GRADE LEVEL** | **Test 1** | 12.4 (11.3-13.1) | 9.9 (8.9-11.4) | 12.5 (10.8-13.9) | 10.9 (9.6-12.0) | 10.4 (9.5-11.4) |
|  | **Test 2** | 11.9 (11.0-12.9) | 10.4 (9.7-11.4) | 12.1 (11.2-13.1) | 11.4 (10.2-12.5) | 10.8 (9.7-11.1) |
|  | **Test 3** | 12.2 (10.9-13.1) | 10.8 (9.2-12.6) | 12.4 (11.5-13.6) | 11.5 (10.6-12.2) | 11.4 (10.1-11.9) |
|  |  |  |  |  |  |  |
|  | ***P value*** | *0.64* | *0.46* | *0.74* | *0.45* | *0.19* |
|  |  |  |  |  |  |  |
| **GUNNING FOG SCORE** | **Test 1** | 14.4 (13.3-15.3) | 11.4 (10.3-13.4) | 14.9 (13.3-16.5) | 12.7 (11.5-13.5) | 11.9 (11.1-13.3) |
|  | **Test 2** | 13.9 (12.2-15.8) | 12.3 (10.7-13.5) | 14.5 (13.5-15.6) | 13.0 (12.2-14.7) | 12.1 (11.3-13.1) |
|  | **Test 3** | 14.5 (13.0-15.1) | 11.7 (10.7-14.9) | 15.4 (14.6-16.4) | 13.0 (12.4-14.2) | 13.2 (12.2-13.9) |
|  |  |  |  |  |  |  |
|  | ***P value*** | *0.72* | *0.77* | *0.44* | *0.17* | *0.13* |
|  |  |  |  |  |  |  |
| **SMOG INDEX** | **Test 1** | 10.5 (9.5-11.1) | 8.1 (7.2-9.7) | 11.0 (9.5-11.9) | 9.4 (8.0-9.7) | 8.3 (7.5-9.1) |
|  | **Test 2** | 10.1 (8.6-11.1) | 8.8 (7.4-9.7) | 10.3 (9.5-11.1) | 9.5 (8.7-10.5) | 8.3 (7.8-9.3) |
|  | **Test 3** | 10.1 (9.2-10.8) | 8.3 (7.4-10.5) | 11.1 (10.1-11.8) | 9.3 (8.9-10.3) | 9.1 (8.3-9.8) |
|  |  |  |  |  |  |  |
|  | ***P value*** | *0.77* | *0.72* | *0.4* | *0.28* | *0.18* |
|  |  |  |  |  |  |  |
| **COLEMAN LIAU INDEX** | **Test 1** | 15.8 (14.7-16.5) | 14.7 (13.5-15.8) | 14.7 (14.1-15.9) | 15.8 (14.4-16.5) | 14.5 (12.9-15.1) |
|  | **Test 2** | 15.6 (14.8-16.3) | 15.4 (14.3-16.5) | 15.2 (13.8-16.3) | 16.1 (14.6-17.1) | 13.6 (13.2-14.9) |
|  | **Test 3** | 15.2 (14.4-16.7) | 14.7 (13.8-17.0) | 15.1 (14.3-16.2) | 16.1 (14.8-16.7) | 14.4 (13.5-15.2) |
|  |  |  |  |  |  |  |
|  | ***P value*** | *0.81* | *0.54* | *0.87* | *0.58* | *0.51* |
|  |  |  |  |  |  |  |
| **AUTOMATED READABILITY INDEX** | **Test 1** | 14.3 (13.2-15.5) | 12.1 (11.0-13.4) | 14.2 (12.9-15.6) | 12.8 (12.4-13.6) | 12.6 (11.7-14.2) |
|  | **Test 2** | 13.5 (12.9-15.1) | 12.8 (11.5-13.8) | 14.1 (12.7-15.3) | 13.4 (11.8-14.3) | 12.7 (11.4-13.3) |
|  | **Test 3** | 14.1 (13.1-15.4) | 13.5 (10.9-14.8) | 14.3 (13.1-15.5) | 13.1 (12.1-14.3) | 13.7 (12.4-14.4) |
|  |  |  |  |  |  |  |
|  | ***P value*** | *0.52* | *0.47* | *0.9* | *0.52* | *0.13* |
|  |  |  |  |  |  |  |
| **SENTENCES** | **Test 1** | 6.0 (6.0–7.0) | 9.0 (8.0–10.0) | 6.0 (5.0–6.0) | 6.0 (6.0–7.0) | 6.0 (6.0–7.0) |
|  | **Test 2** | 7.0 (6.0–7.0) | 8.0 (8.0–9.0) | 6.0 (5.0–7.0) | 6.0 (6.0–7.0) | 6.0 (6.0–7.0) |
|  | **Test 3** | 6.0 (6.0–7.0) | 8.0 (7.0–9.0) | 6.0 (5.0–6.0) | 6.0 (6.0–7.0) | 6.0 (5.0–7.0) |
|  |  |  |  |  |  |  |
|  | ***P value*** | *0.13* | *0.53* | *0.76* | *0.99* | *0.19* |
|  |  |  |  |  |  |  |
| **WORDS** | **Test 1** | 136.0 (132.0–140.0) | 159.0 (155.0–169.0) | 122.0 (109.0–128.0) | 118.0 (109.0–126.0) | 129.0 (120.0–138.0) |
|  | **Test 2** | 137.0 (134.0–139.0) | 159.0 (151.0–164.0) | 128.0 (112.0–135.0) | 122.0 (112.0–124.0) | 125.0 (121.0–140.0) |
|  | **Test 3** | 136.0 (130.0–141.0) | 161.0 (156.0–168.0) | 122.0 (114.0–138.0) | 119.0 (112.0–131.0) | 125.0 (115.0–134.0) |
|  |  |  |  |  |  |  |
|  | ***P value*** | *0.98* | *0.47* | *0.49* | *0.89* | *0.81* |
|  |  |  |  |  |  |  |
| **COMPLEX WORDS** | **Test 1** | 20.0 (17.0–24.0) | 17.0 (15.0–22.0) | 19.0 (16.0–21.0) | 15.0 (14.0–17.0) | 13.0 (11.0–15.0) |
|  | **Test 2** | 20.0 (15.0–23.0) | 18.0 (14.0–23.0) | 19.0 (15.0–22.0) | 17.0 (14.0–22.0) | 13.0 (11.0–16.0) |
|  | **Test 3** | 19.0 (16.0–21.0) | 18.0 (15.0–24.0) | 20.0 (17.0–25.0) | 18.0 (14.0–22.0) | 14.0 (12.0–16.0) |
|  |  |  |  |  |  |  |
|  | ***P value*** | *0.7* | *0.85* | *0.2* | *0.16* | *0.67* |
|  |  |  |  |  |  |  |
| **% OF COMPLEX WORD** | **Test 1** | 15.0 (12.6–17.2) | 11.3 (8.8–13.9) | 13.8 (13.3–18.6) | 13.6 (11.8–15.6) | 10.5 (8.3–11.9) |
|  | **Test 2** | 15.1 (11.6–16.8) | 11.9 (8.4–14.8) | 14.6 (12.6–17.2) | 14.8 (12.2–18.6) | 10.5 (8.5–12.2) |
|  | **Test 3** | 13.1 (11.9–15.8) | 11.5 (9.1–15.1) | 16.8 (13.6–19.2) | 15.9 (12.3–16.8) | 11.7 (9.4–12.8) |
|  |  |  |  |  |  |  |
|  | ***P value*** | *0.64* | *0.7* | *0.34* | *0.19* | *0.49* |
|  |  |  |  |  |  |  |
| **AVERAGE WORDS PER SENTENCE** | **Test 1** | 20.4 (19.0–23.0) | 18.6 (16.9–19.9) | 21.0 (20.3–24.4) | 18.6 (16.5–20.2) | 20.3 (18.2–23.3) |
|  | **Test 2** | 19.7 (18.9–22.3) | 18.9 (16.1–20.3) | 22.0 (19.5–24.4) | 18.8 (16.0–20.6) | 20.5 (18.6–21.7) |
|  | **Test 3** | 21.5 (20.1–22.7) | 19.1 (18.1–22.4) | 21.4 (20.2–24.0) | 18.5 (16.3–20.2) | 20.8 (19.5–23.0) |
|  |  |  |  |  |  |  |
|  | ***P value*** | *0.17* | *0.43* | *0.97* | *0.99* | *0.41* |
|  |  |  |  |  |  |  |
| **AVERAGE SYLLABLES PER WORD** | **Test 1** | 1.67 (1.59–1.72) | 1.58 (1.48–1.62) | 1.65 (1.57–1.72) | 1.63 (1.57–1.71) | 1.53 (1.47–1.58) |
|  | **Test 2** | 1.65 (1.61–1.71) | 1.58 (1.53–1.67) | 1.64 (1.59–1.71) | 1.69 (1.59–1.75) | 1.53 (1.49–1.59) |
|  | **Test 3** | 1.64 (1.57–1.70) | 1.59 (1.52–1.66) | 1.66 (1.58–1.74) | 1.67 (1.60–1.76) | 1.57 (1.51–1.62) |
|  |  |  |  |  |  |  |
|  | ***P value*** | *0.75* | *0.38* | *0.6* | *0.44* | *0.5* |

**Tabel 6s: Comparison of Readability Scores, Indexes and Text Statistics between GAI-Generated Lay Abstracts and Summaries across Three Consecutive Tests, using a “Generic Prompt” in the Generic LLMs.** This table provides a detailed comparison of GAI-generated lay abstracts and summaries (LASs) across three consecutive tests. The analysis encompasses readability scores, readability indexes, and additional text-level statistics, including average sentence length, total word count, syllable count per word, and the proportion of complex words. Readability scores measure how easily a text can be understood, with higher values indicating greater accessibility. In contrast, readability indexes operate inversely, where lower values reflect higher accessibility. Supporting statistics such as sentence length and word complexity offer further insight into the linguistic characteristics of the texts.

|  |  | **Generic Prompt used in:** | | | | |
| --- | --- | --- | --- | --- | --- | --- |
|  |  | **ChatGPT 5** | **Claude** | **Gemeni** | **DeepSeek** | **Grok** |
| **FLESCH KINCAID READING EASE** | **Test 1** | 41.2 (31.7-46.2) | 38.3 (33.8-42.3) | 41.0 (35.4-46.9) | 36.0 (28.0-42.2) | 35.9 (30.3-40.5) |
|  | **Test 2** | 41.0 (34.5-47.5) | 37.4 (33.2-46.4) | 44.5 (38.8-49.4) | 35.3 (28.5-45.2) | 36.4 (24.2-40.7) |
|  | **Test 3** | 40.5 (32.4-49.4) | 37.5 (31.6-43.0) | 42.1 (33.2-51.2) | 36.4 (30.0-49.3) | 30.4 (27.5-36.9) |
|  |  |  |  |  |  |  |
|  | ***P value*** | *0.9* | *0.86* | *0.37* | *0.89* | *0.32* |
|  |  |  |  |  |  |  |
| **FLESCH KINCAID GRADE LEVEL** | **Test 1** | 12.3 (11.8-14.7) | 13.6 (13.0-15.1) | 12.9 (11.8-13.5) | 13.6 (11.1-14.1) | 13.9 (13.1-14.5) |
|  | **Test 2** | 12.9 (11.6-14.2) | 14.5 (13.5-15.2) | 12.0 (11.6-13.0) | 12.8 (10.8-14.2) | 14.2 (13.2-15.9) |
|  | **Test 3** | 12.7 (11.3-14.2) | 14.3 (13.2-15.1) | 12.7 (11.4-14.7) | 12.7 (11.1-13.9) | 15.1 (13.6-16.5) |
|  |  |  |  |  |  |  |
|  | ***P value*** | *0.99* | *0.61* | *0.47* | *0.81* | *0.14* |
|  |  |  |  |  |  |  |
| **GUNNING FOG SCORE** | **Test 1** | 14.0 (13.4-16.8) | 16.2 (15.0-17.0) | 15.1 (14.3-16.4) | 15.4 (13.8-17.0) | 16.3 (15.5-16.9) |
|  | **Test 2** | 15.8 (13.0-16.3) | 16.7 (15.6-18.2) | 14.8 (14.0-15.7) | 14.7 (13.6-17.7) | 16.6 (15.7-18.2) |
|  | **Test 3** | 14.1 (13.2-16.5) | 16.9 (15.3-18.2) | 15.0 (13.7-17.4) | 15.1 (13.4-16.6) | 18.0 (16.2-18.9) |
|  |  |  |  |  |  |  |
|  | ***P value*** | *0.99* | *0.58* | *0.77* | *0.79* | *0.06* |
|  |  |  |  |  |  |  |
| **SMOG INDEX** | **Test 1** | 10.6 (10.1-11.9) | 11.6 (11.0-12.3) | 11.0 (10.1-11.9) | 11.2 (10.6-12.3) | 11.6 (11.0-12.6) |
|  | **Test 2** | 11.1 (9.6-11.6) | 11.9 (10.4-12.9) | 10.7 (10.1-11.6) | 10.5 (9.8-12.6) | 11.9 (11.2-12.5) |
|  | **Test 3** | 10.5 (9.6-11.9) | 12.0 (11.2-12.9) | 10.5 (9.7-12.9) | 10.8 (9.7-11.9) | 12.6 (11.4-13.5) |
|  |  |  |  |  |  |  |
|  | ***P value*** | *0.99* | *0.55* | *0.65* | *0.76* | *0.17* |
|  |  |  |  |  |  |  |
| **COLEMAN LIAU INDEX** | **Test 1** | 16.3 (15.6-17.4) | 16.6 (15.8-17.1) | 15.6 (14.0-16.2) | 16.8 (15.6-18.0) | 16.1 (15.0-17.1) |
|  | **Test 2** | 15.6 (14.8-17.8) | 16.3 (14.8-17.5) | 15.0 (14.2-15.7) | 17.4 (15.8-18.3) | 16.3 (14.7-17.2) |
|  | **Test 3** | 16.4 (15.4-16.8) | 16.4 (15.2-17.9) | 14.9 (13.9-15.8) | 17.2 (14.9-18.1) | 15.9 (14.8-17.3) |
|  |  |  |  |  |  |  |
|  | ***P value*** | *0.88* | *0.55* | *0.43* | *0.87* | *0.97* |
|  |  |  |  |  |  |  |
| **AUTOMATED READABILITY INDEX** | **Test 1** | 14.2 (12.9-15.8) | 16.0 (15.0-17.8) | 13.7 (13.3-15.4) | 15.4 (11.9-15.8) | 15.4 (13.9-17.1) |
|  | **Test 2** | 14.2 (13.4-16.6) | 16.8 (15.8-18.5) | 13.3 (12.8-15.4) | 15.2 (11.4-16.3) | 16.0 (14.5-17.6) |
|  | **Test 3** | 15.0 (13.5-15.9) | 16.6 (15.0-18.6) | 14.1 (12.6-15.9) | 14.2 (12.8-15.4) | 16.9 (15.4-18.7) |
|  |  |  |  |  |  |  |
|  | ***P value*** | *0.88* | *0.59* | *0.68* | *0.86* | *0.28* |
|  |  |  |  |  |  |  |
| **SENTENCES** | **Test 1** | 4.0 (3.0-4.0) | 5.0 (4.0-6.0) | 5.0 (4.0-5.0) | 5.0 (4.0-5.0) | 4.0 (4.0-5.0) |
|  | **Test 2** | 4.0 (3.0-4.0) | 4.0 (4.0-5.0) | 5.0 (4.0-5.0) | 4.0 (4.0-5.0) | 4.0 (4.0-5.0) |
|  | **Test 3** | 4.0 (3.0-4.0) | 4.0 (4.0-5.0) | 5.0 (4.0-5.0) | 5.0 (4.0-5.0) | 4.0 (3.0-4.0) |
|  |  |  |  |  |  |  |
|  | ***P value*** | *0.99* | *0.44* | *0.98* | *0.92* | *0.41* |
|  |  |  |  |  |  |  |
| **WORDS** | **Test 1** | 80.0 (75.0-88.0) | 110.0 (96.0-122.0) | 101.0 (91.0-116.0) | 88.0 (77.0-97.0) | 101.0 (94.0-110.0) |
|  | **Test 2** | 81.0 (76.0-86.0) | 110.0 (94.0-120.0) | 104.0 (95.0-110.0) | 84.0 (78.0-94.0) | 99.0 (89.0-111.0) |
|  | **Test 3** | 84.0 (79.0-86.0) | 109.0 (102.0-120.0) | 105.0 (91.0-117.0) | 87.0 (82.0-94.0) | 105.0 (96.0-109.0) |
|  |  |  |  |  |  |  |
|  | ***P value*** | *0.65* | *0.94* | *0.94* | *0.84* | *0.9* |
|  |  |  |  |  |  |  |
| **COMPLEX WORDS** | **Test 1** | 14.0 (12.0-16.0) | 19.0 (16.0-21.0) | 18.0 (15.0-21.0) | 17.0 (14.0-19.0) | 19.0 (16.0-22.0) |
|  | **Test 2** | 14.0 (10.0-17.0) | 18.0 (15.0-20.0) | 17.0 (13.0-20.0) | 17.0 (15.0-20.0) | 18.0 (16.0-21.0) |
|  | **Test 3** | 13.0 (12.0-17.0) | 19.0 (17.0-22.0) | 17.0 (13.0-22.0) | 16.0 (13.0-19.0) | 19.0 (16.0-22.0) |
|  |  |  |  |  |  |  |
|  | ***P value*** | *0.92* | *0.4* | *0.57* | *0.75* | *0.76* |
|  |  |  |  |  |  |  |
| **% OF COMPLEX WORD** | **Test 1** | 17.1 (14.9-18.4) | 16.8 (15.7-19.1) | 17.0 (15.5-20.8) | 18.9 (16.7-22.5) | 18.2 (15.7-21.7) |
|  | **Test 2** | 16.5 (13.6-21.6) | 16.8 (13.3-18.9) | 17.2 (14.7-19.2) | 19.6 (17.8-23.2) | 18.0 (16.0-23.7) |
|  | **Test 3** | 16.0 (12.9-22.0) | 17.3 (15.3-20.0) | 16.2 (12.7-19.5) | 18.5 (16.3-22.0) | 18.6 (16.5-23.3) |
|  |  |  |  |  |  |  |
|  | ***P value*** | *0.84* | *0.65* | *0.45* | *0.69* | *0.68* |
|  |  |  |  |  |  |  |
| **AVERAGE WORDS PER SENTENCE** | **Test 1** | 20.6 (18.0-24.0) | 23.3 (22.0-25.2) | 22.0 (19.4-23.8) | 18.5 (17.2-22.5) | 24.0 (19.3-25.5) |
|  | **Test 2** | 21.0 (19.0-23.0) | 26.8 (22.5-28.3) | 21.2 (19.0-23.3) | 18.8 (16.6-20.5) | 24.8 (19.2-27.8) |
|  | **Test 3** | 21.0 (19.8-23.5) | 23.8 (20.5-28.3) | 21.8 (19.6-23.7) | 18.4 (16.6-21.8) | 26.0 (20.3-29.5) |
|  |  |  |  |  |  |  |
|  | ***P value*** | *0.84* | *0.4* | *0.8* | *0.96* | *0.4* |
|  |  |  |  |  |  |  |
| **AVERAGE SYLLABLES PER WORD** | **Test 1** | 1.69 (1.67-1.84) | 1.72 (1.64-1.78) | 1.70 (1.62-1.80) | 1.76 (1.66-1.85) | 1.75 (1.67-1.81) |
|  | **Test 2** | 1.68 (1.59-1.81) | 1.69 (1.62-1.74) | 1.67 (1.59-1.71) | 1.78 (1.68-1.88) | 1.77 (1.65-1.88) |
|  | **Test 3** | 1.71 (1.62-1.81) | 1.70 (1.63-1.76) | 1.68 (1.59-1.76) | 1.75 (1.66-1.85) | 1.76 (1.69-1.86) |
|  |  |  |  |  |  |  |
|  | ***P value*** | *0.69* | *0.7* | *0.34* | *0.89* | *0.78* |

**Table 7s: Comparison of Readability Scores, Indexes and Text Statistics between GAI-Generated Lay Abstracts and Summaries across Three Consecutive Tests, using a task-specific LLM powered tool (Pub2post).** This table provides a detailed comparison of GAI-generated lay abstracts and summaries (LASs) across three consecutive tests. The analysis encompasses readability scores, readability indexes, and additional text-level statistics, including average sentence length, total word count, syllable count per word, and the proportion of complex words. Readability scores measure how easily a text can be understood, with higher values indicating greater accessibility. In contrast, readability indexes operate inversely, where lower values reflect higher accessibility. Supporting statistics such as sentence length and word complexity offer further insight into the linguistic characteristics of the texts.

|  | **Test 1** | **Test 2** | **Test 3** | ***P value*** |
| --- | --- | --- | --- | --- |
| **Readability Metrics** | | | | |
| Flesch Kincaid Reading Ease | 78.8 (76.8-81.3) | 78.2 (76.8-79.9) | 80.4 (76.4-82.3) | *0.35* |
| Flesch Kincaid Grade Level | 5.7 (5.4-6.2) | 6.0 (5.6-6.7) | 5.7 (5.3-6.4) | *0.3* |
| Gunning Fog Score | 7.2 (6.6-7.6) | 7.5 (7.1-8.0) | 7.4 (6.4-7.6) | *0.16* |
| Smog Index | 4.9 (4.4-5.5) | 5.0 (4.6-5.6) | 4.7 (4.2-5.2) | *0.44* |
| Coleman Liau Index | 10.7 (10.3-11.5) | 10.7 (10.3-11.5) | 10.6 (10.2-11.4) | *0.87* |
| Automated Readability Index | 7.0 (6.1-7.6) | 7.2 (6.7-8.1) | 7.0 (6.4-7.4) | *0.42* |
| **Text Metrics** | | | | |
| Sentences | 26.0 (24.0–29.0) | 27.0 (24.0–29.0) | 26.0 (24.0–29.0) | *0.95* |
| Words | 383.0 (340.0–408.0) | 394.0 (378.0–418.0) | 365.0 (337.0–418.0) | *0.26* |
| Complex Words | 17.0 (12.0–23.0) | 18.0 (15.0–22.0) | 15.0 (12.0–19.0) | *0.33* |
| % Of Complex Word | 4.4 (3.6–5.9) | 4.4 (3.9–5.7) | 4.1 (3.3–4.9) | *0.48* |
| Average Words Per Sentence | 14.0 (13.4–15.0) | 15.0 (14.0–15.3) | 14.4 (13.5–15.2) | *0.24* |
| Average Syllables Per Word | 1.34 (1.31–1.36) | 1.34 (1.32–1.38) | 1.32 (1.31–1.37) | *0.58* |

***Table 8s. Comparison* of Readability Scores, Indexes and Text Statistics between GAI-Generated Lay Abstracts and Summaries Generated by Task Specific vs Generic LLMs (using a “Specific Prompt” in the Generic LLMs).**

|  | **Pub2Post** | **Specific Prompt used in:** | | | | | **P-VALUE** |
| --- | --- | --- | --- | --- | --- | --- | --- |
|  |  | **ChatGPT 5** | **Claude** | **Gemeni** | **DeepSeek** | **Grok** |  |
| **Readability scores and indexes** | | | | | | | |
| Flesch Kincaid Reading Ease | 82.3 (80.0-83.5) | 50.1 (44.9-54.4) | 58.0 (53.4-66.0) | 53.0 (45.6-55.5) | 52.9 (43.8-57.1) | 61.6 (56.1-64.4) | <.0001 |
| Flesch Kincaid Grade Level | 5.2 (4.9-5.5) | 11.4 (10.4-12.2) | 9.6 (8.8-10.4) | 11.3 (10.6-12.4) | 10.2 (9.6-11.5) | 9.6 (8.9-10.2) | <.0001 |
| Gunning Fog Score | 6.6 (6.3-6.8) | 12.8 (12.1-14.4) | 10.6 (10.2-12.4) | 13.8 (13.3-14.6) | 12.2 (11.0-12.9) | 11.2 (10.3-12.3) | <.0001 |
| Smog Index | 4.4 (4.1-4.7) | 8.8 (8.6- 10.1) | 7.6 (6.6-8.8) | 9.8 (9.2-10.4) | 8.7 (8.0-9.4) | 7.7 (7.1-8.3) | <.0001 |
| Coleman Liau Index | 10.4 (9.9-10.7) | 15.1 (14.4-15.9) | 14.3 (13.3-15.3) | 14.3 (13.6-15.5) | 15.3 (14.0-16.8) | 13.4 (12.6-14.0) | <.0001 |
| Automated Readability Index | 6.2 (5.9-6.8) | 13.3 (12.8-14.1) | 11.0 (10.6-12.5) | 13.1 (12.3-14.2) | 12.5 (11.4-13.4) | 11.8 (10.7-12.5) | <.0001 |
| **Text Metrics** | | | | | | | |
| Sentences | 27.0 (26.0-31.0) | 7.0 (6.0- 7.0) | 9.0 (8.0-10.0) | 6.0 (5.0-6.0) | 7.0 (6.0-8.0) | 7.0 (6.0-7.0) | <.0001 |
| Words | 388.0 (340.0-414.0) | 136.0 (129.0-140.0) | 156.0 (152.0-164.0) | 124.0 (114.0-133.0) | 118.0 (112.0-127.0) | 132.0 (122.0-138.0) | <.0001 |
| Complex Words | 14.0 (11.0-17.0) | 16.0 (15.0-20.0) | 15.0 (11.0-18.0) | 16.0 (15.0-19.0) | 15.0 (13.0-16.0) | 12.0 (9.0-16.0) | 0.0051 |
| % Of Complex Word | 3.7 (3.1-4.2) | 11.9 (10.9-15.0) | 9.7 (7.6-11.6) | 13.5 (11.3-15.6) | 12.0 (11.4-13.7) | 8.8 (7.4-11.9) | <.0001 |
| Average Words Per Sentence | 13.5 (12.8-14.2) | 20.0 (19.1-21.5) | 18.6 (16.9-19.6) | 21.0 (20.0-22.2) | 18.0 (15.6-19.5) | 19.0 (17.5-20.8) | <.0001 |
| Average Syllables Per Word | 1.31 (1.29-1.33) | 1.62 (1.55-1.66) | 1.53 (1.48-1.58) | 1.59 (1.55-1.64) | 1.62 (1.54-1.71) | 1.50 (1.47-1.53) | <.0001 |

***Table 9s. Comparison* of Readability Scores, Indexes and Text Statistics between GAI-Generated Lay Abstracts and Summaries Generated by Task Specific vs Generic LLMs (using a “Generic Prompt” in the Generic LLMs).**

|  | **Pub2Post** | **Generic Prompt used in:** | | | | | **P-VALUE** |
| --- | --- | --- | --- | --- | --- | --- | --- |
|  |  | **ChatGPT 5** | **Claude** | **Gemeni** | **DeepSeek** | **Grok** |  |
| **Readability scores and indexes** | | | | | | | |
| Flesch Kincaid Reading Ease | 82.3 (80.0-83.5) | 47.5 (39.2-50.6) | 40.6 (38.1-46.6) | 47.1 (41.0-54.0) | 38.9 (33.3-49.3) | 38.0 (34.6-42.5) | <.0001 |
| Flesch Kincaid Grade Level | 5.2 (4.9-5.5) | 11.8 (11.2-12.7) | 13.2 (12.5-14.0) | 11.8 (10.5-12.3) | 12.3 (10.5-12.9) | 13.4 (12.8-14.2) | <.0001 |
| Gunning Fog Score | 6.6 (6.3-6.8) | 13.7 (12.7-14.9) | 15.6 (14.4-16.4) | 14.1 (12.7-14.8) | 13.9 (12.9-15.5) | 15.9 (14.8-16.3) | <.0001 |
| Smog Index | 4.4 (4.1-4.7) | 10.1 (9.0-11.0) | 11.2 (9.5-11.9) | 9.7 (9.0-10.6) | 10.3 (9.2-11.3) | 11.2 (10.7-11.6) | <.0001 |
| Coleman Liau Index | 10.4 (9.9-10.7) | 15.9 (15.1-16.8) | 15.9 (15.2-16.9) | 14.2 (13.8-15.9) | 17.0 (15.3-18.1) | 16.0 (14.7-16.9) | <.0001 |
| Automated Readability Index | 6.2 (5.9-6.8) | 14.1 (13.0-14.7) | 15.2 (14.5-17.4) | 12.9 (11.7-13.7) | 13.5 (11.3-15.2) | 15.2 (13.5-16.3) | <.0001 |
| **Text Metrics** | | | | | | | |
| Sentences | 27.0 (26.0-31.0) | 4.0 (4.0-5.0) | 5.0 (4.0-5.0) | 5.0 (5.0-6.0) | 5.0 (4.0-5.0) | 4.0 (4.0-6.0) | <.0001 |
| Words | 388.0 (340.0-414.0) | 81.0 (76.0-86.0) | 108.0 (101.0-118.0) | 106.0 (92.0-117.0) | 83.0 (77.0-90.0) | 101.0 (97.0-114.0) | <.0001 |
| Complex Words | 14.0 (11.0-17.0) | 12.0 (10.0-16.0) | 17.0 (14.0-19.0) | 15.0 (12.0-19.0) | 15.0 (13.0-17.0) | 16.0 (15.0-22.0) | 0.004 |
| % Of Complex Word | 3.7 (3.1-4.2) | 15.2 (12.4-18.4) | 16.1 (11.7-17.8) | 14.7 (12.6-17.8) | 18.1 (14.8-19.7) | 16.8 (14.9-20.2) | <.0001 |
| Average Words Per Sentence | 13.5 (12.8-14.2) | 20.0 (18.5-21.5) | 23.2 (21.6-25.8) | 19.3 (18.4-21.8) | 17.8 (16.6-19.4) | 24.0 (18.0-25.5) | <.0001 |
| Average Syllables Per Word | 1.31 (1.29-1.33) | 1.66 (1.59-1.75) | 1.66 (1.59-1.72) | 1.67 (1.57-1.72) | 1.74 (1.64-1.81) | 1.72 (1.65-1.81) | <.0001 |

Table 10s. ***Comparison of Task specific vs General-purpose LLM Best Tests using the specific prompt***

| Metric | Pub2Post vs. Comparator | Mean difference | 95% CI Low | 95% CI High | % Difference | p_value |
| --- | --- | --- | --- | --- | --- | --- |
| **FRES** | OpenAI GPT-5 | 32.5 | 29.229581 | 35.698419 | 39.8% | <.0001 |
|  | Claude | 23.2 | 20.260194 | 26.139806 | 28.4% | <.0001 |
|  | Gemini | 31.1 | 27.9633951 | 34.2686049 | 38.0% | <.0001 |
|  | DeepSeek | 30.4 | 27.4748923 | 33.3491077 | 37.3% | <.0001 |
|  | Grok | 21.7 | 19.4434114 | 23.9645886 | 26.5% | <.0001 |
| **FKGL** | OpenAI GPT-5 | -6.1 | -6.7303121 | -5.5656879 | -119.5% | <.0001 |
|  | Claude | -4.4 | -5.0747087 | -3.7812913 | -86.3% | <.0001 |
|  | Gemini | -6.3 | -6.8794005 | -5.6885995 | -122.3% | <.0001 |
|  | DeepSeek | -5.3 | -5.7998889 | -4.7041111 | -102.2% | <.0001 |
|  | Grok | -4.4 | -4.866875 | -3.989125 | -85.9% | <.0001 |
| **GFS** | OpenAI GPT-5 | -6.4 | -7.0647479 | -5.7992521 | -98.3% | <.0001 |
|  | Claude | -4.5 | -5.2807842 | -3.7352158 | -68.7% | <.0001 |
|  | Gemini | -7.3 | -7.8921452 | -6.6198548 | -110.9% | <.0001 |
|  | DeepSeek | -5.4 | -5.9546841 | -4.8773159 | -83.1% | <.0001 |
|  | Grok | -4.8 | -5.2417565 | -4.2942435 | -72.5% | <.0001 |
| **SI** | OpenAI GPT-5 | -4.9 | -5.3500555 | -4.3619445 | -113.1% | <.0001 |
|  | Claude | -3.4 | -3.9549307 | -2.8130693 | -78.4% | <.0001 |
|  | Gemini | -5.3 | -5.8490485 | -4.8309515 | -124.5% | <.0001 |
|  | DeepSeek | -4.2 | -4.6419957 | -3.8300043 | -99.4% | <.0001 |
|  | Grok | -3.4 | -3.8315779 | -2.9124221 | -78.3% | <.0001 |
| **CLI** | OpenAI GPT-5 | -4.8 | -5.3706184 | -4.2133816 | -46.4% | <.0001 |
|  | Claude | -3.7 | -4.2229074 | -3.2090926 | -35.9% | <.0001 |
|  | Gemini | -3.9 | -4.5888604 | -3.2751396 | -38.2% | <.0001 |
|  | DeepSeek | -5 | -5.6055543 | -4.3304457 | -48.1% | <.0001 |
|  | Grok | -3 | -3.5650375 | -2.4269625 | -29.2% | <.0001 |
| **ARI** | OpenAI GPT-5 | -7.1 | -7.8648111 | -6.3591889 | -115.3% | <.0001 |
|  | Claude | -5.4 | -6.2088207 | -4.5751793 | -87.2% | <.0001 |
|  | Gemini | -7.1 | -7.8366701 | -6.3633299 | -114.8% | <.0001 |
|  | DeepSeek | -6 | -6.7410185 | -5.3149815 | -97.9% | <.0001 |
|  | Grok | -5.3 | -5.8526392 | -4.6593608 | -84.5% | <.0001 |
| **SENTENCES** | OpenAI GPT-5 | 21.2 | 19.9750627 | 22.8249373 | 75.5% | <.0001 |
|  | Claude | 19.4 | 17.9370812 | 20.9429188 | 68.5% | <.0001 |
|  | Gemini | 22.3 | 20.8979583 | 23.6620417 | 78.7% | <.0001 |
|  | DeepSeek | 21.2 | 19.7792419 | 22.7007581 | 75.0% | <.0001 |
|  | Grok | 21.2 | 19.7841101 | 22.6958899 | 75.0% | <.0001 |
| **WORDS** | OpenAI GPT-5 | 246.9 | 226.902695 | 266.937305 | 64.2% | <.0001 |
|  | Claude | 224 | 203.15769 | 244.84231 | 58.0% | <.0001 |
|  | Gemini | 256 | 234.683814 | 277.316186 | 66.6% | <.0001 |
|  | DeepSeek | 263 | 243.821498 | 282.098502 | 68.5% | <.0001 |
|  | Grok | 251 | 231.060236 | 270.859764 | 65.3% | <.0001 |
| **COMPLEX_WORDS** | OpenAI GPT-5 | -3.1 | -5.2157126 | -1.0242874 | -33.6% | 0.0052 |
|  | Claude | -1.3 | -3.4238271 | 0.7838271 | -16.2% | 0.2077 |
|  | Gemini | -2.8 | -5.5587358 | -0.0412642 | -32.9% | 0.0469 |
|  | DeepSeek | -1 | -2.8916901 | 0.8116901 | -17.0% | 0.2578 |
|  | Grok | 1.7 | -0.229227 | 3.589227 | 5.6% | 0.0819 |
| **Percent_complex** | OpenAI GPT-5 | -9 | -10.307638 | -7.7667616 | -280.3% | <.0001 |
|  | Claude | -6 | -7.1969335 | -4.8542665 | -181.7% | <.0001 |
|  | Gemini | -9.6 | -10.922696 | -8.364504 | -297.3% | <.0001 |
|  | DeepSeek | -9 | -10.113255 | -7.9459448 | -277.6% | <.0001 |
|  | Grok | -5.8 | -6.8953575 | -4.6342425 | -177.9% | <.0001 |
| **AVE_wordsPerSen** | OpenAI GPT-5 | -6.5 | -7.6482401 | -5.4421599 | -49.4% | <.0001 |
|  | Claude | -4.8 | -6.0027054 | -3.6276946 | -36.3% | <.0001 |
|  | Gemini | -7.9 | -9.0133171 | -6.7690829 | -58.9% | <.0001 |
|  | DeepSeek | -4.1 | -5.4105511 | -2.7462489 | -31.0% | <.0001 |
|  | Grok | -5.7 | -6.9145804 | -4.4894196 | -42.7% | <.0001 |
| **AVE_sylPer_word** | OpenAI GPT-5 | -0.31 | -0.3425601 | -0.2686399 | -23.2% | <.0001 |
|  | Claude | -0.22 | -0.2445342 | -0.1890658 | -16.5% | <.0001 |
|  | Gemini | -0.27 | -0.3087849 | -0.2384151 | -20.9% | <.0001 |
|  | DeepSeek | -0.31 | -0.3487058 | -0.2744942 | -23.7% | <.0001 |
|  | Grok | -0.2 | -0.2189056 | -0.1586944 | -14.4% | <.0001 |

Table 11s. ***Comparison of Task specific vs General-purpose LLM Best Tests using the generic prompt***

|  | **Pub2Post vs. Comparator** | **Mean difference** | **95% CI Low** | **95% CI High** | **% Difference** | ***p_value*** |
| --- | --- | --- | --- | --- | --- | --- |
| **FRES** | **OpenAI GPT-5** | 36.3 | 32.5 | 40.0 | 44.3% | *<0.0001* |
|  | **Claude** | 40.0 | 37.3 | 42.7 | 49.0% | *<0.0001* |
|  | **Gemini** | 34.0 | 30.2 | 37.8 | 41.6% | *<0.0001* |
|  | **DeepSeek** | 39.5 | 34.4 | 44.5 | 48.2% | *<0.0001* |
|  | **Grok** | 43.0 | 39.3 | 46.7 | 52.6% | *<0.0001* |
| **FKGL** | **OpenAI GPT-5** | -6.6 | -7.3 | -5.9 | -127.8% | *<0.0001* |
|  | **Claude** | -8.0 | -8.7 | -7.3 | -155.6% | *<0.0001* |
|  | **Gemini** | -6.3 | -7.0 | -5.7 | -123.0% | *<0.0001* |
|  | **DeepSeek** | -6.6 | -7.6 | -5.7 | -130.5% | *<0.0001* |
|  | **Grok** | -8.1 | -8.9 | -7.4 | -158.1% | *<0.0001* |
| **GFS** | **OpenAI GPT-5** | -7.2 | -8.0 | -6.4 | -110.2% | *<0.0001* |
|  | **Claude** | -8.8 | -9.6 | -7.9 | -134.3% | *<0.0001* |
|  | **Gemini** | -7.3 | -8.0 | -6.6 | -111.1% | *<0.0001* |
|  | **DeepSeek** | -7.6 | -8.5 | -6.6 | -115.9% | *<0.0001* |
|  | **Grok** | -9.0 | -9.8 | -8.2 | -138.2% | *<0.0001* |
| **SI** | **OpenAI GPT-5** | -5.6 | -6.3 | -5.0 | -131.8% | *<0.0001* |
|  | **Claude** | -6.4 | -7.0 | -5.8 | -148.6% | *<0.0001* |
|  | **Gemini** | -5.6 | -6.1 | -5.1 | -128.8% | *<0.0001* |
|  | **DeepSeek** | -5.9 | -6.5 | -5.2 | -137.0% | *<0.0001* |
|  | **Grok** | -6.8 | -7.3 | -6.2 | -158.6% | *<0.0001* |
| **CLI** | **OpenAI GPT-5** | -5.4 | -6.1 | -4.8 | -52.5% | *<0.0001* |
|  | **Claude** | -5.6 | -6.1 | -5.1 | -53.8% | *<0.0001* |
|  | **Gemini** | -4.1 | -4.8 | -3.4 | -39.9% | *<0.0001* |
|  | **DeepSeek** | -5.9 | -6.8 | -4.9 | -57.0% | *<0.0001* |
|  | **Grok** | -5.3 | -6.1 | -4.5 | -51.1% | *<0.0001* |
| **ARI** | **OpenAI GPT-5** | -7.4 | -8.3 | -6.5 | -119.2% | *<0.0001* |
|  | **Claude** | -9.3 | -10.3 | -8.3 | -151.1% | *<0.0001* |
|  | **Gemini** | -6.5 | -7.3 | -5.6 | -105.8% | *<0.0001* |
|  | **DeepSeek** | -7.0 | -8.3 | -5.8 | -114.9% | *<0.0001* |
|  | **Grok** | -8.6 | -9.6 | -7.5 | -138.6% | *<0.0001* |
| **SENTENCES** | **OpenAI GPT-5** | 23.9 | 22.6 | 25.2 | 84.6% | *<0.0001* |
|  | **Claude** | 23.3 | 21.9 | 24.7 | 82.4% | *<0.0001* |
|  | **Gemini** | 22.8 | 21.4 | 24.2 | 80.6% | *<0.0001* |
|  | **DeepSeek** | 23.3 | 21.8 | 24.8 | 82.4% | *<0.0001* |
|  | **Grok** | 23.1 | 21.5 | 24.7 | 81.7% | *<0.0001* |
| **WORDS** | **OpenAI GPT-5** | 300.4 | 282.0 | 318.8 | 78.5% | *<0.0001* |
|  | **Claude** | 271.8 | 252.6 | 291.1 | 70.9% | *<0.0001* |
|  | **Gemini** | 277.1 | 257.0 | 297.1 | 72.3% | *<0.0001* |
|  | **DeepSeek** | 296.9 | 277.6 | 316.1 | 77.5% | *<0.0001* |
|  | **Grok** | 277.8 | 256.8 | 298.9 | 72.4% | *<0.0001* |
| **COMPLEX_WORDS** | **OpenAI GPT-5** | 1.2 | -1.2 | 3.6 | -1.1% | *0.3161* |
|  | **Claude** | -2.9 | -5.2 | -0.6 | -29.1% | *0.014* |
|  | **Gemini** | -2.1 | -5.1 | 0.9 | -24.4% | *0.1584* |
|  | **DeepSeek** | -0.9 | -3.1 | 1.2 | -16.7% | *0.3897* |
|  | **Grok** | -4.4 | -7.4 | -1.3 | -46.4% | *0.0065* |
| **Percent_complex** | **OpenAI GPT-5** | -12.1 | -14.0 | -10.1 | -371.5% | *<0.0001* |
|  | **Claude** | -11.7 | -13.3 | -10.1 | -343.9% | *<0.0001* |
|  | **Gemini** | -11.5 | -13.2 | -9.9 | -338.8% | *<0.0001* |
|  | **DeepSeek** | -14.0 | -15.8 | -12.2 | -426.2% | *<0.0001* |
|  | **Grok** | -13.9 | -15.8 | -12.1 | -432.6% | *<0.0001* |
| **AVE_wordsPerSen** | **OpenAI GPT-5** | -6.1 | -7.6 | -4.6 | -45.4% | *<0.0001* |
|  | **Claude** | -9.8 | -11.8 | -7.7 | -73.1% | *<0.0001* |
|  | **Gemini** | -6.4 | -7.8 | -5.1 | -48.2% | *<0.0001* |
|  | **DeepSeek** | -4.7 | -6.4 | -2.9 | -34.8% | *<0.0001* |
|  | **Grok** | -8.6 | -11.0 | -6.3 | -64.3% | *<0.0001* |
| **AVE_sylPer_word** | **OpenAI GPT-5** | -0.4 | -0.4 | -0.3 | -27.1% | *<0.0001* |
|  | **Claude** | -0.4 | -0.4 | -0.3 | -27.0% | *<0.0001* |
|  | **Gemini** | -0.3 | -0.4 | -0.3 | -24.8% | *<0.0001* |
|  | **DeepSeek** | -0.4 | -0.5 | -0.4 | -31.3% | *<0.0001* |
|  | **Grok** | -0.4 | -0.5 | -0.4 | -30.7% | *<0.0001* |
